# Stereochemical Dissection of the Strobilurin PKS Reveals the Complex Biosynthetic Logic of Iterative *EZE* Triene Construction

**DOI:** 10.1002/anie.202519062

**Published:** 2025-12-12

**Authors:** Maurice Hauser, Jing Pang, Daowan Lai, Yunlong Sun, Hao Yao, Russell J. Cox

**Affiliations:** ^1^ Institute for Organic Chemistry and BMWZ Leibniz Universität Hannover Schneiderberg 38 30167 Hannover Germany; ^2^ Department of Plant Pathology College of Plant Protection China Agricultural University Beijing 100193 P.R. China

**Keywords:** *C*‐Methyltransferase, Dehydratase, Ketoreductase, Polyketide Synthase, Stereoselectivity

## Abstract

Type I Iterative polyketide synthases (PKS) use a limited set of catalytic extension and β‐processing domains to create complex polyketides. A remarkable case is that of the strobilurin PKS where a single dehydratase (DH) domain creates an *EZE* triene over three dehydration cycles. Here we dissect the strobilurin PKS and assay catalytic domains individually and in combination with stereo‐defined synthetic substrates in vitro, to reveal the complex and varying selectivities of methylation, ketoreduction and dehydration that lead to this remarkable result. At the diketide stage all stereoselectivities are consistent with those known for other related systems, giving an *E* product. But at the triketide stage, 2*R*‐methylation is selectively achieved, that is followed by rapid keto‐reduction to give an unusual 3‐L‐alcohol. In‐turn, this is eliminated to give the unusual *Z*‐alkene. These selectivities are flexible and change in response to the structure of the substrate at every stage. This uncovers the complete and intricate regio‐ and stereo‐selectivities of a highly reducing iterative Type I PKS for the first time, and highlights the important differences to the well‐studied *cis*‐AT modular PKS β‐processing enzymes that appear to have inflexible selectivities.

## Graphical abstract

The strobilurin iterative PKS assembles a remarkable *EZE* triene by *varying* its stereoselectivity *during* synthesis. Stereoselective 2*R*‐methylation of the triketide intermediate by the C‐terminal *C*‐methyltransferase domain induces the KR and DH domains to invert their native stereoselectivities and produce a *Z*‐configured intermediate.

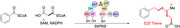

## Introduction

Iterative highly‐reducing polyketide synthases (hr‐iPKS) are remarkable multifunctional mega‐enzymes that are commonly found in fungi. Their protein architectures consist of (from N to C termini) β‐ketoacyl synthase (KS), acyl‐transferase (AT), dehydratase (DH), *C*‐methyl transferase (*C*‐MeT), Ψ‐KR (structural part of the KR), enoyl‐reductase (ER), ketoreductase (KR) and acyl carrier protein (ACP) components. In this respect they are very similar to vertebrate fatty acid synthases (vFAS) that are also iterative systems, but which lack a fully functional *C*‐MeT catalytic domain. Crystal structure (vFAS)^[^ ^1^ ^]^ and cryo electron microscopy (cryo‐EM, hr‐iPKS)^[^ ^2^ ^]^ structures emphasise this similarity. The hr‐iPKS are programmed systems: very many ostensibly similar hr‐iPKS proteins are known; but they are each able to make widely different products. The programme^[^ ^3^ ^]^ of these systems controls starter unit selection, the chain‐length, methylation pattern and functional group pattern of the completed polyketide.

**Scheme 1:**
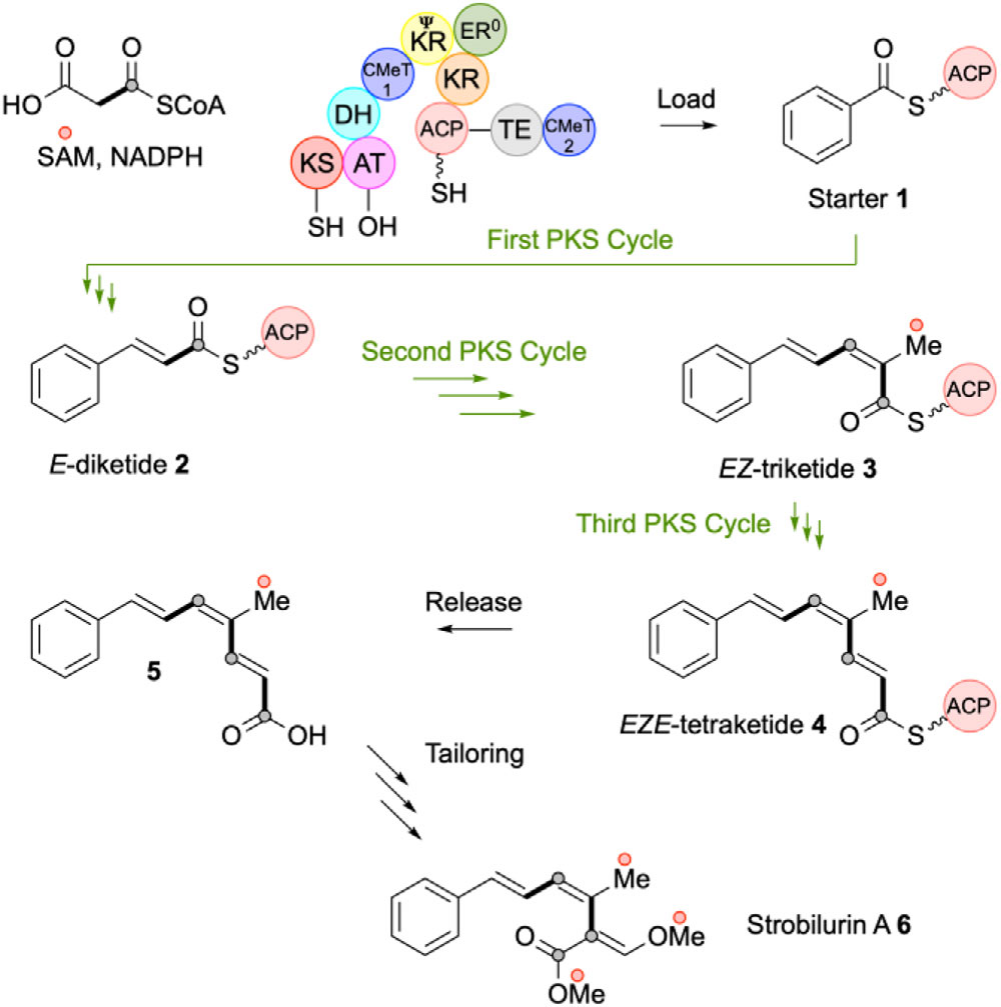
Overview of the biosynthesis of strobilurin A **6**.

The iterative nature of the hr‐iPKS means that the molecular origin of the programming is cryptic. The core KS, AT and ACP domains that are responsible for chain construction are used repeatedly,^[^ ^4^ ^]^ but after each round of construction the β‐processing domains follow a well‐defined programme. For example, during the biosynthesis of the strobilurin A **6** precursor **5**,^[^ ^5^ ^]^ by the 2824‐residue strobilurin hr‐iPKS (strPKS, Scheme 1), benzoyl CoA is selected as the starter unit and loaded onto the ACP. The ACP‐linked benzoate **1** is extended by two carbons (KS, AT) to a diketide, and operation of the KR and DH is thought to give an *E*‐cinnamoyl unit **2**. The strobilurin hr‐iPKS (strPKS) ER domain is known to be non‐functional (ER^0^) and so the enoyl product **2** is the substrate for further chain extension (KS, AT) to a triketide.

After this extension, the β‐processing cycle features *C*‐MeT, then KR and DH reactions. Remarkably, in this cycle, the same DH now appears to afford a *Z*‐olefin intermediate presumed to be **3**. Finally, the chain is extended again (KS, AT) to give a tetraketide, and after this extension the KR and DH now appear to revert to forming an *E*‐olefin likely to be **4**. This is released as the *EZE* triene **5** known to be the precursor of **6** itself.^[^ ^5^, ^6^ ^]^ Thus the strPKS appears to synthesise a remarkable *EZE* tetraketide intermediate using a single set of β‐processing domains. vFAS and hr‐iPKS catalytic domains have previously been shown to be highly stereoselective,^[^ ^7^ ^]^ and so this ability to *vary* the stereoselectivity of dehydration during polyketide biosynthesis is highly unusual.

Previous in vivo experiments had shown the overall function of the strPKS,^[^ ^5^ ^]^ but questions regarding the detailed selectivities displayed during each β‐processing cycle remain. These selectivities could be *intrinsic*, i.e., controlled by the inherent selectivities of the catalytic domains themselves, or *extrinsic*, i.e., controlled by the structure of the growing polyketide, or by a combination of factors.^[^ ^3^ ^]^ We thus set out to interrogate the selectivities of the β‐processing domains *C*‐MeT, KR and DH of the strPKS in vitro using stereo‐defined synthetic substrate analogues, with the aim of discovering key determinants of the stereoselectivity at each stage, and the origin of the remarkable *EZE* unit.

## Results and Discussion

The general strategy was to set up sensitive in vitro assays that measure the reaction of particular substrates with particular enzyme partners. For this purpose potential substrates and products were synthesized de novo as *N*‐acetylcysteamine thiolesters (SNACs, see Supporting Information for all details of synthesis). SNAC mimics the first few atoms of the phosphopantetheine prosthetic group of the *holo*‐ACP. This obviates the need for ACP‐linked substrates and allows for greater turn‐over and detection of assay products by liquid chromatography mass spectrometry (LCMS). The protein components were expressed in *E. coli* and purified before use. Full‐length strPKS could not be obtained, but a large soluble fragment (residues 899–2186, based on an AlphaFold model) could be purified from *E. coli* as an apparently monomeric protein consisting of the DH, *C*‐MeT1, ΨKR, ER^0^, KR and ACP domains. The ACP was shown to be in its non‐phosphopantetheinylated (*apo*) form by MS (see Supporting Information for all details of protein expression, purification and analysis).

The strPKS is unusual in that it contains a second *C*‐MeT downstream of the ACP (residues 2475–2824, Scheme 1).^[^ ^5^ ^]^ We designated this as *C*‐MeT2 and expressed and purified it as a separate protein. Activity assays for all enzyme‐catalyzed reactions were set up in aqueous buffers with the appropriate proteins, substrates and cofactors and incubated at 25 °C. At the end of the incubation time, one volume of CH_3_CN were added to precipitate the protein that was then removed by centrifugation. The supernatant was directly interrogated by LCMS.

Diketide and triketide substrates and products were synthesized as SNAC thiolesters using established methodology (all details in Schemes S3.1.1–S3.1.4). In brief, 2‐methyl‐3‐hydroxy compounds were made using Evans auxiliary chemistry.^[^ ^8^ ^]^ *E* and *Z* alkenes and *EE* and *EZ* dienes were made using Horner‐Wadsworth‐Emmons^[^ ^9^ ^]^ or Still‐Gennari^[^ ^10^ ^]^ chemistry respectively. Relative stereoselectivity was assessed by NMR and comparison to literature precedents and absolute stereochemistry was assessed by optical rotation and comparison to known compounds. The nature of the substituent at C‐3 changes its Cahn‐Ingold‐Prelog stereochemical priorities (*e.g*., CH_3_ versus Ph). The Fischer stereochemical designators at this position are consistent for all substrates and products, and therefore used here for consistency.

### DH Selectivity

**Scheme 2:**
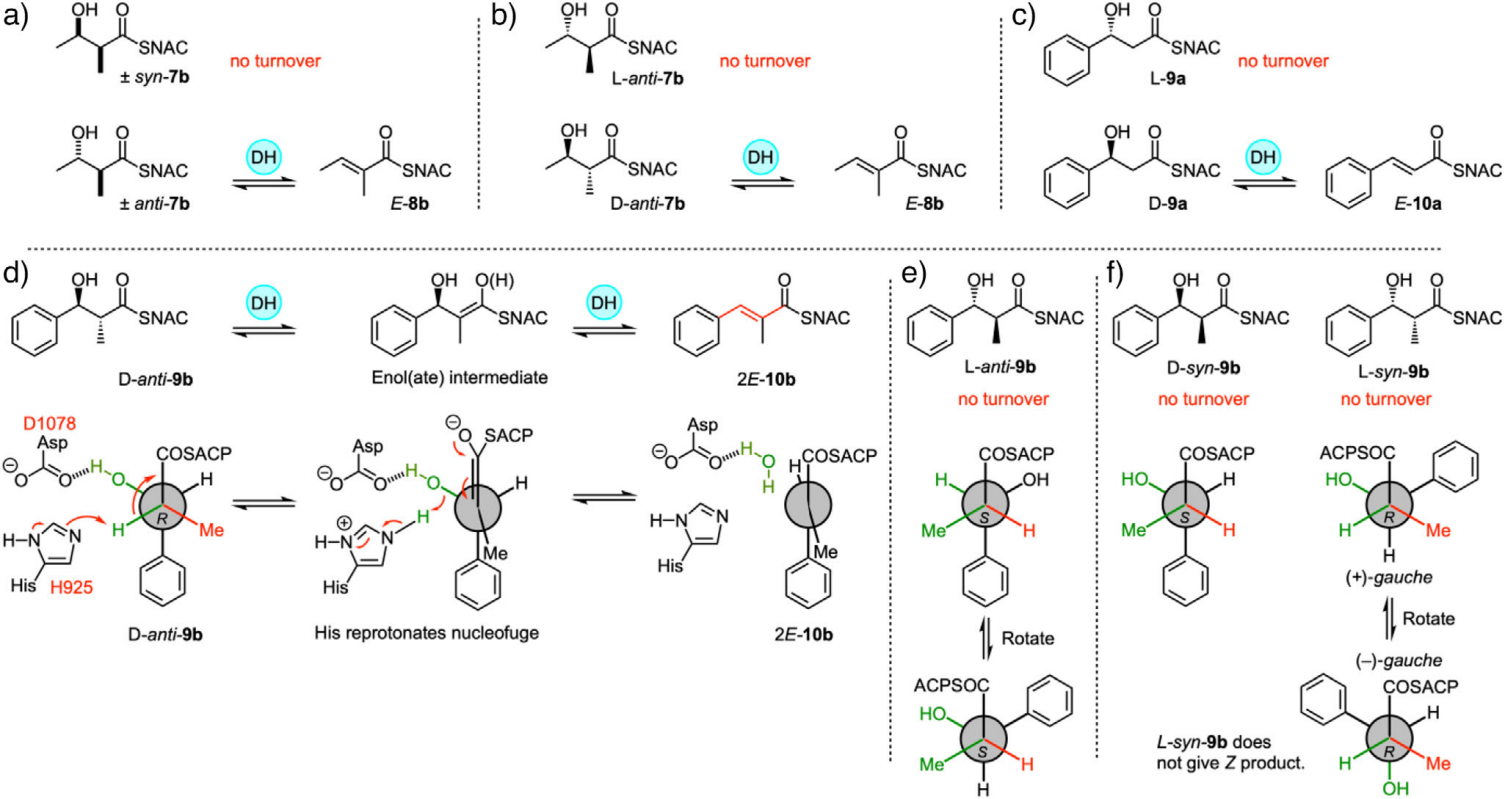
In vitro assays of DH domain with diketide substrates. a) DH is selective for 2,3‐*anti*‐diketides; b) of which only the D‐enantiomer reacts; c) unmethylated diketides are also processed, but only the D‐enantiomer reacts; d) stereochemical analysis of catalysis of D‐*anti* diketide; e) stereochemical analysis of L‐*anti* diketide; f) stereochemical analysis of 2,3‐*syn* diketides. See Figure 1, and Supporting Information for full LCMS analysis.

The DH domain of strPKS (899–1164) was expressed as a soluble monomeric protein in *E. coli*. The protein was active when assayed in vitro, but less active than the DH‐ACP multidomain (StrM) in similar assays. Therefore, all DH measurements were set up using the 1287‐residue StrM protein. In initial reactions the protein was incubated separately with racemic acetate‐derived diketides *syn*‐**7b** and *anti*‐**7b** (Scheme 2a). We had previously used these substrates and developed quantitative assays using the isolated DH domain from the squalestatin tetraketide synthase (SQTKS).^[^ ^7^ ^]^ A product was detected only in the case of the racemic *anti*‐diastereomer. The product was shown to be the diketide *E*‐**8b** by comparison to standard material (Figure S4.2.1A–C for details). Next, the two individual *anti* enantiomers were synthesized^[^ ^7^ ^]^ and tested (Scheme 2b). Only D‐*anti*‐**7b** reacted to give the expected *E*‐**8b** diketide product, in agreement with the previously observed stereoselectivity of SQTKS,^[^ ^7^ ^]^ while L‐*anti*‐**7b** was not a substrate (Figure S4.2.2).

**Figure 1:**
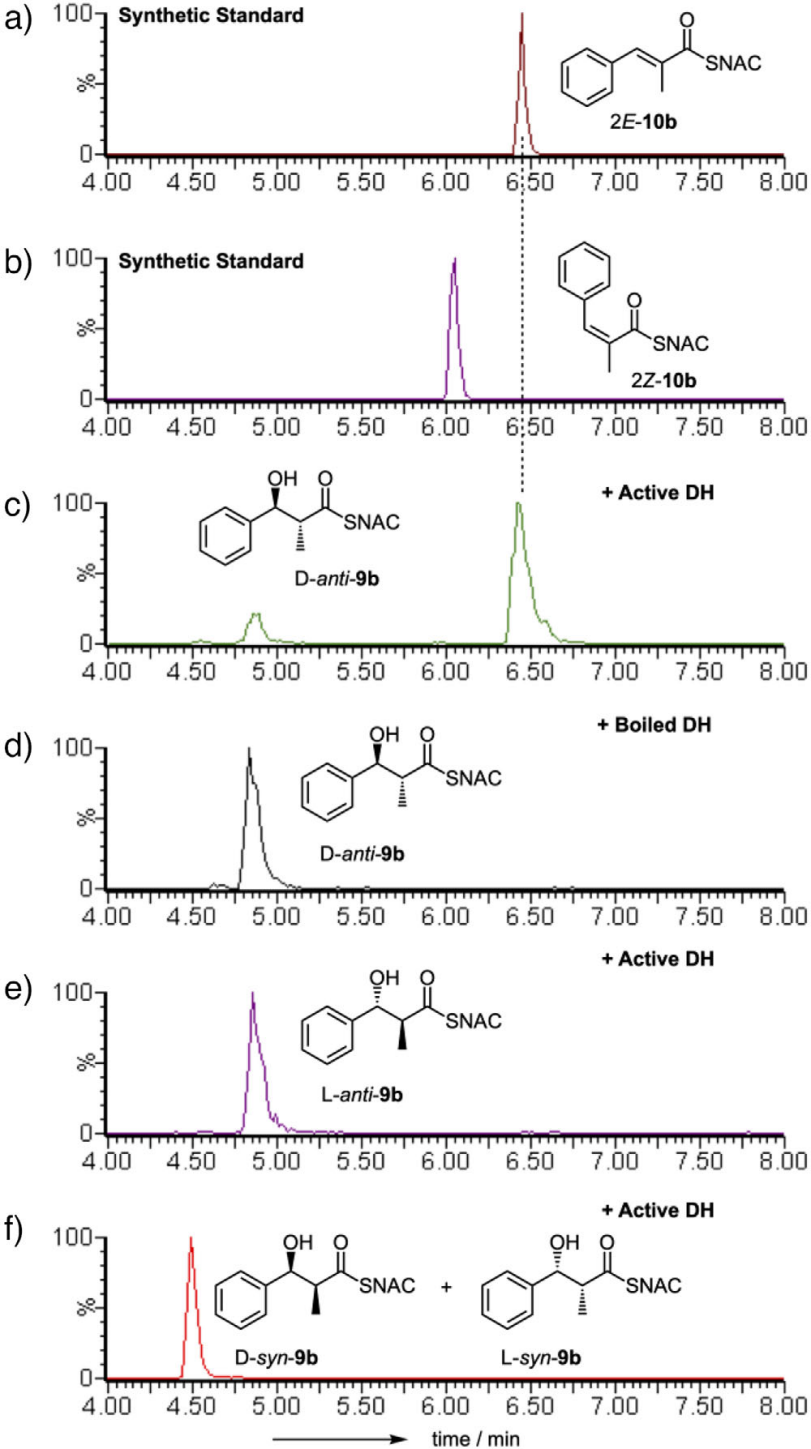
In vitro assays of indicated synthetic diketide substrates with StrM. EIC data in ES + mode at 264.1 Da (alkenes) and 282.1 Da (alcohols). a), b) synthetic standards. c)–f) reactions as indicated. See Figures S4.2.1–S4.2.5 for detailed data & controls.

We then tested substrates derived from the correct benzyl starter unit (Figure 1a–f). Individual enantiomers of 3‐hydroxy‐3‐phenyl propanoyl SNAC L‐**9a** and D‐**9a** were tested first (Scheme 2c, Figure S4.2.3). Only the D‐substrate reacted to give the expected *E*‐cinnamoyl product **10a**. The L‐substrate did not react (see Supporting Information for details), and neither *E‐* nor *Z‐*alkene products were observed. We next synthesized and tested the *syn* and *anti* 2‐methyl diastereomers **9b** (Scheme 2d–f, Figure 1). Once again, only the *anti*‐diastereomer reacted. The D and L *anti* stereoisomers of **9b** were synthesized in enantiopure form. Only D‐*anti*‐**9b** reacted to give the expected *E*‐3‐phenylpropanoyl SNAC product **10b**. L‐*anti*‐**9b** did not react and neither *E* nor *Z*‐ product isomers were observed (Figure S4.2.4).

The results of these assays are consistent with a stereochemical model in which only unmethylated or 2*R*‐methyl substrates can enter the active site productively. The conserved D1078 and H925 active site residues (Figure 3) can then interact with a bound conformation of D‐*anti*‐**9b** in which the phenyl starter unit is *anti* to the thiolester group (Scheme 2d). This places the 2‐hydrogen and 3‐hydroxyl adjacent to the active site residues, allowing *syn* elimination to give the observed *E*‐product.

The L‐*syn*‐**9b** stereoisomer should also be able to bind in the active site (Scheme 2f). Three possible binding‐conformations of L‐*syn*‐**9b** can be envisaged. When the phenyl is *anti* to the 1‐carbonyl, an *anti*‐elimination would be required to give the *E*‐product. This is not observed, suggesting that *anti*‐elimination is not possible, presumably through lack of suitable catalytic residues. The two possible conformers in which the phenyl and carbonyl are *gauche* also appear to be non‐viable (Scheme 2f). In the (+)‐*gauche* conformation, *syn* elimination would be possible using D1078 and H925 to give a *Z*‐product. Elimination from the (‐)‐*gauche* conformer would also give the *Z*‐product, but as this is not observed, we conclude that either the gauche conformers are inaccessible because of lack of a suitable binding‐pocket for the phenyl group, or suitable base catalysis is sterically inaccessible.

**Scheme 3:**
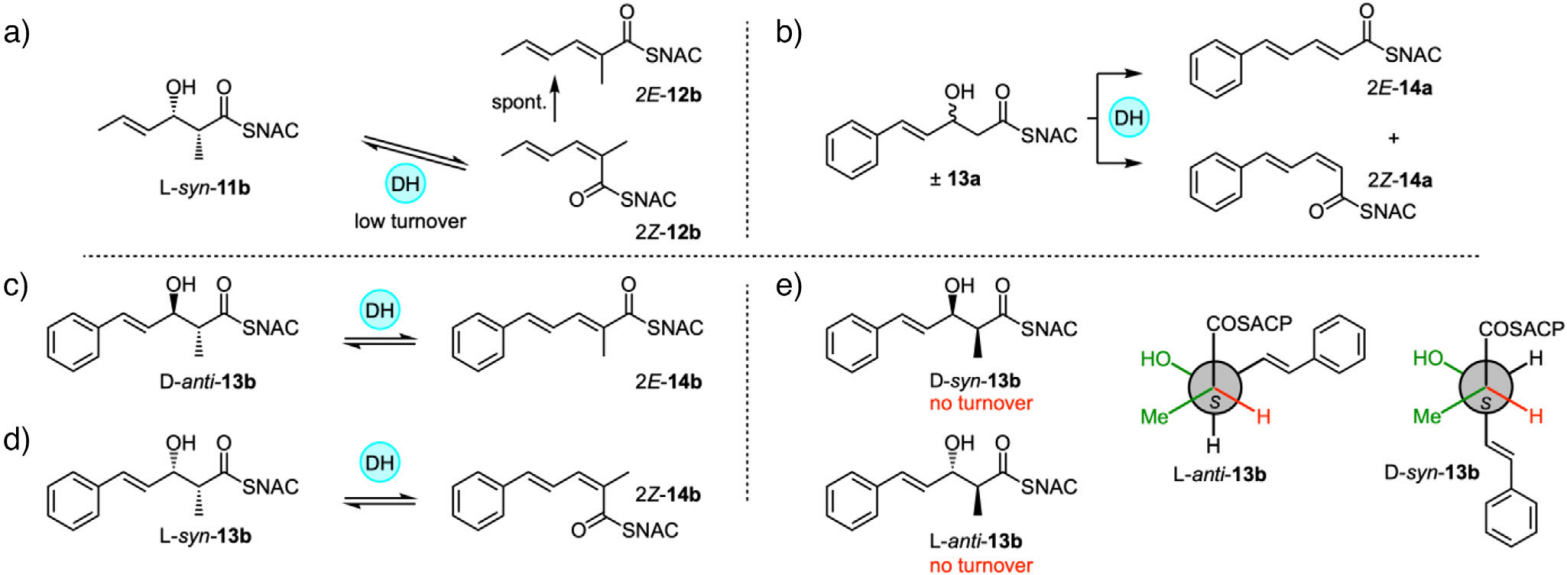
In vitro assays of DH domain with triketides. a) Reaction of acetate‐derived triketide L‐*syn*‐**11b**. b) Reaction of racemic unmethylated triketide **13a**. c) Reaction and stereochemical analysis of methylated triketide D‐*anti*‐**13b**. d) Reaction and stereochemical analysis of methylated triketide L‐*syn*‐**13b**. e) Stereochemical analysis of D‐*syn*‐ and L‐*anti*‐triketides that are not substrates.

Triketide SNAC substrates were also synthesized and reacted with StrM (Scheme 3). In an initial experiment the L‐*syn* triketide alcohol SNAC **11b** derived from an acetyl starter unit was reacted with the DH. This yielded two products that were shown to be the stereoisomeric products 2*E*‐**12b** and 2*Z*‐**12b** by comparison with standard materials (Scheme 3a, Figure S4.2.6). In the case of the unmethylated racemic benzoate‐derived triketide **13a**, both *E* and *Z* isomers of **14a** were also observed (Scheme 3b, Figure S4.2.7).

**Figure 2:**
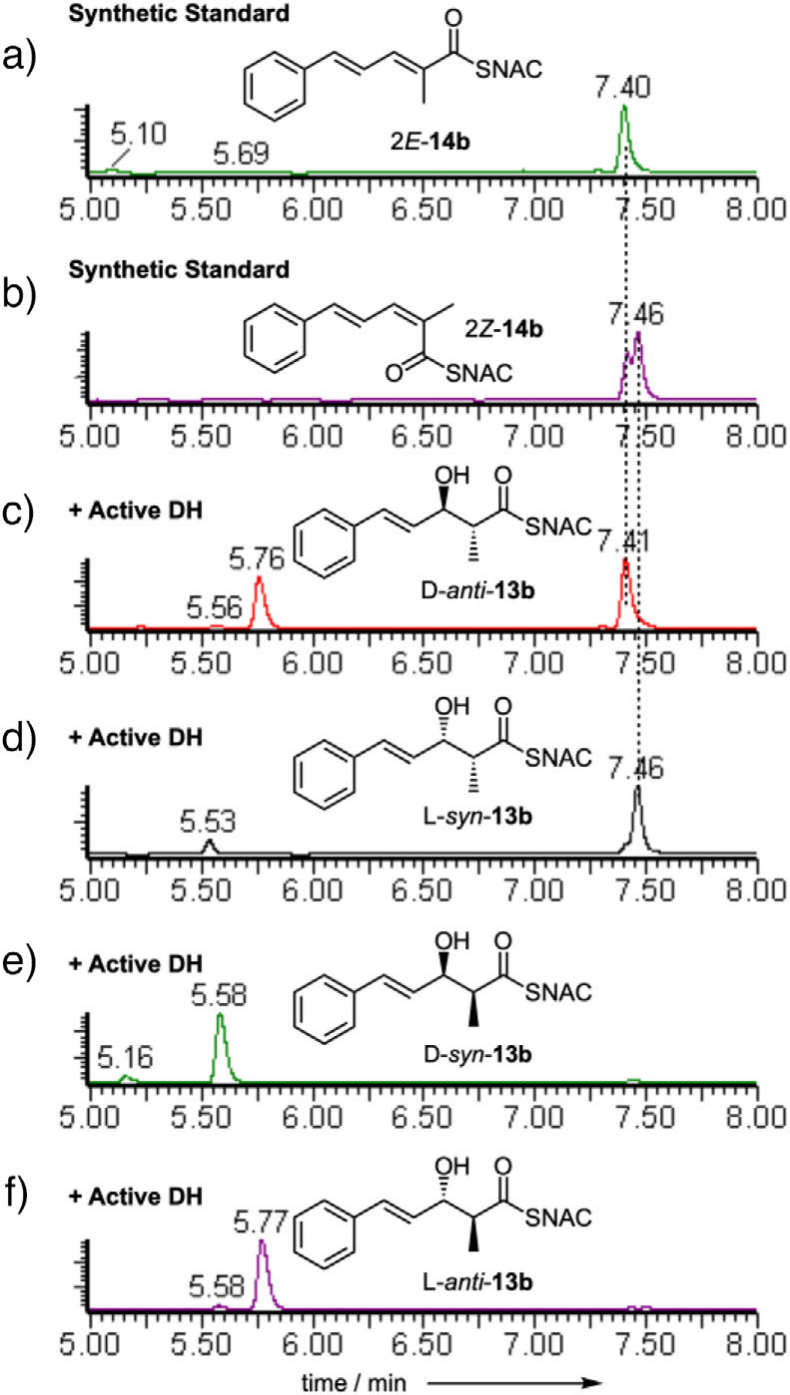
In vitro assays of indicated synthetic triketide substrates with StrM. DAD data (200‐600 nm). a), b) synthetic standards. c)–f) reactions as indicated.

**Scheme 4:**
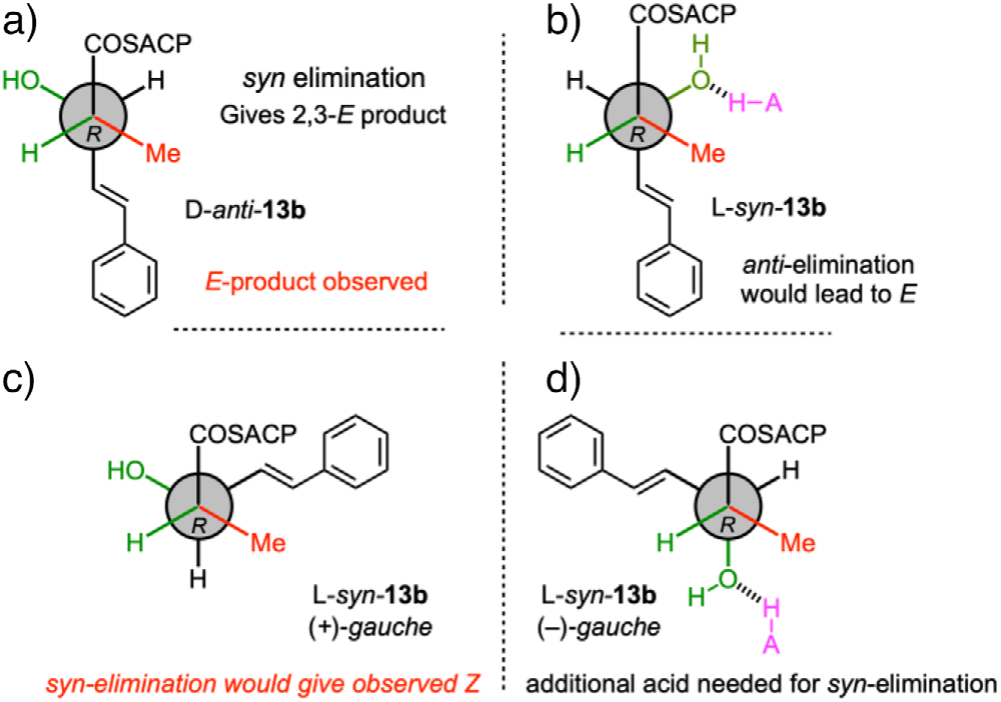
Stereochemical model for triketide elimination. a) Deduced binding mode for D‐*anti* **13b**. b)–d) possible binding modes for L‐*syn*‐**13b**. Only the conformation in c) leads to the observed product.

Finally, all four individual stereoisomers of the 2‐methyl,3‐hydroxy triketides **13b** were synthesized and tested (Figure 2a–f, and Scheme 3c–e, Figures S4.2.8–S4.2.10). Here, we observed that D‐*anti*‐**13b** gave 2*E*‐**14b**, while L‐*syn*‐**13b** gave 2*Z*‐**14b** as the products. Neither D‐*syn*‐**13b** nor L‐*anti*‐**13b** (*i.e*., both 2*S* compounds) reacted under the same conditions. Based on these observed results, we extended the stereochemical model developed for the diketides (Scheme 4). Once again 2*S*‐methyl triketides are not substrates.

The D‐*anti*‐**13b** triketide substrate apparently binds in the same conformation as the D‐*anti*‐**9b** diketide and gives the same observed *E*‐stereochemistry of product (Scheme 4a). The L‐*syn*‐**13b** substrate gives a *Z*‐configured product and this can only arise from a *gauche* conformation (Scheme 4b–d). Of the two possibilities, the (+)‐*gauch*e conformer (Scheme 4c) would preserve the position of the 2‐proton and 3‐hydroxyl for interaction with the active site residues. In the case of the diketide this conformer was not observed (Scheme 2f), presumably because of steric clashes with the phenyl group of **9b**. However, the much smaller CH═CH group of **13b** probably allows productive binding, with a larger pocket beyond to accommodate the phenyl group. Elimination from the (‐)‐*gauche* conformer would not allow D1078 to easily hydrogen‐bond to the nucleofuge. If this were the reactive conformation then an additional acidic residue would likely be required.

**Figure 3:**
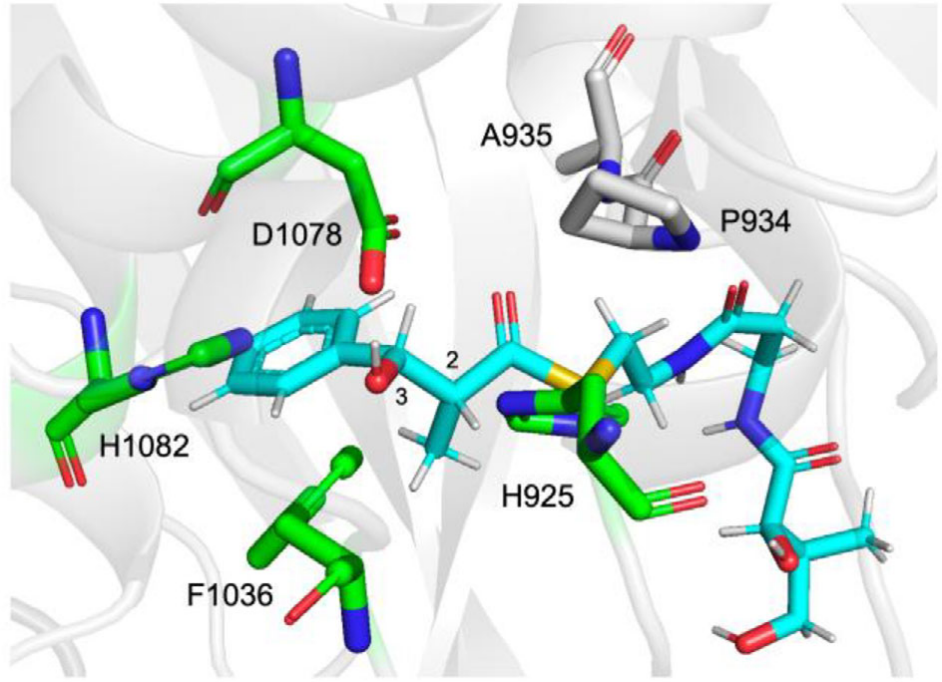
Model structure of DH active site with D‐*anti*‐**9b** (as its pantetheine thiolester) bound.

No structural details are available for any element of the strobilurin hr‐iPKS. We therefore generated a model structure of the DH using AlphaFold3.^[^ ^11^ ^]^ The diketide D‐*anti*‐**9b** was docked into the active site using AutoDock Vina,^[^ ^12^ ^]^ and the resulting structure minimized using the YASARA forcefield.^[^ ^13^ ^]^ The model shows that the thiolester carbonyl is located within hydrogen bonding distance (2.2 Å) of the backbone alanine NH of the conserved P934‐A935 active site motif (Figure 3). Satisfactory docked models of the other stereoisomers or triketides could not be obtained.

At the substrate 2‐position the methyl is accommodated by a hydrophobic pocket formed by I927, A1034, L1085, L1100 and L1158. PKS and FAS DH enzymes are well known to catalyse *syn* E1cb eliminations using a highly conserved active site histidine residue.^[^ ^14^ ^]^ A conserved aspartate or glutamic acid is also found in the active sites, but does not participate directly in catalysis.^[^ ^14^ ^]^ This analysis led to the identification of H925 and D1078 of the strPKS as the likely active site residues. In the model D1078 appears to hydrogen‐bond to the 3‐hydroxyl (1.8 Å), while the substrate 2‐hydrogen lies within 3 Å of the H925 sidechain NH. The geometry is consistent with *syn* elimination to give the observed *E*‐product. The involvement of H925 was confirmed by formation of H925A and H925Q mutants that were both inactive.

In some bacterial modular PKS DH proteins the residue corresponding to F1036 has been shown to be involved in vinylogous rearrangement and elimination reactions, rarely resulting in 2*E‐4Z* dienes (vide infra). In the case of the strPKS DH we created F1036Y and F1036A mutations. There was no change in reaction or selectivity for the F1036Y variant, while the F1036A mutation was inactive. The strPKS DH is also unusual in lacking a glycine residue three positions upstream of F1036 (see Supporting Information section 5.1 for detailed sequence comparisons). We created mutation T1033_A1034:insG to probe this observation. This mutant appeared to slightly increase turnover, but not change selectivity. Finally, examination of the docked model (Figure 3) suggested that H1082 might have potential as an alternative active site base. However, H1082Q, H1082A and H1082F mutations were all inactive.

### KR Selectivity

**Scheme 5:**
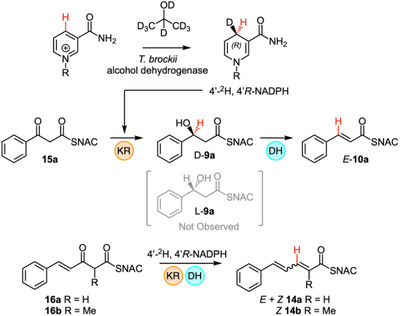
Stereochemical analysis of hydride transfer from NADPH by the strPKS KR domain.

PKS KR domains are classified as A‐, B‐ or C‐types. A‐type KRs give L‐products; B‐type KRs give D‐products; and C‐type KRs are redox inactive. At the outset of this study the stereoselectivity of the strPKS KR was unknown. Two protein constructs were used to assay the strPKS KR activity. In the WT StrM protein both the KR and DH are active; the H925A mutant in which the DH is inactive was used to assay the KR alone. We first determined the stereoselectivity of the KR protein at the cofactor. 4′‐^2^H‐NADPH was prepared from NADP^+^ and isopropanol‐d_8_ in the presence of the *T. brockii* alcohol dehydrogenase (see Supporting Information section 4.7 for MS analysis).^[^ ^15^ ^]^ This enzyme is known to produce the 4′‐*R* cofactor with ^2^H in the *pro‐R* position (Scheme 5).

Incubation of this isotopically labelled cofactor with the diketide **15a** in the presence of H925A StrM yielded a product containing ^1^H rather than ^2^H at the β‐position (MS analysis, Figures S4.7.1–S4.7.3). The methylated and unmethylated triketides **16a** and **16b** were tested in the same way. In the case of the methylated triketide, two 3‐hydroxy diastereomeric products are formed. In both cases no transfer of ^2^H was detected (Scheme 5). Likewise, in the case of the unmethylated triketide **16a** only ^1^H was transferred. This indicates that the KR transfers the 4′‐*pro‐S* hydrogen of NADPH to both diketides and triketides.^[^ ^16^ ^]^

**Scheme 6:**
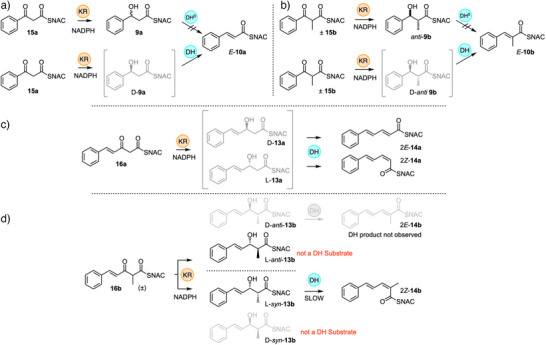
In vitro assays of KR. Grey compounds not observed. Intermediates in brackets must have been present. a) Reaction of unmethylated diketide **15a**. b) Reaction of racemic methylated diketide **15b**. c) Reaction of unmethylated triketide **16a**. d) Reaction of racemic methylated triketide **16b**.

Using H925A StrM, the unmethylated diketide **15a** was converted to the corresponding alcohol **9a**, although its absolute stereochemistry could not (yet) be determined (Scheme 6a, Figure S4.3.2). In the case of the methylated diketide **15b**, only *anti‐* **9b,** was detected by LCMS when using H925A StrM (Scheme 6b, Figures S4.3.3A,B).

We next ran the same assays in the presence of the fully active StrM protein (*i.e*., both KR and DH active). In the case of the unmethylated diketide **15a** only *E*‐cinnamoyl SNAC *E*‐**10a** was observed (Scheme 6a), and L‐**9a** that we know is not a substrate for the DH (Scheme 2c) was not observed to accumulate. In the case of the methylated diketide **15b**, the product *E‐* **10b** was observed by LCMS. Since we had already determined the stereoselectivity of the DH domain, these results show that both diketides **15a** and **15b** are processed by the KR to give only D‐configured products (Scheme 6a,b) and that for diketides the strPKS is B‐type.

We next tested triketide substrates. The unmethylated triketide **16a** gave a mixture of 2*E* and 2*Z* isomers of **14a** (Scheme 6c, Figures S4.3.4A,B) with WT StrM that must arise from a mixture of D‐ and L‐configured alcohols (Scheme 6c). Alcohol intermediates were not observed by LCMS, indicating that the DH reaction is relatively fast in this case, and intermediates do not accumulate. This shows that for unmethylated triketides the KR displays ***a mixture*** of A‐type and B‐type reduction.

The methylated triketide (±)‐**16b** gave only a single diastereomeric product that was shown to be 2*Z*‐**14b** by comparison to a synthetic standard (Scheme 6d, Figures S4.3.5A–F). In this case we also detected both L‐*syn*‐**13b** (a known DH substrate) and L‐*anti*‐**13b** (not a DH substrate) alcohol intermediates. This was confirmed by Mosher's analysis (see Supporting Information) indicating that the DH reacts relatively slowly with this series of substrates and the intermediate alcohols accumulate. From this experiment we conclude that the KR domain would normally only ever encounter 2*R*‐**16b** as its true substrate, giving L‐*syn*‐**13b** as the on‐pathway intermediate (Scheme 6d). This also shows that for the methylated triketide substrate the KR shows A‐type selectivity.

The stereoselectivity of KR domains has been studied by X‐ray crystallography for bacterial *cis*‐AT modular PKS. For example, *cis*‐AT PKS KR domains that specify B‐type reduction (i.e., give D‐alcohols, see Scheme 8 for details) are known to possess a 3‐residue “I/L/V‐X‐D” motif in the protein's loop region, and usually lack a tryptophan in the catalytic region.^[^ ^17^ ^]^ In contrast, *cis*‐AT modular KRs that catalyze A‐type reduction (i.e., give L‐alcohols) lack the LXD motif and usually do possess a W residue in the catalytic region.^[^ ^17^, ^18^ ^]^

Although the fungal iterative PKS KR sequences are distantly related to the modular *cis*‐AT PKS KRs, sequence and structure alignment of the strPKS KR domain with *cis*‐AT modular KR domains (Supporting Information section 5.2) indicates the presence of sequence motifs that predict B‐type reduction as observed for diketides.

Although weak, these motifs exist as 1942‐MAD‐1944 in the loop region and lack of a conserved W in the catalytic region. In vFAS, to which strPKS shows high homology and that is also a B‐type reductase, this sequence is LRD and W is again missing from the catalytic region. We investigated the role of these residues in the stereoselectivity of the strPKS KR by the creation of three mutants: M1942L; A1943R; and F1990W.

These mutants were assayed with diketide (±)‐**15b** and triketide (±)‐**16b**. No changes in selectivity were observed for M1942L for either substrate; while F1990W was inactive for both substrates. Mutant A1943R also showed no change in selectivity for the diketide, but with the triketide (±)‐**16b**, both products *E*‐**14b** and *Z*‐**14b** were observed (Figure S4.5.1). This must be because this mutant can now produce *both* L‐*syn*‐**13b** *and* D‐*anti*‐**13b** intermediates (see Scheme 6d). In contrast, the WT protein makes only L‐*syn*‐**13b**. This result shows that the A1943R mutation changes the native selectivity for methylated triketides from A‐type to a mix of A‐ and B‐types.

### *C*‐MeT Selectivity and Epimerization

**Scheme 7:**
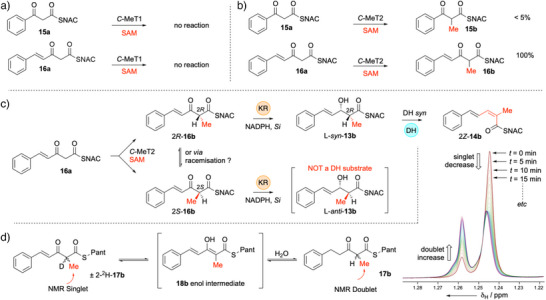
In vitro methylation assays. a) *C*‐MeT1 is inactive. b) *C*‐MeT2‐ is active and selective for triketide substrates. c) Stereochemical analysis of the *C*‐MeT2‐KR‐DH reaction sequence for triketides. d) ^1^H NMR assay to determine ability of strPKS to racemise methylated triketides.

*C*‐MeT2 was cloned as an individual catalytic domain (residues 2475–2821) and expressed in *E. coli* as an N‐terminal hexa‐his‐tagged monomeric protein and purified in the usual way. *C*‐MeT1 was assayed as part of WT StrM. Isothermal titration calorimetry (ITC) showed that StrM, containing *C*‐MeT1, does not bind SAM, but *C‐*MeT2 binds SAM with a *K_d_* of 33 µM (Supporting Information). In initial reaction assays, *C*‐MeT1 was incubated with diketide **15a** or triketide **16a** in the presence of SAM and absence of NADPH (i.e., KR is inactive). Protein was precipitated and the progress of reaction was examined by LCMS. Neither substrate was methylated in these assays (Scheme 7a, Figure S4.4.1).

However, under identical conditions the triketide **16a** was converted fully to its methylated product **16b** by *C*‐MeT2. Control reactions lacking SAM or with boiled protein did not afford any product. In the case of diketide **15a**, LCMS analysis did detect the methylated diketide **15b**, but at less than 5% the ion intensity of the triketide under identical conditions (Scheme 7b, Figure S4.4.1). These results agree with sequence analysis that suggests that a proposed active site basic histidine^[^ ^19^ ^]^ is replaced by G1366 in *C*‐MeT1 (Supporting Information section 5.3). In contrast, the non‐canonical *C*‐MeT2 is active in vitro, and this correlates with the expected presence of H2723 in the active site motif of this domain.

**Figure 4:**
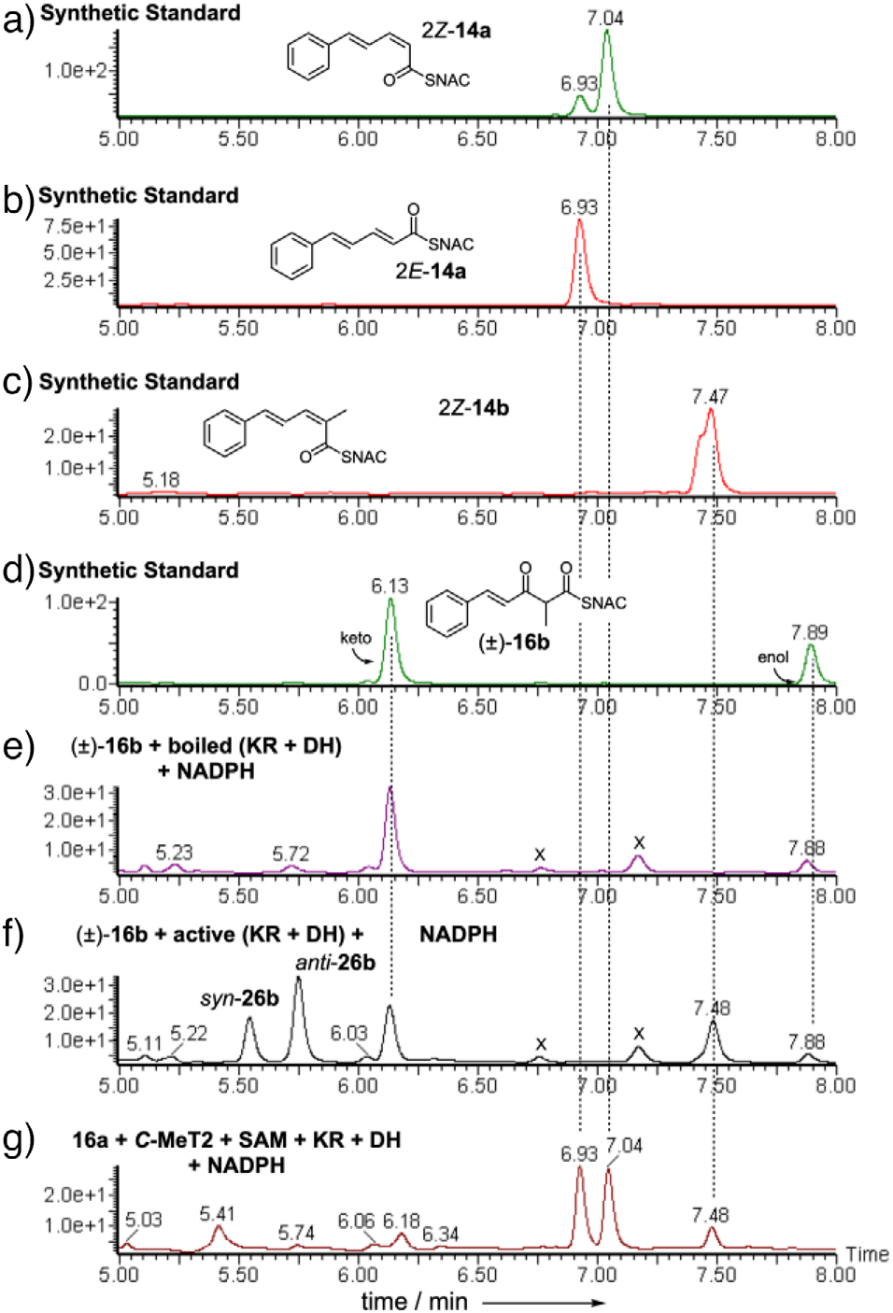
In vitro assays of indicated synthetic triketide substrates with StrM. DAD data (200‐600 nm). **a)**‐**d)** synthetic standards; **e)** (±)‐**16b** plus boiled proteins results in no reaction; **f)** (±)‐**16b** plus active KR + NADPH + DH proteins give 2*Z*‐**14b** and alcohol intermediates L‐*syn‐* **13b** and L‐*anti*‐**13b**; **g)** **16a** + *C*‐MeT2 + SAM generates **16b** in vitro. **16a** gives 2*E*‐**14a** and 2*Z*‐**14a** while **16b** gives 2*Z*‐**14b**. Intermediates not observed when *C*‐MeT2 is present. X = unrelated compound.

The stereoselectivity of the strPKS *C*‐MeT2 is unknown. Logically, since the DH only accepts 2*R*‐methylated substrates, then the KR must produce 2*R* products in the WT strPKS, and presumably therefore accepts 2*R* substrates. In the assay of the KR alone using *racemic* **16b**, *both 2R‐* and 2*S‐* **16b** were reduced producing both L‐*anti*‐**13b** and L‐*syn*‐**13b** triketides (Scheme 6c). L‐*syn‐* **13b** is dehydrated to form 2*Z*‐**14b**, but the L‐*anti* diastereomer accumulates because it is not a DH substrate. We probed this point further by setting up in vitro assays in which triketide **16a** was incubated with *C*‐MeT2, SAM, StrM and NADPH (Scheme 7c, Figure 4a–g). As expected, this produced 2*Z*‐**14b** and the two non‐methylated triketide dienes 2*E*‐**14a** and 2*Z*‐**14a** that come from direct reduction of **16a** and subsequent dehydration (*e.g*., Scheme 6c). However, intermediate alcohols were not observed, indicating that *C*‐MeT2 produces the expected 2*R*‐methyl product **16b** that is rapidly reduced by the KR to give L‐*syn*‐**13b** that is, in‐turn, dehydrated to 2*Z*‐**14b**. If 2*S*‐**16b** had been produced then this would have been reduced by KR to give L‐*anti*‐**13b** that should have accumulated in the assay, but this was not observed.

An alternative explanation would be that *C*‐MeT2 produces 2*S*‐**16b** that could be epimerized by some part of the PKS, providing 2*R*‐**16b** to the KR. Some modular PKS KR domains are known to epimerise their substrates (e.g., A2 and B2 types).^[^ ^17^, ^20^ ^]^ Sequence analysis suggests that the strPKS KR is a non‐racemising B1‐type (i.e., no proline in the catalytic region),^[^ ^17^ ^]^ although some other protein component of strPKS might catalyse this activity. To probe this point we synthesized triketide ketone 2–^2^H‐**17b** as its pantetheine thiolester and measured its epimerization rate (Scheme 7d, Supporting Information Section 4.4.3) in unlabelled buffer. Pantetheine was selected instead of SNAC because its increased solubility in aqueous buffer allowed the direct use of ^1^H‐NMR to follow the epimerization at the 2‐carbon. In initial assays 2–^2^H‐**17b** was incubated in standard assay buffer at pH 7.1. The background epimerization reaction in reaction buffer was observed in the ^1^H NMR spectrum as the singlet corresponding to the 2‐methyl of 2–^2^H‐**17b** slowly changed to the doublet of unlabelled **17b** over *ca* 2h. The apparent half‐life for exchange was in excess of 30 min, in agreement with the observation that keto and enol tautomers of **16b** are separable by HPLC (Figure 4). Then, the same reaction was monitored in the presence of either StrM or *C*‐MeT2. The experiments were repeated with or without appropriate cofactors. In no case was the epimerization rate observed to significantly increase (Supporting Information Section 4.4.3).

Interestingly, we observed a significant difference in the outcome of KR + DH reactions starting from (±)‐**16b** versus using **16a** + *C*‐MeT2 + SAM. In the first case the reaction proceeded slowly, and *syn‐* and *anti*‐alcohol intermediates accumulated. However, in the latter case intermediates did not accumulate suggesting that 2*R*‐**16b** is more rapidly processed. This may be explained if either 2*R*‐**16b** itself, or its KR product L‐*anti*‐**13b** is an inhibitor of the DH. In order to test this hypothesis DH assays were set up containing either **16a**, (±)‐**16b** or L‐*anti*‐**13b**. After 8 h the amount of *Z*‐**14b** was quantified by peak integration (Supporting Information Section 4.6) versus the control reaction. These assays showed that the presence of (±)‐**16b** reduces product formation by 80%, while the presence of **16a** or L‐*anti*‐**13b** reduce product formation by 50%.

The full stereochemical programme of the iterative strPKS is now revealed for the first two β‐processing cycles. The first possible reaction after chain extension is methylation. *C*‐MeT1, the canonical methyltransferase of strPKS, is inactive in vitro with both diketide and triketide substrates, consistent with key active site mutations that prevent SAM binding. However, the non‐canonical *C*‐MeT2 is active and selective for triketides. Vederas, Tang and coworkers observed similar behaviour in the case of the lovastatin PKS,^[^ ^21^ ^]^ although here it is the canonical *C*‐MeT1 that is selective for methylation at the tetraketide stage.

The *C*‐MeT2 domain appears to specify 2*R* products in agreement with a previous study by Cane and coworkers that showed that bacterial *trans*‐AT PKS *C*‐MeT domains show the same stereoselectivity,^[^ ^20^ ^]^ and our previous studies of canonical iterative Type I PKS components that also supported 2*R* methylation.^[^ ^7^ ^]^ The strPKS KR can reduce both 2*R* and 2*S* methylated substrates, but the 2*S*‐products cannot be turned‐over by the downstream DH and therefore 2*S* methylated compounds cannot be intermediates. Since neither *C*‐MeT2 nor StrM can racemise 2‐methyl‐3‐keto substrates (e.g., **17b**) faster than the slow background rate, the 2*R*‐methylated triketide must be the direct product of methylation. It is noteworthy that in strPKS the methylation is actually performed by the appended *C*‐MeT2. This does not appear to be for stereochemical reasons, but it is likely because the *regioselectivity* of *C*‐MeT2 for triketides is key for the later stereoselectivity of the DH domain.

**Scheme 8:**
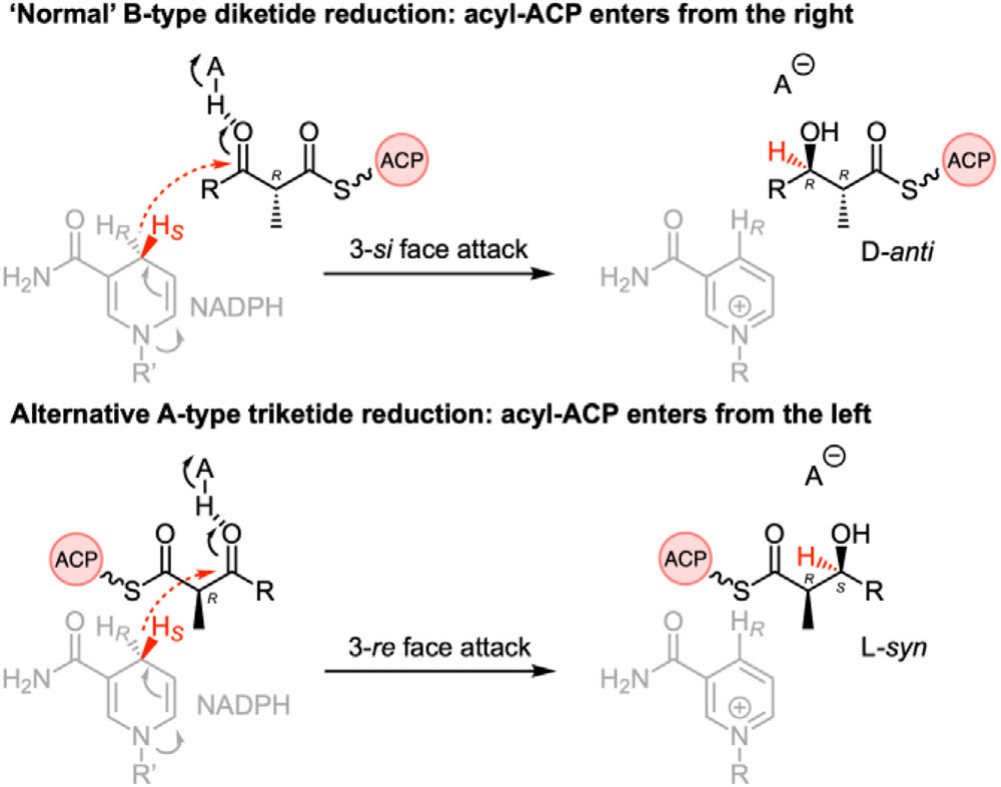
Stereochemical analysis of substrate reduction by the KR domain.

The next reaction in series is catalyzed by the KR domain. We showed that the 4′‐*pro‐S* hydrogen of NADPH is transferred during reduction, consistent with the known cofactor stereoselectivity of the vFAS^[^ ^1^, ^16^, ^22^ ^]^ and the few fungal PKS that have been studied in detail.^[^ ^2^, ^23^ ^]^ These KRs are known to be B‐type SDRs that give D‐alcohol products. B‐type KRs are known to be indicated by a conserved I/L/V‐X‐D sequence motif in modular *cis*‐AT PKS KR domains that corresponds to M‐A‐D in the strPKS KR sequence (Supporting Information Section 5.2). The 4′‐*pro‐S* hydrogen of NADPH is transferred to both diketide and triketide substrates showing that the cofactor does not change its position in the active site for different chain‐length substrates. Many KR enzymes are also known that display A‐type reduction to give L‐alcohol products. The change in selectivity is achieved by the substrate entering the KR active site from the *opposite direction* to that observed for B‐type KRs (Scheme 8). A‐type KRs are indicated by lack of the I/L/V‐X‐D sequence motif. In bacterial modular *cis*‐AT PKS, KR sequence motifs are good predictors of A‐ or B‐type selectivity.

In the case of unmethylated triketide **16a** the strPKS KR shows a ***mixture*** of B‐type and A‐type selectivities that most‐likely arise from the triketide substrate being able to enter the active site from *either direction* with low selectivity. An alternative mechanism could involve the substrate entering from the same direction, but rotating by 180° to present the opposite face of the ketone to the 4′‐*pro‐S* hydrogen of NADPH. This seems unlikely as the conserved tyrosine (AH in Scheme 8) that polarises the carbonyl for reduction could not then function in this way, and no alternative polarising group appears to be present. Remarkably, when the triketide is 2‐methylated to **16b**, the selectivity improves so that only A‐type selectivity is observed and only the L‐product geometry is produced. This change in selectivity in response to substrate structure is the key catalytic difference between the fungal iterative PKS studied here and the much‐more well‐known bacterial *cis*‐AT modular KR domains.

This ability of iterative fungal KR domains to be able to alter their selectivity in response to the changing chain‐length of the growing polyketide has been rarely observed before. For example during the biosynthesis of hypothemycin the KR displays A‐type reactivity for diketide substrates and B‐type reactivity for tetraketides.^[^ ^24^ ^]^ However the precise molecular determinants of this varying selectivity are not yet known due to the lack of detailed structural information. Here we mutated the M‐A‐D sequence motif. M1942L made no selectivity difference, unsurprisingly for a conservative change. However A1943R, reflecting the vFAS B‐type motif, did observably change the stereoselectivity to be more B‐type, although did not completely reverse the selectivity (Figure S4.5.1). We also previously showed that mutations in the KR loop region of the tenellin iterative Type I hr‐PKS can modify the rate of KR‐catalyzed reaction, altering competition with chain‐extension (i.e., KS) and termination (*i.e*., NRPS) and thus influencing chain‐length programming by the PKS.^[^ ^23^ ^]^ It seems clear that fungal PKS KR domains, and KR domains in general, likely have significantly more substrate selectivity flexibility than has been hitherto appreciated or directly assayed. This conclusion is emphasized by the results of Hahn and coworkers who have recently shown flexibility of stereoselectivity in bacterial modular PKS KR domains.^[^ ^25^ ^]^

The final layer of stereoselectivity is enacted by the strPKS DH domain. PKS and FAS DH domains have been long known to proceed via an E1cb‐type mechanism that, to‐date, always involves a 2,3‐*syn* elimination of water.^[^ ^14^ ^]^ The origin of the stereochemical preferences of DH domains from bacterial modular PKS have been well studied, such that these enzymes have found application in kinetic resolution, for example.^[^ ^26^ ^]^ The strPKS DH appears no different in the case of diketides. For acetate‐derived diketides (e.g., **7b**) the observed selectivity is identical to that observed in the SQTKS DH, that was shown to be, in turn, identical to the classical selectivity shown by vFAS DH domains,^[^ ^16^, ^22^ ^]^ and the typical modular PKS DH EryDH4.^[^ ^27^ ^]^ Only the D*‐anti* stereoisomer of **7b** can be processed to give the 2*E* olefinic product. The other three stereoisomers are not substrates – the same selectivity observed for vFAS,^[^ ^16^, ^22^ ^]^ the SQTKS DH^[^ ^7^ ^]^ and eryDH4.^[^ ^27^ ^]^ This selectivity remains the same for the benzoate derived diketides **9b** where only the D*‐anti*‐**9b** substrate can be processed.

However, in the case of strPKS, the strict selectivity is relaxed for triketides. The DH is sensitive to the presence of a methyl group at the 2‐position. In the absence of a 2‐methyl, racemic substrate gives both *E‐* and *Z*‐products consistent with a stereospecific dehydration in which D‐alcohols give *E*‐olefins and L‐alcohols give *Z*‐olefins. If a 2‐methyl is present, only 2*R*‐substrates can be processed: again, D‐2*R*‐alcohols (D‐*anti*) give *E*‐olefins and L‐2*R*‐alcohols (L‐*syn*) give *Z*‐olefins. The 2*S* epimers cannot be dehydrated.

**Scheme 9:**
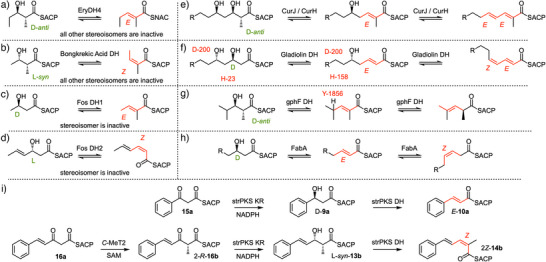
Known stereoselectivities of PKS and FAS DH domains in the: a) erythromycin; b) bongkrekic acid; c), d) fostriecin; e) curacin; f) gladiolin; g) gephyronic acid; and h) fatty acid synthase systems. Implicated residues are indicated in red where known. i) deduced stereochemical course of the strPKS for diketides and triketides.

**Figure 5:**
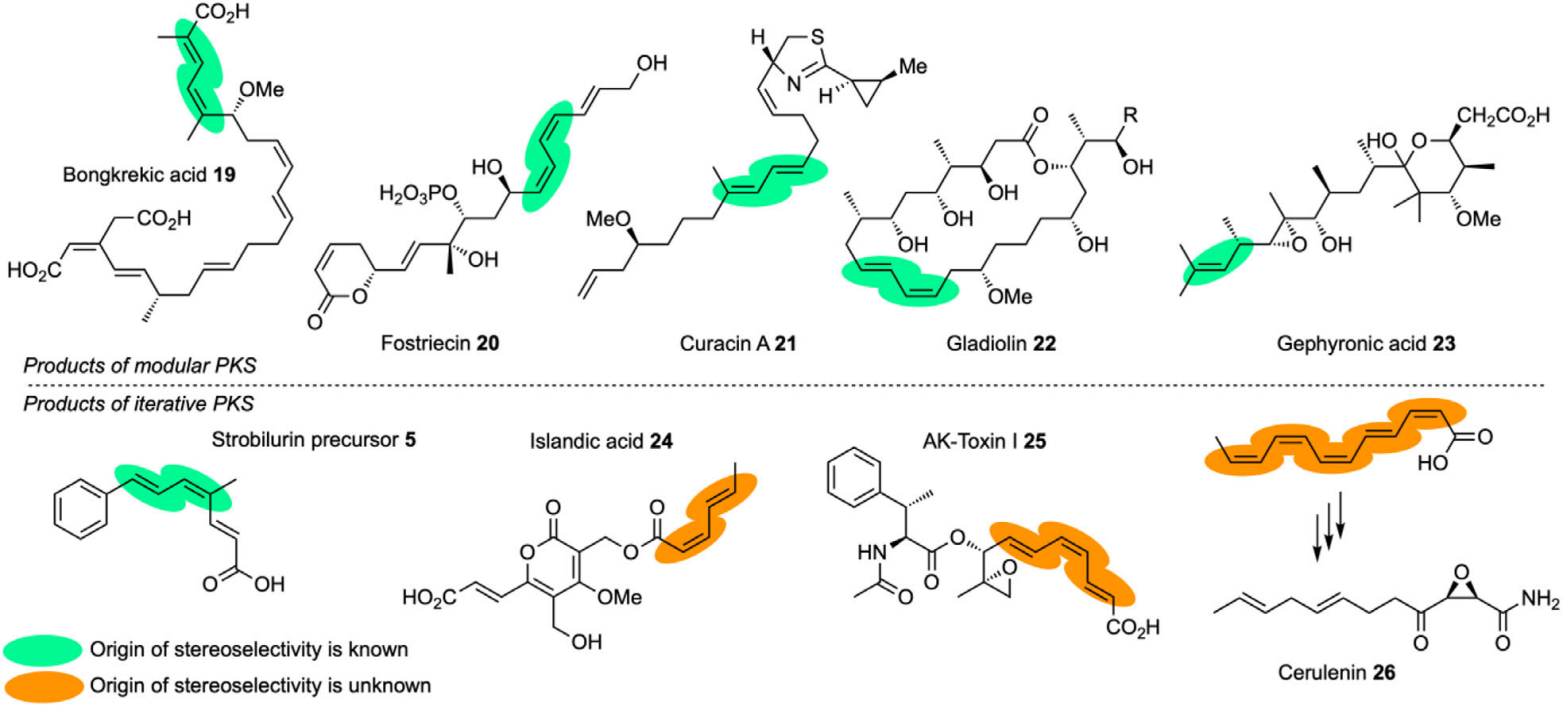
Structures of polyketides with isomerized olefins discussed in the text.

DH domains with different selectivities are known. For example EryDH4 selectively catalyses elimination from a D‐*anti* substrate to give an *E* product (Scheme 9a, Figure 5).^[^ ^27^ ^]^ In the case of bongkrekic acid **19** DH, the L‐*syn* substrate gives the *Z*‐product in accordance with the stereospecific *syn* elimination of water (Scheme 9b).^[^ ^28^ ^]^ However, neither of these DHs can catalyse the reaction of alternative stereoisomers. In non‐2‐methylated cases, the fostriecin **20** DH1 and DH2 domains have been shown to catalyse stereospecific reactions: FosDH1 eliminates water from a D‐diketide to give an *E*‐product; and FosDH2 eliminates an *L*‐triketide to give a *Z*‐product (Scheme 9c,d).^[^ ^29^ ^]^ Again, neither FosDH can catalyse reactions of alternative stereoisomers. DH enzymes are also known that can catalyse *vinylogous* eliminations. For example, the CurJ and CurH DH domains from the curacin **21** modular PKS catalyse sequential eliminations from 3,5‐diol substrates to generate *EE* dienes (Scheme 9e).^[^ ^30^ ^]^ Recently an *EZ* diene has been shown to be created by an analogous DH from the gladiolin **22** modular PKS (Scheme 9f).^[^ ^31^ ^]^ Finally, DH enzymes are known to be able to isomerise 2‐enes to 3‐enes in the cases of the bacterial Type II FAS DH FabA^[^ ^32^ ^]^ and the modular PKS DH from the gephyronic acid **23** PKS (Scheme 9g,h).^[^ ^33^ ^]^

The strPKS DH is different to all of these known cases because it *changes* its selectivity with the chain‐length and methylation of the substrate, and can catalyse the formation of *both E* and *Z* olefins. We investigated its relationship to the other types of DH through selective mutation experiments. As expected the conserved active site H925 is required for reaction. F1036 is an interesting residue because this position is often tyrosine in many other DH enzymes and is histidine in the gladiolin **22** DH (Supporting Information Section 5.1). In the case of the gladiolin **22** DH, Challis and coworkers showed that this histidine could act as a second base, driving the formation of the *Z*‐olefin.^[^ ^31^ ^]^ However, in our case F1036A, and dual mutation F1036H/H1082F that mimics the gladiolin DH active site, were inactive for all reactions, giving no useful information. Mutant F1036Y was active, but showed no selectivity differences to WT protein. Our conclusion is that this residue likely fulfils a structural role in the strPKS DH active site, orientating the substrate, but not taking part in chemical reaction. Mutation F1036Y was also interesting to us because Y at this position in the gephyronic acid DH was implicated in the olefin isomerization.^[^ ^33^ ^]^ However we did not observe isomerization activity in this mutant.

## Conclusion

The results of our in vitro assays show how the programming of the β‐processing reactions catalyzed by the iterative strPKS are orchestrated by a complex *and varying* interplay of selectivities (Scheme 9i). The fully‐determined β‐processing pathway starts from the diketide ketone **15a**. At this stage there is competition between α‐methylation and β‐reduction. It appears that the β‐reduction to D‐**9a** is faster than methylation and this occurs with B‐type stereoselectivity. The KR can reduce methylated diketides, even though these are not normally formed showing that it must be kinetic competition of the *C*‐MeT2 and KR that ensures diketides are not methylated. The DH then eliminates to form the *E*‐cinnamoyl diketide **10a**. As strPKS has an inactive ER, the ACP‐bound intermediate is extended again to form triketide **16a**. Again, at this point there is possible competition between methylation and reduction, and here *C*‐MeT2 methylates to give a 2*R*‐intermediate **16b**. The KR then reduces this to give L‐*syn*‐**13b**, now with A‐type selectivity. The DH then proceeds to catalyze a 2,3‐*syn* elimination to give the observed *Z*‐product **14b**. Again, the selectivity alters in response to the substrate structure. Thus, the *Z*‐selectivity of the DH at this stage is a response to the earlier stereoselective methylation by *C*‐MeT2 and consequent L‐selective reduction of the triketide ketone by the KR, rather than an intrinsic ability to form *Z*‐olefins. Although we did not test tetraketide substrates, it seems likely that the lack of activity by *C*‐MeT2 with a tetraketide substrate then allows the system to form the final *E*‐olefin after reduction and dehydration.

Other fungal polyketides that are made by iterative PKS are known where unusual polyene geometries are key structural features, for example in the cases of the cytotoxin islandic acid **24**,^[^ ^34^ ^]^ the phytotoxin AK‐toxin **25** ^[^ ^35^ ^]^ and the remarkable *ZEZZZ* precursor of cerulenin **26**.^[^ ^36^ ^]^ In all these cases it seems highly likely that varying stereoselectivity by KR domains results in subsequent *E* or *Z* selectivity by the DH. However, all of these cases differ from the strPKS where the alteration in KR and DH stereoselectivity is initiated by 2*R*‐methylation at the triketide stage. In the cases of **24**–**26** there is no methylation, and so the stereoselectivity is likely controlled by very precise chain‐length detection by the KR, or by isomerization. In the case of the strPKS, and many others, the ER domain is clearly inactive, allowing the production of polyenes. However, in iterative PKS that contain a functional ER domain the geometry of the intermediate olefin, and hence the stereoselectivities of the DH and KR, remain cryptic. We previously showed that *Z*‐olefins can be reduced by the ER domain of the squalestatin PKS.^[^ ^37^ ^]^ Our work here shows that the stereoselectivities of iterative PKS domains should no longer be assumed to follow the known stereochemical choices of the vertebrate FAS. The results of the in vitro assays also illustrate that the individual catalytic domains of the strPKS possess broad substrate selectivity.

For example, the strPKS KR and DH domains would not normally encounter 2‐methylated diketide substrates, or substrates lacking an aromatic starter unit, but both domains can process these intermediates. Similarly, unmethylated triketides, and triketides derived from an acetyl starter unit are not on‐pathway intermediates, but they can be chemically processed by the strPKS KR and DH. Thus, while the presence of a 2‐methyl is not essential for chemical turnover, it is essential for the stereoselectivity at the triketide stage. We previously showed that enoyl reductase (ER) domains of hrPKS show similarly broad substrate selectivity, while retaining high stereoselectivity.^[^ ^37^ ^]^ Such flexibility is likely to be essential for iterative PKS where evolution of programming must involve changes in selectivity of upstream steps, creating new intermediates that must be processed by downstream domains if the newly reprogrammed PKS is to remain functional.

Thus the strPKS illustrates the remarkable ability of an iterative Type I PKS to generate molecular complexity. At the diketide stage all reactions and stereoselectivity are identical to the vertebrate FAS, to which the Type I iPKS show high homology. But, at the triketide stage, methylation and chain‐length, force a reverse in the selectivity of the KR to generate an L‐alcohol. This, in‐turn, changes the DH selectivity at the triketide stage, allowing an L‐alcohol to be processed to the observed *Z*‐product that does not occur at the diketide stage. The structural reasons for these selectivity changes remain mysterious and our future work will focus on the structural biology of the strPKS to probe these questions in detail.

## Supporting Information

Details of all synthetic and analytic procedures, protein production and purification and enzyme assays are included in the online Supporting Information.

## Conflict of Interests

The authors declare no conflict of interest.

## Supporting information



Supporting Information

## Data Availability

The data that support the findings of this study are available from the corresponding author upon reasonable request.
