# Supplementary material for: Stereochemical Dissection of the Strobilurin PKS Reveals the Complex Biosynthetic Logic of Iterative EZE Triene Construction
Source: Angew Chem Int Ed Engl. 2025 Dec 12;65(4):e19062. doi: 10.1002/anie.202519062 (PMC12828468; doi:10.1002/anie.202519062)
Supplement: Supplementary file 1 — Supporting Information [file ANIE-65-e19062-s001.pdf]

Stereochemical Dissection of the Strobilurin PKS Reveals the Complex Biosynthetic Logic of Iterative *EZE* Triene Construction

Maurice Hauser,†[a] Jing Pang,†[a] Daowan Lai,[a,b] Yunlong Sun,[a] Hao Yao[a] and Russell J. Cox*[a]

[a] Mr. M. Hauser M.Sc., Ms J. Pang M.Sc., Prof. D. Lai, Dr Y. Sun, Dr H. Yao & Prof. R. J. Cox
Institute for Organic Chemistry and BMWZ, Leibniz Universität Hannover,
Schneiderberg 38, 30167 Hannover, Germany
E-mail: russell.cox@oci.uni-hannover.de

[b] Prof. D. Lai
Department of Plant Pathology, College of Plant Protection,
China Agricultural University, Beijing 100193,
People's Republic of China. E-mail: dwlai@cau.edu.cn.

† These authors contributed equally

Electronic Supplementary Information

**1.**	**General Methods and Instrumentation**	**2**
**2.**	**Protein Production, Purification, Characterisation, Mutation and Assay**	**4**
**3.**	**Chemical Synthesis and Characterisation**	**21**
**4.**	**Analytical Assays of *in vitro* Enzyme Reactions**	**109**
**5.**	**Catalytic Domain Sequence Analysis**	**151**
**6.**	**References**	**154**

1. General Methods and Instrumentation

1.1 NMR

Bruker Ultrashield 400 equipped with Avance-I console and a DuL ^{1}H, ^{13}C probe at 400 MHz (^{1}H)/101 MHz (^{13}C) Bruker Avance 500 (equipped with a cryo-cooled probe) at 500 MHz (^{1}H)/125 MHz (^{13}C)) and 600 MHz (^{1}H)/150 MHz (^{13}C) spectrometers were used for all NMR analysis. Standard parameters were used for the collection of 2D spectra (1H, 1H-correlation spectroscopy [COSY], heteronuclear single-quantum coherence [HSQC] and Heteronuclear Multiple Bond Correlation (HMBC) spectra in the indicated solvents. ^{1}H and ^{13}C spectra are referenced relative to residual protonated solvents (for $CDCl_3$ ^{1}H: δ = 7.26; ^{13}C: δ = 77.16; for CD_3CN ^{1}H: δ = 1.94 ^{13}C: δ = 118.26; for $(CD_3)_2SO$ ^{1}H: δ = 2.50 ^{13}C δ: = 39.52). All δ values are quoted in ppm and all *J* values in Hz.

1.2 Analytical LCMS and Low Resolution MS

LCMS data were obtained using a Waters LCMS system comprising of a Waters 2767 autosampler, Waters 2545 pump system and a Phenomenex Kinetex column (2.6 μ, C_{18}, 100 Å, 4.6 × 100 mm) equipped with a Phenomenex Security Guard precolumn (Luna C_5 300 Å) eluted at 1 mL/min. Detection was performed by Waters 2998 diode array detector between 200 and 600 nm; Waters 2424 ELSD and Waters SQD-2 mass detector operating simultaneously in ES+ and ES- modes between 100 *m/z* and 650 *m/z*. Solvents were **A**, HPLC-grade H_2O containing 0.05% formic acid; and **B**, HPLC-grade CH_3CN containing 0.045% formic acid. Gradients were as follows: Method 1 (optimised for non-polar compounds): 0 min, 10% **B**; 10 min 90% **B**; 12 min, 90% **B**; 13 min, 10% **B** and 15 min, 10% **B**. Method 2 (optimised for polar compounds): 0 min, 10% **B**; 10 min 40% **B**; 12 min, 90% **B**; 13 min, 10% **B** and 15 min, 10% **B**.

1.3 High Resolution MS

HRMS was obtained using a UPLC system (Waters Acquity Ultraperformance, running the same method and column as above) connected to a Waters Q-TOF Premier mass spectrometer.

1.4 Optical Rotation

Optical rotations were measured on Perkin Elmer 341 polarimeter at the sodium D line (λ_{max} = 589.3 nm) and a cuvette length of d = 1 dm. The concentrations are given in g/100 mL, and the used solvent is mentioned in brackets.

1.5 Preparative LCMS

Compounds were purified using a Waters mass-directed autopurification system consisting of a Waters 2545 pump and Waters 2767 autosampler. The chromatography column was a Phenomenex Kinetex Axia column (5μ, C_{18}, 100 Å, 21.2 × 250 mm) fitted with a Luna C_5 300 Å Phenomenex Security Guard precolumn. The column was eluted at 20 mL/min at 22 °C. Solvents used were **A**, H_2O + 0.05% formic acid; and **B**, CH_3CN + 0.045% formic acid. All solvents were HPLC grade. The column outlet was split (100:1) and the minority flow was supplemented with HPLC-grade MeOH + 0.045% formic acid to 1000 μL/min and diverted for interrogation by diode array (Waters 2998) and evaporative light-scattering (Waters 2424) detectors. The flow was also analysed by mass spectrometry (Waters SQD-2 in ES^+ and ES^- modes). Desired compounds were collected into glass test tubes. Combined fractions were evaporated *in vacuo*, then dissolved directly in HPLC-grade MeOH to make the final concentration 10 mg / mL, 300 μL solution was loaded into the column for purification.

1.6 Isothermal Titration Calorimetry (ITC)

ITC experiments were performed on a Nano ITC machine (TA Instruments) using the indicated buffers and proteins.

1.7 FPLC and MALS

All protein purification was performed using an Åkta Pure system equipped with the described chromatographic column and eluted as described in the text. MALS measurement was performed with an in-line SEC-MALS set-up, comprising a Superose 6 10/300 (GE Healthcare) column on an Åkta Pure system equipped with an Optilab T-rEX detector and mini-DAWN TREOS system (Wyatt). The sample was prepared in 50 mM Tris-HCl, pH 7.5 with 150 mM NaCl. ASTRA 7.0 software package (Wyatt Technologies) was used for data analysis.

2. Protein Production, Purification, Characterisation, Mutation and Assay

The template for cloning the StrDH domain, the DH-ACP multidomain (StrM), the StrACP domain and the StrC-MeT2 domain was gDNA obtained from *A. oryzae* that had previously been transformed with the full strPKS1 CDS and shown to produce the strobilurin tetraketide precursor by LCMS.[1]

2.1 Cloning, Expression and Purification of StrDH

PCR was used to amplify a region corresponding to residues 899-1164 of stpks1 using oligos StrM-F and StrM-R. Linearised (*Nde*I and *Xho*I) pET28a vector (5 µL, 115 ng) was incubated with the purified PCR product (1 µL) that had been digested with *Nde*I and *Xho*I, water (11 µL), T4 ligase 10 × buffer (2 µL), and T4 ligase (1 µL), and incubated at 24 ˚C for 20 min. The enzyme was deactivated at 65 ˚C for 10 min, and the mixture was transformed into *E. coli* Top10. The cells were plated onto LB-agar containing kanamycin (50 µg/ml), and incubated overnight at 37 ˚C. Colonies were screened by colony PCR. One PCR-confirmed colony was picked and grown in 5 mL LB + kanamycin (50 µg/ml) overnight at 37 ˚C. Cells were harvested by centrifugation and plasmids were isolated using the Nucleospin® kit (MACHEREY-NAGEL), and the insert DNA was confirmed by sequencing (Eurofins). One confirmed clone was designated as pET28a-DH.

E. coli BL21(DE3) was transformed with pET28a-DH and selected on agar plates containing kanamycin (50 µg/ml). A single colony was used to inoculate a 5 mL sterile LB starter culture medium containing kanamycin (50 µg/ml). This was grown to stationary phase overnight at 37 °C with shaking. This starter culture was used to inoculate medium supplemented with kanamycin (50 µg/ml) in 500 mL flasks (5 ×) each containing 100 mL of 2TY medium. The flasks were incubated at 37 °C with shaking for 3~4 h until OD_{600} reached ~0.6. The temperature of the culture was then adjusted to 14 °C. The cultures were induced with 1 mM IPTG and incubated with shaking overnight, before harvest by centrifugation. The cell pellets were suspended in 25 mL of binding buffer (50 mM Tris-HCl pH 8.0, 150 mM NaCl, 20 mM imidazole, 10% glycerol). The cell suspension was sonicated in a glass beaker at 0 ˚C at 37% of power for 30 second bursts with 30 seconds rest between bursts for a total of 7 minutes. The lysate was clarified by centrifugation (10,000 g, 45 min, at 4 ˚C).

A 5 mL Protino® Ni-NTA column (MACHEREY-NAGEL) attached to an FPLC instrument, was equilibrated with 15 mL binding buffer. The clarified lysate was then loaded onto the column using an FPLC super loop. The column was then washed with enough binding buffer for the UV trace to settle. The bound protein was eluted over a gradient of 0-100% elution buffer (50 mM Tris-HCl pH 8.0, 150 mM NaCl, 500 mM imidazole, 10% glycerol) in 100 mL. 4 mL fractions were collected. Fractions for which a peak in the UV280 absorbance trace was observed were analysed by SDS-PAGE to determine the purity and size of the eluted protein. Fractions containing the DH protein were combined and loaded onto a HiPrep™ 26/10 desalting column (GE Healthcare) which had previously been equilibrated with 1.5 column volumes of desalting buffer (50 mM Tris-HCl pH 8.0, 150 mM NaCl, 10% glycerol). Up to 15 mL of protein was injected and eluted over 1.5 column volume. Fractions containing DH protein were concentrated using a Amicon® ultra-15 centrifugal concentrator with a 10 kDa cutoff, and used for *in vitro* analysis.

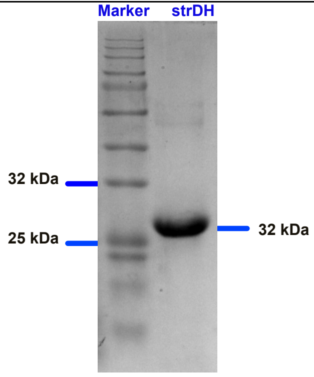

Figure S2.1. Purification of DH protein. SDS-PAGE analysis of concentrated Ni^{2+}-affinity-purified StrDH (lane 2). Lane M, molecular weight markers as indicated.

StrDH Sequence

MGSSHHHHHHSSGLVPRGSHMMKRSRKGPLNYDTLAVNALTHPDLAEHVIKGEPILPATGFFEMIFEEGARTIWDIELRSLLPLLPEKVLNVNVKSDGHAWSIVSS
SGGRNPRLHATGFMTTEVMDKDAGPIDLAAIRARTTPADISNLYAILNNTAAFGPLYRRIEACYEGDHEILYQVRGNAPELTAHYNYVFHPSLLDSCIHGLLHPVF
TGNADKSVFYLPSHIGRVTLYDRAIEEAVPETLYSYVVPHDWTPDSIACDAFIVNERGERLVTLIDCVLSKHWTG

green sequence from vector

2.1.1 Tryptic Digestion and Analysis of StrDH

Proteins were analysed by in-gel digestion. A band of interest was excised from a freshly-run SDS-PAGE gel, digested using trypsin, and analyzed by ESI-MS/MS as described by Klodmann and coworkers.[2]

Accession	Description	Score	Coverage	# Proteins	# Unique Peptides	# Peptides	# PSMs	# AAs	MW [kDa]	calc. pI
DHSTPKS1	DHSTPKS1	4247.31	71.78	1	16	16	90	287	31.9	6.57

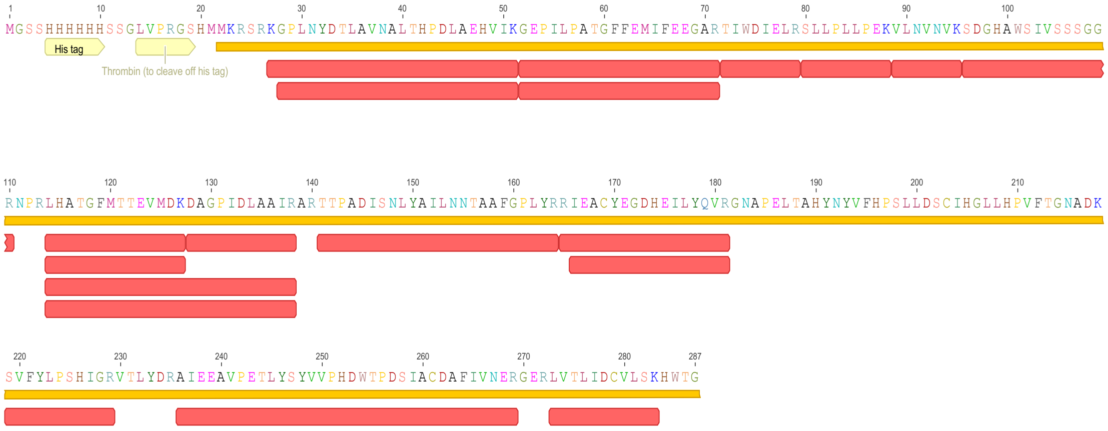

Figure S2.1.1. ESI-MS/MS analysis of His6-StPKS1-DH. Red bars indicate detected peptide fragments.

2.1.2 Biophysical Characterisation of StrDH

An ESI+ spectrum (Waters Quattro Micro) of StrDH was collected between *m/z* 500 - 1500 and deconvoluted using the Max-Ent function of Waters MassLynx Software. The observed mass of the protein (31 808 Da) matched the expected mass of the protein without the N-terminal methionine residue (31 801 Da).

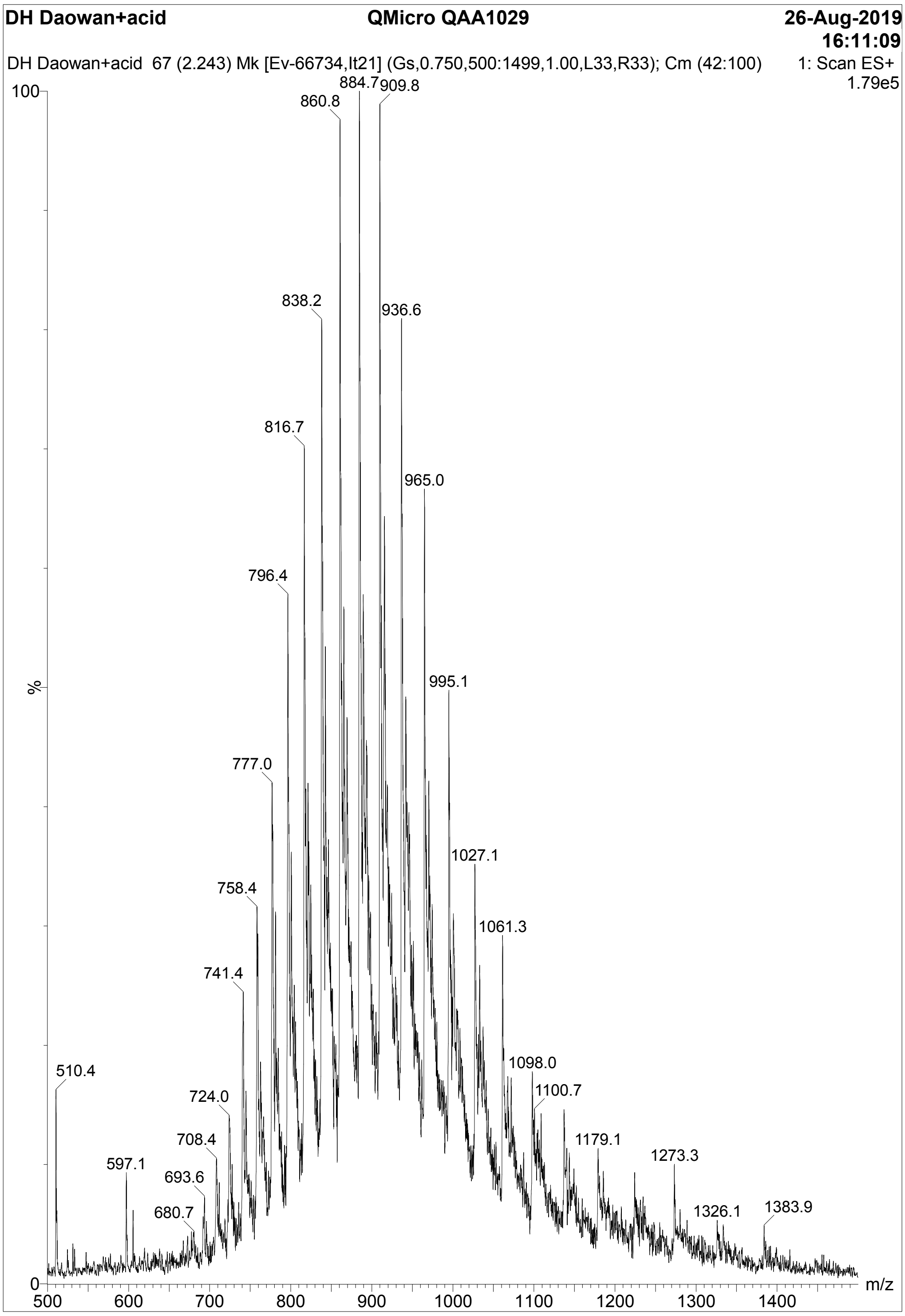

Figure S2.1.2A. ESI+ spectrum of DH protein.

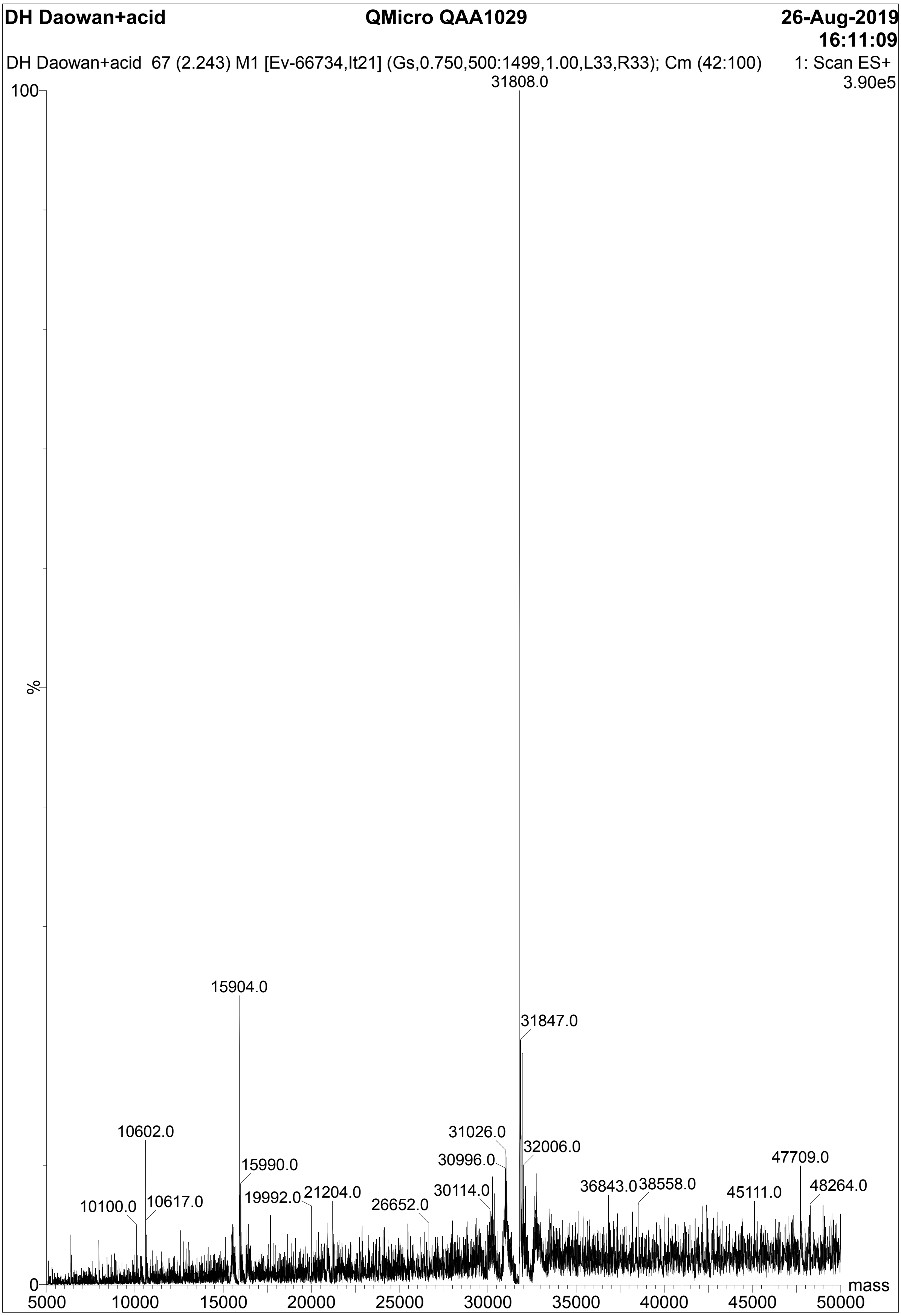

Figure S2.1.2B. Deconvoluted ESI+ spectrum of isolated DH domain. Calc. mass 31 801 Da, obs. mass 31 808 Da.

2.1.3 MALS Analysis/Gel Filtration Analysis of StrDH

Purified DH protein (400 µL) was injected into the S200 column that had been previously calibrated with bovine serum albumin (BSA). The elution buffer was 50 mM Tris, 150 mM NaCl, pH 8.0. The results are consistent with a monomeric DH species (measured, 33 KDa; calculated monomer 32 KDa)

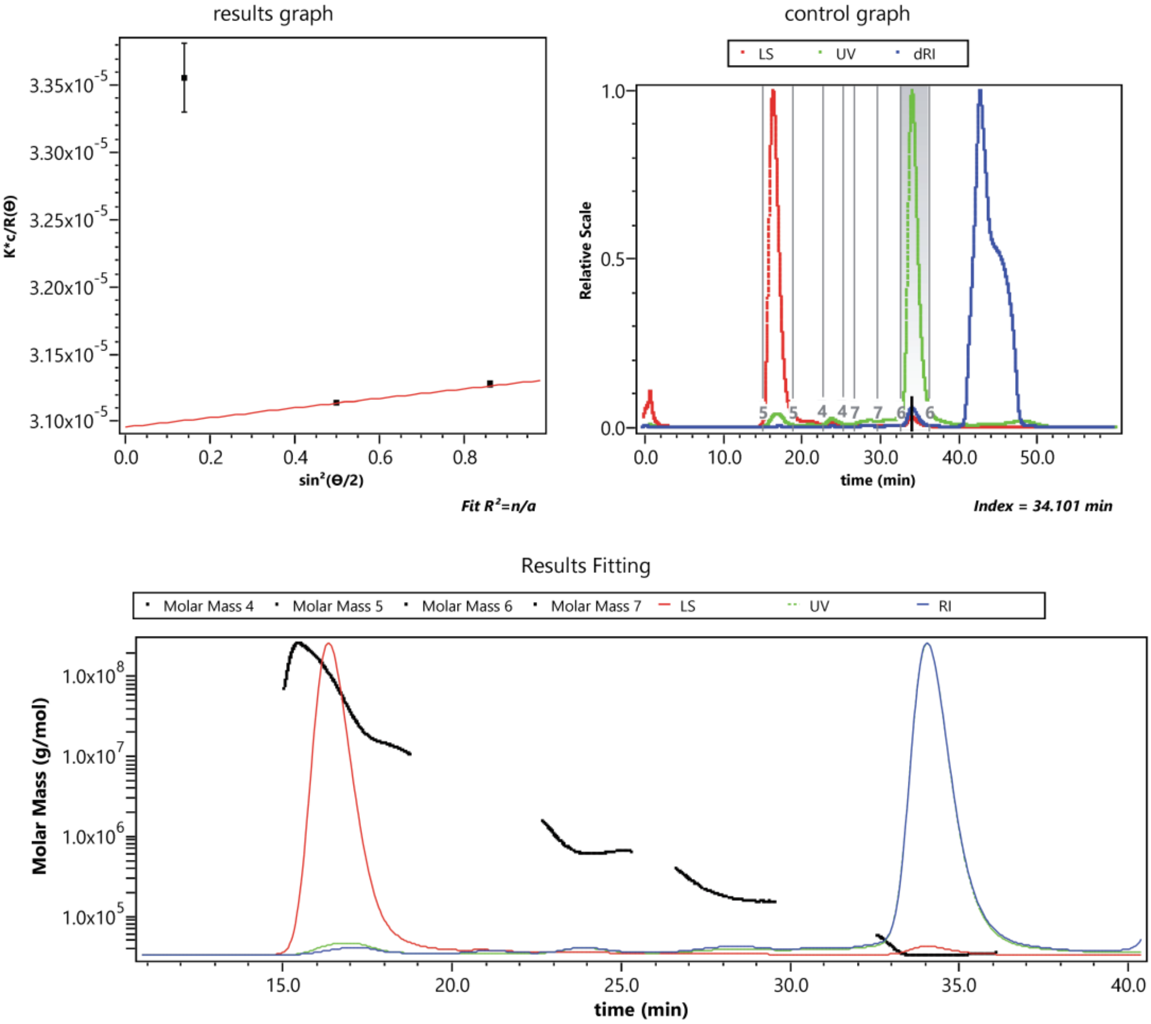

Figure S2.1.3. MALS result of STPKS1-DH

2.2 Cloning, Expression and Purification of strPKS1 DH-ACP multidomain Protein (StrM)

2.2.1 Plasmid Construction

The 3867-base-pair StrM fragment was inserted into the pET28a vector using the Quick-Change site-directed mutagenesis method.[3] This technique involves a supercoiled double-stranded plasmid DNA and a PCR-amplified DNA fragment with flanking sequences complementary to the vector (Fig. S2.2.1). The StrM domain sequence was amplified from StPKS1 using primers (DH-ACP-F/R) containing 30-base-pair sequences overlap to the vector at the 5′ and 3′ ends of the PCR product (Fig. S2.2.1, Table 1, 4). The amplified products were purified *via* agarose gel extraction by using a PCR clean up kit (TAKARA). The purified PCR product and plasmid vector were combined in a thermal cycler (Table 2). Each primer, complementary to opposite strands of the vector, was extended by high-fidelity Q5® DNA polymerase (New England Biolabs) during thermal cycling. This process denatured the plasmid and facilitated annealing of the insert’s 5′ and 3′ flanking regions to the vector, resulting in the formation of nicked circular DNA molecules (Fig. S2.2.1A). After thermal cycling, the reaction mixture was treated with *Dpn*I endonuclease to selectively digest the methylated parental plasmid DNA, leaving only the newly synthesized,

unmethylated plasmid containing the insert (Table 3). The nicked recombinant plasmid was transformed into *E. coli* Top10 competent cells for *in vivo* nick repair. Plasmids were then extracted using a modified mini-prep protocol. The presence and accuracy of the inserted StrM fragment were confirmed by PCR amplification and DNA sequencing (Fig. S2.2.1, Eurofins).

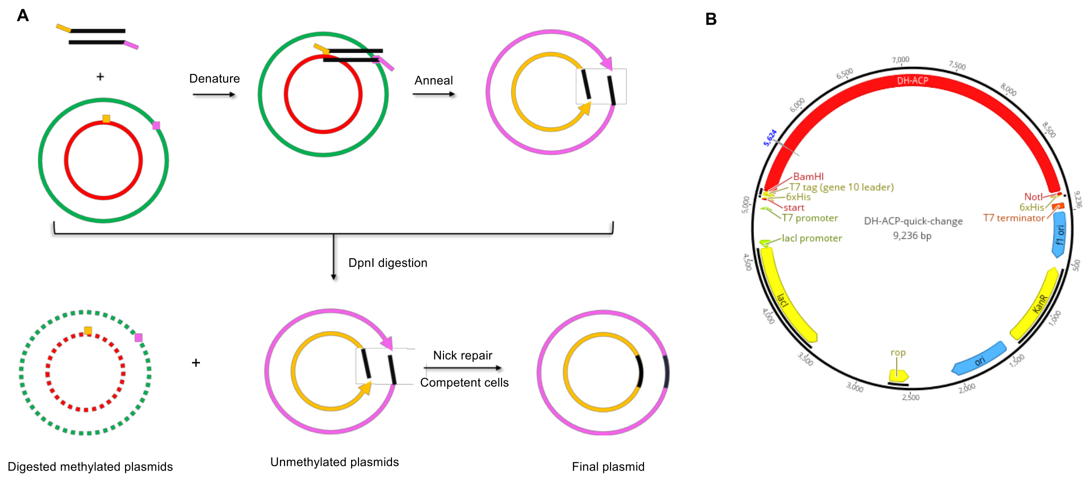

Fig. S2.2.1. **A,** Overview of the steps involved in the Quick-Change method; **B,** Plasmid map of the constructed DH-ACP-pET28a vector.

Table 1. PCR conditions for amplifying the DH-ACP fragment

**Materials**	**Concentration/volume**
Template (stPKS1)	10 ng
Primers (DH-ACP-F/ DH-ACP-R)	10 μM for each
Q5® DNA polymerase	12.5 μL
Sterile distilled water	Up to 25 μL
Begin by heating the reaction mixture to 96 °C for 5 minutes to denature the DNA. Then, perform 30 cycles of PCR consisting of: denaturation at 95 °C for 30 seconds, annealing at 55 °C for 30 seconds, and extension at 72 °C for 30 seconds per kilobase of template DNA. Complete the reaction with a final extension at 72 °C for 10 minutes, followed by a hold at 4 °C.	

Table 2. PCR conditions for Quick-change method

**Materials**	**Concentration/volume**
Template (pET28a plasmid)	25-50 ng
PCR product (DH-ACP with complementary sequences)	250 ng
Q5® DNA polymerase	12.5 μL
Sterile distilled water	Up to 25 μL
Heat the reaction to 96°C for 5 min to denature. Perform 36 cycles of PCR [95° C for 30 sec (denaturation), 55°C for 30 sec (annealing), and 72 °C for 30/kbp template (elongation)]. Followed by final elongation at 72 °C for 10 min hold at 4 °C	

Table 3. *Dpn* I treatment for digestion of the reaction mixture

**Materials**	**Volume**
Reaction mixture	10 μL
10*NEB buffer	2 μL
Dpn I (NEB)	1 μL
Sterile distilled water	7 μL
Digestion for 3 h, use 15 μL result mixture for transformation with chemical competent cells.	

2.2.2 Expression and Purification of StrM

The DH-ACP-pET28a recombinant plasmid was transformed into *E. coli* BL21(DE3) for protein expression. Transformants were selected on LB agar plates containing kanamycin (100 µg/mL). Individual colonies were picked and cultured overnight in 10 mL LB medium at 37 °C. The following day, 1 mL of the overnight culture was used to inoculate 100 mL of fresh LB medium. Cultures were incubated at 37 °C until they reached an optical density at 600 nm (OD_{600}) of 0.6 - 0.8. Protein expression was then induced by adding 0.5 mM isopropyl β-D-1-thiogalactopyranoside (IPTG), followed by incubation at different temperatures (12 °C, 16 °C, 20 °C, and 25 °C) to optimize protein yield and solubility (Fig. S2.2.2).

For protein purification, induction at 16 °C with 0.5 mM IPTG was used. Cells were harvested by centrifugation at 4,000 × g for 10 minutes and resuspended in 10 mL of lysis buffer containing 50 mM Tris-HCl (pH 8.0), 50 mM glycine, 500 mM NaCl, 20 mM imidazole, and 5% (*v/v*) glycerol. The buffer was supplemented with 0.2 mg/mL lysozyme and an EDTA-free protease inhibitor cocktail (Roche). The suspension was incubated on ice for 30 minutes to allow enzymatic digestion of the cell wall. Cell lysis was then carried out using a Sonics Vibra Cell sonicator at 40% amplitude, with pulses of 3 seconds on and 3 seconds off, for a total of 10 minutes.

Following sonication, lysates were clarified by centrifugation at 25,000 × g for 20 minutes. The supernatant was incubated with 250 µL of Ni-NTA agarose beads (Macherey-Nagel) for 1 hour at 4 °C to capture His-tagged proteins. After binding, the resin was collected by centrifugation at 1,000 × g for 2 minutes and washed three times with the lysis buffer. Proteins were then eluted using an elution buffer consisting of 50 mM Tris-HCl (pH 8.0), 50 mM glycine, 500 mM NaCl, 250 mM imidazole, and 5% (*v/v*) glycerol. Dialysis and buffer exchange were performed using centrifugal concentrators with a molecular weight of 30 kDa (StrM protein >30 kDa) into buffer containing 20 mM HEPES (pH 7.5) and 150 mM NaCl. Final protein samples were aliquoted in 30 µL volumes, flash-frozen in liquid nitrogen, and stored at -80 °C for long-term use.

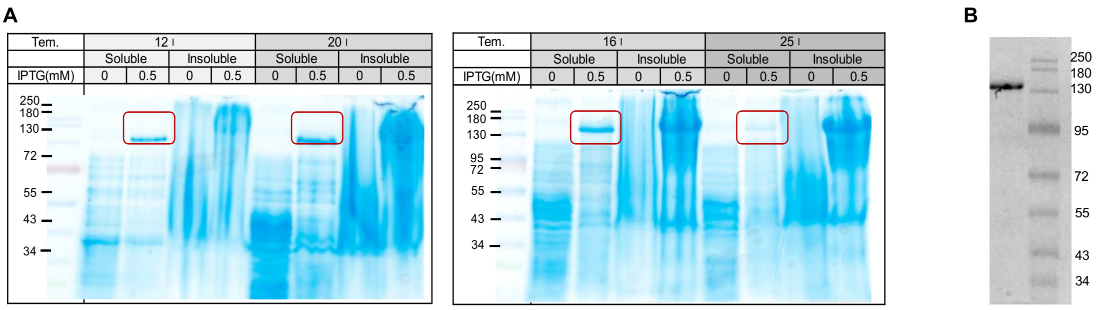

Figure S2.2.2 A, SDS-PAGE analysis of crude protein extracts from BL21 cells expressing 6*HIS-StrM after induction at four different temperatures. The bands include the soluble crude protein from the supernatant, as well as the crude protein from the inclusion bodies. **B,** SDS-PAGE analysis of StrM purified from BL21 cells using Ni-NTA agarose following induction with 0.5 mM IPTG at 16 °C

2.2.3 StrM sequences

ATGAAACGCAGTCGCAAAGGACCTCTGAACTATGATACCTTGGCAGTGAATGCGCTGACGCATCCCGATCTCGCTGAGCACGTCATCAAAGGTGAACCTATCTTAC
CCGCTACTGGTTTCTTCGAAATGATTTTCGAGGAGGGTGCACGCACTATCTGGGACATCGAGCTCCGCAGCTTGCTACCCCTGCTCCCGGAGAAAGTCTTGAATGT
GAATGTCAAATCTGACGGTCACGCCTGGAGCATTGTATCCTCGTCGGGTGGTCGCAACCCGCGTCTACATGCTACTGGTTTCATGACAACGGAGGTCATGGACAAG
GATGCTGGGCCAATCGACCTCGCTGCTATTCGTGCGCGCACCACACCTGCAGACATTTCAAACCTCTATGCTATTCTCAACAACACTGCGGCGTTTGGTCCTCTCT
ATCGTCGTATTGAGGCGTGCTATGAGGGCGATCATGAGATTCTCTACCAAGTTCGGGGAAATGCTCCAGAGCTTACTGCCCACTACAACTACGTCTTCCACCCGTC
GCTCTTGGACTCCTGCATTCACGGCCTCCTGCATCCTGTCTTCACAGGCAATGCCGACAAGAGCGTCTTCTATCTGCCCTCTCACATTGGCCGTGTAACTCTTTAC
GACCGTGCAATTGAGGAGGCAGTTCCTGAGACACTATATTCCTACGTGGTGCCCCATGATTGGACGCCCGACTCTATCGCCTGCGACGCCTTCATTGTCAACGAGC
GAGGAGAACGACTCGTGACCTTGATAGATTGTGTTCTGTCGAAACATTGGACTGGCGCGGTACCCACACGACCAGACACGAGCTACGAGTACATCTATCAACCTCT
TGGCCTGCCCGCTGCGGAGCTCGTCAAATCAGAAGCTCAACAGCAAGACTATGCCTTCCTGGATGCCATAGTTGCTCACGCTGACGAGAAGGTAGCGCCTCCTTCT
GCCAACGGTCATGCTAATGGTCATGCCAACGGTTCTGCTAACGGTTCTGCCGTTGGAACTGTGGGCGAGGATCGTAAAGTCTTCGAGGAGATAGTGCAGTCTATCG
CTTCCGATGAGCTCGAGCTCAAAGCATCTTCGATCCTGGGCCTGTTCTCCGCTTCACTGGACGCCCCTGTGGCGGCCGTTCGCCAGATTTTAGATCATGCTGCGAA
ATCCGGAAAGCAGGTTGTGCGCATCTTGGACATCGGTGATGCCACTGCATCTTTGTACAAGCAGATCAATGCTTTTGCCTCGGAATATCCCTCTCTAAGAGTGGAT
TACACTGCATGTGGCCACGAGCACGCCACGCTTGATCTGCGCCTCGCGTCCTACAACGTTGACAACGTCTCGAAGCAGGCTGGACTTTCGCCTTCCACATACGATG
TCATTATCGAGACACACACTCTTGGCTTTGCGGCCGAATTGGATCGATCGCTCGAGTACTTGCACGGGCTTCTACTTCCTGGTGGTTTCCTCGTCGCCCTTGAGGC
TAATGGGTCTGCACAAGCTTCCGGTGGAAAATGGATAGATCAAGTCTTCTCCCCTCAAGGACGGTGGTCTGGTTTACGCTCCGGGAAACAGCACCATCGGCTGTCA
CAGTCGGAGTGGAGCGGCCAGCTGCAGAAAGCCAAGTTCCAGGTTGTCGATGGGGCACAGGACGCCGAGAACACCTTGTTCTTGACGCTGCTGGCGCAAAAGCACT
CCCTGTCTACCGTCTCCGCGTCCTCCGCAGCATCCTCTGCCAAGGTTGAAGAACCTGCCGTCTTCAGCTTCGACCATTCTCGCGTGTTAGATCTGCAAAAGACCGT
GCTCGCATCCATGTCATCGGGCGCAAGCAACACCAAGCTGTGGATCGAATCTACCACAGGAACCTTCGACGGGGCCGTTGCCACTGGCTTCGCGCGCTCGCTGATG
CGAGAACTTGTGGCCGTTGATGTTCGCTTAGTGCTCTTCGATCCTGCCTGGAAAGCGGAGTCCAGAATTCCTGCCATTCGACAGCTCTCGACCCTTCCCTCCCTCG
AGTCCGAGATCGTGCTCGATGCCTCGGGCGTGGTGATGGTACCCCGTCTGCGCTCCTACGCGCCGCGTGCTCCCGATAGCTTGGATACCACCAAGTATTGGGTGGT
GGATGAGACTAAGACTGTCGTCCAACCAGCACAACCATTGCCCGGCCCTCATCAGGTACTCGTCAAGATCTCGTCTCTGTCTGAGGCGGAGGGCGGACTGCGGGGT
ATTGTTGGGACAGTCGCACGCAGCGGGTCGTCACAATGGCCAGTAGGAGCTCATGTGGTGACCGTTGCGCCGTCTGCACTGTCCAACTTTACCCTAGTCCACGAGG
GTCAGCTCGCTCAAGCCCCCCAGACAGCTGACGAACACAGCACCGCAAAGGTTGCCCTCCTCTTGGTTTTTGCGGCTCTTGGTCTTCGCCTCGACTCCAGGCCACT
GCAGAGCTTGCAACAAATCAAGGTCGTCGTCATTCATACCGGCACGGTTGCTTCCTCTCTCGCGCGACTTCTCGAATACCTGGGTGTGAAACCGGTTTTGGTTGCA
CCCTCATTCCCACTGCTCCTTCCTCGTCTGTCCCCCGGAGACGTTATCATCGGTGGATTATCTGCCGCCTTTGCTCGAACTGTTCCTCGGATCAATGGCGTTTCCG
TCTTCAACTGGGAAGATCCAGAACAAGGTGCCCTCGCCGCCGTCGCTCAAAACCCATGGCTCGTAGGTACCACCGTTGACGCGCACTTGGCGCGTGCTCTCCCACA
GGTCAGCGTTGAGGGCAGCTCGCTCACGCCGGACCAATTGCTTCCATCCGATTTCAGCGTATCCCAGTCTCTGGCCTTGGCGGACGATAAAACCTACCTGGTTCTT
GGTGGAATTGGTTCTTTGGGCCTCCAGATCGCCATCTGGATGTACCAAAAAGGCGCAAGGCATATTGTCCTGACCTCTCGAACTGGTGTTAGTCGACTCGCTGGCA
CGAAGAACAGATCCCTGCGCGGCGCCGTAGAGTATCTCAAGACTCTGCCTGACCTCGAGCTTAGGCTCGAGCCGTGTGACGCTTCATCGGAGGAATCTCTTTCCAA
GCTGATCAGCTCACTGGATCGTCCCCTTGCTGGAGCCATGCTCACAGCCGCCGTCATGGCCGATGGGCTGTTCCTCAAACAGAGTGCCGACACCTACCCGATACCT
TTCAAACCCAAGACTGACGCGTATTTCGCCTTCGAGAAGGTTGTTGATATCAAGAAACTCGATTTCCTGCTTGCTGTCTCCTCCGTGGCTGGTTTCGGAGCCGCTG
GTCAGACTAACTATGCAAGTGCCAACACTGGTATCGAGTATTTGACGGCTCGGTATCCCAACGCTTGGTCCTTCGTGGCACCTGGTATCGCGGACTCCAACGTCGG
CTTCGACCTGTTCACTTCCACCAACTCGCACCTGGAGCAGTGGGAATCCTCTACCATGAATAGTTACGAAATCTGCCTGTGTCTCGAGGACGGCCTGCTACGTATG
GCAAACAATGAGCGTATCTCGATCTATGTGCCTAACTTGAATTGGGATGCCATCTCCCAATCGGTTAGCGAGTCGGTACTCTACAACCATCTGGTGAAGCTGGATG
CGGCGACAGACGAACTCGAGGTTGAGGACCCCTACGAGGTTCTCCAGGAGATTGTGCTCAAGTTTGTTGATGCTTCTGAGGAGGAGTTTGAGAGGAACGTTCCCCT
CACGTCGTATGGACTCGACTCTCTTTCTGCTGCCAGAATGTCCACAGCCTTGAAGCCATACCTCGCGATAACCCAGATCCAGCTGTTGGGTGACCTTAGTCTCGAT
GATCTTGTTGAGAAGATGCAGGCCACCAAGCACGTAGCAGTAGAGGAATAA

StrM

MGSSHHHHHHSSGLVPRGSHMMKRSRKGPLNYDTLAVNALTHPDLAEHVIKGEPILPATGFFEMIFEEGARTIWDIELRSLLPLLPEKVLNVNVKSDGHAWSIVSS
SGGRNPRLHATGFMTTEVMDKDAGPIDLAAIRARTTPADISNLYAILNNTAAFGPLYRRIEACYEGDHEILYQVRGNAPELTAHYNYVFHPSLLDSCIHGLLHPVF
TGNADKSVFYLPSHIGRVTLYDRAIEEAVPETLYSYVVPHDWTPDSIACDAFIVNERGERLVTLIDCVLSKHWTGAVPTRPDTSYEYIYQPLGLPAAELVKSEAQQ
QDYAFLDAIVAHADEKVAPPSANGHANGHANGSANGSAVGTVGEDRKVFEEIVQSIASDELELKASSILGLFSASLDAPVAAVRQILDHAAKSGKQVVRILDIGDA
TASLYKQINAFASEYPSLRVDYTACGHEHATLDLRLASYNVDNVSKQAGLSPSTYDVIIETHTLGFAAELDRSLEYLHGLLLPGGFLVALEANGSAQASGGKWIDQ
VFSPQGRWSGLRSGKQHHRLSQSEWSGQLQKAKFQVVDGAQDAENTLFLTLLAQKHSLSTVSASSAASSAKVEEPAVFSFDHSRVLDLQKTVLASMSSGASNTKLW
IESTTGTFDGAVATGFARSLMRELVAVDVRLVLFDPAWKAESRIPAIRQLSTLPSLESEIVLDASGVVMVPRLRSYAPRAPDSLDTTKYWVVDETKTVVQPAQPLP
GPHQVLVKISSLSEAEGGLRGIVGTVARSGSSQWPVGAHVVTVAPSALSNFTLVHEGQLAQAPQTADEHSTAKVALLLVFAALGLRLDSRPLQSLQQIKVVVIHTG
TVASSLARLLEYLGVKPVLVAPSFPLLLPRLSPGDVIIGGLSAAFARTVPRINGVSVFNWEDPEQGALAAVAQNPWLVGTTVDAHLARALPQVSVEGSSLTPDQLL
PSDFSVSQSLALADDKTYLVLGGIGSLGLQIAIWMYQKGARHIVLTSRTGVSRLAGTKNRSLRGAVEYLKTLPDLELRLEPCDASSEESLSKLISSLDRPLAGAML
TAAVMADGLFLKQSADTYPIPFKPKTDAYFAFEKVVDIKKLDFLLAVSSVAGFGAAGQTNYASANTGIEYLTARYPNAWSFVAPGIADSNVGFDLFTSTNSHLEQW
ESSTMNSYEICLCLEDGLLRMANNERISIYVPNLNWDAISQSVSESVLYNHLVKLDAATDELEVEDPYEVLQEIVLKFVDASEEEFERNVPLTSYGLDSLSAARMS
TALKPYLAITQIQLLGDLSLDDLVEKMQATKHVAVEE

green sequence from vector

2.2.4 Biophysical Characterisation of StrM

2.2.4.1 Gel Filtration Analysis of StrM and StrDH

A freshly prepared, filtered solution of Blue Dextran 2000 (1 mg/mL) was used to determine the Superdex® 200 Increase 10/300 GL column's excluded volume (V_o). The protein standard mix was dissolved in 1 mL of deionized water, and 200 µL of the solution was injected into the column. Separately, 200 µL of myoglobin (1 mg/mL) was also injected into the Superdex® 200 Increase 10/300 GL column pre-equilibrated with two column volumes of elution buffer (50 mM Tris-HCl pH 8.0, 150 mM NaCl). Proteins were eluted with two column volumes of the elution buffer (50 mM Tris-HCl pH 8.0, 150 mM NaCl) at a flow rate of 0.5 mL/min. The elution volumes (V_e) of the protein standard mix and myoglobin were recorded.

K_{av} values for all these samples were calculated according to the equation: $K_{av} = (V_e - V_o)/(V_c - V_o)$.

V_e = elution volume of the sample, V_o = elution volume for Blue Dextran 2000, V_c = total column volume.

A calibration curve was generated by plotting K_{av} values against the logarithm of molecular weight (log MW). The data were fitted with a linear regression to establish the standard curve for molecular weight determination.

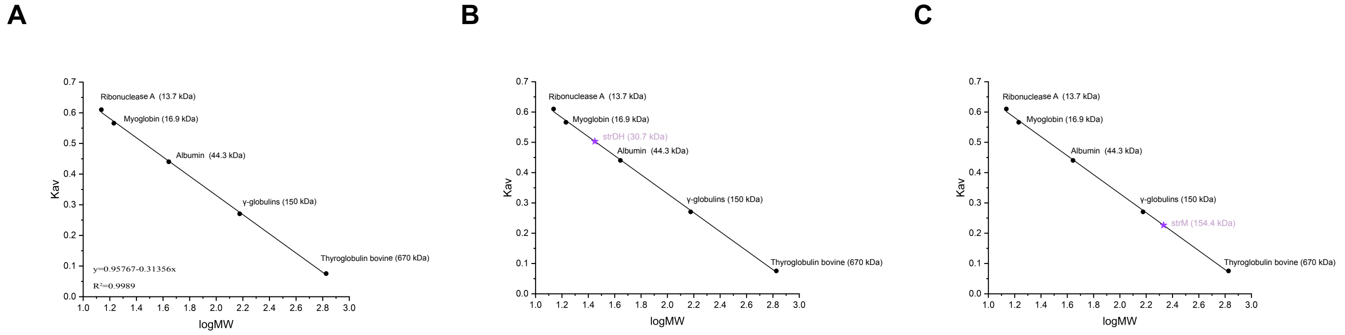

Figure S 2.2.4.1 A, Standard calibration curve for gel filtration chromatography using: Thyroglobulin bovine, γ-globulins, Albumin, Myoglobin, Ribonuclease; **B**, Gel Filtration Analysis of StrDH, Expected molecular weight of StrDH monomer = 31.8 kDa. Estimated molecular weight of StrDH = 30.7 kDa; **C**, Gel Filtration Analysis of StrM, Expected molecular weight of StrM monomer = 142.7 kDa. Estimated molecular weight of StrM = 154.4 kDa.

StrM (400 µL, 1 mg/ml) or StrDH (400 µL, 1.8 mg/ml) were injected into a Superdex® 200 Increase 10/300 GL column pre-equilibrated with two column volumes of elution buffer (50 mM Tris-HCl pH 8.0, 150 mM NaCl). Proteins were eluted with two column volumes of the elution buffer (50 mM Tris-HCl pH 8.0, 150 mM NaCl) at a flow rate of 0.5 mL/min. After obtaining the elution volume (V_e) of StrDH and StrM, the value was converted into a K_{av} and applied to the standard calibration curve to determine the apparent molecular weight of these proteins.

2.3 Cloning, Expression and Purification of StrC-MeT2

The gene fragment StrCMeT2 was amplified from the template vector StPKS1 using PCR (oligos StrCMeT2-F and StrCMeT2-R). The amplified DNA fragment and the pET28a vector were digested separately with *Nde*I and *Xho*I at 37 °C for 1h. The digestion products were purified using the Nucleospin® Gel and PCR Clean-up Kit (MACHEREY-NAGEL) and then ligated into the purified vector at 16 °C overnight using T4 DNA ligase to construct pET28a+StrCMeT2 that was transformed into E.coli TOP10. Plasmids were extracted using the NucleoSpin® Plasmid Kit (MACHEREY-NAGEL). Sequences were confirmed by full plasmid sequencing (Eurofins) before use.

The pET28a protein expression vector was transformed into *E. coli* BL21(DE3). Transformants were selected on LB agar plates containing kanamycin (50 µg/ml). A single colony was inoculated into LB culture contains kanamycin (5 mL, 50 µg/ml) and grown overnight. This seed culture was used to inoculate LB media with kanamycin (700 mL, 50 µg/ml) in a 2 L flask. The culture was incubated at 37 °C, 210rpm until the OD600 reached around 0.8. The culture was then cooled to 17 °C and the protein expression was induced with 0.5 mM IPTG. The cultures continue incubated overnight at 17 °C ,180rpm. The cells were harvested 18 h post-induction by centrifugation and resuspended in lysis buffer (50 mM Tris, 150 mM NaCl, pH 8.0, 10 ml). The suspension was sonicated, and the cell debris was removed by centrifugation at 12,000 × g for 40 min at 4 C. The supernatant was collected and filtered through a 0.45 µm membrane.

A 5 mL HisTrap HP column (Cytiva) was attached to an ÄKTA pure instrument. The column was first equilibrated with 25 mL of elution buffer A (50 mM Tris-HCl pH 8.0, 150 mM NaCl, 20 mM imidazole, 10% glycerol). The filtered supernatant was loaded onto the column using a superloop injection system. The His-tagged protein was eluted using a linear gradient from 0% to 100% of elution buffer B (50 mM Tris-HCl pH 8.0, 150 mM NaCl, 500 mM imidazole, 10% glycerol). Eluted fractions were analyzed by SDS-PAGE to verify the size and purity of the his-tagged protein. Fractions containing the target protein were desalted on a Superdex® 200 10/300 GL column with elution buffer (50 mM Tris-HCl pH 8.0, 150 mM NaCl, 10% glycerol). Fractions containing the desired protein were concentrated using an Amicon® ultra-15 centrifugal concentrator with an appropriate molecular weight cutoff. The concentrated protein (*ca* 10 mg/ml) was used for *in vitro* assays and stored at -20 °C.

2.3.1 Sequences

StrCMeT2

MGSSHHHHHHSSGLVPRGSHMEKPDFSRNLEFDWVDPHPVHQLATMIHNPAMNDLRALFKILDTKALQVMADTISQNPVVGSEISRQRLFEVCKEFVRTQKHSTWT
DEEYEHSKALFPTYFETTERISKVHPSIMESPAAAVGALYSDDMIDGFYRQNKVFTSMNQEAAKTFKALVSSPDFGKQRPIRVLEVGAGVGGLTKFLVEALCDMPN
ADVEYTVTDLSYTLASSLAESFSYKNMVAKMYDLSKKPSEQGLQLGHYDVITGLNVIHAVPDLNATLTDLHSLLAPGGRILIVDTDGTARTSNPPRPGAIWNDFIW
GSFQGWFGYTDDRTHCTIDEDEWRKRLTATGYSNVQVCHEDAGTCILFEAEKV

green sequence from vector

StrCMeT2

GAGAAGCCCGACTTCTCGCGCAACCTCGAATTCGATTGGGTTGACCCGCACCCTGTCCACCAGCTCGCCACGATGATACACAACCCCGCCATGAATGATCTCAGGG
CATTGTTCAAGATCCTGGATACCAAGGCGCTGCAAGTCATGGCCGACACTATCAGCCAGAACCCTGTCGTGGGCTCTGAGATCAGCCGTCAACGCTTGTTCGAGGT
GTGCAAGGAGTTCGTCCGAACTCAGAAACACAGCACTTGGACAGACGAGGAATACGAGCATTCCAAGGCATTGTTCCCGACGTACTTTGAGACCACCGAACGTATC
TCGAAGGTGCACCCGTCCATCATGGAGTCGCCTGCGGCAGCCGTTGGGGCCTTGTACTCGGACGACATGATTGACGGCTTCTATCGCCAGAACAAGGTGTTCACCA
GTATGAACCAGGAGGCTGCGAAGACCTTCAAGGCGCTCGTCTCGTCTCCAGACTTTGGAAAGCAGCGTCCTATCAGGGTCTTGGAAGTTGGTGCAGGTGTCGGTGG
TTTGACGAAGTTCCTCGTCGAGGCTTTGTGCGACATGCCAAACGCCGATGTCGAATACACCGTCACGGATCTCTCCTATACGCTGGCCAGCAGTTTGGCCGAATCG
TTCTCCTACAAGAACATGGTGGCGAAGATGTACGACCTCTCGAAGAAGCCGAGCGAGCAGGGTCTCCAACTGGGCCACTACGACGTGATTACGGGCCTGAATGTTA
TCCACGCCGTCCCCGATCTCAATGCCACCCTCACCGATCTCCACTCACTCCTCGCGCCAGGCGGGCGCATCCTGATTGTCGACACCGACGGCACTGCGCGTACCTC
AAATCCTCCTCGCCCAGGAGCGATTTGGAATGACTTTATCTGGGGTTCCTTCCAGGGATGGTTCGGGTACACCGACGACCGCACGCACTGCACAATCGACGAGGAC
GAATGGAGGAAGCGTCTCACGGCGACAGGGTACTCGAATGTCCAGGTCTGCCACGAAGATGCCGGGACGTGTATCCTGTTCGAAGCAGAGAAGGTC

2.3.2 Biophysical Characterisation of Purified StrC-MeT2

2.3.2.1 Mass Spectrometry of Purified StrC-MeT2

A 0.4 mg/mL protein (StrC-MeT2) solution in ddH$_2$O was directly injected into the mass spectrometer (Waters Quattro Micro) at a flow rate of 0.2 mL/min using a 90:10 (*v/v*) water/acetonitrile (ACN) mixture as the mobile phase. No chromatographic column was installed for separation. An ESI+ spectrum was collected between *m/z* 500 - 1500 and deconvoluted using the Max-Ent function of Waters MassLynx Software. The observed mass of the protein (41 409 Da) matched the expected mass of the protein without the N-terminal methionine residue (41 405 Da).

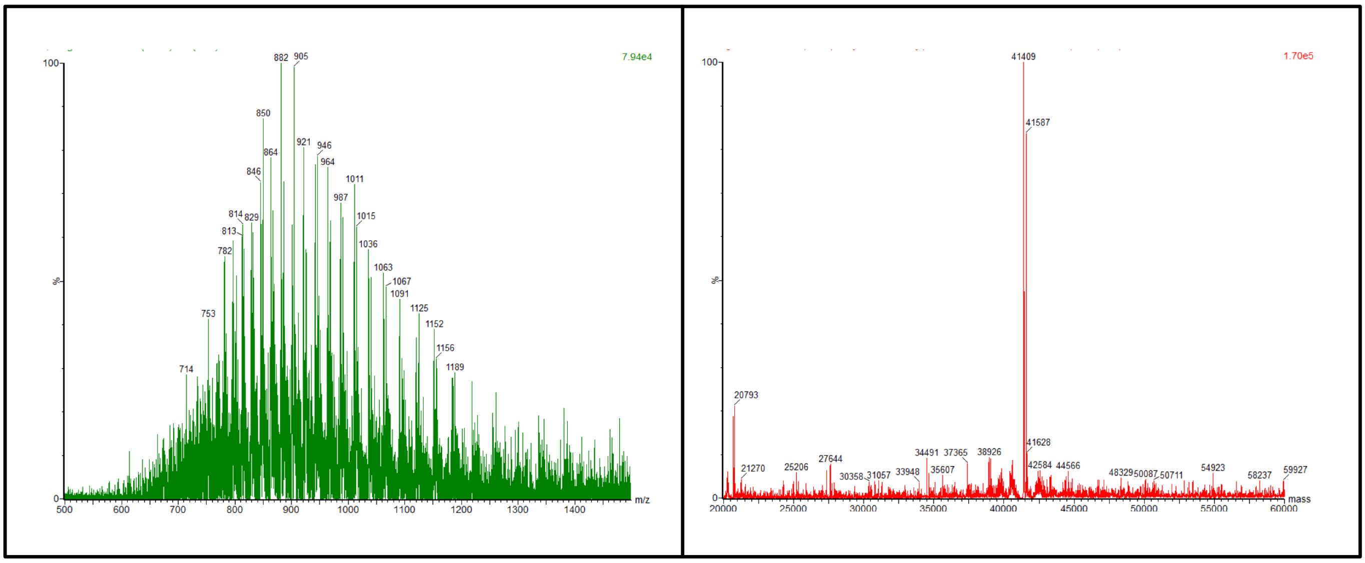

Figure S2.3.2.1 ESI+ spectrum of StrC-MeT2 protein (left) and deconvoluted spectrum (right). *m/z* 41 587 corresponds to N-terminal glucoside.

2.3.2.2 Gel Filtration Analysis of Purified StrC-MeT2

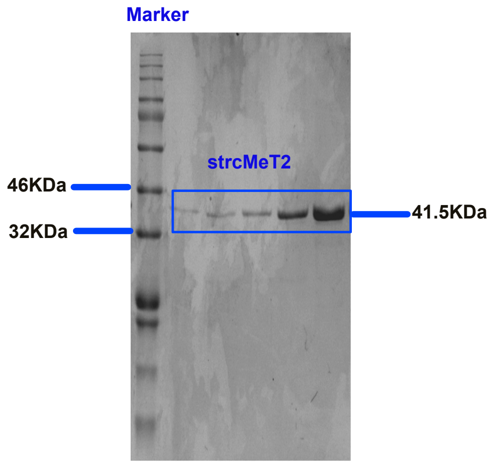

Figure S2.3.2.2A SDS-PAGE analysis of StrCMeT2 Ni-NTA purification fractions

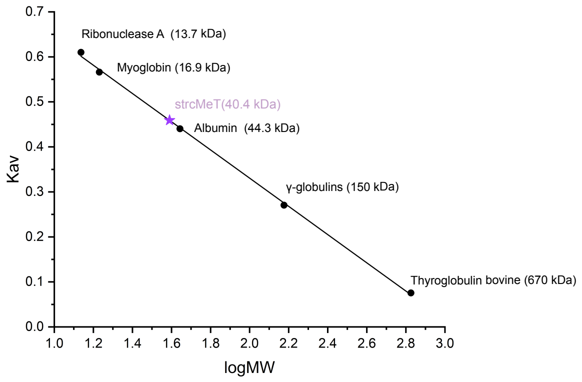

Figure S2.3.2.2B. Size exclusion chromatography of purified StrC-MeT2.

400 μl of the multidomain protein (*i.e.* containing StrC-MeT2, 1 mg/mL) was injected into a Superdex® 200 Increase 10/300 GL column pre-equilibrated with two column volumes of elution buffer (50 mM Tris-HCl pH 8.0, 150 mM NaCl). Proteins were eluted with two column volumes of the elution buffer (50 mM Tris-HCl pH 8.0, 150 mM NaCl) at a flow rate of 0.5 mL/min. After obtaining the elution volume (V_e) of StrC-MeT2, the value was converted into a K_{av} and applied to the standard calibration curve to determine the apparent molecular weight.

2.3.2.3 Isothermal Titration Calorimetry (ITC) of purified StrM and StrC-MeT2 *vs* SAM

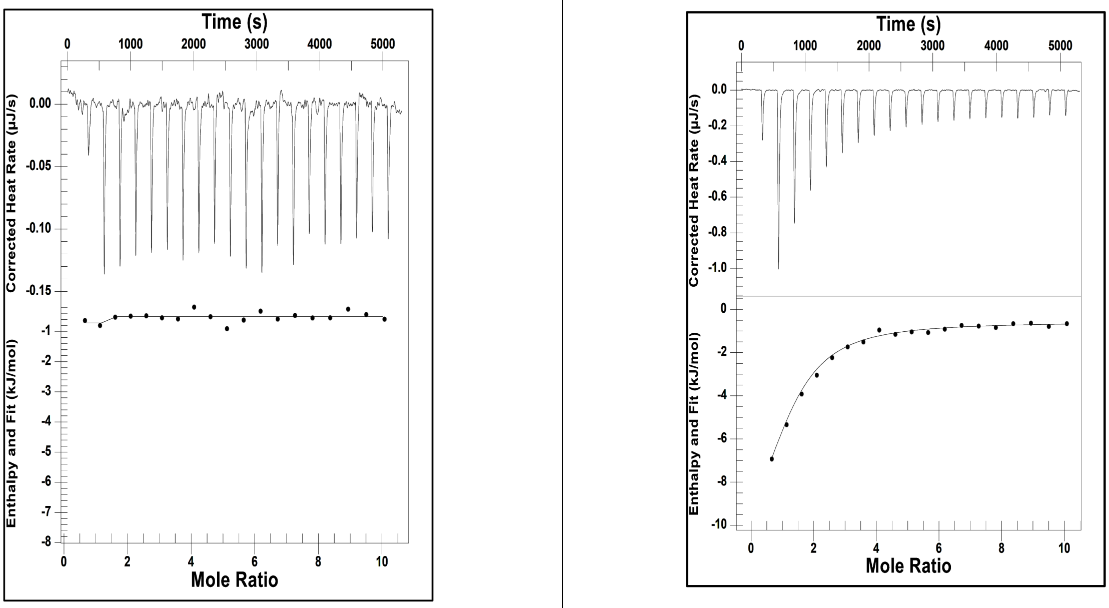

Independent		
	Kd (M)	1.000E-9
	n	1.175
	ΔH (kJ/mol)	-0.212
	ΔS (J/mol·K)	171.6

Independent		
	Kd (M)	3.291E-5
	n	1.071
	ΔH (kJ/mol)	-12.82
	ΔS (J/mol·K)	42.84

Figure S2.3.2.3 ITC investigation of the binding between SAM and StrC-MeT1 (*i.e.* StrM) and StrC-MeT2. Left, SAM to StrM; Right, SAM to StrC-MeT2.

The syringe contained 50 µL of 2 mM *S*-adenosylmethionine (SAM), and 2 µL was injected at each step for a total of 20 injections, with 250 s intervals and a stirring rate of 350 rpm. The cell contained 300 µL of 50 µM protein in buffer (50 mM Tris, 150 mM NaCl, pH 8.0). All titrations were performed at 25 °C under continuous stirring.

2.4 Cloning, Expression and Purification of StrACP

The gene fragment StrACP was amplified from the template vector StPKS1 using PCR. The amplified DNA fragment and the pET28a vector were digested separately with *Nde*I and *Not*I at 37 °C for 1h. The digestion products were purified using the NucleoSpin® Gel and PCR Clean-up Kit (MACHEREY-NAGEL) and then ligated into the purified vector at 16 °C overnight using T4 DNA ligase to construct pET28a+StrCMeT2 that was transformed into *E. coli* TOP10. Plasmids were extracted using the NucleoSpin® Plasmid Kit (MACHEREY-NAGEL). Sequences were confirmed by full plasmid sequencing (Eurofins) before use.

The pET28a protein expression vector was transformed into *E. coli* BL21(DE3). Transformants were selected on LB agar plates containing kanamycin (50 µg/ml). A single colony was inoculated into LB culture contains kanamycin (5 mL, 50 µg/ml) and grown overnight. This seed culture was used to inoculate LB media with kanamycin (700 mL, 50 µg/ml) in a 2 L flask. The culture was incubated at 37 °C, 210 rpm until the OD600 reached around 0.8. The culture was then cooled to 17 °C and the protein expression was induced with 0.5 mM IPTG. The cultures continue incubated overnight at 17 °C ,180 rpm. The cells were harvested 18 h post-induction by centrifugation and resuspended in lysis buffer (50 mM Tris, 150 mM NaCl, pH 8.0, 10 ml). The suspension was sonicated, and the cell debris was removed by centrifugation at 12,000 × g for 40 min at 4 °C. The supernatant was collected and filtered through a 0.45 µm membrane.

A 5 mL HisTrap HP column (Cytiva) was attached to an ÄKTA pure instrument. The column was first equilibrated with 25 mL of elution buffer A (50 mM Tris-HCl pH 8.0, 150 mM NaCl, 20 mM imidazole, 10% glycerol). The filtered supernatant was loaded onto the column using a superloop injection system. The His-tagged protein was eluted using a linear gradient from 0% to 100% of elution buffer B (50 mM Tris-HCl pH 8.0, 150 mM NaCl, 500 mM imidazole, 10% glycerol). Eluted fractions were analyzed by SDS-PAGE to verify the size and purity of the his-tagged protein. Fractions containing the target protein were desalted on a Superdex® 200 10/300 GL column with elution buffer (50 mM Tris-HCl pH 8.0, 150 mM NaCl, 10% glycerol). Fractions containing the desired protein were desalted and concentrated using an Amicon® ultra-15 centrifugal concentrator with 3 KDa molecular weight cutoff. The concentrated protein (*ca* 30 mg/ml) was used for *in vitro* assays and stored at -20 °C. The concentrated protein was used for *in vitro* assays and stored at -20 °C.

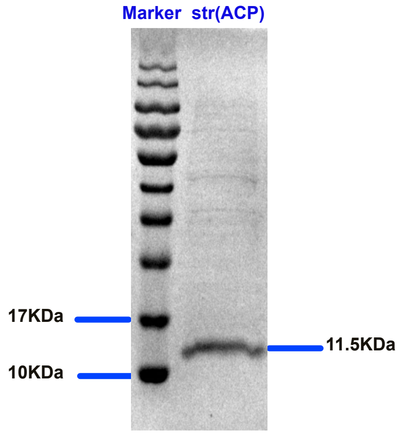

Figure S2.4 SDS-PAGE analysis of purified *apo*-StrACP.

2.4.1 Biophysical Characterisation of Purified StrACP

2.4.1.1 Gel Filtration Analysis of Purified *apo*-StrACP

400 µl of StrACP (1mg/mL) was injected into a Superdex® 200 Increase 10/300 GL column pre-equilibrated with two column volumes of elution buffer (50 mM Tris-HCl pH 8.0, 150 mM NaCl). Proteins were eluted with two column volumes of the elution buffer (50 mM Tris-HCl pH 8.0, 150 mM NaCl) at a flow rate of 0.5 mL/min.

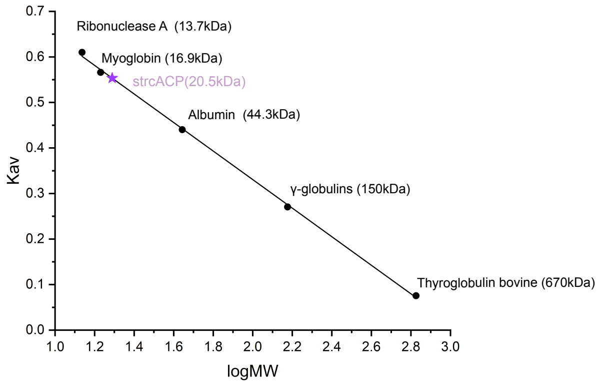

Figure S2.4.1.1 Gel-filtration analysis of StrACP. Expected molecular weight of StrACP monomer = 11.5 kDa. Estimated molecular weight of *apo*-StrACP = 20.5 kDa.

2.4.1.2 MS of *apo*-StrACP

A 0.5 mg/mL protein (StrACP) solution in ddH$_2$O was directly injected into the mass spectrometer (waters Quattro Micro) at a flow rate of 0.2 mL/min using a 90:10 (*v/v*) water/acetonitrile mixture as the mobile phase. No chromatographic column was installed for separation. The protein's identity was confirmed by mass spectrometry. An ESI+ spectrum was collected between *m/z* 500 - 1500 and deconvoluted using the Max-Ent function of Waters MassLynx Software. The observed mass of the protein (11 391 Da) matched the expected mass of the protein without the N-terminal methionine residue (11 389.7 Da).

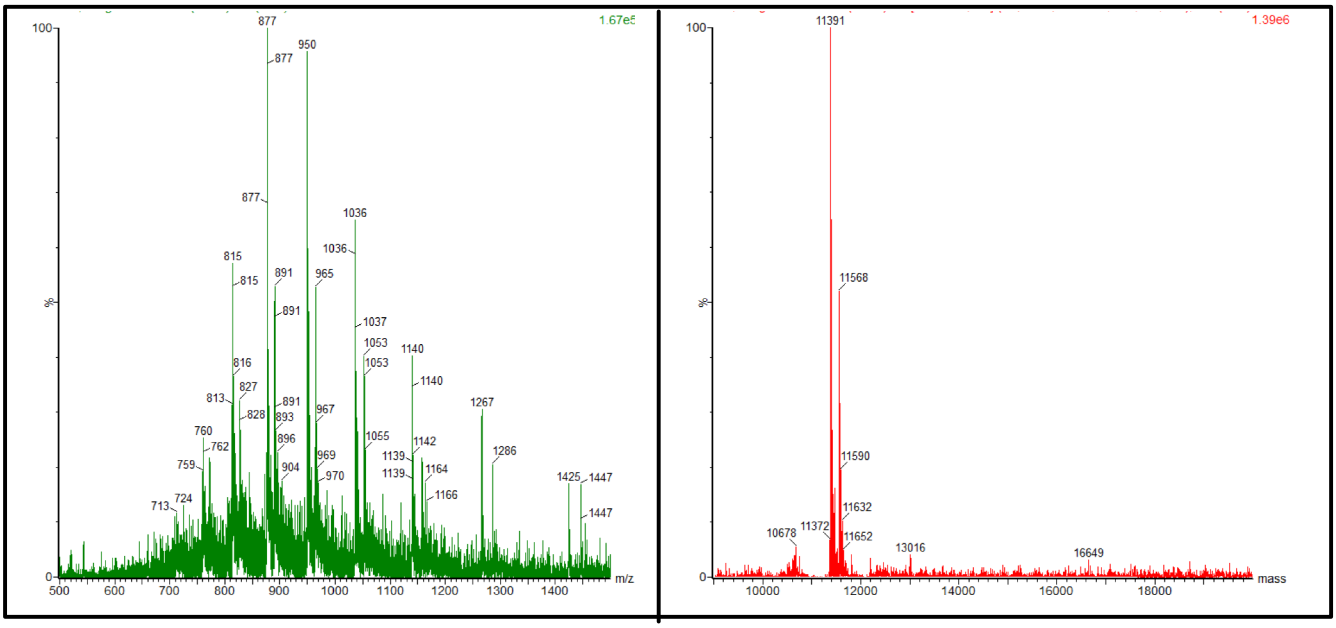

Figure S2.4.1.2 ESI+ spectrum of StrACP protein (left) and deconvoluted spectrum (right). Calc 11 389.7 for pET28a-expressed protein minus methionine. *m/z* 11 568 corresponds to N-terminal glucoside.

2.4.1.3 Conversion of Purified *apo*-StrACP to *holo*-StrACP

In a final reaction volume of 400 µL, the solution contained 10 mM $MgCl_2$, 5 mg/mL *apo*-ACP, 0.5 mg/mL Sfp, and 1 mM CoA. After incubation at 30 °C for 2 h, the sample was diluted ten-fold, and 20 µL was injected for MS analysis.

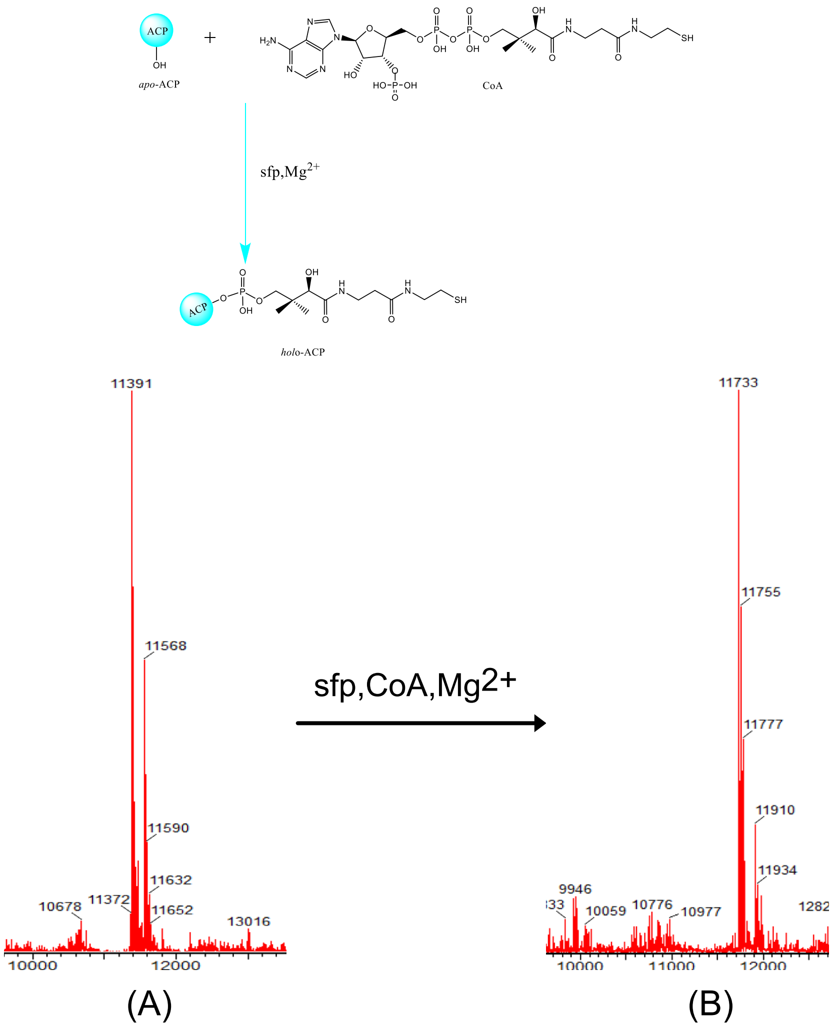

Figure S2.4.1.3 (A) deconvoluted spectrum of *apo*-StrACP; **(B)** deconvoluted spectrum of *holo*-StrACP. *holo*-ACP calc 11 731.3; *m/z* 1755 and 11 777 correspond to Na adducts; *m/z* 11 910 corresponds to N-terminal glucoside.

2.4.2 Sequences

StrACP

MGSSHHHHHHSSGLVPRGSHMVEDPYEVLQEIVLKFVDASEEEFERNVPLTSYGLDSLSAARMSTALKPYLAITQIQLLGDLSLDDLVEKMQATKHVAVEETAV

green residues from pET28a vector.

StrACP

GTTGAGGACCCCTACGAGGTTCTCCAGGAGATTGTGCTCAAGTTTGTTGATGCTTCTGAGGAGGAGTTTGAGAGGAACGTTCCCCTCACGTCGTATGGACTCGACT
CTCTTTCTGCTGCCAGAATGTCCACAGCCTTGAAGCCATACCTCGCGATAACCCAGATCCAGCTGTTGGGTGACCTTAGTCTCGATGATCTTGTTGAGAAGATGCA
GGCCACCAAGCACGTAGCAGTAGAGGAAACCGCGGTC

2.5 Site Directed Mutagenesis

The Quick-Change PCR method was used to perform the site-directed mutagenesis. The protein expression plasmid was used as the template. A pair of primers was designed containing the target mutation(s), but otherwise exactly matching the plasmid template. The mutation site was positioned near the centre of the primers. The primers (0.8 µM)and template (6 ng) were used in two separate PCR reactions (25 µL) each containing either the forward or reverse primer. NEB Q5 master mix was used over 30 PCR cycles. The two single-primer PCR products were combined into one tube. 10 × CutSmart buffer (6 µl), dd H_2O (2 µL) and 1 µL *Dpn*I was added, to a final volume of 60 µL. The tube was heated to 37 °C, 3 h to activate the *Dpn*I, then 80 °C for 20 min to inactivate *Dpn*I, then 90 °C for 15 min for denaturation, then cooled to 25 °C for re-annealing of the complementary strands. The re-annealed plasmid was transformed into competent *E. coli* OneShot Top10 cells, then plated on LB agar containing kanamycin (50 µg/mL). Single colonies (2-3) were picked and inoculated into 15 mL LB media plus kanamycin and incubated overnight at 37 °C with shaking at 200 rpm. Plasmids were extracted and fully sequenced (Eurofins) before use.

2.6 Enzyme Assay Procedures

DH enzyme assays were performed in a final volume of 50 µL, containing the substrate (1 mM), StrDH or StrM (10 mg/ml) in Tris-HCl buffer (50 mM Tris, 150 mM NaCl, pH 8.0). A negative control was prepared using boiled enzyme. The reaction sample was incubated at 25 °C for 12 h. Acetonitrile (50 µL) was added to precipitate the enzyme and quench the reaction. The suspension was then centrifuged at 12,000 × g for 15 minutes to remove the precipitated protein. The resulting supernatant was analyzed directly by LCMS.

KR enzyme assays were performed in a final volume of 50 µL, containing the substrate (1 mM), StrM, (10mg/ml), NADPH (2 mM) in Tris-HCl buffer (50 mM Tris, 150 mM NaCl, pH 8.0). A negative control was prepared using boiled StrM enzyme. The reaction sample was incubated at 25 °C for 12 h. Acetonitrile (50 µL) was added to precipitate the enzyme and quench the reaction. The suspension was then centrifuged at 12,000 × g for 15 minutes to remove the precipitated protein. The resulting supernatant was analyzed directly by LCMS.

C-MeT2 enzyme assays were performed in a final volume of 50 µL, containing the substrate (1 mM), StrC-MeT2 (10 mg/ml) , SAM (2 mM) in Tris-HCl buffer (50 mM Tris, 150 mM NaCl, pH 8.0). A negative control was prepared using boiled StrC-MeT2 enzyme. The reaction sample was incubated at 25 °C for 12 h. Acetonitrile (50 µL) was added to precipitate the enzyme and quench the reaction. The suspension was then centrifuged at 12,000 × g for 15 minutes to remove the precipitated protein. The resulting supernatant was analyzed directly by LCMS. The same procedure was used for *C*-MeT1 assays, but using StrM as the enzyme.

2.7 Oligo Sequences

Table. 4 Oligonucleotide sequences

Primer	Sequence (5'- 3')	Information
StrDH-F	gggaattccatatgATGAAACGCAGTCGCAAAGGA	restriction site red
StrDH-R	ccgctcgagCTAGCCAGTCCAATGTTTCGACAG	restriction site red; stop codon green
StrM-F	actggtggacagcaaatgggtcgcggatccATGAAACGCAGTCGCAAAGG	The part where the primer sequence overlaps with the plasmid is in lower case; stop codon green
StrM-R	gccgcaagcttgtcgacggagctcgaattcTTATTCCTCTACTGCTACGT	
StrCMeT2-F	gggaattccatatgGAGAAGCCCGACTTCTCG	restriction site green-red
StrCMeT2-R	ccgctcgagCTAGACCTTCTCTGCTTCGAACAGG	restriction site red; stop codon green
StrACP-F	CGCGGATCCGTTGAGGACCCCTACGAGGTTC	restriction site red
StrACP-R	ATAGTTTAGCGGCCGCCTAGACCGCGGTTTCCTC	restriction site red; stop codon green
H925A-F	CGATCTCGCTGAGGCCGTCATCAAAGG	DH Mutation Primer, mutation site red
H925A-R	CCTTTGATGACGGCCTCAGCGAGATCG	DH Mutation Primer, mutation site red
H925Q-F	CCGATCTCGCTGAGCAAGTCATCAAAGG	DH Mutation Primer, mutation site red
H925Q-R	CCTTTGATGACTTGCTCAGCGAGATCGG	DH Mutation Primer, mutation site red
H1082A-F	GGACTCCTGCATTGCCGGCCTCCTGCATCC	DH Mutation Primer, mutation site red
H1082A-R	GGATGCAGGAGGCCGGCAATGCAGGAGTCC	DH Mutation Primer, mutation site red
H1082Q-F	GACTCCTGCATTCAAGGCCTCCTGCATCC	DH Mutation Primer, mutation site red
H1082Q-R	GGATGCAGGAGGCCTTGAATGCAGGAGTC	DH Mutation Primer, mutation site red
F1036A-F	CACTGCGGCGGCTGGTCCTCTCTATCG	DH Mutation Primer, mutation site red
F1036A-R	CGATAGAGAGGACCAGCCGCCGCAGTG	DH Mutation Primer, mutation site red
F1036Y-F	CAACACTGCGGCGTATGGTCCTCTCTATCG	DH Mutation Primer, mutation site red
F1036Y-R	CGATAGAGAGGACCATACGCCGCAGTGTTG	DH Mutation Primer, mutation site red
F1036H-F	CACTGCGGCGCATGGTCCTCTCTATC	DH Mutation Primer, mutation site red
F1036H-R	GATAGAGAGGACCATGCGCCGCAGTG	DH Mutation Primer, mutation site red
H1082F-F	GACTCCTGCATTTTCGGCCTCCTGCATC	DH Mutation Primer, mutation site red
H1082F-R	GATGCAGGAGGCCGAAAATGCAGGAGTC	DH Mutation Primer, mutation site red
Add 1034G-F	CTATTCTCAACAACACTGGAGCGGCGTTTGGTCCTC	DH Mutation Primer, mutation site red
Add 1034G-R	GAGGACCAAACGCCGCTCCAGTGTTGTTGAGAATAG	DH Mutation Primer, mutation site red
A1943R-F	GCCGCCGTCATGAGGGATGGGCTGTTCC	KR Mutation Primer, mutation site red
A1943R -R	GGAACAGCCCATCCCTCATGACGGCGGC	KR Mutation Primer, mutation site red
F1990W-F	CCGTGGCTGGTTGGGGAGCCGCTGG	KR Mutation Primer, mutation site red
F1990W-R	CCAGCGGCTCCCCAACCAGCCACGG	KR Mutation Primer, mutation site red
M1942L-F	CAGCCGCCGTCCTGGCCGATGGGCTG	KR Mutation Primer, mutation site red
M1942L-R	CAGCCCATCGGCCAGGACGGCGGCTG	KR Mutation Primer, mutation site red

3. Chemical Synthesis and Characterisation

3.1 Overview

Diketide substrates were prepared for the DH, KR and *C*-MeT domains. In initial work ethyl 3-oxo-3-phenylpropanoate **27a** was *C*-methylated and reduced to give a racemic mixture of 2-methyl-3-hydroxy diastereomers **28b** (Scheme S3.1A-A). This was converted to the corresponding mixture of SNACs **9b** by *tert*-butyldimethylsilyl (TBDMS) protection of the secondary alcohol, base hydrolysis of the ester and then EDCI-coupling with HSNAC **29**. The racemic 2,3-*syn* and α,β-*anti* diastereomers were then separated by HPLC (Scheme S3.1.1-A).

Enantiomerically pure isomers L-*anti*-**9b** and D-*anti*-**9b** were prepared using Evans aldol chemistry from benzaldehyde **31**, and a similar sequence of reactions after peroxide-removal of the chiral auxiliary (Scheme S3.1.1-B). The β-oxo diketides **15a** and **15b** were most conveniently prepared by addition of lithium enolates of either ethyl acetate **34a** (non-methylated series) or ethyl propionate **34b** (methylated series) to **31**, followed by Dess-Martin oxidation to **27a** and **27b**. The ethyl ester products were first protected as cyclic ketals **35a** and **35b** with ethylene glycol, before basic ester hydrolysis and EDCI-mediated coupling to **29**. Final ketal deprotection was achieved in a mixture of TFA, CH_2Cl_2 and H_2O (Scheme S3.1.1-C).

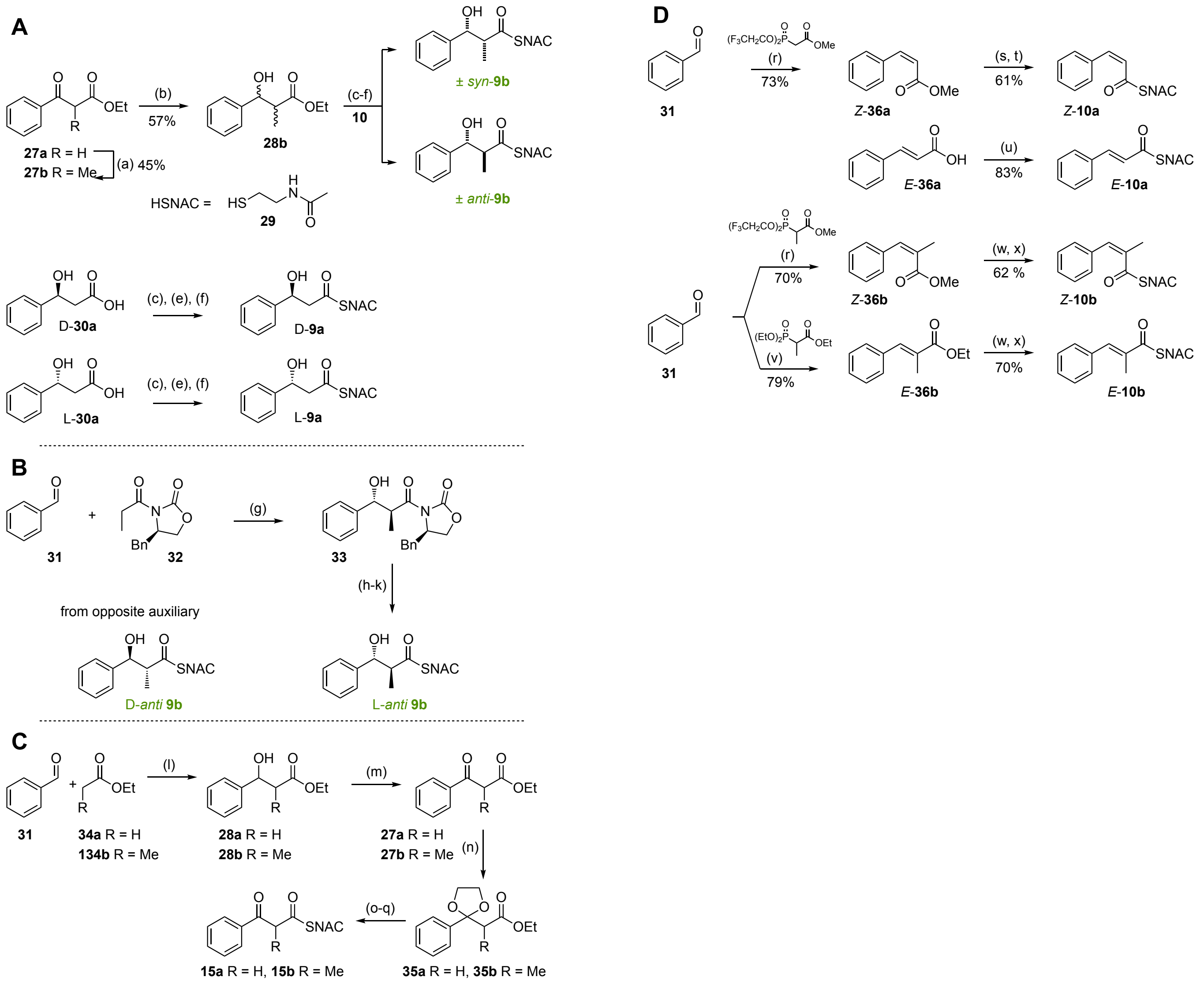

Scheme S3.1.1 Synthesis of diketide substrates and products. Reagents and conditions: (a) MeI, NaH, THF; (b) $NaBH_4$, EtOH; (c) TBDMSCl, CH_2Cl_2, DMAP, imidazole, rt; (d) aq. LiOH, THF, MeOH, 60 °C; (e) **29**, DMAP, EDCI, CH_2Cl_2, 0 °C to rt; (f) TBAF, AcOH, THF, rt; (g) $MgCl_2$, Et_3N, TMSCl, EtOAc, rt, then TFA, MeOH, rt; (h) TBDMSCl, CH_2Cl_2, DMAP, imidazole, rt; (i) LiOOH, H_2O, 0 °C to rt; (j) **29**, EDCI, DMAP, 0 °C to rt; (k) TBAF, AcOH, THF, rt, 5d; (l) LDA, EtOAc, THF, -78 °C to -40 °C; (m) DMP, CH_2Cl_2, rt; (n) $(CH_2OH)_2$, pTsOH, $CH(CH_3O)_3$, CH_2Cl_2, rt; (o) aq. NaOH, 60 °C (R = H) or 90 °C (R = Me); (p) **29**, DMAP, EDCI, CH_2Cl_2, 0 °C to RT (q) TFA, H_2O, CH_2Cl_2, rt; (r) NaHMDS, THF, -78 °C; (s) aq. LiOH, 60 °C; (t) **29**, EDCI, DMAP, 0 °C; (u) **29**, EDCI, DMAP, 0 °C; (v) NaH, THF, rt; (w) aq. LiOH, 60 °C; (x) **29**, EDCI, DMAP, 0 °C to RT.

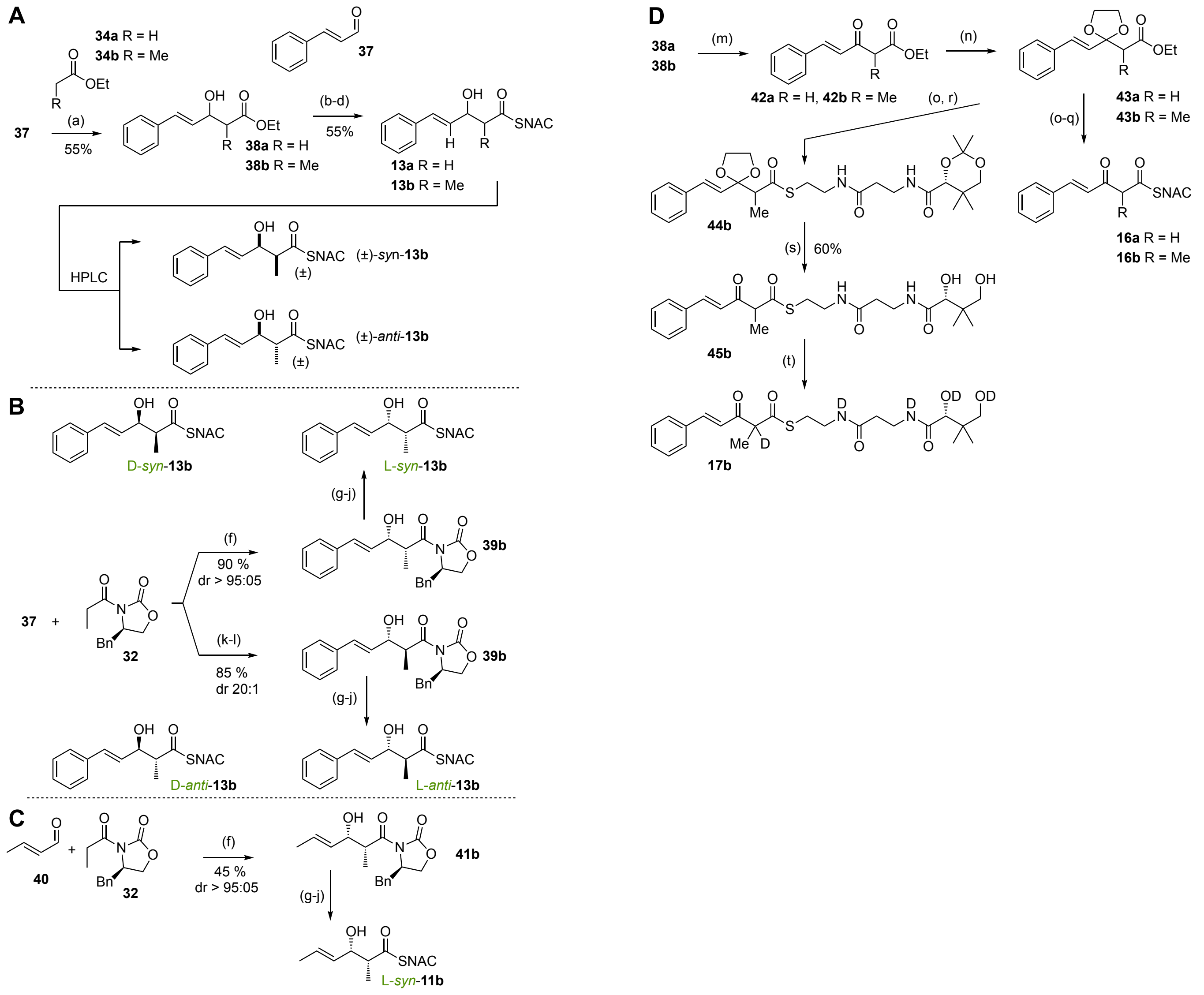

Scheme S3.1.2. Synthesis of triketides. Reagents and conditions: (a) LDA, THF, -78 °C to -40 °C; (b) aq. LiOH, 60 °C; (c) TBDMS-Cl, Imidazole, DMAP, CH_2Cl_2, 0 °C to rt; (d) **29**, EDCI, DMAP, 0 °C - rt; (e) TBAF, AcOH, THF, rt, 4d; (f) nBu_2BOTf, CH_2Cl_2, Et_3N, -78 °C; (g) TBDMSOTf, Pyridine, CH_2Cl_2, 0 °C; (h) LiOOH, H_2O, 0 °C to rt; (i) **29**, EDCI, DMAP, 0 °C to rt; (j) TBAF, AcOH, THF, rt, 5-7d; (k) $MgCl_2$, Et_3N, TMSCl, EtOAc, rt; (l) TFA, MeOH, rt; (m) $KMnO_4$, CH_2Cl_2, 30 °C; (n) $(CH_2OH)_2$, pTsOH, $CH(CH_3O)_3$, CH_2Cl_2, rt; (o) aq. NaOH, 60 °C;(p) **29**, DMAP, EDCI, CH_2Cl_2, 0 °C to rt; (q) TFA, H_2O, CH_2Cl_2, rt; (r) protected pantetheine, DMAP, EDCI, CH_2Cl_2, 0 °C to rt; (s) HCl, H_2O, THF, 40 °C, 0.5 to 1h; (t) D_2O, d_6-DMSO, rt, 16h.

Diketide olefins *E*- and *Z*-**10a** (unmethylated) and *E*- and *Z*-**10b** (methylated) were prepared using Still-Gennari chemistry (*Z* series) or the analogous Horner-Wadsworth-Emmons (HWE) olefination (*E* series, Scheme S3.1.1-D). The ester intermediates were hydrolysed under basic conditions before final EDCI-mediated coupling with **29** to form the required SNACs.

Similar chemistry was used to create the triketide series of substrates and products. Racemic unmethylated triketide alcohol **13a** was prepared by aldol addition to β-phenylpropenal **37** (Scheme S3.1.2-A). Individual enantiomers of the α-methyl α,β-*syn* alcohols **11** and **13** were made *via* Evans boron enolate chemistry, followed by secondary alcohol protection, auxiliary cleavage, SNAC formation and final deprotection. Both enantiomers were formed from the corresponding enantiomeric *N*-propionyl auxiliaries, and the 4-alkene and 4-alkane series were created by starting with **37** or **40** respectively). The enantiomeric α,β-*anti* alcohols **13b** were made similarly, but using silyl enol ethers starting from the same *N*-propionyl auxiliaries (Scheme S3.1.2-B). Using the same chemistry, but starting from prop-2-enal **40** afforded the *syn* triketide **11b** (Scheme S3.1.2-C).

The β-oxo triketides **16a** and **16b** were prepared by permanganate oxidation of the corresponding β-alcohols, followed by ketal protection, basic ester hydrolysis, EDCI-mediated SNAC formation and final ketal deprotection (Scheme S3.1.2-D). We also synthesised a 2-^{2}H 2-methyl-3-oxo triketide. Here we utilised pantetheine instead of SNAC because its increased

solubility in aqueous buffer allows a higher substrate concentration to be used *in vitro*, in-turn allowing reactions to be followed by NMR. In this case, ester protected triketide **43b** was hydrolysed, coupled to protected pantetheine (EDCI) before global deprotection using HCl in H_2O/THF. The product was dissolved in D_2O/d_6-DMSO and after 16 h ^{1}H-NMR analysis showed > 98% incorporation of ^{2}H at the 2-position (Scheme S3.1.2-D). Individual triketide dienes diastereomers *E*- and *Z*-**14a** and *E*- and *Z*-**14b** were synthesised using either Still-Gennari chemistry (2*Z*-series) or HWE chemistry (2*E*-series) starting from *E*-3-phenylpropenal **37** (Scheme S3.1.3). As previously, basic ester hydrolysis was followed by EDCI-mediated SNAC formation.

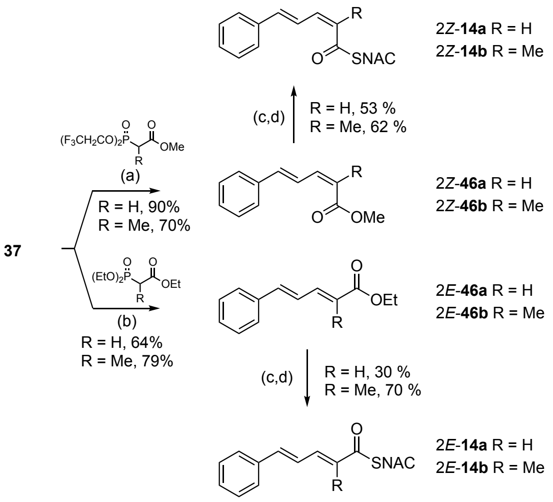

Scheme S3.1.3 Synthesis of dienes. Reagents and conditions: (a) NaHMDS, THF, -78 °C; (b) NaH, THF, rt; (c) aq. LiOH, 60 °C; (d) **29**, EDCI, DMAP, 0 °C to RT.

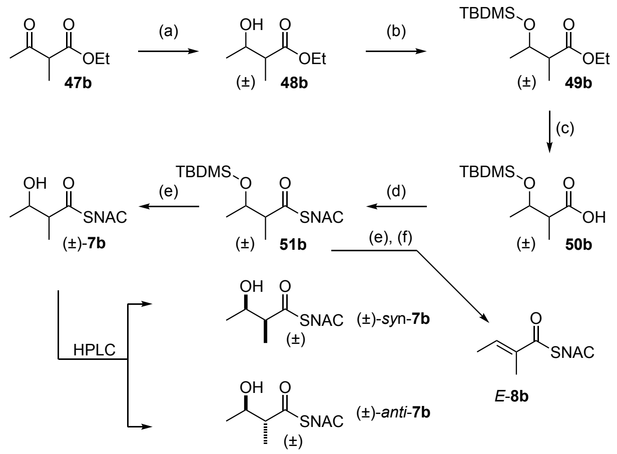

Scheme S3.1.4 Synthesis of racemic acetyl-derived diketide SNACs. Reagents and conditions: (a) $NaBH_4$, EtOH; (b) TBDMS-Cl, Imidazole, DMAP, CH_2Cl_2, 0 °C to rt; (c) aq. LiOH, 60 °C; (d) **29**, EDCI, DMAP, 0 °C to rt; (e) TBAF, AcOH, THF, rt; (f) $MeSO_2Cl$, Et_3N, CH_2Cl_2, RT.

Finally, acetyl-derived diketide SNACs were prepared from ethyl 2-methylacetoacetate **47b** (Scheme S3.1.4). Initial borohydride reduction to **48b** was followed by TBDMS-protection of the alcohol to give **49b**. Ester hydrolysis to acid **50b**, and then SNAC formation in the usual way gave the protected diketide thiolester **51b**. TBAF-mediated deprotection then gave a mixture of racemic diastereomers (±)-**7b** that were separated by HPLC. Tigloyl SNAC *E*-**8b** was synthesised from **51b** by mesylate mediated elimination.

3.2 General Methods

All reagents and solvents were purchased from commercial suppliers and used without further purification, unless otherwise stated. Diisopropylamine, triethylamine and acetic acid were distilled over recommended drying agents and directly used or stored under an inert nitrogen atmosphere over activated molecular sieves (3Å). All air and moisture sensitive reactions were carried out in oven-dried glassware equipped with a magnetic stirrer under a positive pressure of N_2 using standard Schlenk-line techniques. Anhydrous solvents were purchased from commercial suppliers. Reactions requiring cooling to -78 °C were conducted in a dry ice bath in acetone, 0 °C were conducted in an ice bath and equipped with a thermometer. Heating of reactions was achieved in a silicone oil bath equipped with a contact thermometer. Removal of organic solvents after each reaction was performed under reduced pressure using a rotary evaporator with a water bath temperature of 40 °C, unless otherwise stated. After aqueous work-up of reaction mixtures, organic solutions were routinely dried with anhydrous magnesium sulphate or sodium sulphate, filtered through a funnel equipped with cotton or paper filter. Flash chromatography was performed using silica gel from Macherey-Nagel (silica 60 M, particle diameter 40 - 63 µm), eluting with the solvent system stated in brackets. Column size and filling level were adapted to the individual purification. Columns were eluted under pressure and the fractions analysed by thin layer chromatography (TLC). TLC was carried out on ALUGRAM® Xtra SIL G/UV 254 from Macherey-Nagel (0.2 mm layer thickness, median pore size 60 Å, and with a fluorescent indicator). Plates were visualised under UV light at 254 nm or if necessary developed with a $KMnO_4$ or Cer(IV) staining solutions and developed with a heating gun. Reaction progress was controlled by either TLC or analytical LCMS.

3.3 Compound Synthesis and Characterisation

3.3.1 Compounds 7

Ethyl 3-hydroxy-2-methylbutanoate 48b[4]

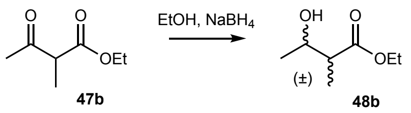

Ethyl 2-methyl-3-oxobutanoate **47b** (Sigma-Aldrich, 7.0 mL, 50 mmol) was dissolved in anhydrous ethanol (20 mL) under nitrogen. Sodium borohydride (0.95 g, 25 mmol) was added dropwise, and the mixture stirred at RT for 2 h, before concentration *in vacuo* to remove the solvent. Then, 20 mL water was added to the residue, and extracted with ethyl acetate (3 × 25 mL). The organic phases were combined, washed with brine, dried ($MgSO_4$), filtered and concentrated *in vacuo*, which yielded **48b**[4] (4.915 g, 33.62 mmol, 67%) as a colourless oil.

^{1}H-NMR (400 MHz, $CDCl_3$): δ = 4.16 (2 × 2H, q, *J* = 7.1 Hz, -OCH_2CH$_3$), 4.06 (1H, m, H-3), 3.87 (1H, m, H-3), 2.72 (1H, d, *J* = 5.8 Hz, 2-OH), 2.62 (1H, d, *J* = 4.8 Hz, 2-OH), 2.49 (1H, m, H-2), 2.43 (1H, m, H-2), 1.27 (2 × 3H, t, *J* = 7.2 Hz, -OCH$_2$CH_3), 1.22-1.16 (4 × 3H, 2-CH$_3$, and 3-CH$_3$).
ESI-MS (*m/z*): 147.2 $[M + H]^+$, 169.3 $[M + Na]^+$

Ethyl 3-((*tert*-butyldimethylsilyl)oxy)-2-methylbutanoate 49b[5]

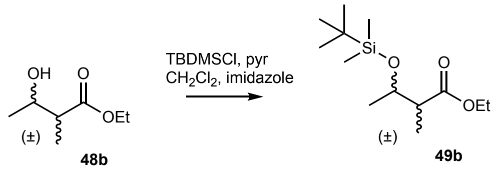

Ethyl 3-hydroxy-2-methylbutanoate, **48b** (3.655g, 25 mmol) was dissolved in anhydrous CH_2Cl_2 (20 mL) under nitrogen. Pyridine (4.04 mL, 50 mmol) and imidazole (3.0 g, 44.1mmol) was added. The mixture was cooled to 0 °C and *tert*-butyldimethylsilyl chloride (4.522 g, 30 mmol) dissolved in anhydrous CH_2Cl_2 (10 mL) was added dropwise. The reaction was stirred at RT overnight, after which it was quenched with saturated NH_4Cl aq. solution (32 mL). The layers were separated, and the aqueous layer was extracted with CH_2Cl_2 (2 × 30 mL). The organic phases were combined, washed with brine, dried ($MgSO_4$), filtered and concentrated *in vacuo*. Purification of the crude residue by flash chromatography (petroleum ether/EtOAc = 15/1) gave the title compound **49b**[5] (3.484 g, 13.38 mmol, 53.5%) as a colourless oil.

^{1}H-NMR (400 MHz, $CDCl_3$): δ = 4.11 (2 × 2H, m, -OCH_2CH$_3$), 4.01 (2 × 1H, m, H-3), 2.47 (1H, m, H-2), 2.38 (1H, m, H-2), 1.25 (2 × 3H, t, *J* = 7.2 Hz, -OCH$_2$CH_3), 1.15-1.07 (4 × 3H, 2-CH$_3$, and 3-CH$_3$), 0.86 (9H, s, SiC(CH$_3$)$_3$), 0.85 (9H, s, SiC(CH$_3$)$_3$), 0.05 (2 × 3H, s, SiCH$_3$), 0.02 (2 × 3H, s, SiCH$_3$).
ESI-MS (*m/z*): 261.4 $[M + H]^+$.

3-((*tert*-Butyldimethylsilyl)oxy)-2-methylbutanoic acid 50b[6]

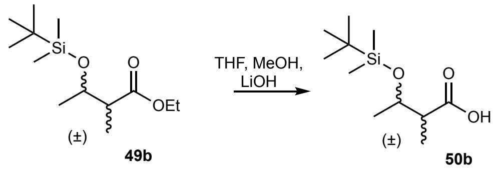

To ester **49b** (1.50 g, 5.76 mmol) in THF (5 mL), MeOH (12 mL) and lithium hydroxide (414 mg, 17.2 mmol) in water (2 mL) were added. The reaction mixture was heated to 60 °C overnight. The reaction was diluted with water (10 mL), and acidified to pH 1.0 with HCl (6 N) and extracted with EtOAc (3 × 25 mL). The organic layers were combined, washed with brine, dried ($MgSO_4$), filtered and concentrated *in vacuo* to give the acid **50b**[6] as a pale-yellow oil (1.188 g, 5.11 mmol, 89%).

^{1}H-NMR (400 MHz, $CDCl_3$): δ = 4.09 (1H, qd, *J* = 6.3, 5.1 Hz, H-3), 4.00 (1H, p, *J* = 6.1 Hz, H-3), 2.52 (2 × 1H, m, H-2), 1.20-1.14 (4 × 3H, 2-CH_3, and 3-CH_3), 0.89 (9H, s, $SiC(CH_3)_3$), 0.88 (9H, s, $SiC(CH_3)_3$), 0.10 (3H, s, $SiCH_3$), 0.08 (2 × 3H, s, $SiCH_3$), 0.07 (3H, s, $SiCH_3$);
^{13}C-NMR (100 MHz, $CDCl_3$): δ = 179.8/178.5 (C-1), 70.3/69.9 (C-3), 47.8/46.6 (C-2), 25.68/25.67 (Si-C(CH$_3$)$_3$), 21.0/20.4 (3-CH_3), 17.91/17.89 (Si-*C*(CH_3)), 13.4/11.9 (2-CH_3), -4.4/-4.5 (Si-CH_3), -5.1/-5.2 (Si-CH_3).
ESI-MS (*m/z*): (ESI +) 233.4 $[M + H]^+$; (ESI-) 231.2 $[M - H]^-$, 463.5 $[2M - H]^-$.

3-(*tert*-Butyldimethylsilyloxy)-2-methylbutyric acid *N*-acetylcysteamine thiolester 51b[7]

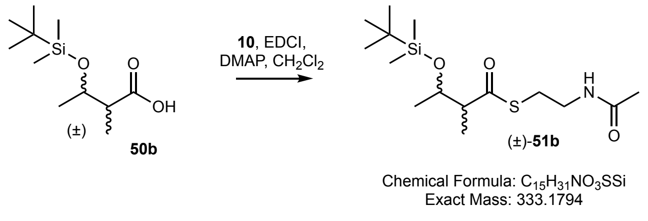

To a solution of acid **50b** (457 mg, 1.97 mmol) in anhydrous CH_2Cl_2 (18 mL) at 0 °C under nitrogen was added EDCI (418 mg, 2.68 mmol) and DMAP (50.0 mg, 0.41 mmol). After stirring at 0 °C for 15 minutes, HSNAC **29** (283 mg, 2.37 mmol) was added in anhydrous CH_2Cl_2 (10 mL). The mixture was allowed to warm to room temperature and stirred overnight. The reaction was quenched with water (20 mL) and the layers separated. The aqueous phase was extracted with CH_2Cl_2 (2 × 20 mL). The organic layers were combined, washed with brine, dried ($MgSO_4$), filtered and concentrated *in vacuo*. Purification by flash chromatography (CH_2Cl_2/MeOH, 32:1) gave the title compound **51b**[7] (561 mg, 1.68 mmol, 85%) as a pale yellow oil.

^{1}H-NMR (400 MHz, $CDCl_3$): δ = 5.82 (2 × 1H, brs, NH), 4.02 (2 × 1H, m, H-3), 3.43 (2 × 2H, m, N-CH_2), 3.00 (2 × 2H, m, S-CH_2), 2.71 (1H, m, H-2), 2.66 (1H, m, H-2), 1.96 (2 × 3H, s, CO-CH_3), 1.19 (3H, d, *J* = 7.0 Hz, 2-CH_3), 1.15 (3H, d, *J* = 6.1 Hz, 3-CH_3), 1.12 (3H, d, *J* = 6.3 Hz, 3-CH_3), 1.10 (3H, d, *J* = 7.1 Hz, 2-CH_3), 0.87 (9H, s, $SiC(CH_3)_3$), 0.85 (9H, s, $SiC(CH_3)_3$), 0.052 (3H, s, $SiCH_3$), 0.045 (3H, s, $SiCH_3$), 0.04 (3H, s, $SiCH_3$), 0.01 (3H, s, $SiCH_3$);
^{13}C-NMR (100 MHz, $CDCl_3$): δ = 203.0/202.9 (C-1), 170.14/170.12 (N-CO), 70.2/69.9 (C-3), 56.7/56.4 (C-2), 39.8/39.7 (N-CH_2), 28.4/28.3 (S-CH_2), 25.8/25.7 (Si-C(CH$_3$)$_3$), 23.2 (COCH$_3$), 21.8/20.7 (3-CH_3), 18.01/17. 95 (Si-*C*(CH_3)), 13.49/13.45 (2-CH_3), -4.30/-4.34 (Si-CH_3), -4.9/-5.2 (Si-CH_3).
ESI-MS (*m/z*): 334.5 $[M + H]^+$, 356.5 $[M + Na]^+$.

syn-3-Hydroxy-2-methylbutyric acid _N_-acetylcysteamine thiolester (±)-_syn_-7b[7] and _anti_-3-Hydroxy-2-methylbutyric acid _N_-acetylcysteamine thiolester (±)-_anti_-7b[7]

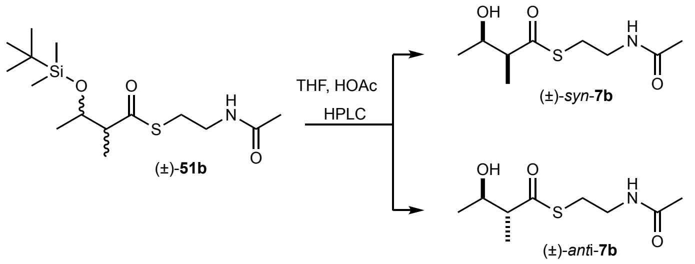

SNAC **51b** (20 mg, 0.06 mmol) was dissolved in THF (0.2 mL), water (0.6 mL) and acetic acid (0.6 mL) and stirred at room temperature for 2 days. The reaction mixture was dried using N_2 stream, and the residue was subjected to LCMS purification on a Kinetex® C_{18} column (i.d., 250 × 21.2 mm, 20 mL/min) eluting with 15% CH_3CN in H_2O to yield the racemic *syn* (3.8 mg, 0.017 mmol, 28%)[7] and *anti* (5.3 mg, 0.024 mmol, 40%),[7] diastereomers as colourless oils, respectively.

(±)-_syn_-7b: ^{1}H-NMR (400 MHz, $CDCl_3$): δ = 5.80 (1H, br. s, NH), 4.11 (1H, qd, *J* = 6.4, 4.0 Hz, H-3), 3.45 (2H, m, N-CH_2), 3.04 (2H, m, S-CH_2), 2.70 (1H, m, H-2), 2.39 (1H, br. s, OH), 1.97 (3H, s, CO-CH_3), 1.23 (3H, d, *J* = 7.1 Hz, 2-CH_3), 1.20 (3H, d, *J* = 6.4 Hz, 3-CH_3).
ESI-MS (*m/z*): 220.3 $[M + H]^+$, 242.3 $[M + Na]^+$.

(±)-_anti_-7b: ^{1}H-NMR (400 MHz, $CDCl_3$): δ = 5.81 (1H, br. s, NH), 3.95 (1H, m, H-3), 3.45 (2H, m, N-CH_2), 3.05 (2H, m, S-CH_2), 2.69 (1H, p, *J* = 7.1 Hz, H-2), 2.33 (1H, br. s, OH), 1.96 (3H, s, CO-CH_3), 1.24 (3H, d, *J* = 6.4 Hz, 3-CH_3), 1.20 (3H, d, *J* = 7.1 Hz, 2-CH_3).
ESI-MS (*m/z*): 220.3 $[M + H]^+$, 242.3 $[M + Na]^+$.

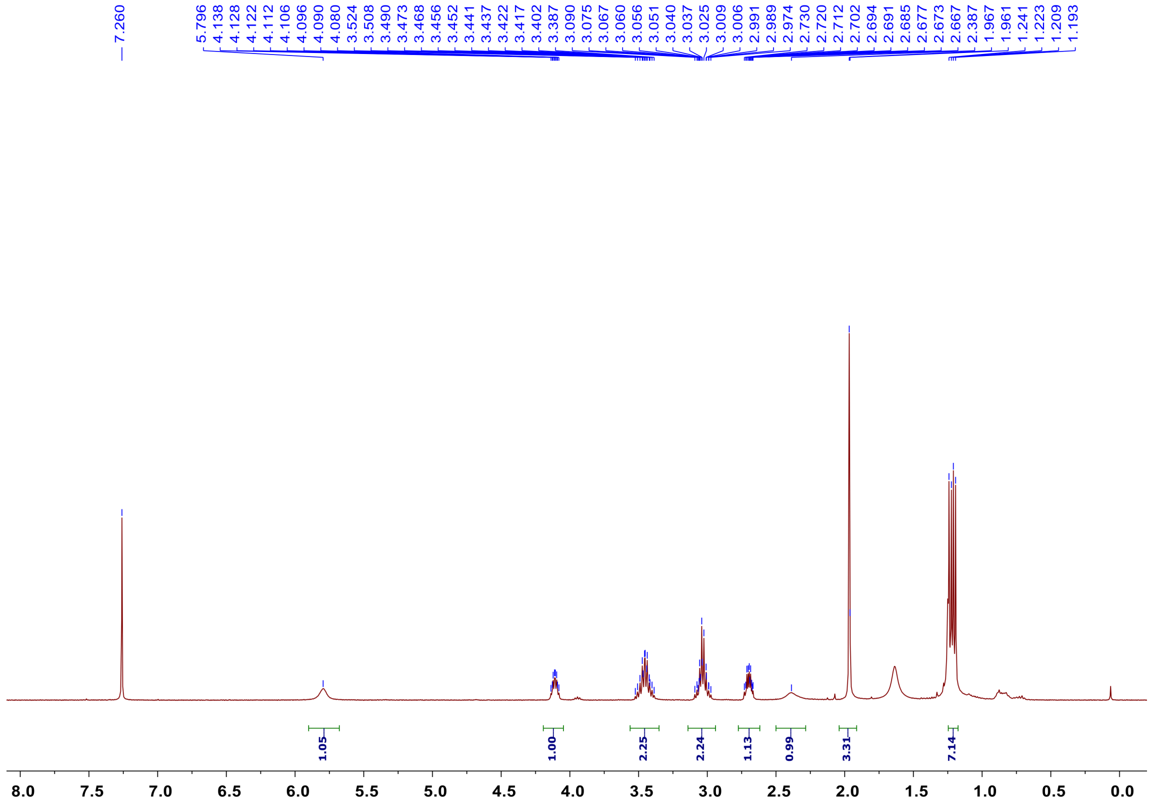

Figure S3.3.1A. ^{1}H NMR of (±)-*syn*-**7b** in $CDCl_3$

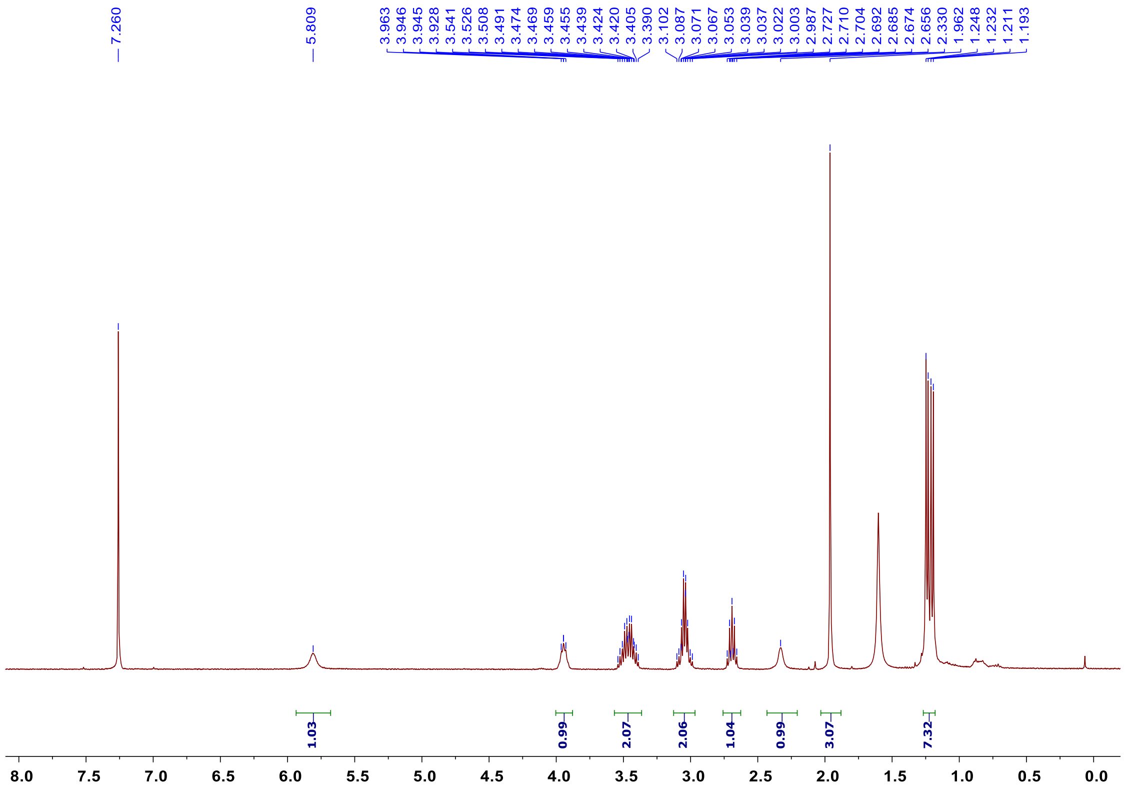

Figure S3.3.1B. ^{1}H NMR of (±)-*anti*-**7b** in $CDCl_3$

L-*anti*-7b

Synthesised following the procedure of Liddle and coworkers.[7]

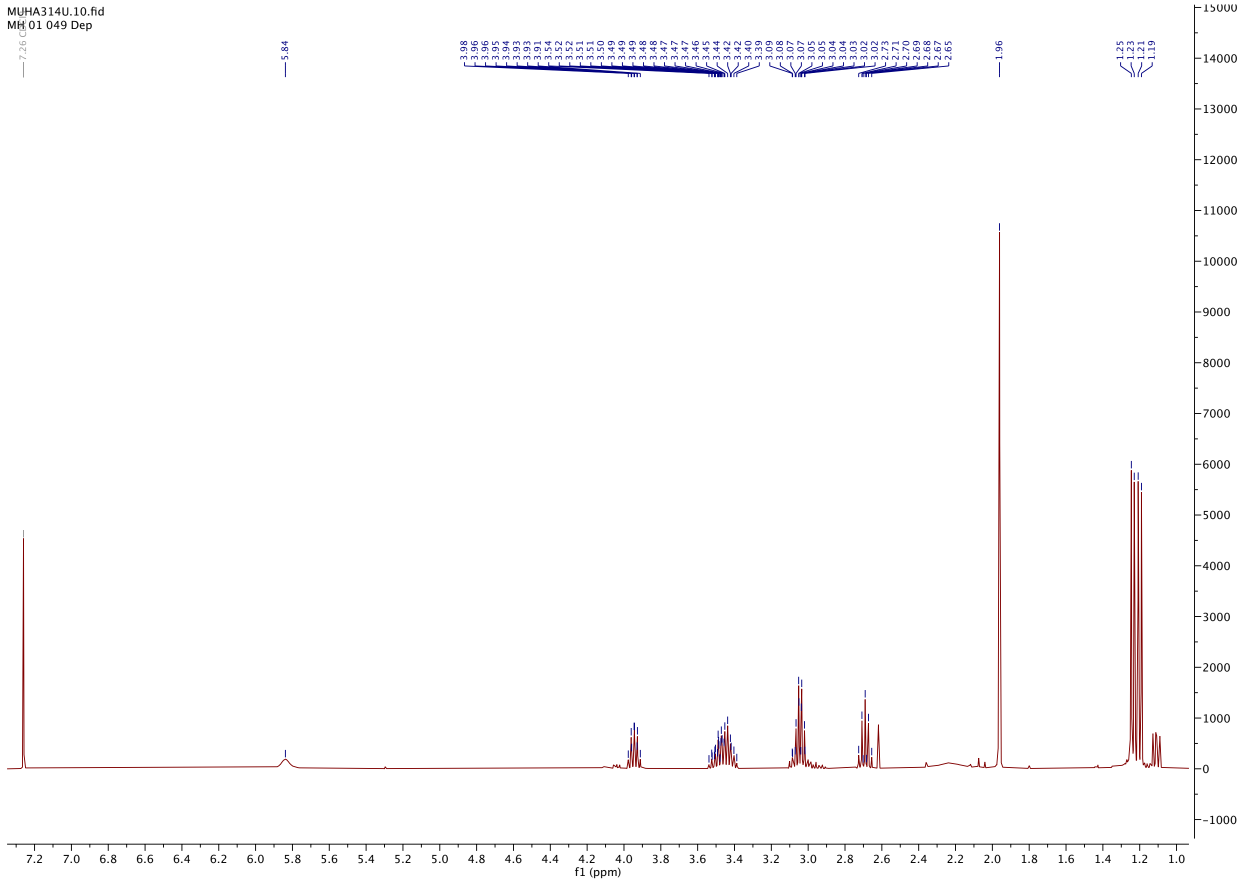

Figure S3.3.1C. ^{1}H NMR of L-*anti*-**7b** in $CDCl_3$.

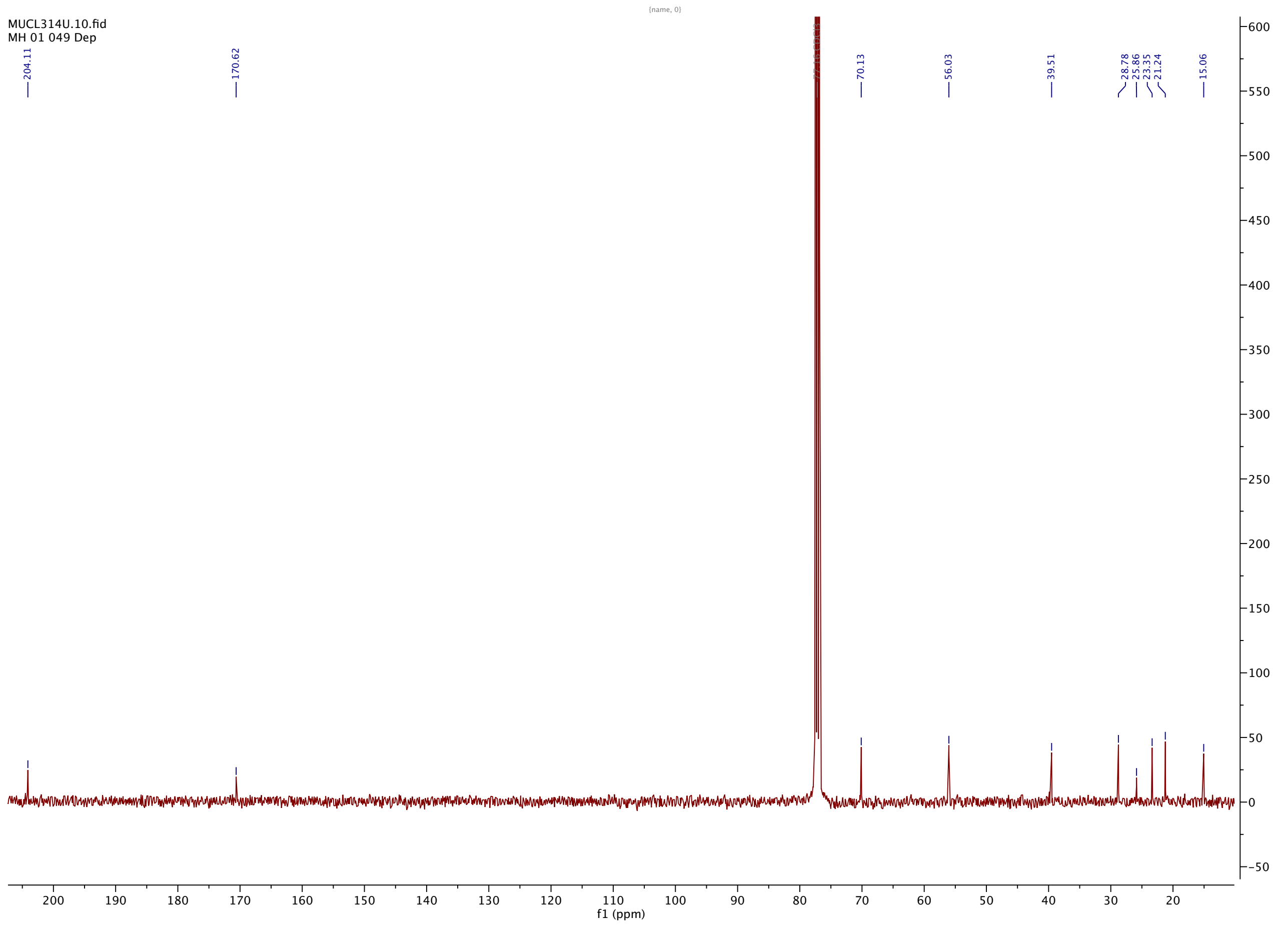

Figure S3.3.1D. ^{13}C NMR of L-*anti*-**7b** in $CDCl_3$.

D-*anti*-7b

Synthesised following the procedure of Liddle and coworkers.[7]

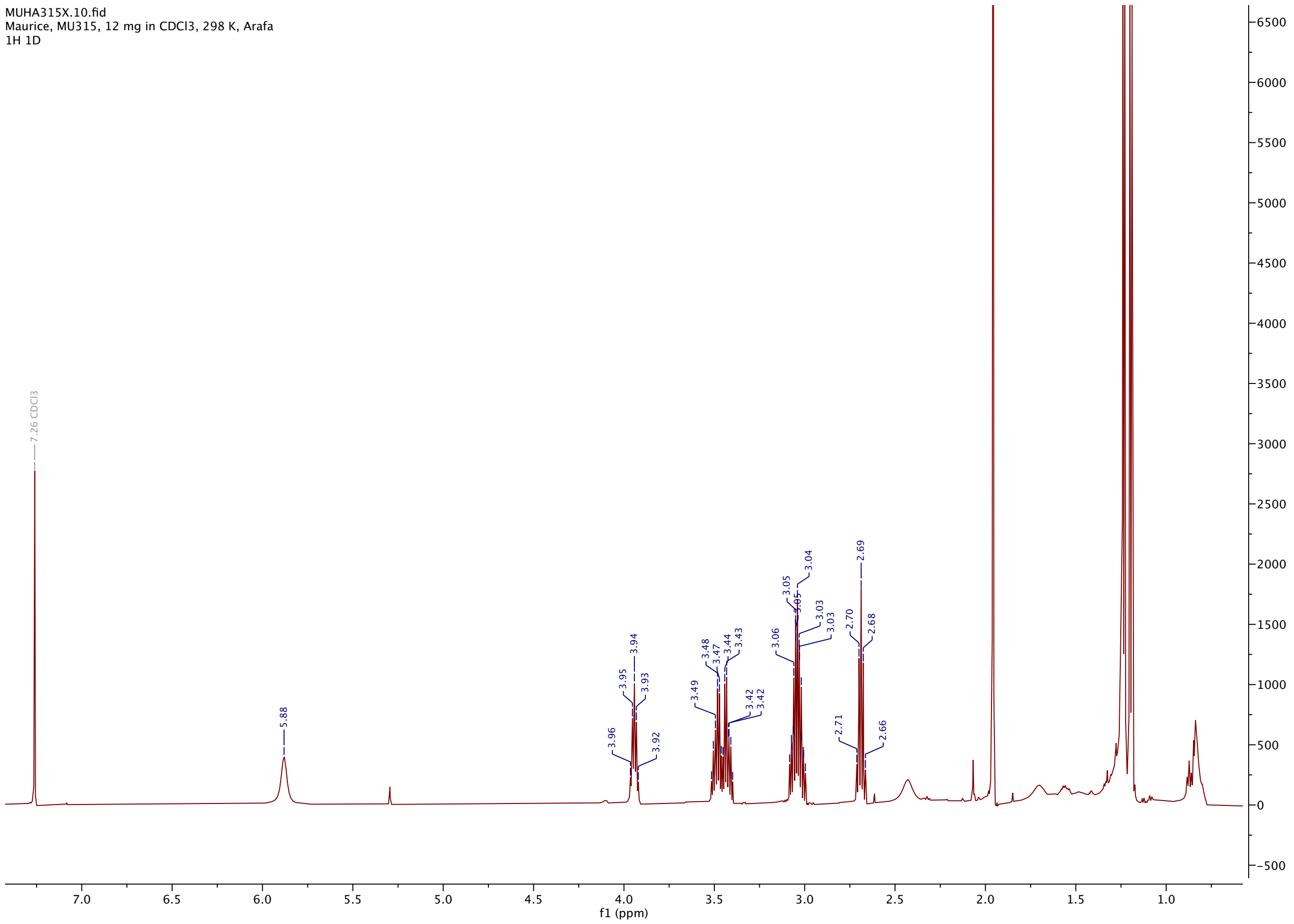

Figure S3.3.1E. ^{1}H NMR of D-*anti*-**7b** in $CDCl_3$.

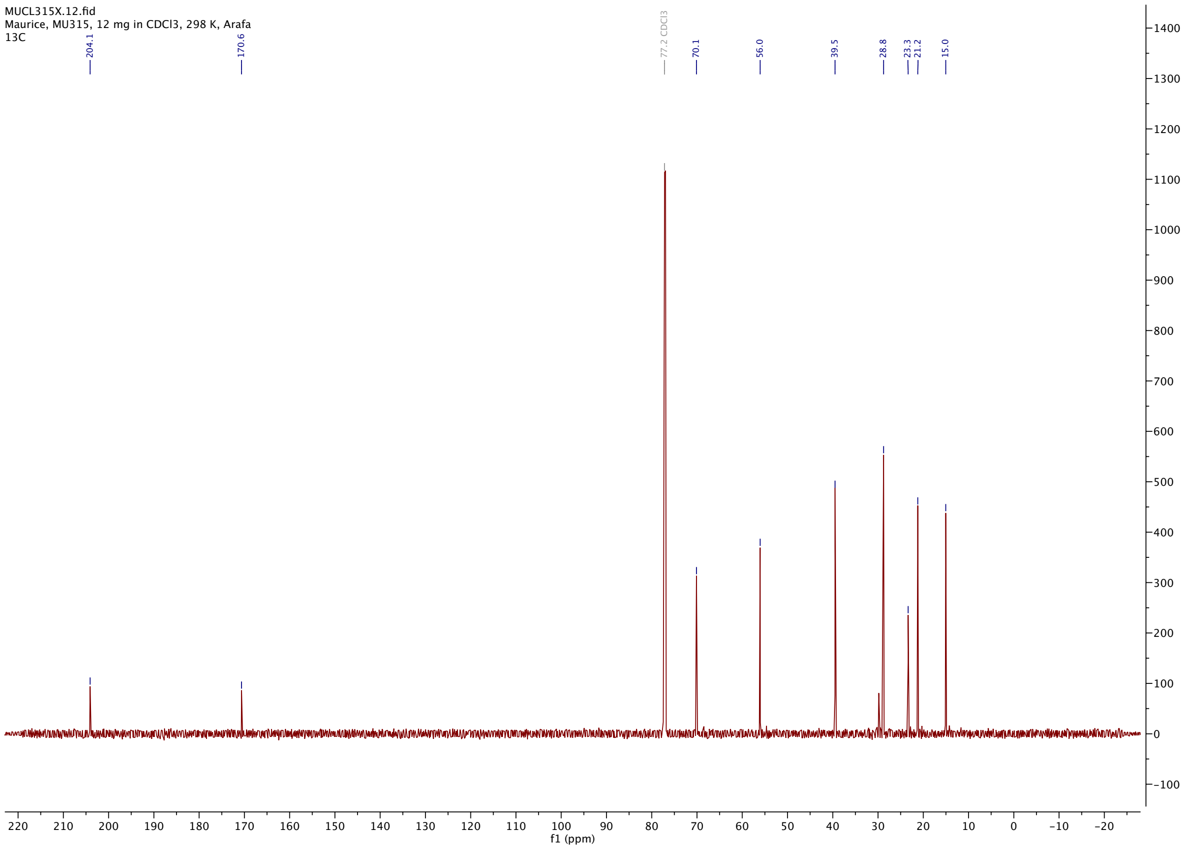

Figure S3.3.1F. ^{13}C NMR of D-*anti*-**7b** in $CDCl_3$.

3.3.2 Compounds 8b

S-(2-acetamidoethyl) *E*-2-methylbut-2-enethioate *E*-8b[10]

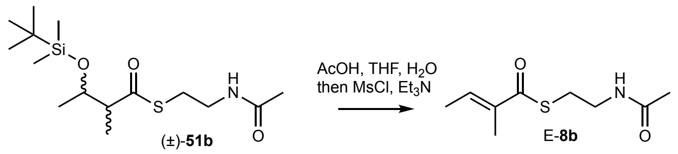

Compound **51b** (30 mg, 0.09 mmol) was dissolved in THF (0.3 mL), water (0.9 mL) and acetic acid (0.9 mL) and stirred at room temperature for 2 days. LCMS indicated all the starting material was consumed, and the alcohol was generated in good yield. Then, the solvent was removed *in vacuo* to give the alcohol as a colourless oil. The alcohol was dissolved in anhydrous CH_2Cl_2 (3 mL) under nitrogen, to which methanesulfonyl chloride (20 mg, 0.17 mmol) and triethylamine (39 mg, 0.39 mmol) were added. The mixture was stirred for 2 days before quench with saturated NH_4Cl aq. solution (10 mL), and layers were separated. The aqueous layer was extracted with CH_2Cl_2 (3 × 15 mL). The organic phases were combined, washed with brine, dried ($MgSO_4$), filtered and concentrated *in vacuo*. The residue was subjected to LC-MS preparation eluting with a gradient of acetonitrile in water to afford tigloyl-SNAC *E*-**8b**[3,10] as a colourless oil (3.5 mg, 0.017 mmol, 19% over two steps).

^{1}H-NMR (400 MHz, $CDCl_3$): δ = 6.87 (1H, qq, *J*=6.9, 1.3 Hz, H-3), 5.89 (1H, br. s, NH), 3.44 (2H, m, *N*-CH_2), 3.06 (2H, dd, *J*=6.8, 5.9 Hz, *S*-CH_2), 1.96 (3H, s, $COCH_3$), 1.87 (3H, q, *J*=1.2 Hz, 2-CH_3), 1.84 (3H, dq, *J*=7.0, 1.1 Hz, 3-CH_3);
^{13}C-NMR (100 MHz, $CDCl_3$): δ = 193.9 (*S*-CO), 170.2 (*N*-CO), 136.8 (C-2, C-3), 39.9 (*N*-CH_2), 28.3 (*S*-CH_2), 23.2 (*C*H_3CO), 14.5 (2-CH_3), 12.1 (3-CH_3).
ESI-MS (*m/z*): 202.2 $[M + H]^+$, 224.3 $[M + Na]^+$.

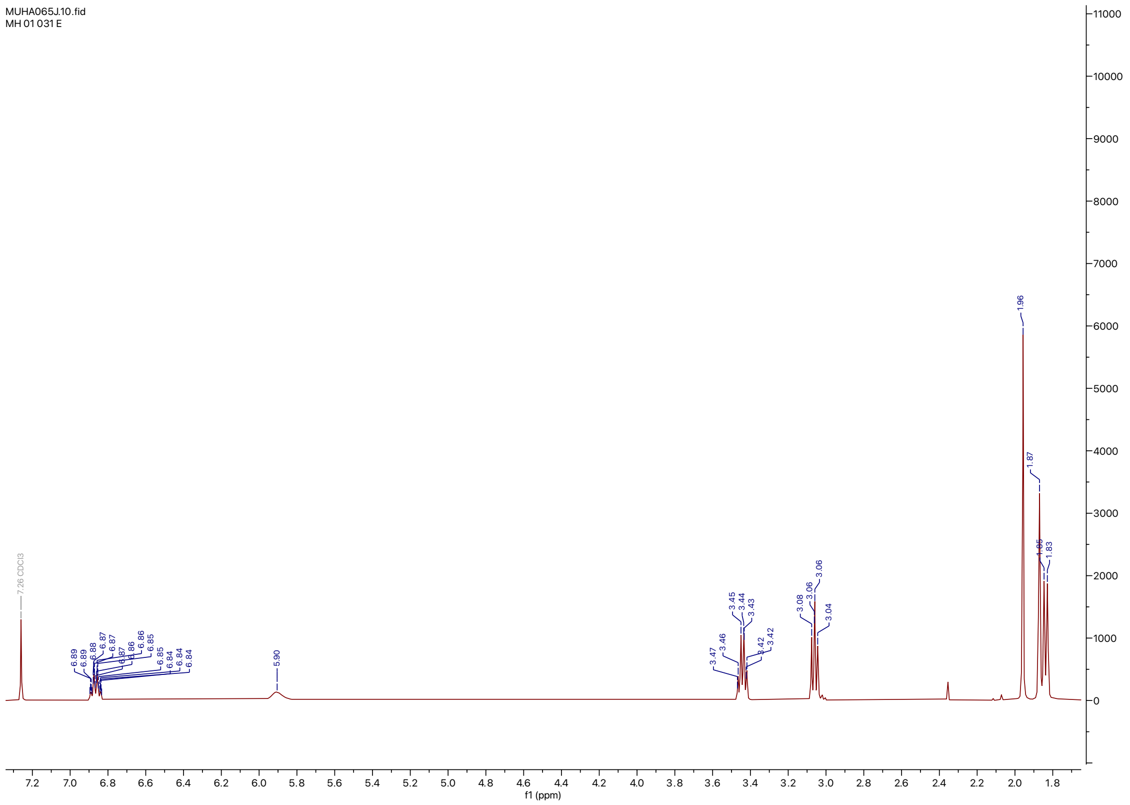

Figure S3.3.2A ^{1}H NMR spectrum of *E*-**8b** in $CDCl_3$.

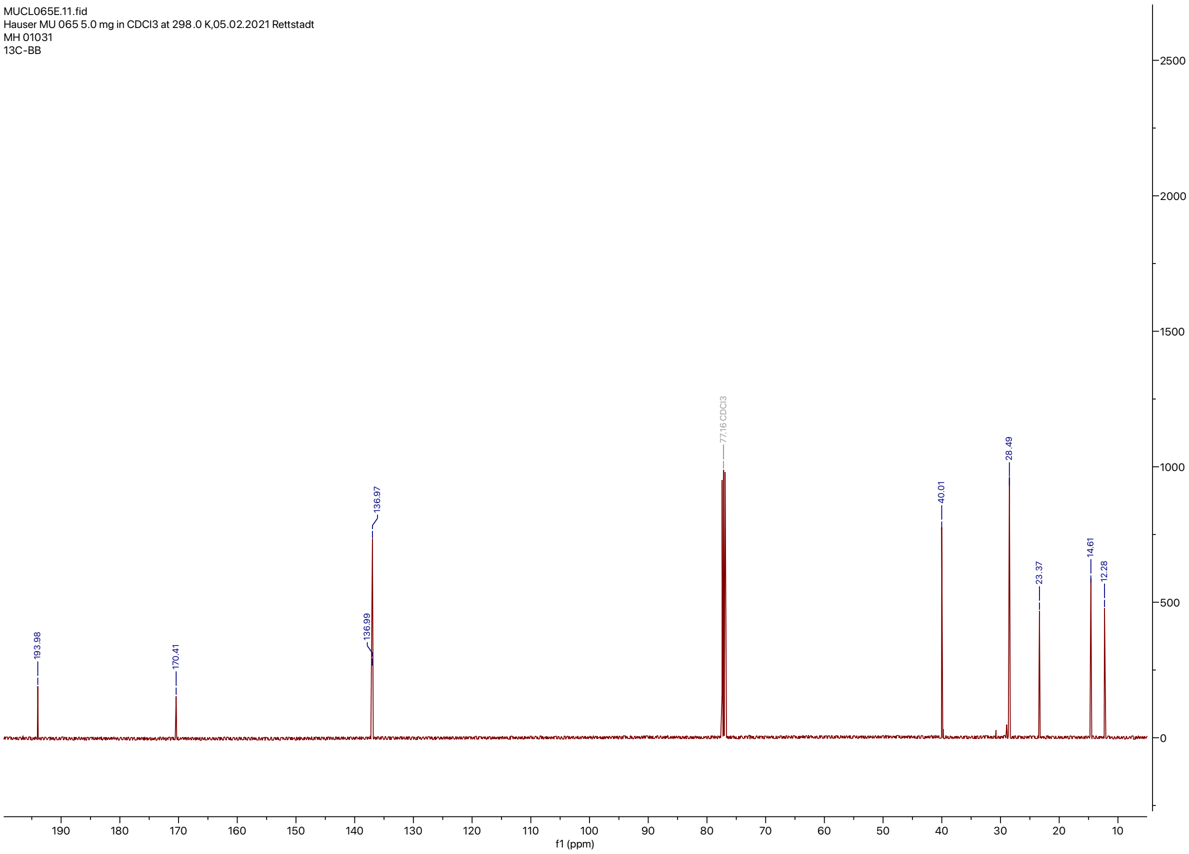

Figure S3.3.2B ^{13}C NMR spectrum of *E*-**8b** in $CDCl_3$.

S-(2-acetamidoethyl) *Z*-2-methylbut-2-enethioate *Z*-8b

Angelic acid (TCI, 41 mg, 0.41 mmol, 1.0 eq.) was dissolved in CH_2Cl_2 (5 mL) and cooled to 0 °C. After cooling EDCl (1.5 eq, 117 mg, 0.61 mmol) and DMAP (0.1 eq., 5 mg, 0.04 mmol) were added. The solution was allowed to stir for 5 min before HSNAC **29** (1.0 eq., 50 mg, 0.41 mmol) was added. After complete addition the reaction mixture was allowed to warm to room temperature and stirred for 3h. After completion the reaction was quenched by the addition of H_2O (6 mL). The phases were separated, and the aqueous phase was extracted with CH_2Cl_2 (2 × 5 mL). The combined organic phases were dried over $MgSO_4$, filtered and concentrated in *vacuo*. The crude residue was purified by flash chromatography (petroleum ether : ethyl acetate 1:3) to give the desired alkene (44 mg, 0.22 mmol, 53 %) as a pure diastereomer based on ^{1}H NMR.

^{1}H-NMR (400 MHz, $CDCl_3$): δ = 5.92 - 5.86 (2H, m, H-3, H-8), 3.47 (2H, q, *J* = 5.9 Hz, H-7), 3.09 (2H, dd, *J* = 6.7, 5.9 Hz, H-6), 1.99 (3H, p, *J* = 1.4 Hz, H-5), 1.97 (3H, s, H-10), 1.94 (3H, dq, *J* = 7.3, 1.5 Hz, H-5) ppm.
^{13}C-NMR (100 MHz, $CDCl_3$): δ = 194.5 (C-1), 170.4 (C-9), 134.9 (C-3), 134.4 (C-2), 40.0 (C-7), 28.4 (C-6), 23.4 (C-10), 20.7 (C-4), 16.0 (C-5) ppm.
ESI-MS (*m/z*): 202.2 [M + H]$^{+}$, 224.3 [M + Na]$^{+}$.

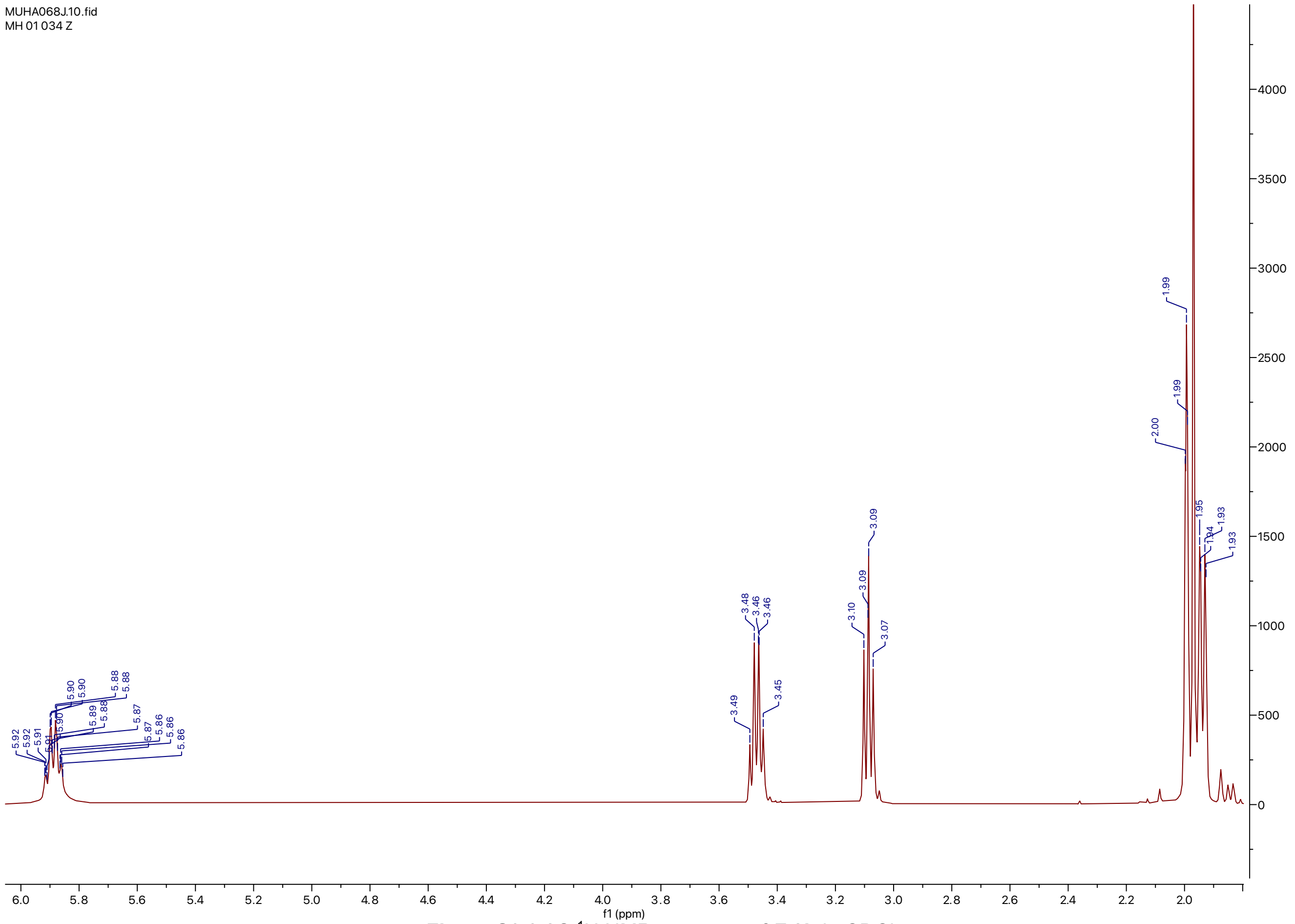

Figure S3.3.2C ^{1}H NMR spectrum of *Z*-**8b** in $CDCl_3$.

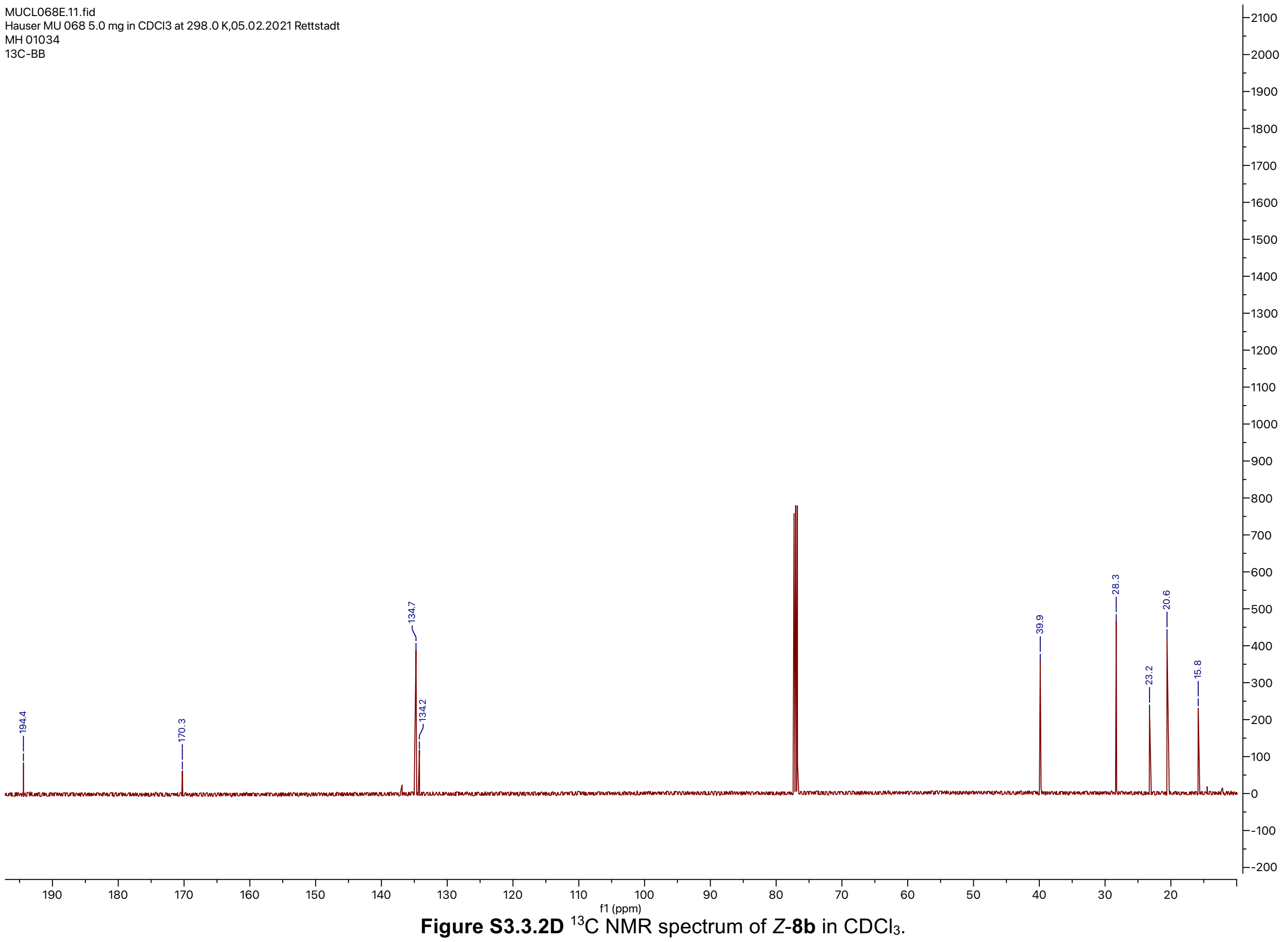

Figure S3.3.2D ^{13}C NMR spectrum of *Z*-**8b** in $CDCl_3$.

3.3.3 Compounds 9

Ethyl (2-methyl-3-phenyl-3-oxo)-propionate 27b[8]

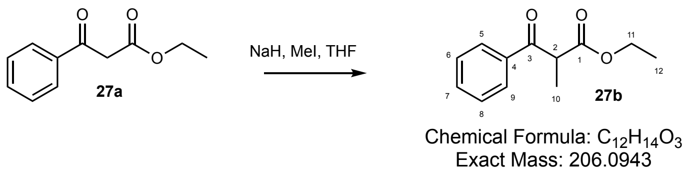

To a solution of ethyl benzoyl acetate **27a** (Sigma-Aldrich, 1 eq., 0.98 g, 5.1 mmol) in anhydrous THF (15 mL), sodium hydride (1.2 eq, 245 mg, 6.1 mmol, 60% dispersion in mineral oil) was added in portions. After stirring for 20 min, methyl iodide (1.2 eq., 0.38 ml, 6.1 mmol) was added dropwise. The mixture was stirred at RT for 3.5 h, then quenched with saturated aq. NH_4Cl (25 mL). The layers were separated, and the aqueous layer was extracted with ethyl acetate (2 × 30 mL). The organic phases were combined, washed with brine, dried over $MgSO_4$, filtered and concentrated *in vacuo*. Purification by flash chromatography over silica gel (petroleum ether/ethyl acetate, 10:1) gave the title compound **27b**[8] (476.3 mg, 2.31 mmol, 45%) as a colourless oil.

^{1}H-NMR (400 MHz, $CDCl_3$): δ = 7.97 (2H, d, *J* = 8.0 Hz, H-5/9), 7.57 (1H, m, H-7), 7.47 (2H, t-like, *J* = 7.6 Hz, H-6/8), 4.38 (1H, q, *J* = 7.1 Hz, H-2), 4.14 (2H, q, *J* = 7.1 Hz, H-10), 1.48 (3H, d, *J* = 7.1 Hz, H-12), 1.16 (3H, t, *J* = 7.1 Hz, H-11) ppm;
^{13}C-NMR (100 MHz, $CDCl_3$): δ = 196.0 (C-3), 170.9 (C-1), 135.8 (C-4), 133.4 (C-7), 128.7 (C-6/8), 128.5 (C-5/9), 61.4 (C-10), 48.3 (C-2), 13.9 (C-12), 13.8 (C-11).
ESI-MS (*m/z*): 207.3 $[M + H]^+$, 229.3 $[M + Na]^+$.

(±)-Ethyl (2-methyl-3-phenyl-3-hydroxy)-propionate 28b[9]

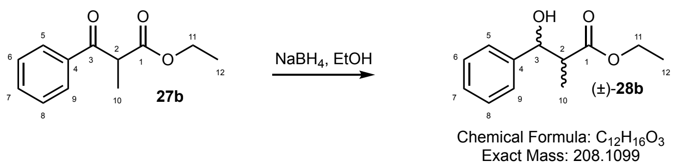

To a solution of Ethyl 2-benzoylpropionate **27b** (1 eq., 459.6 mg, 2.23 mmol) in anhydrous ethanol (8 mL), was added sodium borohydride (1.2 eq., 102 mg, 2.68 mmol) in portions. The suspension was stirred at RT for 70 min, before slowly quenching it with H_2O (15 mL). The aqueous phase was extracted with EtOAc (3 × 20 mL). The organic layers were combined, washed with brine, dried over $MgSO_4$, filtered and concentrated *in vacuo* to give the crude alcohol. Purification by flash chromatography over silica gel (petroleum ether/acetone 10:1) gave the title compound (±)-**28b**[9] as a mixture of diastereomers (264.4 mg, 1.27 mmol, 57%) as a colourless oil.

^{1}H-NMR (400 MHz, $CDCl_3$): δ = 7.38 - 7.24 (10H, m, ArH), 5.08 (1H, dd, *J* = 4.3, 3.0 Hz, H-3), 4.74 (1H, dd, *J* = 8.4, 4.3 Hz, H-3), 4.18 (3H, q, *J* = 7.1 Hz, H-11), 4.12 (3H, q, *J* = 7.1 Hz, H-11), 3.05 (1H, dd, *J* = 4.5, 2.1 Hz, OH), 3.01 (1H, dd, *J* = 3.3, 1.9 Hz, OH), 2.84 - 2.71 (2H, m, H-2), 1.25 (6H, t, *J* = 7.1 Hz, H-12), 1.02 (3H, d, *J* = 7.2 Hz, H-10), 1.13 (3H, d, *J* = 7.1 Hz, H-10) ppm.
^{13}C-NMR (100 MHz, $CDCl_3$): δ = 175.82/175.79 (C-1), 141.6/141.4 (C-4), 128.0/127.4 (C-7), 128.4/128.2 (C-6/8), 126.6/126.0 (C-5/9), 76.3/73.6 (C-3), 60.74/60.69 (C-11), 47.1/46.4 (C-2), 14.5/10.8 (C-10) , 14.12/14.05 (C-12) ppm.

ESI-MS (*m/z*): 191.3 $[M + H - H_2O]^+$, 209.3 $[M + H]^+$, 231.3 $[M + Na]^+$.

3*R*-3-hydroxy-3-phenylpropanoyl-*N*-acetylcysteamine thiolester L-9a and 3*S*-3-hydroxy-3-phenylpropanoyl-*N*-acetylcysteamine thiolester D-9a

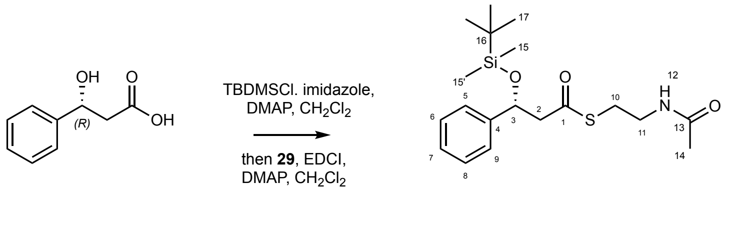

To a solution of (*R*)-3-hydroxy-3-phenylpropanoic acid (ThermoFisher, 1.0 eq., 59 mg, 0.35 mmol) in CH_2Cl_2 (2 mL), imidazole (4.0 eq., 97 mg, 1.42 mmol) and DMAP (0.1 eq., 3 mg, 0.03 mmol) were added, followed by the final addition of TBDMSCl (3.0 eq., 160 mg, 1.07 mmol). The reaction mixture was stirred 3 h before it was quenched with the addition of sat. aqueous $NaHCO_3$ (2 mL). This mixture was vigorously stirred for an additional 10 min before the phases were separated. The aqueous phase was extracted with CH_2Cl_2 (2 × 3 mL), dried over $MgSO_4$, filtered and concentrated in *vacuo*. The crude residue was used without any further purification for the next step. The 3-*O*-TBDMS protected acid was dried under high vacuum prior to dissolving it in CH_2Cl_2 (3 mL). The solution was cooled to 0 °C and EDCl (2.5 eq, 138 mg, 0.88 mmol) and DMAP (0.2 eq., 8.7 mg, 0.07 mmol) were added. The solution was allowed to stir for 10 min before HSNAC **29** (1.2 eq., 51 mg, 0.43 mmol) was added. The mixture was allowed to stir and warm to room temperature overnight before it was quenched with the addition of H_2O (2 mL). The aqueous phase was extracted with CH_2Cl_2 (2 × 3 mL) and the combined organic phases were dried over Na_2SO_4, filtered and concentrated in *vacuo*. The crude oily residue was purified by flash chromatography (ethyl acetate/petroleum ether 1:1) to give a colourless oil (78 mg, 0.20 mmol, 58%).

$[\alpha]_D^{20}$ (c = 0.20 in $CHCl_3$) = + 70.1
^{1}H-NMR (400 MHz, $CDCl_3$): δ = 7.32 (4H, d, *J* = 4.3 Hz, ArH), 7.30 - 7.24 (1H, m, ArH), 5.86 (1H, brs, H-12), 5.15 (1H, dd, *J* = 8.9, 4.1 Hz, H-3), 3.41 (2H, p, *J* = 6.3 Hz, H-11), 3.08 - 2.94 (3H, m, H-10 & H-2), 2.75 (1H, dd, *J* = 14.3, 4.2 Hz, H-2), 1.97 (3H, s, H-14), 0.84 (9H, s, H-17), 0.00 (3H, s, H-15), -0.19 (3H, s, H-15') ppm.
^{13}C-NMR (100 MHz, $CDCl_3$): δ = 197.7 (C-1), 170.5 (C-13), 143.7 (C-4), 128.5 (C-6/8), 127.8 (C-7), 126.0 (C-5/9), 72.4 (C-3), 55.2 (C-2), 39.7 (C-11), 28.9 (C-10), 25.8 (C-17), 23.3 (C-16), 18.3 (C-14), -4.6 (C-15), -5.2 (C-15') ppm.
ESI-MS (*m/z*): 382.1 $[M + H]^+$, 404.2 $[M + Na]^+$.
HRESIMS (*m/z*): calculated for $C_{19}H_{31}NO_3SSiNa$ $[M + Na]^+$: 404.1686 found 404.1688

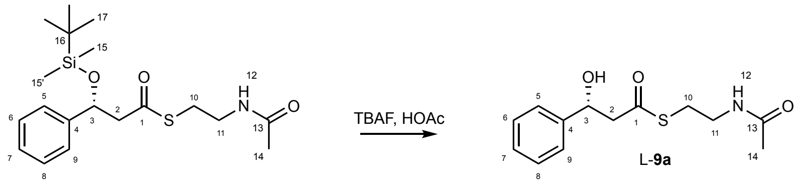

Chemical Formula: $C_{13}H_{17}NO_3S$
Exact Mass: 267.0929

The 3*S*-3-*O*-TBDMS protected SNAC as prepared above (1.0 eq, 30 mg, 0.078 mmol) was dissolved in THF (1 mL) and cooled to 0 °C. To the cooled reaction mixture was added a mixture of TBAF (5 eq., 400 µL, 0.39 mmol, 1M in THF) and acetic acid (7 eq., 32 µL, 0.56 mmol) in THF (0.5 mL) dropwise. The mixture was allowed to slowly warm to room temperature and stirred for up to 3 days, monitored by LCMS. After completion, the reaction was quenched by the addition of sat. aqueous NH_4Cl (3

mL) and extracted with Et_2O (3 × 2 mL). The combined organic phases were washed additionally with sat. aqueous NH_4Cl (3 mL), dried over Na_2SO_4, filtered and concentrated in *vacuo*. This furnished L-**9a** as a colourless oil (14 mg, 0.05 mmol, 70%) without any additional purification.

[α]$_D$^{25} (c 0.12 in $CHCl_3$) = + 25.07 (lit. **[α]$_D$^{20}** (c 0.20, $CHCl_3$) = + 21.2)[10]

^{1}H-NMR (400 MHz, $CDCl_3$): δ = 7.38-7.29 (5H, m, ArH), 5.87 (1H, brs, H-12), 5.19 (1H, dd, *J* = 9.2, 3.6 Hz, H-3), 3.41 (2H, m, H-11), 3.03 (3H, m, H-2 & H-10), 2.93 (1H, dd, *J* = 15.6, 3.6 Hz, H-2), 1.94 (3H, s, H-14) ppm.

^{13}C-NMR (100 MHz, $CDCl_3$): δ = 198.9 (C-1), 170.9 (C-13), 142.4 (C-4), 128.8 (C-6/8), 128.1 (C-7), 125.8 (C-5/9), 71.1 (C-3), 52.9 (C-2), 39.4 (C-11), 29.0 (C-10), 23.3 (C-14) ppm.

ESI-MS (*m/z*): 250.1 [M - H_2O + H]$^+$, 268.1 [M + H]$^+$.

HRESIMS (*m/z*): calculated for $C_{13}H_{17}NO_3SNa$ [M + Na]$^+$: 290.0821 found 290.0827

D-9a

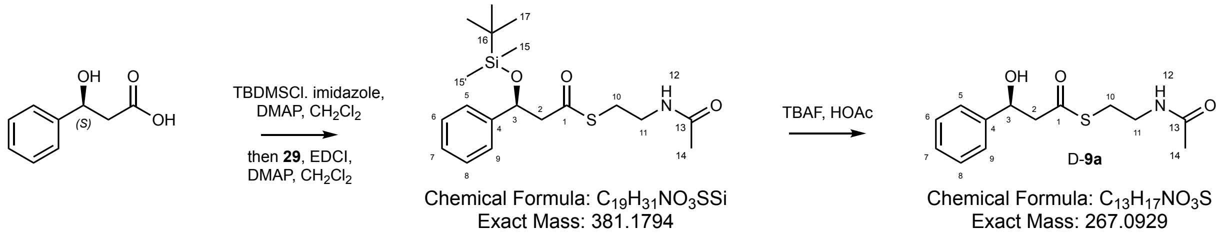

Same procedure as L-**9a** starting from (*S*)-3-hydroxy-3-phenylpropanoic acid.

[α]$_D$^{20} (c 0.13 in $CHCl_3$) = - 78.4 (*O*-TBDMS protected SNAC)

[α]$_D$^{20} (c 0.14 in $CHCl_3$) = - 18.7 (lit. **[α]$_D$^{20}** (c 0.50, $CHCl_3$) = - 18.2)[10, 11]

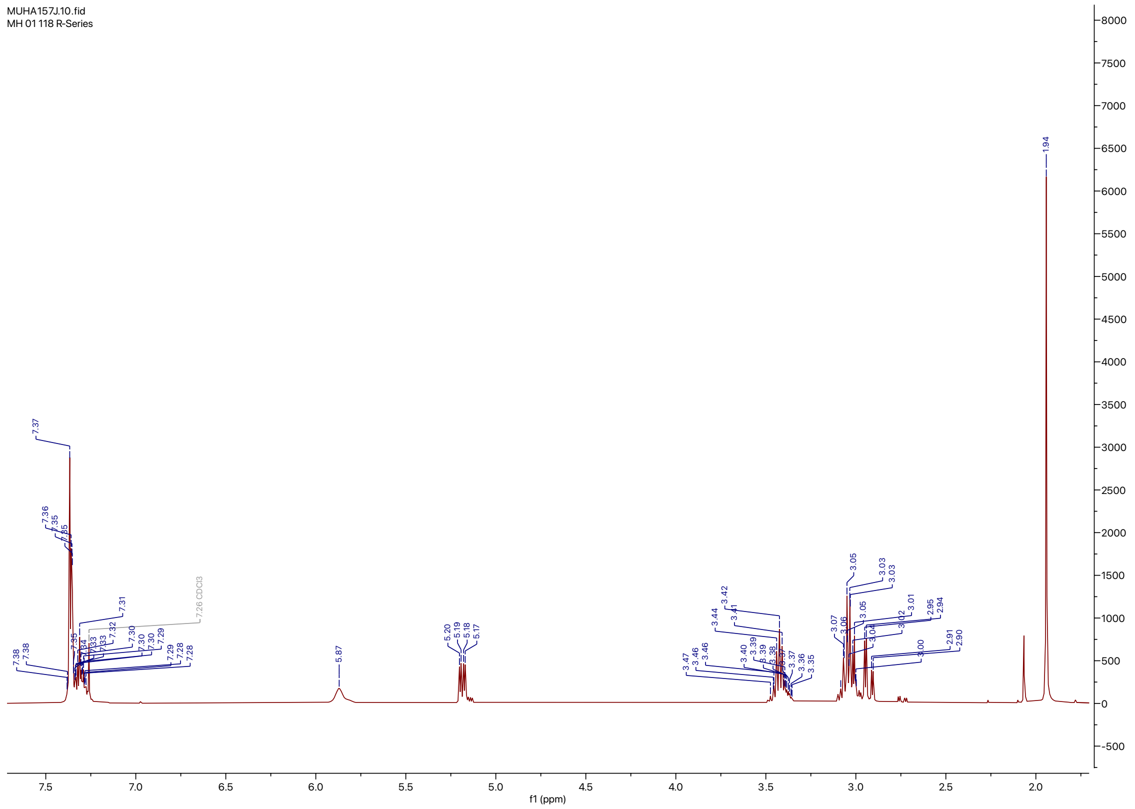

Figure S3.3.3A ^{1}H NMR of L-**9a** in $CDCl_3$.

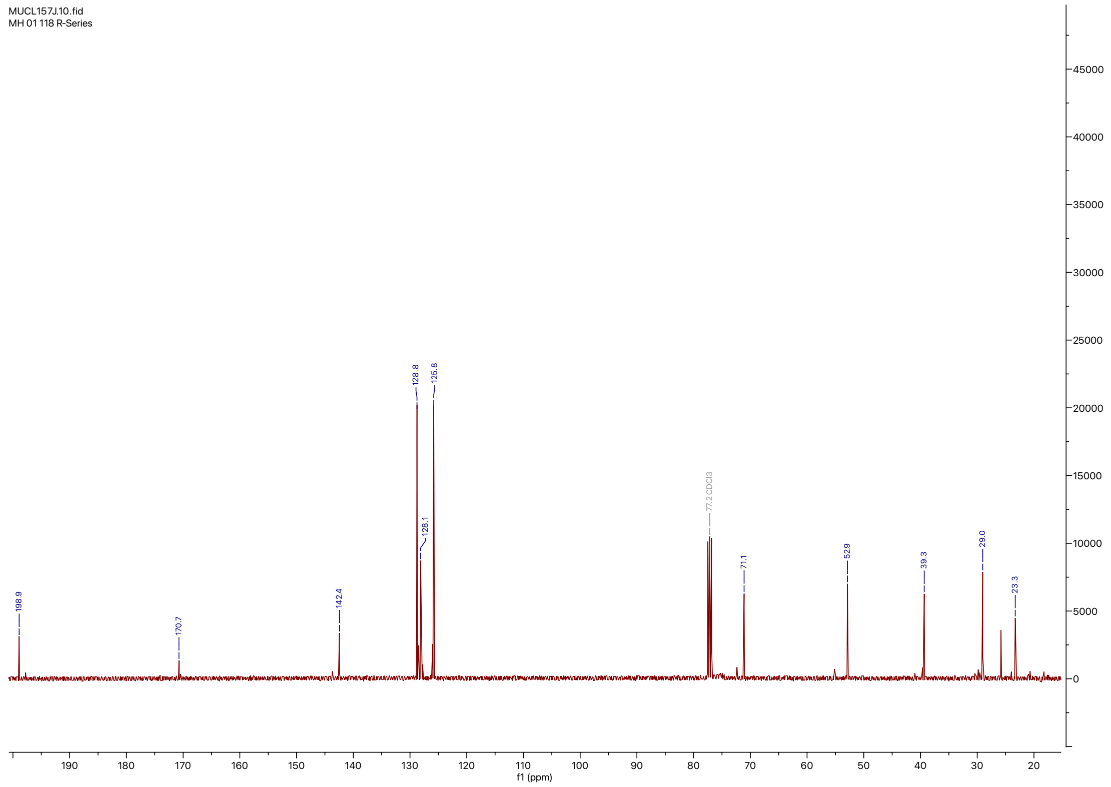

Figure S3.3.3A ^{13}C NMR of L-**9a** in $CDCl_3$.

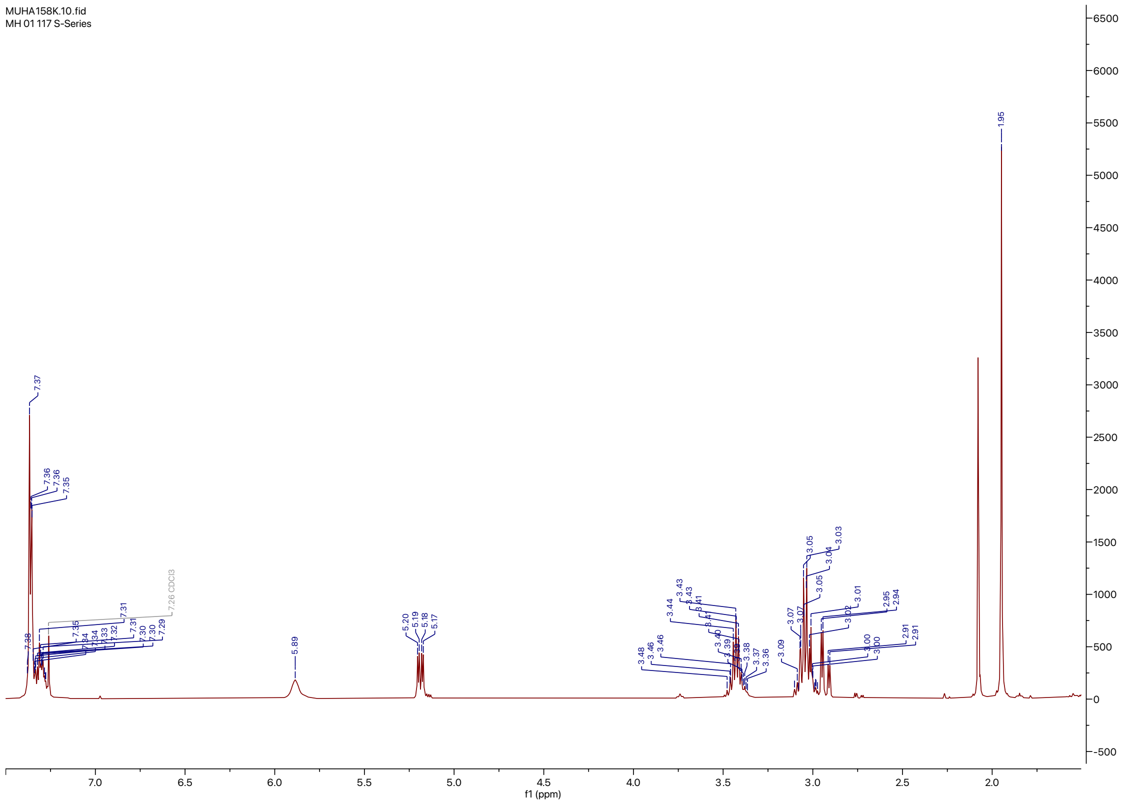

Figure S3.3.3C ^{1}H NMR of D-**9a** in $CDCl_3$.

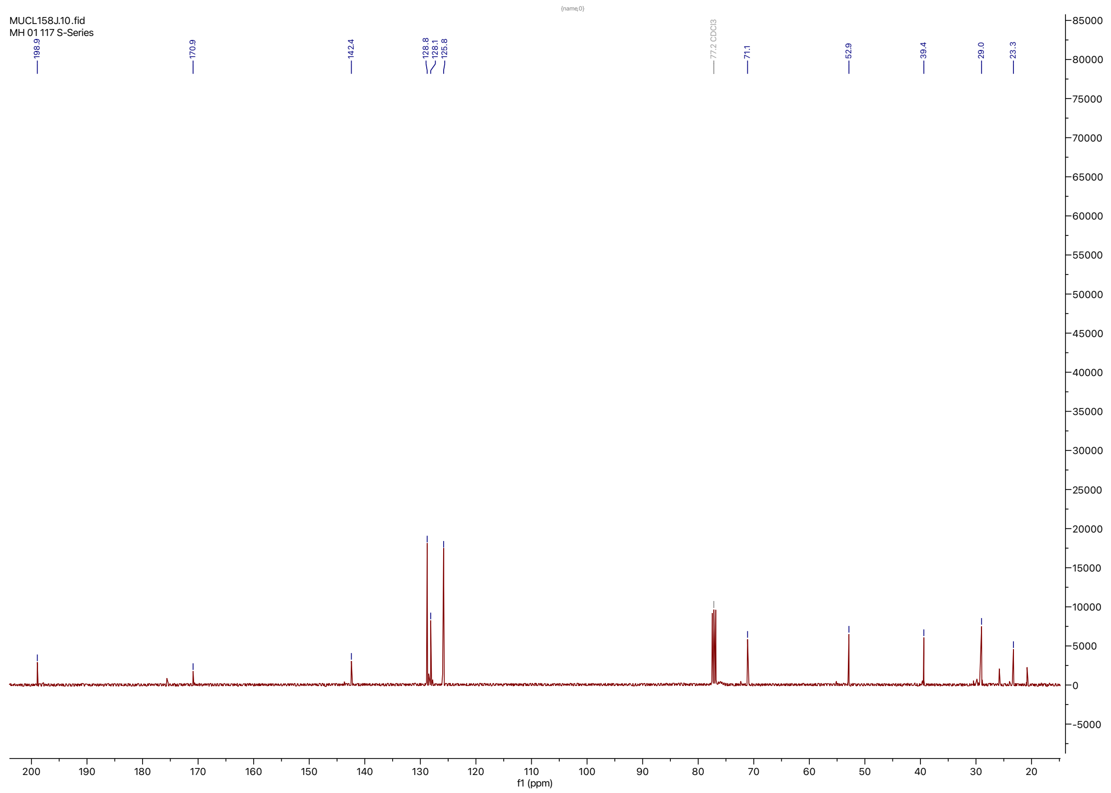

Figure S3.3.3D ^{13}C NMR of D-**9a** in $CDCl_3$.

***S*-(2-acetamidoethyl) (2*R**,3*R**)-3-hydroxy-2-methyl-3-phenylpropanethioate ± *syn*-9b[12] and *S*-(2-acetamidoethyl) (2*S**,3*R**)-3-hydroxy-2-methyl-3-phenylpropanethioate ± *anti*-9b[12]**

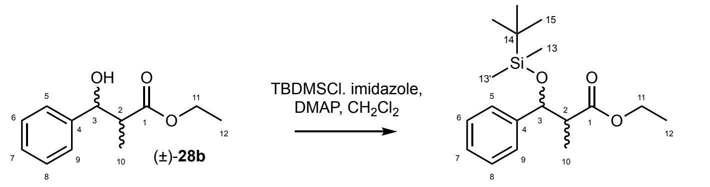

(±)-Ethyl 3-hydroxy-2-methyl-3-phenylpropanoate **28b** (1 eq., 316.4 mg, 1.52 mmol) was dissolved in anhydrous CH_2Cl_2 (4 mL) under nitrogen and imidazole (1.2 eq., mg, mmol) was added. Tert-butyldimethylsilyl chloride (1.2 eq., mg, mmol) was dissolved in anhydrous CH_2Cl_2 (1.3 mL) prior addition and then added dropwise. The reaction was stirred at RT overnight followed by the addition of H_2O (10 mL). The phases were separated, and the aqueous layer was extracted with CH_2Cl_2 (3 × 10 mL). The organic phases were combined, washed with brine, dried over $MgSO_4$, filtered and concentrated *in vacuo*. Purification of the crude residue by flash chromatography (petroleum ether/EtOAc=19:1) gave ethyl ester (305.3 mg, 0.95 mmol, 62%) as a colourless oil and as a diastereomeric mixture (*anti/syn* ≈ 3.3/1) based on ^{1}H NMR.[12]

anti: **^{1}H-NMR** (400 MHz, $CDCl_3$): δ = 7.36 - 7.19 (5H, m, ArH), 4.71 (1H, d, *J* = 9.3 Hz, H-3), 4.16 (2H, m, H-11), 2.73 (1H, dq, *J* = 9.2, 7.1 Hz, H-2), 1.29 (3H, t, *J* = 7.1 Hz, H-12), 0.84 (3H, d, *J* = 7.0 Hz, H-10), 0.80 (9H, s, H-15), -0.02 (3H, s, H-13), -0.30 (3H, s, H-13') ppm.

^{13}C-NMR (100 MHz, $CDCl_3$): δ = 175.4 (C-1), 142.3 (C-4), 128.1 (C-6/8), 127.7 (C-7), 127.1 (C-5/9), 77.7 (C-3), 60.3 (C-11), 49.4 (C-2), 25.6 (C-15), 17.9 (C-14), 14.2 (C-10), 13.9 (C-12), -4.7 (C-13), -5.4 (C-13') ppm.

ESI-MS (*m/z*): 323.4 $[M + H]^+$, 345.4 $[M + Na]^+$.

syn: **^{1}H-NMR** (400 MHz, $CDCl_3$): δ = 7.36 - 7.19 (5H, m, ArH), 4.96 (1H, d, *J* = 6.0 Hz, H-3), 3.99 (2H, m, H-11), 2.65 (1H, m, H-2), 1.10 (3H, t, *J* = 7.1 Hz, H-12), 1.16 (3H, d, *J* = 7.0 Hz, H-10), 0.87 (9H, s, H-15), 0.02 (3H, s, H-13), -0.22 (3H, s, H-13‘) ppm;

^{13}C-NMR (100 MHz, $CDCl_3$): δ = 174.3 (C-1), 143.2 (C-4), 127.9 (C-6/8), 127.3 (C-7), 126.5 (C-5/9), 76.0 (C-3), 60.2 (C-11), 49.2 (C-2), 25.7 (C-15), 18.1 (C-14), 11.8 (C-10), 14.0 (C-12), -4.6 (C-13), -5.3 (C-13‘) ppm;

ESI-MS (*m/z*): 323.4 $[M + H]^+$, 345.4 $[M + Na]^+$.

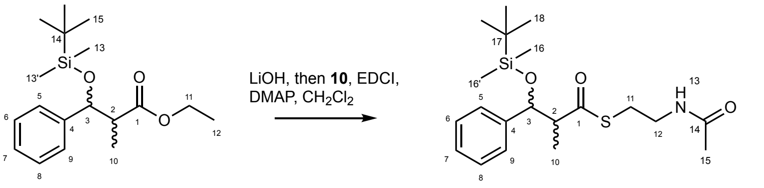

Chemical Formula: $C_{20}H_{33}NO_3SSi$
Exact Mass: 395.1950

To the ethyl ester prepared above (1 eq., 285 mg, 0.88 mmol) in THF (1 mL), were added MeOH (2.4 mL) and $LiOH{\cdot}H_2O$ (3 eq., 63.5 mg, mmol) in water (0.4 mL). The reaction mixture was heated to 60 °C overnight. The reaction was diluted with water (10 mL) and acidified to pH 1.0 with HCl (6 M) and extracted with EtOAc (3 × 12 mL). The organic layers were combined, washed with brine, dried ($MgSO_4$), filtered and concentrated *in vacuo* to give the free acid as a colourless oil (206 mg, 0.70 mmol, 80%). The residue was used without further purification or characterisation. The solution of the crude residue (1 eq., 188 mg, 0.64mmol) in anhydrous CH_2Cl_2 (4 mL) was cooled to 0 °C prior the addition of EDCI (1.2 eq, 147 mg, 0.77 mmol), DMAP (0.13 eq, 10 mg, 0.08 mmol) and HSNAC (1.3 eq., 96.3 mg, 0.81 mmol). The mixture was allowed to warm to RT and stirred overnight. The reaction was quenched with water (5 mL) and the layers were separated. The aqueous phase was extracted with CH_2Cl_2 (2 × 5 mL). The combined organic layers were washed with brine, dried over $MgSO_4$, filtered and concentrated *in vacuo*. The crude residue was purified by flash chromatography (ethyl acetate / petroleum ether 1:1) to give the diastereomeric mixture of 3-*O*-TBDMS protected SNACs (208.9 mg, 0.53 mmol, 82%) as a pale yellow oil (*dr.* 2/1) based on ^{1}H NMR.

major (anti): **$UV_{\lambda max}$** (CH_3CN:H_2O): 230 nm. **^{1}H-NMR** (400 MHz, $CDCl_3$): 7.41 - 7.22 (5H, m, ArH), 5.87 (1H, brs, H-13), 4.74 (1H, d, *J* = 9.1 Hz, H-3), 3.46 (2H, m, H-12), 3.14-2.86 (3H, m, H-11 & H-2), 1.97 (3H, s, H-15), 0.82 (3H, d, *J* = 6.9 Hz, H-10), 0.80 (9H, s, H-18), -0.05 (3H, s, H-16), -0.33 (3H, s, H-16’) ppm.

^{13}C-NMR (100 MHz, $CDCl_3$): δ = 203.2 (C-1), 170.1 (C-14), 142.0 (C-4), 128.2 (C-6/8), 127.9 (C-7), 127.0 (C-5/9), 77.5 (C-3), 57.9 (C-2), 39.7 (C-12), 28.6 (C-11), 25.6 (C-18), 23.23 (C-15), 18.0 (C-17), 14.8 (C-10), -4.7 (C-16), -5.5 (C-16’) ppm.

ESI-MS (*m/z*): 396.4 $[M + H]^+$, 418.4 $[M + Na]^+$.

HRMS (ESI): calculated for $C_{20}H_{34}NO_3SSi$ $[M + NH]^+$: 396.2029; found: 396.2029

minor (syn): **$UV_{\lambda max}$** (CH_3CN:H_2O): 230 nm. **^{1}H-NMR** (400 MHz, $CDCl_3$): δ = 7.41 - 7.22 (5H, m, ArH), 5.41 (1H, brs, H-13), 4.81 (1H, d, *J* = 7.1 Hz, H-3), 3.46 (2H, m, H-12), 3.14 - 2.86 (3H, m, H-11 & H-2), 1.89 (3H, s, H-15), 1.27 (3H, d, *J* = 6.8 Hz, H-10), 0.86 (9H, s, H-18), 0.01 (3H, s, H-16), -0.24 (3H, s, H-16’) ppm.

^{13}C-NMR (100 MHz, $CDCl_3$): δ = 202.1 (C-1), 170.0 (C-14), 142.9 (C-4), 128.0 (C-6/8), 127.5 (C-7), 126.9 (C-5/9), 76.6 (C-3), 57.4 (C-2), 39.7 (C-12), 28.7 (C-11), 25.7 (C-18), 23.16 (C-15), 18.1 (C-17), 13.5 (C-10), -4.6 (C-16), -5.2 (C-16’) ppm.

ESI-MS (*m/z*): 396.4 $[M + H]^+$, 418.4 $[M + Na]^+$.

HRMS (ESI): calculated for $C_{20}H_{34}NO_3SSi$ $[M + H]^+$: 396.2029; found: 396.2027

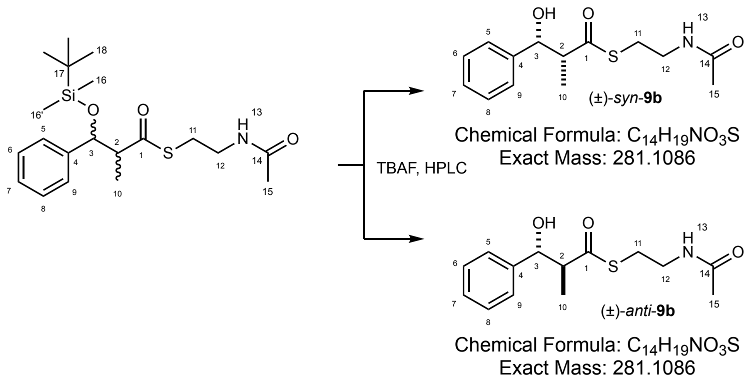

To the solution of 3-*O*-TBDMS protected SNACs as prepared above (1.0 eq., 128 mg, 0.32 mmol) in THF (0.3 ml), were added TBAF (2M in hexane, 4 eq., 0.64 ml, 1.28 mmol) and HOAc (4.4 eq., 0.08 ml, 1.41 mmol). The mixture was stirred at RT for up to 6 days (monitored by LCMS). After completion, the reaction was quenched with H_2O (6 mL) and extracted with ethyl acetate (3 × 7 mL). The organic layers were combined, washed with brine, dried over $MgSO_4$, filtered and concentrated *in vacuo* to give a light-yellow oil. The residue was purified by preparative LCMS using a Kinetex® C_{18} column (i.d., 250 × 21.2 mm, 20 mL/min) eluting with a gradient of CH_3CN in H_2O to yield (±)-*syn*-**9b**[13] (12.1 mg, 0.043 mmol, 17%) and (±)-*anti*-**9b** (17.5 mg, 0.062mmol, 25%) respectively as colourless oils/solid. In addition, 27.7 mg of starting material was recovered.

(±)-*syn*-**9b**

^{1}H-NMR (400 MHz, $CDCl_3$): δ = 7.37 - 7.26 (5H, m, ArH), 5.65 (1H, brs, H-13), 5.06 (1H, d, *J* = 4.9 Hz, H-3), 3.39 (1H, m, H-12), 3.29 (1H, m, H-12), 3.04 - 2.88 (3H, m, H11 & H-2), 1.92 (3H, s, H-15), 1.21 (3H, d, *J* = 7.0 Hz, H-10) ppm.

^{13}C-NMR (100 MHz, $CDCl_3$): δ = 203.4 (C-1), 170.4 (C-14), 141.3 (C-4), 128.3 (C-6/8), 127.7 (C-7), 126.2 (C-5/9), 74.3 (C-3), 55.5 (C-2), 39.1 (C-12), 28.7 (C-11), 23.2 (C-15), 11.7 (C-10) ppm.

ESI-MS (*m/z*): 282.4 $[M + H]^+$, 304.3 $[M + Na]^+$.

HRMS (ESI): calculated for $C_{14}H_{19}NO_3SNa$ $[M + Na]^+$: 304.0983; found: 304.0985

(±)-*anti*-**9b**

$UV_{\lambda max}$ (CH_3CN:H_2O): 210 nm

^{1}H-NMR (400 MHz, $CDCl_3$): δ = 7.38 - 7.28 (5H, m, ArH), 5.94 (1H, brs, H-12), 4.79 (1H, d, *J* = 8.6 Hz, H-3), 3.42 (2H, m, H-11), 3.12-2.96 (3H, m, H-2 & H-10), 1.93 (3H, s, H-14), 0.99 (3H, d, *J* = 7.1 Hz, H-15) ppm.

^{13}C-NMR (100 MHz, $CDCl_3$): δ = 203.6 (C-1), 170.4 (C-13), 141.5 (C-4), 128.6 (C-6/8), 128.2 (C-7), 126.6 (C-5/9), 76.7 (C-3), 55.7 (C-2), 39.3 (C-11), 28.8 (C-10), 23.2 (C-14), 15.3 (C-15) ppm.

ESI-MS (*m/z*): 282.4 $[M + H]^+$, 304.3 $[M + Na]^+$.

HRESIMS (*m/z*): calculated for $C_{14}H_{19}NO_3SNa$ $[M + Na]^+$: 304.0983; found: 304.0985

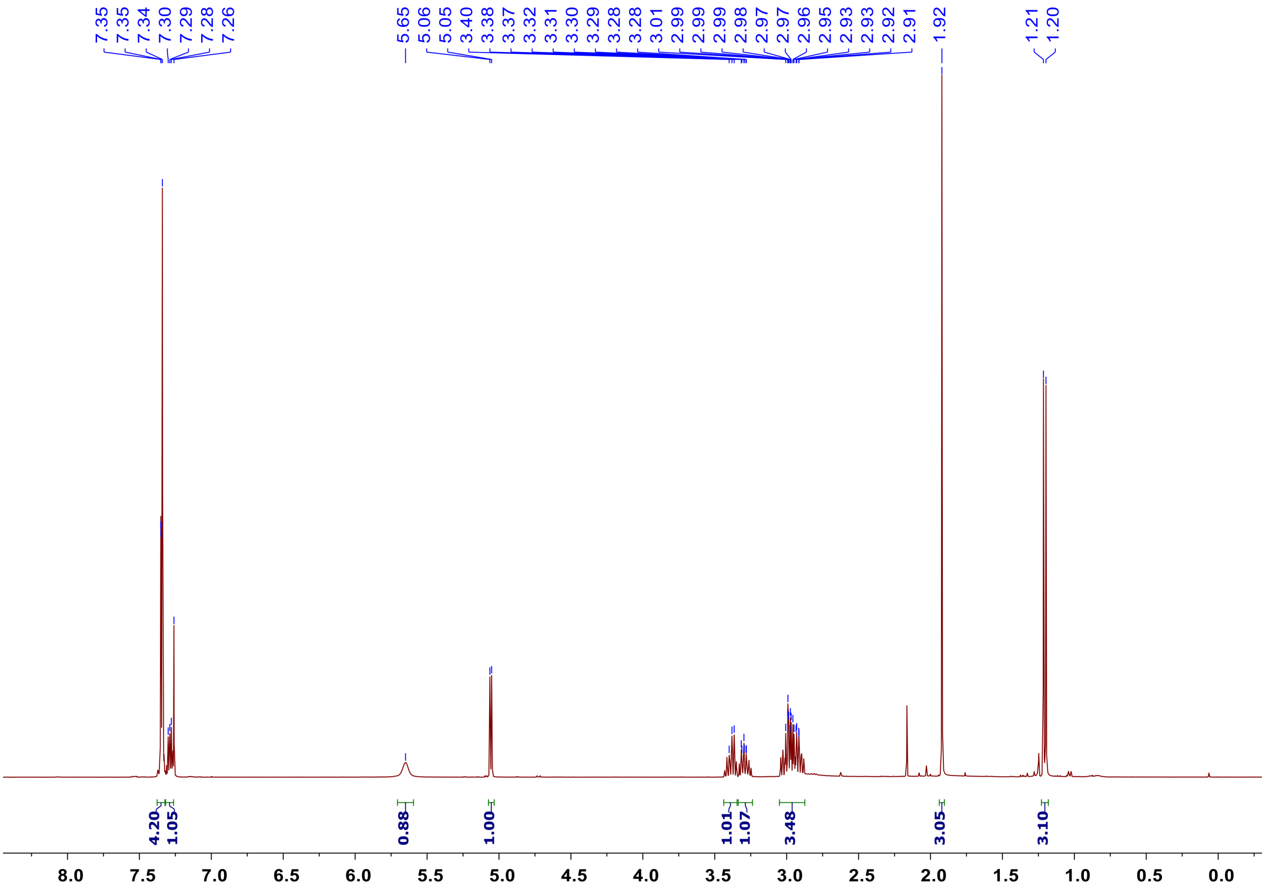

Figure S3.3.3E ^{1}H NMR of (±)-*syn*-**9b** in $CDCl_3$.

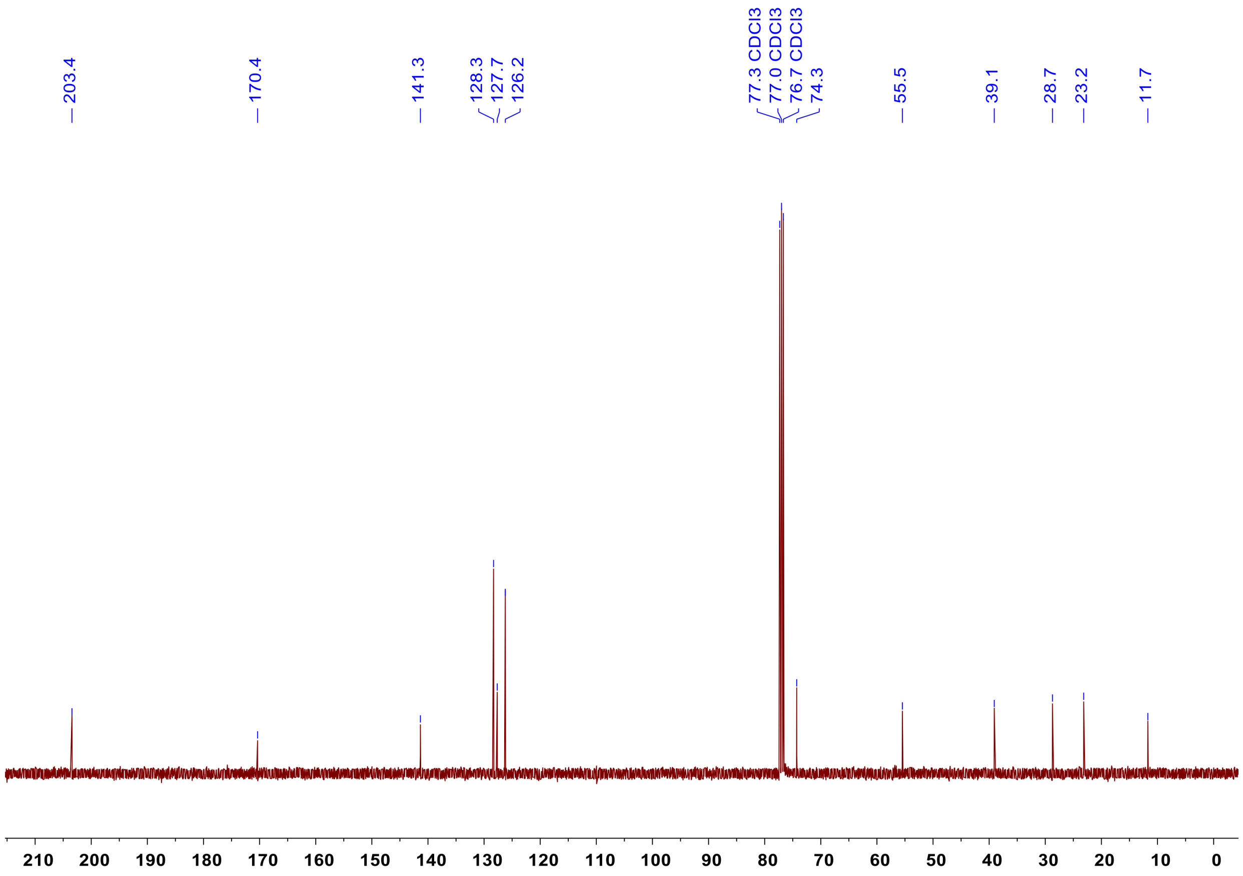

Figure S3.3.3F ^{13}C NMR of (±)-*syn*-**9b** in $CDCl_3$.

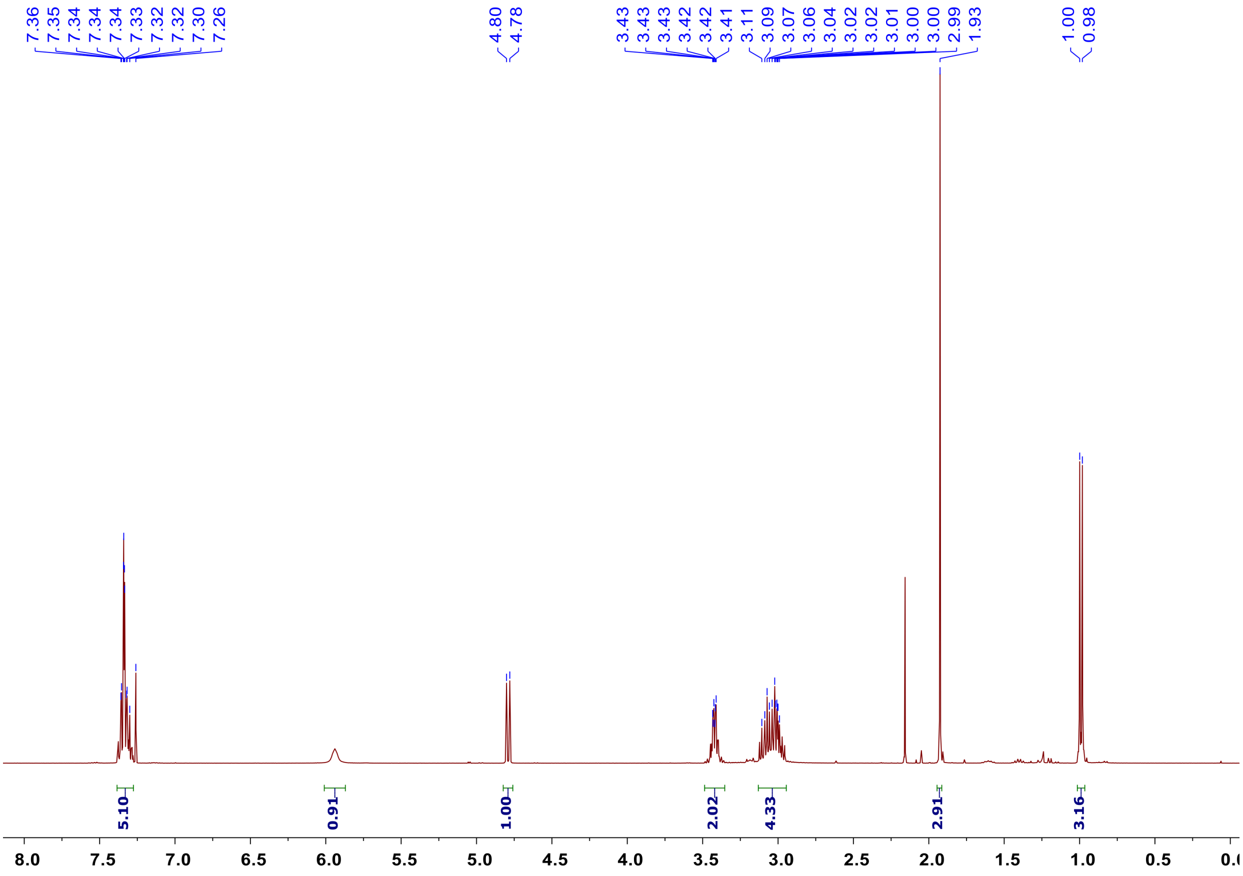

Figure S3.3.3G ^{1}H NMR of (±)-*anti*-**9b** in $CDCl_3$.

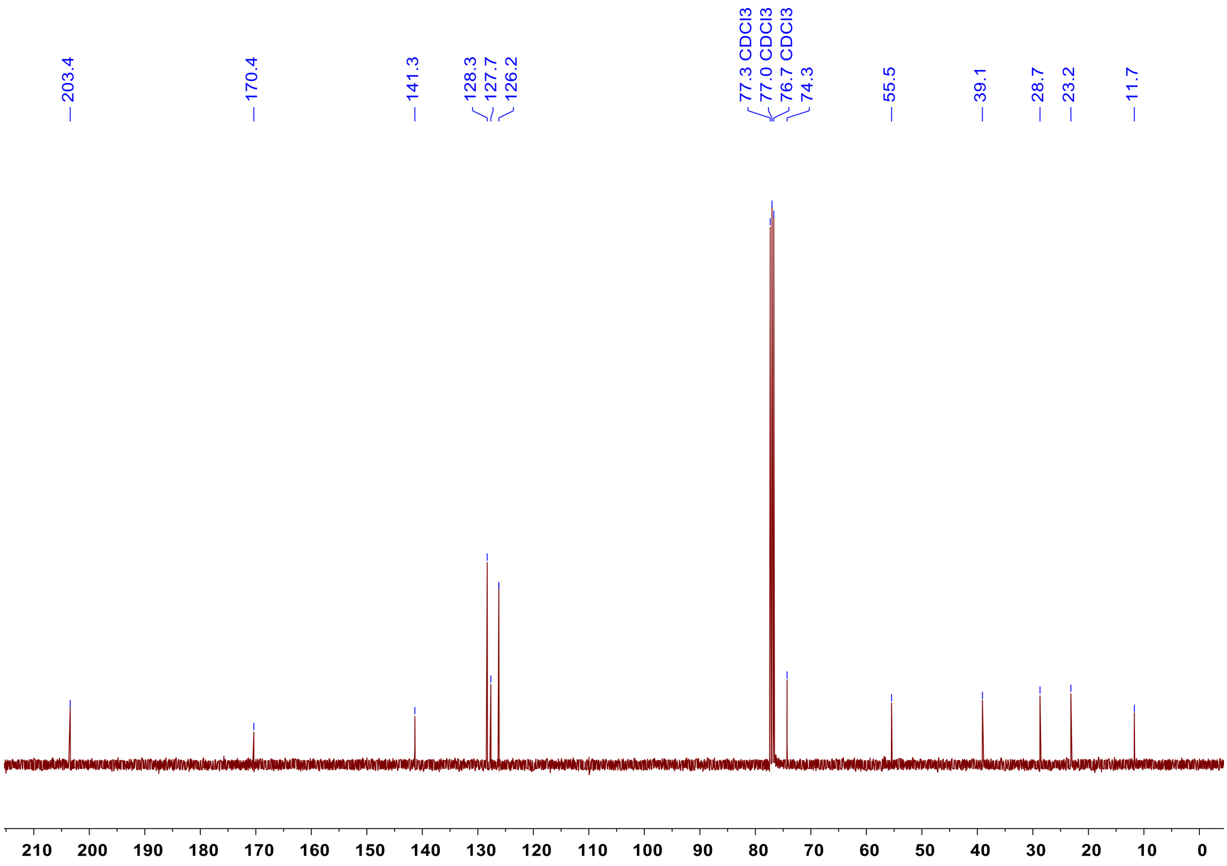

Figure S3.3.3H ^{13}C NMR of (±)-*anti*-**9b** in $CDCl_3$.

***R*-4-benzyl-3-((2*S*,3*R*)-3-hydroxy-2-methyl-3-phenylpropanoyl)oxazolidin-2-one L-33**[14]

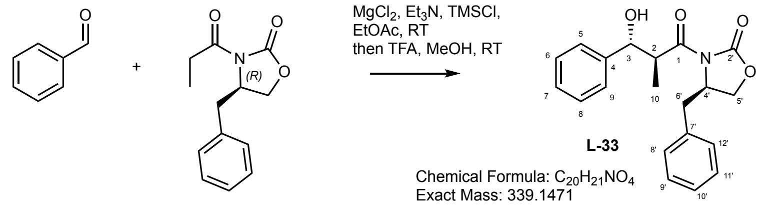

The procedure was slightly adjusted as described by Evans et al. for the enantiomer. *R*-Oxazolidinone (abcr, 1.0 eq., 200 mg, 0.86 mmol) was dissolved in EtOAc (2 mL), and $MgCl_2$ (0.1 eq., 8.18 mg, 0.086 mmol), Et_3N (2.0 eq., 0.24 mL, 1.72 mmol), benzaldehyde (1.2 eq., 0.11 mL, 1.03 mmol) and $(CH_3)_3SiCl$ (1.5 eq., 0.16 mL, 1.29 mmol) were added sequentially. The suspension was stirred for 22 h at RT. The reaction mixture was then filtered through a silica plug with 50 mL of Et_2O. The solution was concentrated *in vacuo* and redissolved in MeOH (10 mL). To the solution were added 2 drops of TFA and stirred vigorously. After 30 min the solution was concentrated *in vacuo* to give a crude yellow oil. The crude residue was purified by flash chromatography (acetone/petroleum ether 1:9) to give L-**33**[14] as a colourless oil (265 mg, 0.78 mmol, 91%).

$[\alpha]_D^{20}$ (c = 1.00, $CHCl_3$) = + 12.3 (lit. enantiomer $[\alpha]_D^{25}$ (c = 0.78, $CHCl_3$) = - 10.2)[14]

^{1}H-NMR (400 MHz, $CDCl_3$): δ = 7.47 - 7.37 (m, 2H), 7.37 - 7.31 (m, 2H), 7.31 - 7.21 (m, 4H), 7.18 - 7.10 (m, 2H), 4.80 (d, *J* = 8.1 Hz, 1H), 4.72 - 4.58 (m, 1H), 4.40 - 4.26 (m, 1H), 4.20 - 4.03 (m, 2H), 3.17 (dd, *J* = 13.6, 3.4 Hz, 1H), 3.13 (brs, 1H), 2.64 (dd, *J* = 13.6, 9.3 Hz, 1H), 1.07 (d, *J* = 6.9 Hz, 3H) ppm.

^{13}C-NMR (100 MHz, $CDCl_3$): δ = 176.5 (C-1), 153.4 (C-2'), 142.0 (C-4), 135.1 (C-7'), 129.3 (C-6/8), 128.8 (C-9'/11'), 128.4 (C-5/9), 127.8 (C-7), 127.1 (C-8'/12'), 126.5 (C-10'), 77.2 (C-3), 65.8 (C-5'), 55.2 (C-4'), 44.2 (C-2), 37.4 (C-6'), 14.7 (C-10) ppm.

***S*-(2-Acetamidoethyl) (2*S*,3*R*)-3-hydroxy-2-methyl-3-phenylpropanethioate L-*anti*-9b**
***and S*-(2-Acetamidoethyl) (2*R*,3*S*)-3-hydroxy-2-methyl-3-phenylpropanethioate D-*anti*-9b**

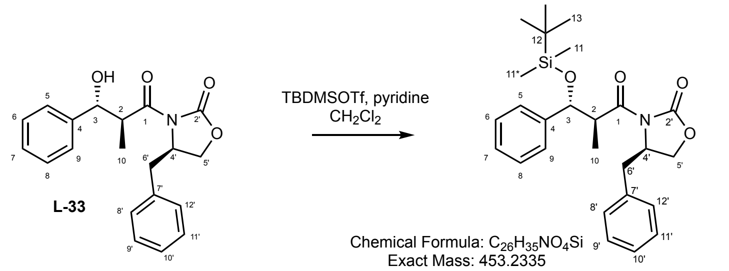

The Evans aldol product **33** (1.0 eq., 140 mg, 0.41 mmol) was dissolved in CH_2Cl_2 (3 mL). To the solution pyridine (2.0 eq., 0.07 mL, 0.82 mmol) was added and allowed to stir for 10 min. Then the reaction mixture was cooled to 0 °C and TBDMSOTf (1.2 eq., 0.11 mL, 0.49 mmol) was added dropwise. After complete addition the reaction mixture was allowed to warm to rt until completion. The mixture was quenched with the addition of a sat. aqueous $NaHCO_3$ (2 mL). The phases were separated, and the aqueous phase was extracted with CH_2Cl_2 (3 × 5 mL). The combined organic layers were dried over Na_2SO_4, filtered and concentrated in *vacuo*. The crude oily residue was purified by flash chromatography (petroleum ether/ethyl acetate 3:1) to give a clear oil/opaque wax (154 mg, 0.34 mmol, 83%).

^{1}H-NMR (400 MHz, $CDCl_3$): δ = 7.39-7.27 (10H, m, ArH), 4.94 (1H, d, *J* = 9.3 Hz, H-3), 4.70 (1H, ddt, *J* = 10.5, 7.0, 3.5 Hz, H-4'), 4.25 (1H, dq, *J* = 9.3, 7.0 Hz, H-2), 4.14 (2H, m, H-5'), 3.47 (1H, dd, *J* = 13.3, 3.3 Hz, H-6'), 2.70 (1H, dd, *J* = 13.3 Hz, 10.5 Hz, H-6'), 0.85 (3H, d, *J* = 7.0 Hz, H-10), 0.79 (9H, s, H-13), -0.01 (3H, s, H-11), -0.29 (3H, s, H-11') ppm;
^{13}C-NMR (100 MHz, $CDCl_3$): δ = 176.1 (C-1), 153.3 (C-2'), 142.5 (C-4), 135.8 (C-7'), 129.6 (C-6/8), 129.2 (C-9'/11'), 128.3 (C-5/9), 128.0 (C-7), 127.8 (C-8'/12'), 127.5 (C-10'), 77.8 (C-3), 66.0 (C-5'), 55.7 (C-4'), 46.5 (C-2), 38.6 (C-6'), 25.9 (C-13), 24.0 (C-12), 14.8 (C-10), -4.5 (C-11), -4.8 (C-11') ppm.
ESI-MS (*m/z*): 476.3 $[M + Na]^+$.

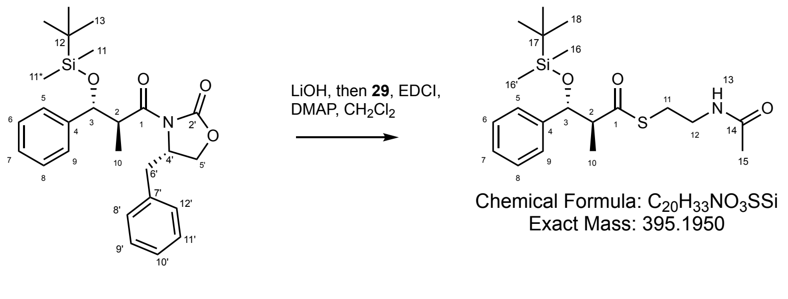

The 3-*O*-TBDMS protected aldol product as prepared above (1.0 eq., 150 mg, 0.33 mmol) was dissolved in THF (6 mL) and cooled to 0 °C. The solution was then treated with H_2O_2 (30% in H_2O, 7 eq., 0.24 mL, 2.32 mmol) and $LiOH{\cdot}H_2O$ (2.5 eq., 35 mg, 0.83 mmol) dissolved in H_2O (2 mL). The solution was slowly allowed to reach RT and stirred for 4 h. The reaction mixture was carefully quenched with sat. aqueous $Na_2S_2O_3$ (3 mL) under constant stirring. After additional 10 min stirring, the phases were separated, and the aqueous phase was acidified with HCl (2 M) and extracted with EtOAc (3 × 8 mL). The combined organic phases were dried over Na_2SO_4, filtered and concentrated *in vacuo*. The residue was then purified by flash chromatography (ethyl acetate/petroleum ether 1:10 + 1% formic acid) to give the desired acid as a white solid that was dried under high vacuum and immediately subjected to the next step. The residue was dissolved in CH_2Cl_2 (1.5 mL) and cooled to 0 °C. Then DMAP (0.25 eq., 5 mg, 0.04 mmol) and EDCl (1.1 eq., 36 mg, 0.19 mmol) were added and the reaction mixture was allowed to stir for 5 min, prior the final addition of HSNAC **29** (1.1 eq., 23 mg, 0.19 mmol). After complete addition the reaction mixture was allowed to warm to room temperature and stirred overnight. The reaction mixture was then quenched by the addition of H_2O (2 mL) and extracted with CH_2Cl_2 (3 × 3 mL). The combined organic phases were dried over $MgSO_4$, filtered and concentrated in *vacuo*. The crude residue was then purified by flash chromatography (ethyl acetate / petroleum ether 1:1) to give a colourless oil (88 mg, 0.22 mmol, 67%).

$[\alpha]_D^{20}$ (c = 1.0 in $CHCl_3$) = + 97.5
^{1}H-NMR (400 MHz, $CDCl_3$): δ = 7.41 - 7.22 (5H, m, ArH), 4.77 (1H, d, *J* = 9.1 Hz, H-3), 3.49 (2H, m, H-12), 3.13 (1H, m, H-11), 2.99 (2H, m, H-2 & H-11), 2.03 (3H, s, H-15), 0.87 (3H, d, *J* = 7.1 Hz, H-10), 0.82 (9H, s, H-18), -0.02 (3H, s, H-16), -0.30 (3H, s, H-16') ppm.
^{13}C-NMR (100 MHz, $CDCl_3$): δ = 203.3 (C-1), 170.7 (C-14), 142.1 (C-4), 128.2 (C-6/8), 127.9 (C-7), 127.0 (C-5/9), 77.5 (C-3), 57.4 (C-2), 39.9 (C-12), 28.5 (C-11), 25.6 (C-18), 23.1 (C-15), 18.0 (C-17), 14.8 (C-10), -4.7 (C-16), -5.5 (C-16') ppm.
ESI-MS (*m/z*): 396.4 $[M + H]^+$, 418.3 $[M + Na]^+$.
HRESIMS (*m/z*): calculated for $C_{20}H_{33}NO_3SSiNa$ $[M + Na]^+$: 418.1843 found 418.1854.

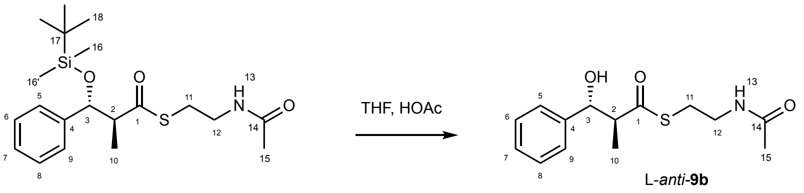

Chemical Formula: $C_{20}H_{33}NO_3SSi$
Exact Mass: 395.1950

Chemical Formula: $C_{14}H_{19}NO_3S$
Exact Mass: 281.1086

The 3-*O*-TBDMS protected intermediate as prepared above (1.0 eq., 10 mg, 0.025 mmol.) was dissolved in THF (400 µL) and glacial acetic acid (200 µL) was added to the mixture. The reaction mixture was then sealed and heated to 40 °C overnight. After cooling it to room temperature the reaction mixture was diluted with the addition of H_2O (1 mL). The aqueous phase was extracted with CH_2Cl_2 (2 × 2 mL). The combined organic phases were dried over $MgSO_4$, filtered and dried in *vacuo*. The crude residue was then purified by flash chromatography (ethyl acetate/ petroleum ether 1:1 to pure ethyl acetate) to give L-*anti*-**9b** as a colourless oil (5 mg, 0.018 mmol, 70%).

$[\alpha]_D^{20}$ (c = 0.33 in $CHCl_3$) = + 73.6

^{1}H-NMR (400 MHz, $CDCl_3$): δ = 7.39 - 7.30 (5H, m, ArH), 6.06 (1H, brs, H-12), 4.80 (1H, d, *J* = 8.6 Hz, H-3), 3.48 - 3.43 (2H, m, H-12), 3.15 - 2.98 (3H, m, H-2 & H-11), 1.98 (3 H, s, H-15), 1.01 (3H, d, *J* = 7.1 Hz, H-10) ppm.

^{13}C-NMR (100 MHz, $CDCl_3$): δ = 203.7 (C-1), 171.9 (C-13), 141.6 (C-4), 128.8 (C-6/8), 128.4 (C-7), 126.8 (C-5/9), 76.9 (C-3), 55.9 (C-2), 39.9 (C-12), 28.5 (C-11), 22.7 (C-15), 15.4 (C-10) ppm.

ESI-MS (*m/z*): 282.4 $[M + H]^+$, 304.3 $[M + Na]^+$.

HRESIMS (*m/z*): calculated for $C_{14}H_{19}NO_3SNa$ $[M + Na]^+$: 304.0983 found 304.0981.

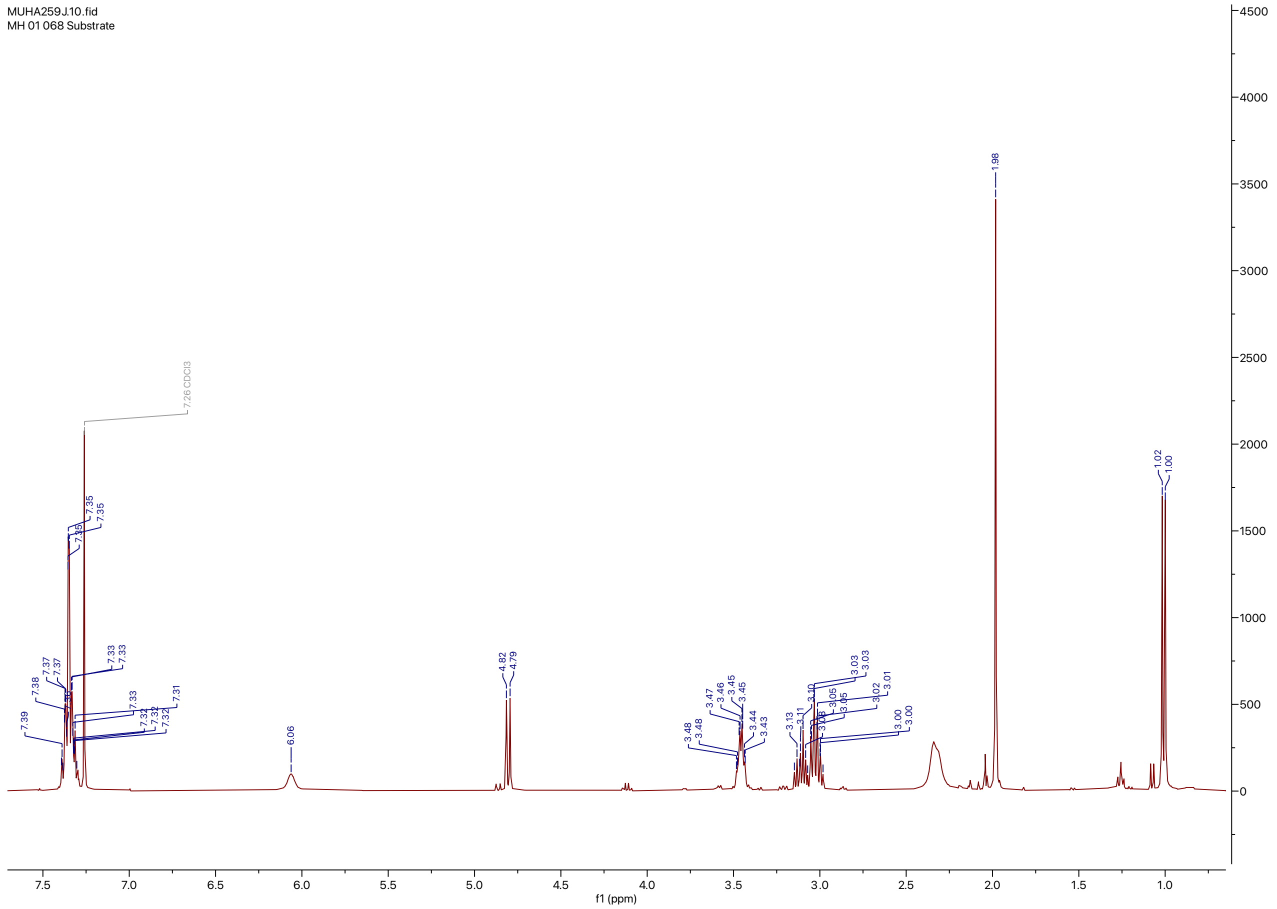

Figure S3.3.3l ^{1}H NMR of L-*anti*-**9b** in $CDCl_3$.

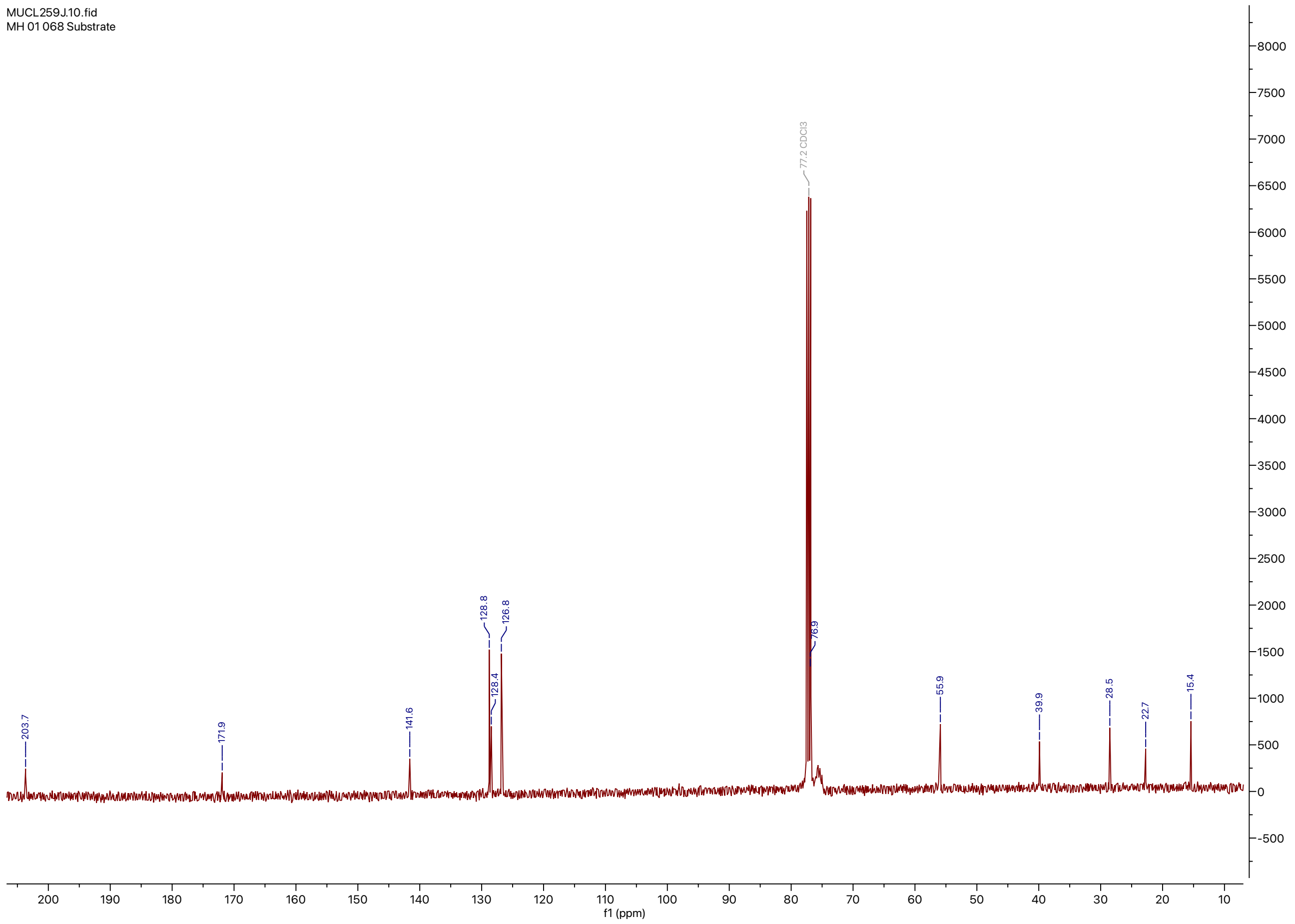

Figure S3.3.3J ^{13}C NMR of L-*anti*-**9b** in $CDCl_3$.

D-*anti*-9b

Same procedure as described in L-*anti*-**9b** starting from *S*-Oxazolidinone.

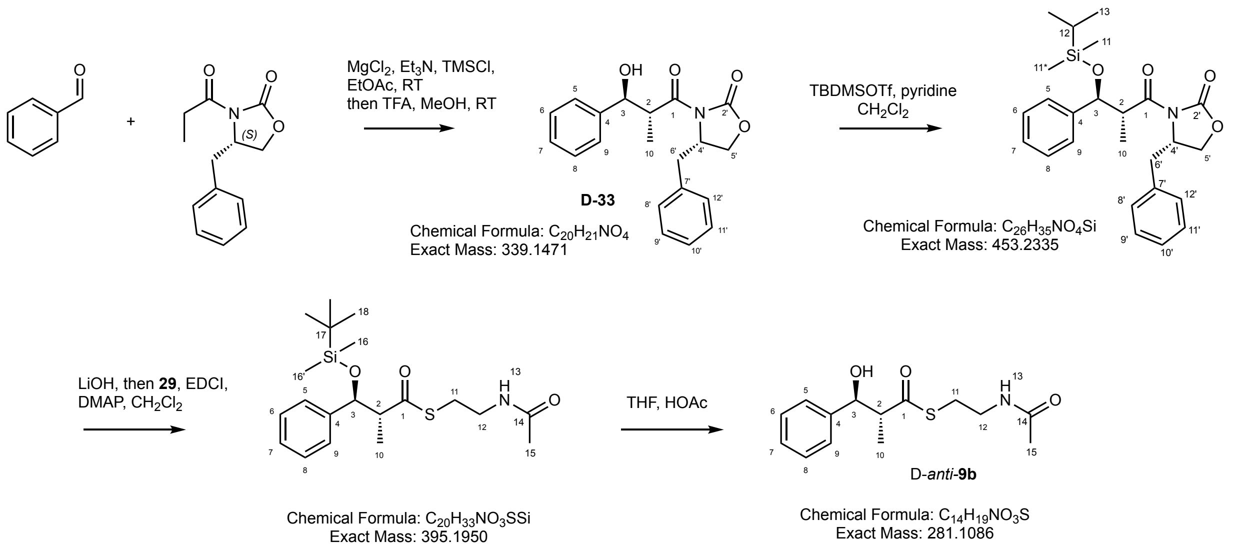

$[\alpha]_D^{20}$ (c 0.22 in $CHCl_3$) = - 68.3

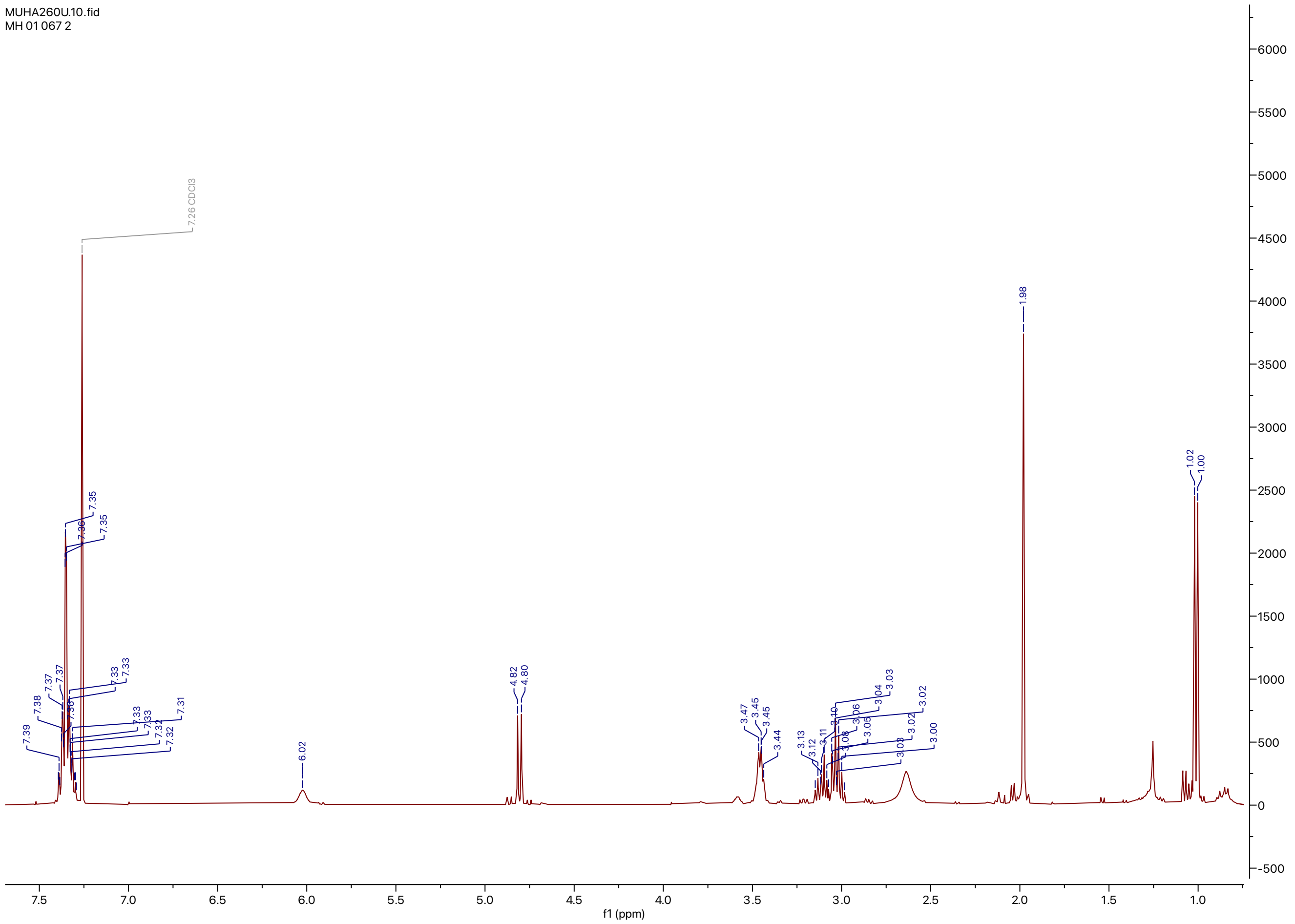

Figure S3.3.3K ^{1}H NMR of D-*anti*-**9b** in $CDCl_3$.

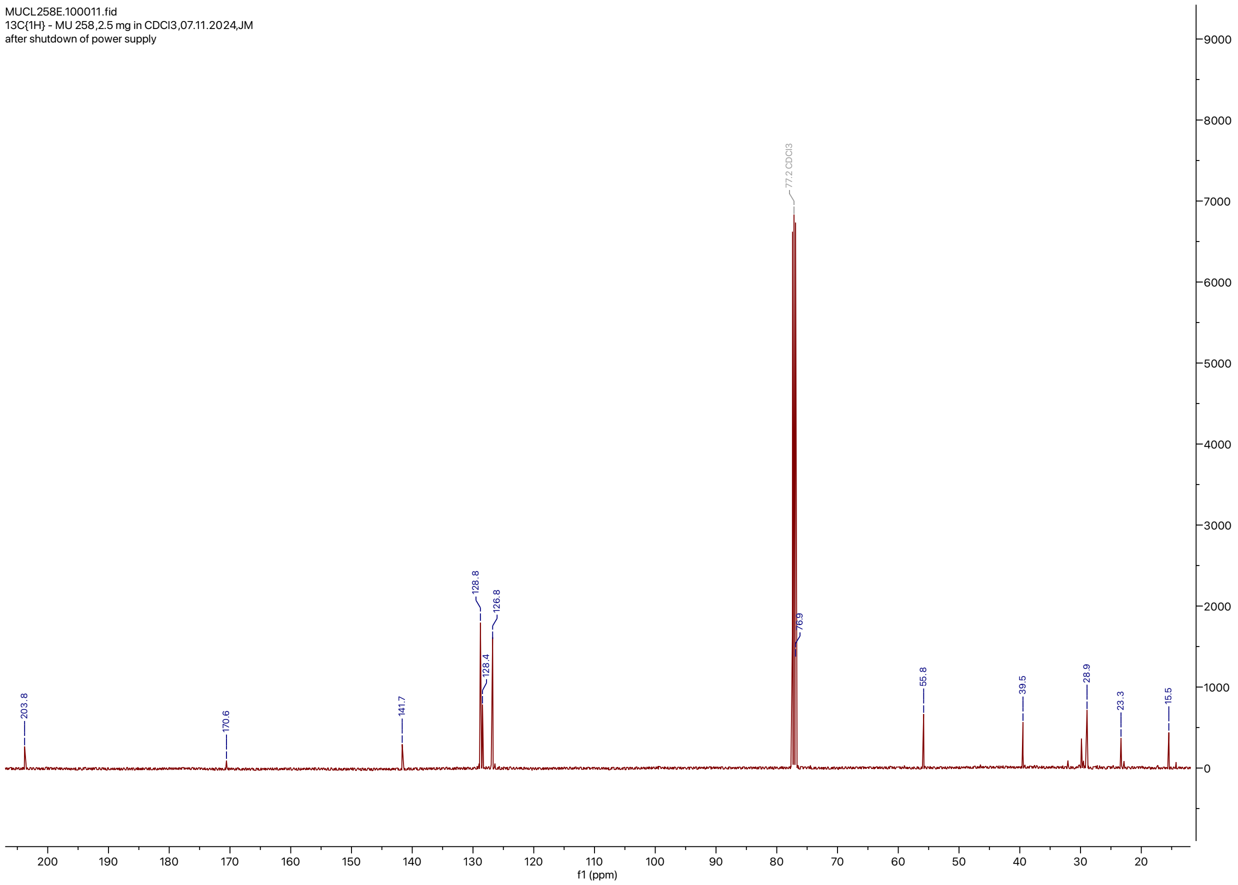

Figure S3.3.3L ^{13}C NMR of D-*anti*-**9b** in $CDCl_3$.

3.3.4 Compounds 10

Methyl *Z*-3-phenylacrylate *Z*-36a[15]

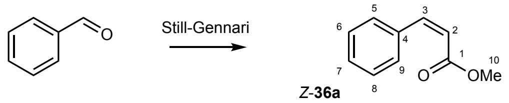

Methyl bis(2,2,2-trifluoroethoxy)phosphinyl acetate (Sigma-Aldrich, 1.4 eq., 0.14 mL, 0.66 mmol) was dissolved in THF (2 mL) and the solution was cooled to -78 °C. To the cooled solution was added NaHMDS (2 M in THF, 1.4 eq., 0.33 mL, 0.66 mmol) dropwise. After complete addition the solution was stirred for 5 min before benzaldehyde (1 eq., 0.05 mL, 0.45 mmol) was added. The reaction mixture was stirred for 1h at -78 °C before it was quenched with sat. NH_4Cl and allowed to warm to room temperature. The phases were separated, and the aqueous phase was extracted with EtOAc (2 × 3 mL), dried over $MgSO_4$, filtered and concentrated in *vacuo*. The crude residue was purified by flash chromatography (petroleum ether : ethyl acetate 20:1) to give the desired alkene **36a**[15] (45 mg, 0.27 mmol, 60%) in a diastereomeric ratio of 4:1 (*Z:E*) based on ^{1}H NMR.

^{1}H-NMR (400 MHz, $CDCl_3$): δ = 7.59 (2H, m, ArH), 7.37 (3H, m, ArH), 6.96 (1H, d, *J* = 12.6 Hz, H-3), 5.96 (1H, d, *J* = 12.6 Hz, H-2), 3.72 (3H, s, H-10) ppm.
^{13}C-NMR (100 MHz, $CDCl_3$): δ = 166.7 (C-1), 143.5 (C-3), 134.9 (C-4), 129.8 (C-6/8), 129.2 (C-7), 128.2 (C-5/9), 119.4 (C-2), 51.5 (C-10) ppm.
ESI-MS (*m/z*): 163.1 $[M + H]^+$, 185.1 $[M + Na]^+$.

S-(2-Acetamidoethyl) *Z*-3-phenylprop-2-enethioate *Z*-10a

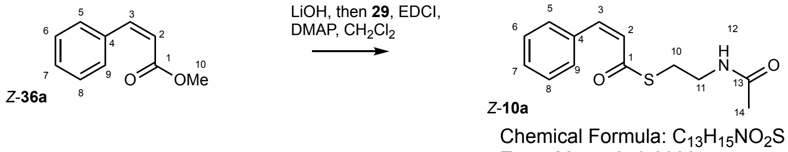

Methyl *Z*-3-phenylacrylate **36a** (1.0 eq., 20 mg, 0.12 mmol) was dissolved in THF (0.5 mL) and to this solution was added $LiOH·H_2O$ (4.0 eq., 20 mg, 0.48 mmol) dissolved in H_2O (0.5 mL). The reaction mixture was heated to 40 °C and vigorously stirred until completion. The reaction mixture was allowed to cool before it was diluted with water (2 mL). The aqueous phase was acidified with aq. HCl (2M) and extracted with CH_2Cl_2 (3 × 2 mL), dried over $MgSO_4$, filtered and concentrated *in vacuo*. The crude solid was dissolved in CH_2Cl_2 (1 mL) and cooled to 0 °C. After cooling EDCl (2.5 eq, 57 mg, 0.3 mmol) and DMAP (0.2 eq., 3 mg, 0.02 mmol) were added. The solution was allowed to stir for 5 min before HSNAC **29** (1.0 eq., 14 mg, 0.12 mmol) was added. After complete addition the reaction mixture was allowed to warm to room temperature and stirred for 4h. After completion the reaction was quenched by the addition of H_2O (3 mL). The phases were separated, and the aqueous phase was extracted with CH_2Cl_2 (2 × 2 mL). The combined organic phases were dried over $MgSO_4$, filtered and concentrated in *vacuo*. The crude residue was purified by flash chromatography (petroleum ether : ethyl acetate 3:1 to 1:1) to give the desired alkene (14 mg, 0.056 mmol, 46%) in a diastereomeric ratio of 1:2.2 (*Z:E*) based on ^{1}H NMR.

UV$_{λmax}$ ($CH_3CN:H_2O$): 213 nm 292 nm

^{1}H-NMR (400 MHz, $CDCl_3$): δ = 7.56 - 7.54 (2H, m, ArH), 7.42 - 7.39 (2H, m, ArH), 7.37 - 7.36 (1H, m, H-7), 6.78 (1H, d, *J* = 12.4 Hz, H-2), 6.21 (1H, d, *J* = 12.4 Hz, H-3)), 3.46 (2H, dt, *J* = 13.5, 6.4 Hz, H-11), 3.09 (2H, t, *J* = 6.4 Hz, H-10), 1.98 (3H, s, H-14).

^{13}C-NMR (100 MHz, $CDCl_3$): δ = 190.4 (C-1), 170.7 (C-13), 141.2 (C-3), 134.7 (C-4), 130.1 (C-6/8), 129.9 (C-7), 128.3 (C-5/9), 125.9 (C-2), 39.9 (C-11), 29.1 (C-10), 28.9 (C-14) ppm.

ESI-MS (*m/z*): 250.1 $[M + H]^+$, 272.1 $[M + Na]^+$.

HRESIMS (*m/z*): calculated for $C_{13}H_{15}NO_2SNa$ $[M + Na]^+$: 272.0716 found 272.0725

a

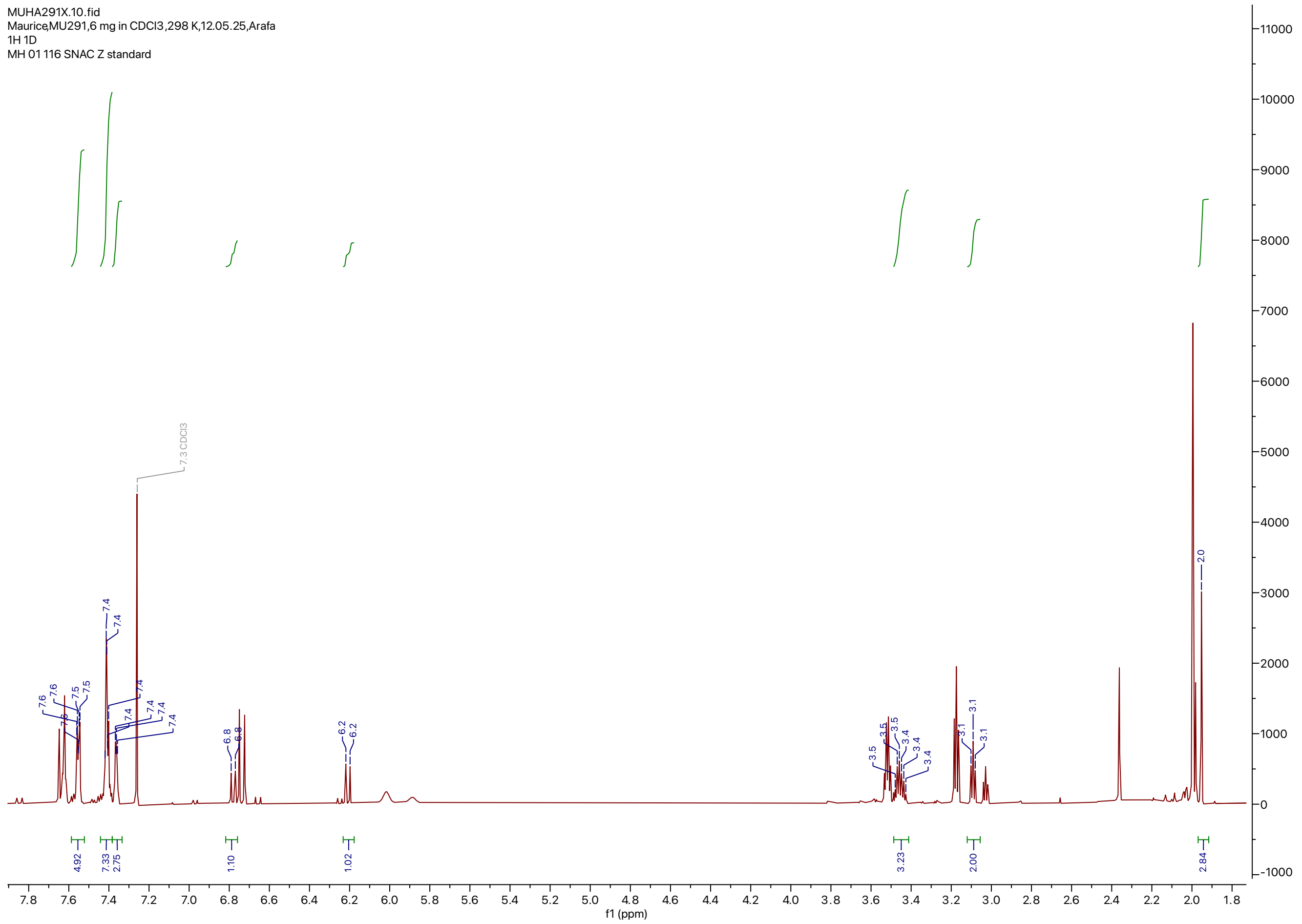

Figure S3.3.4A ^{1}H NMR of *Z*-**10a** in $CDCl_3$. Approximately 1:2.2 *Z*:*E* based in integrals.

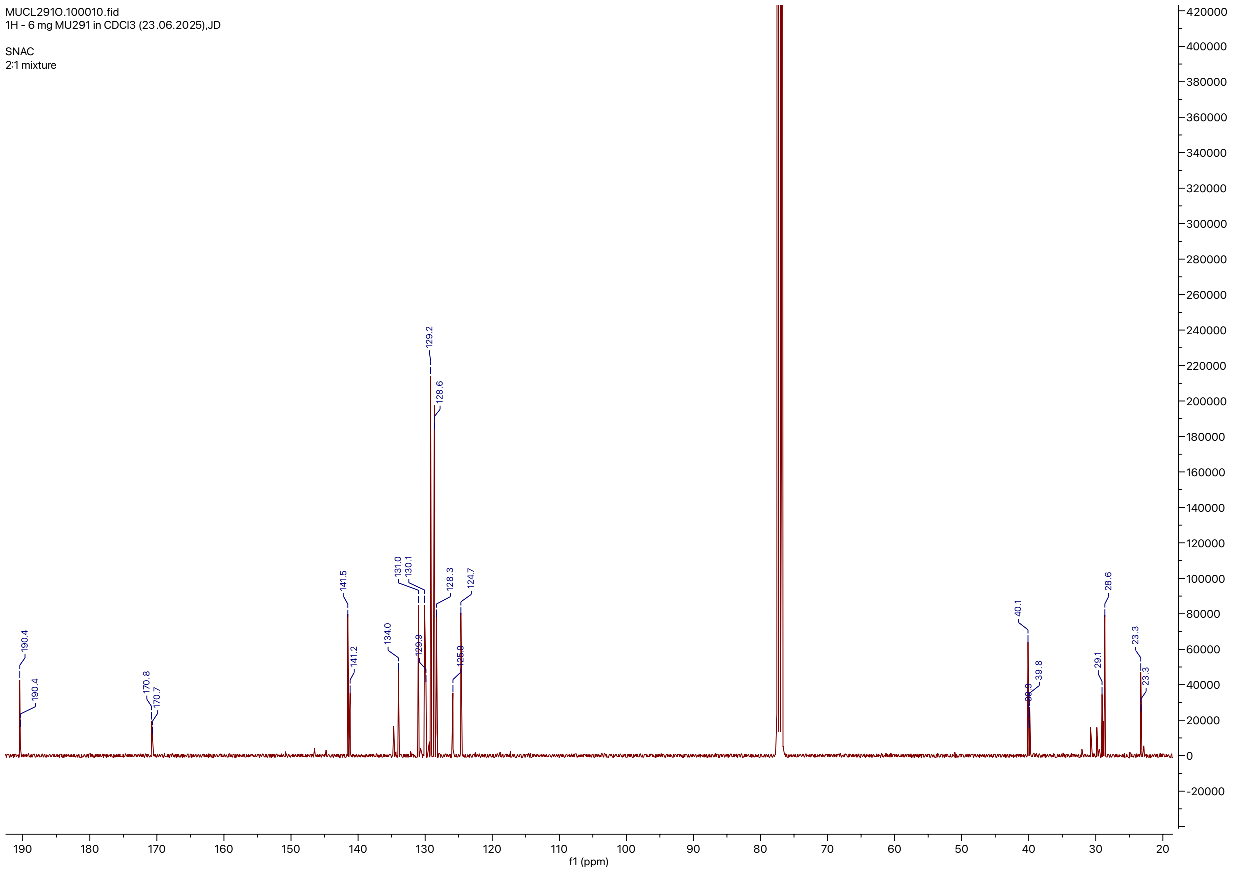

Figure S3.3.4B ^{13}C NMR of *Z*-**10a** in $CDCl_3$.

S-(2-Acetamidoethyl) *E*-3-phenylprop-2-enethioate *E*-10a[10]

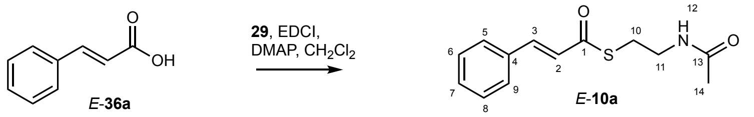

Chemical Formula: $C_{13}H_{15}NO_2S$
Exact Mass: 249.0823

Trans-Cinnamic acid *E*-**36a** (TCI, 1.0 eq., 100 mg, 0.67 mmol) was dissolved in CH_2Cl_2 (2 mL) and cooled to 0 °C. After cooling EDCl (2.5 eq, 322 mg, 1.68 mmol) and DMAP (0.2 eq., 16 mg, 0.13 mmol) were added. The solution was allowed to stir for 10 min before HSNAC **29** (1.1 eq., 88 mg, 0.74 mmol) was added. After complete addition the reaction mixture was allowed to warm to room temperature and stirred for overnight. After completion the reaction was quenched by the addition of H_2O (4 mL). The phases were separated, and the aqueous phase was extracted with CH_2Cl_2 (2 × 4 mL). The combined organic phases were dried over $MgSO_4$, filtered and concentrated in *vacuo*. The crude residue was purified by flash chromatography (petroleum ether : ethyl acetate 3:1 to 1:1) to give the desired alkene *E*-**10a**[10] (68 mg, 0.27 mmol, 40%) as a colourless crystalline solid.

UV$_{\lambda max}$ ($CH_3CN:H_2O$): 217 nm 303 nm
^{1}H-NMR (400 MHz, $CDCl_3$): δ = 7.63 (1H, d, *J* = 15.8 Hz, H-3), 7.56 (2H, m, ArH), 7.41 (3H, m, ArH), 6.74 (1H, d, *J* = 15.8 Hz, H-2), 5.92 (1H, brs, H-12), 3.51 (2H, m, H-11), 3.17 (2H, m, H-10), 1.98 (3H, s, H-14) ppm.
^{13}C-NMR (100 MHz, $CDCl_3$): δ = 190.4 (C-1), 170.5 (C-13), 141.5 (C-3), 134.0 (C-4), 131.0 (C-7), 129.2 (C-5/9), 128.6 (C-6/8), 124.7 (C-2), 40.0 (C-11), 28.7 (C-10), 23.4 (C-14) ppm.
ESI-MS (*m/z*): 250.2 $[M + H]^+$, 272.1 $[M + Na]^+$.

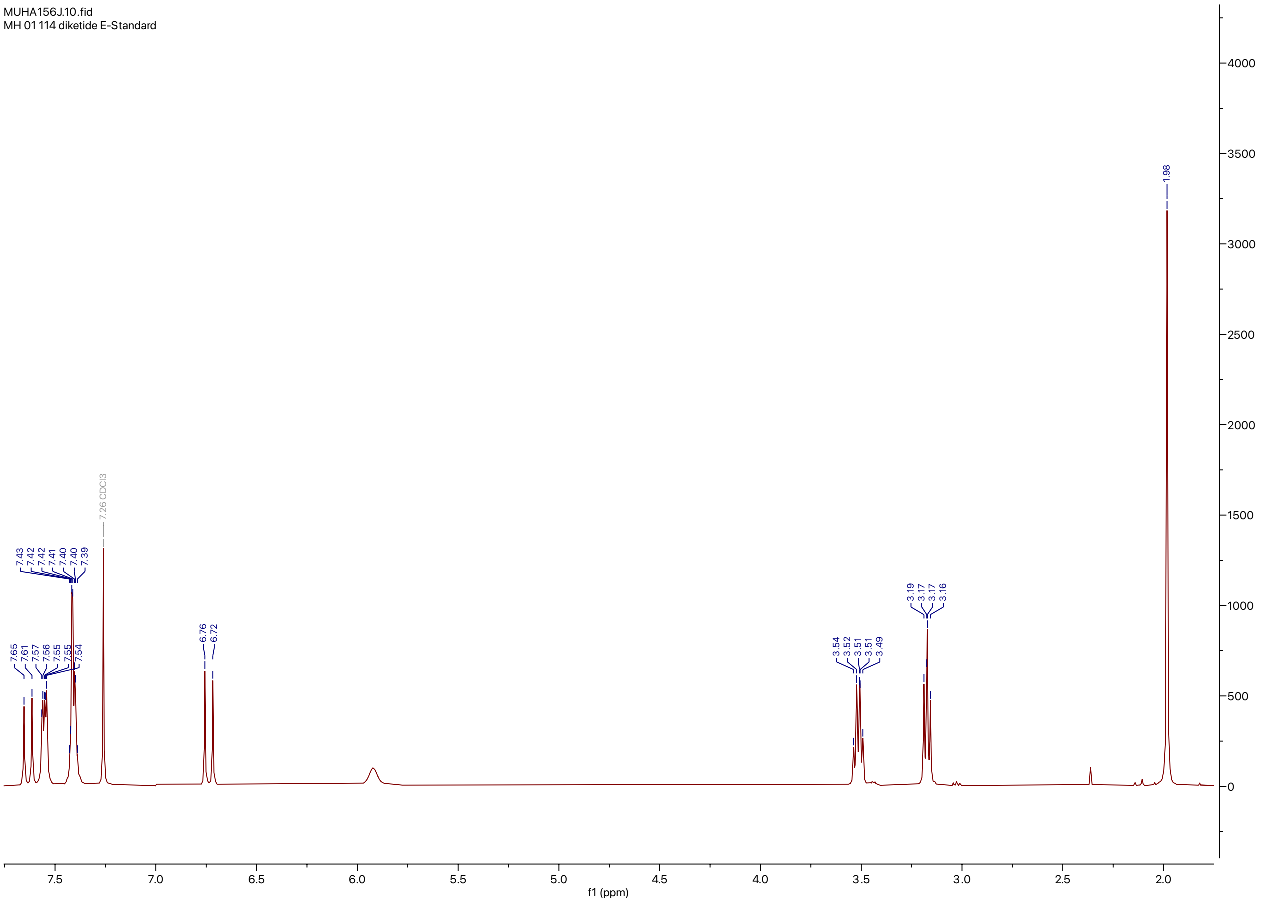

Figure S3.3.4C ^{1}H NMR of *E*-**10a** in $CDCl_3$.

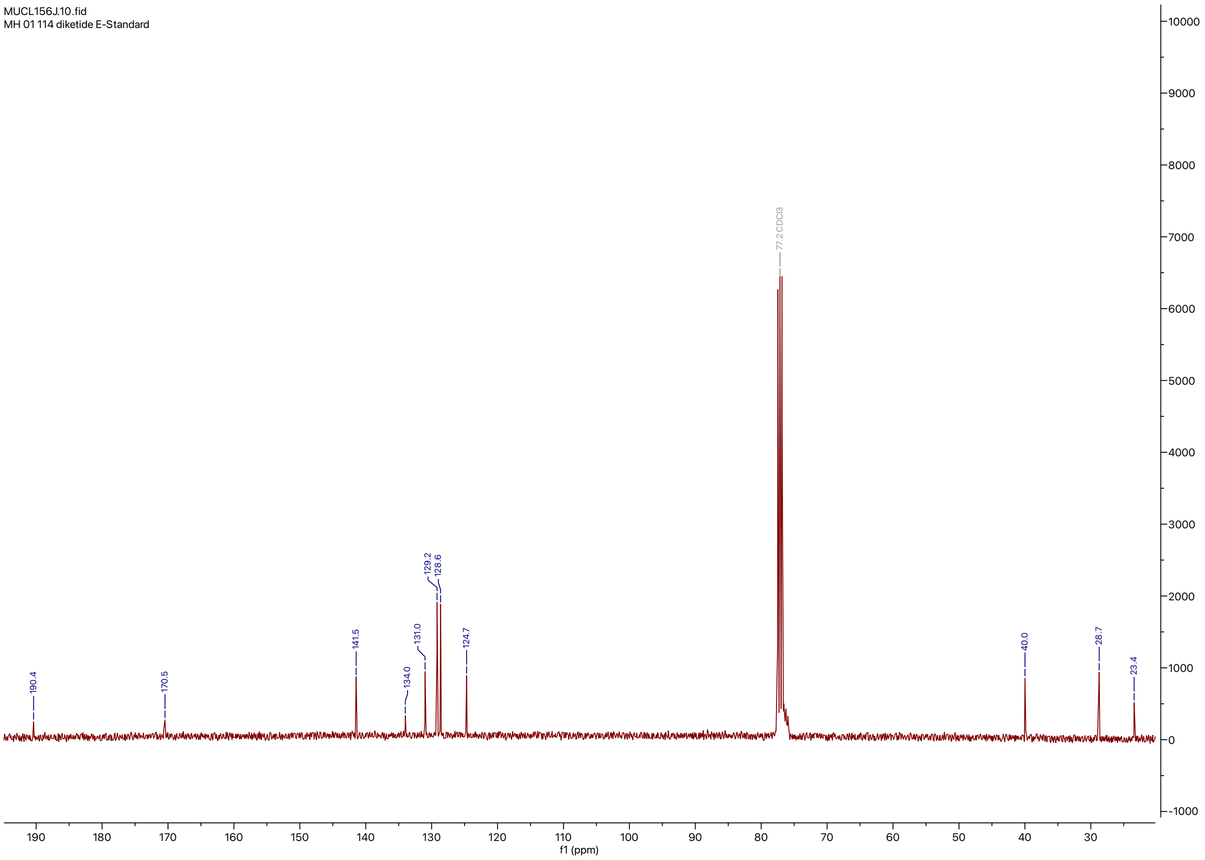

Figure S3.3.4D ^{13}C NMR of *E*-**10a** in $CDCl_3$.

Ethyl *Z*-2-methyl-3-phenylacrylate *Z*-36b[16]

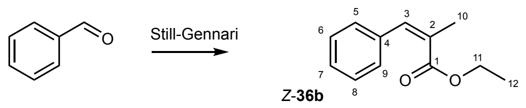

Ethyl-bis(2,2,2-trifluoroethoxy)phosphinyl propionate (Sigma-Aldrich, 1.0 eq., 50 mg, 0.144 mmol) was dissolved in THF (2 mL) and the solution was cooled to -78 °C. To the cooled solution was added NaHMDS (2 M in THF, 1.1 eq., 0.08 mL, 0.16 mmol) dropwise. After complete addition the solution was stirred for 15 min before benzaldehyde (1.0 eq., 0.015 mL, 0.144 mmol) was added. The reaction mixture was stirred for 1h at -78 °C before it was quenched carefully with sat. NH_4Cl and allowed to warm to room temperature. The phases were separated, and the aqueous phase was extracted with petroleum ether (2 × 2 mL), dried over $MgSO_4$, filtered and concentrated in *vacuo*. The crude residue was purified by flash chromatography (petroleum ether : ethyl acetate 20:1) to give the desired alkene **Z-36b**[16] (20 mg, 0.11 mmol, 73%) as a pure diastereomer based on ^{1}H NMR.

UV$_{\lambda max}$ (CH_3CN:H_2O): 217, 303 nm
^{1}H-NMR (400 MHz, $CDCl_3$): δ = 7.31-7.22 (5H, m, ArH), 6.71 (1H, d, *J* = 1.6 Hz, H-3), 4.11 (2H, q, *J* = 7.2 Hz, H-10), 2.10 (3H, d, *J* = 1.6 Hz, H-12), 1.10 (3H, t, *J* = 7.2 Hz, H-11) ppm.
^{13}C-NMR (100 MHz, $CDCl_3$): δ = 169.7 (C-1), 136.4 (C-3), 134.4 (C-4), 130.2 (C-2), 128.2 (C-5/9), 128.1 (C-6/8), 127.7 (C-7), 60.7 (C-10), 21.6 (C-12), 13.9 (C-11) ppm.
ESI-MS (*m/z*): 191.2 $[M + H]^+$, 213.2 $[M + Na]^+$.

S-(2-Acetamidoethyl) *Z*-2-methyl-3-phenylprop-2-enethioate *Z*-10b

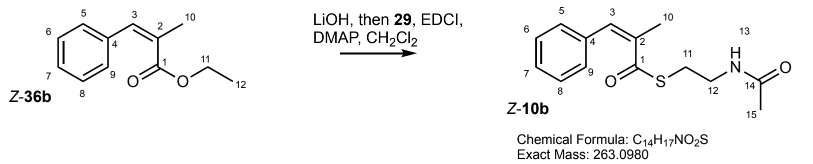

Ester *Z*-**36b** (1.0 eq., 20 mg, 0.11 mmol) was dissolved in THF : MeOH (1:1.2 mL) and LiOH·H_2O (3.0 eq., 13.2 mg, 0.32 mmol) dissolved in H_2O (0.5 mL) was added. The reaction vial was closed and heated to 40 °C. After completion the reaction mixture was allowed to cool before it was diluted with water (2 mL). The aqueous phase was acidified with aq. HCl (2M) and extracted with CH_2Cl_2 (3 × 2 mL), dried over $MgSO_4$, filtered and concentrated in *vacuo*. The crude solid was dissolved in CH_2Cl_2 (1 mL) and cooled to 0 °C. After cooling EDCl (1.4 eq, 23 mg, 0.15 mmol) and DMAP (0.2 eq., 3 mg, 0.01 mmol) were added. The solution was allowed to stir for 5 min before HSNAC **29** (1.4 eq., 17.5 mg, 0.15 mmol) was added. After complete addition the reaction mixture was allowed to warm to room temperature and stirred for 4h. After completion, the reaction was quenched by the addition of H_2O (3 mL). The phases were separated, and the aqueous phase was extracted with CH_2Cl_2 (2 × 2 mL). The combined organic phases were dried over $MgSO_4$, filtered and concentrated *in vacuo*. The crude residue was purified by flash chromatography (petroleum ether : ethyl acetate 3:1 to 1:1) to give the desired alkene (7 mg, 0.026 mmol, 24%) as a pure diastereomer based on ^{1}H NMR.

UV$_{\lambda max}$ (CH_3CN:H_2O): 239 nm
^{1}H-NMR (500 MHz, $CDCl_3$): δ = 7.32-7.23 (5H, m, ArH), 6.65 (1H, d, *J* = 1.9 Hz, H-3), 5.53 (1H, brs, H-12), 3.38 (2H, q, *J* = 6.0 Hz, H-11), 2.99 (2H, dd, *J* = 6.8, 5.6 Hz, H-10), 2.10 (3H, d, *J* = 1.9 Hz, H-15), 1.89 (3h, s, H-14) ppm.

^{13}C-NMR (125 MHz, $CDCl_3$): δ = 198.0 (C-1), 170.1 (C-13), 137.7 (C-3), 136.0 (C-4), 131.8 (C-2), 128.6 (C-6/8), 128.2 (C-5/9), 127.9 (C-7), 39.0 (C-11), 28.9 (C-10), 21.7 (C-14) ppm.

ESI-MS (*m/z*): 264.1 $[M + H]^+$, 286.1 $[M + Na]^+$.

HRESIMS (*m/z*): calculated for $C_{14}H_{17}NO_2SNa$ $[M + Na]^+$: 286.0872; found: 286.0869.

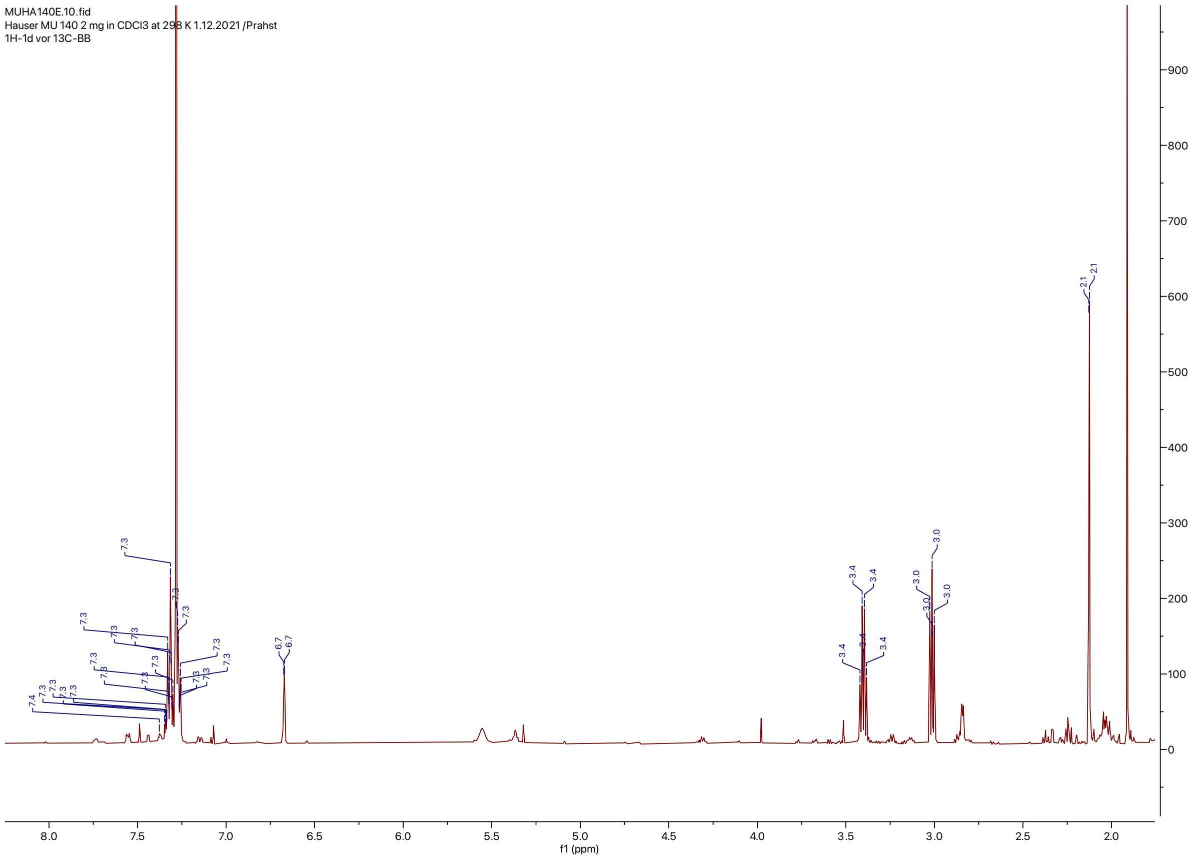

Figure S3.3.4E ^{1}H NMR of *Z*-**10b** in $CDCl_3$.

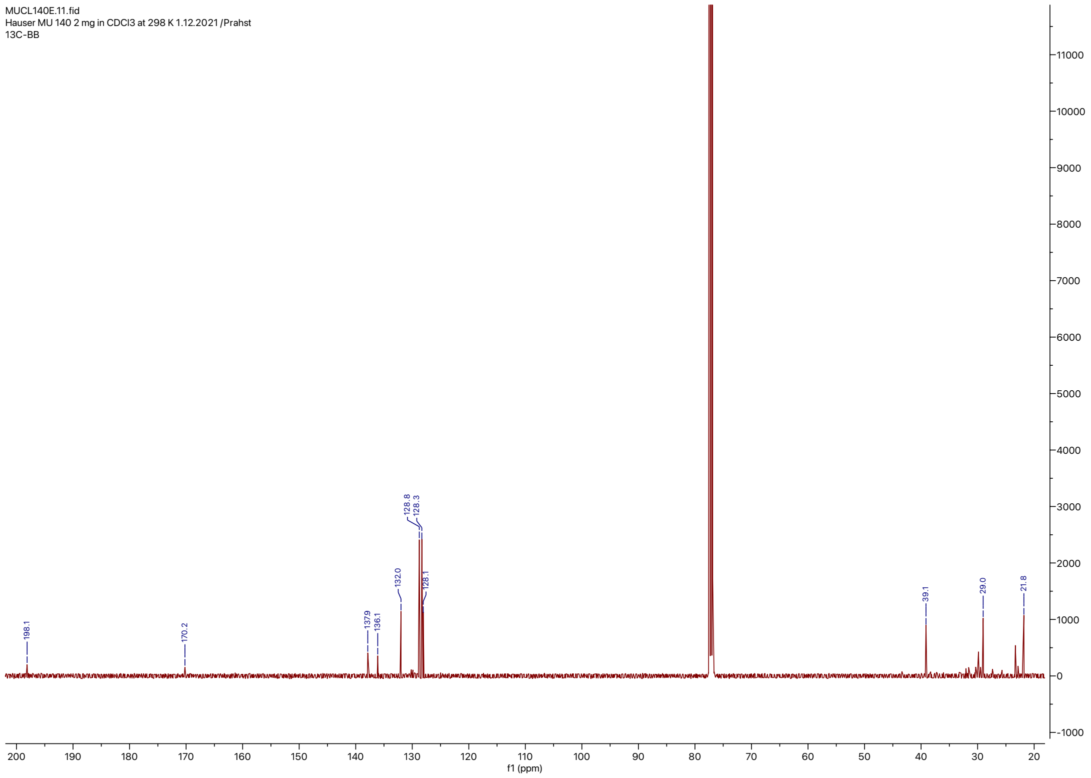

Figure S3.3.4F ^{13}C NMR of *Z*-**10b** in $CDCl_3$.

S-(2-Acetamidoethyl) *E*-2-methyl-3-phenylprop-2-enethioate *E*-10b

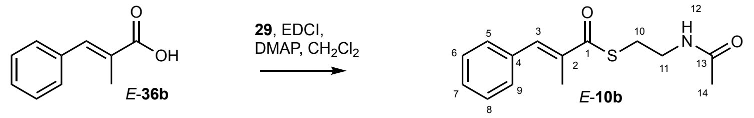

Chemical Formula: $C_{14}H_{17}NO_2S$
Exact Mass: 263.0980

α-Methyl cinnamic acid *E*-**36b** (Sigma-Aldrich, 1.0eq., 50 mg, 0.31 mmol) was dissolved in CH_2Cl_2 (1.5 mL) and cooled to 0 °C. After cooling EDCl (1.5 eq, 88 mg, 0.46 mmol) and DMAP (0.2 eq., 3.7 mg, 0.03 mmol) were added. The solution was allowed to stir for 5 min before HSNAC **29** (1.1 eq., 37.4 mg, 0.32 mmol) was added. After complete addition the reaction mixture was allowed to warm to room temperature and stirred for 4h. After completion, the reaction was quenched by the addition of H_2O (3 mL). The phases were separated, and the aqueous phase was extracted with CH_2Cl_2 (2 × 2 mL). The combined organic phases were dried over $MgSO_4$, filtered and concentrated in *vacuo*. The crude residue was purified by flash chromatography (petroleum ether : ethyl acetate 3:1 to 1:1) to give the desired alkene (67 mg, 0.25 mmol, 81%) as a pure diastereomer based on ^{1}H NMR.

UV$_{\lambda max}$ (CH_3CN:H_2O): 239 nm

^{1}H-NMR (400 MHz, $CDCl_3$): δ = 7.66 (1H, q, *J* = 1.5 Hz, H-3), 7.42 (4H, d, *J* = 4.4 Hz, ArH), 7.36 (1H, m, ArH), 5.90 (1H, brs, H-12), 3.51 (2H, q, *J* = 6.1 Hz, H-11), 3.15 (2H, m, H-10), 2.17 (3H, d, *J* = 1.5 Hz, H-15), 1.99 (3H, s, H-14) ppm.

^{13}C-NMR (100 MHz, $CDCl_3$): δ = 194.7 (C-1), 170.3 (C-13), 137.9 (C-3), 135.9 (C-2), 135.1 (C-4), 129.8 (C-6/8), 128.8 (C-7), 128.5 (C-5/9), 39.8 (C-11), 28.8 (C-10), 23.3 (C-14), 14.2 (C-15) ppm.

ESI-MS (*m/z*): 264.1 $[M + H]^+$, 286.1 $[M + Na]^+$.

HRESIMS (*m/z*): calculated for $C_{14}H_{17}NO_2SNa$ $[M + Na]^+$: 286.0872; found: 286.0873.

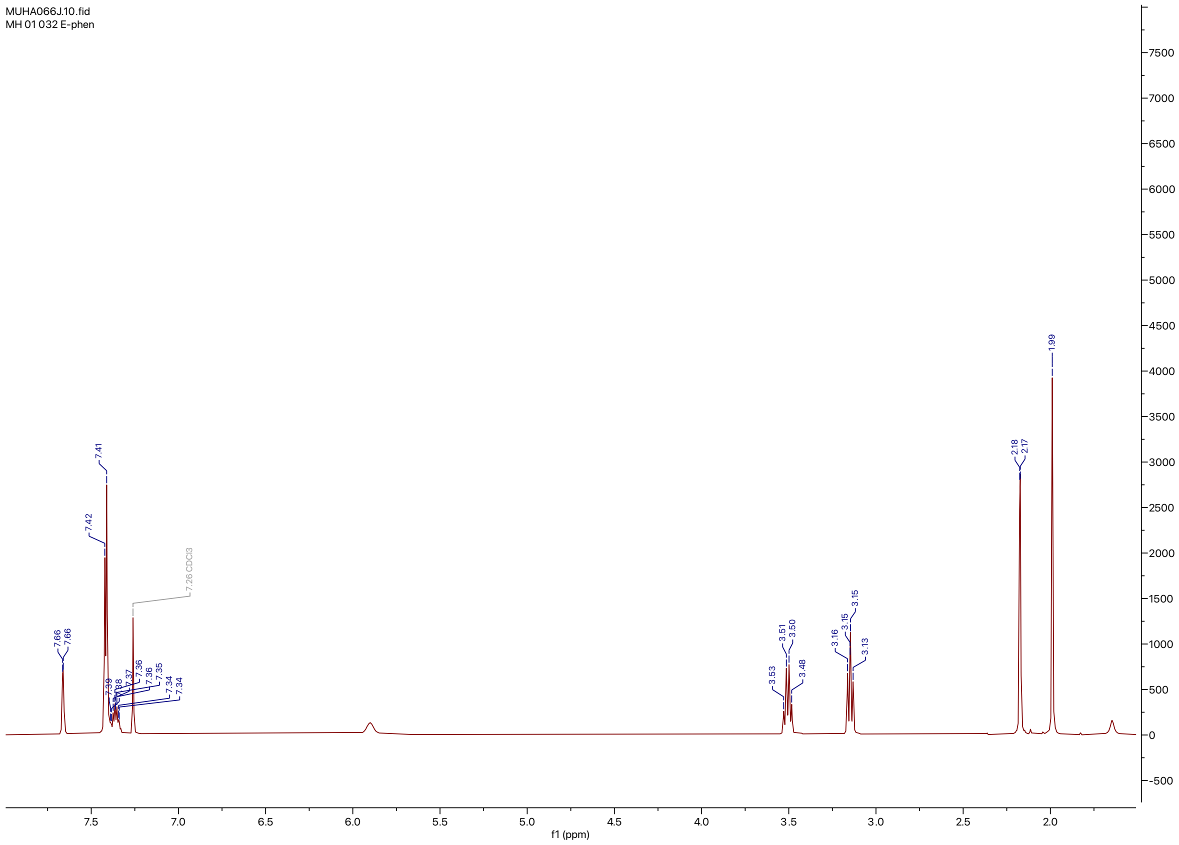

Figure S3.3.4G ^{1}H NMR of *E*-**10b** in $CDCl_3$.

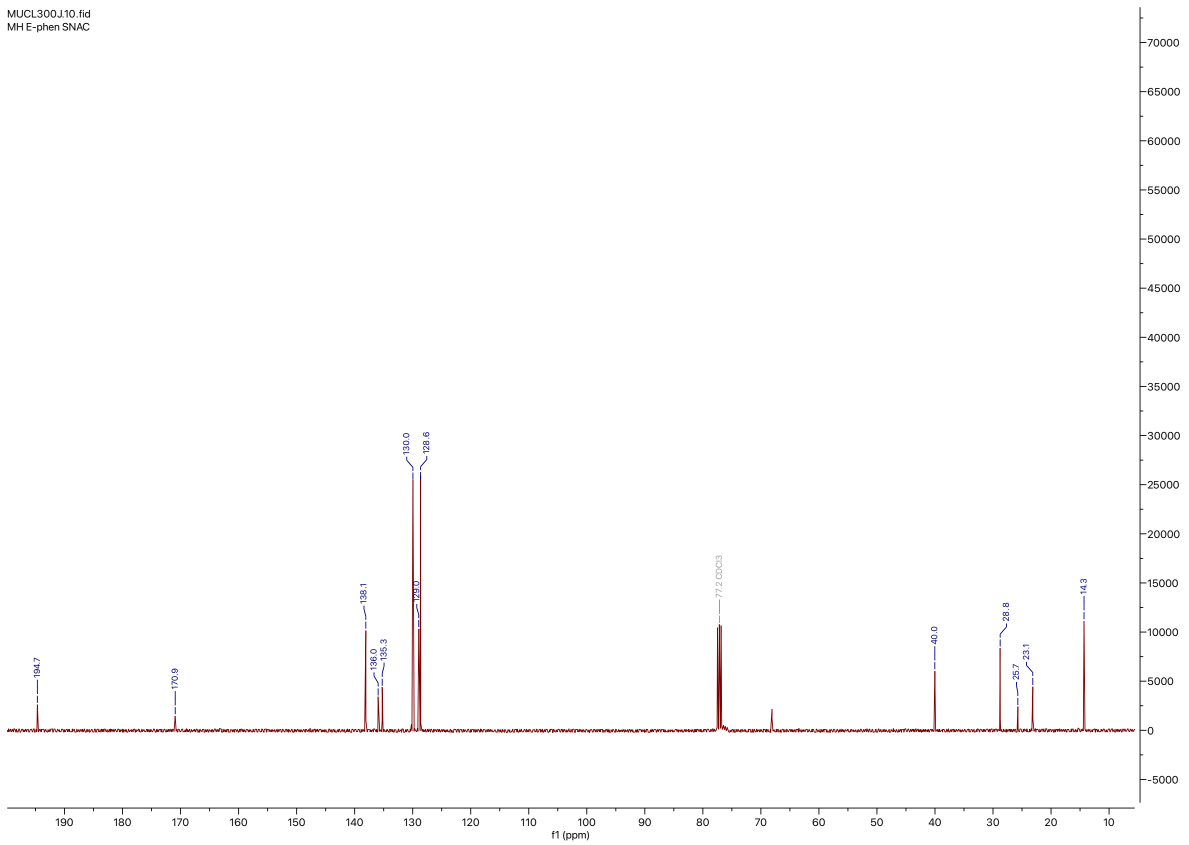

Figure S3.3.4H ^{13}C NMR of *E*-**10b** in $CDCl_3$.

3.3.5 Compound L-*syn*-11b

_R_-4-Benzyl-3-((2_R_,3_S_,_E_)-3-hydroxy-2-methylhex-4-enoyl)oxazolidin-2-one 41b[17]

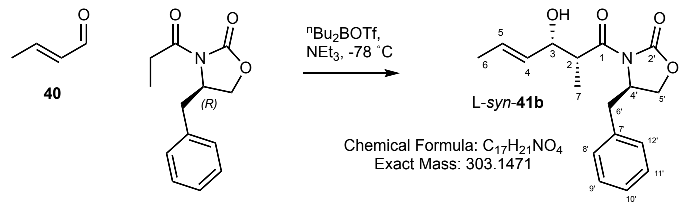

The procedure was followed as described by Evans *et al.* and slightly adapted.[17] (*R*)-4-benzyl-3-propionyloxazolidin-2-one (1.0 eq., 210 mg, 0.90 mmol) was dissolved in CH_2Cl_2 (1 mL) and cooled to 0 °C. To the cooled solution $^nBu_2BOTf$ (1M in THF, 1.2 eq., 1.08 mL, 1.08 mmol) followed by NEt_3 (1.2 eq., 0.15 mL, 1.08 mmol) were added dropwise. The solution was allowed to stir for 5 min before it was cooled to -78 °C. After additional 15 min stirring crotonaldehyde (1.2 eq., 0.4 mL, 1.08 mmol) was added dropwise. The reaction mixture was stirred at - 78 °C for 45 min and allowed to warm to 0 °C over 20 min. After additional 40 min the reaction was cooled to -15 °C and the reaction was slowly quenched by the addition of phosphate buffer (pH 7, 1 mL) and MeOH (2.5 mL). A solution of MeOH/30% H_2O_2 (2:1, 2.5 mL) was added and the solution was allowed to stir for 50 min at 0 °C. The mixture was concentrated in *vacuo* and extracted with Et_2O (3 × 3 mL). The combined organic phases were washed with sat. $NaHCO_3$ and Brine, dried over $MgSO_4$, filtered and concentrated *in vacuo*. The crude residue was purified by flash chromatography (petroleum ether : ethyl acetate 5:2) to give the desired aldol product L-*syn*-**41b**[17] (123 mg, 0.41 mmol, 45%).

$[\alpha]_D^{20}$ (c = 0.44 in $CHCl_3$) = -55.1 (lit. $[\alpha]_D^{20}$ (c = 0.645 in $CHCl_3$) = -58.1)[17]
^{1}H-NMR (400 MHz, $CDCl_3$): δ = 7.36 - 7.27 (3H, m, ArH), 7.22 - 7.19 (2H, m, ArH), 5.77 (1H, dqd, *J* = 15.3, 6.5, 1.2 Hz, H-5), 5.52 (1H, ddq, *J* = 15.3, 6.4, 1.6 Hz, H-4), 4.70 (1H, ddt, *J* = 9.3, 6.9, 3.4 Hz, H-4'), 4.42 (1H, brs/t, H-3), 4.24 - 4.17 (2H, m, H-5'), 3.87 (1H, qd, *J* = 7.0, 3.8 Hz, H-2), 3.26 (1H , dd, *J* = 13.4, 3.4 Hz, H-6'), 2.79 (1H, dd, *J* = 13.4, 9.3 Hz, H-6'), 1.72 (3H, d, *J* = 6.5 Hz, H-6), 1.25 (3H, d, *J* = 7.0 Hz, H-7) ppm.
^{13}C-NMR (100 MHz, $CDCl_3$): δ = 176.8 (C-1), 153.3 (C-2'), 135.2 (C-7'), 130.3 (C-4), 129.6 (C-9'/11'), 129.1 (C-8'/12'), 128.5 (C-5), 127.6 (C-10'), 73.0 (C-3), 66.3 (C-5'), 55.3 (C-4'), 43.0 (C-2), 38.0 (C-6'), 17.9 (C-6), 11.4 (C-7) ppm.
ESI-MS (*m/z*): 326.3 $[M + Na]^+$.

_S_-(2-Acetamidoethyl) (2_R_,3_S_,_E_)-3-hydroxy-2-methylhex-4-enethioate L-_syn_-11b

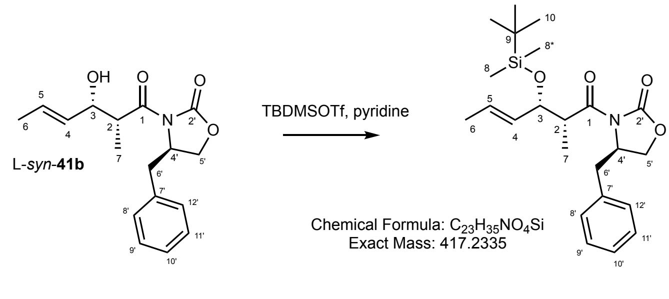

The aldol product L-*syn*-**41b** (1.0 eq., 200 mg, 0.66 mmol) was dissolved in CH_2Cl_2 (2.0 mL) and cooled to 0 °C. To the cooled reaction mixture were added DMAP (0.1 eq., 8 mg, 0.06 mmol) and imidazole (4.0 eq., 180 mg, 2.64 mmol). TBDMS-Cl (2.5 eq., 250 mg, 1.66 mmol) was dissolved in CH_2Cl_2 (2 mL) and added dropwise. After complete addition the reaction mixture was allowed warm to room temperature and stirred until completion. The solution was quenched by the addition of H_2O (5 mL). The

phases were separated, and the aqueous phase was extracted with CH_2Cl_2 (3 × 4 mL). The combined organic phases were washed with brine, dried over $MgSO_4$, filtered and concentrated *in vacuo*. The crude residue was purified by flash chromatography (petroleum ether : ethyl acetate 20:1) to give the protected intermediate (120 mg, 0.28 mmol, 42%) as a colourless oil.

^{1}H-NMR (400 MHz, $CDCl_3$): δ = 7.35 - 7.22 (5H, m, ArH), 5.63 - 5.55 (1H, m, H-5), 5.50 - 5.44 (1H, m, H-4), 4.59 (1H, m, H-4'), 4.25 (1H, t, *J* = 6.8 Hz, H-3), 4.17 - 4.09 (2H, m, H-5'), 3.98 (1H, dq, *J* = 6.8, *J* = 6.8 Hz, H-2), 3.28 (1H, dd, *J* = 13.4, 3.3 Hz, H-6'), 2.76 (1H, dd, *J* = 13.4, 9.7 Hz, H-6'), 1.68 - 1.65 (3H, m, H-6), 1.20 (3H, d, *J* = 6.8 Hz, H-7), 0.87 (9H, s, H-10), 0.01 (3H, s, H-8), -0.01 (3H,s, H-8*) ppm.
^{13}C-NMR (100 MHz, $CDCl_3$): δ = 174.9 (C-1), 153.2 (C-2'), 135.4 (C-7'), 132.2 (C-4), 129.5 (C-9'/11'), 128.9 (C-8'/12'), 127.3 (C-5), 127.1 (C-10'), 75.2 (C-3), 65.9 (C-5'), 55.7 (C-4'), 44.2 (C-2), 37.8 (C-6'), 25.8 (C-10), 18.1 (C-9), 17.6 (C-6), 12.7 (C-7), -4.2 (C-8), -5.0 (C-8*).
ESI-MS (*m/z*): 440.4 $[M + Na]^+$.

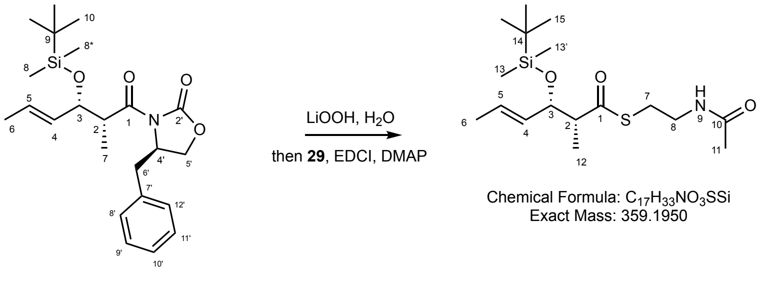

The 3-*O*-TBDMS-protected aldol product as prepared above (1.0 eq., 113 mg, 0.26 mmol) was dissolved in THF (2 mL) and cooled to 0 °C. To the cooled solution 30% H_2O_2 (7.0 eq., 0.13 mL, 1.82 mmol) was added followed by the slow addition of $LiOH{\cdot}H_2O$ (2.5 eq., 27 mg, 0.65 mmol) dissolved in H_2O (0.6 mL). The mixture was allowed to warm to RT and stirred until completion. The solution was quenched with sat. $Na_2S_2O_3$ (2 mL) and concentrated *in vacuo*. The resulting slurry was carefully acidified with HCl (1M) and extracted with EtOAc (3 × 2 mL). The combined organic phases were dried over Na_2SO_4, filtered and concentrated in *vacuo*. The crude residue was purified by flash chromatography (petroleum ether : ethyl acetate 10:1 + 1% formic acid) to give the free acid that was dissolved in CH_2Cl_2 (2 mL) and cooled to 0 °C. After cooling EDCI (1.1 eq, 56 mg, 0.29 mmol) and DMAP (0.2 eq., 6 mg, 0.05 mmol) were added. The solution was allowed to stir for 5 min before HSNAC **29** (1.1 eq., 34 mg, 0.29 mmol) was added. After complete addition the reaction mixture was allowed to warm to room temperature and stirred for 2h. After completion the reaction was quenched by the addition of H_2O (3 mL). The phases were separated, and the aqueous phase was extracted with CH_2Cl_2 (3 × 3 mL). The combined organic phases were dried over $MgSO_4$, filtered and concentrated in *vacuo*. The crude residue was purified by flash chromatography (petroleum ether : ethyl acetate 1:1) to give the desired product as a colourless oil (46 mg, 0.13 mmol, 50%).

$[\alpha]_D^{25}$ (c = 0.3 in $CHCl_3$) = -25.7
^{1}H-NMR (400 MHz, $CDCl_3$): δ = 5.82 (1H, brs, H-9), 5.56 (1H, dq, *J* = 15.3, 0.6 Hz, H-5), 5.41 (1H, ddq, *J* = 15.3, 7.5, 1.5 Hz, H-4), 4.25 - 4.22 (1H, m, H-3), 3.48 - 3.20 (2H, m, H-8), 3.04 - 2.92 (2H, m, H-7), 2.76 - 2.70 (1H, m, H-2), 1.96 (3H, s, H-11), 1.67 (3H, ddd, *J* = 6.4, 1.6, 0.6 Hz, H-6), 1.17 (3H, d, *J* = 6.9 Hz, H-12), 0.87 (9H, s, H-15), 0.02 (3H, s, H-13), -0.01 (3H, s, H-13') ppm.
^{13}C-NMR (100 MHz, $CDCl_3$): δ = 202.6 (C-1), 170.3 (C-10), 132.0 (C-5), 127.6 (C-4), 75.5 (C-3), 55.8 (C-2), 40.0 (C-7), 28.5 (C-8), 25.9 (C-15), 23.4 (C-11), 18.3 (C-14), 17.7 (C-6), 13.2 (C-12), -3.9 (C-13), -4.8 (C-13') ppm.
ESI-MS (*m/z*): 360.4 $[M + H]^+$, 382.3 $[M + Na]^+$.

HRESIMS (*m/z*): calculated for $C_{17}H_{33}NO_3SSiNa$ [M + Na]$^+$: 382.1843 found 382.1845

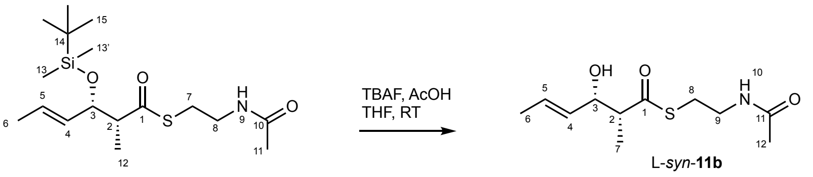

Chemical Formula: $C_{11}H_{19}NO_3S$
Exact Mass: 245.1086

The procedure was followed as described in L-*syn*-**13b**.

[α]$_D^{25}$ (c = 0.5 in $CHCl_3$) = -22.7

^{1}H-NMR (400 MHz, $CDCl_3$): δ = 6.18 (1H, brs, H-10), 5.73 (1H, dqd, *J* = 15.2, 6.5, 1.4 Hz, H-5), 5.47 (1H, ddq, *J* = 15.2, 6.5, 1.4 Hz, H-4), 4.37 - 4.34 (1H, m, H-3), 3.51 - 3.37 (2H, m, H-9), 3.09 - 2.96 (2H, m, H-8), 2.79 (1H, qd, *J* = 7.1, 4.4 Hz, H-2), 1.99 (3H, s, H-12), 1.70 - 1.68 (3H, ddd, *J* = 6.4, 1.7, 0.9 Hz, H-6), 1.20 (d, *J* = 7.0 Hz, H-7) ppm.

^{13}C-NMR (100 MHz, $CDCl_3$): δ = 203.4 (C-1), 171.2 (C-11), 130.3 (C-4), 128.8 (C-5), 73.6 (C-3), 53.9 (C-2), 39.7 (C-9), 28.5 (C-8), 23.1 (C-12), 17.9 (C-6), 12.0 (C-7) ppm.

ESI-MS (*m/z*): 246.3 [M + H]$^+$, 268.3 [M + Na]$^+$, 228.3 [M - H_2O + H]$^+$,

HRESIMS (*m/z*): calculated for $C_{11}H_{19}NO_3SNa$ [M + Na]$^+$: 268.0978 found 268.0981

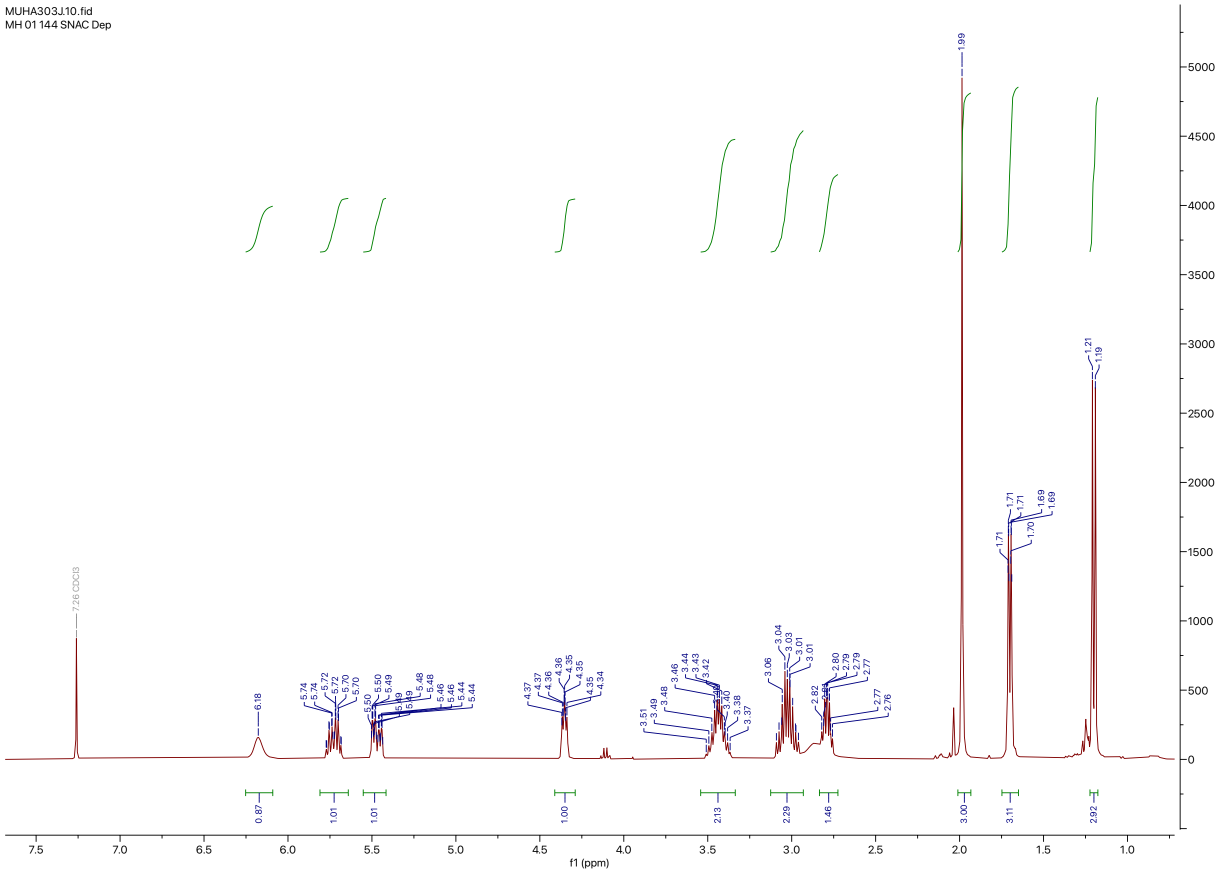

Figure S3.3.5A ^{1}H NMR of *L-syn*-**11b** in $CDCl_3$.

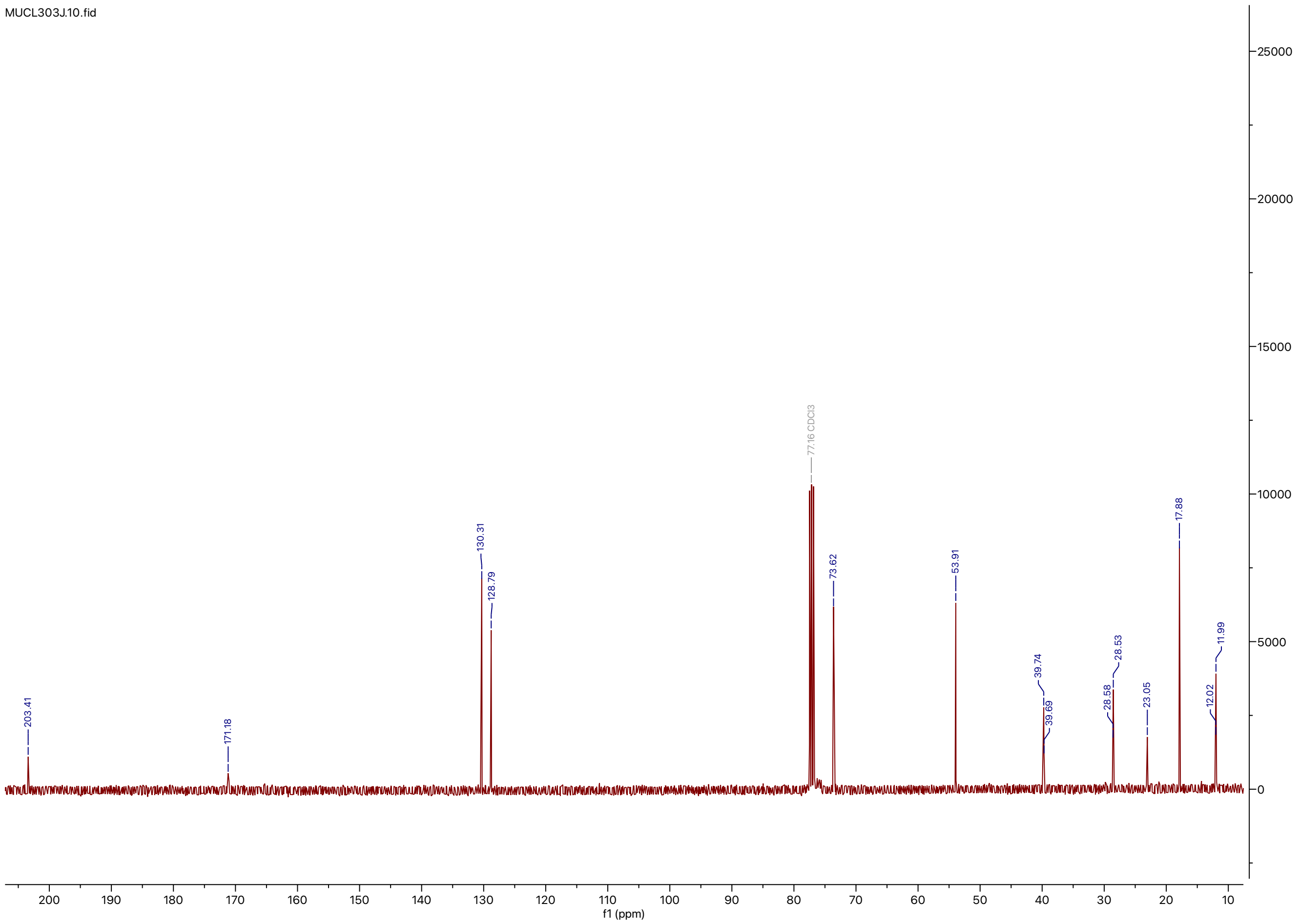

Figure S3.3.5B ^{13}C NMR of *L-syn*-**11b** in $CDCl_3$.

3.3.6 Compounds 13

(±)-Ethyl *E*-3-hydroxy-5-phenylpent-4-enoate 38a[18]

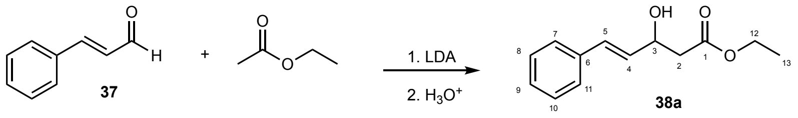

Ethyl acetate (1.5 eq., 0.34 mL, 3.41 mmol) was dissolved in THF (2 mL) and cooled to - 78 °C. After cooling LDA (2M, 1.3 eq., 1.5 mL, 2.95 mmol) was slowly added. The solution was allowed to stir for 20 min before the dropwise addition of cinnamaldehyde (1.0 eq., 0.29 mL, 2.27 mmol). The solution was allowed to warm slowly until completion and carefully quenched with sat. NH_4Cl (3 mL), while the reaction mixture was still cooled. After stirring vigorously for 5 min at room temperature the phases were separated, and the aqueous phase was extracted with EtOAc (2 × 5 mL). The combined organic phases were washed with brine (4 mL), dried over $MgSO_4$, filtered and concentrated in *vacuo*. The crude residue was purified by flash chromatography (petroleum ether : ethyl acetate 4:1 to 3:1) to give **38a**[18] (354 mg, 1.61 mmol, 71%) as a pale yellow oil.

UV$_{λmax}$ (CH_3CN:H_2O): 209, 248 nm
^{1}H-NMR (400 MHz, $CDCl_3$): δ = 7.39 - 7.23 (5H, m, ArH), 6.66 (1H, dd, *J* = 16.0, 1.4 Hz, H-5), 6.23 (1H, dd, *J* = 16.0, 6.1 Hz, H-4), 4.73 (1H, dddd, *J* = 7.8, 6.0, 4.4, 1.4 Hz, H-3), 4.19 (2H, q, *J* = 7.1 Hz, H-14), 2.70 - 2.58 (2H, m, H-2), 1.28 (3H, t, *J* = 7.1Hz, H-15) ppm.
ESI-MS (*m/z*): 203.3 $[M - H_2O + H]^+$, 243.3 $[M + Na]^+$.

Ethyl *E*-3-hydroxy-2-methyl-5-phenylpent-4-enoate (±)-38b[19]

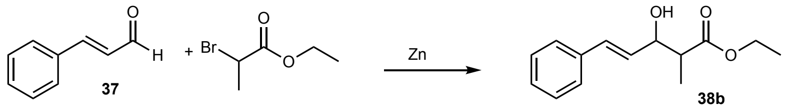

Zinc (1.1 eq., 0.62 g, 9.12 mmol) was activated for 16 min with 2N HCl and then washed with two portions each of water, acetone and toluene. It was then suspended in 4 mL of dry toluene, and a mixture of cinnamaldehyde (1 eq., 1 ml, 7.94 mmol) and ethyl 2-bromopropionate (1 eq., 1.03 ml, 7.94 mmol) was added dropwise under nitrogen atmosphere. The reaction temperature was controlled by the addition rate and/or cooling. After complete addition, the mixture was refluxed for additional 2.5 h. After cooling to rt, 2N HCl (6.4 mL) was added, the phases were separated, and the organic layer was washed successively with 2N HCl (10 mL), sat. $NaHCO_3$ solution (10 mL) and brine (10 mL). Then dried over $MgSO_4$, filtered and concentrated in *vacuo*. The crude residue was purified by flash chromatography (petroleum ether: acetone 6:1) to give the title compound (±)-*E*-**38b**[19] as a light yellow oil (835 mg, 3.56 mmol, 39%) and a diastereomeric ratio of 1.2:1 (*anti : syn*) based on ^{1}H NMR.

anti **^{1}H-NMR** (400 MHz, $CDCl_3$): δ = 7.40-7.36 (2H, m, H-2′/6′), 7.35-7.29 (2H, m, H-3′/5′), 7.27-7.21 (1H, m, H-4′), 6.66 (1H, dd, *J* = 15.9, 1.4 Hz, H-5), 6.20 (1H, dd, *J* = 15.9, 6.1 Hz, H-4), 4.58 (1H, dtd, *J* = 5.9, 4.4, 1.4 Hz, H-3), 4.18 (2H, q, *J* = 7.1 Hz, OCH_2), 2.87-2.82 (1H, overlapped, OH), 2.72 (1H, qd, *J* = 7.2, 4.3 Hz, H-2), 1.26 (3H, t, *J* = 7.1 Hz, OCH_2CH_3), 1.23 (3H, d, *J* = 7.3 Hz, 2-Me);

^{13}C-NMR (100 MHz, $CDCl_3$): δ = 175.3 (C-1), 136.5 (C-1′), 131.5 (C-5), 128.6 (C-4), 128.52 (C-3′/5′), 127.7 (C-4′), 126.48 (C-2′/6′), 73.0 (C-3), 60.73 (-OCH_2), 45.0 (C-2), 14.2 (2-CH_3), 14.17 (-OCH_2CH_3);
ESI-MS (*m/z*): 217.2 $[M + H - H_2O]^+$, 257.3 $[M + Na]^+$.

syn **^{1}H-NMR** (400 MHz, $CDCl_3$): δ = 7.40-7.36 (2H, m, H-2′/6′), 7.35-7.29 (2H, m, H-3′/5′), 7.27-7.21 (1H, m, H-4′), 6.64 (1H, br. d, *J* = 15.9 Hz, H-5), 6.19 (1H, dd, *J* = 15.9, 6.9 Hz, H-4), 4.38 (1H, tdd, *J* = 6.9, 5.2, 1.1 Hz, H-3), 4.19 (2H, q, *J* = 7.1 Hz, OCH_2), 2.87-2.82 (1H, overlapped, OH), 2.66 (1H, p, *J* = 7.2 Hz, H-2), 1.27 (3H, t, *J* = 7.1 Hz, OCH_2CH_3), 1.22 (3H, d, *J* = 7.2 Hz, 2-Me);
^{13}C-NMR (100 MHz, $CDCl_3$): δ = 175.5 (C-1), 136.4 (C-1′), 132.1 (C-5), 129.3 (C-4), 128.54 (C-3′/5′), 127.8 (C-4′), 126.53 (C-2′/6′), 74.6 (C-3), 60.71 (OCH_2), 45.6 (C-2), 11.5 (2-CH_3), 14.15 (OCH_2CH_3);
ESI-MS (*m/z*): 217.2 $[M + H - H_2O]^+$, 257.3 $[M + Na]^+$.

S-(2-Acetamidoethyl) *E*-3-hydroxy-5-phenylpent-4-enethioate 13a

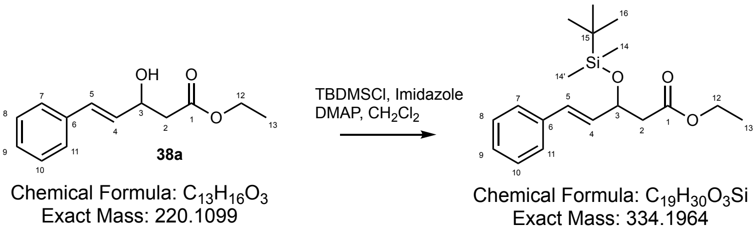

Ethyl *E*-3-hydroxy-5-phenylpent-4-enoate **38a**, as prepared above (1.0 eq., 200 mg, 0.91 mmol) was dissolved in anhydrous CH_2Cl_2 (3 mL) under nitrogen and cooled to 0 °C. Then, imidazole (2 eq., 124 mg, 1.82 mmol), DMAP (0.1 eq., 10 mg, 0.1 mmol) and *tert*-butyldimethylsilyl chloride (2 eq., 272 mg, 1.82 mmol) were added. The reaction was stirred at 0 °C for 1h before the ice bath was removed. After completion the reaction was quenched with H_2O (3 mL). The layers were separated, and the aqueous layer was extracted with CH_2Cl_2 (2 × 4 mL). The organic phases were combined, washed successively with $NaHCO_3$ solution, NH_4Cl, dried over $MgSO_4$, filtered and the concentrated in *vacuo*. The crude residue was purified by flash chromatography (petroleum ether: ethyl acetate 20:1) to give the pure title compound (296 mg, 0.88 mmol, 96%) as a clear oil.

^{1}H-NMR (400 MHz, $CDCl_3$): δ = 7.38 - 7.22 (5H, m, ArH), 6.57 (1H, dd, *J* = 15.9, 1.1 Hz, H-5), 6.19 (1H, dd, *J* = 15.9, 6.7 Hz, H-4), 4.77 (1H, dddd, *J* = 8.0, 6.7, 5.2, 1.1 Hz, H-3), 4.14 (2H, p, *J* = 7.1 Hz, H-12), 2.62 (1H, dd, *J* = 14.5, 8.0 Hz, H-2), 2.52 (1H, dd, *J* = 14.5, 5.2 Hz, H-2), 1.26 (3H, t, *J* = 7.1 Hz, H-13), 0.89 (9H, s, H-16), 0.08 (3H, s, H-14), 0.06 (3H, s, H-14’) ppm.
^{13}C-NMR (100 MHz, $CDCl_3$): δ = 171.2 (C-1), 136.8 (C-6), 131.8 (C-5), 130.1 (C-9), 128.7 (C-8/10), 127.8 (C-4), 126.6 (C-7/11), 70.9 (C-3), 60.6 (C-12), 44.2 (C-2), 25.9 (C-16), 18.3 (C-15), 14.4 (C-13), -4.1 (C-14), -4.9 (C-14’) ppm.
ESI-MS (*m/z*): 357.4 $[M + Na]^+$.

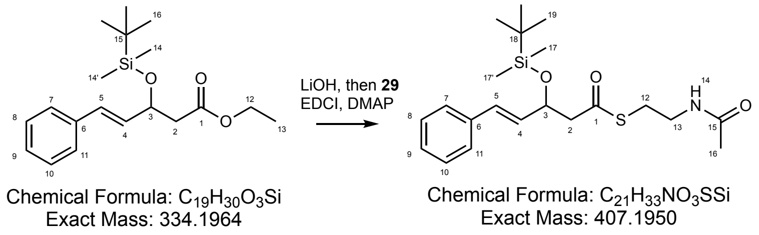

To ethyl *E*-3-((tert-butyldimethylsilyl)oxy)-5-phenylpent-4-enoate as prepared above (1.0 eq., 150 mg, 0.48 mmol) in THF (0.5 mL), MeOH (1 mL) and LiOH·H_2O (3.0 eq., 61 mg, 1.44 mmol) in water (0.5 mL) were added. The reaction mixture was heated

to 40 °C overnight. The reaction was diluted with water (5 mL), and acidified to pH 3.0 with HCl (2 N) and extracted with EtOAc (3 × 5 mL). The organic layers were combined, washed with brine, dried over $MgSO_4$, filtered and concentrated *in vacuo* to give E-3-((tert-butyldimethylsilyl)oxy)-2-methyl-5-phenylpent-4-enoic acid as a white solid (112 mg, 0.37 mmol, 78%).

To a solution of the 3-*O*-TBDMS protected acid as prepared above (1.0 eq. 30 mg, 0.10 mmol) in CH_2Cl_2 (2 mL) was added EDCl (2.5 eq., 47 mg, 0.122 mmol) and DMAP (0.2 eq, 2.4 mg, 0.010 mmol) at 0 °C. After stirring for 10 min HSNAC **29** (1.2 eq., 14 mg, 0.12 mmol) was added and the reaction mixture was allowed to warm to RT and stirred overnight. The reaction was quenched with water (3 mL) and extracted with CH_2Cl_2 (3 × 3 mL). The combined organic phases were dried over $MgSO_4$, filtered and concentrated in *vacuo*. The crude residue was purified by flash chromatography (petroleum ether : ethyl acetate 1:1) to give pure title compound (35 mg, 0.09 mmol, 85%) as a colourless oil.

^{1}H-NMR (400 MHz, $CDCl_3$): δ = 7.37 - 7.23 (5H, m, ArH), 6.55 (1H, d, *J* = 15.8 Hz, 1 H, H-5), 6.08 (1H, dd, *J* = 15.8, 6.8 Hz, H-4), 4.77 (1H, ddd, *J* = 8.0, 6.8, 5.0 , H-3), 3.47 - 3.33 (2H, m, H-13), 3.09 - 2.95 (2H, m, H-12), 2.90 (1H, dd, *J* = 14.2, 8.0 Hz, 2 H, H-2), 2.75 (1H, dd, *J* = 14.2, 5.0 Hz, H-2), 1.91 (3 H, s, H-16), 0.89 (9H, s, 9 H, H-19), 0.07 (6H, s, H-17 & H-17’) ppm.

^{13}C-NMR (100 MHz, $CDCl_3$): δ = 197.3 (C-1), 170.3 (C-15), 136.3 (C-6), 131.1 (C-5), 130.2 (C-9), 128.6 (C-8/10), 127.8 (C-4), 126.4 (C-7/11), 52.6 (C-2), 42.1 (C-13), 39.5 (C-12), 28.8 (C-18), 25.7 (C-19), 23.1 (C-16), −4.3 (C-17/17’) ppm.

ESI-MS (*m/z*): 407.2 $[M + H]^+$.

HRESIMS (*m/z*): calculated for $C_{21}H_{33}NO_3SSiNa$ $[M + Na]^+$: 430.1843 found 430.1844

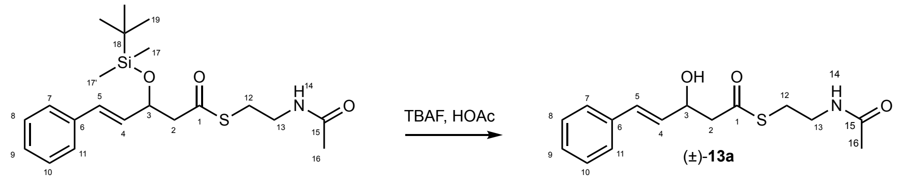

Protected silyl ether as prepared above (1.0 eq., 9 mg, 0.021 mmol) was dissolved in THF (1 mL) and cooled to 0 °C. In a separate vial were added TBAF (1M in THF, 0.2 mL) and glacial acetic acid (20 µL) and mixed. This mixture was then transferred to the reaction mixture and added dropwise. The reaction was allowed to warm to room temperature and stirred for 2 days. The reaction progress was checked by LCMS. When there was starting material left another equivalent of TBAF + AcOH was added, and the mixture was stirred for additional 2 days. The reaction was quenched with sat. aqueous NH_4Cl (2 mL) and extracted with Et_2O (3 × 2 mL). The combined organic layers were washed with sat. aqueous NH_4Cl (2 × 2 mL), dried over $MgSO_4$, filtered and concentrated *in vacuo* to give the desired product (4.1 mg, 0.014 mmol, 64%) as a colourless oil.

UVmax ($CH_3CN:H_2O$): 217 nm

^{1}H-NMR (400 MHz, $CDCl_3$): δ = 7.42 - 7.28 (5H, m, ArH), 6.66 (1H, d, *J* = 15.9 Hz, H-5), 6.20 (1H, dd, *J* = 15.9, 6.2 Hz, H-4), 5.77 (1H, brs, H-14), 4.78 (1H, ddd, *J* = 6.5, 6.2, 1.4 Hz, H-3), 3.50 - 3.38 (2H, m, H-13), 3.12 - 3.00 (2H, m, H-12), 2.90 (2H, d, *J* = 5.5 Hz, H-2), 1.93 (3H, s, H-16) ppm.

^{13}C-NMR (100 MHz, $CDCl_3$): δ = 198.5 (C-1), 170.5 (C-15), 136.2 (C-6), 131.0 (C-5), 129.6 (C-9), 128.6 (C-7/11), 127.9 (C-4), 126.5 (C-8/10), 69.5 (C-3), 50.9 (C-2), 39.2 (C-13), 29.6 (C-12), 23.1 (C-16) ppm.

ESI-MS (*m/z*): 316.1 $[M + Na]^+$, 276.1 $[M - H_2O + H]^+$.

HRESIMS (*m/z*): calculated for $C_{15}H_{19}NO_3SNa$ $[M + Na]^+$: 316.0978 found 316.0991.

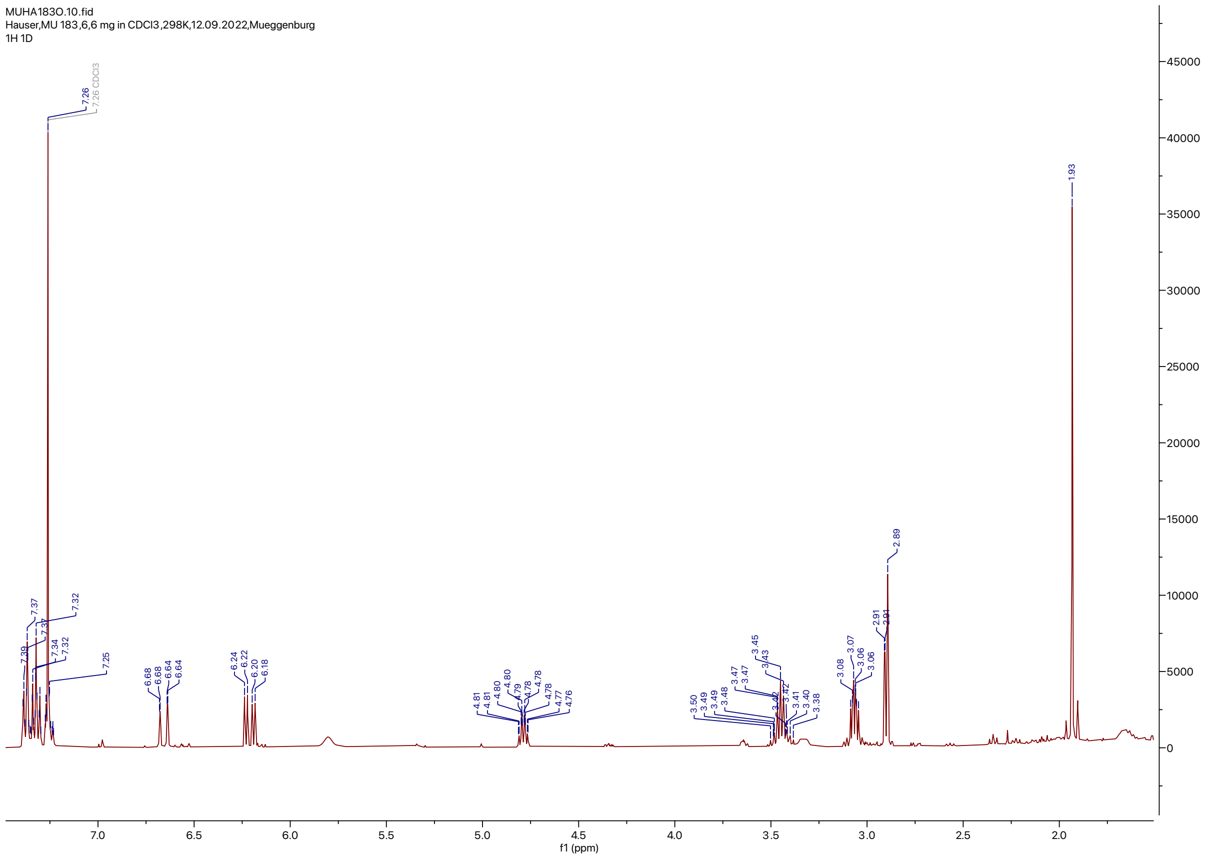

Figure S3.3.6A ^{1}H NMR of (±)-**13a** in $CDCl_3$.

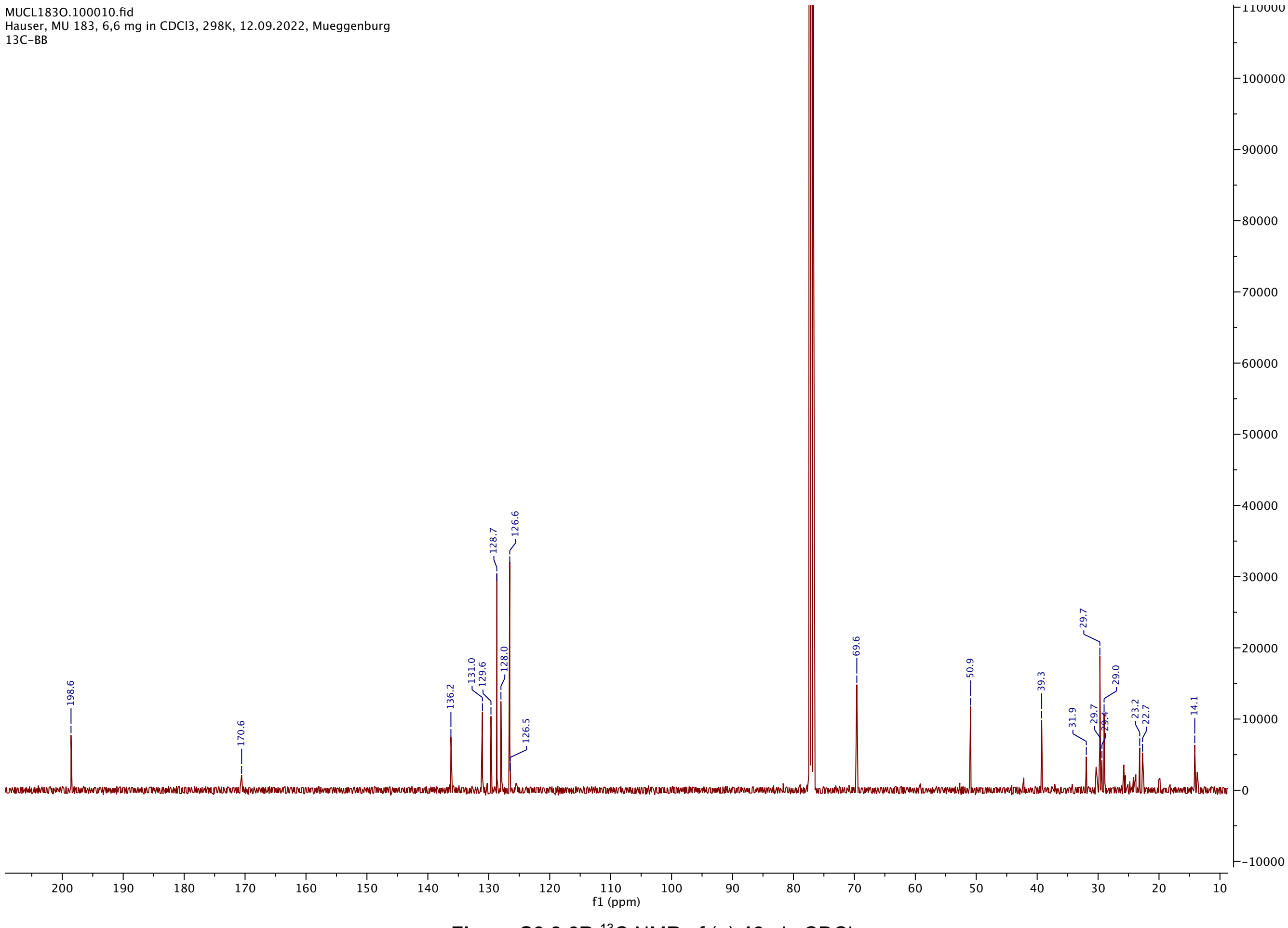

Figure S3.3.6B ^{13}C NMR of (±)-**13a** in $CDCl_3$.

Ethyl *E*-3-((tert-butyldimethylsilyl)oxy)-2-methyl-5-phenylpent-4-enoate

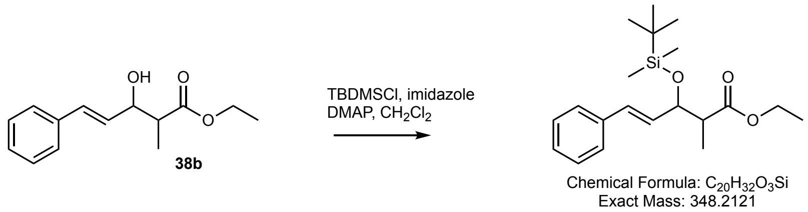

Ethyl *E*-3-hydroxy-2-methyl-5-phenylpent-4-enoate **38b**, as prepared above (787.5 mg, 3.37 mmol) was dissolved in anhydrous CH_2Cl_2 (5 mL) under nitrogen. Then, imidazole (2 eq.) and *tert*-butyldimethylsilyl chloride (2 eq.) was added. The reaction was stirred at RT overnight, before quench with H_2O (5 mL). The layers were separated, and the aqueous layer was extracted with CH_2Cl_2 (2 × 5 mL). The organic phases were combined, washed successively with $NaHCO_3$ solution, NH4Cl, and brine, then dried ($MgSO_4$), filtered. Removal of the solvent *in vacuo* yielded 1.1015 g (3.16 mmol, 94%) of the title compound as a light-yellowish oil and a diastereomeric mixture (major/minor ≈ 1.2/1 based on ^{1}H NMR).

major:

^{1}H-NMR (400 MHz, $CDCl_3$): δ = 7.40-7.29 (4H, m, Ph-2′,3′,5′,6′), 7.28-7.21 (1H, m, H-4′), 6.52 (1H, dd, *J* = 16.0 Hz, H-5), 6.19 (1H, dd, *J* = 15.9, 7.0 Hz, H-4), 4.54 (1H, ddd, *J* = 7.0, 5.9, 1.1 Hz, H-3), 4.10 (2H, overlapped, OCH_2), 2.60 (1H, m, H-2), 1.22 (3H, t, *J* = 7.1 Hz, OCH_2CH_3), 1.21 (3H, d, *J* = 7.0 Hz, 2-Me), 0.91 (9H, s, $SiC(CH_3)_3$), 0.07 (3H, s, $SiCH_3$), 0.03 (3H, s, $SiCH_3$);

^{13}C-NMR (100 MHz, $CDCl_3$): δ = 174.3 (C-1), 136.8 (C-1′), 131.0 (C-5), 130.6 (C-4), 128.5 (C-3′/5′), 127.5 (C-4′), 126.4 (C-2′/6′), 75.0 (C-3), 60.28 (-OCH_2), 47.2 (C-2), 25.8 ($SiC(CH_3)_3$), 18.1 ($SiC(CH_3)_3$), 14.2 (-OCH_2CH_3), 12.0 (2-CH_3), -4.1 ($SiCH_3$), -5.0 ($SiCH_3$);

ESI-MS (*m/z*): 371.4 $[M + Na]^+$.

minor:

^{1}H-NMR (400 MHz, $CDCl_3$): δ = 7.40-7.29 (4H, m, Ph-2′,3′,5′,6′), 7.28-7.21 (1H, m, H-4′), 6.53 (1H, d, *J* = 15.9 Hz, H-5), 6.08 (1H, dd, *J* = 16.0, 7.7 Hz, H-4), 4.44 (1H, td, *J* = 7.9, 0.9 Hz, H-3), 4.15 (2H, overlapped, OCH_2), 2.62 (1H, m, H-2), 1.28 (3H, t, *J* = 7.1 Hz, OCH_2CH_3), 1.08 (3H, d, *J* = 7.1 Hz, 2-Me), 0.88 (9H, s, $SiC(CH_3)_3$), 0.07 (3H, s, $SiCH_3$), 0.03 (3H, s, $SiCH_3$);

^{13}C-NMR (100 MHz, $CDCl_3$): δ = 174.9 (C-1), 136.6 (C-1′), 131.8 (C-5), 130.2 (C-4), 128.6 (C-3′/5′), 127.7 (C-4′), 126.5 (C-2′/6′), 76.1 (C-3), 60.30 (-OCH_2), 47.4 (C-2), 25.7 ($SiC(CH_3)_3$), 18.0 ($SiC(CH_3)_3$), 14.2 (-OCH_2CH_3), 13.2 (2-CH_3), -4.0 ($SiCH_3$), -5.1 ($SiCH_3$);

ESI-MS (*m/z*): 371.4 $[M + Na]^+$.

S-(2-acetamidoethyl) *E*-3-((*tert*-butyldimethylsilyl)oxy)-2-methyl-5-phenylpent-4-enethioate

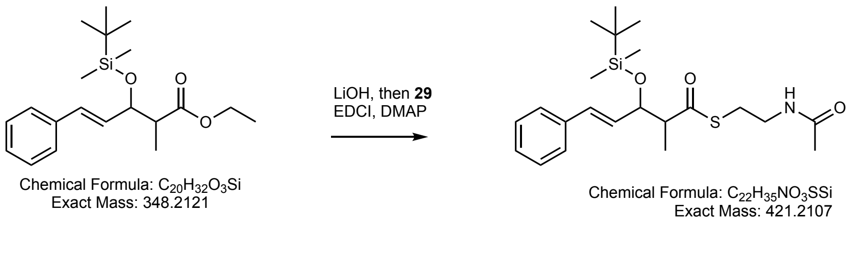

To ethyl *E*-3-((tert-butyldimethylsilyl)oxy)-2-methyl-5-phenylpent-4-enoate as prepared above (305 mg, 0.86 mmol) in THF (0.75 mL), MeOH (1.8 mL) and lithium hydroxide (61.9 mg, 3eq) in water (0.3 mL) were added. The reaction mixture was heated to 60 °C overnight. The reaction was diluted with water (5 mL), and acidified to pH 1.0 with HCl (6 N) and extracted with EtOAc (3 × 13 mL). The organic layers were combined, washed with brine, dried ($MgSO_4$), filtered and concentrated *in vacuo* to give E-3-((tert-butyldimethylsilyl)oxy)-2-methyl-5-phenylpent-4-enoic acid as a yellowish oil (156.2 mg, 0.487 mmol, 56%). To this acid (156.2 mg, 0.487 mmol) in anhydrous CH_2Cl_2 (3 mL) under nitrogen was added EDCI (1.2eq), DMAP (0.2eq), and HSNAC (69.8 mg, 1.2eq). The mixture was stirred overnight at RT. The reaction was quenched with water (8 mL), and CH_2Cl_2 (5 mL) was added, then the layers were separated. The organic phases were combined, washed successively with $NaHCO_3$ solution, NH_4Cl, and brine, then dried ($MgSO_4$), filtered. Removal of the solvent *in vacuo* yielded 149.1 mg (0.35 mmol, 73%) of the title compound as a light-yellowish oil, and as a mixture of diastereomers (major/minor ≈ 1.2/1 based on ^{1}H NMR).

major:

^{1}H-NMR (400 MHz, $CDCl_3$): δ = 7.40-7.23 (5H, m, Ph), 6.52 (1H, d, *J* = 15.9 Hz, H-5), 6.05 (1H, dd, *J* = 15.9, 7.7 Hz, H-4), 5.82 (1H, brs), 4.45 (1H, m, H-3), 3.52-3.35 (2H, m, N-CH_2), 3.25-2.80 (3H, m, S-CH_2, and H-2), 1.94 (3H, s, CO-CH_3), 1.25 (3H, d, *J* = 6.9 Hz, 2-Me), 0.86 (9H, s, SiC(CH_3)$_3$), 0.04 (3H, s, SiCH_3), 0.01 (3H, s, SiCH_3);

^{13}C-NMR (100 MHz, $CDCl_3$): δ = 202.7 (C-1), 170.1 (N-CO), 136.39 (C-1′), 132.1 (C-5), 129.8 (C-4), 128.7 (C-3′/5′), 127.89 (C-4′), 126.5 (C-2′/6′), 76.1 (C-3), 55.5 (C-2), 39.7 (NCH_2), 28.6 (SCH_2), 25.7 (SiC(*C*H$_3$)$_3$), 23.2 (CO*C*H$_3$), 18.1 (Si*C*(CH$_3$)$_3$), 14.1 (2-CH_3), -3.95 (SiCH_3), -5.2 (SiCH_3);

ESI-MS (*m/z*): 444.4 [M + Na]$^+$; 466.2 [M + HCOO]$^-$.

minor:

^{1}H-NMR (400 MHz, $CDCl_3$): δ = 7.40-7.23 (5H, m, Ph), 6.45 (1H, d, *J* = 15.9 Hz, H-5), 6.16 (1H, dd, *J* = 15.9, 7.4 Hz, H-4), 5.50 (1H, brs), 4.42 (1H, m, H-3), 3.52-3.35 (2H, m, N-CH_2), 3.25-2.80 (3H, m, S-CH_2, and H-2), 1.79 (3H, s, CO-CH_3), 1.09 (3H, d, *J* = 7.0 Hz, 2-Me), 0.90 (9H, s, SiC(CH_3)$_3$), 0.07 (3H, s, SiCH_3), 0.02 (3H, s, SiCH_3);

^{13}C-NMR (100 MHz, $CDCl_3$): δ = 202.0 (C-1), 170.1 (N-CO), 136.43 (C-1’), 130.9 (C-5), 130.6 (C-4), 128.8 (C-3′/5′), 127.86 (C-4′), 126.4 (C-2′/6′), 75.5 (C-3), 55.8 (C-2), 39.4 (NCH_2), 28.8 (SCH_2), 25.8 (SiC(*C*H$_3$)$_3$), 23.0 (CO*C*H$_3$), 18.0 (Si*C*(CH$_3$)$_3$), 13.3 (2-CH_3), -4.03 (SiCH_3), -4.9 (SiCH_3);

ESI-MS (*m/z*): 444.4 [M + Na]$^+$; 466.2 [M + HCOO]$^-$.

(±)-*syn*-13b and (±)-*anti*-13b

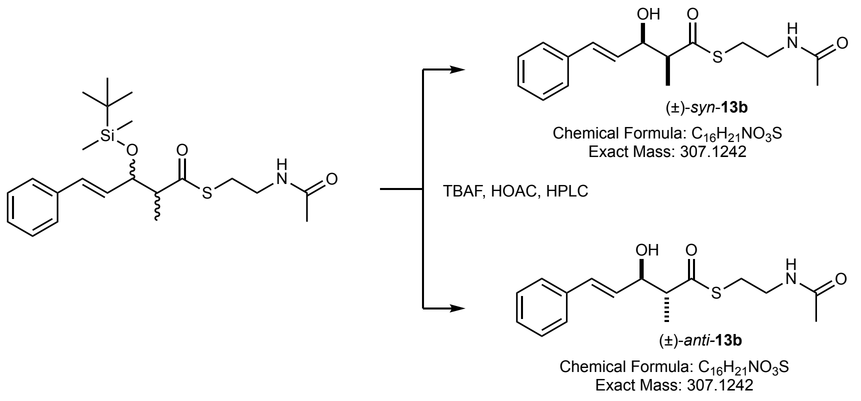

To *S*-(2-acetamidoethyl) *E*-3-((*tert*-butyldimethylsilyl)oxy)-2-methyl-5-phenylpent-4-enethioate as prepared above (140.6 mg, 0.33 mmol), TBAF in THF (10 eq) and HOAc (11 eq) were added. The mixture was stirred at room temperature under N_2 atmosphere for 27 h. Then, quenched with 5 mL of H_2O, and extracted with dichloromethane (2 × 5 mL). The organic layers were combined, washed with brine, dried ($MgSO_4$), filtered and concentrated *in vacuo* to give a yellowish oil. The residue was subjected to LCMS purification on a Kinetex® C_{18} column (i.d., 250 × 21.2 mm, 20 mL/min) eluting with a gradient of CH_3CN in H_2O to yield (±)-*syn*-**13b** (8.8 mg, 0.029 mmol, 8.8%), (±)-*anti*-**13b** (12.4 mg, 0.040 mmol, 12.1%), as colourless oils, and 2*E*-**14b** (21.1 mg, 0.073 mmol, 22.1%) as a light-greenish amorphous solid.

(±)-*syn*-13b:

^{1}H-NMR (400 MHz, $CDCl_3$): δ = 7.40 - 7.24 (5H, m, Ph), 6.63 (1H, dd, *J* = 15.9, 1.3 Hz, H-5), 6.19 (1H, dd, *J* = 15.9, 6.3 Hz, H-4), 5.71 (1H, s, NH), 4.59 (1H, ddd, *J* = 6.3, 4.8, 1.3 Hz, H-3), 3.40 (2H, m, N-CH_2), 3.11-2.94 (2H, m, S-CH_2), 2.91 (1H, m, H-2), 1.88 (3H, s, CO-CH_3), 1.28 (3H, d, *J* = 7.0 Hz, 2-CH_3);

^{13}C-NMR (100 MHz, $CDCl_3$): δ = 203.1 (C-1), 170.5 (N-CO), 136.3 (C-1′), 131.7 (C-5), 128.6 (C-4), 128.7 (C-3′/5′), 127.9 (C-4′), 126.5 (C-2′/6′), 73.5 (C-3), 53.9 (C-2), 39.2 (N-CH_2), 28.8 (S-CH_2), 23.1 (CO*C*H_3), 12.1 (2-CH_3);

ESI-MS (*m/z*): 308.3 [M + H]+ , 330.3 [M + Na]+ ; 306.2 $[M - H]^-$, 352.2 $[M + HCOO]^-$.

HRESIMS (*m/z*): 330.1138 [M + Na]+ (calc. for $C_{16}H_{21}NO_3SNa$, 330.1140).

(±)-*anti*-13b:

^{1}H-NMR (400 MHz, $CDCl_3$): δ = 7.40 - 7.22 (5H, m, Ph), 6.63 (1H, dd, *J* = 15.8 Hz, H-5), 6.17 (1H, dd, *J* = 15.9, 7.0 Hz, H-4), 5.88 (1H, s, NH), 4.43 (1H, t, *J* = 7.3 Hz, H-3), 3.49-3.35 (2H, m, N-CH_2), 3.09 (1H, dt, *J* = 13.9, 6.1 Hz), 3.04 - 2.96 (1H, m) (S-CH_2), 2.89 (1H, p, *J* = 7.2 Hz, H-2), 1.90 (3H, s, CO-CH_3), 1.21 (3H, d, *J* = 7.1 Hz, 2-CH_3);

^{13}C-NMR (100 MHz, $CDCl_3$): δ = 203.2 (C-1), 170.5 (N-CO), 136.1 (C-1′), 132.4 (C-5), 129.1 (C-4), 128.7 (C-3′/5′), 128.0 (C-4′), 126.5 (C-2′/6′), 75.1 (C-3), 54.2 (C-2), 39.3 (N-CH_2), 28.7 (S-CH_2), 23.1 (CO*C*H_3), 14.9 (2-CH_3);

ESI-MS (*m/z*): 308.3 [M + H]+ , 330.3 $[M + Na]^+$; 306.2 $[M - H]^-$, 352.2 $[M + HCOO]^-$.

HRESIMS (*m/z*): 330.1143 $[M + Na]^+$ (calc. for $C_{16}H_{21}NO_3SNa$, 330.1140).

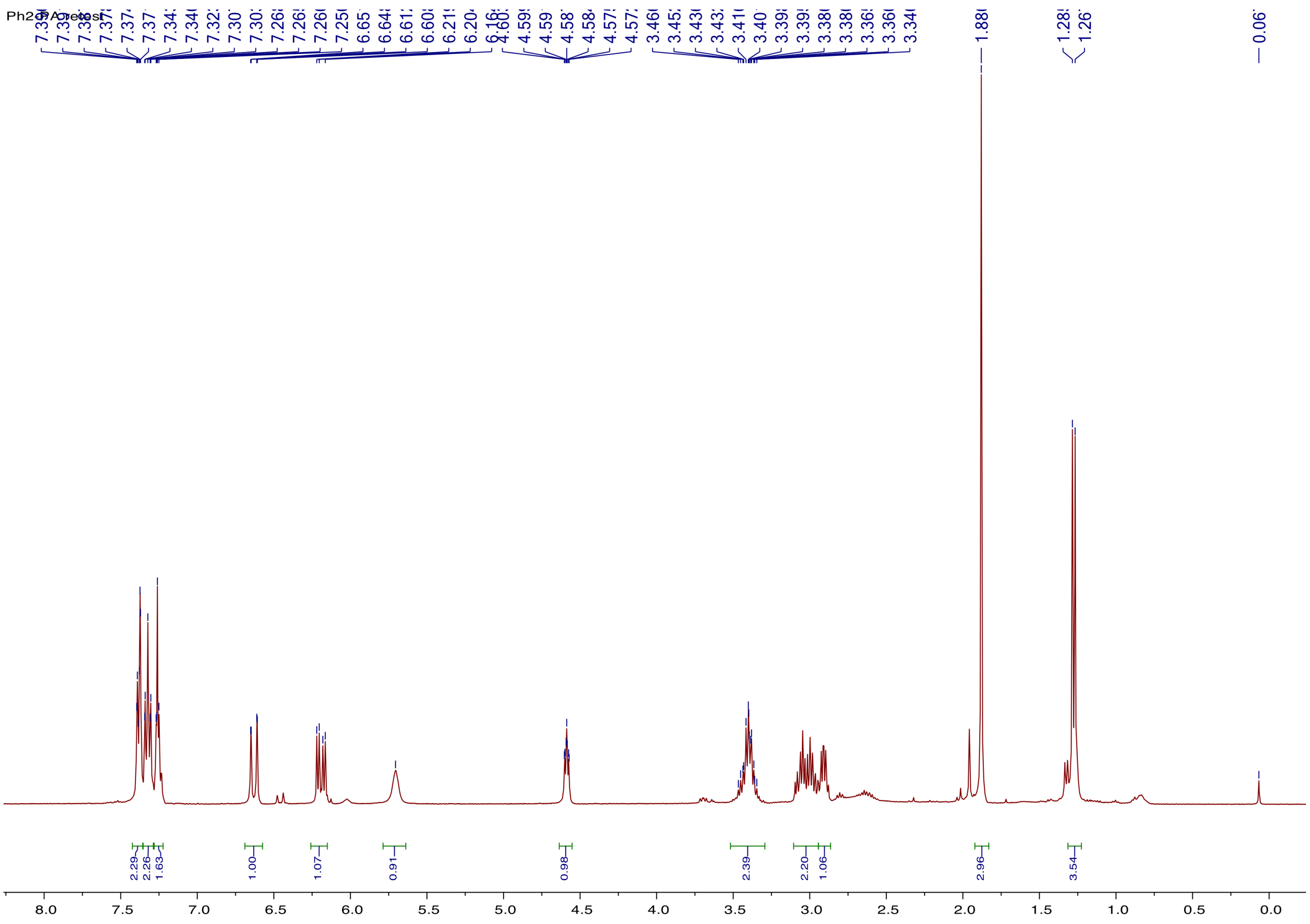

Figure S3.3.6C ^{1}H NMR of (±)-*syn*-**13b** in $CDCl_3$.

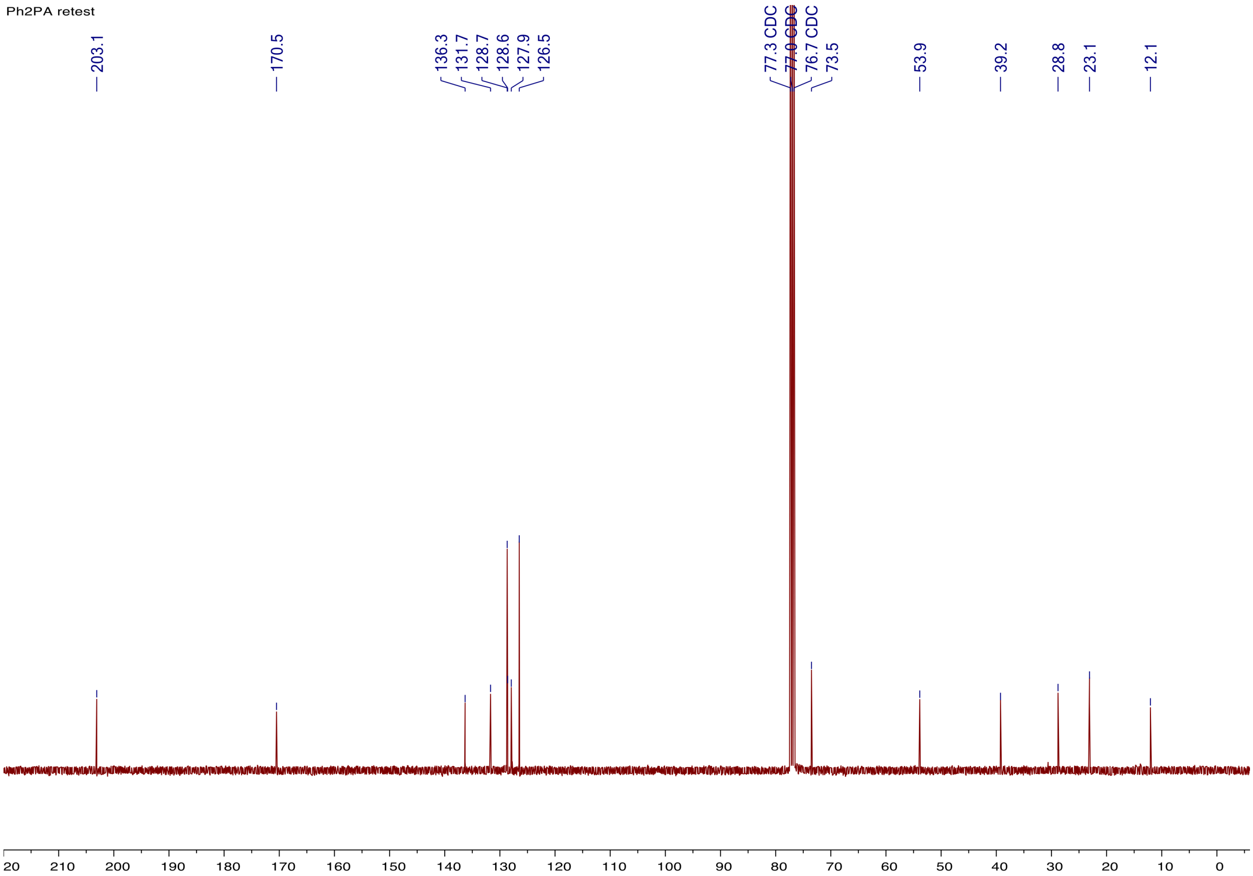

Figure S3.3.6D ^{13}C NMR of (±)-*syn*-**13b** in $CDCl_3$.

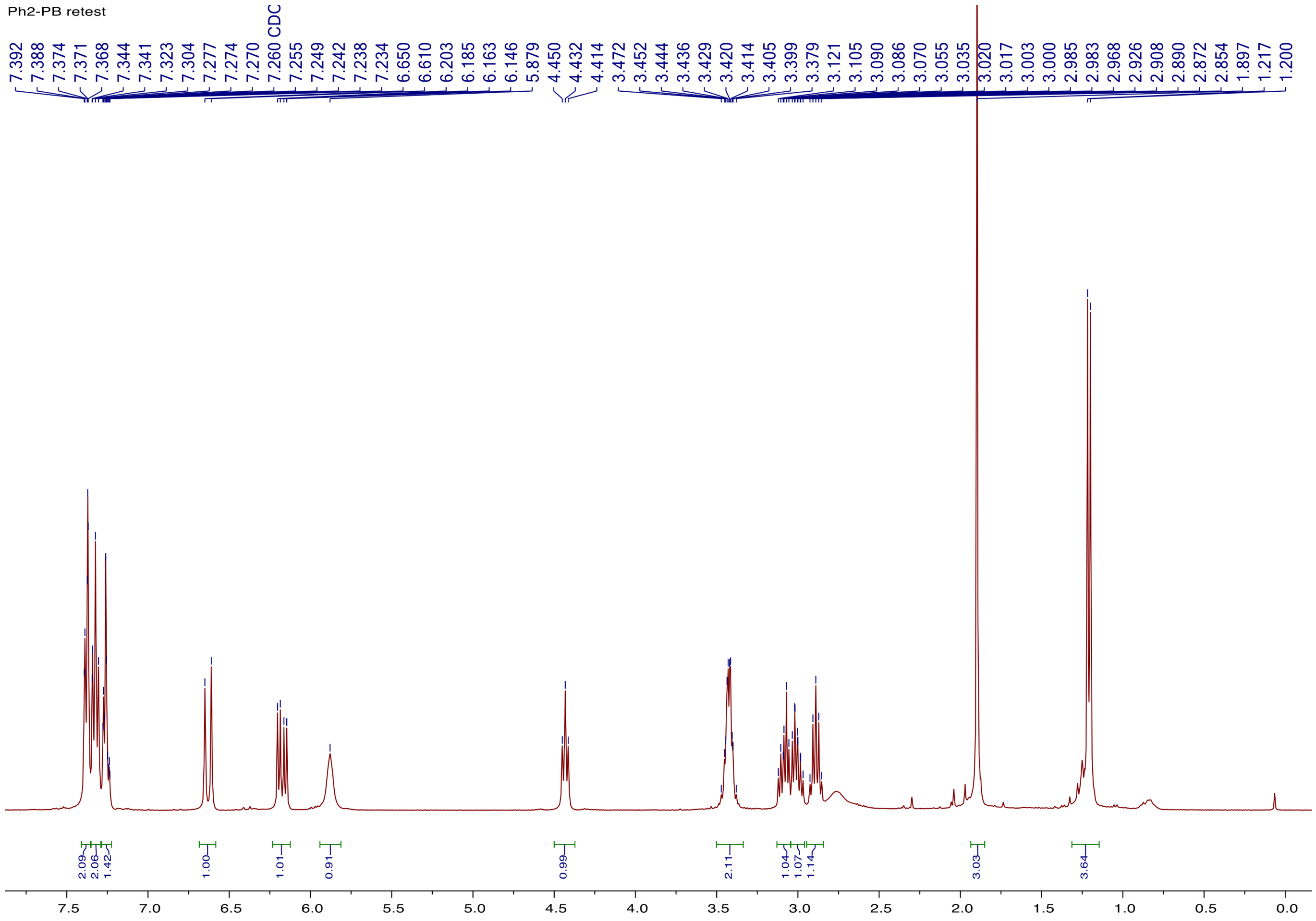

Figure S3.3.6C ^{1}H NMR of (±)-*anti*-**13b** in $CDCl_3$.

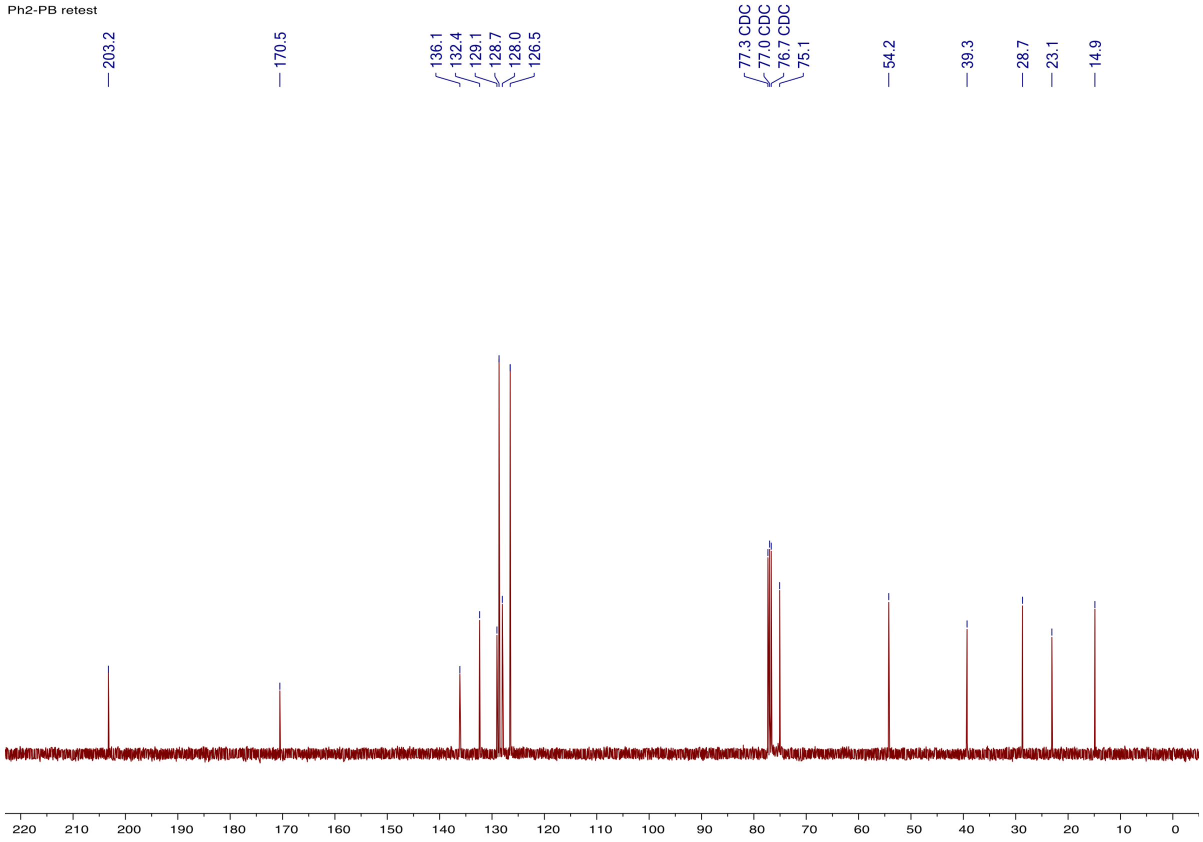

Figure S3.3.6D ^{13}C NMR of (±)-*anti*-**13b** in $CDCl_3$.

R-4-Benzyl-3-((2*R*,3*S*,*E*)-3-hydroxy-2-methyl-5-phenylpent-4-enoyl)oxazolidin-2-one 39b[20]

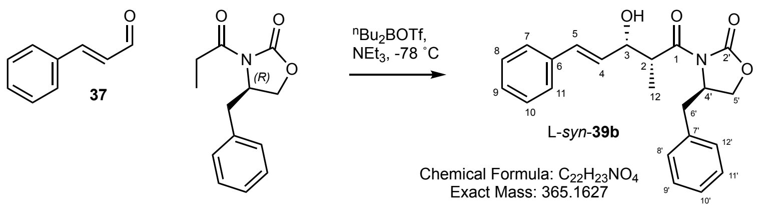

The procedure was followed as described by Evans et al. and slightly adapted. (*R*)-4-benzyl-3-propionyloxazolidin-2-one (1.0 eq., 200 mg, 0.86 mmol) was dissolved in CH_2Cl_2 (2.5 mL) and cooled to 0 °C. To the cooled solution $^nBu_2BOTf$ (1M in THF, 1.1 eq., 0.99 mL, 0.99 mmol) followed by NEt_3 (1.5 eq., 0.18 mL, 1.29 mmol) was added. The solution was allowed to stir for 5 min before it was cooled to -78 °C. After additional 15 min stirring, *trans*-cinnamaldehyde (1.1 eq., 0.13 mL, 0.99 mmol) was added dropwise. The reaction mixture was stirred at - 78 °C for 60 min and allowed to warm to 0 °C over 20 min. After additional 30 min at 0 °C the reaction was slowly quenched by the addition of NaOAc (1M in MeOH:H_2O 9:1, 2.7 mL) followed by 30% H_2O_2 (0.4 mL). The phases were separated, and the aqueous phase was extracted with CH_2Cl_2 (3 × 5 mL). The combined organic phases were washed with sat. $NaHCO_3$ and Brine, dried over $MgSO_4$, filtered and concentrated in *vacuo*. The crude residue was purified by flash chromatography (hexane : ethyl acetate 5:2) to give the desired aldol product **39b**[20] (282 mg, 0.77 mmol, 90%).

$[\alpha]_D^{20}$ (c 0.2 in $CHCl_3$) = -75.7 (lit. **$[\alpha]_D^{20}$** (c 1.08 in CH_2Cl_2) = -83.3)[21]
^{1}H-NMR (400 MHz, $CDCl_3$): δ = 7.41 - 7.20 (10H, m, ArH), 6.68 (1H, dd, *J* = 16.0, 1.5 Hz, H-5), 6.22 (1H, dd, *J* = 16.0, 5.8 Hz, H-4), 4.73 - 4.66 (2H, m, H-3 & 4'), 4.19 - 4.14 (2H, m, H-5'), 3.99 (1H, qd, *J* = 7.0, 3.8 Hz, H-2), 3.26 (1H, dd *J* = 13.4, 3.4 Hz, H-6'), 2.96 (1H, d, *J* = 3.0 Hz, OH), 2.80 (1H, dd, *J* = 13.4, 9.4 Hz, H-6'), 1.31 (3H, d, *J* = 7.0 Hz, H-12) ppm.
^{13}C-NMR (100 MHz, $CDCl_3$): δ = 176.7 (C-1), 153.3 (C-2'), 136.7 (C-7'), 135.1 (C-6), 131.6 (C-9 & C-10'), 129.6 (C-8/10), 129.1 (C-7/11), 128.7 (C-9'/11'), 127.9 (C-5), 127.6 (C-4), 126.7 (C-8'/12'), 72.9 (C-3), 66.4 (C-5'), 55.3 (C-4'), 43.1 (C-2), 38.0 (C-6'), 11.5 (C-12) ppm.
ESI-MS (*m/z*): 388.3 $[M + Na]^+$, 348.2 $[M - H_2O + H]^+$.

S-(2-Acetamidoethyl) (2*R*,3*S*,*E*)-3-hydroxy-2-methyl-5-phenylpent-4-enethioate L-*syn*-13b

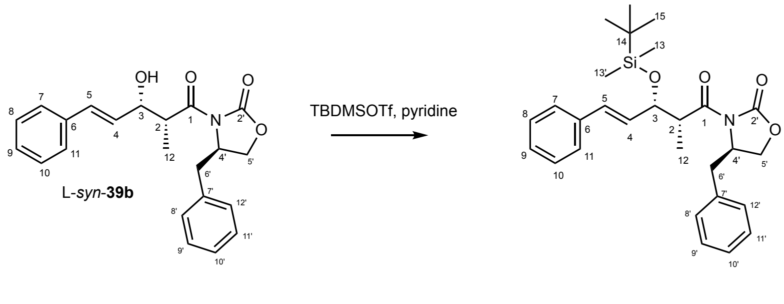

The aldol product **39b** (1.0 eq., 246 mg, 0.71 mmol) was dissolved in CH_2Cl_2 (3 mL) and cooled to 0 °C. To the cooled reaction mixture were added DMAP (0.1 eq., 8.7 mg, 0.07 mmol), Imidazole (4.0 eq., 193 mg, 2.8 mmol) and TBDMS-Cl (2.0 eq. 214 mg, 1.42 mmol) sequentially. After complete addition the reaction mixture was allowed warm to room temperature and stirred until completion. The solution was quenched by the addition of H_2O (3 mL). The phases were separated, and the aqueous phase was extracted with CH_2Cl_2 (3 × 4 mL). The combined organic phases were washed with Brine, dried over $MgSO_4$, filtered and concentrated in *vacuo*. The crude residue was purified by flash chromatography (petroleum ether : ethyl acetate 1:20 to 1:10) to give the protected intermediate (275 mg, 0.57 mmol, 81%).

^{1}H-NMR (400 MHz, $CDCl_3$): δ = 7.37 - 7.14 (10H, m, ArH), 6.49 (1H, d, *J* = 16.0 Hz, H-5), 6.22 (1H, dd, *J* = 16.0, 7.2 Hz, H-4), 4.56 - 4.52 (1H, m, H-3), 4.46 (t, *J* = 6.9 Hz, H-4'), 4.13 - 4.06 (2H, m, H-5'), 3.91 (1H, t, *J* = 8.3 Hz, H-2), 3.25 (1H, dd, *J* = 13.3, 3.3 Hz, H-6'), 2.76 (1H, dd, *J* = 13.4, 9.7 Hz, H-6'), 1.27 (3H, d, *J* = 6.8 Hz, H-12), 0.90 (9H, s, H-15), 0.05 (3H, s, H-13), 0.02 (3H, s, H-13') ppm.

^{13}C-NMR (100 MHz, $CDCl_3$): δ = 174.9 (C-1), 153.4 (C-2'), 136.8 (C-7'), 135.5 (C-6), 130.9 (C-5), 129.6 (C-8/10), 129.1 (C-7/11), 128.8 (C-9'/11'), 127.8 (C-10'), 127.5 (C-4), 126.6 (C-8'/12'), 75.6 (C-3), 66.1 (C-5'), 55.8 (C-4'), 44.7 (C-6'), 38.0 (C-2), 25.9 (C-15), 24.0 (C-14), 13.0 (C-12), -4.0 (C-13), -4.9 (C-13') ppm.

ESI-MS (*m/z*): 502.4 $[M + Na]^+$.

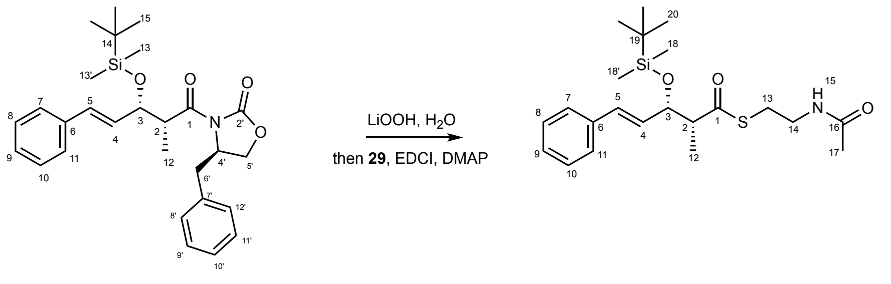

The 3-*O*-TBDMS-protected aldol product as prepared above (1.0 eq., 100 mg, 0.21 mmol) was dissolved in THF (2 mL) and cooled to 0 °C. To the cooled solution 30% H_2O_2 (7.0 eq., 0.11 mL, 1.46 mmol) was added followed by the slow addition of $LiOH{\cdot}H_2O$ (2.5 eq., 21.8 mg, 0.52 mmol) dissolved in H_2O (0.6 mL). The mixture was allowed to warm to RT and stirred until completion. The solution was quenched with sat. $Na_2S_2O_3$ (2 mL) and concentrated in *vacuo*. The resulting slurry was carefully acidified with HCl (1M) and extracted with EtOAc (3x2 mL). The combined organic phases were dried over Na_2SO_4, filtered and concentrated in *vacuo*. The crude residue was purified by flash chromatography (petroleum ether : ethyl acetate 10:1 + 1% formic acid) to give the free acid that was dissolved in CH_2Cl_2 (2 mL) and cooled to 0 °C. After cooling EDCl (1.1 eq, 50 mg, 0.26 mmol) and DMAP (0.2 eq., 7 mg, 0.06 mmol) were added. The solution was allowed to stir for 5 min before HSNAC **29** (1.1 eq., 32 mg, 0.26 mmol) was added. After complete addition the reaction mixture was allowed to warm to room temperature and stirred overnight. After completion the reaction was quenched by the addition of H_2O (3 mL). The phases were separated, and the aqueous phase was extracted with CH_2Cl_2 (3 × 3 mL). The combined organic phases were dried over $MgSO_4$, filtered and concentrated in *vacuo*. The crude residue was purified by flash chromatography (petroleum ether : ethyl acetate 1:1) to give the desired product 3-*O*-protected L-*syn*-triketide as a colourless oil (55 mg, 0.13 mmol, 63%).

$[\alpha]_D^{20}$ (c = 1.0 $CHCl_3$) = - 3.5

^{1}H-NMR (400 MHz, $CDCl_3$): δ = 7.38 - 7.23 (5H, m, ArH), 6.45 (1H, d, *J* = 15.9 Hz, H-5), 6.16 (1H, dd, *J* = 15.9, 7.4 Hz, H-4), 5.56 (1H, brs, H-14), 4.42 (1H, ddd, *J* = 7.4, 6.7, 1.0 Hz, H-3), 3.42 - 3.36 (1H, m, H-2), 3.26 - 3.13 (1H, m, H-12), 3.05 - 2.99 (1H,m, H-12), 2.93 - 2.81 (2H, m, H-13), 1.79 (3H, s, H-16), 1.24 (3H, d, *J* = 6.9 Hz, H-17), 0.90 (9H, s, H-20), 0.07 (3H, s, H-13), 0.02 (3H, s, H-13') ppm.

^{13}C-NMR (100 MHz, $CDCl_3$): δ = 202.2 (C-1), 170.4 (C-15), 136.6 (C-6), 131.1 (C-5), 130.7 (C-9), 128.9 (C-8/10), 128.0 (C-4), 126.6 (C-7/11), 75.7 (C-3), 55.9 (C-2), 39.5 (C-13), 28.9 (C-12), 25.9 (C-20), 23.2 (C-16), 18.3 (C-19), 13.4 (C-17), -3.9 (C-16), -4.8 (C-16') ppm.

ESI-MS (*m/z*): 444.3 $[M + Na]^+$

HRESIMS (*m/z*): calculated for $C_{22}H_{35}NO_3SSiNa$ $[M + Na]^+$: 444.1999 found 444.1985.

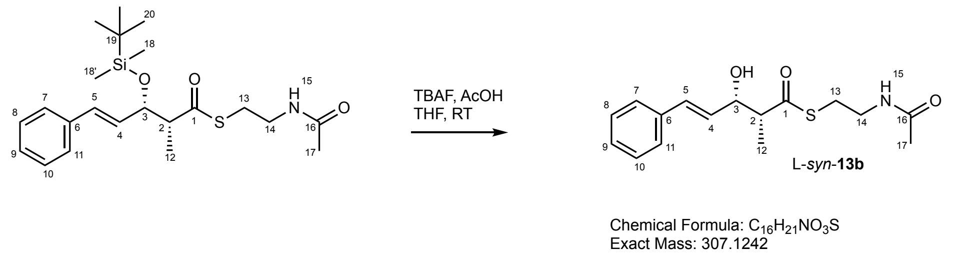

The 3-*O*-TBDMS-protected SNAC triketide as prepared above (1.0 eq., 14 mg, 0.033 mmol) was dissolved in THF (1 mL) and cooled to 0 °C. In a separate vial were added TBAF (1M in THF, 0.3 mL) and glacial acetic acid (30 µL) and mixed. This mixture was then transferred to the reaction mixture and added dropwise. The reaction was allowed to warm to room temperature and stirred for 2 days. The reaction progress was checked by LCMS. When there was starting material left another equivalent of TBAF+ AcOH was added, and the mixture was stirred for additional 3-4 days. The reaction was quenched with NH_4Cl (2 mL) and extracted with Et_2O (3 × 2 mL). The combined organic layers were washed with NH_4Cl (2 × 2 mL), dried over $MgSO_4$, filtered and concentrated in *vacuo* to give the desired L-*syn*-**13b** (6.4 mg, 0.021 mmol, 63%) as a colourless opaque oil.

dr. 96:4 (*syn : anti*)
[α]$_D^{25}$ (c = 0.30 $CHCl_3$) = -24.1
^{1}H-NMR (400 MHz, $CDCl_3$): δ = 7.40 - 7.23 (5H, m, ArH), 6.63 (1H, dd, *J* = 15.9, 1.4 Hz, H-5), 6.19 (1H, dd, *J* = 15.9, 6.3 Hz, H-4), 5.74 (1H, brs, H-14), 4.59 (1H, ddd, *J* = 6.3, 4.7, 1.4 Hz, H-3), 3.45 - 3.23 (2H, m, H-13), 3.10 - 2.95 (2H, m, H-12), 2.91 (1H, qd, *J* = 7.0, 4.6 Hz, H-2) 1.89 (3H, s, H-16), 1.27 (3H, d, *J* = 7.0 Hz, H-17) ppm.
^{13}C-NMR (100 MHz, $CDCl_3$): δ = 203.3 (C-1), 170.7 (C-15), 136.5 (C-6), 131.8 (C-5), 128.8 (C-8/10), 128.8 (C-9), 128.1 (C-4), 126.7 (C-7/11), 73.6 (C-3), 54.0 (C-2), 39.4 (C-13), 29.0 (C-12), 23.3 (C-16), 12.2 (C-17) ppm.
ESI-MS (*m/z*): 330.2 $[M + Na]^+$, 290.2 $[M - H_2O + H]^+$.
HRESIMS (*m/z*): calculated for $C_{16}H_{21}NO_3SNa$ $[M + Na]^+$: 330.1134 found 330.1129.

S-(2-Acetamidoethyl) (2*S*,3*R*,*E*)-3-hydroxy-2-methyl-5-phenylpent-4-enethioate D-*syn*-13b[22]

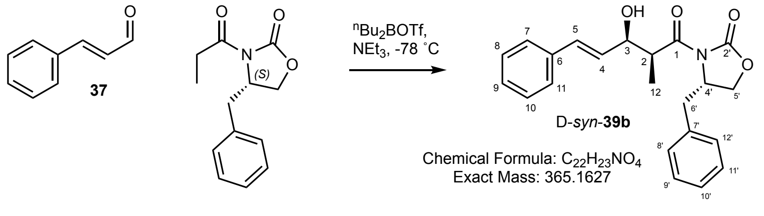

The procedure was followed as described for L-*syn*-**39b**.

[α]$_D^{20}$ (c = 0.9 in $CHCl_3$) = + 73.5 (lit. **[α]$_D^{20}$** (c = 2.0 in $CHCl_3$) = +78.6)[23]
^{1}H-NMR (400 MHz, $CDCl_3$): δ = identical to L-*syn*-**39b**.
^{13}C-NMR (100 MHz, $CDCl_3$): δ = identical to L-*syn*-**39b**.

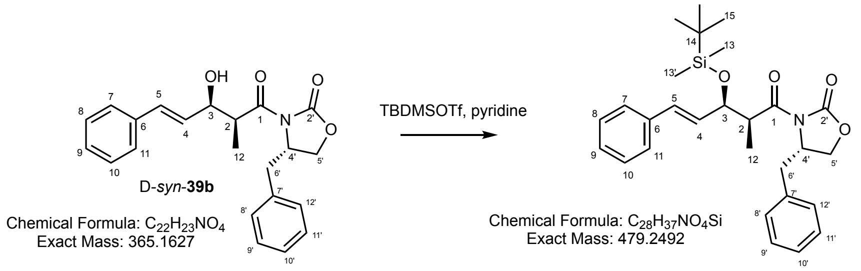

The procedure was followed as described for the enantiomer.

^{1}H-NMR (400 MHz, $CDCl_3$): δ = identical to enantiomer.

^{13}C-NMR (100 MHz, $CDCl_3$): δ = identical to enantiomer.

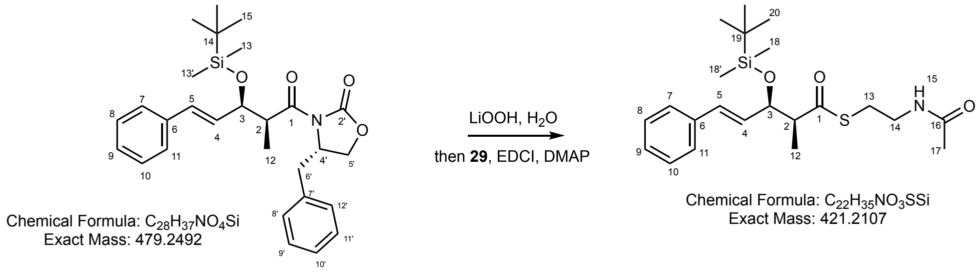

The procedure was followed as described for the enantiomer.

$[\alpha]_D^{20}$ (c =0.9 $CHCl_3$) = + 4.3

^{1}H-NMR (400 MHz, $CDCl_3$): δ = identical as the enantiomer.

^{13}C-NMR (100 MHz, $CDCl_3$): δ = identical as the enantiomer.

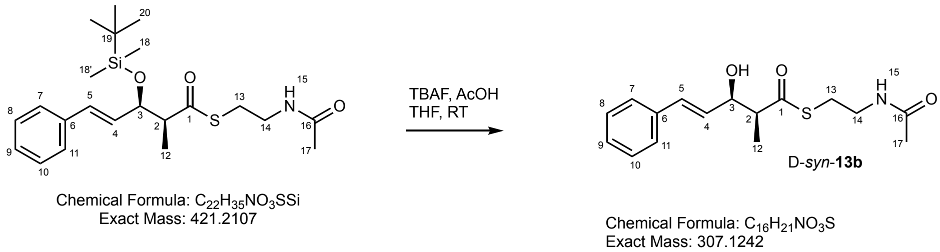

The procedure was followed as described for L-*syn*-**13b**.

dr. 93:7

$[\alpha]_D^{20}$ (c = 0.35 in $CHCl_3$) = + 19.9

^{1}H-NMR (400 MHz, $CDCl_3$): δ = identical to L-*syn* **13b**.

^{13}C-NMR (100 MHz, $CDCl_3$): δ = identical to L-*syn* **13b**.

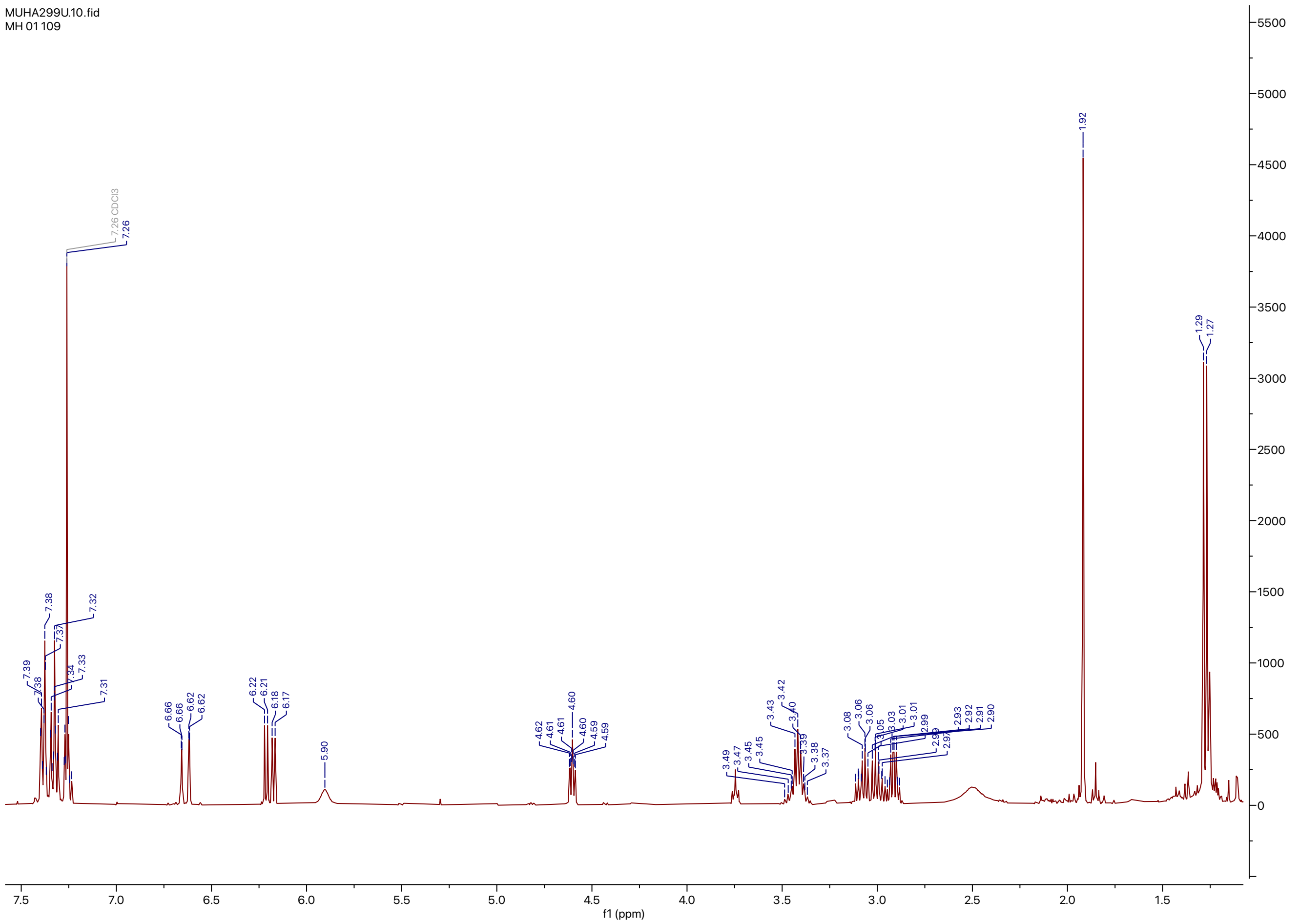

Figure S3.3.6E ^{1}H NMR of L-*syn*-**13b** in $CDCl_3$.

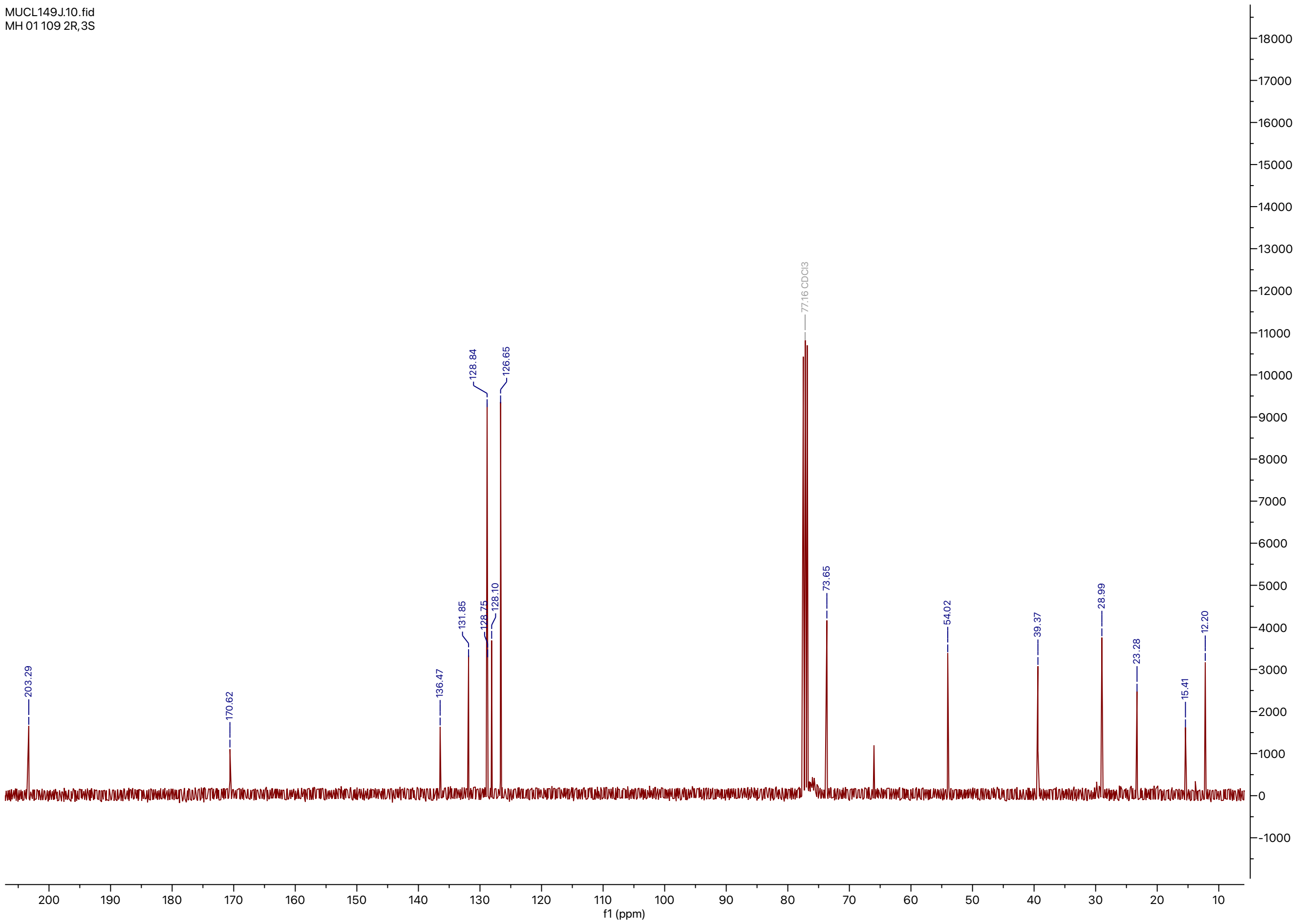

Figure S3.3.6F ^{13}C NMR of L-*syn*-**13b** in $CDCl_3$.

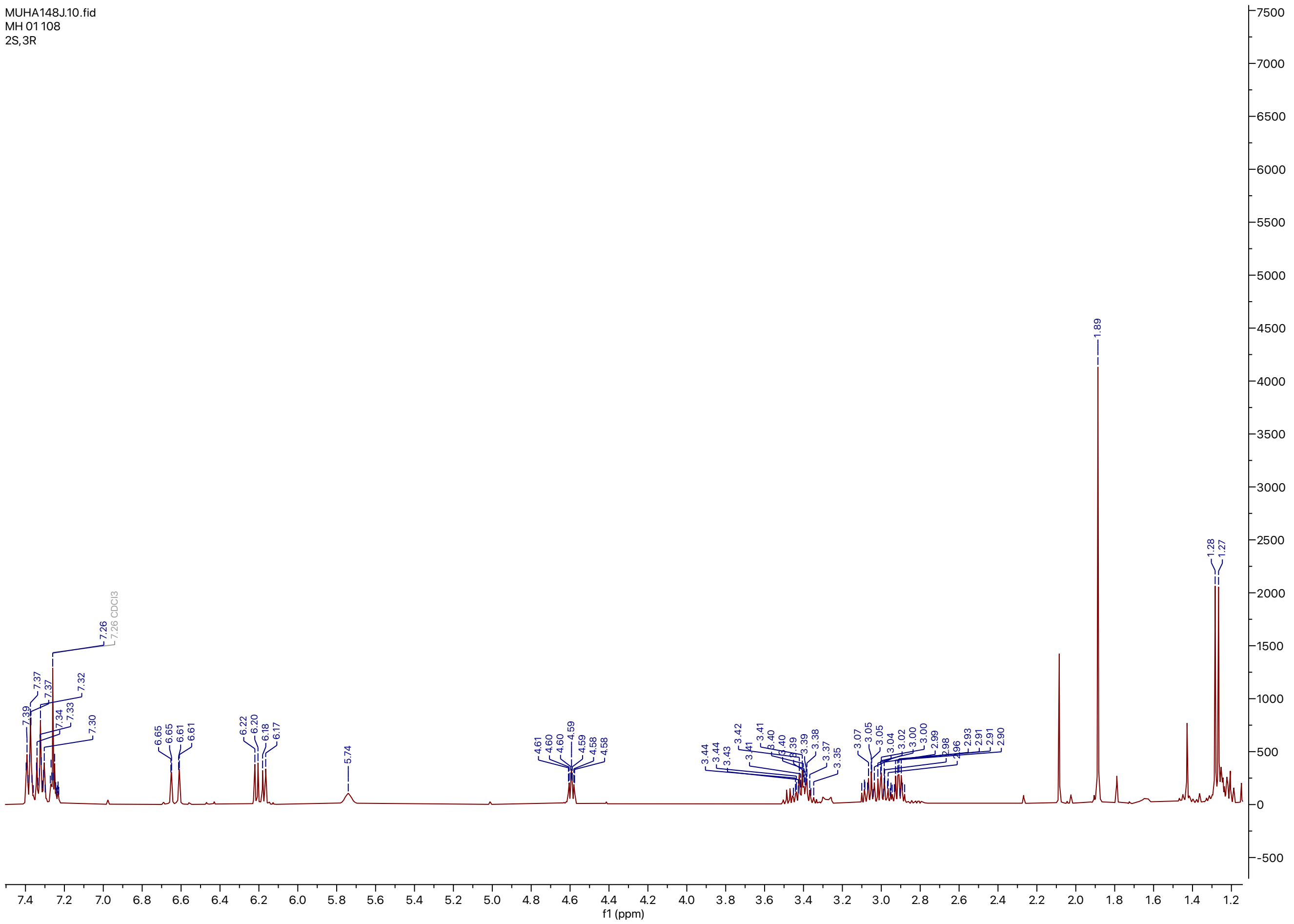

Figure S3.3.6G ^{1}H NMR of D-*syn*-**13b** in $CDCl_3$.

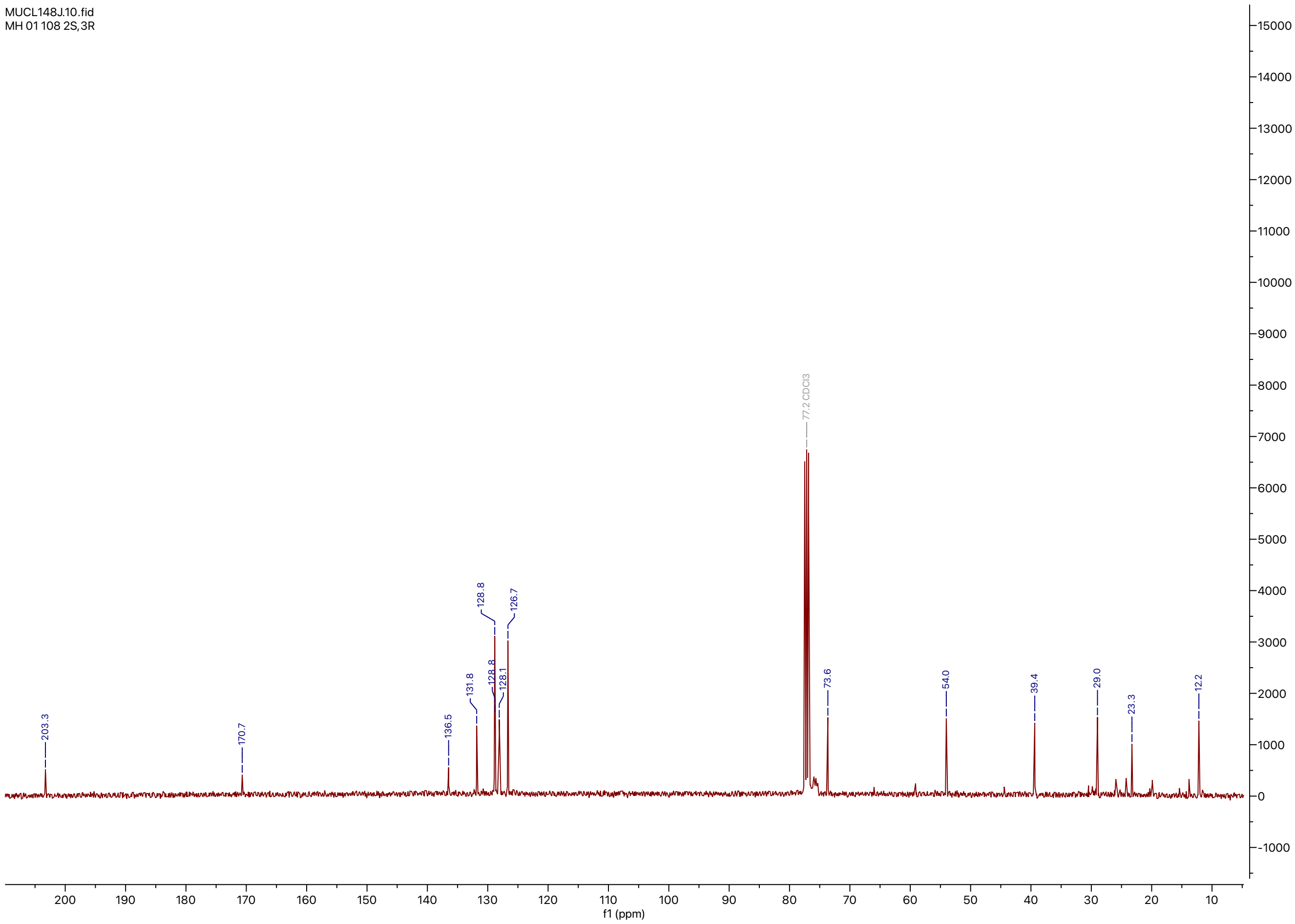

Figure S3.3.6H ^{13}C NMR of D-*syn*-**13b** in $CDCl_3$.

R-4-Benzyl-3-((2*S*,3*S*,*E*)-3-hydroxy-2-methyl-5-phenylpent-4-enoyl)oxazolidin-2-one 39b

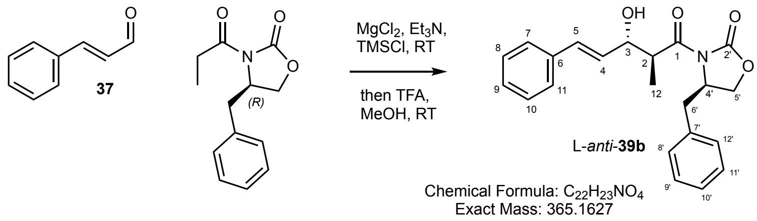

The procedure was followed as described by Evans et al. and slightly adapted. (*R*)-4-benzyl-3-propionyloxazolidin-2-one (1.0 eq., 200 mg, 0.86 mmol) and $MgCl_2$ (0.1 eq., 8.2 mg, 0.09 mmol) were suspended in EtOAc (2 mL). Subsequently NEt_3 (2.0 eq. 0.24 mL, 1.72 mmol), *trans*-cinnamaldehyde (1.2 eq., 0.12 mL, 1.03 mmol) and $(CH_3)_3SiCl$ (1.5 eq., 0.16 mL, 1.29 mmol) were added. The resulting suspension was stirred at RT for 24 h and quenched with the addition of H_2O (3 mL). The phases were separated, and the aqueous phase was extracted with Et_2O (3 × 3 mL). The combined organic layers were dried over $MgSO_4$, filtered and concentrated in *vacuo*. The crude residue was dissolved in MeOH (5 mL) and TFA (3 drops) were added. After 30 min the solution was concentrated in *vacuo* to give a crude yellow oil. The crude residue was purified by flash chromatography (hexane : ethyl acetate 5:2) to give a colourless wax (267 mg, 0.73 mmol, 85%).

$[\alpha]_D^{20}$ (c = 0.8 in CH_2Cl_2) = -35.8 (lit. enantiomer $[\alpha]_D^{25}$ (c = 0.54 in CH_2Cl_2) = +37.1)[14]
^{1}H-NMR (400 MHz, $CDCl_3$): δ = 7.41 - 7.24 (7H, m, ArH), 7.20 - 7.17 (3H, m, ArH), 6.68 (1H, d, *J* = 15.9 Hz, H-5), 6.29 (1H, dd, *J* = 15.9, 6.7 Hz, H-4), 4.71 (1H, ddt, *J* = 9.5, 7.5, 3.2 Hz, H-4'), 4.47 (1H, td, *J* = 7.1, 1.3 Hz, H-3), 4.23 - 4.08 (3H, m, H-2 & H-5'), 3.28 (1H, dd, 13.5, 3.4 Hz, H-6'), 2.71 (1H, dd, *J* = 13.5, 9.5 Hz, H-6'), 1.27 (3H, d, *J* = 6.9 Hz, H-12) ppm.
^{13}C-NMR (100 MHz, $CDCl_3$): δ = 176.5 (C-1), 153.7 (C-2'), 136.4 (C-7'), 135.3 (C-6), 132.5 (C-5), 129.6 (C-9), 129.5 (C-8/10), 129.1 (C-9'/11'), 128.8 (C-7/11), 128.1 (C-10'), 127.5 (C-4), 126.8 (C-8'/12'), 75.9 (C-3), 66.2 (C-5'), 55.6 (C-4'), 43.3 (C-2), 37.9 (C-6'), 14.8 (C-12) ppm.
ESI-MS (*m/z*): 348.2 $[M - H_2O + H]^+$, 388.2 $[M + Na]^+$.

S-(2-Acetamidoethyl) (2*S*,3*S*,*E*)-3-hydroxy-2-methyl-5-phenylpent-4-enethioate L-*anti*-13b

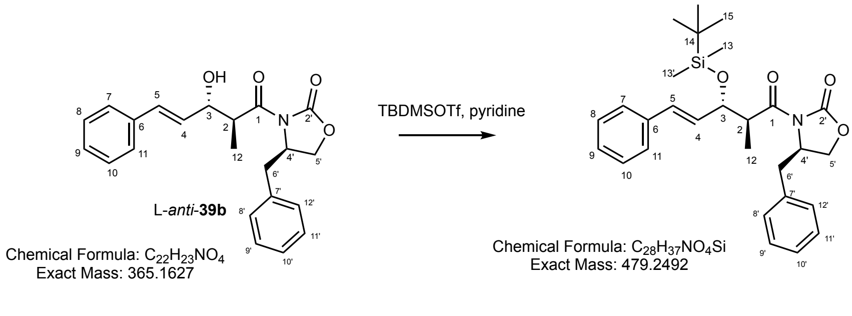

The aldol product L-*anti*-**39b** (1.0 eq., 130 mg, 0.36 mmol) was dissolved in CH_2Cl_2 (2 mL) and cooled to 0 °C. To the cooled solution pyridine (1.05 eq, 30 µL, 0.37 mmol) was added and allowed to stir for 10 min, before the addition of TBDMS-OTf (1.05 eq. 85 µL, 0.37 mmol). After complete addition, the reaction mixture was allowed to warm to room temperature and stirred until completion. The solution was quenched by the addition of H_2O (3 mL). The phases were separated, and the aqueous phase was extracted with CH_2Cl_2 (2 × 4 mL). The combined organic phases were washed with Brine, dried over $MgSO_4$, filtered and concentrated in *vacuo*. The crude residue was purified by flash chromatography (petroleum ether : ethyl acetate 1:20 to 1:10) to give the protected intermediate (133 mg, 0.28 mmol, 78%).

^{1}H-NMR (400 MHz, $CDCl_3$): δ = 7.40 - 7.22 (10H, m, ArH), 6.55 (1H, d, *J* = 15.9 Hz, H-5), 6.15 (1H, *J* = 15.9, 7.9 Hz, H-4), 4.73 - 4.67 (1H, m, H-4'), 4.67 - 4.62 (1H, m, H-3), 4.19 - 4.05 (3H, m, H-2 & H-5'), 3.40 (1H, dd, *J* = 13.3, 3.4 Hz, H-6'), 2.66 (1H, dd, *J* = 13.3, 10.1 Hz, H-6'), 1.11 (3H, d, *J* = 7.0 Hz, H-12), 0.88 (9H, s, H-15), 0.09 (3H, s, H-13), 0.06 (3H, s, H-13') ppm.
^{13}C-NMR (100 MHz, $CDCl_3$): δ = 175.4 (C-1), 153.3 (C-2'), 136.7 (C-6), 135.7 (C-7'), 132.3 (C-5), 130.5 (C-9) 129.5 (C8/10), 129.1 (C-9'/11'), 128.8 (C-7/11), 127.9 (C-10'), 127.4 (C-4), 126.7 (C8'/12'), 76.1 (C-3), 66.0 (C-5'), 55.5 (C-4'), 44.8 (C-2), 38.5 (C-6'), 26.1 (C-15), 20.9 (C-14), 14.1 (C-12), -3.8 (C-13), -4.4 (C-13').
ESI-MS (*m/z*): 502.3 $[M + Na]^+$.

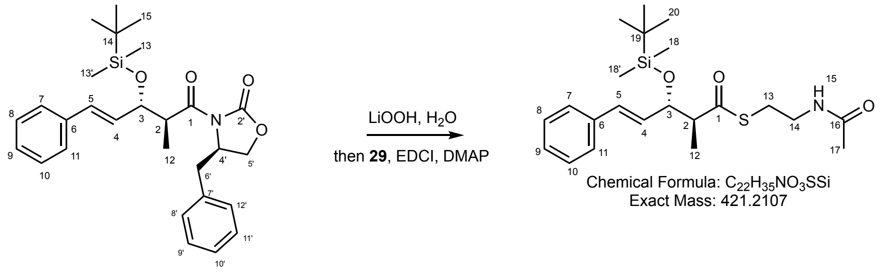

The 3-*O*-TBDMS-protected aldol product as prepared above (1.0 eq., 100 mg, 0.20 mmol) was dissolved in THF (2 mL) and cooled to 0 °C. To the cooled solution 30% H_2O_2 (7.0 eq., 0.11 mL, 1.46 mmol) was added followed by the slow addition of $LiOH·H_2O$ (2.5 eq., 22 mg, 0.52 mmol) dissolved in H_2O (0.6 mL). The mixture was allowed to warm to RT and stirred until completion. The solution was quenched with sat. $Na_2S_2O_3$ (2 mL) and concentrated in *vacuo*. The resulting slurry was carefully acidified with HCl (1M) and extracted with EtOAc (3 × 2 mL). The combined organic phases were dried over Na_2SO_4, filtered and concentrated in *vacuo*. The crude residue was purified by flash chromatography (petroleum ether : ethyl acetate 10:1 + 1% formic acid) to give the free acid that was dissolved in CH_2Cl_2 (2 mL) and cooled to 0 °C. After cooling EDCI (1.1 eq, 50 mg, 0.26 mmol) and DMAP (0.2 eq., 7 mg, 0.06 mmol) were added. The solution was allowed to stir for 5 min before HSNAC **29** (1.1 eq., 32 mg, 0.26 mmol) was added. After complete addition the reaction mixture was allowed to warm to room temperature and stirred overnight. After completion the reaction was quenched by the addition of H_2O (3 mL). The phases were separated, and the aqueous phase was extracted with CH_2Cl_2 (3 × 3 mL). The combined organic phases were dried over $MgSO_4$, filtered and concentrated in *vacuo*. The crude residue was purified by flash chromatography (petroleum ether : ethyl acetate 1:1) to give the desired product as a colourless oil (56 mg, 0.13 mmol, 66%).

$[\alpha]_D^{20}$ (c = 0.4 in $CHCl_3$) = +85.4
^{1}H-NMR (400 MHz, $CDCl_3$): δ = 7.39 - 7.24 (5H, m, ArH), 6.56 (1H, d, *J* = 15.9 Hz, H-5), 6.10 (1H, dd, *J* = 15.9, 7.6 Hz, H-4), 4.45 - 4.41 (1H, m, H-3), 2.67 (1H, p, *J* = 7.1 Hz, H-2), 1.17 (3H, d, *J* = 7.1 Hz, H-17), 0.89 (9H, s, H-20), 0.10 (3H, s, H-18), 0.05 (3H, s, H-18').
^{13}C-NMR (100 MHz, $CDCl_3$): δ = 179.2 (C-15), 136.4 (C-6), 132.3 (C-5), 129.5 (C-9), 128.6 (C-8/10), 128.0 (C-4), 126.6 (C-7/11), 76.0 (C-3), 47.0 (C-13), 25.7 (C-20), 18.1 (C-19), 13.5 (C-17), -4.0 (C-18), -5.1 (C-18') ppm.
ESI-MS (*m/z*): 444.4 $[M + Na]^+$.
HRESIMS (*m/z*): calculated for $C_{22}H_{35}NO_3SSiNa$ $[M + Na]^+$: 444.1999 found 444.1994

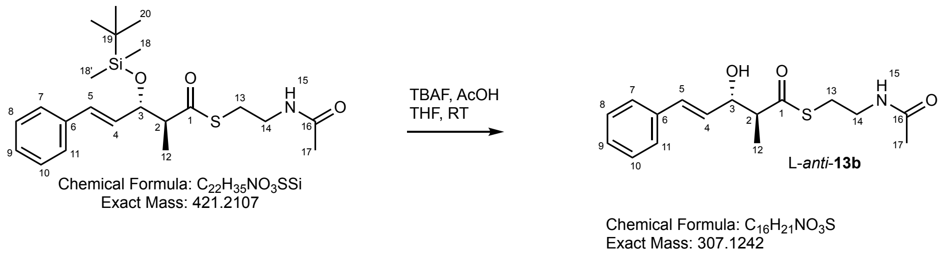

The procedure was followed as described for L-*syn*-**13b.**

dr. 99:1

[α]$_D$^{20} (c = 0.6 in $CHCl_3$) = +30.9

^{1}H-NMR (400 MHz, $CDCl_3$): δ = 7.40 - 7.24 (5H, m, ArH), 6.64 (1H, dd, *J* = 15.9, 1.1 Hz, H-5), 6.18 (1H, dd, *J* = 15.9, 7.0 Hz, H-4), 5.83 (1H, brs, H-14), 4.46 - 4.42 (1H, m, H-3), 3.46 - 3.40 (2H, m, H-13), 3.13 - 2.98 (2H, m, H-12), 2.90 (1H, p, *J* = 7.2 Hz, H-2), 1.90 (3H, s, H-16), 1.22 (3H, d, *J* = 7.1 Hz, H-17) ppm.

^{13}C-NMR (100 MHz, $CDCl_3$): δ = 203.3 (C-1), 170.7 (C-15), 136.5 (C-6), 131.8 (C-5), 128.8 (C-8/10), 128.8 (C-4), 128.1 (C-9), 126.6 (C-7/11), 73.6 (C-3), 54.0 (C-2), 39.4 (C-13), 29.0 (C-12), 23.3 (C-16), 12.2 (C-17) ppm.

ESI-MS (*m/z*): 290.2 [M - H_2O + H]$^+$, 330.2 [M + Na]$^+$.

HRESIMS (*m/z*): calculated for $C_{16}H_{21}NO_3SNa$ [M + Na]$^+$: 330.1134 found 330.1133

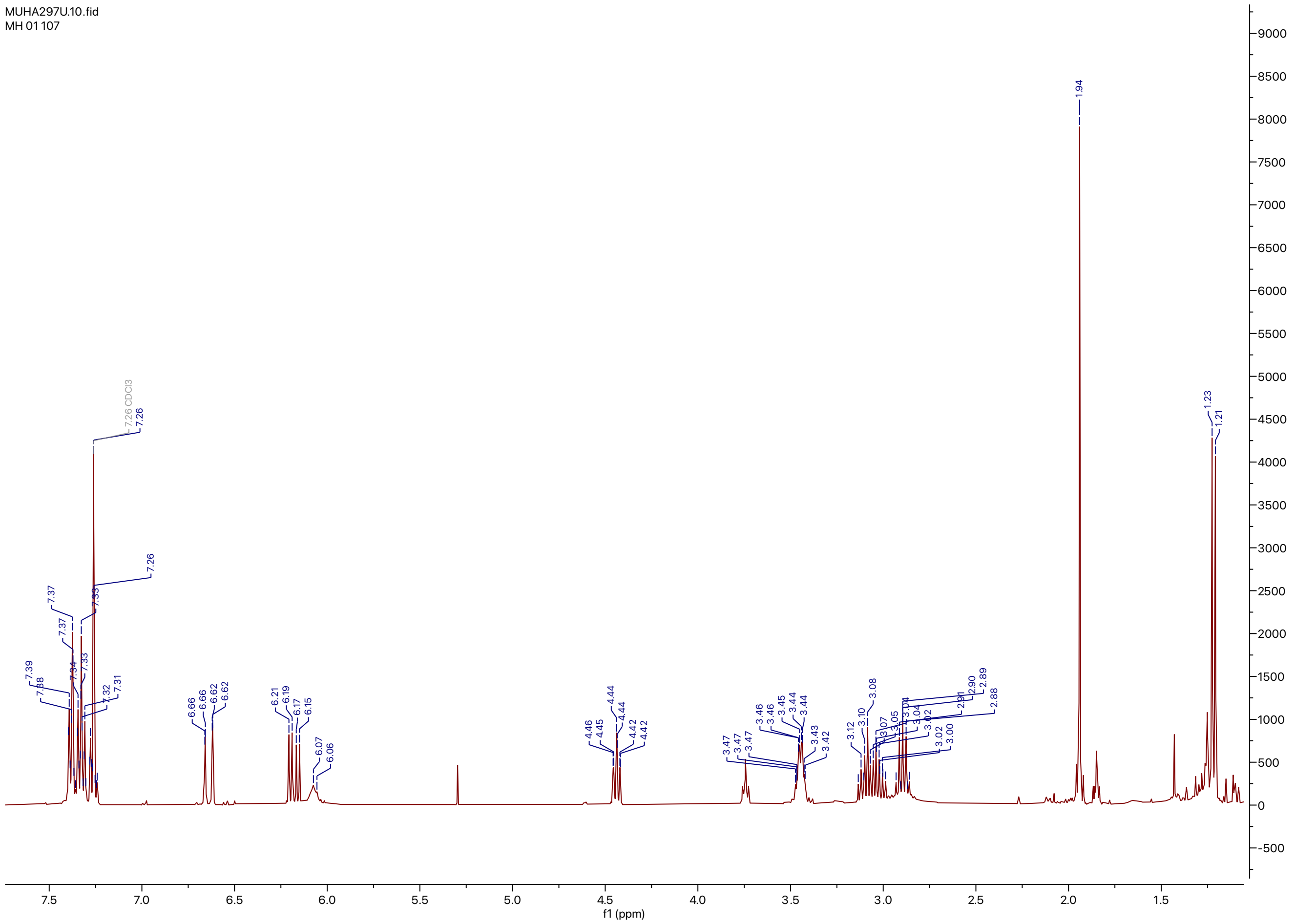

Figure S3.3.6l ^{1}H NMR of L-*anti*-**13b** in $CDCl_3$.

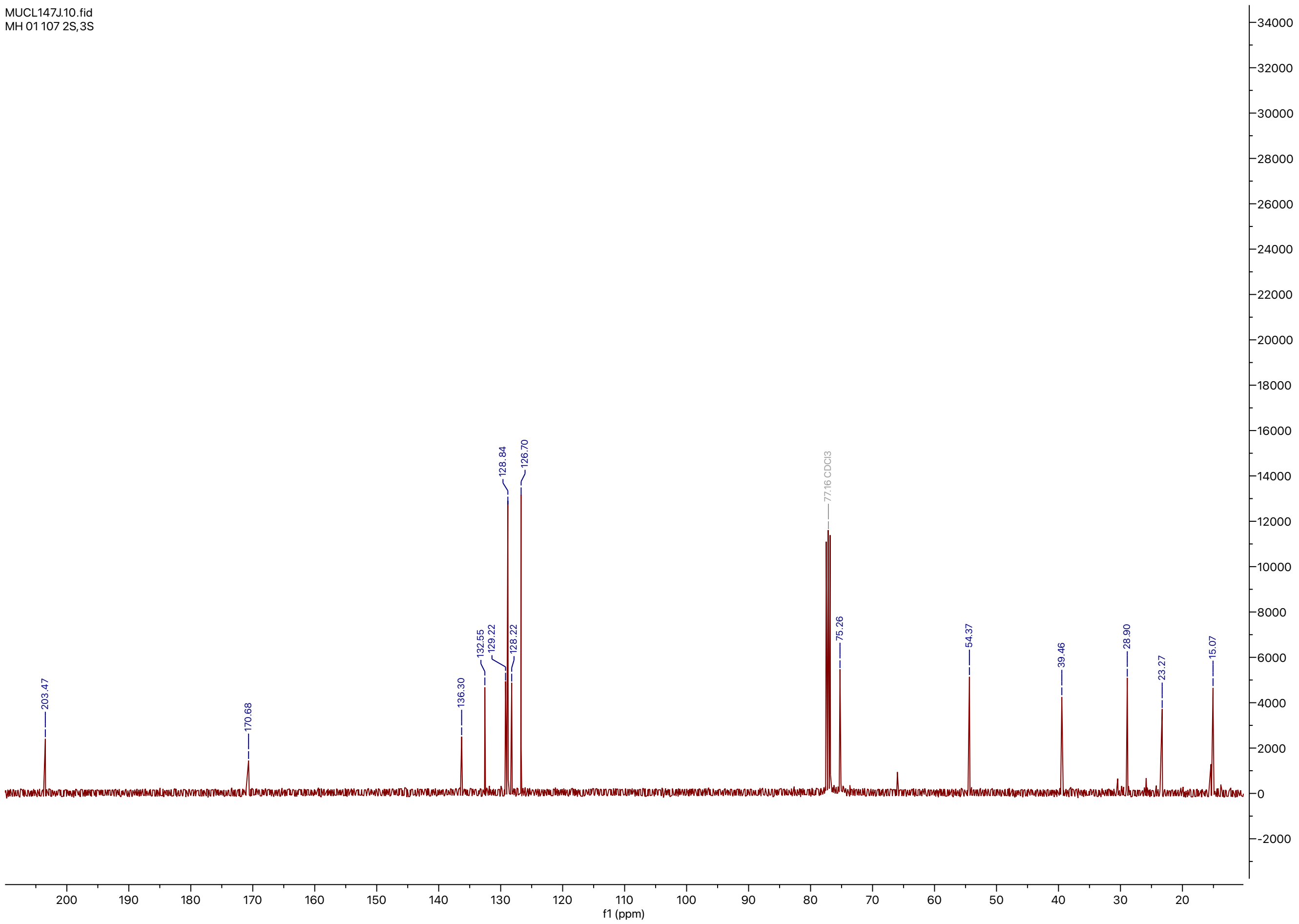

Figure S3.3.6J ^{13}C NMR of L-*anti*-**13b** in $CDCl_3$.

S-(2-Acetamidoethyl) (2*R*,3*R*,*E*)-3-hydroxy-2-methyl-5-phenylpent-4-enethioate D-*anti*-13b[14]

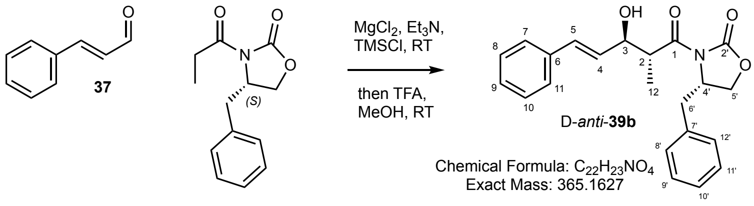

The procedure was followed as described for L-*anti*-**13b**.

[α]D20 (c = 0.23 in CH_2Cl_2) = +33.6 (lit. **[α]D25** (c = 0.54 in CH_2Cl_2) = +37.1)[14]

^{1}H-NMR (400 MHz, $CDCl_3$): δ = identical as described for L-*anti*-**13b**.

^{13}C-NMR (100 MHz, $CDCl_3$): δ = identical as described in L-*anti*-**13b**.

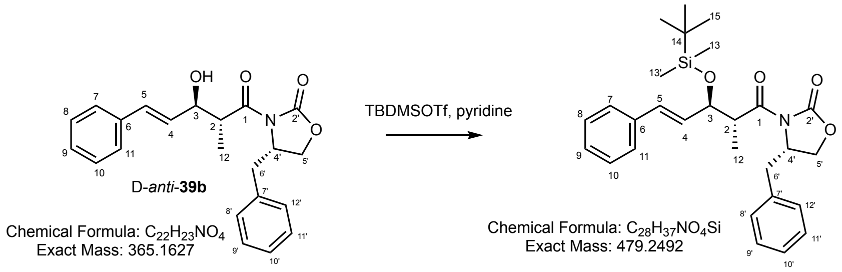

The procedure was followed as described in L-*anti*-**13b**.

^{1}H-NMR (400 MHz, $CDCl_3$): δ = identical to enantiomer.
^{13}C-NMR (100 MHz, $CDCl_3$): δ = identical to enantiomer.

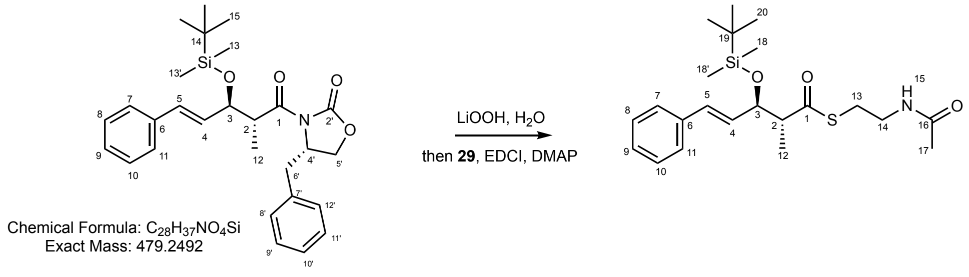

The procedure was followed as described in L-*anti*-**13b**.

[α]$_D^{20}$ (c = 0.44 in $CHCl_3$) = -89.6
^{1}H-NMR (400 MHz, $CDCl_3$): δ = identical to enantiomer.
^{13}C-NMR (100 MHz, $CDCl_3$): δ = identical to enantiomer.

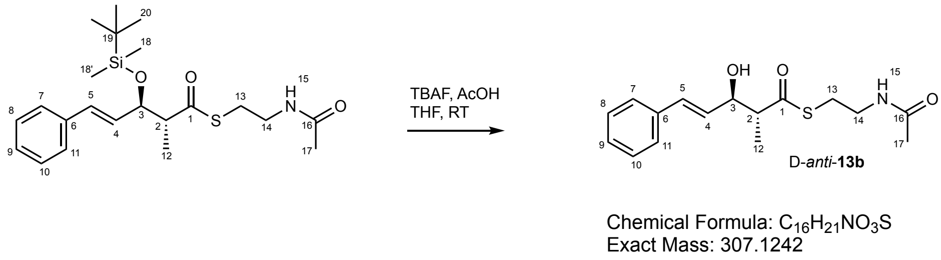

The procedure was followed as described for L-*syn*-**13b**.

dr. 96:4 (anti : syn) based on ^{1}H NMR
[α]$_D^{20}$ (c = 0.4 in $CHCl_3$) = -33.5
^{1}H-NMR (400 MHz, $CDCl_3$): δ = identical to L-*anti*-**13b**.
^{13}C-NMR (100 MHz, $CDCl_3$): δ = identical to L-*anti*-**13b**.

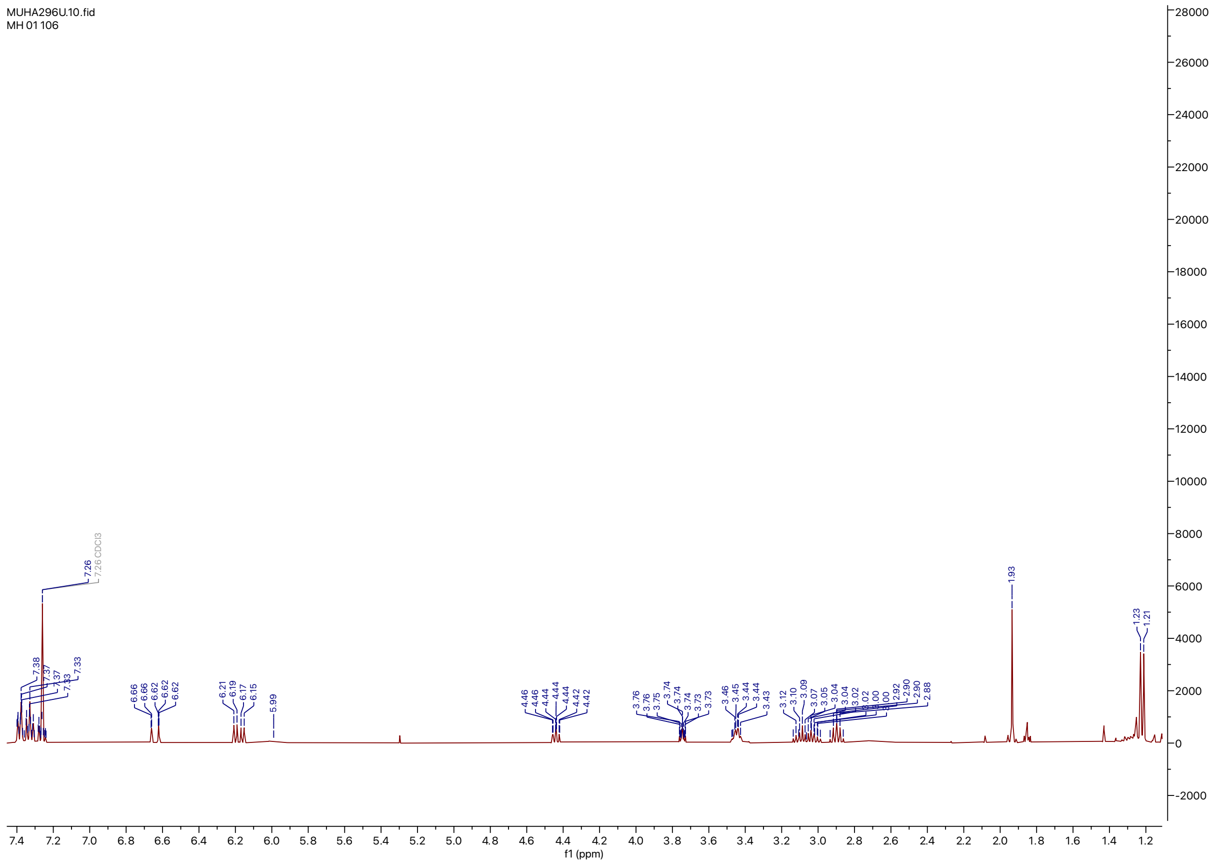

Figure S3.3.6K ^{1}H NMR of D-*anti*-**13b** in $CDCl_3$.

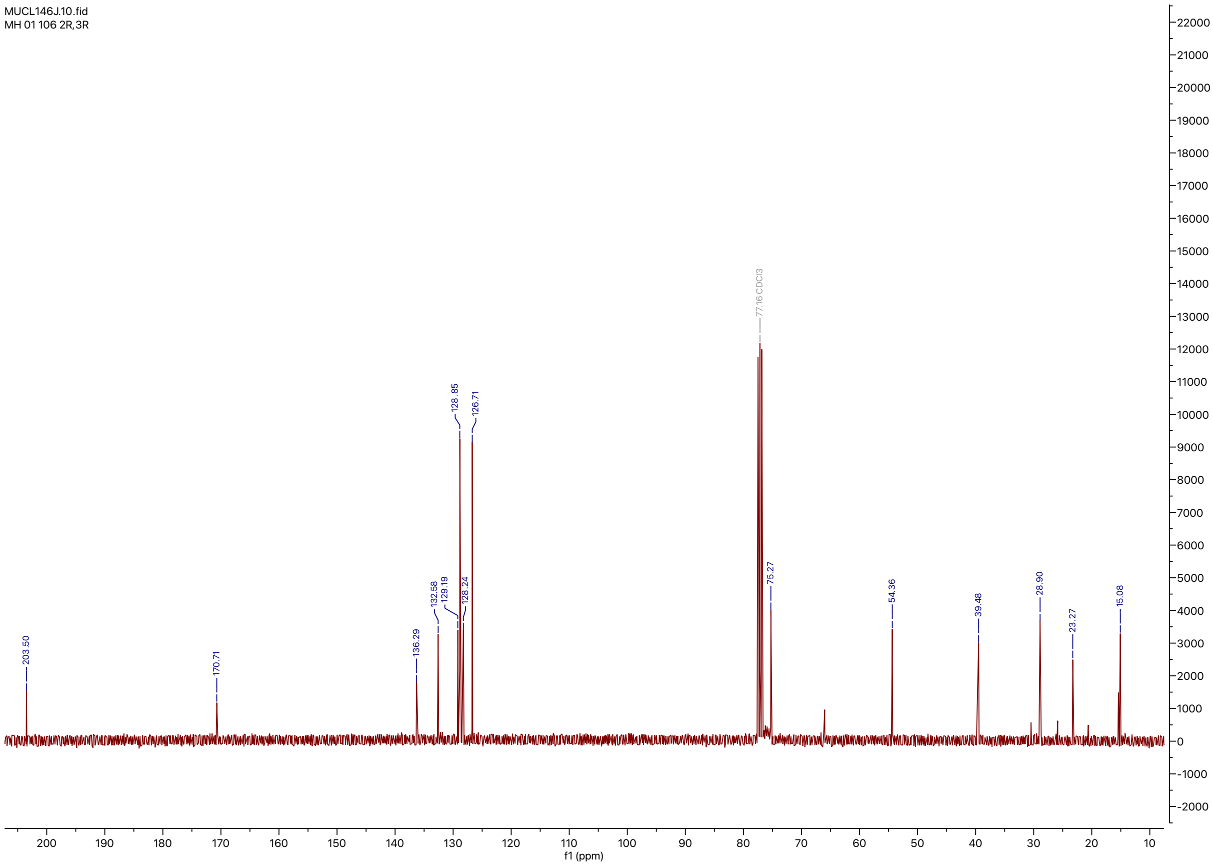

Figure S3.3.6L ^{13}C NMR of D-*anti*-**13b** in $CDCl_3$.

3.3.7 Compounds 14

Methyl (2*Z*,4*E*)-5-phenylpenta-2,4-dienoate 2*Z*-46a[24]

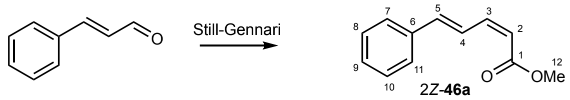

Methyl-2-(bis(2,2,2-trifluoroethoxy)phosphoryl)acetate (Sigma-Aldrich, 1.1 eq., 0.09 mL, 0.42 mmol) was dissolved in THF (3 mL) and cooled to -78 °C, To the cooled solution NaHMDS (2M in THF, 1.0 eq., 0.19 mL, 0.38 mmol) was added dropwise. After complete addition the reaction mixture was allowed to stir for 30 min before trans-cinnamic acid (1.0 eq., 0.05mL, 0.38 mmol) in THF (1 mL) was added. After complete addition the reaction was stirred until completion and carefully quenched with sat. NH_4Cl (3 mL). The phases were separated, and the aqueous phase was extracted with Et_2O (3 × 3 mL). The combined organic layers were dried over $NaSO_4$, filtered and concentrated in *vacuo*. The crude residue was purified by flash chromatography (petroleum ether : ethyl acetate 25:1) to give the desired diene 2*Z*-**46a**[24] (67 mg, 0.36 mmol, 95%) as a yellow oil as a pure diastereomer based on ^{1}H NMR.

UV$_{\lambda max}$ (CH_3CN:H_2O): 230, 310 nm
^{1}H-NMR (400 MHz, $CDCl_3$): δ = 8.14 (1H, ddd, *J* = 15.7, 11.4, 1.2 Hz, H-4), 7.57 - 7.52 (2H, m, ArH), 7.38 - 7.28 (3H, m, ArH)), 6.83 (1H, d, *J* = 15.7 Hz, H-5), 6.76 (1H, dd, *J* = 11.3, 1.2 Hz, H-2), 5.74 (1H, ddd, *J* = 11.3, 1.0 Hz, H-3), 3.77 (3H, s, H-12) ppm.
ESI-MS (*m/z*): 189.2 $[M + H]^+$.

Ethyl (2*Z*,4*E*)-2-methyl-5-phenylpenta-2,4-dienoate 2*Z*-46b[25]

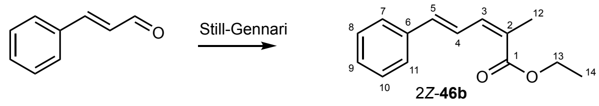

Ethyl-*bis*(2,2,2-trifluoroethoxy)phosphinyl propionate (Sigma-Aldrich, 1.0 eq., 200 mg, 0.58 mmol) was dissolved in THF (4 mL) and cooled to -78 °C. To the cooled solution NaHMDS (2M in THF, 1.1 eq., 0.32 mL, 0.64 mmol) was added dropwise. After complete addition the reaction mixture was allowed to stir for 15 min before *trans*-cinnamaldehyde (1.0 eq., 0.07 mL, 0.58 mmol) in THF (1 mL) was added. After complete addition the reaction was stirred until completion and carefully quenched with sat. NH_4Cl (3 mL). The phases were separated, and the aqueous phase was extracted with Et_2O (3 × 3 mL). The combined organic layers were dried over $NaSO_4$, filtered and concentrated in *vacuo*. The crude residue was purified by flash chromatography (petroleum ether : ethyl acetate 20:1) to give the desired 2*Z*-**46b**[25] diene (87 mg, 0.40 mmol, 69%) as a yellow oil as a pure diastereomer based on ^{1}H NMR.

^{1}H-NMR (400 MHz, $CDCl_3$): δ = 7.92 (1H, dd, *J* = 15.7, 11.3 Hz, H-4), 7.48 - 7.46 (2H, m, ArH), 7.35 - 7.31 (2H, m, ArH), 7.28 - 7.24 (1H, m, ArH), 6.69 (1H, d, *J* = 15.7 Hz, H-5), 6.60 (1H, dq, *J* = 11.1, 1.3 Hz, H-3), 4.27 (2H, q, *J* = 7.2 Hz, H-13), 2.03 (3H, d, *J* = 1.3 Hz, H-12), 1.37 (3H, t, *J* = 7.1 Hz, H-14) ppm.
^{13}C-NMR (100 MHz, $CDCl_3$): δ = 167.7 (C-1), 140.4 (C-3), 138.0 (C-5), 136.9 (C-6), 128.7(C-8/10), 128.3 (C-4), 127.1 (C-7/11), 126.5 (C-2), 126.1 (C-9), 60.4 (C-13), 21.0 (C-12), 14.4 (C-14) ppm.
ESI-MS (*m/z*): 217.2 $[M + H]^+$.

Ethyl (2*E*,4*E*)-5-phenylpenta-2,4-dienoate 2*E*-46a

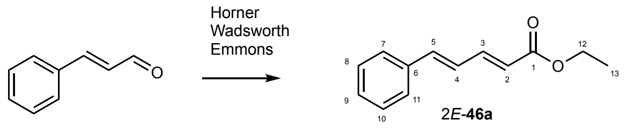

A stirred solution of NaH (60% in mineral oil, eq., 114 mg, 2.84 mmol) in THF (4 mL) was cooled to 0 °C and triethyl phosphonoacetate (1.2 eq., 0.45 mL, 2.27 mmol) was added dropwise. After complete addition the reaction mixture was allowed to stir for 30 min before *trans*-cinnamaldehyde (1.0 eq., 0.24 mL, 1.89 mmol) was added. The reaction mixture was allowed to warm to room temperature and stirred until completion. The reaction was quenched with the addition of sat. NH_4Cl (4 mL) and the phases were separated. The aqueous phase was extracted with Et_2O (3 × 5 mL) and the combined organic layers were dried over $NaSO_4$, filtered and concentrated *in vacuo*. The crude residue was purified by flash chromatography (petroleum ether : ethyl acetate 20:1) to give the desired diene 2*E*-**46a** as a yellow oil (244 mg, 1.21 mmol, 64%) and as a pure diastereomer based on ^{1}H NMR.

UV$_{\lambda max}$ (CH_3CN:H_2O): 225, 309 nm
^{1}H-NMR (400 MHz, $CDCl_3$): δ = 7.50 - 7.25 (6H, m, ArH & H-5), 6.89 (2H, m, H-3 & H-4), 5.99 (1H, d, *J* = 15.3 Hz, H-2), 4.23 (2H, q, *J* = 7.1 Hz, H-12), 1.32 (3H, t, *J* = 7.1 Hz, H-13) ppm.
ESI-MS (*m/z*): 203.1 $[M + H]^+$.

Ethyl (2*E*,4*E*)-2-methyl-5-phenylpenta-2,4-dienoate 2*E*-46b[25]

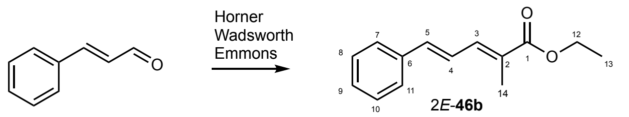

A stirred solution of NaH (60% in mineral oil, 2.0 eq., 60.8 mg, 1.52 mmol) in THF (1.5 mL) was cooled to 0 °C and triethyl 2-phosphonopropionate (1.2 eq., 0.2 mL, 0.91 mmol) was added dropwise. After 30 min stirring, *trans*-cinnamaldehyde (1.0 eq., 0.1 mL, 0.76 mmol) dissolved in THF (1 mL) was added dropwise and the resulting reaction mixture was allowed to warm to room temperature. After completion the reaction was quenched with the addition of sat. NH_4Cl (4 mL) and the phases were separated. The aqueous phase was extracted with Et_2O (3 × 5 mL) and the combined organic layers were dried over $NaSO_4$, filtered and concentrated in vacuo. The crude residue was purified by flash chromatography (petroleum ether : ethyl acetate 20:1) to give the pure diene 2*E*-**46b**[25] as a yellow oil (130 mg, 0.60 mmol, 79%) and as a pure diastereomer based on ^{1}H NMR.

^{1}H-NMR (400 MHz, $CDCl_3$): δ = 7.50 - 7.47 (2H, m, ArH), 7.38 - 7.34 (3H, m, ArH), 7.31 - 7.27 (1H, m, H-3), 7.07 (1H, dd, *J* = 15.4, 11.3 Hz, H-4), 6.87 (1H, d, *J* = 15.4 Hz, H-5), 4.24 (2H, q, *J* = 7.1 Hz, H-13), 2.05 (3H, d, *J* = 1.4 Hz, H-12), 1.33 (3H, t, *J* = 7.1 Hz, H-14) ppm.
^{13}C-NMR (100 MHz, $CDCl_3$): δ = 168.6 (C-1), 139.1 (C-3), 138.3 (C-5), 136.8 (C-6), 128.9 (C-8/10), 128.8 (C-9), 127.6 (C-2), 127.2 (C-7/11), 124.1 (C-4), 60.8 (C-13), 14.5 (C-14), 13.0 (C-12) ppm.
ESI-MS (*m/z*): 217.2 $[M + H]^+$.

***S*-(2-Acetamidoethyl) (2*Z*,4*E*)-5-phenylpenta-2,4-dienethioate 2*Z*-14a**

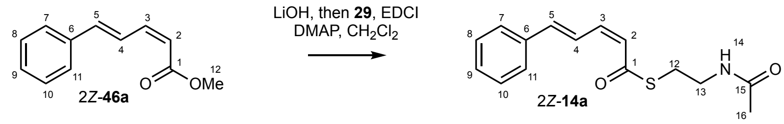

Chemical Formula: $C_{15}H_{17}NO_2S$
Exact Mass: 275.0980

To a stirred solution of ester 2*Z*-**46a** (1eq., 20 mg, 0.11 mmol) in THF (1 mL) and MeOH (1 mL) was added LiOH·H_2O (3.0 eq., 13 mg, 0.32 mmol) in H_2O (1 mL) and heated to 40 °C. After 2 h the reaction was allowed to cool to room temperature, diluted with H_2O (2 mL) and adjusted to a pH of 3 with aq. HCl (1 M). The solution was extracted with EtOAc (3 × 3 mL), the combined organic layers were dried over $MgSO_4$, filtered and concentrated under reduced pressure to give the carboxylic acid, which was directly used without further characterisation. To a solution of the acid in CH_2Cl_2 (1 mL) was added EDCI (2.5 eq., 53 mg, 0.28 mmol), DMAP (0.2 eq., 2.4 mg, 0.02 mmol) and HSNAC **29** (1.2 eq., 16 mg, 0.13 mmol) subsequently at 0 °C. After 16 h the reaction mixture was quenched with H_2O (3 mL) and extracted with CH_2Cl_2 (3 × 2 mL). The combined organic phases were dried over $MgSO_4$, filtered and concentrated in *vacuo*. The resulting yellow oil was purified by flash chromatography (ethyl acetate/petroleum ether 1:1) to give title compound 2Z-**14a** (16 mg, 0.06 mmol, 53%) as a white solid and in a diastereomeric mixture of 10:1 Z/*E* based on ^{1}H NMR.

UV$_{\lambda max}$ (CH_3CN:H_2O): 233, 336 nm
^{1}H-NMR (400 MHz, $CDCl_3$): δ = 8.08 (1H, ddd, *J* = 15.7, 11.4, 1.1 Hz, H-4), 7.54 - 7.52 (2H, m, ArH), 7.39 - 7.31 (3H, m, ArH), 6.91 (1H, d, *J* = 15.7 Hz, H-5), 6.61 - 6.50 (1H, td, *J* = 10.6, 0.9 Hz, H-2), 6.00 (dt, *J* = 10.9, 0.9 Hz, H-3), 5.90 (1H, brs, H-14), 3.50 (2H, q, *J* = 6.3 Hz, H-13), 3.13 (2H, dd, *J* = 6.8, 5.8 Hz, H-12), 1.98 (3H, s, H-16) ppm.
^{13}C-NMR (100 MHz, $CDCl_3$): δ = 189.5 (C-1), 170.4 (C-15), 143.6 (C-4), 141.9 (C-5), 136.0 (C-6), 129.4 (C-2), 128.8 (C-8/10), 127.7 (C-7/11), 125.4 (C-9), 123.0 (C-3), 39.8 (C-13), 28.8 (C-12), 23.3 (C-16) ppm.
ESI-MS (*m/z*): 276.3 $[M + H]^+$, 298.3 $[M + Na]^+$.
HRESIMS (*m/z*): calculated for $C_{15}H_{17}NO_2SNa$ $[M + Na]^+$: 298.0872 found 298.0880

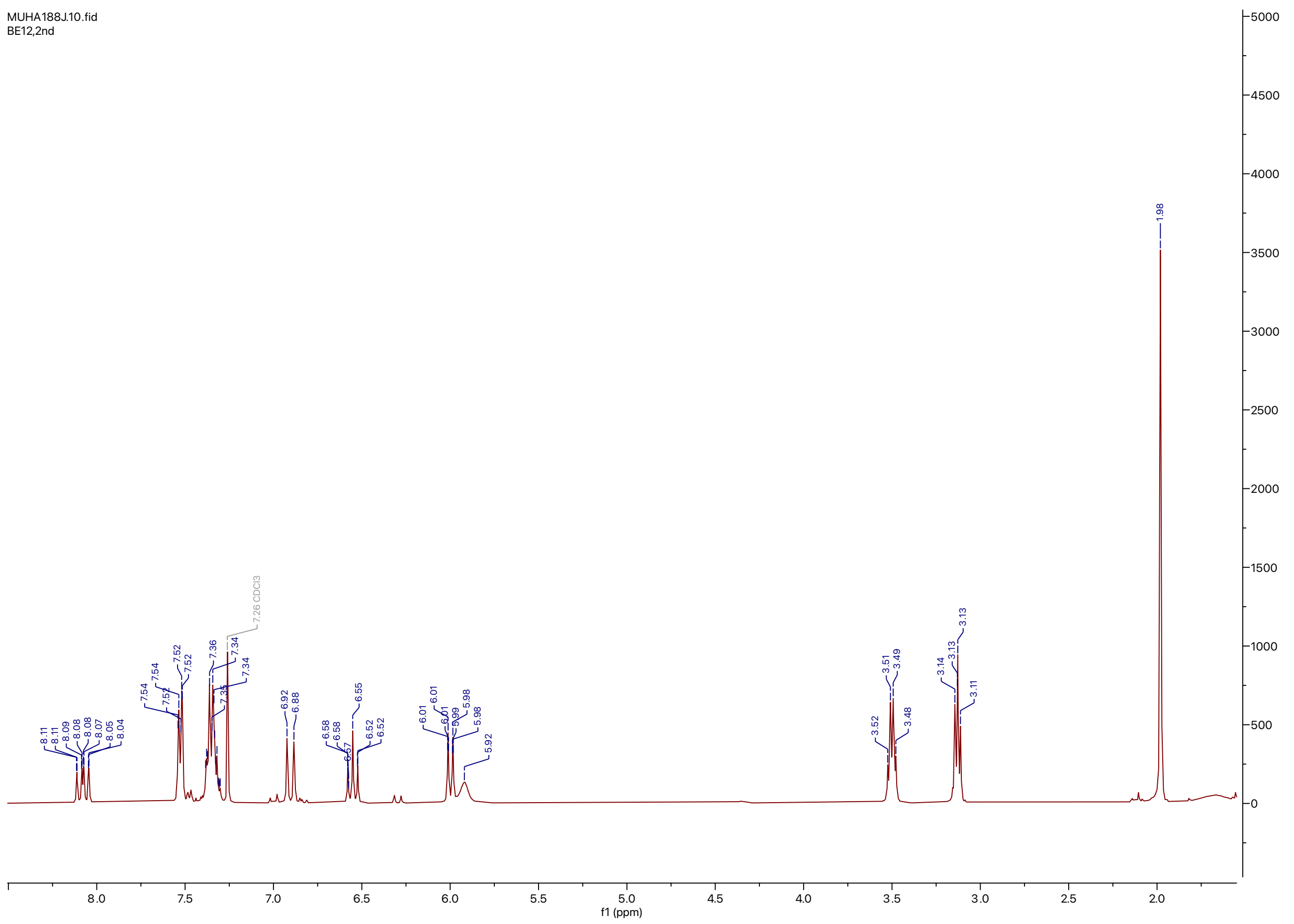

Figure S3.3.7A ^{1}H NMR of 2*Z*-**14a** in $CDCl_3$.

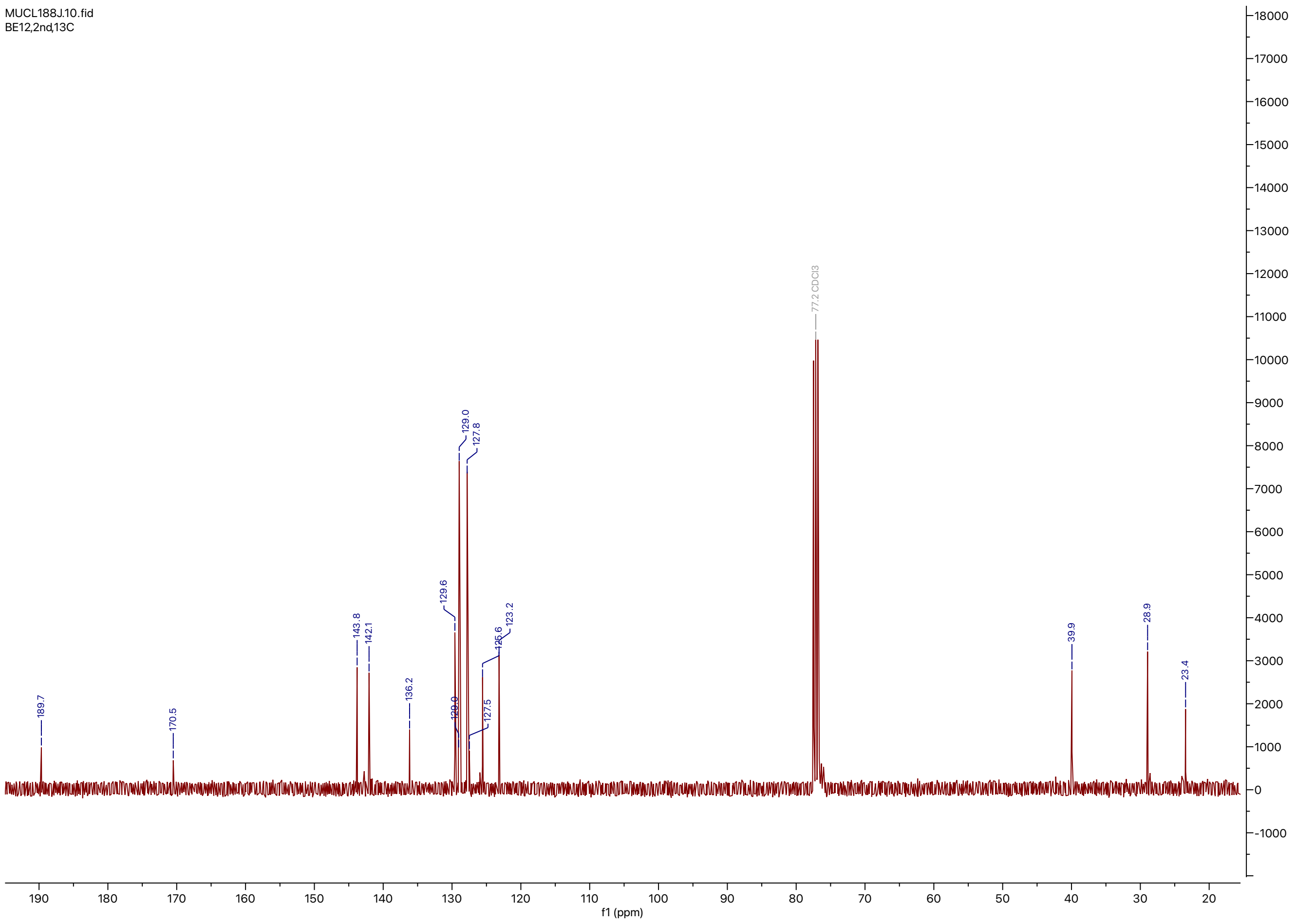

Figure S3.3.7B ^{13}C NMR of 2*Z*-**14a** in $CDCl_3$.

S-(2-Acetamidoethyl) (2*Z*,4*E*)-2-methyl-5-phenylpenta-2,4-dienethioate 2*Z*-14b

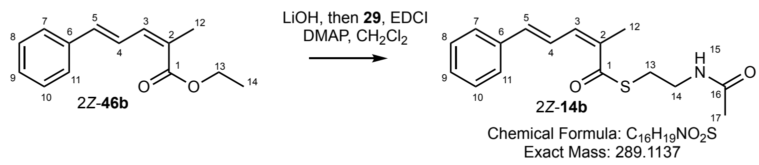

To a stirred solution of ester 2*Z*-**46b** (1eq., 20 mg, 0.09 mmol) in THF (1 mL) and MeOH (1 mL) was added LiOH·H_2O (3.0 eq., 11.6 mg, 0.277 mmol) in H_2O (0.5 mL) and heated to 40 °C. After 16 h the reaction was allowed to cool to room temperature, diluted with H_2O (2 mL) and adjusted to a pH of 3 with aq. HCl (1 M). The solution was extracted with EtOAc (3 × 3 mL), the combined organic layers were dried over $MgSO_4$, filtered and concentrated under reduced pressure to give the carboxylic acid, which was directly used without further characterisation. To a solution of the acid in CH_2Cl_2 (1 mL) was added EDCI (1.4 eq., 20.2 mg, 0.13 mmol), DMAP (0.1 eq., 1 mg, 0.01 mmol) and HSNAC (1.4 eq., 15.5 mg, 0.13 mmol) subsequently at 0 °C. After 16 h the reaction mixture was quenched with H_2O (3 mL) and extracted with CH_2Cl_2 (3 × 2 mL). The combined organic phases were dried over $MgSO_4$, filtered and concentrated in *vacuo*. The resulting yellow oil was purified by flash chromatography (ethyl acetate/petroleum ether 1:1) to give 2*Z*-**14b** (7 mg, 0.02 mmol, 22%) as a white solid and diastereomeric mixture of 2:1 *Z*/*E* based on ^{1}H NMR.

UV$_{\lambda max}$ (CH_3CN:H_2O): 222, 331 nm

^{1}H-NMR (400 MHz, $CDCl_3$): δ = 7.82 (1H, dd, *J* = 15.6, 11.3 Hz, H-4), 7.51 - 7.46 (3H, m, ArH), 7.49 - 7.31 (2H, m, ArH), 6.76 (1H, d, *J* = 15.6 Hz, H-5), 6.44 (1H, dq, *J* = 11.3, 1.2 Hz, H-3), 5.89 (1H, brs, H-15), 3.53 - 3.47 (2H, m, H-14), 3.16 - 3.11 (2H, m, H-13), 2.16 (3H, brs, H-12), 1.98 (3H, s, H-17) ppm.

^{13}C-NMR (100 MHz, $CDCl_3$): δ = 193.2 (C-1), 170.4 (C-16), 141.0 (C-3), 140.4 (C-5), 138.8 (C-9), 136.7 (C-6), 131.5 (C-2), 128.9 (C-8/10), 127.4 (C-7/11), 125.7 (C-9), 39.9 (C-14), 28.7 (C-13), 20.9 (C-17), 13.1 (C-12) ppm.

ESI-MS (*m/z*): 290.3 $[M + H]^+$, 312.3 $[M + Na]^+$.

HRESIMS (*m/z*): calculated for $C_{16}H_{19}NO_2SNa$ $[M + Na]^+$: 312.1029 found 312.1021.

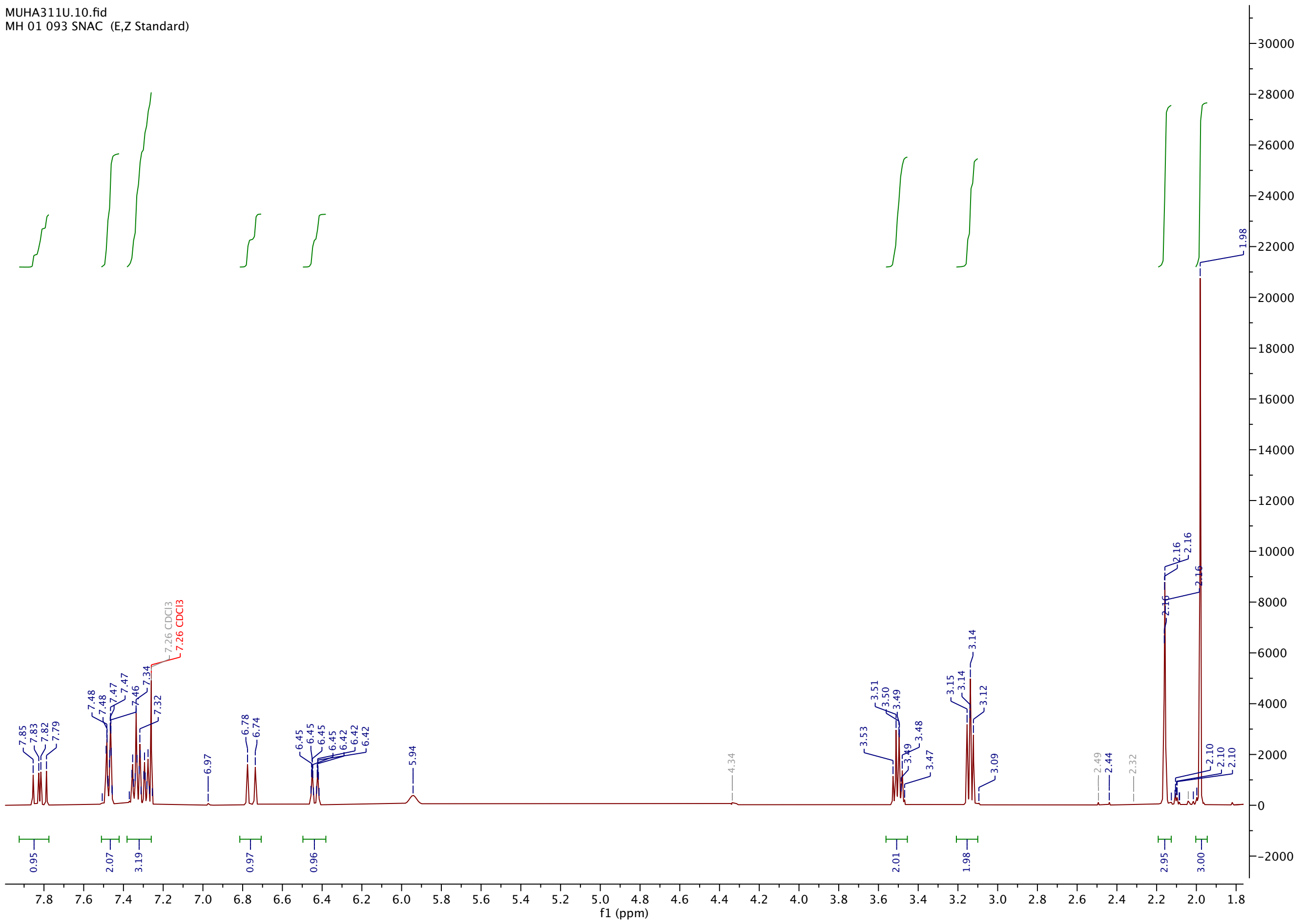

Figure S3.3.7C ^{1}H NMR of 2*Z*-**14b** in $CDCl_3$.

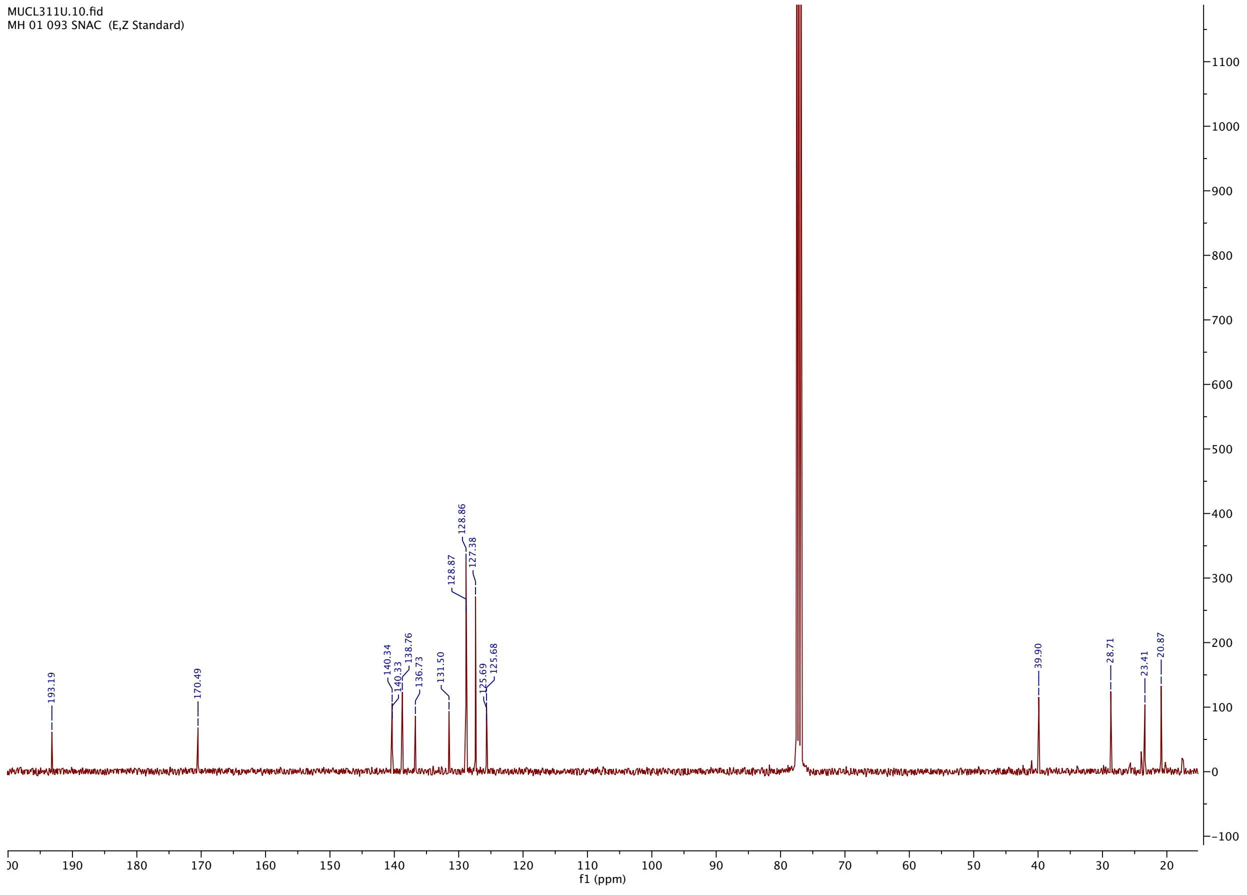

Figure S3.3.7D ^{13}C NMR of 2*Z*-**14b** in $CDCl_3$.

S-(2-Acetamidoethyl) (2*E*,4*E*)-5-phenylpenta-2,4-dienethioate 2*E*-14a

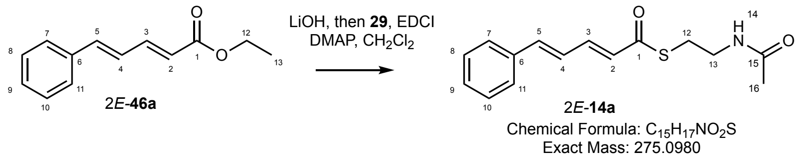

To a stirred solution of ester 2*E*-**46a** (1eq., 50 mg, 0.25 mmol) in THF (2 mL) and MeOH (1.5 mL) was added LiOH·H_2O (3 eq., 31 mg, 0.74 mmol) in H_2O (1.5 mL). After 2 h the reaction was diluted with H_2O (2mL) and adjusted to a pH of 3 with aq. HCl (1 M). The solution was extracted with EtOAc (3 × 3 mL), the combined organic layers were dried over $MgSO_4$ and concentrated under reduced pressure to give the free acid. To a solution of the crude acid in CH_2Cl_2 (5 mL) and HSNAC (1.2 eq., 35 mg, 0.30 mmol) was added EDCI (2.5 eq., 118 mg, 0.62 mmol) and DMAP (0.2 eq., 6 mg, 0.05 mmol) at 0 °C. After 16 h the reaction mixture was quenched with H_2O (5 mL) and extracted with CH_2Cl_2 (3 × 5 mL). The combined organic phases were dried over $MgSO_4$, filtered and concentrated in *vacuo*. The resulting yellow oil was purified by flash chromatography (ethyl acetate/petroleum ether 1:1) to give pure 2*E*-**14a** (25 mg, 0.09 mmol, 36%) as a white solid and as a pure diastereomer based on ^{1}H NMR.

UV$_{\lambda max}$ (CH_3CN:H_2O): 234, 328 nm

^{1}H-NMR (400 MHz, $CDCl_3$): δ = 7.49 - 7.30 (6H, m, ArH & H-3), 7.00 (1H, d, *J* = 15.6 Hz, H-5), 6.84 (1H, dd, *J* = 15.6, 10.9 Hz, H-4), 6.29 (1H, d, *J* = 15.1 Hz, H-2), 5.98 (1H, brs, H-14), 3.51 - 3.47 (2H, m, H-13), 3.14 (2H, t, *J* = 6.3 Hz, H-12), 1.98 (3H, s, H-16) ppm.

^{13}C-NMR (100 MHz, $CDCl_3$): δ = 190.3 (C-1), 170.5 (C-15), 142.7 (C-5), 141.6 (C-4), 136.0 (C-6), 129.6 (C-3), 129.0 (C-8/10), 127.7 (C-9), 127.5 (C-7/11), 125.9 (C-2), 40.0 (C-13), 28.6 (C-12), 23.4 (C-16) ppm.

ESI-MS (*m/z*): 276.3 $[M + H]^+$, 298.3 $[M + Na]^+$.

HRESIMS (*m/z*): calculated for $C_{15}H_{17}NO_2SNa$ $[M + Na]^+$: 298.0872 found 298.0878

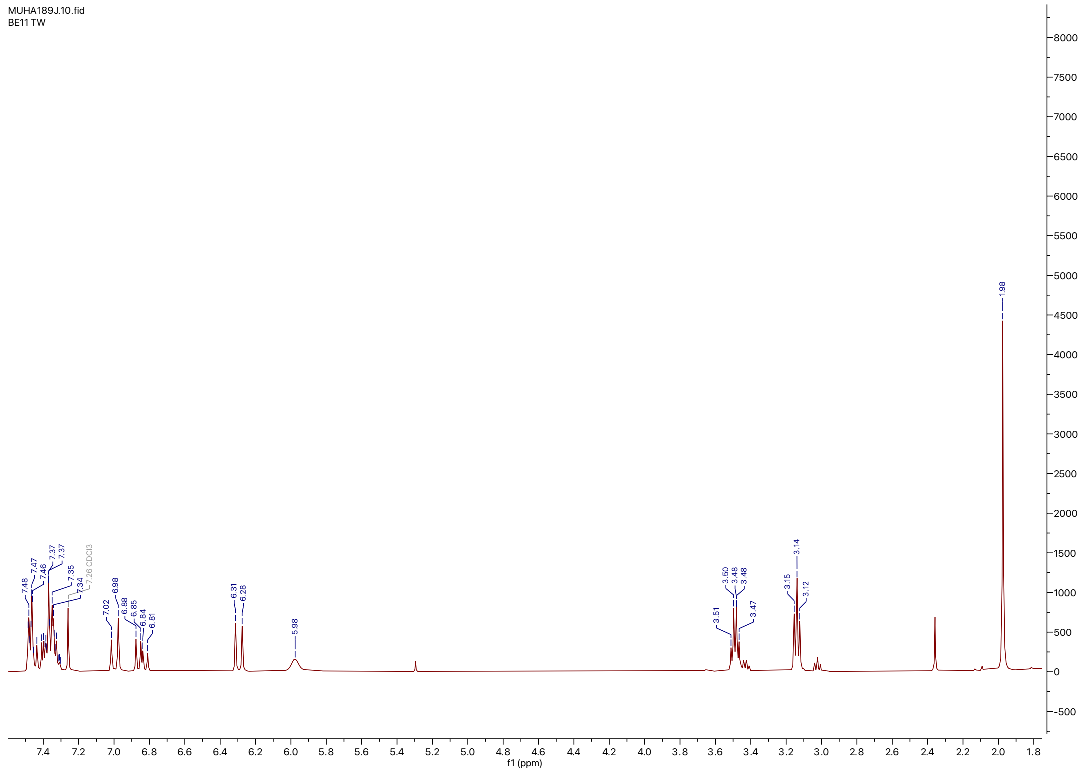

Figure S3.3.7E ^{1}H NMR of 2*E*-**14a** in $CDCl_3$.

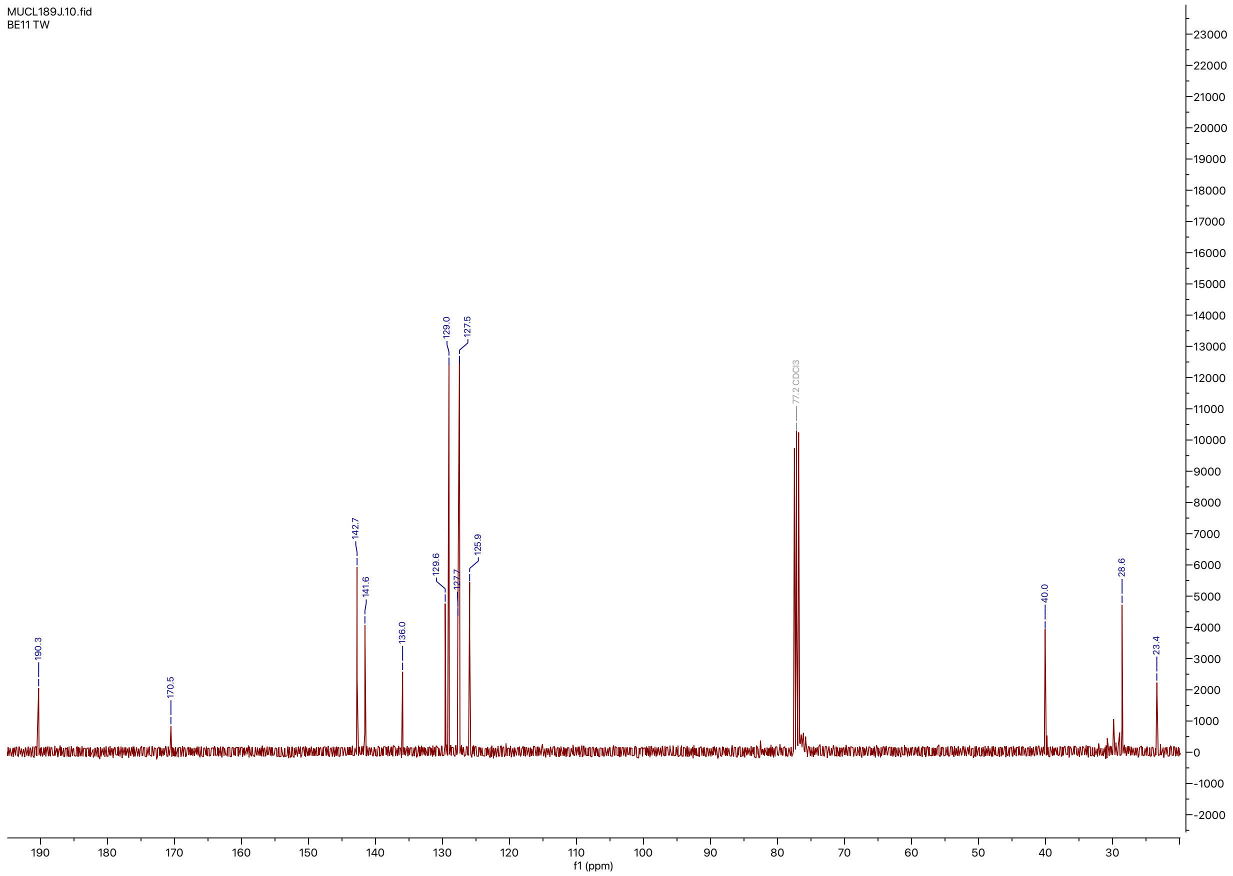

Figure S3.3.7F ^{13}C NMR of 2*E*-**14a** in $CDCl_3$.

S-(2-Acetamidoethyl) (2*E*,4*E*)-2-methyl-5-phenylpenta-2,4-dienethioate 2*E*-14b

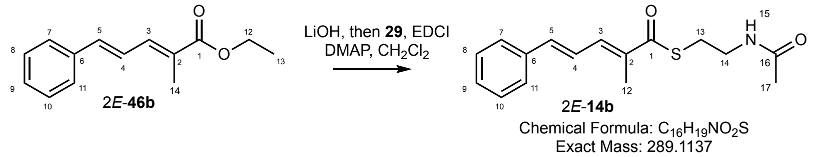

To a stirred solution of ethyl ester 2*E*-**46b** (1eq., 40 mg, 0.19 mmol) in THF (2 mL) and MeOH (2 mL) was added LiOH·H_2O (3.0 eq., 23 mg, 0.56 mmol) in H_2O (1 mL) and heated to 40 °C. After 16 h the reaction was allowed to cool to room temperature, diluted with H_2O (2 mL) and adjusted to a pH of 3 with aq. HCl (1 M). The solution was extracted with EtOAc (3 × 3 mL), the combined organic layers were dried over $MgSO_4$, filtered and concentrated under reduced pressure to give the free acid, which was directly used without further characterisation. To a solution of the acid in CH_2Cl_2 (1.5 mL) was added EDCI (1.4 eq., 40.4 mg, 0.26 mmol), DMAP (0.1 eq., 2 mg, 0.02 mmol) and HSNAC (1.4 eq., 31 mg, 0.26 mmol) subsequently at 0 °C. After 16 h the reaction mixture was quenched with H_2O (3 mL) and extracted with CH_2Cl_2 (3 × 3 mL). The combined organic phases were dried over $MgSO_4$, filtered and concentrated *in vacuo*. The resulting yellow oil was purified by flash chromatography (ethyl acetate/petroleum ether 1:1) to give pure title compound 2*E*-**14b** (32 mg, 0.11 mmol, 60%) as a white solid.

UV$_{\lambda max}$ (CH3CN:H_2O): 223, 335 nm.

^{1}H-NMR (400 MHz, $CDCl_3$): δ = 7.53 - 7.44 (2H, m, ArH & H-3), 7.41 - 7.27 (4H, m, ArH), 7.07 (1H, dd, *J* = 15.4, 10.9 Hz, H-4), 6.95 (1H, d, *J* = 15.4 Hz, H-5), 5.95 (1H, brs, H-15), 3.48 (2H, dt, *J* = 6.6, 5.8 Hz, H-14), 3.12 (2H, dd, *J* = 6.8, 5.9 Hz, H-13), 2.10 (3H, d, *J* = 1.3 Hz, H-12), 1.98 (3H, s, H-17) ppm.

^{13}C-NMR (100 MHz, $CDCl_3$): δ = 193.6 (C-1), 170.4 (C-16), 140.9 (C-3), 137.9 (C-2), 136.4 (C-5), 134.6 (C-4), 129.3 (C-6),129.0 (C-8/10), 127.4 (C-7/11), 123.5 (C-9), 40.0 (C-13), 28.7 (C-12), 23.4 (C-16), 13.1 (C-14) ppm.

ESI-MS (*m/z*): 290.3 $[M + H]^+$, 312.3 $[M + Na]^+$

HRESIMS (*m/*z): calculated for $C_{16}H_{19}NO_2SNa$ $[M + Na]^+$: 312.1029 found 312.103

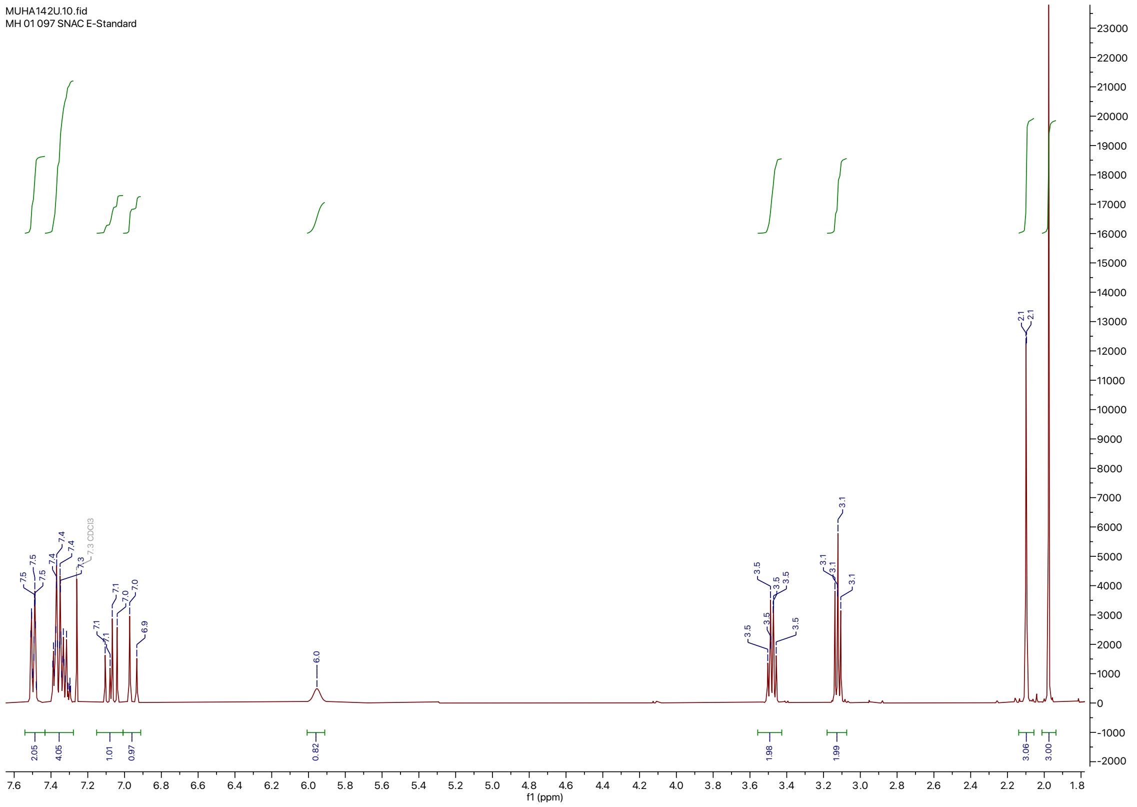

Figure S3.3.7G ^{1}H NMR of 2*E*-**14b** in $CDCl_3$.

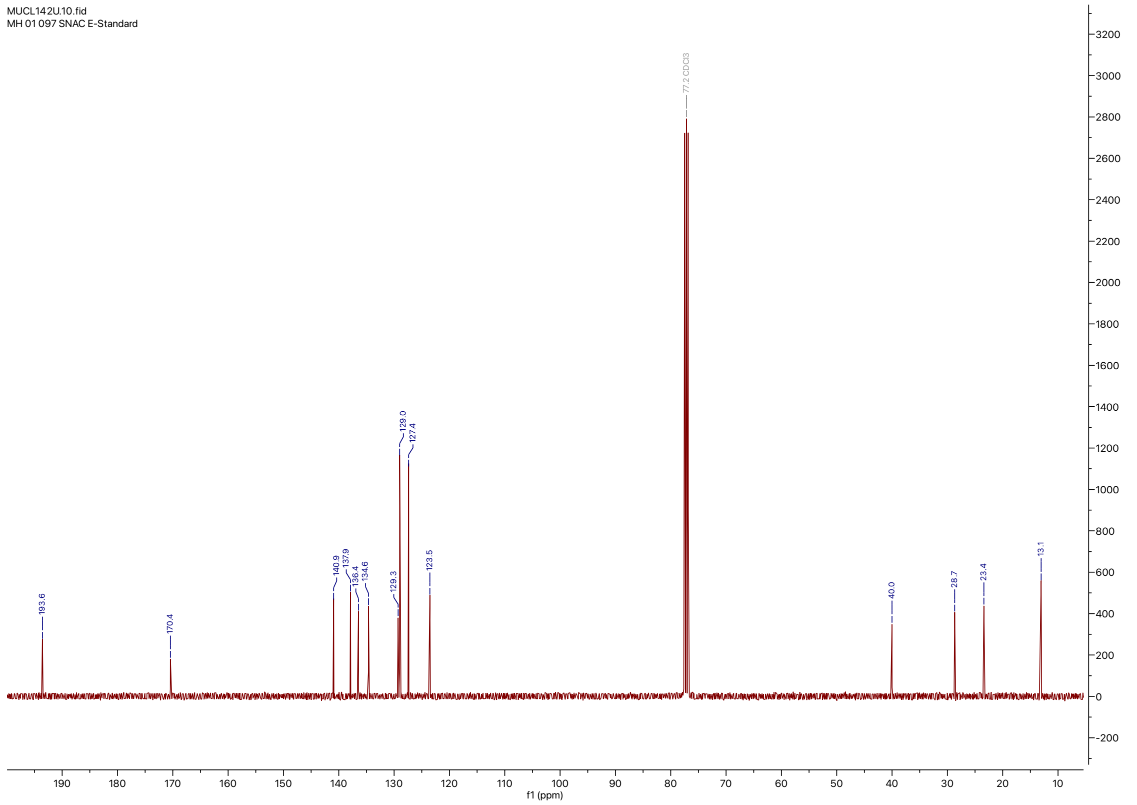

Figure S3.3.7H ^{13}C NMR of 2*E*-**14b** in $CDCl_3$.

3.3.8 Compounds 15

Methyl 2-(2-phenyl-1,3-dioxolan-2-yl)acetate 35a

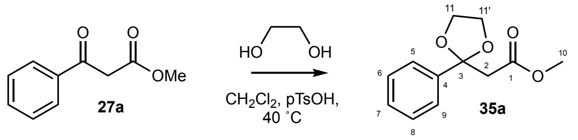

3-Oxo-3-phenyl methyl propionate (Sigma-Aldrich, 1.0 eq., 100 mg, 0.56 mmol) was dissolved in CH_2Cl_2 (2 mL) and *p*-TsOH·H_2O (0.6 eq., 65 mg, 0.34 mmol), ethylene glycol (10 eq., 0.32 mL, 5.6 mmol) and $(CH_3O)_3CH$ (5 eq., 0.30 mL, 2.8 mmol) were added. The reaction vial was then sealed, heated to 40 °C and stirred overnight. The solution was allowed to cool to room temperature before it was diluted with H_2O (3 mL). The phases were separated and the aqueous phase extracted with CH_2Cl_2 (3 × 3 mL). The combined organic phases were dried over $MgSO_4$, filtered and concentrated *in vacuo*. The crude residue was purified by flash chromatography (ethyl acetate/petroleum ether 10:1) to furnish **35a** as a colourless oil (78 mg, 0.35 mmol, 63%).

^{1}H-NMR (400 MHz, $CDCl_3$): δ = 7.51 - 7.49 (2H, m, ArH), 7.37 - 7.29 (3H, m, ArH), 4.09 (2H, m, H-11), 3.82 (2H, m, H-11'), 3.63 (3H, s, H-10), 2.98 (2H, s, H-2) ppm.
^{13}C-NMR (100 MHz, $CDCl_3$): δ = 169.2 (C-1), 141.5 (C-4), 128.4 (C-7), 128.4 (C-6/8), 125.7 (C-5/9), 108.0 (C-3), 65.0 (C-11/11'), 51.9 (C-2), 45.8 (C-1) ppm.
ESI-MS (*m/z*): 223.1 $[M + H]^+$, 245.2 $[M + Na]^+$.

Ethyl 2-(2-phenyl-1,3-dioxolan-2-yl)propanoate 36b[26]

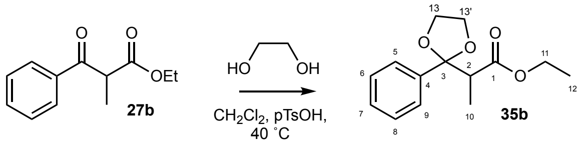

β-Ketoester **27b** (1.0 eq., 200 mg, 0.97 mmol) was dissolved in CH_2Cl_2 (3 mL) and *p*-TsOH·H_2O (0.6 eq., 110 mg, 0.58 mmol), ethylene glycol (10 eq., 0.56 mL, 9.70 mmol) and $(CH_3O)_3CH$ (5 eq., 0.53 mL, 4.85 mmol) were added. The reaction vial was then sealed, heated to 40 °C and stirred for up to 3 days. The solution was allowed to cool to room temperature before it was diluted with H_2O (3 mL). The phases were separated and the aqueous phase extracted with CH_2Cl_2 (3 × 3 mL). The combined organic phases were dried over $MgSO_4$, filtered and concentrated *in vacuo*. The crude residue was purified by flash chromatography (ethyl acetate/petroleum ether 10:1) to furnish **35b**[26] as a colourless oil (115 mg, 0.46 mmol, 47%).

^{1}H-NMR (400 MHz, $CDCl_3$): δ = 7.50 - 7.29 (5H, m, ArH), 4.06 - 4.00 (4H, m, H-13 & H-11), 3.80 - 3.76 (2H, m, H-13'), 3.07 (1H, p, *J* = 7.1 Hz, H-2), 1.18 (3H, d, *J* = 7.1 Hz, H-10), 1.09 (3H, t, *J* = 7.1 Hz, H-12) ppm.
^{13}C-NMR (100 MHz, $CDCl_3$): δ = 172.7 (C-1), 140.9 (C-4), 133.7 (C-7), 128.3, 128.1, 128.1, 126.4, 126.4, 109.7, 65.2, 64.8, 60.5, 49.3, 14.2, 12.2.
ESI-MS (*m/z*): 251.2 $[M + H]^+$, 273.1 $[M + Na]^+$

S-(2-Acetamidoethyl) 3-oxo-3-phenylpropanethioate 15a

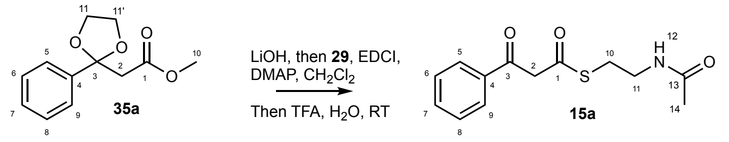

Chemical Formula: $C_{13}H_{15}NO_3S$
Exact Mass: 265.0773

Ethyl 2-(2-phenyl-1,3-dioxolan-2-yl)acetate **35a** (1.0 eq., 50 mg, 0.25 mmol) was dissolved in THF (0.8 mL) and to this solution was added LiOH·H_2O (4.0 eq., 42 mg, 1.0 mmol) dissolved in H_2O (0.8 mL). The reaction mixture was heated to 40 °C and vigorously stirred until completion. The mixture was diluted with H_2O (2 mL) and acidified to a pH 3.0 with aq. HCl (1M). The aqueous solution was extracted with CH_2Cl_2 (3 × 3 mL), dried over $MgSO_4$, filtered and concentrated in *vacuo*. The crude residue was dried under high vacuum prior dissolving it in CH_2Cl_2 (2 mL). The solution was cooled to 0 °C and EDCl (1.5 eq, 73 mg, 0.38 mmol) and DMAP (0.2 eq., 6 mg, 0.05 mmol) were added. The solution was allowed to stir for 10 min before HSNAC **29** (1.1 eq., 33 mg, 0.28 mmol) was added. The mixture was allowed to stir and warm to room temperature overnight before it was quenched with the addition of H_2O (2 mL). The phases were separated, and the aqueous phase was extracted with CH_2Cl_2 (2 × 2 mL). The combined organic phases were dried over $MgSO_4$, filtered and concentrated in *vacuo.* The crude residue was purified by flash chromatography (petroleum ether/ethyl acetate 4:1 to 1:1) topped with a 2-3 cm silica layer of immobilized $CuSO_4$ (to remove unreacted HSNAC) to furnish a colourless oil (52 mg, 0.17 mmol, 67%). In a final step, the acetal intermediate (1.0 eq., 10 mg, 0.03 mmol) was deprotected in CH_2Cl_2 (1 mL) with the addition of TFA (5 drops) and H_2O (50 µL). The solution was vigorously stirred, and the reaction progress was followed by LCMS. The reaction was diluted with the addition of H_2O (3 mL), when there was no starting material left. The phases were separated, and the aqueous phase was extracted with CH_2Cl_2 (2 × 2 ml). The combined organic layers were washed with brine, dried over $MgSO_4$, filtered and the solvent was removed under nitrogen flow. The residue was dried under high vacuum to furnish the desired compound **15a** (5.5 mg, 0.02 mmol, 69%) as a yellow solid.

UV$_{\lambda max}$ (CH_3CN:H_2O): 219 nm Keto : Enol 1.2:1

keto: **^{1}H-NMR** (400 MHz, $CDCl_3$): δ = 7.95 - 7.92 (2H, m, H-5/9), 7.84 - 7.76 (m, 2H), 7.64 - 7.59 (1H ddt, *J* = 8.6, 6.9, 1.3 Hz, H-7), 7.52 - 7.40 (2H, m, H-6/8), 6.13 (1H, brs, H-12), 4.26 (2H, s, H-2), 3.56 - 3.47 (2H, dq, *J* = 15.5, 6.0 Hz, H-11), 3.26 - 3.09 (2H, ddd, *J* = 12.2, 6.8, 5.7 Hz, H-10), 2.02 (3H, s, H-14) ppm.

^{13}C-NMR (100 MHz, $CDCl_3$): δ = 194.8 (C-2), 192.4 (C-3), 171.8 (C-13), 134.3 (C-4), 132.2 (C-7), 129.1 (C-6/8), 128.8 (C-5/9), 53.7 (C-2), 40.1 (C-11), 29.1 (C-10), 22.9 (C-14) ppm.

enol: **^{1}H-NMR** (400 MHz, $CDCl_3$): δ = 13.09 (s, 1H, OH), 7.81 - 7.78 (2H, m, H-5/9), 7.52 - 7.40 (3H, m, H-6/7/8), 6.52 (1H, brs, H-12), 6.13 (1H, s, H-2), 3.56 - 3.47 (2H, dq, *J* = 15.5, 6.0 Hz, H-11), 3.26 - 3.09 (2H, ddd, *J* = 12.2, 6.8, 5.7 Hz, H-10), 2.05 (3H, s, H-14) ppm.

^{13}C-NMR (100 MHz, $CDCl_3$): δ = 195.2 (C-1), 193.0 (C-3), 172.0 (C-13), 135.8 (C-4), 134.3 (C-7), 128.9 (C-6/8), 126.7 (C-5/9), 97.1 (C-2), 40.4 (C-11), 28.0 (C-10), 22.8 (C-14) ppm.

ESI-MS (*m/z*): 266.2 $[M + H]^+$, 288.2 $[M + Na]^+$

HRESIMS (*m/z*): calculated for $C_{13}H_{15}NO_3SNa$ $[M + Na]^+$: 288.0665 found 288.0659

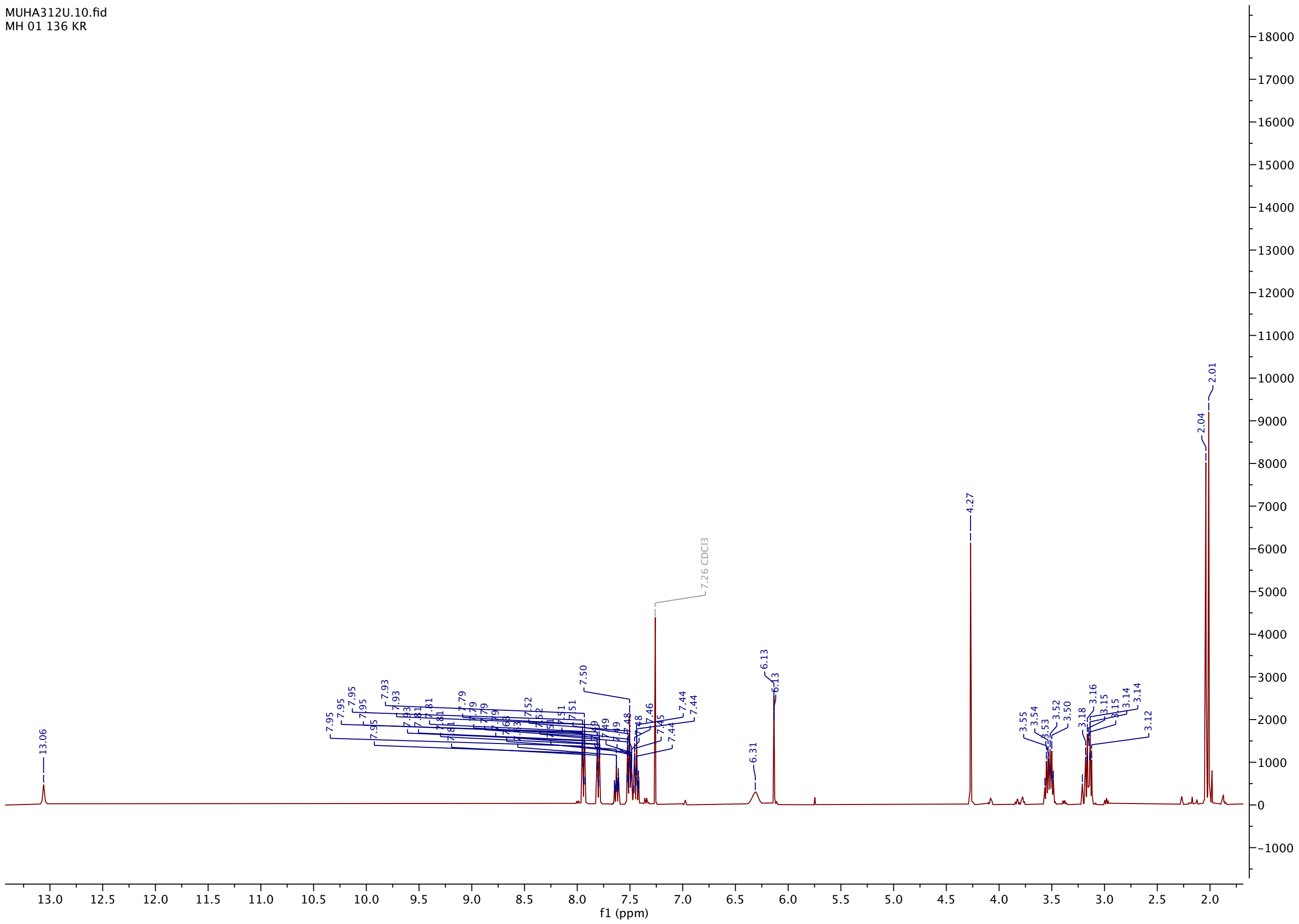

Figure S3.3.8A ^{1}H NMR of 2*E*-**15a** in $CDCl_3$. mixture of keto and enol forms.

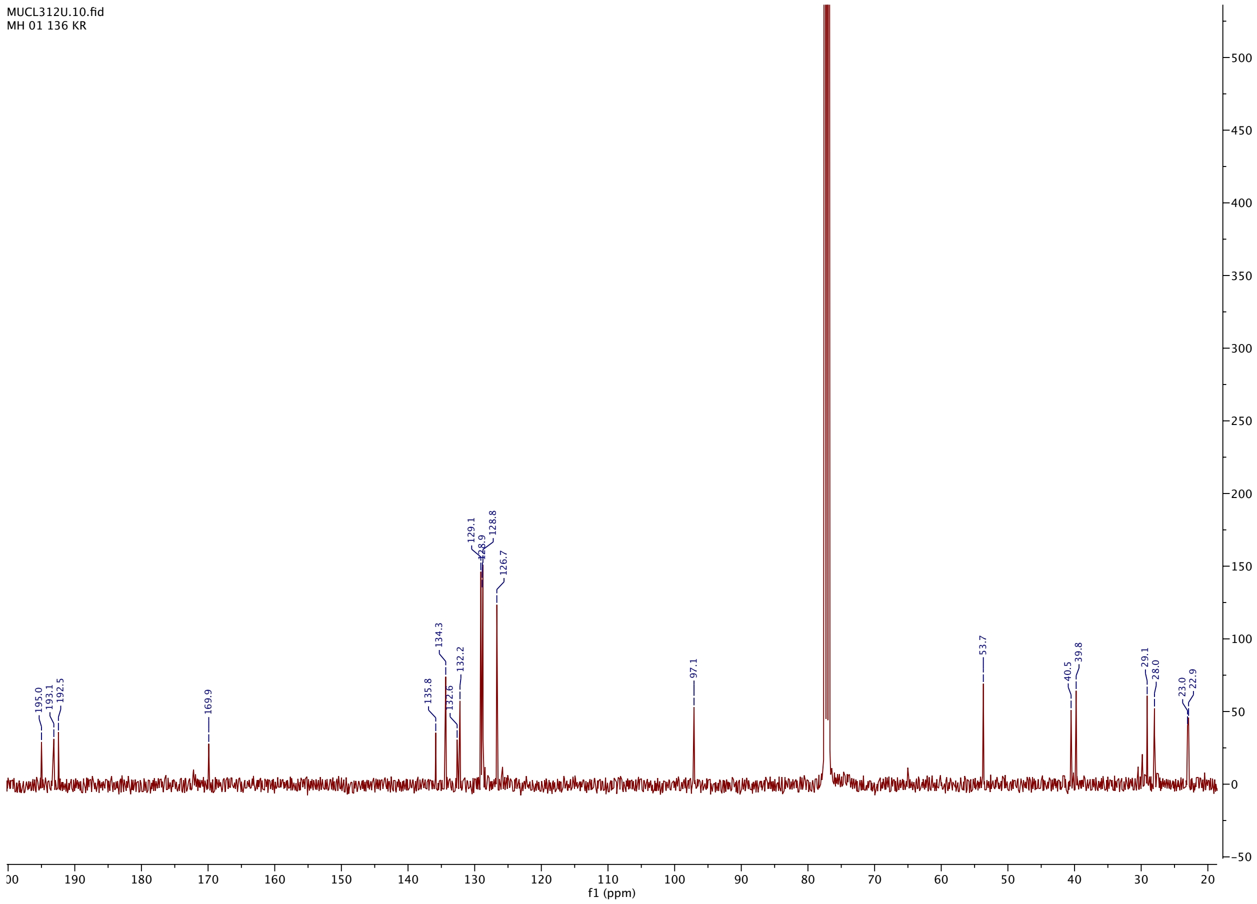

Figure S3.3.8A ^{13}C NMR of 2*E*-**15a** in $CDCl_3$. mixture of keto and enol forms.

S-(2-Acetamidoethyl) 2-methyl-3-oxo-3-phenylpropanethioate 15b

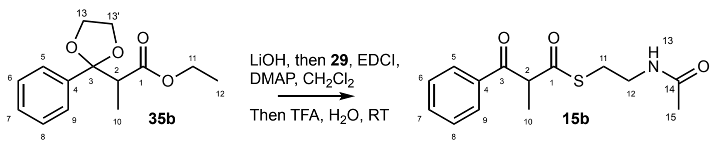

Chemical Formula: $C_{14}H_{17}NO_3S$
Exact Mass: 279.0929

Ethyl 2-(2-phenyl-1,3-dioxolan-2-yl)propanoate **35b** (1.0 eq., 76 mg, 0.30 mmol) was dissolved in THF (1 mL) to this solution was added LiOH·H_2O (4.0 eq., 50 mg, 1.2 mmol) dissolved in H_2O (1 mL). The reaction mixture was heated to 60 °C and vigorously stirred for up to 2 days. The mixture was diluted with H_2O (2 mL) and acidified to a pH 3.0 with aq. HCl (1M). The aqueous solution was extracted with CH_2Cl_2 (3 × 3 mL), dried over $MgSO_4$, filtered and concentrated in *vacuo*. The crude residue was dried under high vacuum prior dissolving it in CH_2Cl_2 (2 mL). The solution was cooled to 0 °C and EDCl (1.5 eq, 86 mg, 45 mmol) and DMAP (0.2 eq., 7 mg, 0.06 mmol) were added. The solution was allowed to stir for 10 min before HSNAC **29** (1.1 eq., 39 mg, 0.33 mmol) was added. The mixture was allowed to stir and warm to room temperature overnight before it was quenched with the addition of H_2O (2 mL). The phases were separated, and the aqueous phase was extracted with CH_2Cl_2 (2 × 2 mL). The combined organic phases were dried over $MgSO_4$, filtered and concentrated in *vacuo.* The crude residue was purified by flash chromatography (petroleum ether/ethyl acetate 4:1 to 2:1) topped with a 2-3 cm silica layer with immobilized $CuSO_4$ (to remove unreacted HSNAC) to furnish a colourless oil (53 mg, 0.16 mmol, 55%). In a final step, the acetal intermediate (1.0 eq., 10 mg, 0.03 mmol) was deprotected in CH_2Cl_2 (1 mL) with the addition of TFA (5 drops) and H_2O (50 µL). The solution was vigorously stirred, and the reaction progress was checked with LCMS. The reaction was diluted with the addition of H_2O (3 mL), when there was no starting material left. The phases were separated, and the aqueous phase was extracted with CH_2Cl_2 (2 × 2 ml). The combined organic layers were washed with brine, dried over $MgSO_4$, filtered and the solvent was removed under nitrogen flow. The residue was dried under high vacuum to furnish the desired compound **15b** (4.6 mg, 0.02 mmol, 55%) as a pale yellow solid.

UV$_{\lambda max}$ (CH_3CN:H_2O): 205, 246 nm

^{1}H-NMR (400 MHz, $CDCl_3$): δ = 8.00 - 7.97 (2H, m, H-6/8), 7.63 - 7.59 (1H, m H-7), 7.52 - 7.47 (2H, m, H-5/9), 5.76 (1H, brs, H-12), 4.68 (1H, q, *J* = 7.0 Hz, H-2), 3.49 - 3.32 (2H, m, H-11), 3.14 - 2.98 (2H, m, H-10), 1.87 (3H, s, H-14), 1.54 (3H, d, *J* = 7.0 Hz, H-15) ppm.

^{13}C-NMR (100 MHz, $CDCl_3$): δ = 197.2 (C-3), 195.1 (C-1), 170.4 (C-13), 135.6 (C-4), 133.8 (C-7), 128.9 (C-6/8), 128.8 (C-5/9), 56.3 (C-2), 39.4 (C-11), 28.8 (C-10), 23.1 (C-14), 14.7 (C-15) ppm.

ESI-MS (*m/z*): 280.2 $[M + H]^+$, 302.3 $[M + Na]^+$.

HRESIMS (*m/z*): calculated for $C_{14}H_{17}NO_3SNa$ $[M + Na]^+$: 302.0821 found 302.0817.

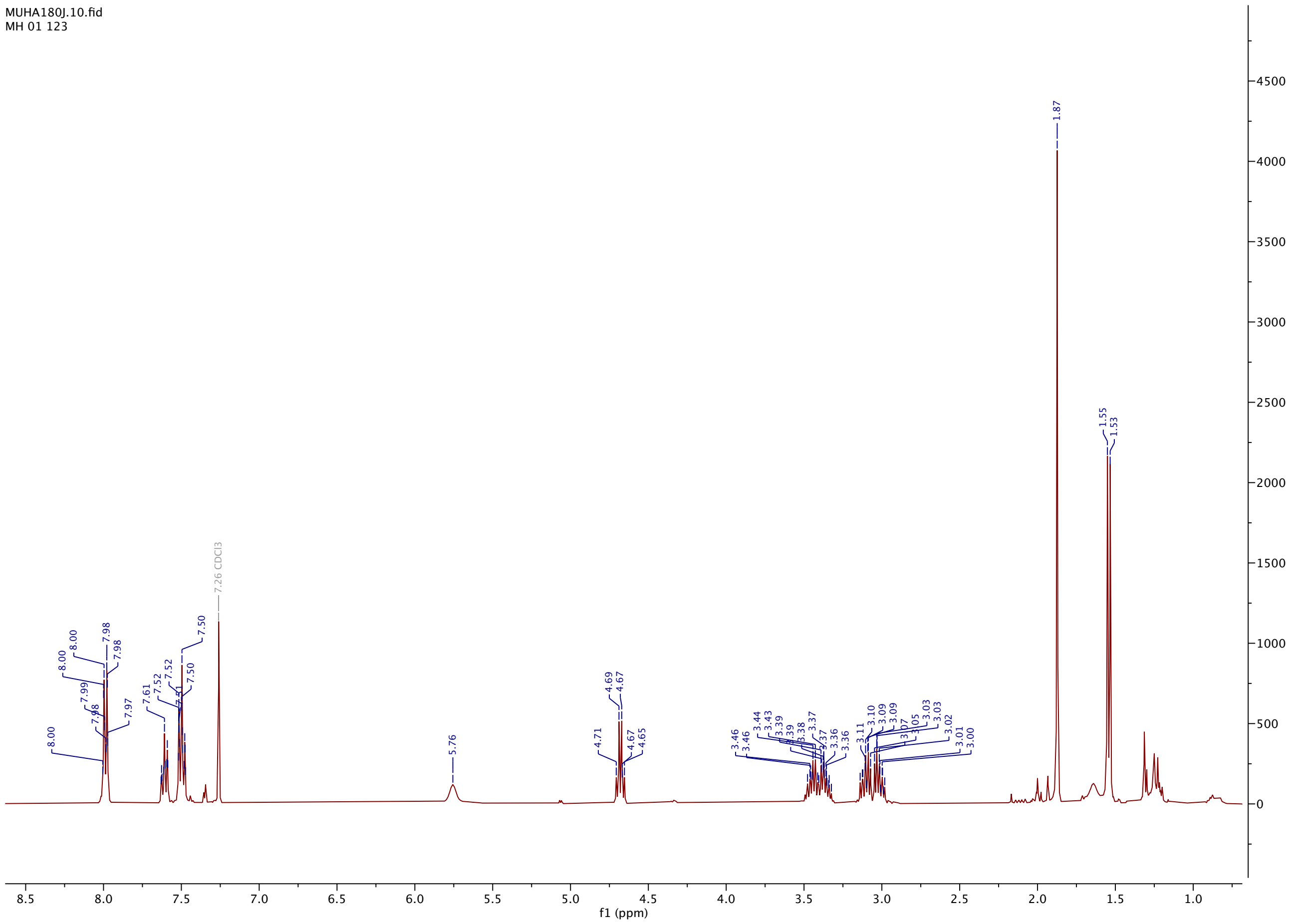

Figure S3.3.8C ^{1}H NMR of **15b** in $CDCl_3$. mixture of keto and enol forms.

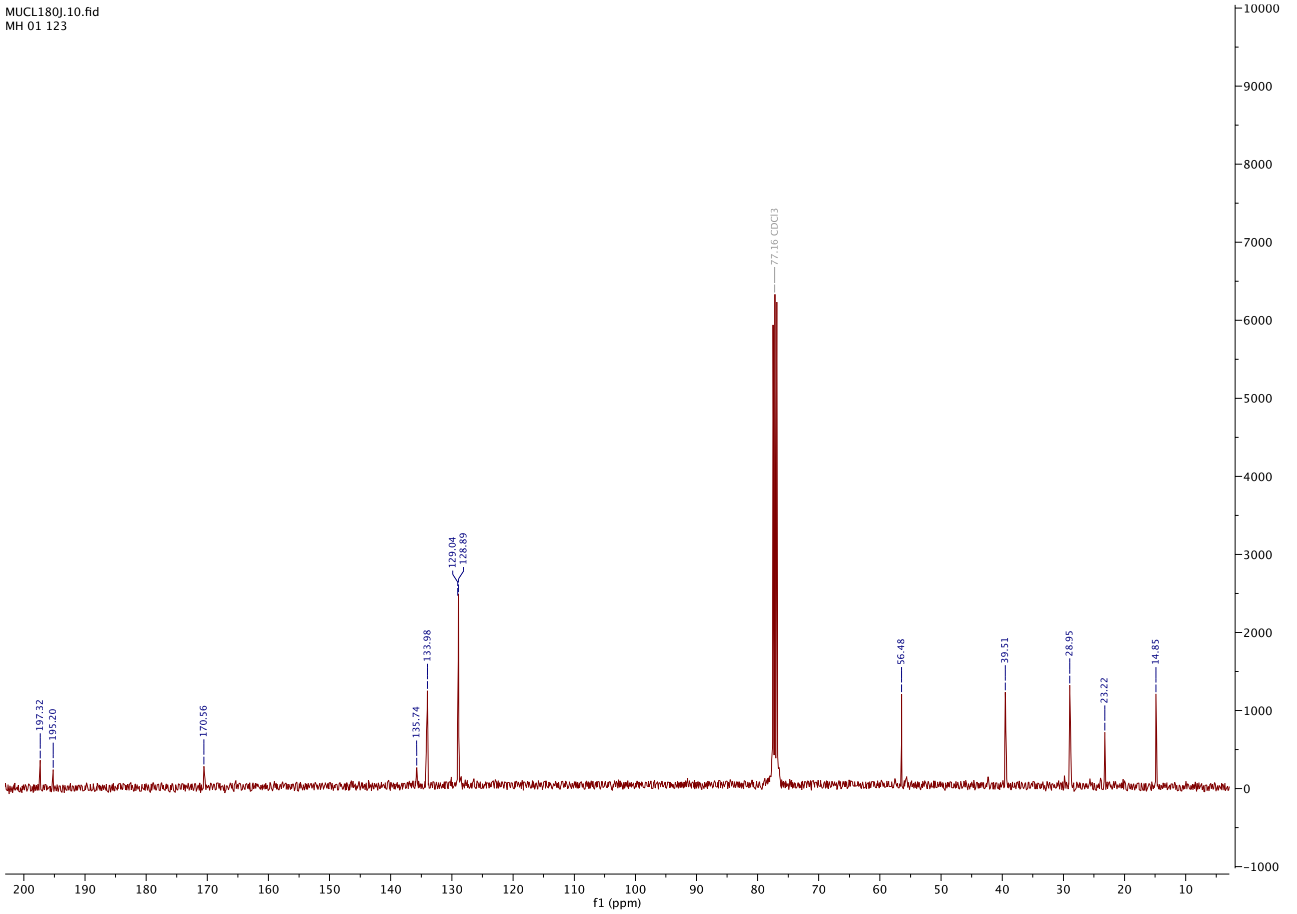

Figure S3.3.8D ^{13}C NMR of **15b** in $CDCl_3$. mixture of keto and enol forms.

3.3.9 Compounds 16

Ethyl *E*-3-oxo-5-phenylpent-4-enoate 42a[27]

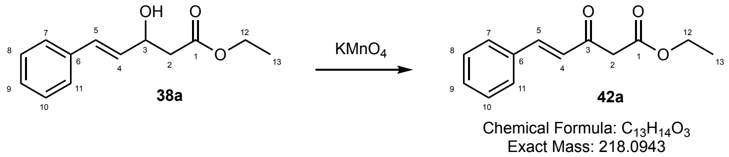

E-ethyl 3-hydroxy-5-phenylpent-4-enoate **38a** (1.0 eq., 260 mg, 1.18 mmol) was dissolved in CH_2Cl_2 (5 mL). To this solution $KMnO_4$ (8 eq., 1.5 g, 9.52 mmol) was added. The vial was sealed and the reaction mixture heated to 30 °C and stirred for up to 24 hours, monitored by TLC. After completion, the solids were removed by filtration through a celite plug. The resulting solution was diluted with CH_2Cl_2 (10 mL) and water (15 mL). The phases were separated, and the aqueous layer was extracted with CH_2Cl_2 (15 mL). The combined organic layers were washed with brine, dried over $MgSO_4$, filtered and concentrated in *vacuo*. The crude oily residue was purified by flash chromatography (petroleum ether : ethyl acetate 10:1) to give **42a**[27] as a yellow oil (189 mg, 0.87 mmol, 74%). Keto : Enol 2:1

keto **^{1}H-NMR** (400 MHz, $CDCl_3$): δ = 7.62 - 7.32 (6H, m, ArH, H-5), 6.81 (1H, d, *J* = 15.9 Hz, H-4), 4.32 - 4.16 (2H, m, H-12), 3.70 (2H, s, H-2), 1.34 - 1.25 (3H, m, H-13) ppm.
^{13}C-NMR (100 MHz, $CDCl_3$): 192.2 (C-3), 160.1 (C-1), 144.8 (C-5), 136.9 (C-6), 131.1 (C-9), 129.2 (C-8/10), 128.7 (C-7/11), 127.7 (C-4), 61.6 (C-12), 47.8 (C-2), 14.3 (C-13) ppm.
enol **^{1}H-NMR** (400 MHz, $CDCl_3$): δ = 11.99 (1H, s, OH), 7.62 - 7.32 (6H, m, ArH, H-5), 6.44 (1H, dd, *J* = 15.9, 1.4 Hz, H-4), 5.17 (1H, s, H-2), 4.32 - 4.16 (2H, m, H-12), 1.34 - 1.25 (3H, m, H-13) ppm.
ESI-MS (*m/z*): 231.3 $[M + Na]^+$

Ethyl *E*-2-methyl-3-oxo-5-phenylpent-4-enoate 42b[28]

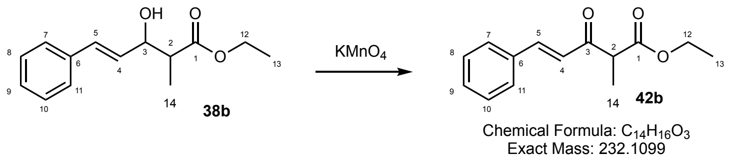

Ethyl *E*-3-hydroxy-2-methyl-5-phenylpent-4-enoate **38b** (1.0 eq., 202 mg, 0.86 mmol) was dissolved in CH_2Cl_2 (5 mL). To this solution $KMnO_4$ (8 eq., 1.10 g, 6.96 mmol) was added. The vial was sealed and the reaction mixture heated to 30 °C and stirred for up to 2 days, monitored by TLC. After completion, the solids were removed by filtration through a celite plug. The resulting solution was diluted with CH_2Cl_2 (10 mL) and water (15 mL). The phases were separated, and the aqueous layer was extracted with CH_2Cl_2 (15 mL). The combined organic layers were washed with brine, dried over $MgSO_4$, filtered and concentrated in *vacuo*. The crude oily residue was purified by flash chromatography (petroleum ether : ethyl acetate 10:1) to give **42b**[28] as a pale yellow oil (144 mg, 0.62 mmol, 72%). Keto : enol form: 3:1

keto **^{1}H-NMR** (400 MHz, $CDCl_3$): δ = 7.67 (1H, d, *J* = 16.0 Hz, H-5), 7.58 - 7. 51 (2H, m, ArH), 7.42 - 7.32 (3H, m, ArH), 6.86 (1H, d, *J* = 16.0 Hz, H-4), 4.33 - 4.17 (2H, m, H-13), 3.83 (1H, q, *J* = 7.1 Hz, H-2), 1.44 (3H, d, *J* = 7.1 Hz, H-12), 1.30 - 1.20 (3H, m, H-13) ppm.

^{13}C-NMR (100 MHz, $CDCl_3$): δ = 195.0 (C-3), 170.8 (C-1), 144.3 (C-5), 134.4 (C-6), 131.0 (C-4), 129.1 (C-8/10), 128.7 (C-7/11), 123.9 (C-9), 61.5 (C-13), 51.8 (C-2), 14.3 (C-12), 13.2 (C-14) ppm.
enol **^{1}H-NMR** (400 MHz, $CDCl_3$): δ = 12.71 (1H, d, *J* =1.6 Hz, OH), 7.58 - 7. 51 (2H, m, ArH), 7.45 (1H, d, *J* =15.7 Hz, H-5), 7.42 - 7.32 (3H, m, ArH), 6.95 (1H, dd, *J* = 15.7, 1.6 Hz, H-4), 4.33 - 4.17 (2H, m, H-13), 1.95 (3H, s, 2-CH3), 1.34 (3H, t, *J* =7.1 Hz, H-14) ppm.
ESI-MS (*m/z*): 233.1 $[M + H]^+$, 255.3 $[M + Na]^+$.

Ethyl *E*-2-(2-styryl-1,3-dioxolan-2-yl)acetate 43a

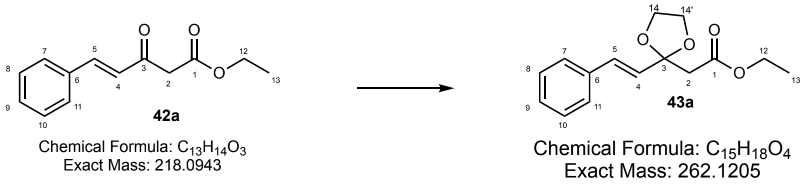

The β-keto ethyl ester **42a** (1.0 eq., 80 mg, 0.37 mmol) was dissolved in CH_2Cl_2 (2 mL) and *p*-TsOH·H_2O (0.6 eq., 42 mg, 0.22 mmol), ethylene glycol (10 eq., 0.2 mL, 3.66 mmol) and $(CH_3O)_3CH$ (5 eq., 0.2 mL, 1.83 mmol) were added. The reaction vial was then sealed, heated to 40 °C and stirred overnight. The solution was allowed to cool to room temperature before it was diluted with H_2O (3 mL). The phases were separated and the aqueous phase extracted with CH_2Cl_2 (3 × 2 mL). The combined organic phases were dried over $MgSO_4$, filtered and concentrated *in vacuo*. The crude residue was purified by flash chromatography (petroleum ether : ethyl acetate 3:1) to furnish **43a** as a colourless oil (71 mg, 0.27 mmol, 73%).

UV$_{\lambda max}$ (CH_3CN:H_2O): 205, 250 nm
^{1}H-NMR (400 MHz, $CDCl_3$): δ = 7.41 - 7.25 (5H, m, ArH), 6.76 (1H, d, *J* = 15.9 Hz, H-5), 6.26 (1H, d, *J* = 15.9 Hz, H-4), 4.16 (2H q, *J* = 7.1 Hz, H-12), 4.07 - 4.02 (2H, m, H-14), 4.01 - 3.95 (2H, m, H-14'), 2.87 (2H, s, H-2), 1.24 (3H, t, *J* = 7.1 Hz, H-13) ppm.
^{13}C-NMR (100 MHz, $CDCl_3$): δ = 169.0 (C-1), 136.1 (C-6), 130.9 (C-5), 128.7 (C-8/10), 128.2 (C-9), 128.0 (C-4), 127.0 (C-7/11), 106.8 (C-3), 65.1 (C-14/14'), 60.8 (C-12), 44.6 (C-2), 14.4 (C-13) ppm.
ESI-MS (*m/z*): 263.5 $[M + H]^+$, 285.3 $[M + Na]^+$.
HRESIMS (*m/z*): calculated for $C_{15}H_{18}O_4Na$ $[M + Na]^+$: 285.1097 found 285.1102

Ethyl *E*-2-(2-styryl-1,3-dioxolan-2-yl)propanoate 43b

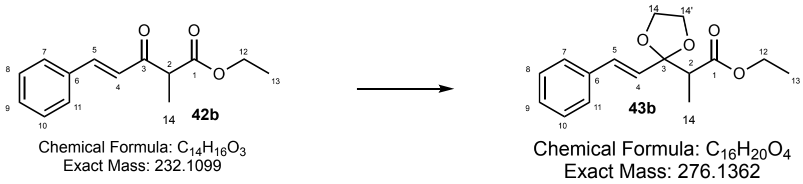

The β-keto ethyl ester **42b** (1.0 eq., 100 mg, 0.43 mmol) was dissolved in CH_2Cl_2 (3 mL) and *p*-TsOH·H_2O (0.6 eq., 49 mg, 0.26 mmol), ethylene glycol (10 eq., 0.24 mL, 4.30 mmol) and $(CH_3O)_3CH$ (5 eq., mL, 2.15 mmol) were added. The reaction vial was then sealed, heated to 40 °C and stirred for up to two days, monitored by TLC. The solution was allowed to cool to room temperature, before it was diluted with H_2O (3 mL). The phases were separated and the aqueous phase extracted with CH_2Cl_2 (3

× 3 mL). The combined organic phases were dried over $MgSO_4$, filtered and concentrated in *vacuo*. The crude residue was purified by flash chromatography (ethyl acetate/petroleum ether 3:1) to furnish **43b** as a colourless oil (77 mg, 0.28 mmol, 65%).

UV$_{\lambda max}$ (CH_3CN:H_2O): 207, 249 nm
^{1}H-NMR (400 MHz, $CDCl_3$): δ = 8.42 - 7.23 (5H, m, ArH), 6.72 (1H, d, *J* = 15.9 Hz, H-5), 6.22 (1H, d, *J* = 15.9 Hz, H-4), 4.15 (2H, q, *J* = 7.1 Hz, H-12), 4.08 - 3.90 (4H, m, H-15/15'), 2.92 (1H, q, *J* = 7.1 Hz, H-2), 1.25 (3H, d, *J* = 7.1 Hz, H-12), 1.23 (3H, t, *J* = 7.2 Hz, H-14) ppm.
^{13}C-NMR (100 MHz, $CDCl_3$): δ = 172.9 (C-1), 136.2 (C-6), 131.5 (C-5), 128.7 (C-8/10), 128.1 (C-9), 127.0 (C-7/11), 127.0 (C-4), 108.9 (C-3), 65.3 (C-15), 64.9 (C-15'), 60.6 (C-13), 48.3 (C-2), 14.4 (C-14), 12.5 (C-12) ppm.
ESI-MS (*m/z*): 277.2 $[M + H]^+$, 299.3 $[M + Na]^+$.
HRESIMS (*m/z*): calculated for $C_{16}H_{20}O_4Na$ $[M + Na]^+$: 299.1254 found 299.1257

S-(2-Acetamidoethyl) *E*-3-oxo-5-phenylpent-4-enethioate 16a

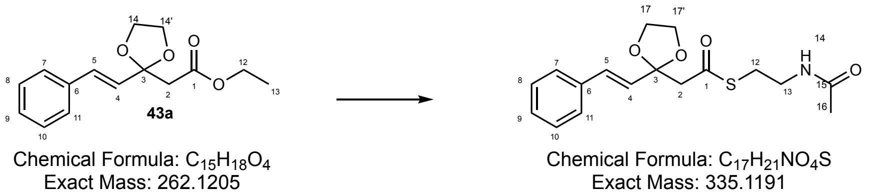

Ethyl *E*-2-(2-styryl-1,3-dioxolan-2-yl)acetate **43a** (1.0 eq., 50 mg, 0.19 mmol) was dissolved in THF (1 mL) to this solution was added $LiOH{\cdot}H_2O$ (4.0 eq., 32 mg, 0.76 mmol) dissolved in H_2O (1 mL). The reaction mixture was heated to 40 °C and vigorously stirred until completion. The mixture was diluted with H_2O (2 mL) and acidified to a pH of 3 with aq. HCl (1M). The aqueous solution was extracted with CH_2Cl_2 (2 × 2 mL), dried over $MgSO_4$, filtered and concentrated *in vacuo*. The crude residue was dried under high vacuum prior dissolving it in CH_2Cl_2 (2 mL). The solution was cooled to 0 °C and EDCl (2.0 eq, 72 mg, 0.38 mmol) and DMAP (0.8 eq., 18 mg, 0.15 mmol) were added. The solution was allowed to stir for 10 min before HSNAC **29** (1.1 eq., 25 mg, 0.21 mmol) was added. The mixture was allowed to stir and warm to room temperature overnight before it was quenched with the addition of H_2O (2 mL). The phases were separated, and the aqueous phase was extracted with CH_2Cl_2 (3 × 2 mL). The combined organic phases were dried over $MgSO_4$, filtered and concentrated *in vacuo*. The crude residue was purified by flash chromatography (petroleum ether/ethyl acetate 3:1 to 1:1) topped with a 2-3 cm silica layer with immobilized $CuSO_4$ (to remove unreacted HSNAC) to furnish a colourless oil (55 mg, 0.16 mmol, 84%).

UV$_{\lambda max}$ (CH_3CN:H_2O): 205, 247 nm
^{1}H-NMR (400 MHz, $CDCl_3$): δ = 7.41 - 7.27 (5H, m, ArH), 6.74 (1H, d, *J* = 15.9 Hz, H-5), 6.19 (1H, d, *J* = 15.9 Hz, H-4), 5.81 (1H, brs, H-14), 4.08 - 3.96 (4H, m, H-17 & H-17'), 3.41 (2H, dd, *J* = 5.9 Hz, H-13), 3.11 (2H, s, H-2), 3.04 (2H, dd, *J* = 6.8, 5.9 Hz, H-12), 1.89 (3H, s, H-16) ppm.
^{13}C-NMR (100 MHz, $CDCl_3$): δ = 195.2 (C-1), 170.8 (C-15), 135.8 (C-6), 131.3 (C-5), 129.1 (C-9), 128.9 (C-8/10), 128.5 (C-4), 127.9 (C-7/11), 106.7 (C-3), 65.1 (C-17/17'), 52.8 (C-2), 39.9 (C-12), 29.0 (C-12), 23.1 (C-16) ppm.
ESI-MS (*m/z*): 336.2 $[M + H]^+$, 358.2 $[M + Na]^+$
HRESIMS (*m/z*): calculated for $C_{17}H_{21}NO_4SNa$ $[M + Na]^+$: 358.1083 found 358.1083

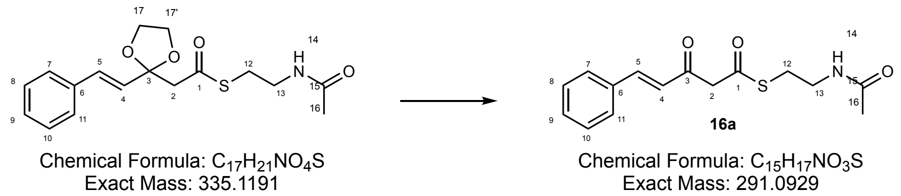

In a final step, the acetal intermediate prepared above (1.0 eq., 10 mg, 0.03 mmol) was deprotected in CH_2Cl_2 (1 mL) with the addition of TFA (5 drops) and H_2O (50 µL). The solution was vigorously stirred, and the reaction progress was checked with LCMS. The reaction was diluted with the addition of H_2O (3 mL), when there was no starting material left. The phases were separated, and the aqueous phase was extracted with CH_2Cl_2 (2 × 2 ml). The combined organic layers were washed with brine, dried over $MgSO_4$, filtered and the solvent was removed under nitrogen flow. The residue was dried under high vacuum to furnish **16a** (5.3 mg, 0.02 mmol, 60%) as a yellow solid. Enol : Keto form: 3:1

UV$_{\lambda max}$ (CH_3CN:H_2O): 207, 249 nm

enol **^{1}H-NMR** (400 MHz, $CDCl_3$): δ = 12.40 (1H, d, *J* = 1.5 Hz, OH), 7.66 - 7.33 (5H, m, ArH), 7.52 (1H, d, *J* = 15.8 Hz, H-5), 6.37 (1H, d, *J* = 15.8 Hz, H-4), 6.03 (1H, brs, H-14), 5.62 (1H, s, H-2), 3.54 - 3.48 (2H, m, H-13), 3.15 - 3.11 (2H, m, H-12), 2.01 (3H, s, H-16) ppm.

^{13}C-NMR (100 MHz, $CDCl_3$): δ = 194.4 (C-1), 170.3 (C-15), 167.1 (C-3), 139.2 (C-5), 135.0 (C-6), 129.8 (C-9), 128.9 (C-8/10), 127.7 (C-7/11), 121.0 (C-4), 101.3 (C-2), 39.9 (C-13), 28.0 (C-12), 23.2 (C-16),

keto **^{1}H-NMR** (400 MHz, $CDCl_3$): δ = 7.66 - 7.33 (5H, m, ArH), 7.61 (1H, d, *J* =16.1 Hz, H-5), 6.80 (1H, d, *J* =16.1 Hz, H-4), 6.03 (1H, brs, H-14), 3.97 (2H, s, H-2), 3.54 - 3.48 (2H, m, H-13), 3.13 (2H, m, H-12), 1.99 (3H, s, H-16) ppm.

^{13}C-NMR (100 MHz, $CDCl_3$): δ = 192.5 (C-1), 191.3 (C-3), 170.5 (C-15), 145.4 (C-5), 133.8 (C-6), 131.2 (C-9), 129.1(C-8/10), 128.6 (C-7/11), 124.9 (C-4), 55.6 (C-2), 39.2 (C-13), 29.3 (C-12), 23.1 (C-16) ppm.

ESI-MS (*m/z*): 292.2 $[M + H]^+$, 314.2 $[M + Na]^+$.

HRESIMS (*m/z*): calculated for $C_{15}H_{17}NO_3SNa$ $[M + Na]^+$: 314.0821 found 314.0825

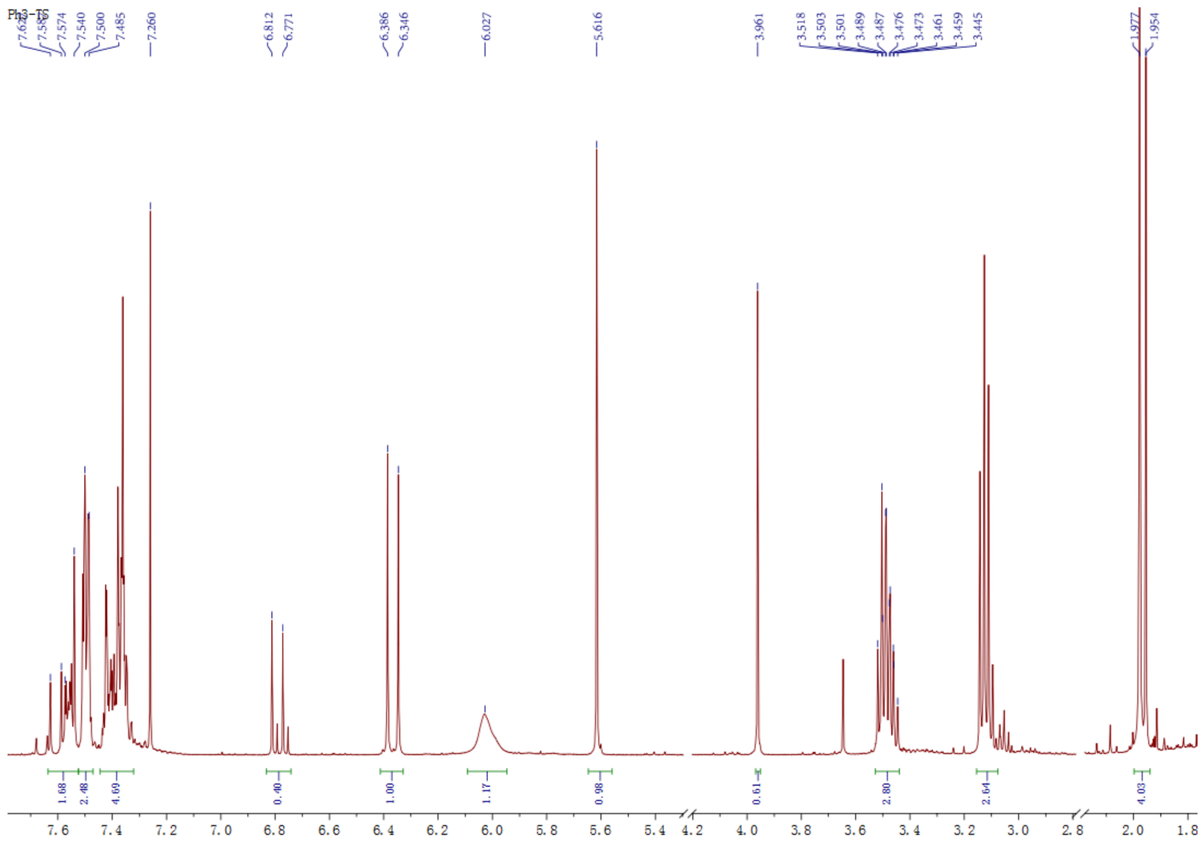

Figure S3.3.9A ^{1}H NMR of **16a** in $CDCl_3$. Mixture of keto and enol forms.

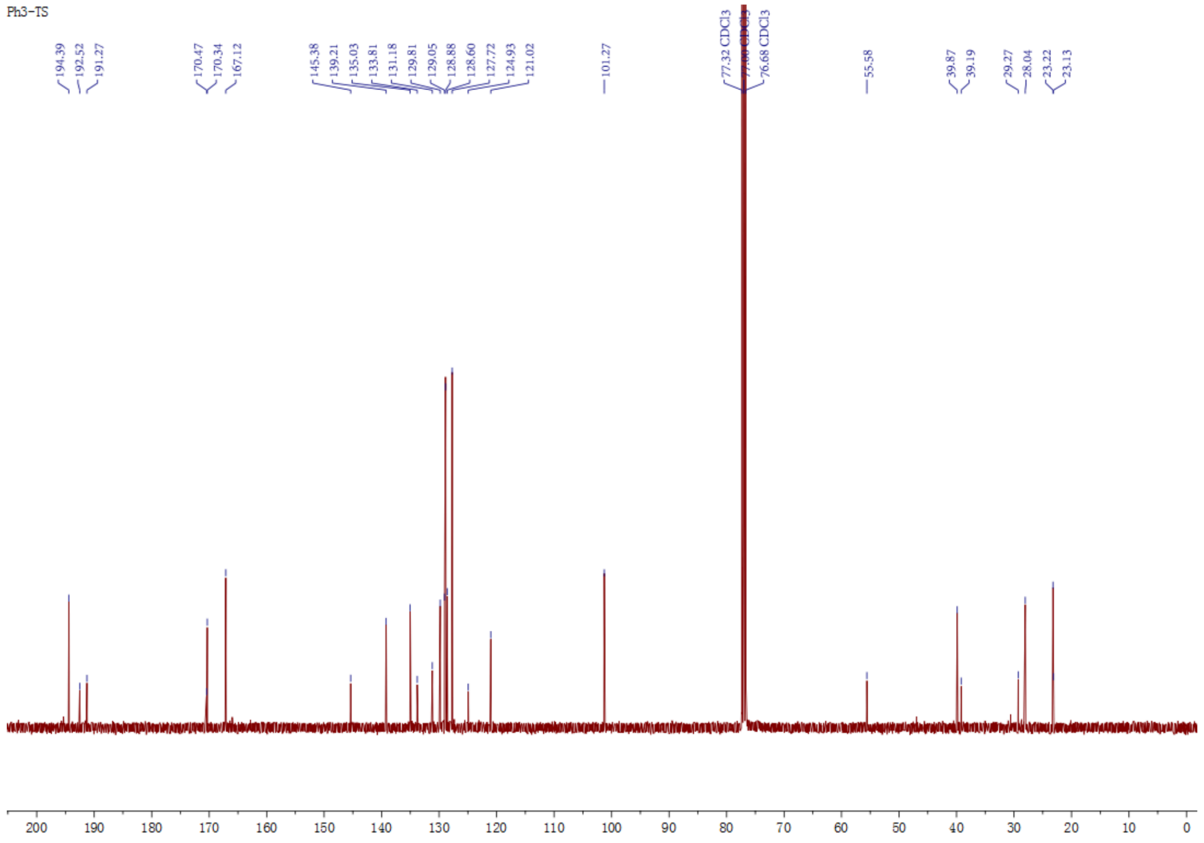

Figure S3.3.9B ^{13}C NMR of **16a** in $CDCl_3$. Mixture of keto and enol forms.

S-(2-Acetamidoethyl) *E*-2-methyl-3-oxo-5-phenylpent-4-enethioate 16b

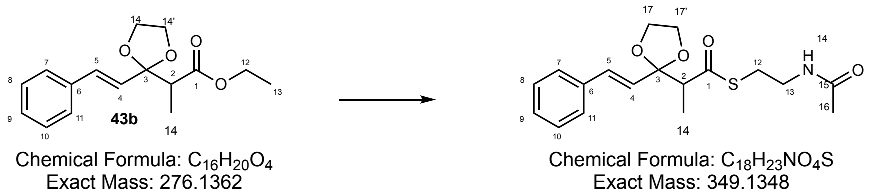

Ethyl *E*-2-(2-styryl-1,3-dioxolan-2-yl)propanoate **43b** (1.0 eq., 70 mg, 0.25 mmol) was dissolved in THF (1.5 mL) to this solution was added LiOH·H2O (4.0 eq., 42 mg, 1.00 mmol) dissolved in H_2O (1.5 mL). The reaction mixture was heated to 60 °C and vigorously stirred for up to two days. The mixture was diluted with H_2O (2 mL) and acidified to a pH of 3 with aq. HCl (1M). The aqueous solution was extracted with CH_2Cl_2 (2 × 2 mL), dried over $MgSO_4$, filtered and concentrated *in vacuo*. The crude residue was dried under high vacuum prior dissolving it in CH_2Cl_2 (2 mL). The solution was cooled to 0 °C and EDCl (2.0 eq, 72 mg, 0.5 mmol) and DMAP (0.3 eq., 18 mg, 0.08 mmol) were added. The solution was allowed to stir for 10 min before HSNAC **29** (1.1 eq., 33 mg, 0.28 mmol) was added. The mixture was allowed to stir and warm to room temperature overnight, before it was quenched with the addition of H_2O (2 mL). The phases were separated, and the aqueous phase was extracted with CH_2Cl_2 (3 × 2 mL). The combined organic phases were dried over $MgSO_4$, filtered and concentrated in *vacuo*. The crude residue was purified by flash chromatography (petroleum ether/ethyl acetate 3:1 to 1:1) topped with a 2-3 cm silica layer with immobilized $CuSO_4$ (to remove unreacted HSNAC) to furnish the protected SNAC as a colourless oil (61 mg, 0.17 mmol, 68%).

UV$_{\lambda max}$ (CH_3CN:H_2O): 207, 249 nm

^{1}H-NMR (400 MHz, $CDCl_3$): δ = 7.56 - 7.17 (5h, m, ArH), 6.70 (1H, d, *J* = 15.9 Hz, H-5), 6.16 (1H, d, *J* = 15.9 Hz, H-4), 5.79 (1H, brs, H-15), 4.06 - 3.92 (4H, m, H-18 & H-18’), 3.45 - 3.33 (2H, m, H-14), 3.15 (1H, q, *J* = 7.1 Hz, H-2), 3.11 - 2.87 (2H, m, H-13), 1.88 (3H, s, H-17), 1.27 (3H, d, *J* = 7.1 Hz, H-12) ppm.

^{13}C-NMR (100 MHz, $CDCl_3$): δ = 200.3 (C-1), 170.7 (C-16), 135.9 (C-6), 131.8 (C-5), 128.9 (C-8/10), 128.4 (C-4), 127.0 (C-7/11), 126.6 (C-9), 108.6 (C-3), 65.4 (C-18), 64.9 (C-18’), 56.3 (C-2), 39.9 (C-14), 28.8 (C-13), 23.1 (C-17), 13.1 (C-12).

ESI-MS (*m/z*): 350.3 $[M + H]^+$, 372.3 $[M + Na]^+$

HRESIMS (*m/z*): calculated for $C_{18}H_{23}NO_4S$ $[M + Na]^+$: 372.1240 found 372.1237

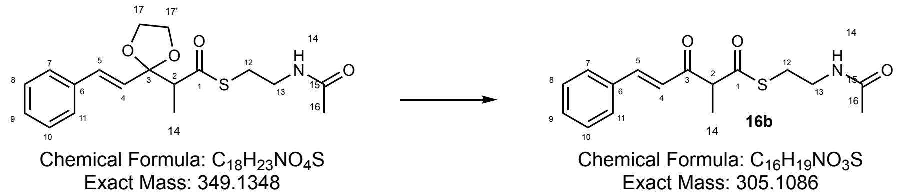

In a final step, the protected SNAC as prepared above (1.0 eq., 30 mg, 0.09 mmol) was deprotected in CH_2Cl_2 (1.5 mL) with the addition of TFA (5 drops) and H_2O (100 µL). The solution was vigorously stirred, and the reaction progress was checked with LCMS. The reaction was diluted with the addition of H_2O (3 mL), when there was no starting material left. The phases were separated, and the aqueous phase was extracted with CH_2Cl_2 (2 × 2 ml). The combined organic layers were washed with brine, dried over $MgSO_4$, filtered and the solvent was removed under nitrogen flow. The residue was dried under high vacuum to furnish **16b** (17 mg, 0.06 mmol, 65%) as a pale yellow solid.

Enol:Keto form: 2:3

UV$_{\lambda max}$ (CH_3CN:H_2O): keto: 223, 300 nm enol: 221, 357 nm

Keto: ^{1}H-NMR (400 MHz, $CDCl_3$): δ = δ 7.69 (1H, d, *J* = 16.0 Hz, H-5), 7.59 – 7.52 (2H, m, ArH), 7.45 – 7.29 (3H, m, ArH), 6.87 (1H, d, *J* = 15.9 Hz, H-4), 4.11 (1H, q, *J* = 7.1 Hz, H-2), 3.48 – 3.40 (2H, m, H-14), 3.14 – 3.04 (2H, m, H-13), 1.92 (3H, s, H-17), 1.49 (3H, d, *J* = 7.1 Hz, H-12) ppm.

^{13}C-NMR (100 MHz, $CDCl_3$): δ = 197.3 (C-1), 194.2 (C-3), 172.1 (C-15), 145.3 (C-6), 134.1 (C-5), 129.2 (C-8/10), 128.4 (C-4), 128.8 (C-7/11), 123.4 (C-9), 60.0 (C-2), 40.0 (C-13), 28.8 (C-12), 22.9 (C-16), 14.0 (C-12) ppm.

Enol : ^{1}H NMR (400 MHz, $CDCl_3$) δ 13.43 (1H, d, *J* = 1.7 Hz, OH), 7.66 – 7.51 (3H, m, ArH & H-5), 7.48 – 7.30 (3H, m, ArH), 6.86 (1H, d, *J* = 15.6 Hz, H-4), 6.30 (1H, s, H-15), 3.50 – 3.34 (2H, m, H-12), 3.06 (2H, m, H-13), 2.08 (3H, s, H-16), 2.04 (3H, s, H-17).

^{13}C-NMR (100 MHz, $CDCl_3$): 197.9 (C-1), 179.3 (C-3), 164.6 (C-15), 139.7 (C-6), 135.7 (C-5), 129.9 (C-9), 129.1 (C-8/10), 128.0 (C-7/11), 118.8 (C-4), 105.9 (C-2), 40.6 (C-13), 28.3 (C-12), 22.9 (C-16), 11.8 (C-17).

ESI-MS (*m/z*): 306.3 $[M + H]^+$, 328.3 $[M + Na]^+$.

HRESIMS (*m/z*): calculated for $C_{16}H_{19}NO_3SNa$ $[M + Na]^+$: 328.0978 found 328.0994.

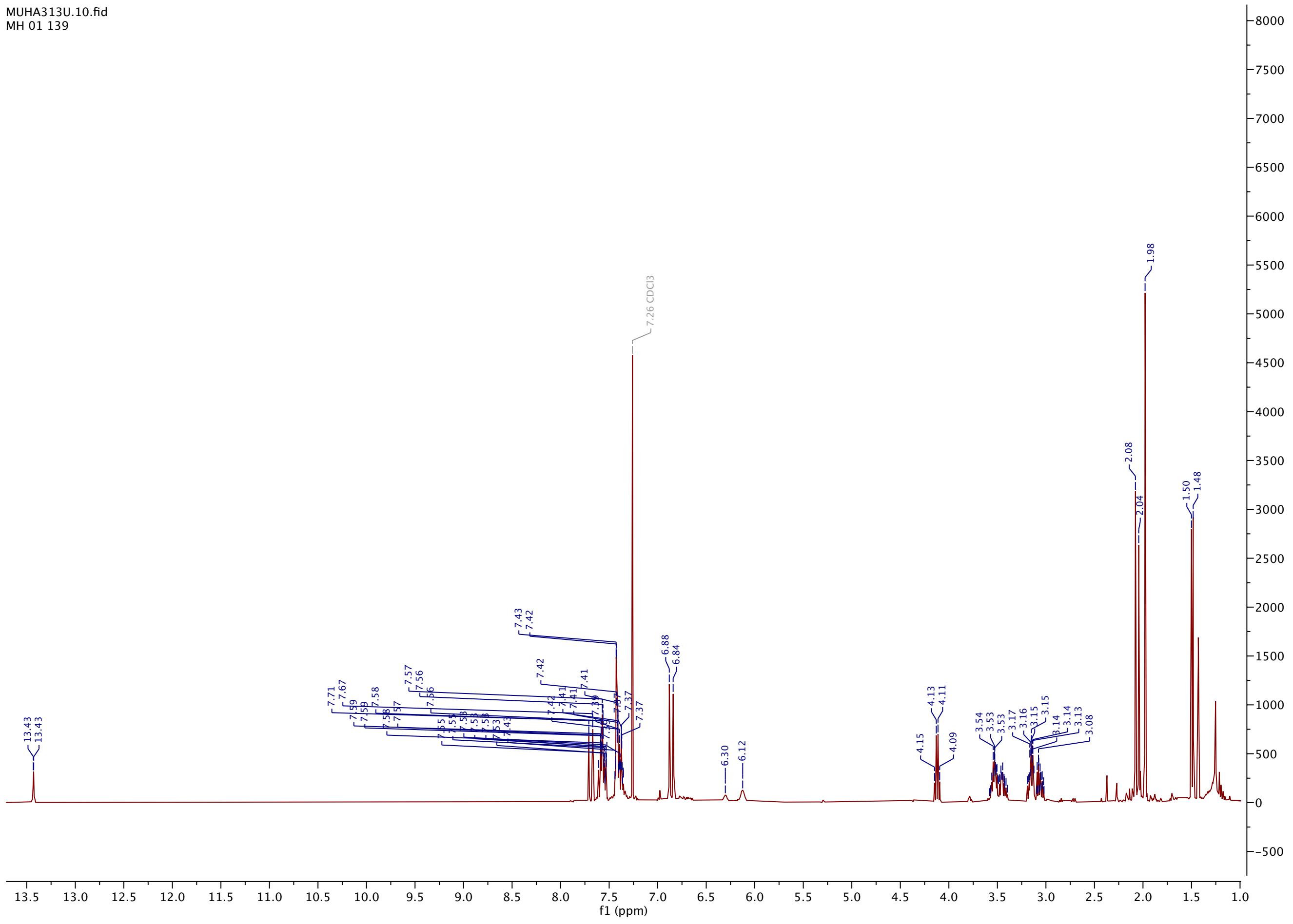

Figure S3.3.9C ^{1}H NMR of **16b** in $CDCl_3$. Mixture of keto and enol forms.

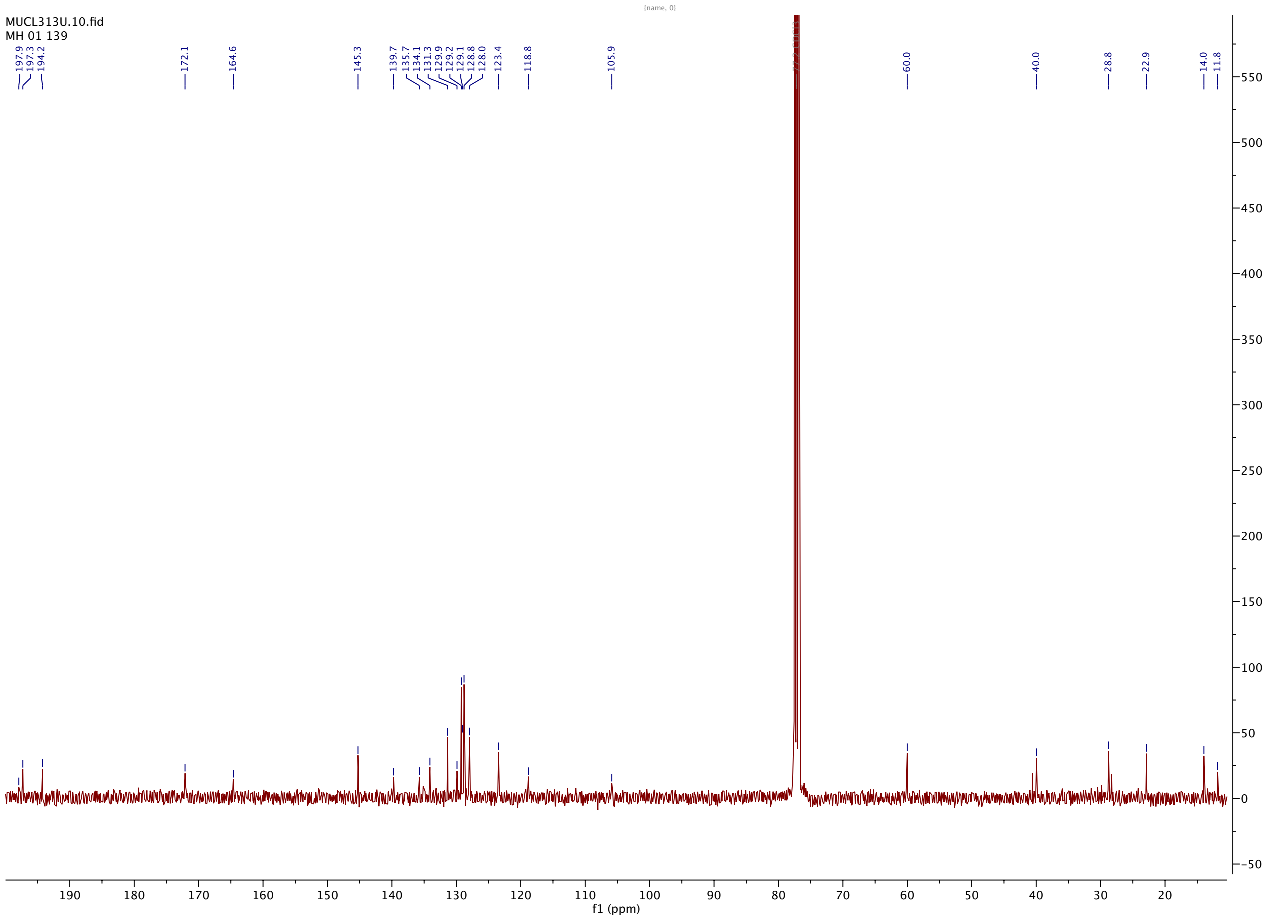

Figure S3.3.9D ^{13}C NMR of **16b** in $CDCl_3$. Mixture of keto and enol forms.

3.3.10 Compound 17b

***S*-(2-(3-((*R*)-2,2,5,5-Tetramethyl-1,3-dioxane-4-carboxamido)propanamido)ethyl) 2-(2-(*E*-styryl)-1,3-dioxolan-2-yl)propanethioate 44b**

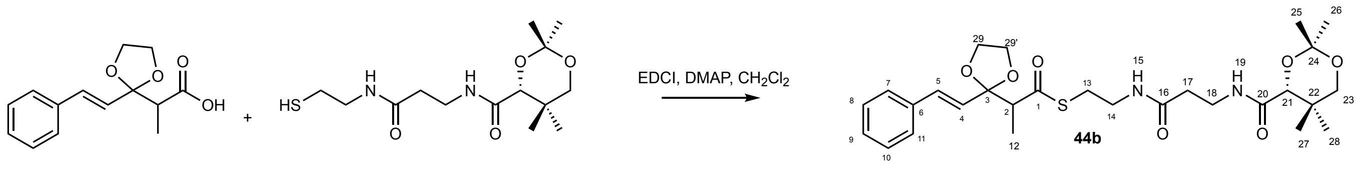

The acetal protected acid (see **16b**, 1.0 eq., 40 mg, 0.16 mmol) intermediate was dried under high vacuum prior dissolving it in CH_2Cl_2 (2 mL). The solution was cooled to 0 °C and EDCl (1.1 eq, 32 mg, 0.17 mmol) and DMAP (0.2 eq., 7 mg, 0.06 mmol) were added. The solution was allowed to stir for 10 min before pantetheine dimethyl ketal (prepared according to Roberts *et al*,[29] 1.2 eq., 61 mg, 0.19 mmol) was added. The mixture was allowed to stir and warm to room temperature overnight before it was quenched with the addition of H_2O (2 mL) and HCl (1M, 1 mL). The phases were separated, and the aqueous phase was extracted with CH_2Cl_2 (3 × 3 mL). The combined organic phases were washed with saturated $NaHCO_3$ (10 mL), dried over $MgSO_4$, filtered and concentrated *in vacuo*. The crude residue was purified by flash chromatography (ethyl acetate) to furnish fully protected **44b** as a white solid (68 mg, 0.12 mmol, 77%).

UV$_{\lambda max}$ (CH_3CN:H_2O): 205, 246 nm
^{1}H-NMR (400 MHz, $CDCl_3$): δ = 7.41 - 7.24 (5H, m, ArH), 7.02 (1H, brt, *J* = 6.2 Hz, H-19), 6.69 (1H, d, *J* = 15.9 Hz, H-5), 6.15 (1H, d, *J* = 15.9 Hz, H-4), 5.99 (1H, brd, *J* = 4.7 Hz, H-15), 4.05 (1H, s, H-21), 4.04 - 3.91 (4H, m, H-29 & H-29'), 3.66 (1H, d, *J* = 11.7 Hz, H-23a), 3.58 - 3.29 (4H, m, H-14 & H-18), 3.26 (1H, d, *J* = 11.7 Hz, H-23b), 3.13 (1H, q, *J* = 7.0 Hz, H-2), 3.04 - 2.95 (2H, m, H-13), 2.33 - 2.29 (2H, m, H-17), 1.44 (3H, s, H-25), 1.40 (3H, s, H-26), 1.26 (3H, d, *J* = 7.1 Hz, H-12), 1.02 (3H, s, H-27), 0.95 (3H, s, H-28) ppm.
^{13}C-NMR (100 MHz, $CDCl_3$): δ = 199.9 (C-1), 171.3 (C-20), 170.1 (C-16), 135.9 (C-5), 131.8 (C-6), 128.8 (C-8/10), 128.4 (C-9), 127.0 (C-7/11), 126.6 (C-4), 108.6 (C-24), 99.2 (C-3), 77.3 (C-21), 71.6 (C-23), 65.4 (C-29), 64.9 (C-29'), 56.3 (C-2), 39.5 (C-17), 35.8 (C-18), 33.1 (C-22), 22.3 (C-25), 19.0 (C-26), 19.0 (C-27), 18.8 (C-28), 13.1 (C-12) ppm.
ESI-MS (*m/z*): 549.6 $[M + H]^+$, 571.6 $[M + Na]^+$.
HRESIMS (*m/z*): calculated for $C_{28}H_{40}N_2O_7SNa$ $[M + Na]^+$: 571.2454 found 571.2463

***S*-(2-(3-((*R*)-2,4-Dihydroxy-3,3-dimethylbutanamido)propanamido)ethyl) *E*-2-methyl-3-oxo-5-phenylpent-4-enethioate 45b**

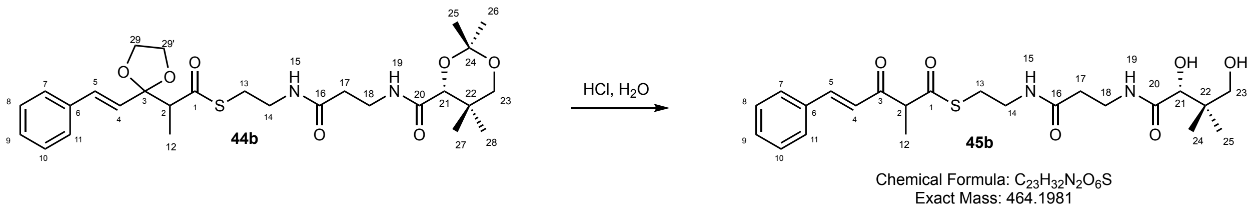

The protected intermediate **44b** (1.0 eq., 41 mg, mmol) was dissolved in THF (1 mL) and H_2O (1 mL) with the addition of conc. HCl (4-6 drops). The solution was vigorously stirred and heated to 40 °C and the reaction progress was checked by LCMS. The reaction was diluted with the addition of H_2O (3 mL), when there was no starting material left (~ 60 min). The phases were

separated, and the aqueous phase was extracted with CH_2Cl_2 (2 × 2 ml). The combined organic layers were washed with brine, dried over $MgSO_4$, filtered and the solvent was removed under nitrogen flow. The residue was dried under high vacuum to furnish the deprotected β-ketoacyl pantetheinyl **45b** (25 mg, mmol,%) as a pale yellow solid. 5:1 Keto : Enol

UV$_{\lambda max}$ (CH_3CN:H_2O): Enol: 224, 358 nm Keton: 224, 300 nm

Keto **^{1}H-NMR** (400 MHz, CD_3CN): δ = 7.69 - 7.65 (3H, m, ArH & H-5), 7.46 - 7.39 (3H, m, ArH), 7.26 (1H, brs, H-15), 6.94 (1H, d, *J* = 16.1 Hz, H-4), 6.63 (1H, brs, H-19), 4.25 (1H, q, *J* = 7.0 Hz, H-2), 3.92 (1H, d, *J* = 5.4 Hz, H-23a), 3.84 (1H, d, *J* = 5.4 Hz, H-23b), 3.45 - 3.27 (4H, m, H-14 & H-18), 3.02 - 2.99 (2H, m, H-13), 2.28 (2H, dd, *J* = 6.6 Hz, H-17), 1.38 (3H, d, *J* = 7.0 Hz, H-12), 0.90 (3H, s, H-25), 0.82 (3H, s, H-24) ppm.

Keto **^{13}C-NMR** (100 MHz, CD_3CN): δ = 197.6 (C-1), 195.3 (C-3), 174.1 (C-16), 172.2 (C-20), 144.9 (C-5), 135.3 (C-6), 131.9 (C-4), 129.1 (C-8/10), 129.6 (C-7/11), 125.4 (C-9), 77.7 (C-21), 70.8 (C-23), 68.3 (C-22), 60.2 (C-2), 40.0 (C-17), 39.3 (C-18), 36.1 (C-14), 35.8 (C-13), 21.7 (C-25), 20.6 (C-24), 13.2 (C-12) ppm.

Enol **^{1}H-NMR** (400 MHz, CD_3CN): δ = 13.51 (1H, d, *J* = 1.6 Hz, OH), 7.69 - 7.65 (2H, m, ArH), 7.55 (1H, d, *J* = 15.7 Hz, H-5) 7.46 - 7.39 (3H, m, ArH), 7.26 (1H, brs, H-15), 7.02 (1H, dd, *J* = 15.7, 1.6 Hz, H-4), 6.63 (1H, brs, H-19), 3.92 (1H, d, *J* = 5.4 Hz, H-23a), 3.84 (1H, d, *J* = 5.4 Hz, H-23b), 3.45 - 3.27 (4H, m, H-14 & H-18), 3.08 (2H, t, *J* = 6.5 Hz, H-13), 2.32 (2H, t-dd, *J* = 6.5 Hz, H-17), 2.08 (3H, s, H-12), 0.90 (3H, s, H-25), 0.83 (3H, s, H-24) ppm.

ESI-MS (*m/z*): 447.5 $[M - H_2O + H]^+$, 465.6 $[M + Na]^+$

HRESIMS (*m/z*): calculated for $C_{23}H_{32}N_2O_6SNa$ $[M + Na]^+$: 487.1873 found 487.1879

S-(2-(3-((*R*)-2,4-Di(hydroxy-*d*)-3,3-dimethylbutanamido-*d*)propanamido-*d*)ethyl) *E*-2-methyl-3-oxo-5-phenylpent-4-enethioate-2-*d* 17b

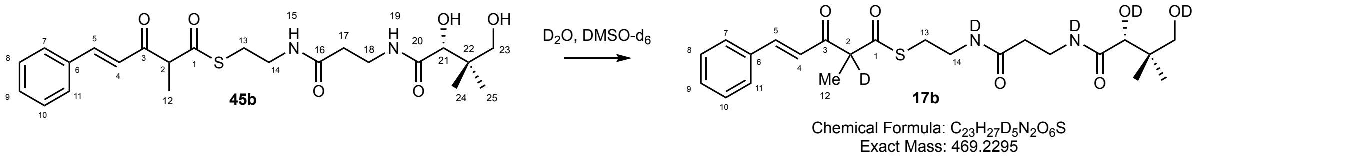

The deprotected pantetheine **45b** (23 mg, 0.049 mmol) was dissolved in DMSO-d6 (0.6 mL) and D_2O (0.6 mL) and transferred to an NMR tube. The deuteration was checked after 2h and 16h by obtaining ^{1}H NMR spectra. When there was no change visible, the solution was kept as stock solution for the NMR based deuterium exchange assay.

^{1}H-NMR (600 MHz, $(CD_3)_2SO/D_2O$ 1:1): δ = 7.63 - 7.60 (3H, m, H-5 & ArH), 7.41 - 7.38 (3H, m, ArH), 6.87 (1H, d, *J* = 16.1 Hz, H-4), 3.71 (1H, s, H-21), 3.58 - 3.53 (2H, m, H-23), 3.31 - 3.26 (1H, m, H-18), 3.23 - 3.12 (3H, m, H-14 & H-18), 2.90 (2H, dd, *J* = 6.7, 2.8 Hz, H-13), 2.21 (2H, dd, *J* = 6.7 Hz, H-17), 1.25 (3H, s, H-12), 0.72 (3H, s, H-24), 0.69 (3H, s, H-25) ppm.

^{13}C-NMR (150 MHz, $(CD_3)_2SO/D_2O$ 1:1): δ = 199.0 (C-1), 197.6 (C-3), 175.4 (C-16), 173.7 (C-20), 146.6 (C-5), 134.9 (C-6), 132.9 (C-4), 130.6 (C-8/10), 130.1 (C-7/11), 125.5 (C-9), 76.4 (C-21), 69.2 (C-23), 68.7 (C-22), 59 (brs, C-2), 40.0 (C-17), 39.9 (C-18), 36.1 (C-14), 36.0 (C-13), 26.3 (C-17), 22.0 (C-25), 20.9 (C-24), 14.8 (C-12) ppm.

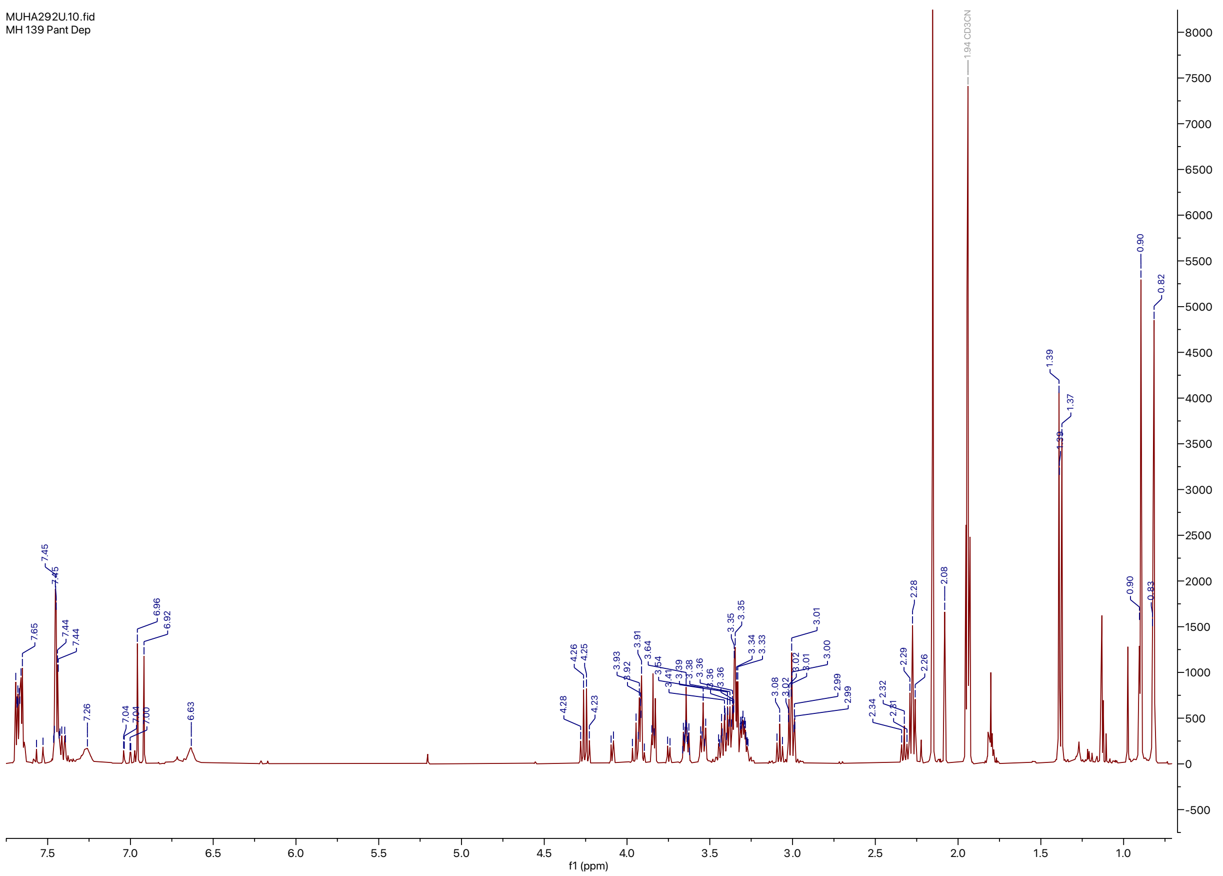

Figure S3.3.10A ^{1}H NMR of **17b** in CD_3CN.

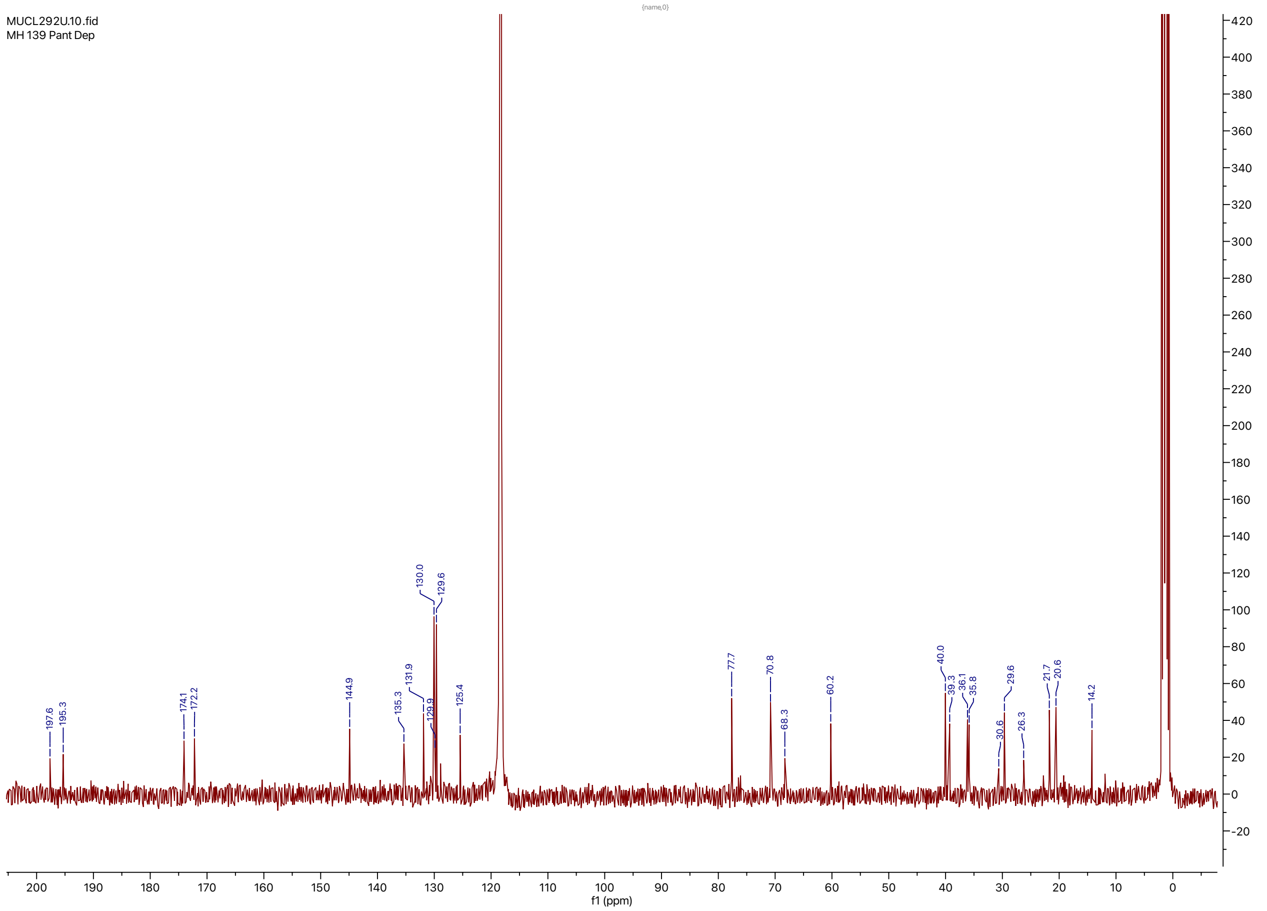

Figure S3.3.10B ^{13}C NMR of **17b** in CD_3CN.

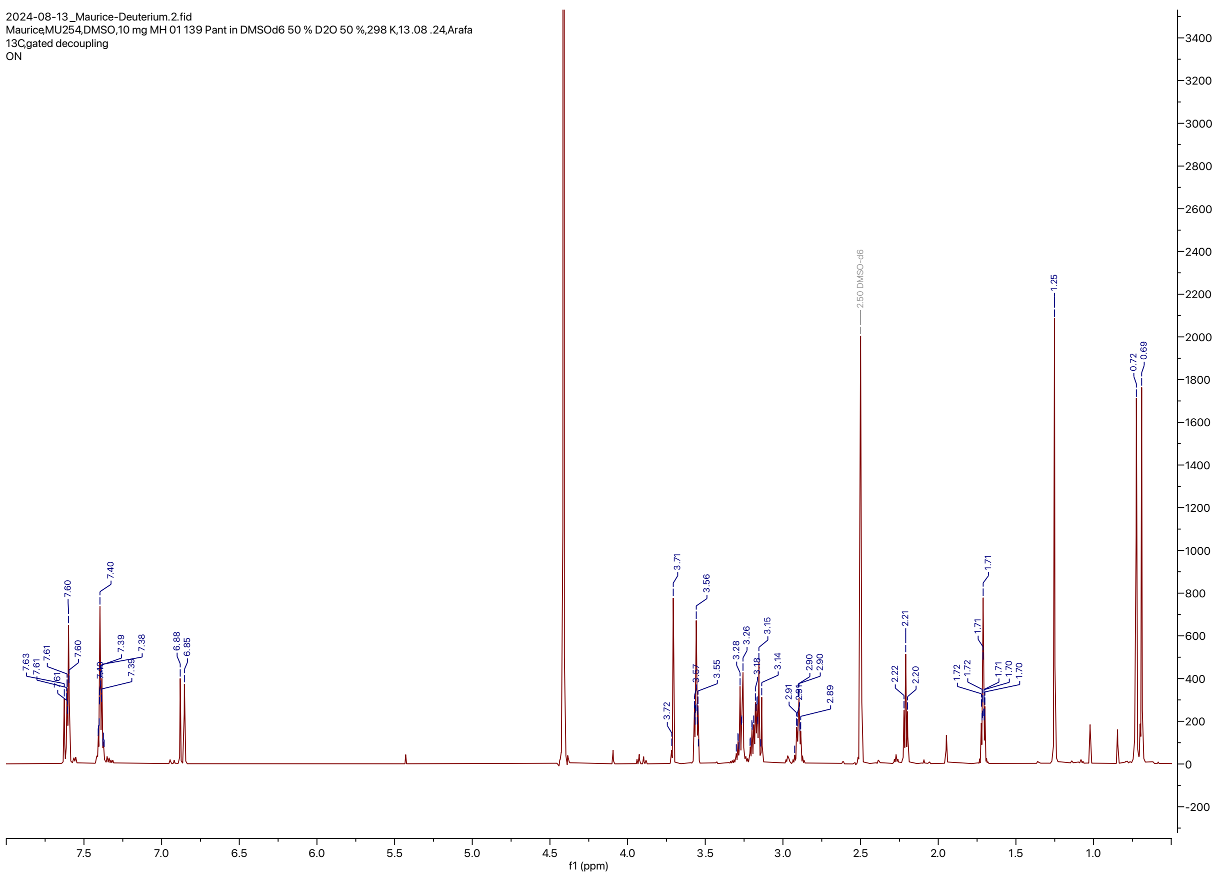

Figure S3.3.10C ^{1}H NMR of **17b** in DMSO-d_6 + D_2O.

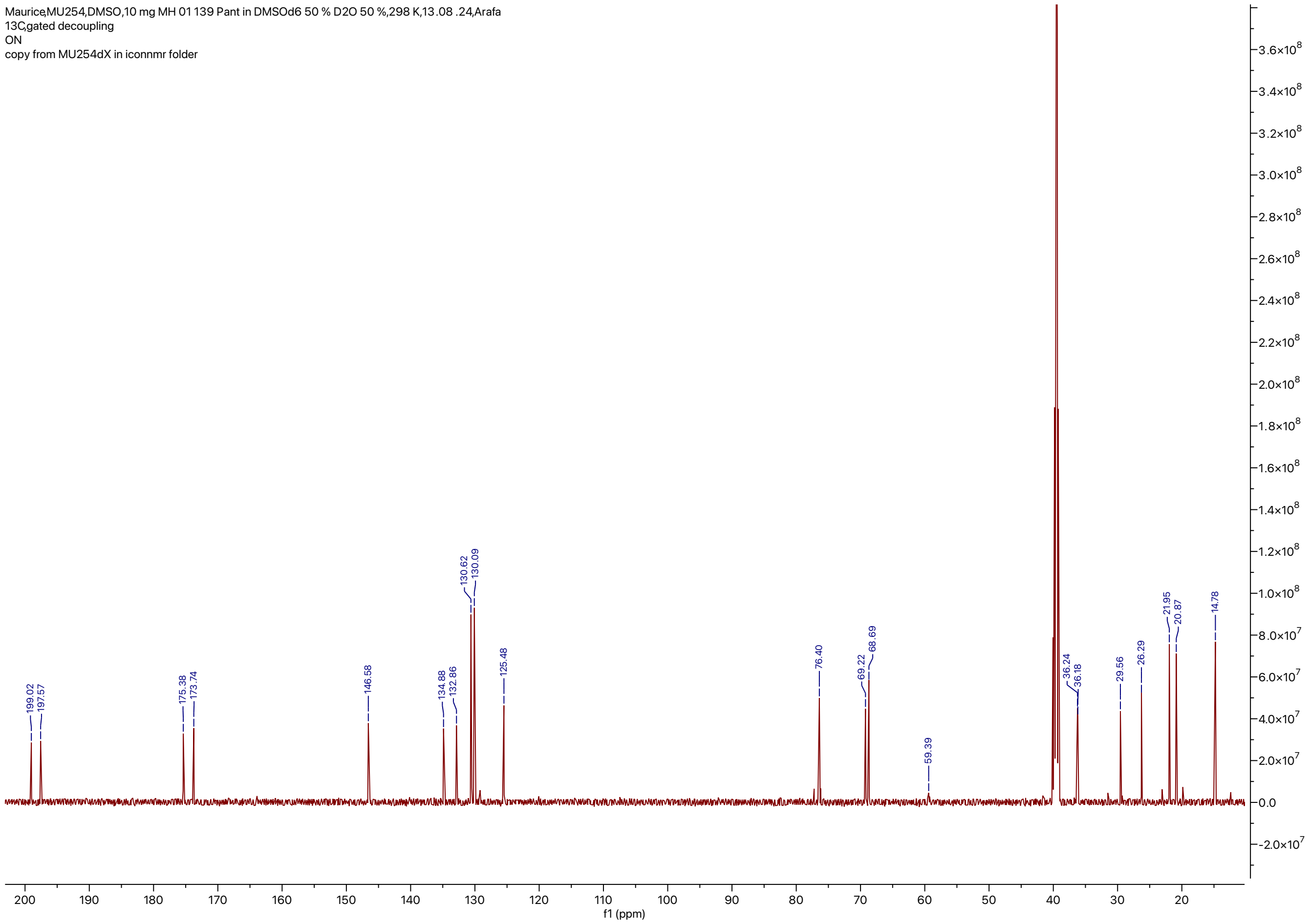

Figure S3.3.10D ^{13}C NMR of **17b** in DMSO-d_6 + D_2O.

HSNAC 29[30]

The preparation was followed as described by Keatinger-Clay *et al.*[30] Cysteamine hydrochloride (1.0 eq., 1.14 g, 10.0 mmol), KOH (1.0 eq., 0.56 g, 10.0 mmol) and $NaHCO_3$ (3.0 eq., 2.53 g, 30.0 mmol) were dissolved in water (50 mL). Acetic anhydride (1.0 eq., 0.95 mL, 10.0 mmol) was added dropwise and the resulting solution was stirred for 15 min at rt. The aqueous solution was then neutralised to a pH of 7 with HCl (10 M) and extracted with EtOAc (150 mL). The phases were separated, and the organic phase was dried over $MgSO_4$, filtered and concentrated in *vacuo* to give a clear oil (quant.), which was used without any further purification and stored under nitrogen in the freezer.

4. Analytical Assays of *in vitro* Enzyme Reactions

4.1 General Methods

See section 2.6 for details of enzyme assay conditions.

4.2 DH Assays

4.2.1 Data for Scheme 2A - racemic methyl diketides 7b

Racemic *syn*- and *anti*- diastereomers of **7b** were synthesised and separated by HPLC (Scheme S3.1.4). Analysis of the NMR data showed that the *syn* diastereomer contained *ca* 10 % of the *anti* diastereomer as an impurity, but the *anti* diastereomer appeared 100% pure.

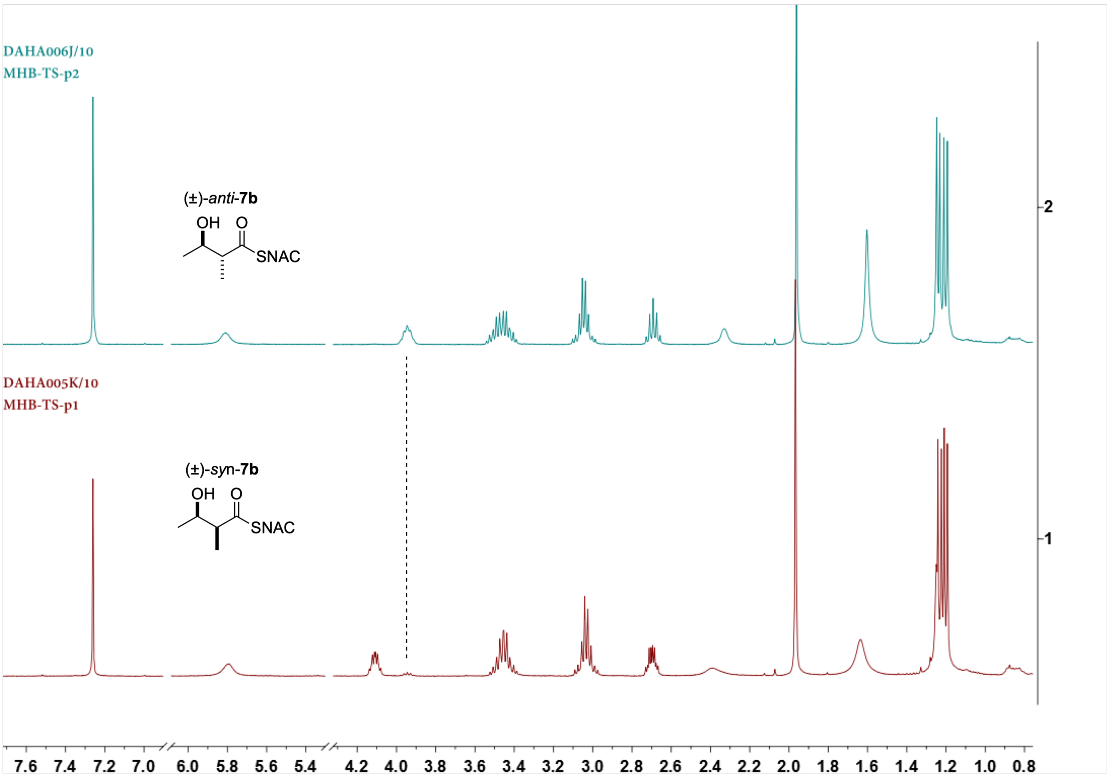

Figure S 4.2.1A. Comparison of ^{1}H NMR of purified (±)-*anti*- and (±)-*syn*-**7b** in $CDCl_3$ showing that the *syn* compound contains *ca* 10% *anti*.

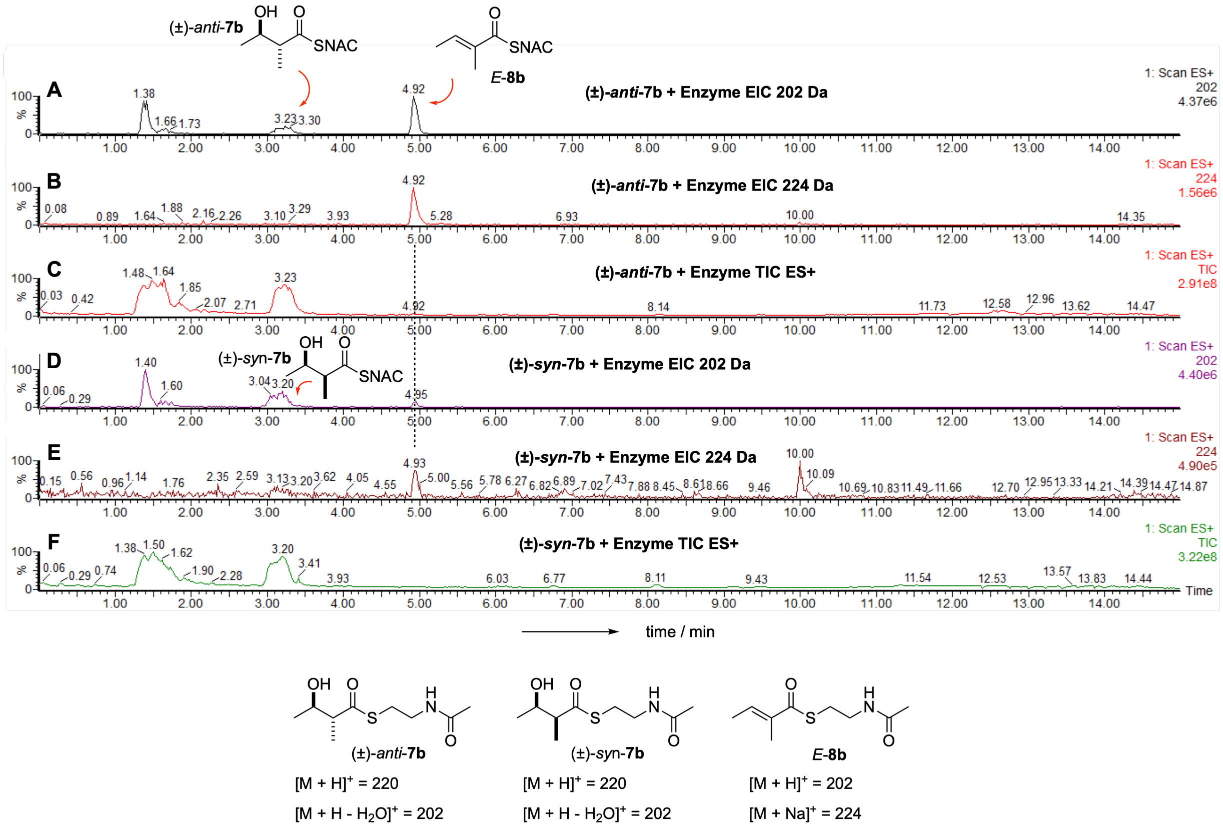

Figure S4.2.1B Incubation of (±)-*anti*- and (±)-*syn*-**7b** with strDH protein. The *syn* sample contans *ca* 10% *anti* and this is converted to **8b**. **A-C** reactions of (±)-*anti*-7b; **D-F** reactions of (±)-*syn*-7b. EIC = extracted ion chromatogram; TIC = total ion chromatogram.

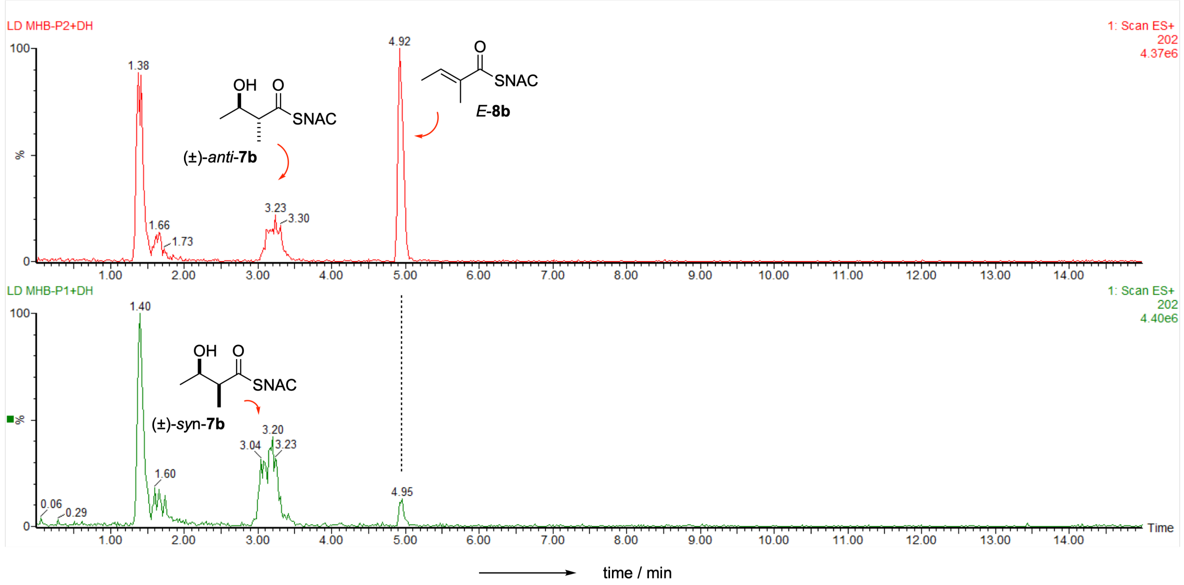

Figure S4.2.1C. Quantitative analysis of EIC data (202 Da, ES+) for assays of (±)-*anti* and (±)-*syn* diastereomers of **7b** with strDH protein. 202 Da corresponds to $[M + H]^+$ for *E*-**8b** and $[M + H - H_2O]^+$ for **7b**. Results show that the *anti*-contaminant in the *syn* sample is responsible for the formation of **8b** in the *syn* sample.

Determination of Stereochemistry of DH Product

DH products *E*-**8b** and *Z*-**8b** co-elute in all tested RP-HPLC conditions. However *E*-**8b** and *Z*-**8b** are easily distinguished by NMR. Therefore, in order to determine the geometry of the product of the strM-catalysed elimination of D-*anti*-**7b** (all other stereoisomers are not substrates) D-*anti*-**7b** was incubated with strM protein. At the end of the reaction CD_3CN was added to precipitate protein and the supernatant was examined directly by ^{1}H NMR.

Reactions were performed in a final volume of 350 µL, containing D-*anti*-**7b** (35 µl, 0.029 mg/µl dissolved in DMSO-d_6), StrM (10 mg/ml) in Tris-HCl buffer (50 mM Tris, 150 mM NaCl, pH 8.0). The reaction sample was incubated at 25 °C for 6 h. Acetonitrile-d_3 (300 µL) was added to precipitate the enzyme and quench the reaction. The suspension was then centrifuged at 14,000 × g for 15 minutes to remove the precipitated protein. A negative control was prepared identical using boiled enzyme. *E*- and *Z*- Standard **8** were similarly treated as described. *E*-**8** or *Z*-**8** (35 µL, 0.029 mg/µL dissolved in DMSO-d_6) were diluted in Tris-HCl buffer (50 mM Tris, 150 mM NaCl, pH 8.0) to a final volume of 350 µL. Incubated at 25°C for 6h and diluted with Acetonitrile-d_3 (300 µL). The resulting supernatants were then transferred to NMR tubes and ^{1}H NMR data were collected with WATERGATE water suppression. Results showed only the production of *E*-**8b**.

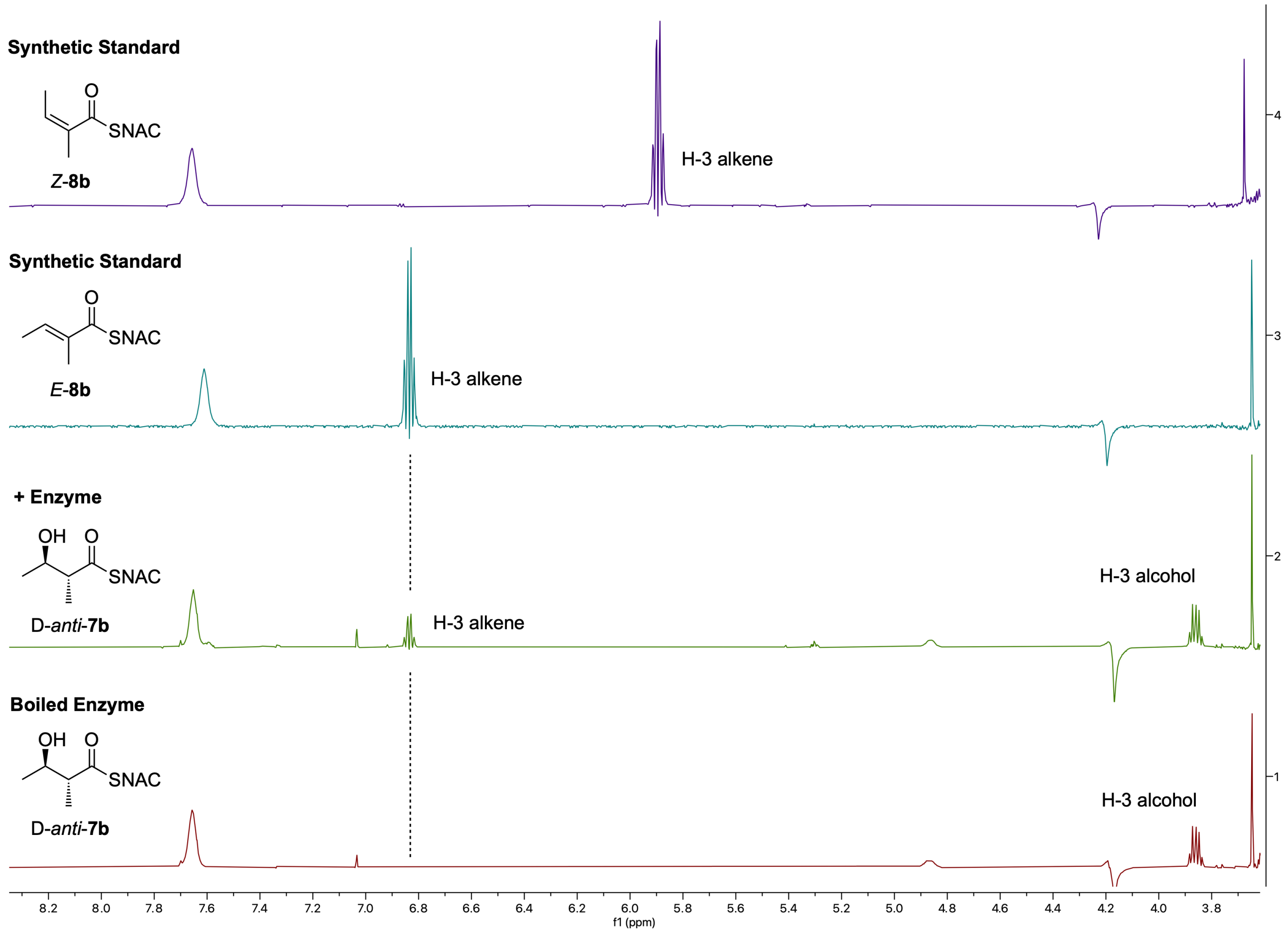

Figure S4.2.1D WATERGATE ^{1}H NMR analysis of the elimination reaction catalysed by strM using D-*anti*-**7b** as the substrate. Only *E*-**8b** was observed after 6h incubation. Solvent is as described above.

4.2.2 Data for Scheme 2B - enantiopure *anti*- and *syn*- diketides 7b

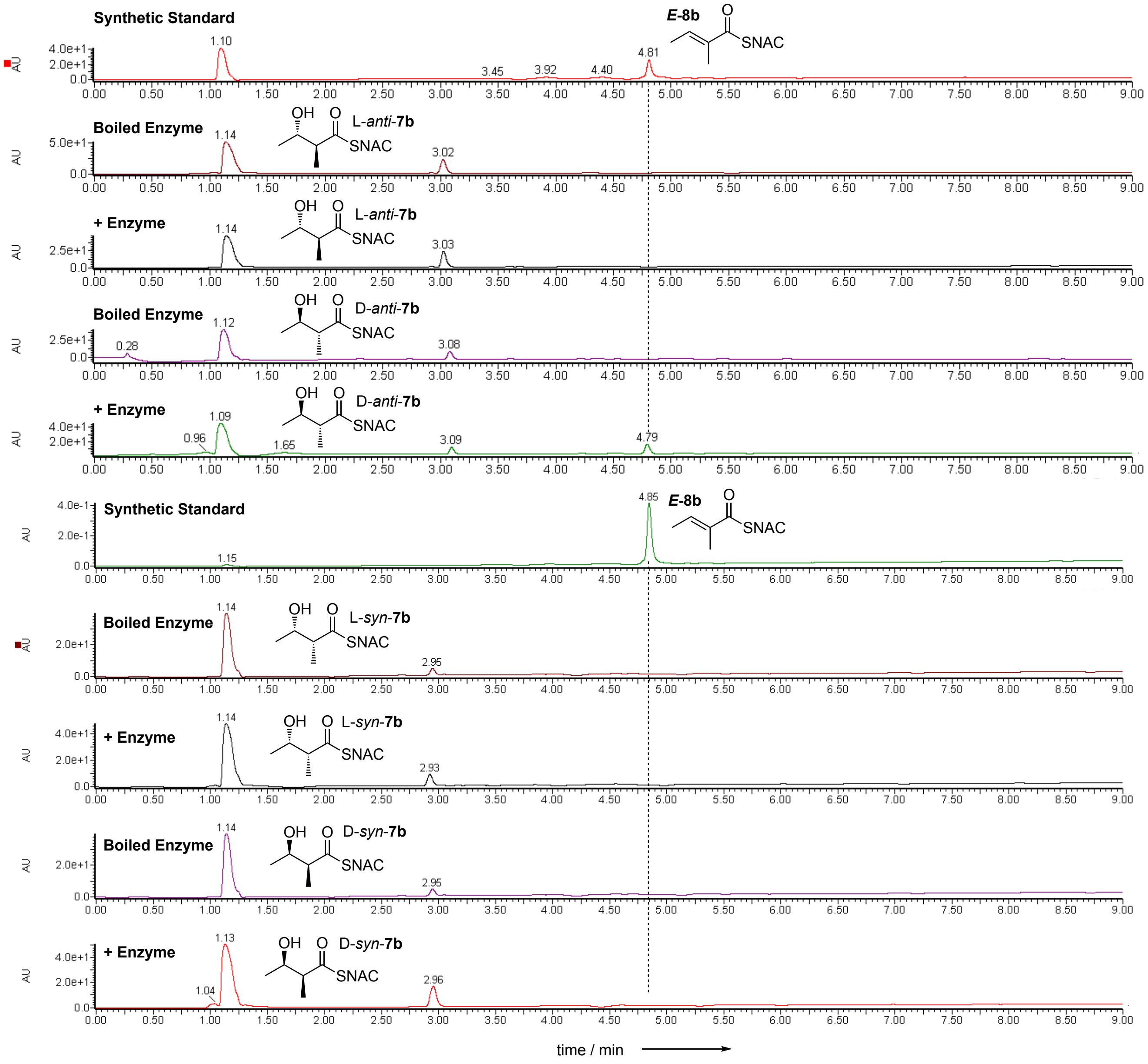

Figure S4.2.2. DAD data (200 - 600 nm) for diketide DH assay. Reactions of individual stereoisomers as shown with either active StrM protein or boiled protein.

4.2.3 Data for Scheme 2C - non methylated phenyl diketide enantiomers 9a

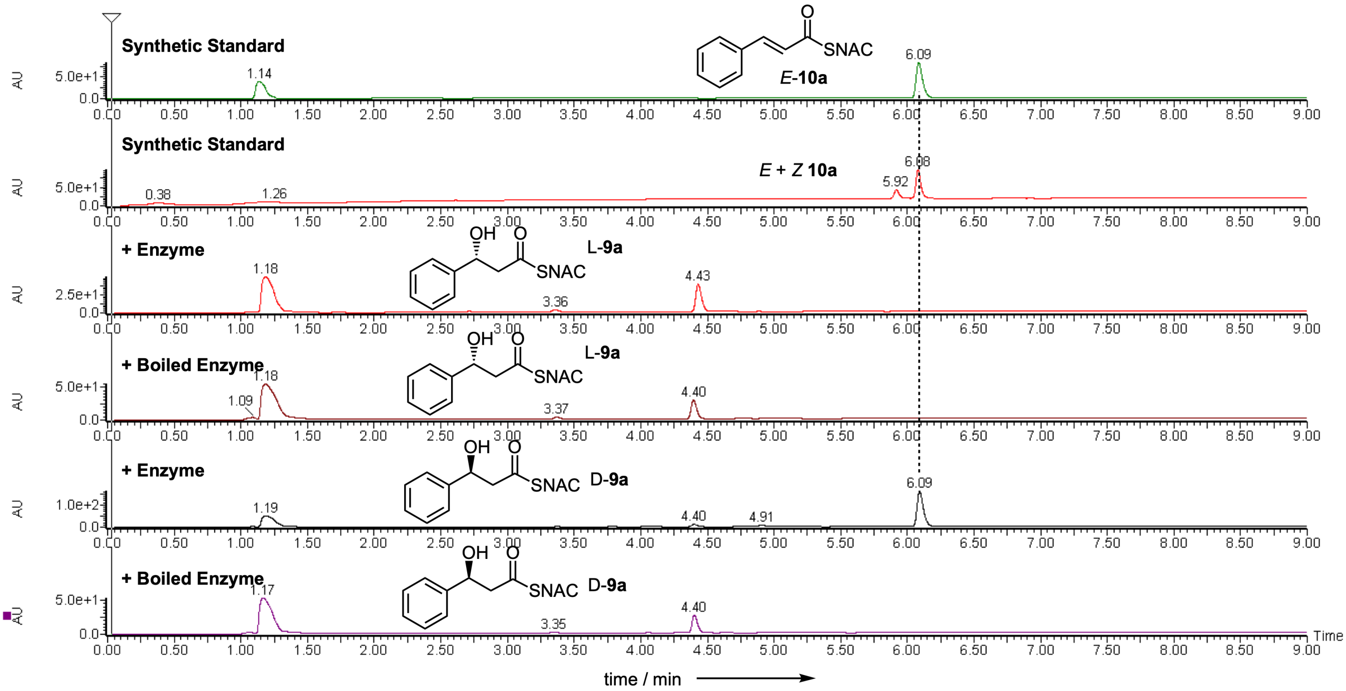

Figure S4.2.3. Elimination reactions catalysed by strPKS multidomain. From top, synthetic standards, reaction of L-enantiomer, reaction of D-enantiomer, (UV DAD chromatograms, 200 - 600 nm).

4.2.4 Data for Scheme 2D - Reaction of D-*anti*-9b

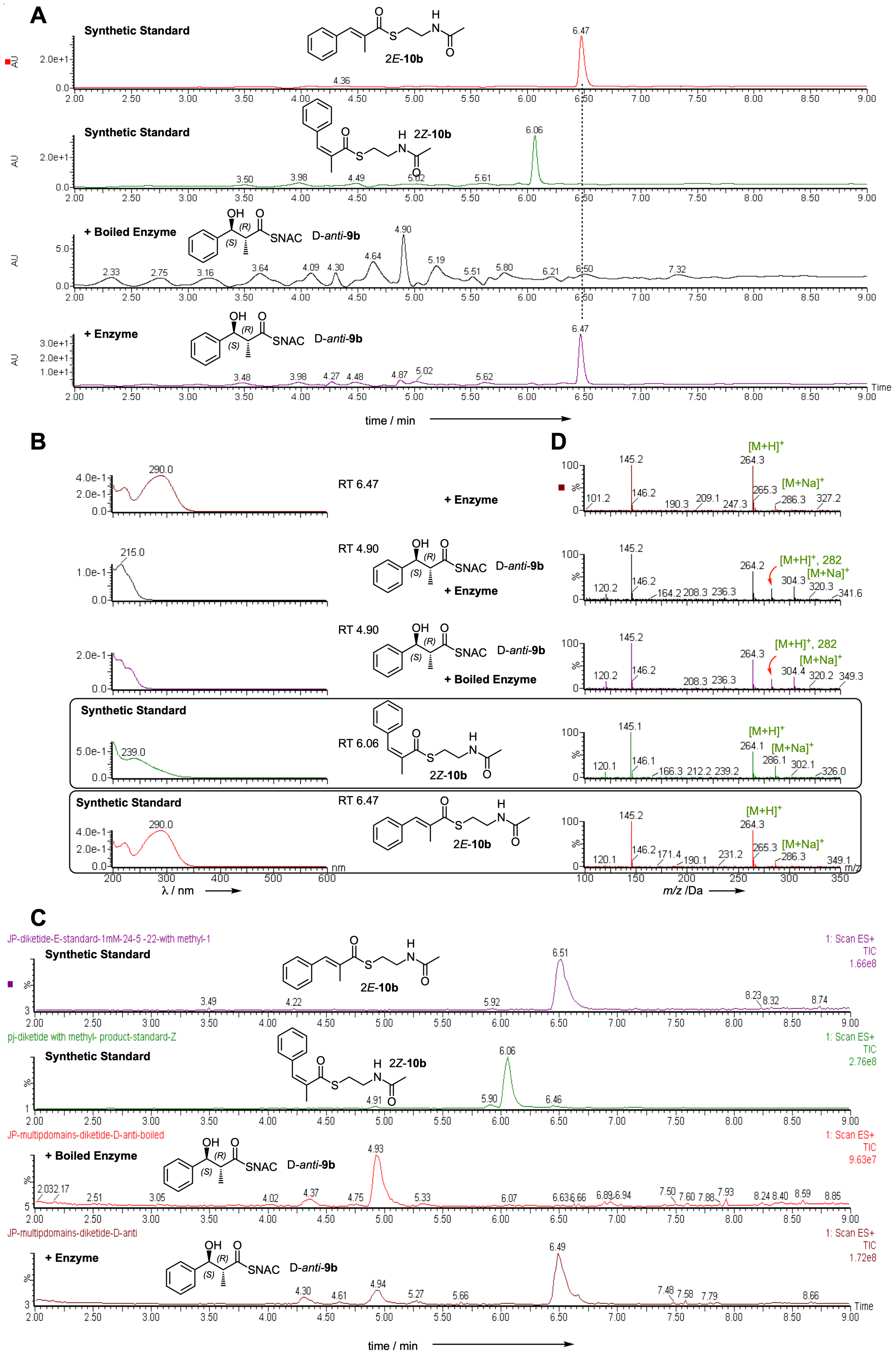

Figure S4.2.4. LCMS data corresponding to Scheme 2. A, DAD data (200-600 nm) for reaction of D-*anti*-**9b** with strPKS multidomain.; **B**, Extracted uv spectra for selected peaks from part A, from top, 6.47 min product peak from enzyme reaction, 4.9 min D-*anti*-**9b** peak from enzyme reaction, 4.9 min D-*anti*-**9b** peak from boiled enzyme assay, 2*Z*-**10b** synthetic standard at 6.06 min, 2*E*-**10b** synthetic standard at 6.47 min; **C**, ES+ TIC analysis of the same reactions scanned between 100 and 1000 Da; **D**, Mass spectra for selected peaks from part C, from top, 6.47 min product peak from enzyme reaction, 4.9 min D-*anti*-**9b** peak from enzyme reaction, 4.9 min D-*anti*-**9b** peak from boiled enzyme assay, 2*Z*-**10b** synthetic standard at 6.06 min, 2*E*-**10b** synthetic standard at 6.47 min.

4.2.5 Data for Schemes 2E + 2F diketides that are not processed

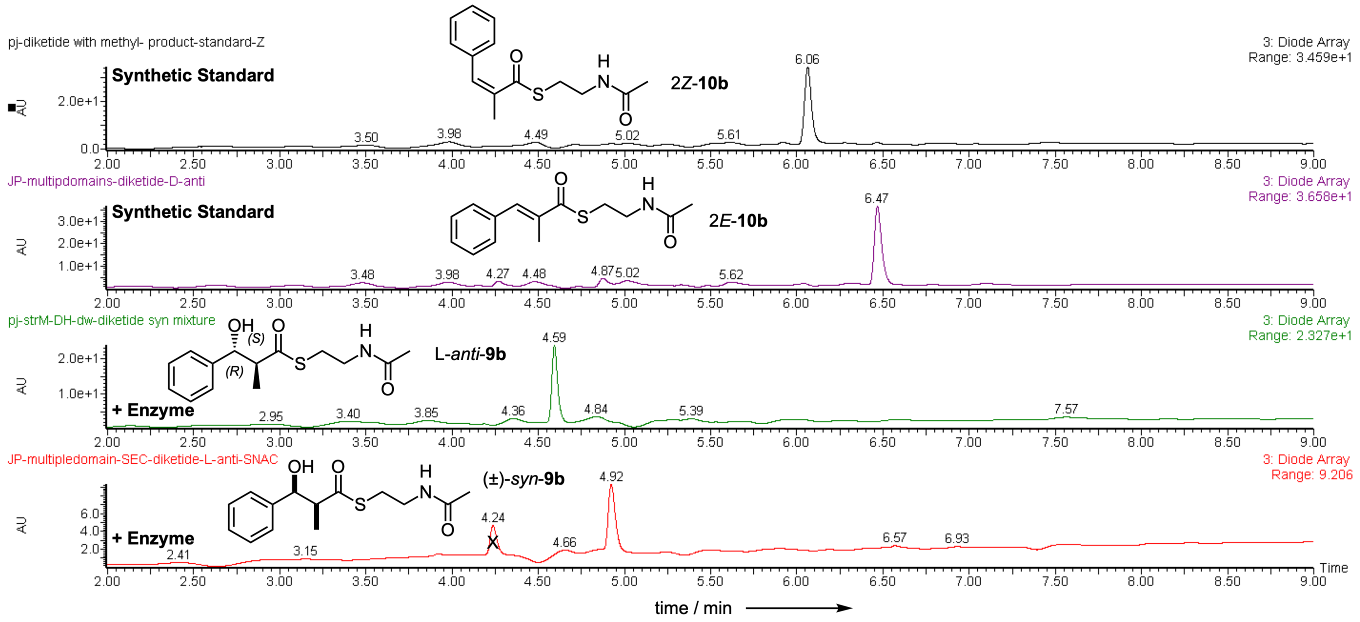

Figure S4.2.5. LCMS data corresponding to Scheme 2E and 2F. From top: synthetic product standards; L-*anti*-**9b** + enzyme; racemic mixture of *syn*-**9b** + enzyme. × = unrelated peak. DAD data 200 - 600 nm.

4.2.6 Data for Scheme 3A - L-*syn*-11b

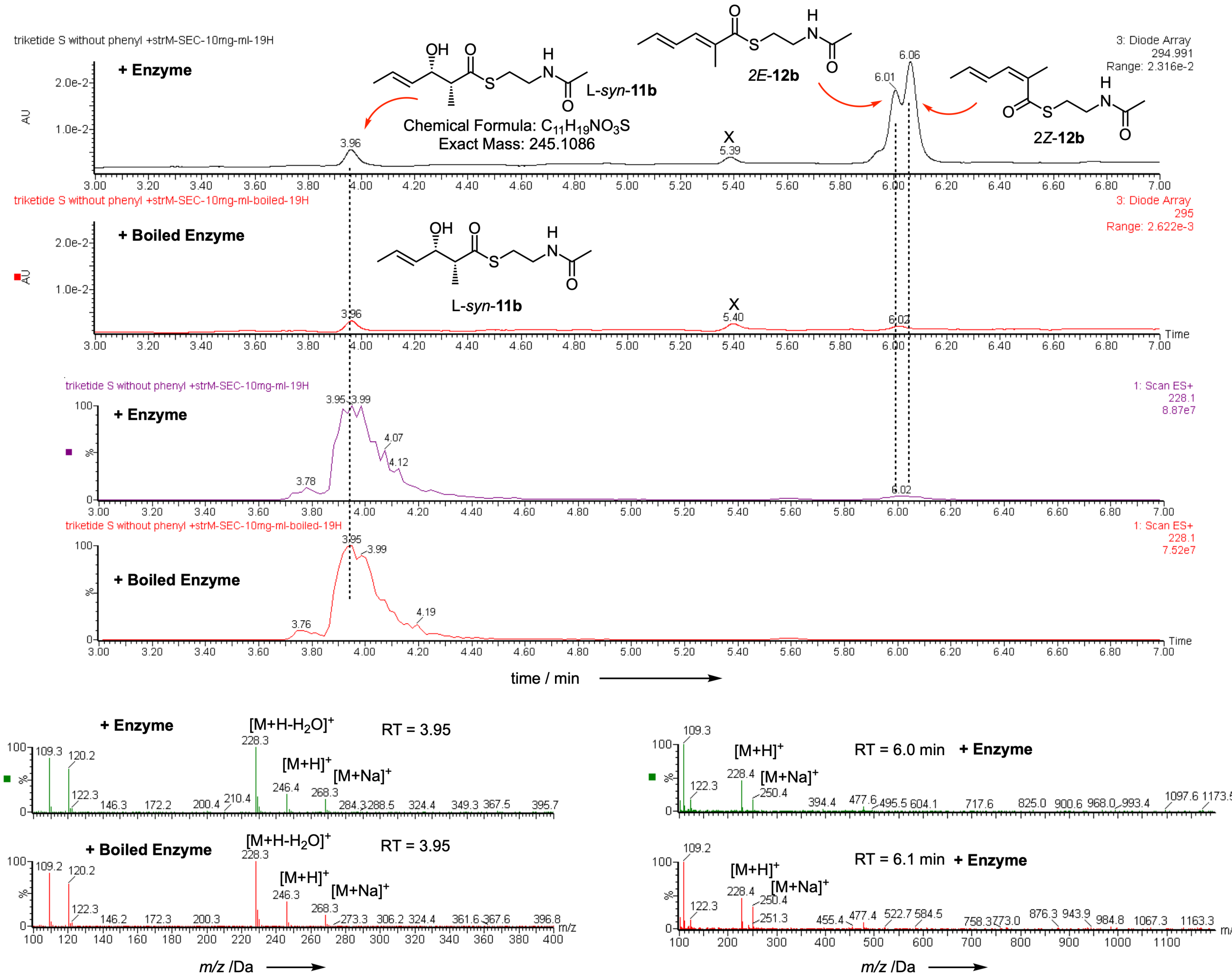

Figure S4.2.6A. LCMS analysis of reaction of L-*syn*-11b catalysed by DH. L-*syn*-**11b** elutes at 3.96 min. In the presence of active enzyme two diene products are observed and are interpreted to be the *E* (6.0 min) and Z (6.1 min) isomers respectively. However resolution could not be improved. × = unrelated peak.

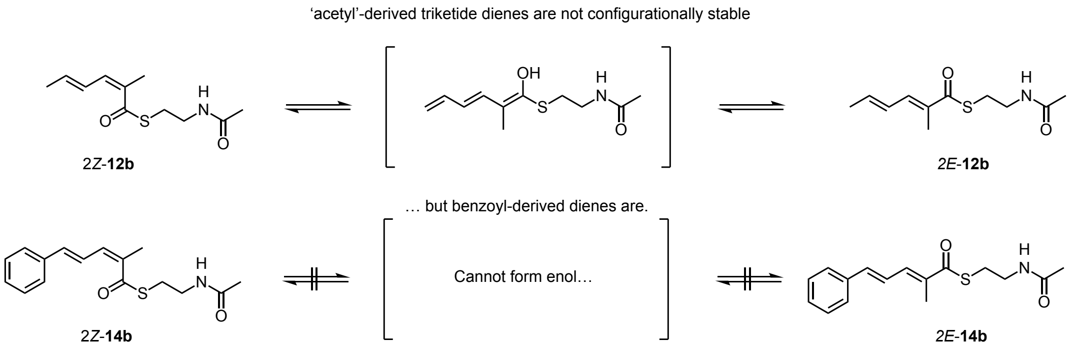

Figure S4.2.6B. Acetyl-derived triketides are not configurationally stable.

4.2.7 Data for Scheme 3B - ± 13a

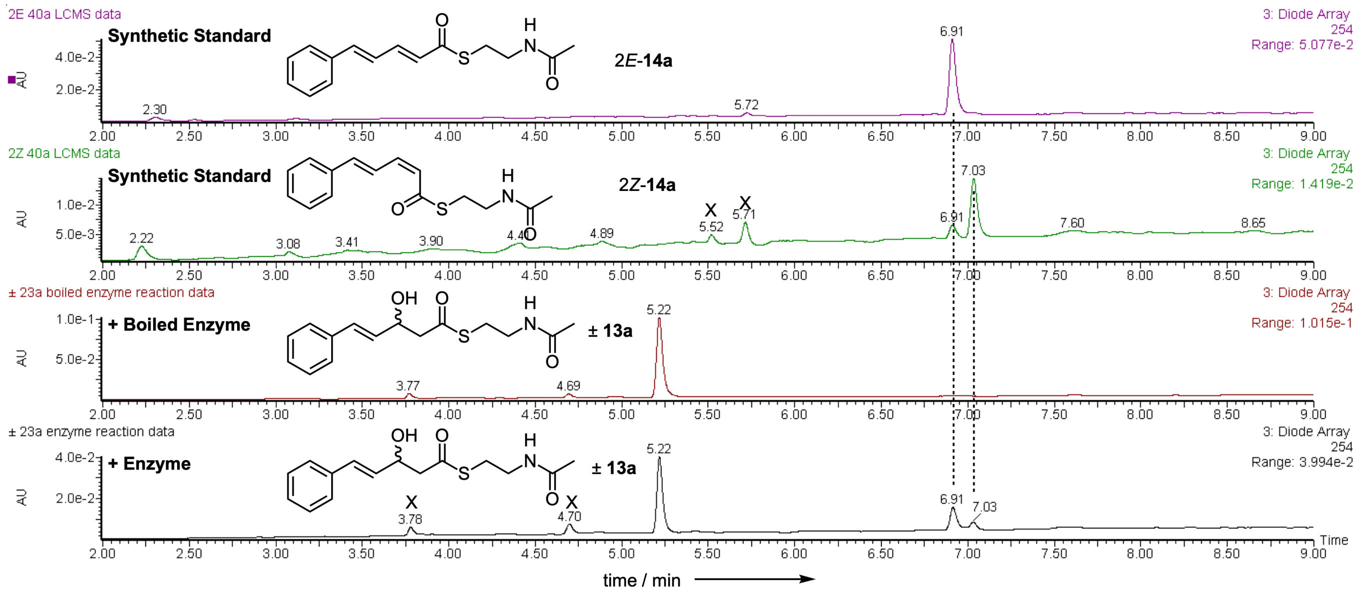

Figure S4.2.7. LCMS data corresponding to Scheme 3B. From top: synthetic product standards; racemic **13a** + boiled enzyme; racemic **13a** + enzyme. × = unrelated peak. DAD data at 254 nm.

4.2.8 Data for Scheme 3C - D-*anti*-13b

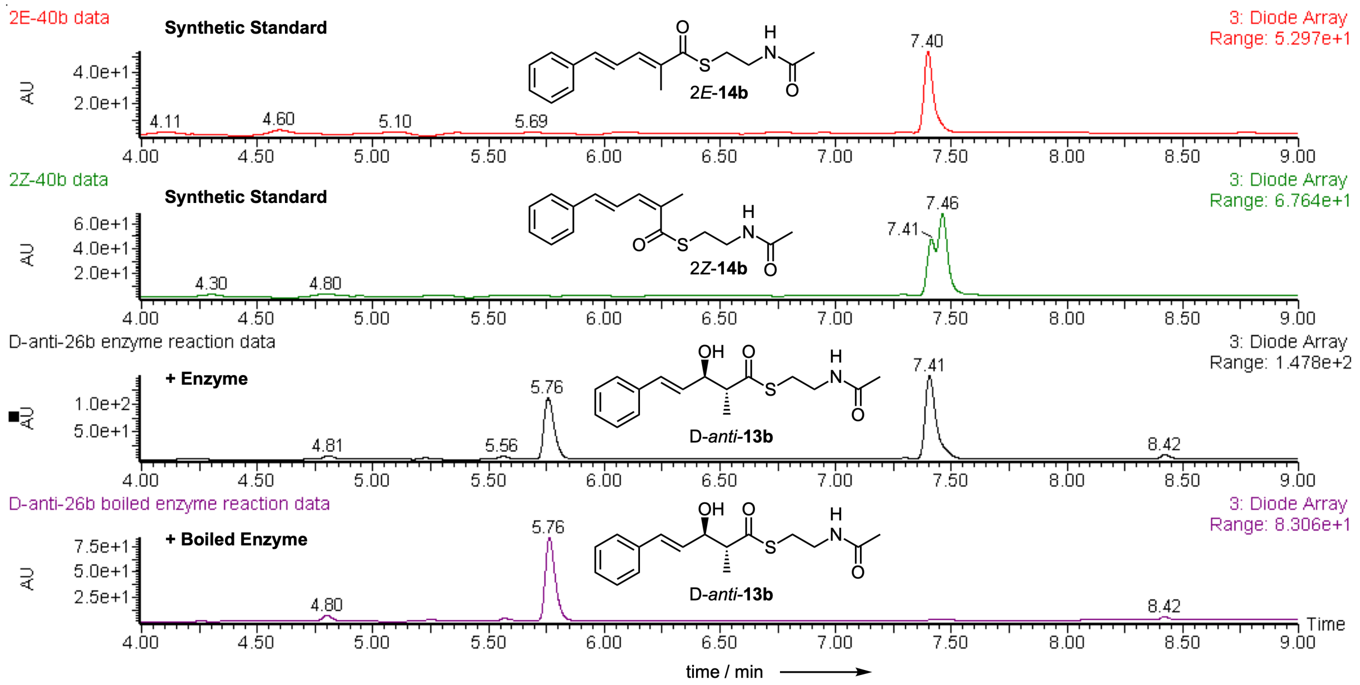

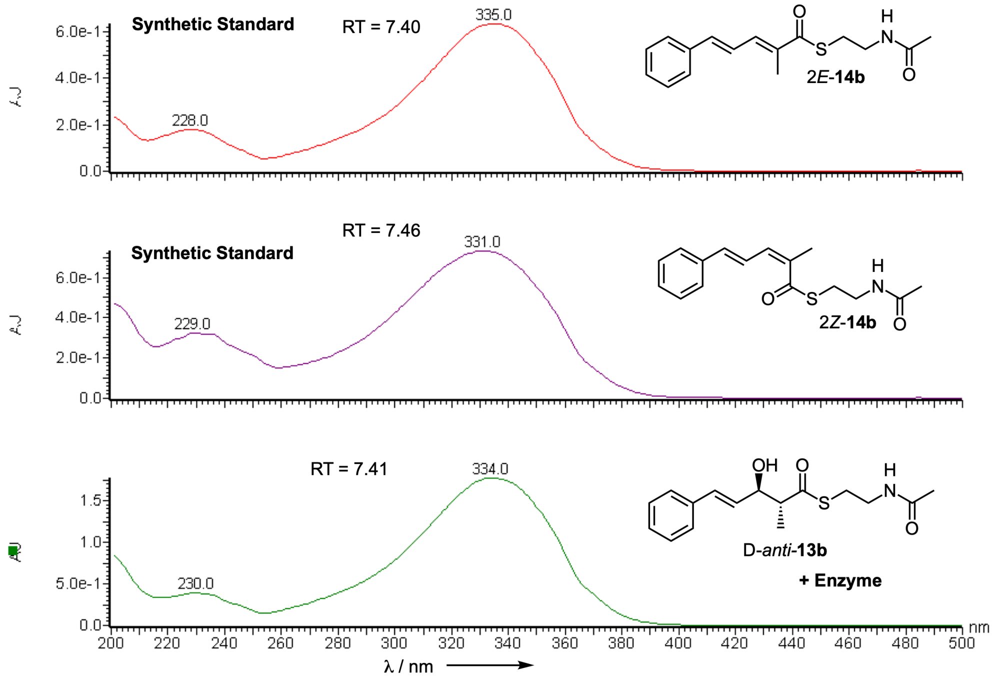

Figure S4.2.8A. LCMS data corresponding to Scheme 3C. From top: synthetic product standards (DAD data 200-600 nm); D-*anti*-**13b** + active enzyme (DAD data 200-600 nm); D-*anti*-**13b** + boiled enzyme (DAD data 200-600 nm); Extracted uv spectrum of 2*E*-**14b** at RT = 7.40 min; Extracted uv spectrum of 2*Z*-**14b** at RT = 7.46 min; Extracted uv spectrum of product peak when D-*anti*-**13b** is reacted with enzyme RT = 7.41 min.

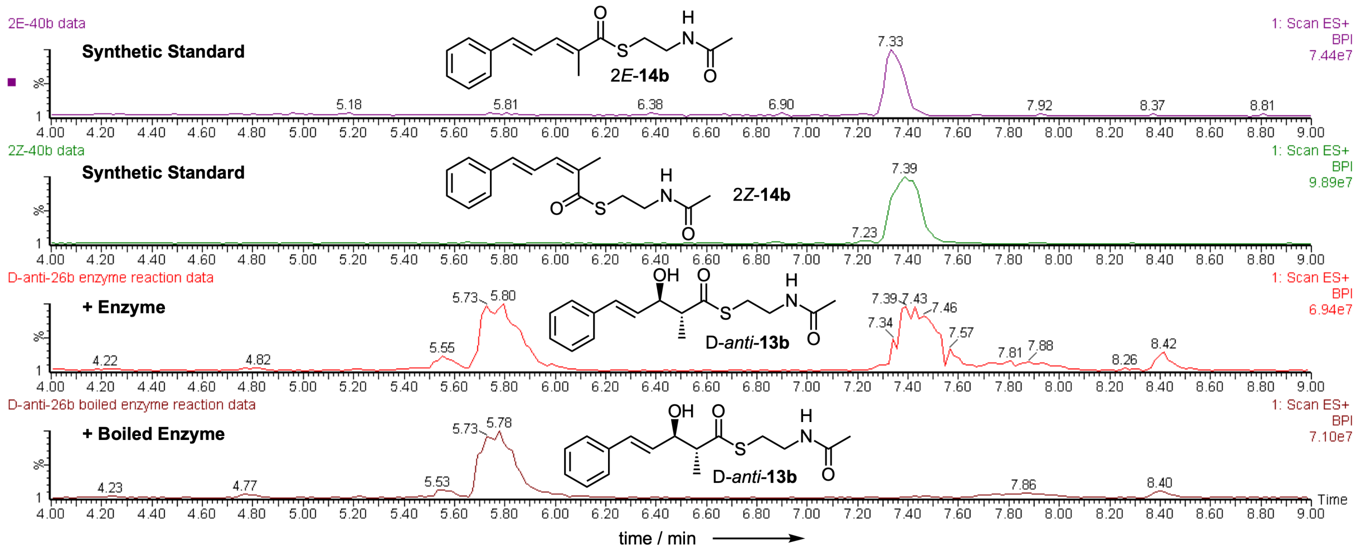

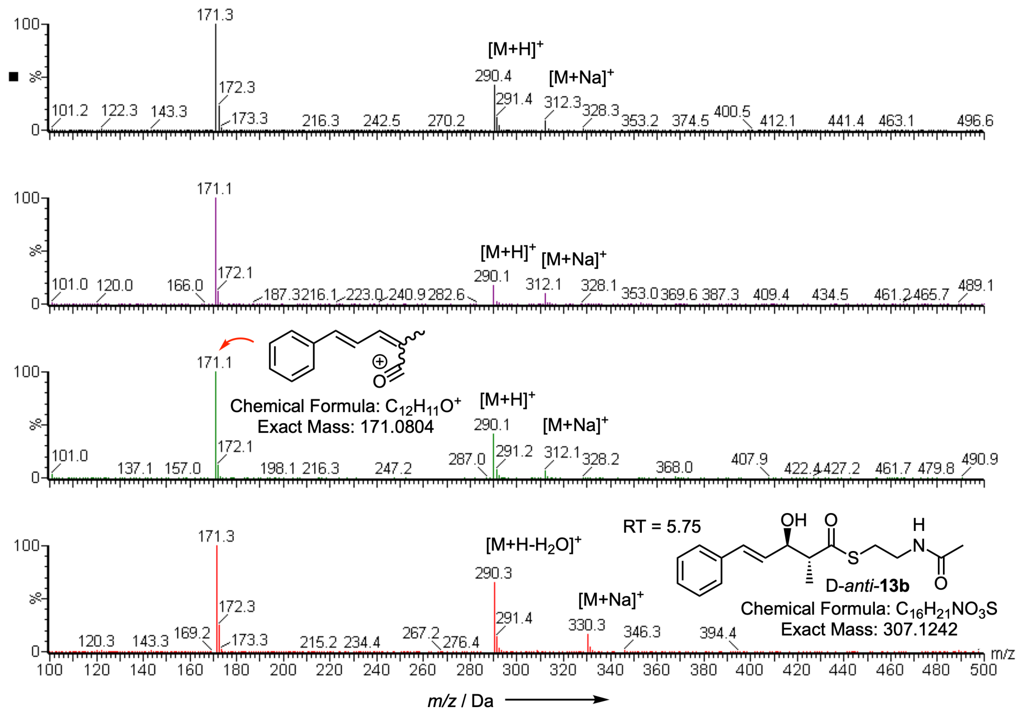

Figure S4.2.8B. LCMS data corresponding to Scheme 3C. From top: synthetic product standards (TIC, ES+, 100 - 1000 Da); D-*anti*-**13b** + active enzyme (TIC, ES+, 100 - 1000 Da); D-*anti*-**13b** + boiled enzyme (TIC, ES+, 100 - 1000 Da); Extracted ES+ spectrum of 2*E*-**14b** at RT = 7.40 min; Extracted ES+ spectrum of 2*Z*-**14b** at RT = 7.46 min; Extracted ES+ spectrum of product peak when D-*anti*-**13b** is reacted with enzyme RT = 7.41 min; Extracted ES+ spectrum of D-*anti*-**13b** RT = 5.75 min.

4.2.9 Data for Scheme 3D - L-*syn*-13b

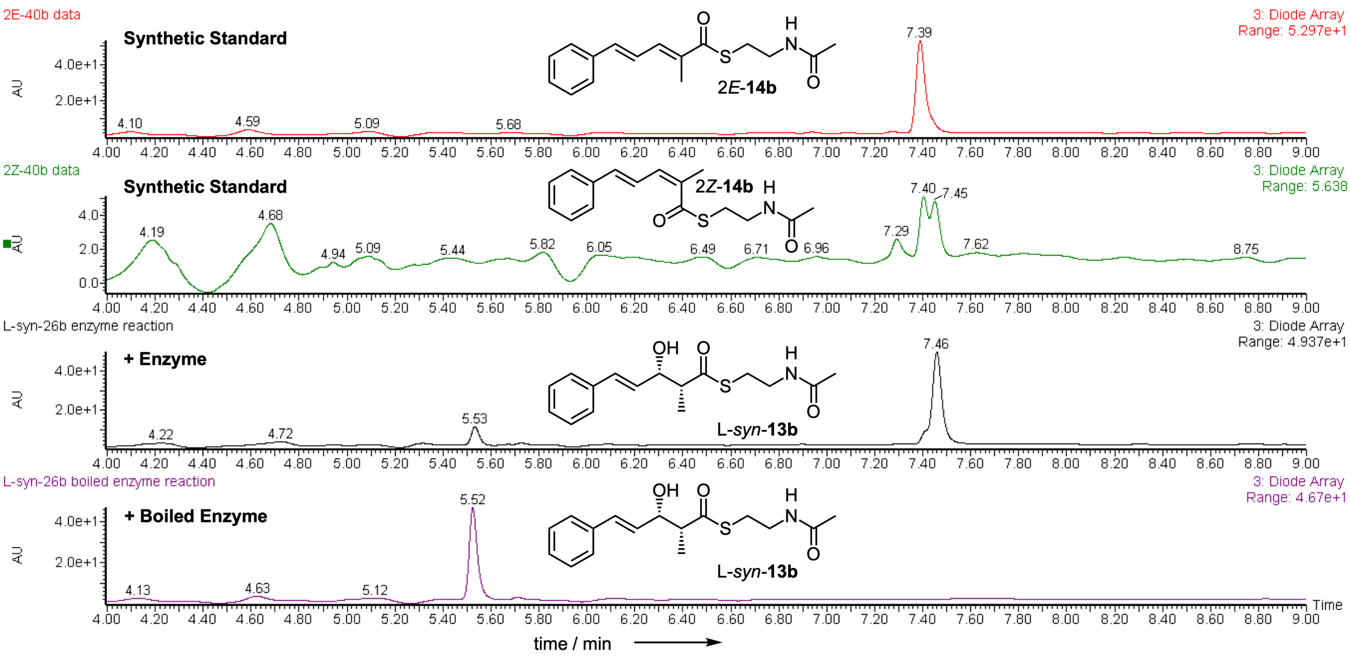

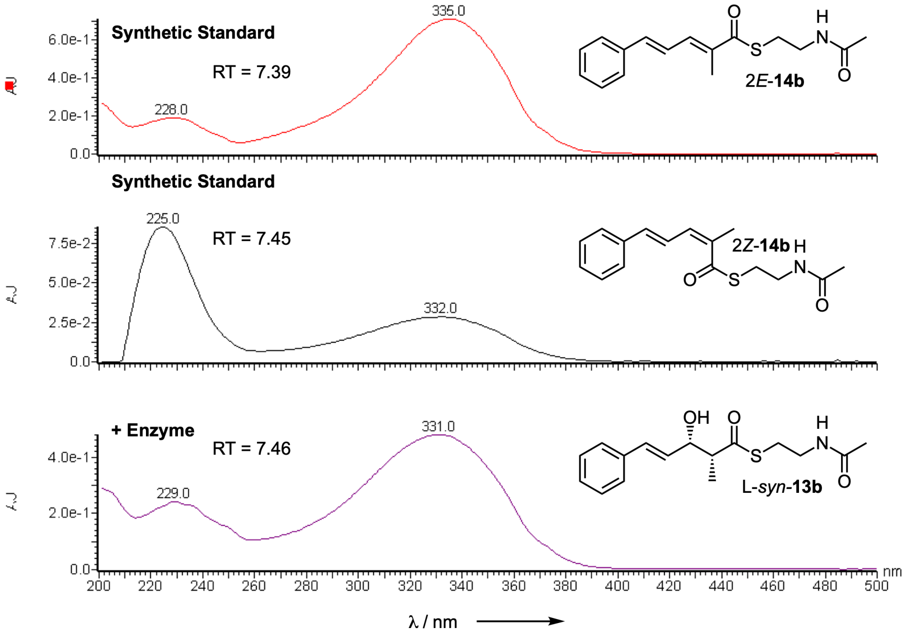

Figure S4.2.9A. LCMS data corresponding to Scheme 3D. From top: synthetic product standards (DAD data 200-600 nm); L-*syn*-**13b** + active enzyme (DAD data 200-600 nm); L-*syn*-**13b** + boiled enzyme (DAD data 200-600 nm); Extracted uv spectrum of 2*E*-**14b** at RT = 7.39 min; Extracted uv spectrum of 2*Z*-**14b** at RT = 7.45 min; Extracted uv spectrum of product peak when L-*syn*-**13b** is reacted with enzyme RT = 7.46 min.

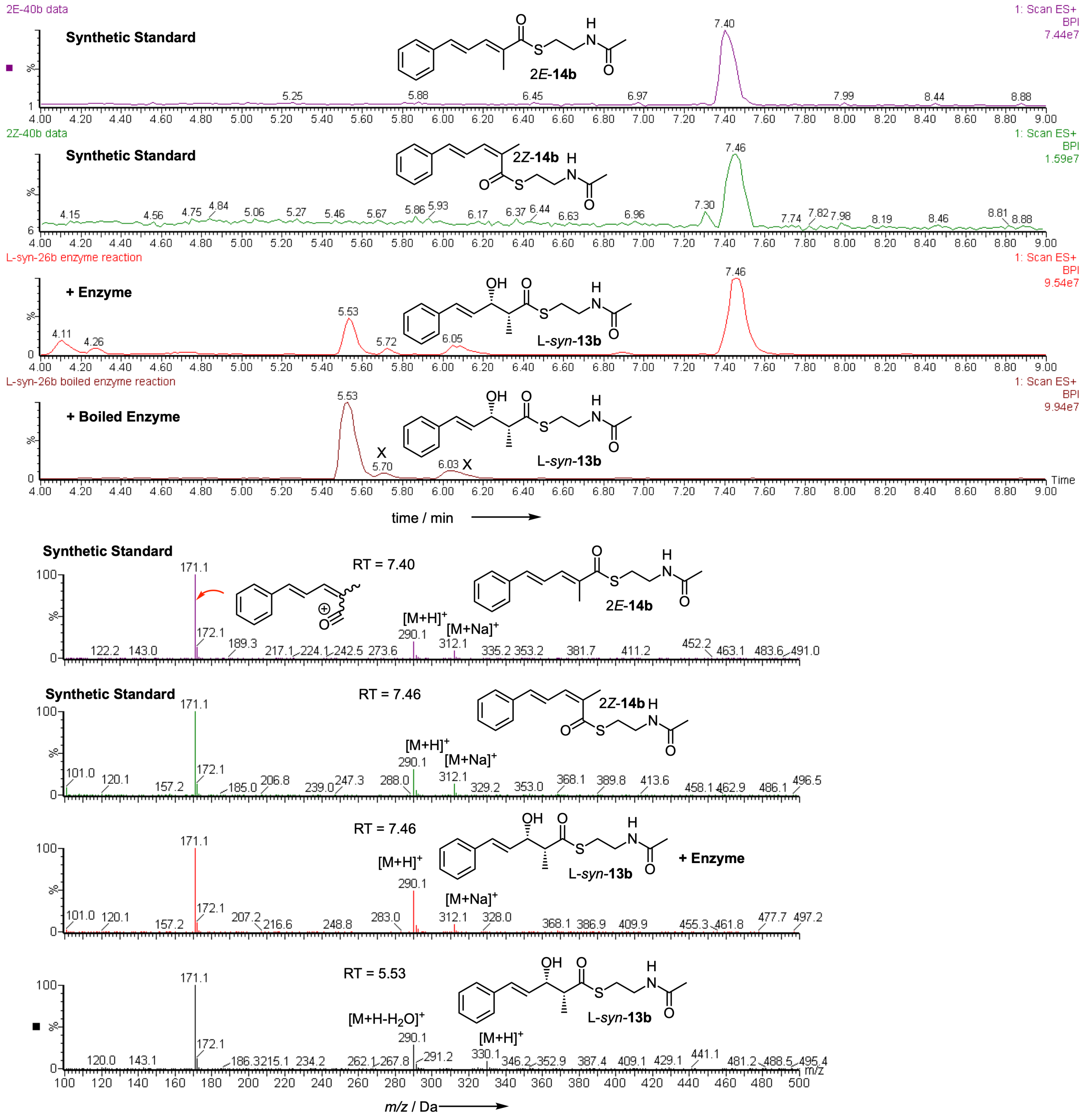

Figure S4.2.9B. LCMS data corresponding to Scheme 3D. From top: synthetic product standards (TIC, ES+, 100 - 1000 Da); L-*syn*-**13b** + active enzyme (TIC, ES+, 100 - 1000 Da); L-*syn*-**13b** + boiled enzyme (TIC, ES+, 100 - 1000 Da); Extracted ES+ spectrum of 2*E*-**14b** at RT = 7.40 min; Extracted ES+ spectrum of 2*Z*-**14b** at RT = 7.46 min; Extracted ES+ spectrum of product peak when L-*syn*-**13b** is reacted with enzyme RT = 7.41 min; Extracted ES+ spectrum of L-*syn*-**13b** RT = 5.53 min.

4.2.10 Data for Scheme 3E - substrates that do not turn over

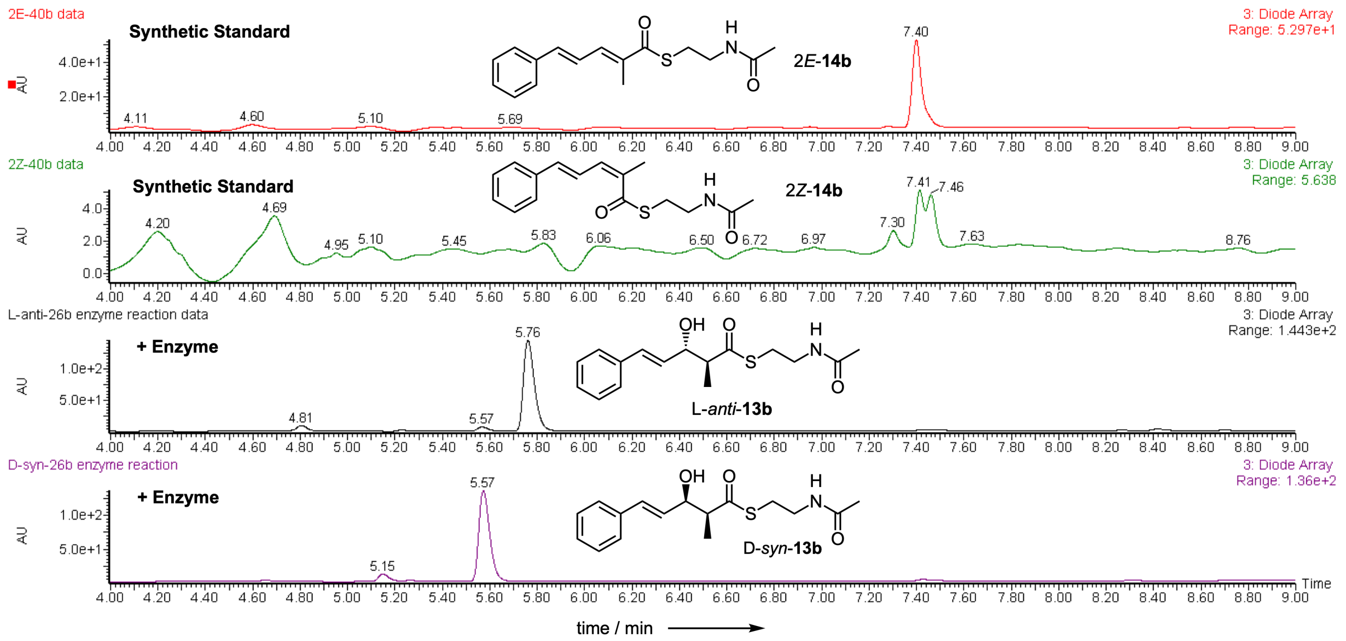

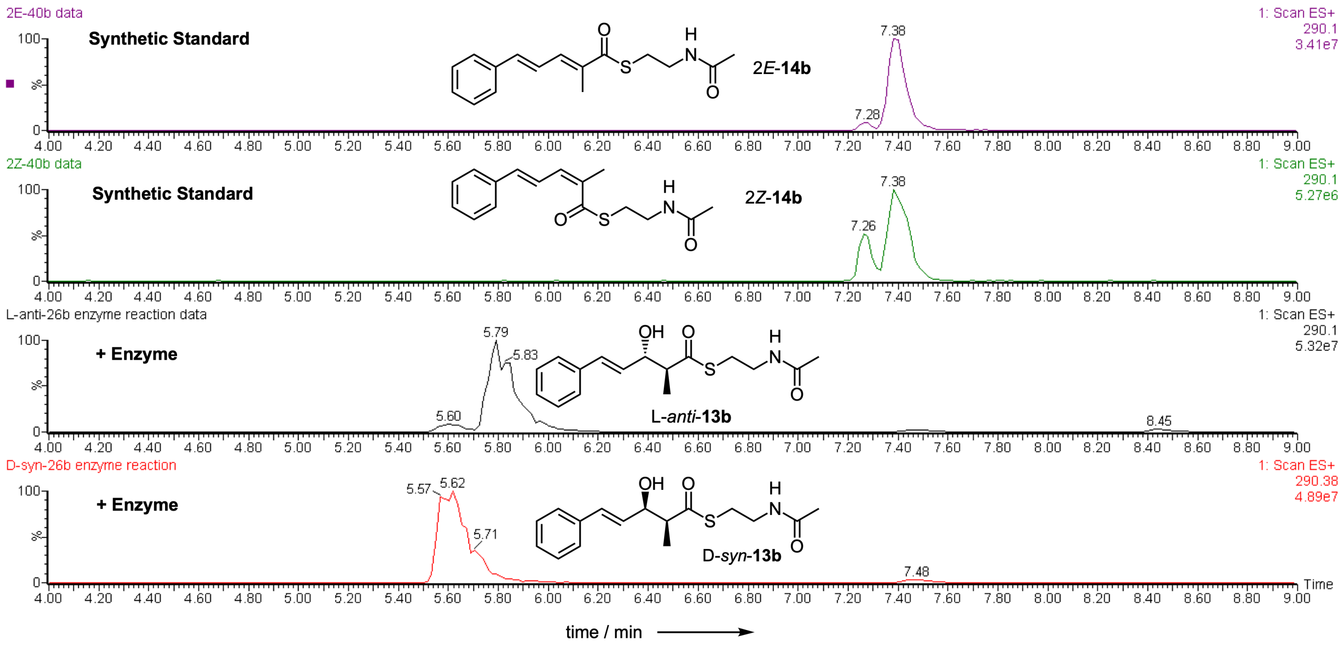

Figure S4.2.10. LCMS data corresponding to Scheme 3E. From top: synthetic product standards (DAD, 200-600nm); L-*anti*-**13b** + active enzyme (DAD, 200-600nm); D-*syn*-**13b** + active enzyme (DAD, 200-600nm); synthetic product standards (EIC, ES+, 290.1 Da); L-*anti*-**13b** + active enzyme (EIC, ES+, 290.1 Da); D-*syn*-**13b** + active enzyme (EIC, ES+, 290.1 Da).

4.3 KR Assays

4.3.1 General Methods

4.3.1 Preparation of 4'-^{2}H,4'-*R*-NADPH and conversion of 15a to E10a and 16a/b to 14a/b

$NADP^+$ disodium trihydrate (4.0 mg, 4.75 µmol) was dissolved in 1.5 mL tris buffer (pH 9.0, 25 mM tris) and 60 µL of isopropanol-d_8 was added. To the reaction mixture $NADP^+$ dependent alcohol dehydrogenase from *Thermoanerobium brockii*[31] (1.0 mg, 3.26 U/mg, TbADH) dissolved in 10 µL tris buffer (pH 8.0, 25 mM tris, 150 mM NaCl) was added. The reaction mixture was incubated at 43 °C and the progress of the reaction was monitored by UV (set to 340 nm) and direct MS. When no further change (~2h) in absorption and no $NADP^+$ ion was observed, the protein was precipitated by the addition of 500 µL CH_3CN. The sample was centrifuged for 20 min, and the supernatant was distributed between two vials. The vials were placed in a SpeedVac vacuum concentrator set to 40 °C and the solvents were removed *in vacuo* to give a yellow oil that was stored at -20 °C and used without any further purification. The crude oil was redissolved in pure H_2O to a desired NADPD concentration and used immediately.

4.3.2 Data for Scheme 6A reactions of 15a

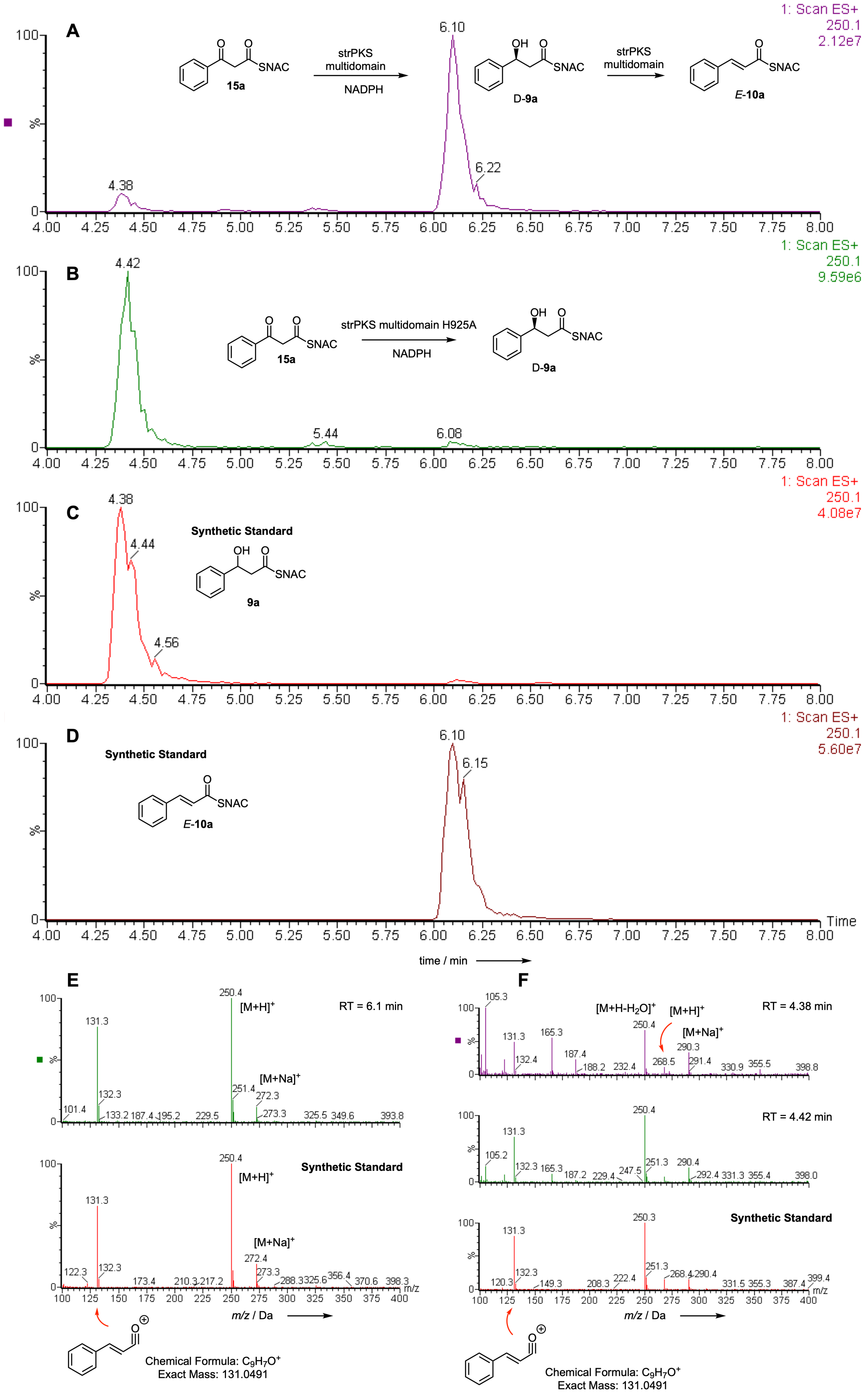

Figure S4.3.2. Reactions of 15a. A, reaction of **15a** with WT multidomain protein + NADPH, (EIC, 250.1 Da, ES+); **B,** reaction of **15a** with H925A multidomain protein + NADPH, (EIC, 250.1 Da, ES+); **C**, Synthetic standard **9a** (EIC, 250.1 Da, ES+); **D**, Synthetic standard *E*-**10a** (EIC, 250.1 Da, ES+); **E**, ESMS^{+} corresponding to *E*-**10a** at indicated retention times; **F**, ESMS^{+} corresponding to **9a** at indicated retention times.

4.3.3 Data for Scheme 6B reactions of 15b

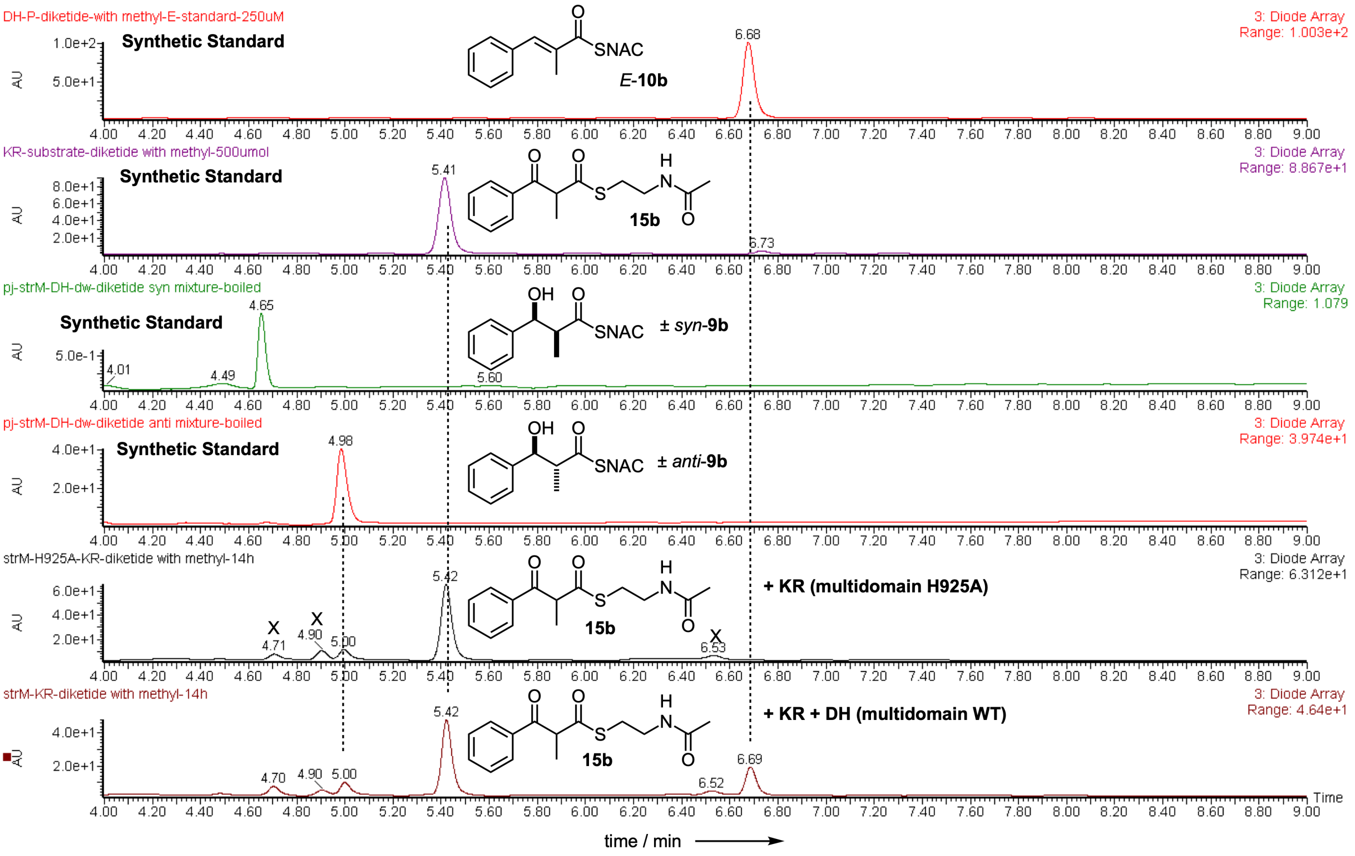

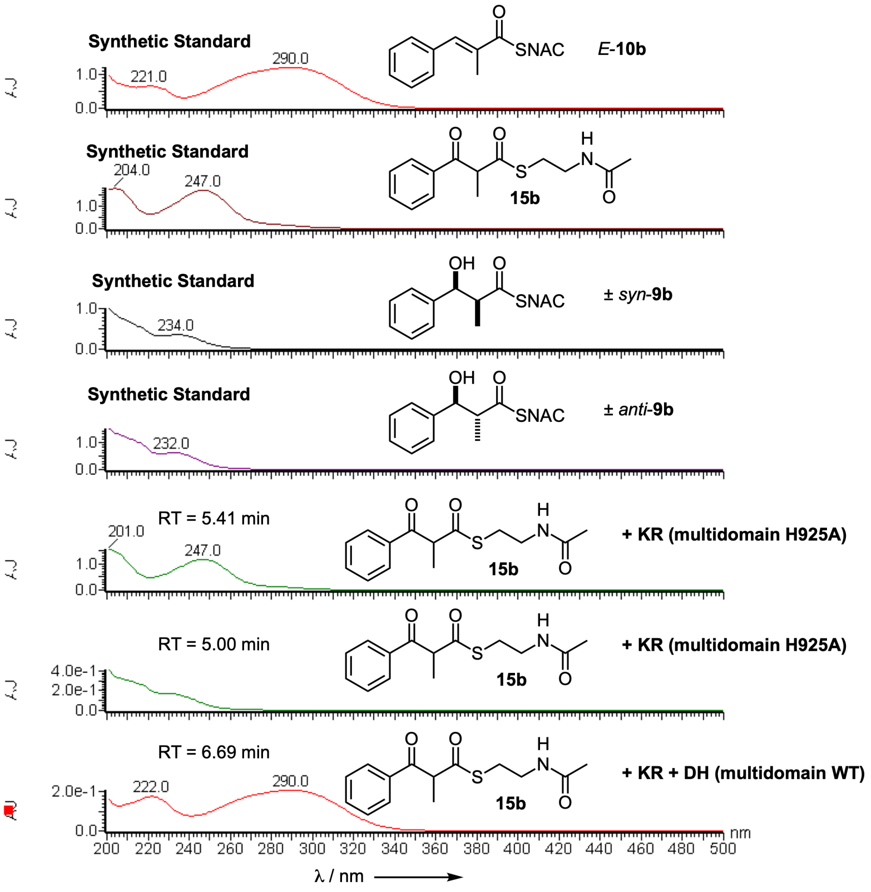

Figure S4.3.3A. LCMS data corresponding to Scheme 6B. From top: synthetic product standards (DAD, 200-600nm); Reaction of **15b** with StrM H925A and NADPH (i.e. KR is active, DH is inactive, DAD, 200-600nm); Reaction of **15b** with StrM WT and NADPH (i.e. KR is active, DH is active, DAD, 200-600nm); uv spectra of indicated synthetic standard compounds; uv spectrum of RT = 5.00 min peak from reaction of **15b** with KR + NADPH; uv spectrum of RT = 6.69 min peak from reaction of **15b** with KR + DH + NADPH.

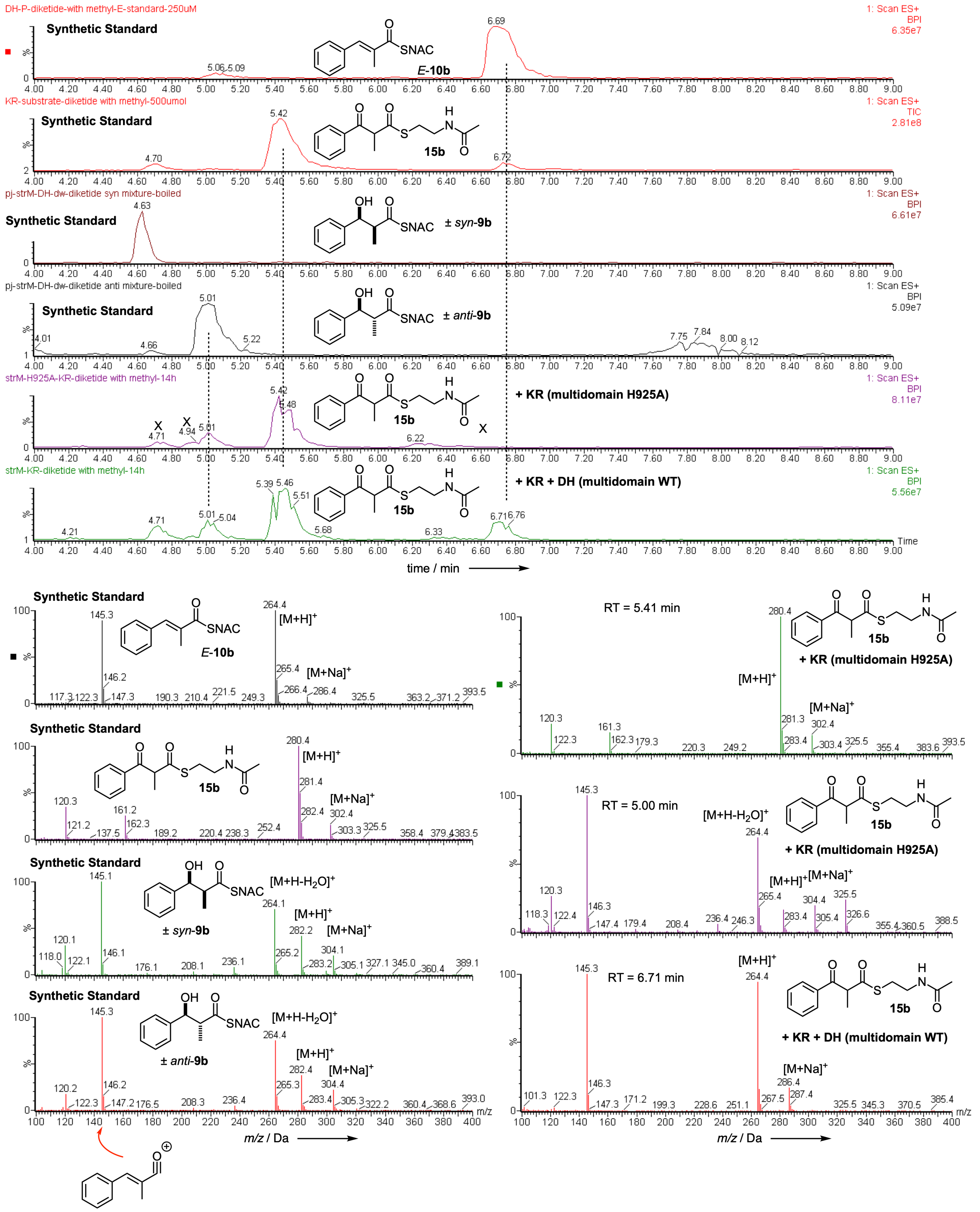

Figure S4.3.3B. LCMS data corresponding to Scheme 6B. From top: synthetic product standards (ES+ chromatograms, 100-1000 Da); Reaction of **15b** with StrM H925A and NADPH (i.e. KR is active, DH is inactive, ES+ chromatograms, 100-1000 Da); Reaction of **15b** with StrM WT and NADPH (i.e. KR is active, DH is active, ES+ chromatograms, 100-1000 Da); ES+ spectra of indicated synthetic standard compounds; ES+ spectrum of RT = 5.41min peak from reaction of **15b** with KR + NADPH; ES+ spectrum of RT = 5.00 min peak from reaction of **15b** with KR + DH + NADPH; ES+ spectrum of RT = 6.71 min peak from reaction of **15b** with KR + DH + NADPH

4.3.4 Data for Scheme 6C - reactions of 16a

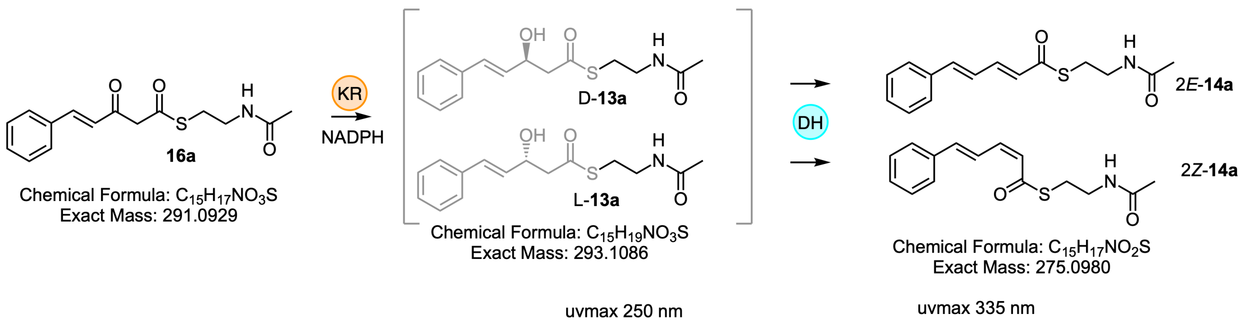

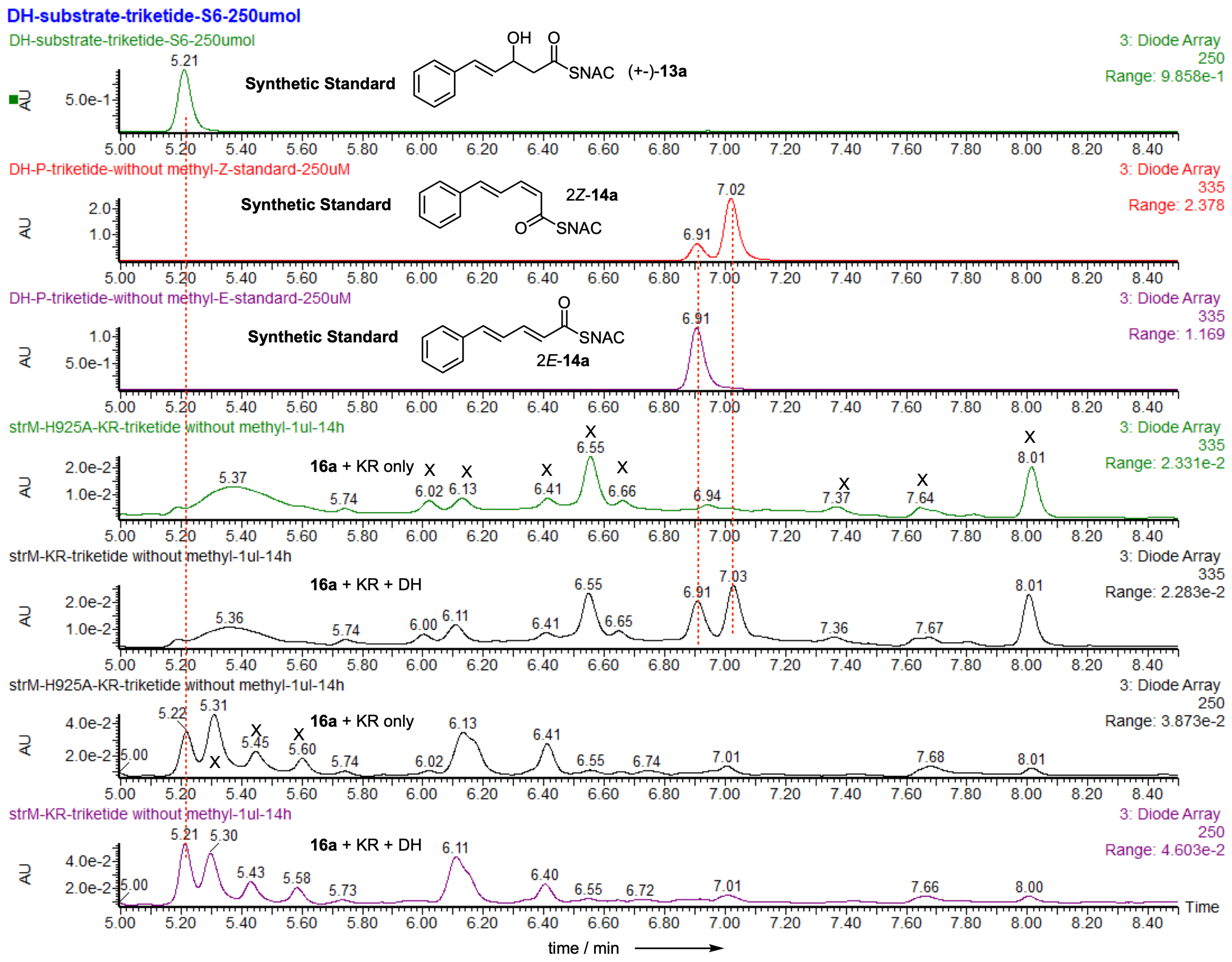

Figure S4.3.4A. LCMS data corresponding to Scheme 6C. From top: synthetic product standards (uv at indicated wavelength); uv chromatogram of reaction of **16a** with StrM H925A and NADPH (*i.e.* KR is active, DH is inactive, 335 nm); uv chromatogram of reaction of **16a** with StrM WT and NADPH (*i.e.* KR is active, DH is active, 335 nm); uv chromatogram of reaction of **16a** with StrM H925A and NADPH (*i.e.* KR is active, DH is inactive, 250 nm); uv chromatogram of reaction of **16a** with StrM WT and NADPH (*i.e.* KR is active, DH is active, 250 nm).

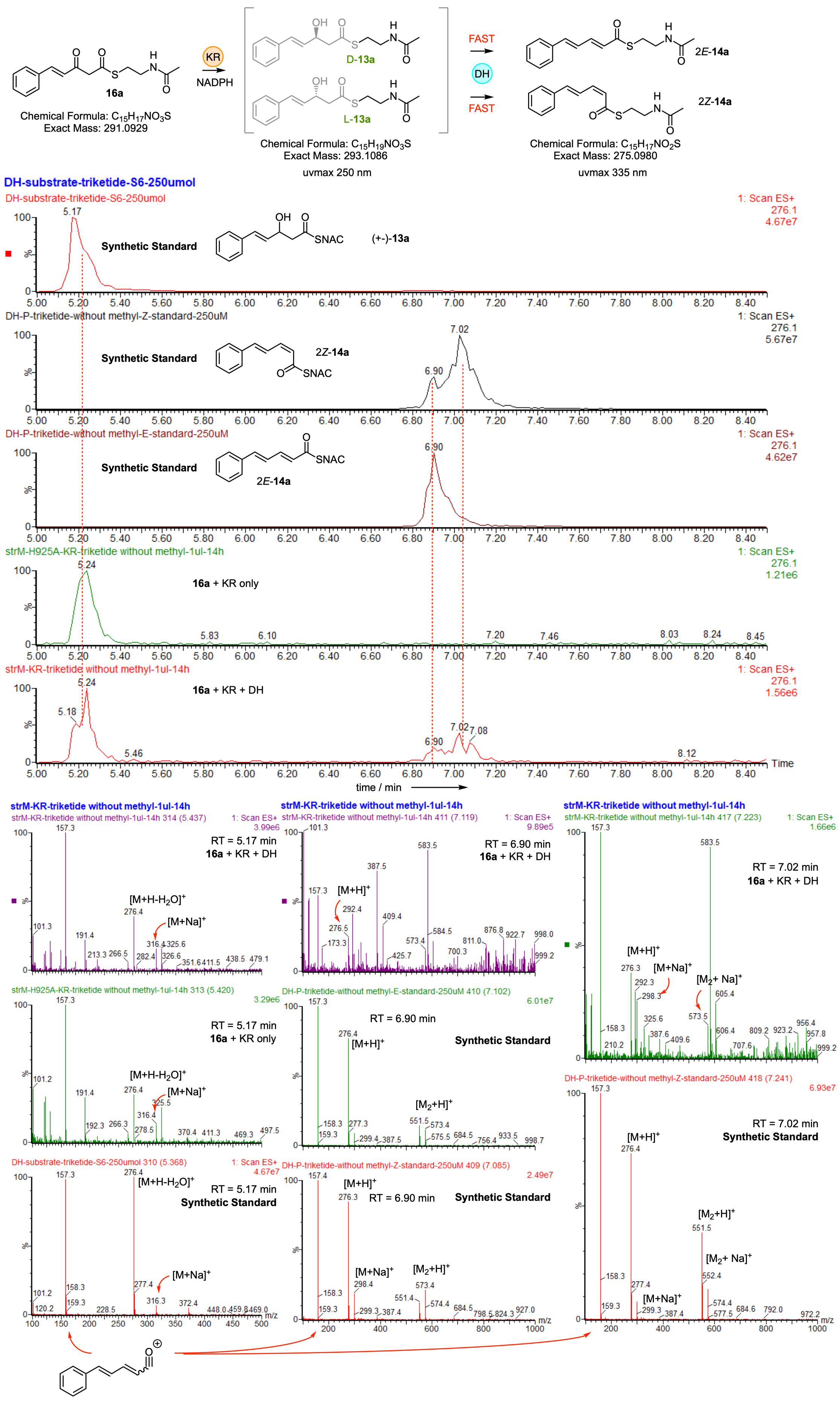

Figure S4.3.4B. LCMS data corresponding to Scheme 6C. From top: synthetic product standards (EIC, ES+ at indicated *m/z*); chromatogram of reaction of **16a** with StrM H925A and NADPH (*i.e.* KR is active, DH is inactive, EIC, ES+ at indicated *m/z*); chromatogram of reaction of **16a** with StrM WT and NADPH (*i.e.* KR is active, DH is active, EIC, ES+ at indicated *m/z*); Extracted mass spectra for indicated experiments at indicated retention times.

4.3.5 Data for Scheme 6D reactions of 16b

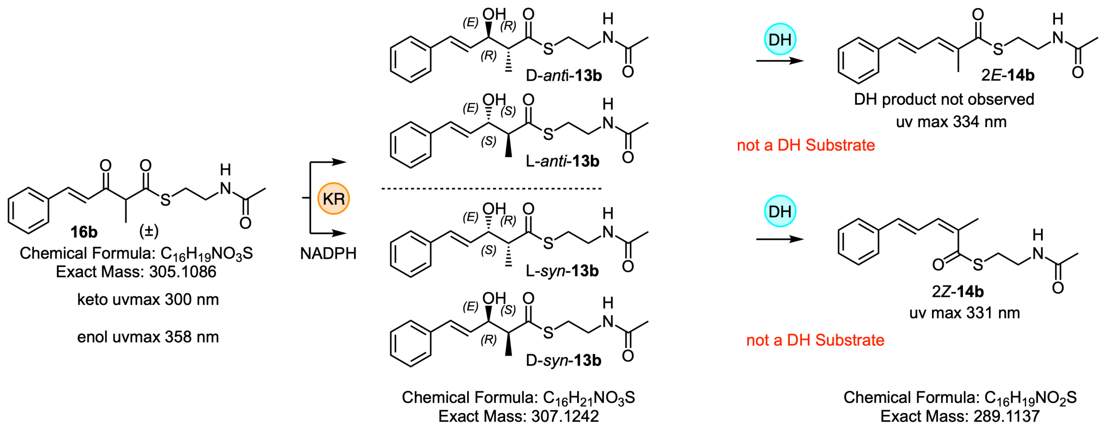

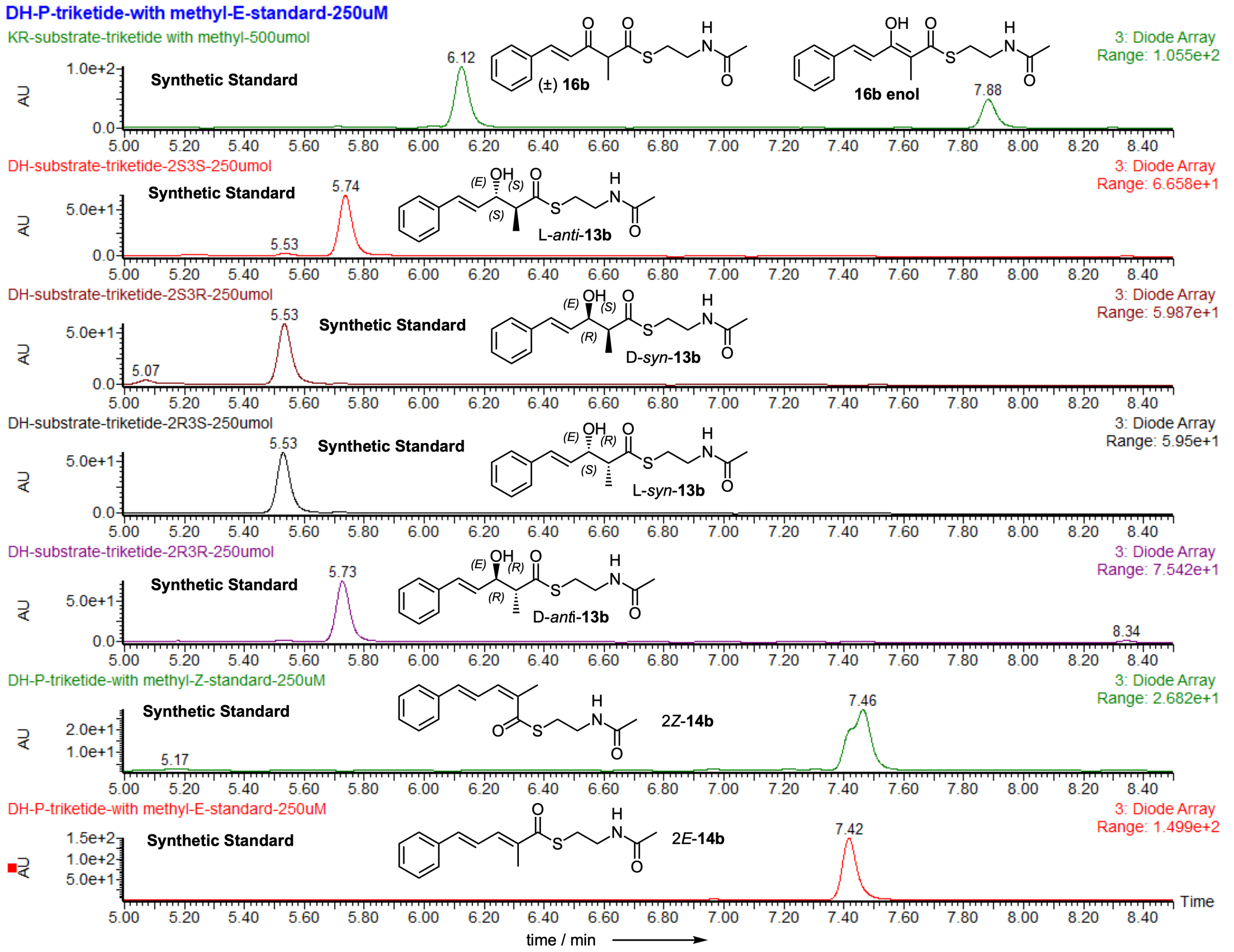

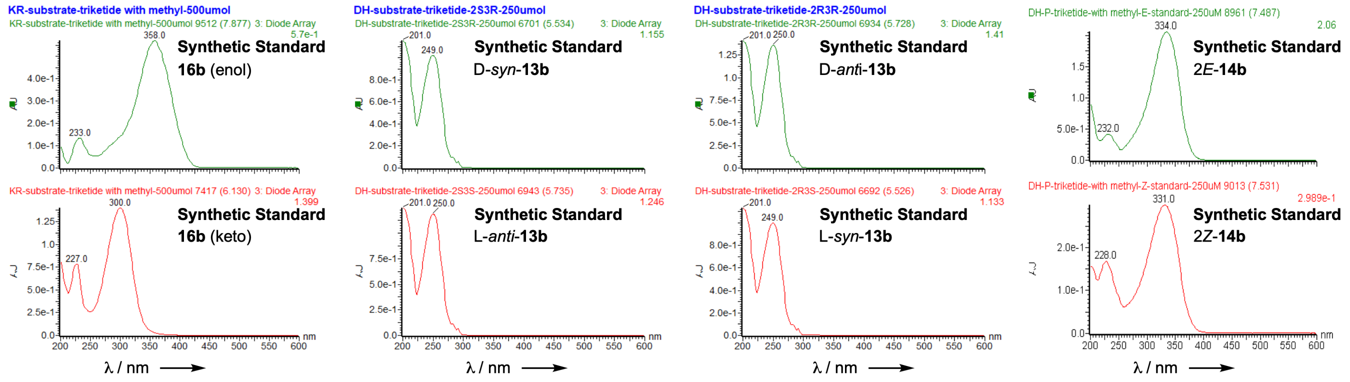

Figure S4.3.5A. LCMS data corresponding to Scheme 6D. From top: synthetic standards (uv, DAD, 200 - 600 nm); uv spectra of indicated peaks.

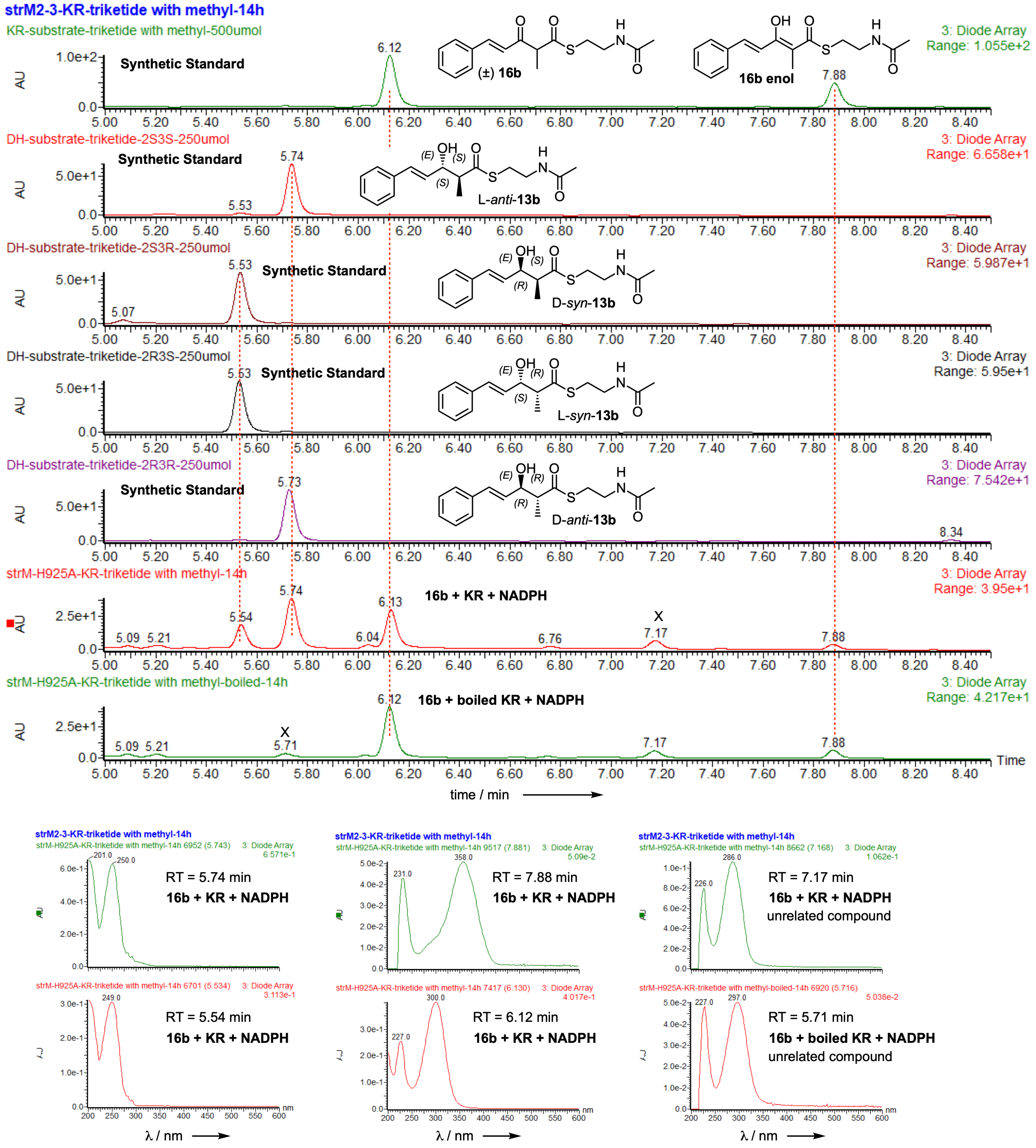

Figure S4.3.5B. LCMS data corresponding to Scheme 6D. From top: synthetic standards (uv, DAD, 200 - 600 nm); uv chromatogram of reaction of **16b** with H925A multidomain protein (only KR active) + NADPH (uv, DAD, 200 - 600 nm); uv chromatogram of reaction of **16b** with boiled H925A multidomain protein + NADPH (uv, DAD, 200 - 600 nm); uv spectra of indicated peaks.

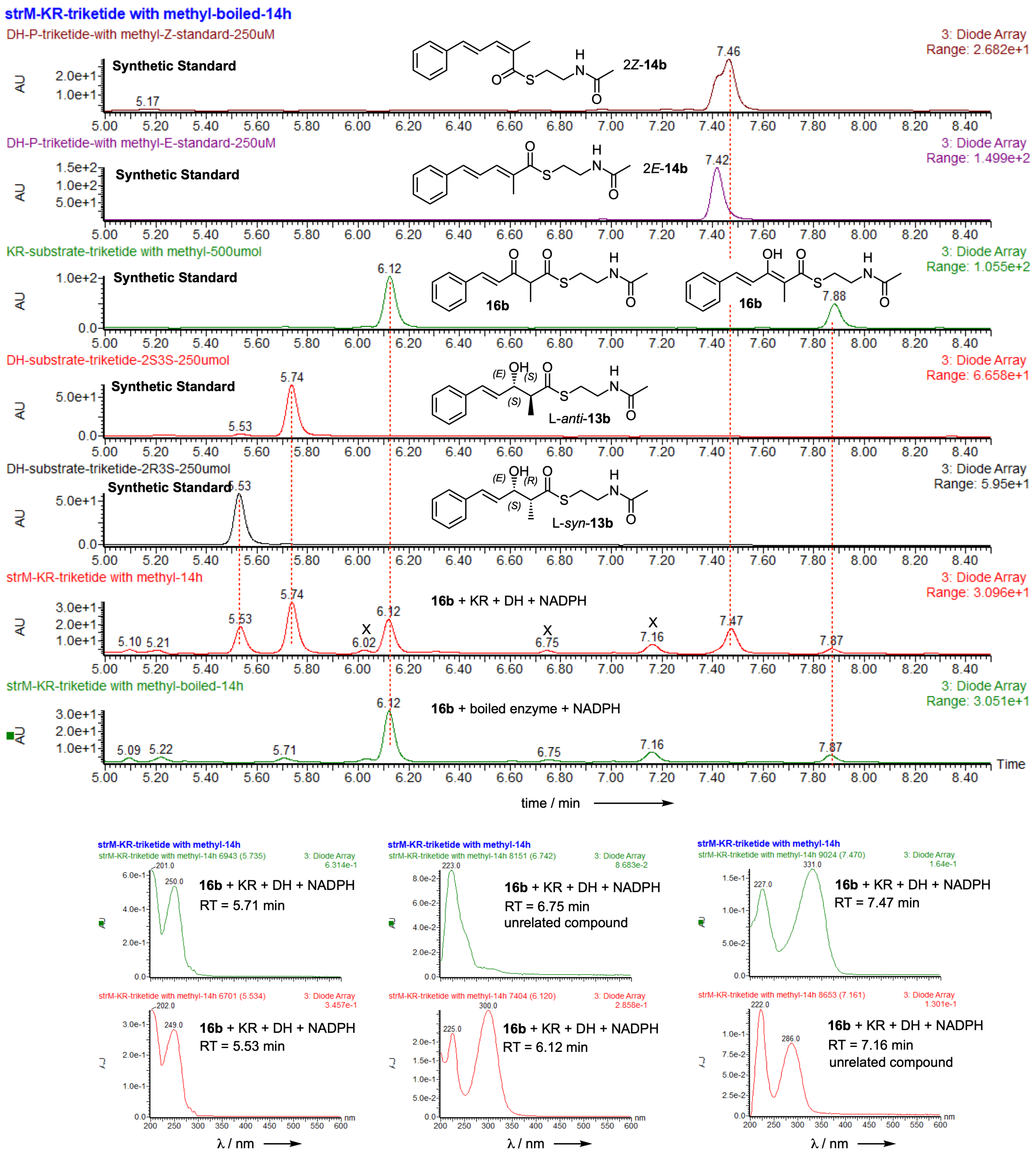

Figure S4.3.5C. LCMS data corresponding to Scheme 6D. From top: synthetic standards (uv, DAD, 200 - 600 nm); uv chromatogram of reaction of **16b** with multidomain protein (both KR and DH active) + NADPH (uv, DAD, 200 - 600 nm); uv chromatogram of reaction of **16b** with boiled multidomain protein + NADPH (uv, DAD, 200 - 600 nm); uv spectra of indicated peaks.

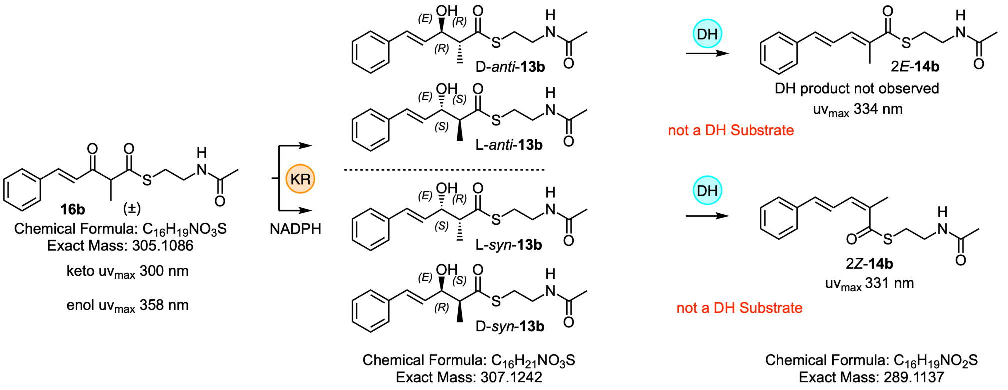

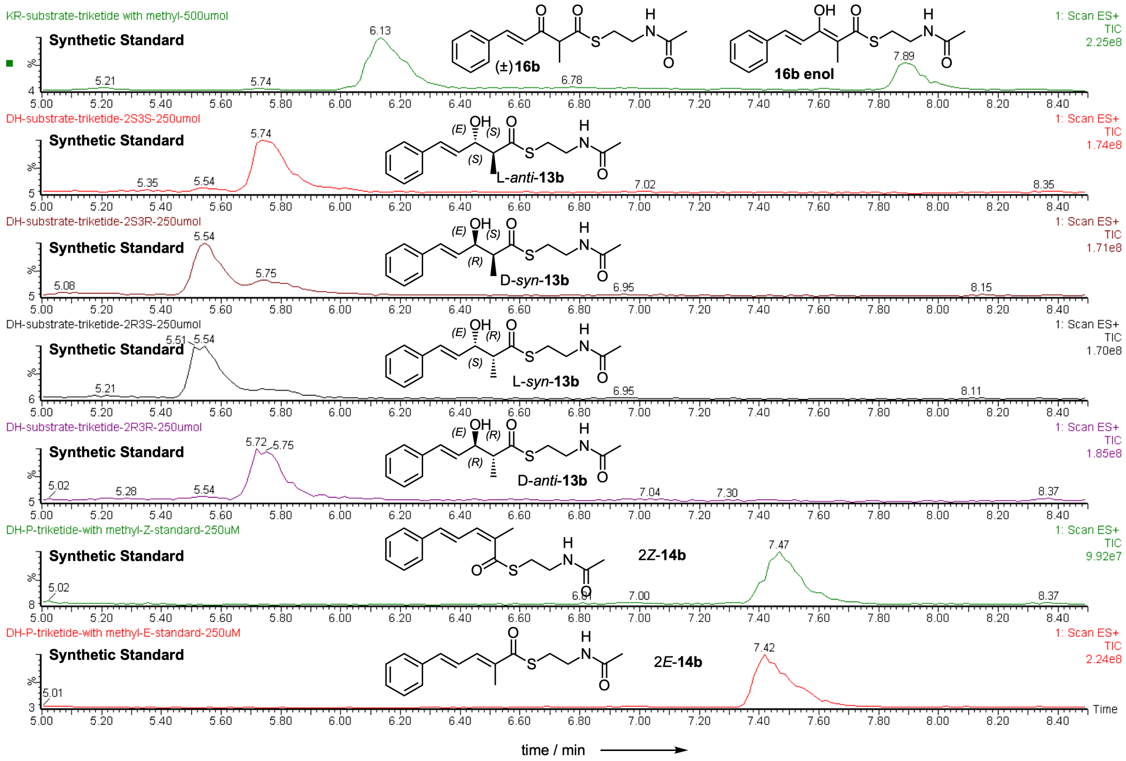

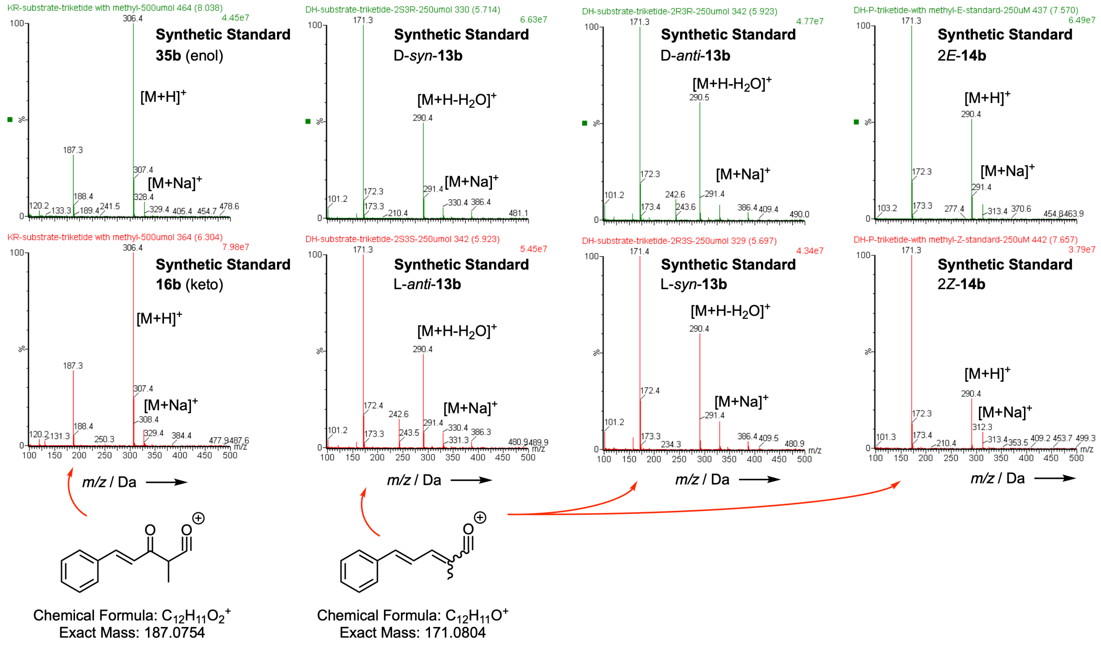

Figure S4.3.5D. LCMS data corresponding to Scheme 6D. From top: synthetic standards (ES+, TIC, 100 - 1000 Da); ES+ spectra of indicated peaks.

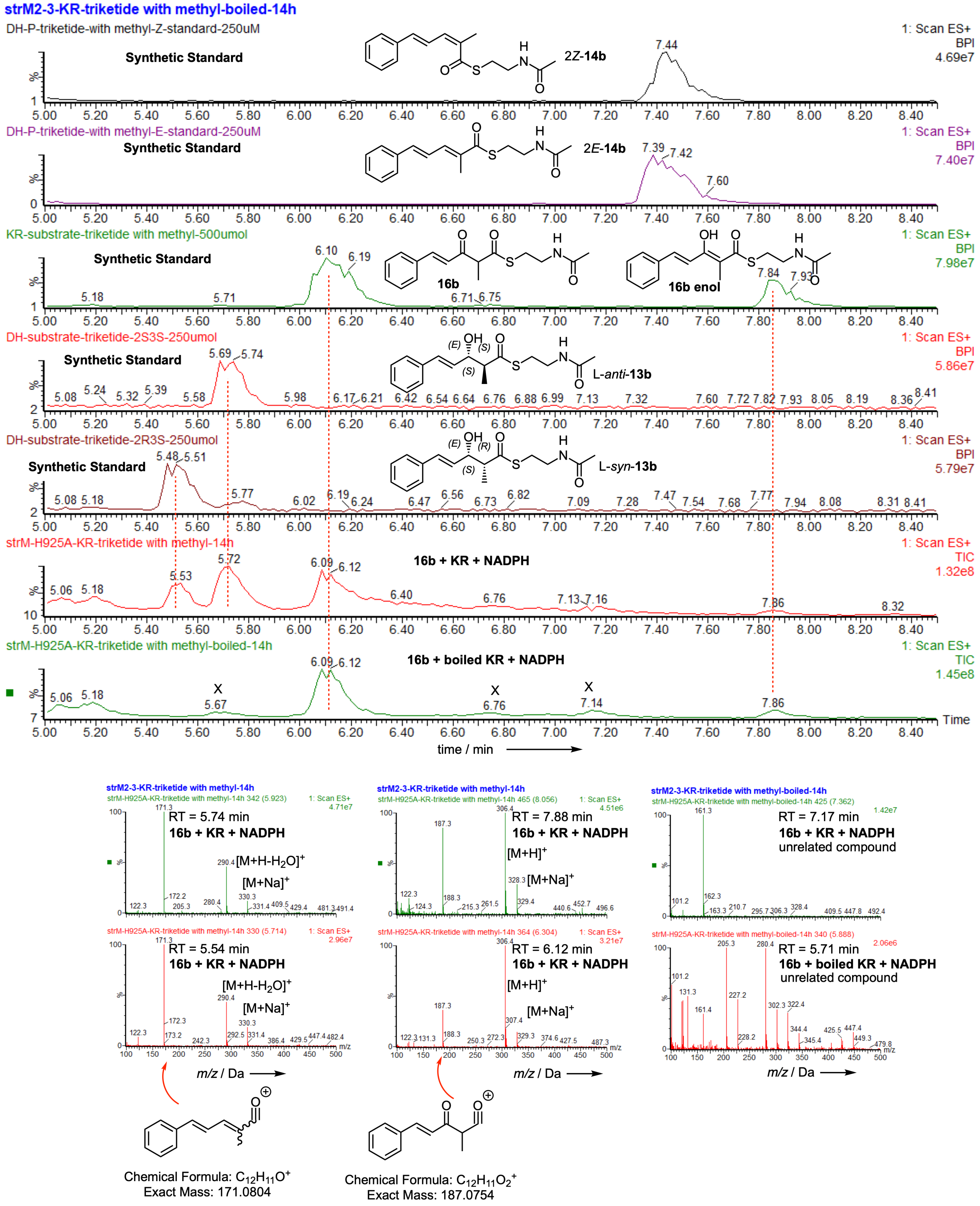

Figure S4.3.5E. LCMS data corresponding to Scheme 6D. From top: synthetic standards (ES+, TIC, 100 - 1000 Da); chromatogram of reaction of **16b** with H925A multidomain protein (only KR active) + NADPH (ES+, TIC, 100 - 1000 Da); chromatogram of reaction of **16b** with boiled H925A multidomain protein + NADPH (ES+, TIC, 100 - 1000 Da); ES+ spectra of indicated peaks.

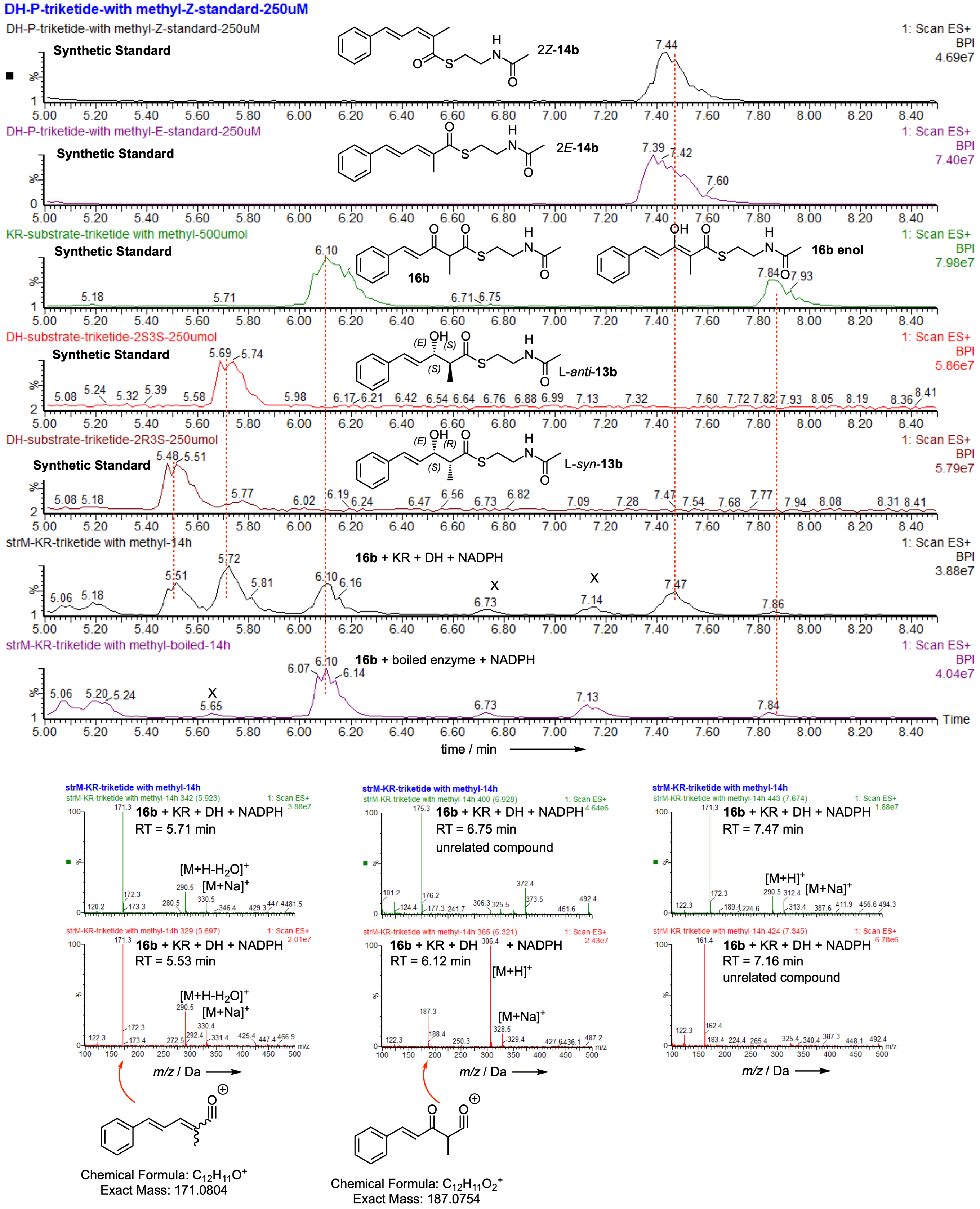

Figure S4.3.5F. LCMS data corresponding to Scheme 6D. From top: synthetic standards (ES+, TIC, 100 - 1000 Da); chromatogram of reaction of **16b** with StrM (both KR and DH active) + NADPH (ES+, TIC, 100 - 1000 Da); uv chromatogram of reaction of **16b** with boiled StrM + NADPH (ES+, TIC, 100 - 1000 Da); ES+ spectra of indicated peaks.

4.4 *C*-MeT and Epimerase Assays

4.4.1 Data for Scheme 7A&B reactions of *C*-MeT1 and *C*-MeT2

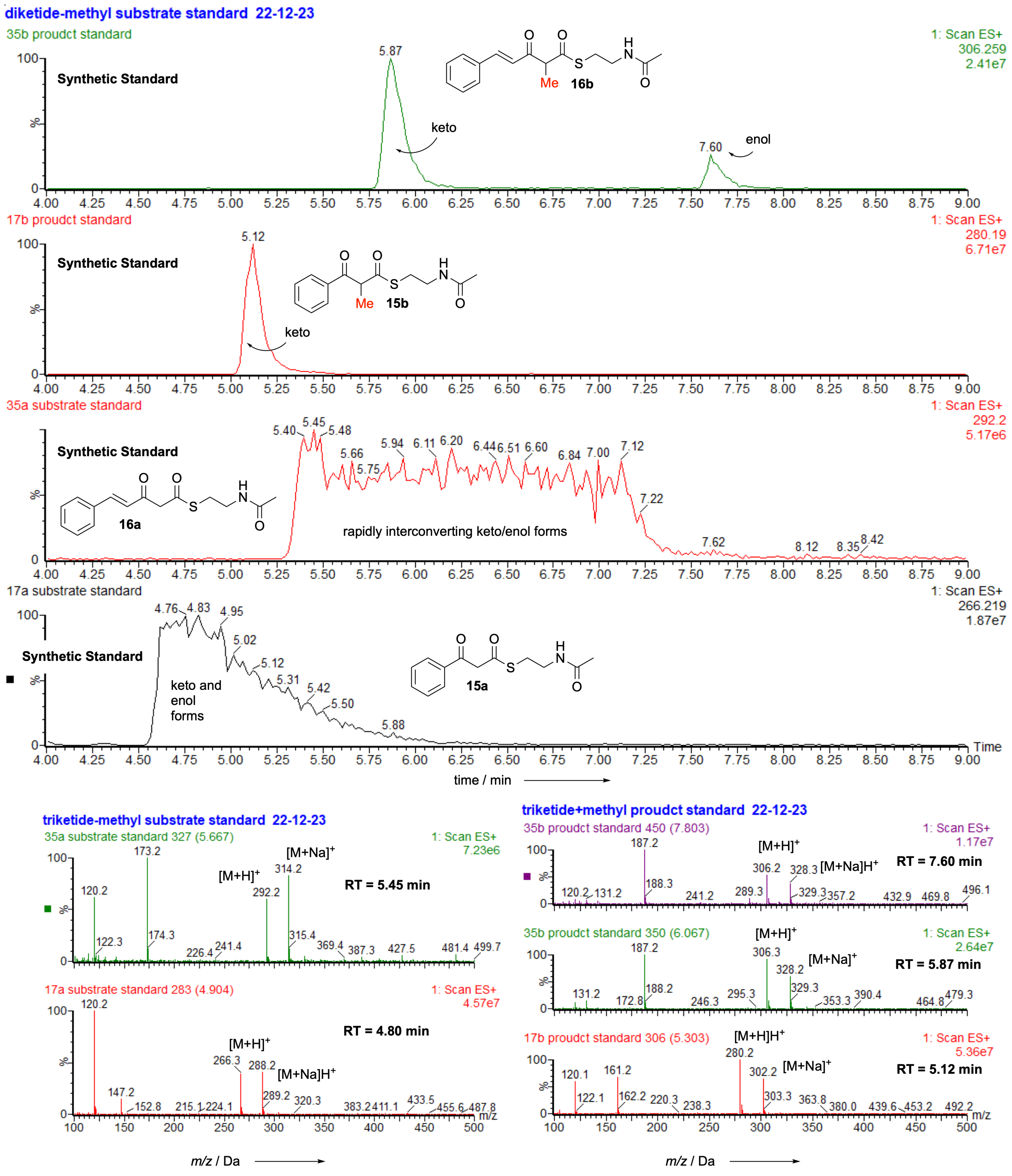

Figure S4.4.1A. LCMS data corresponding to Scheme 7A&B. EIC ES+ chromatograms for Enzyme reactions. Mass corresponds to expected product in each case, (EIC, ES+ at indicated masses corresponding to [M + H]$^+$ for each species); ES$^+$ spectra of indicated product peaks.

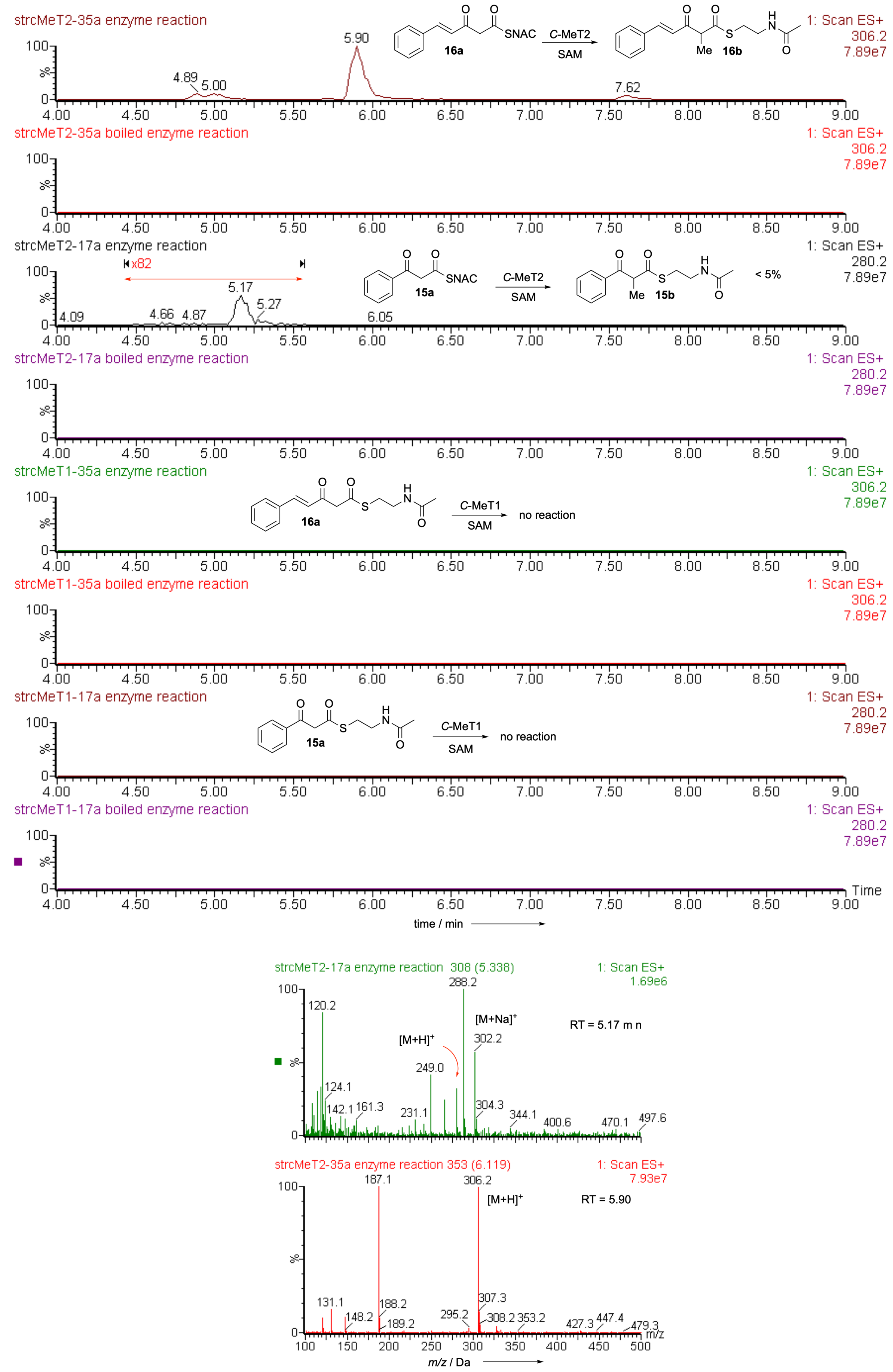

Figure S4.4.1B. LCMS data corresponding to Scheme 7A&B. EIC ES+ chromatograms for Enzyme reactions. Mass corresponds to expected product in each case, (EIC, ES+ at indicated masses corresponding to $[M + H]^+$ for each species); ES^+ spectra of indicated product peaks.

4.4.2 Data for Scheme 7C - reaction of 16a with *C*-MeT2 + KR/DH + SAM + NADPH

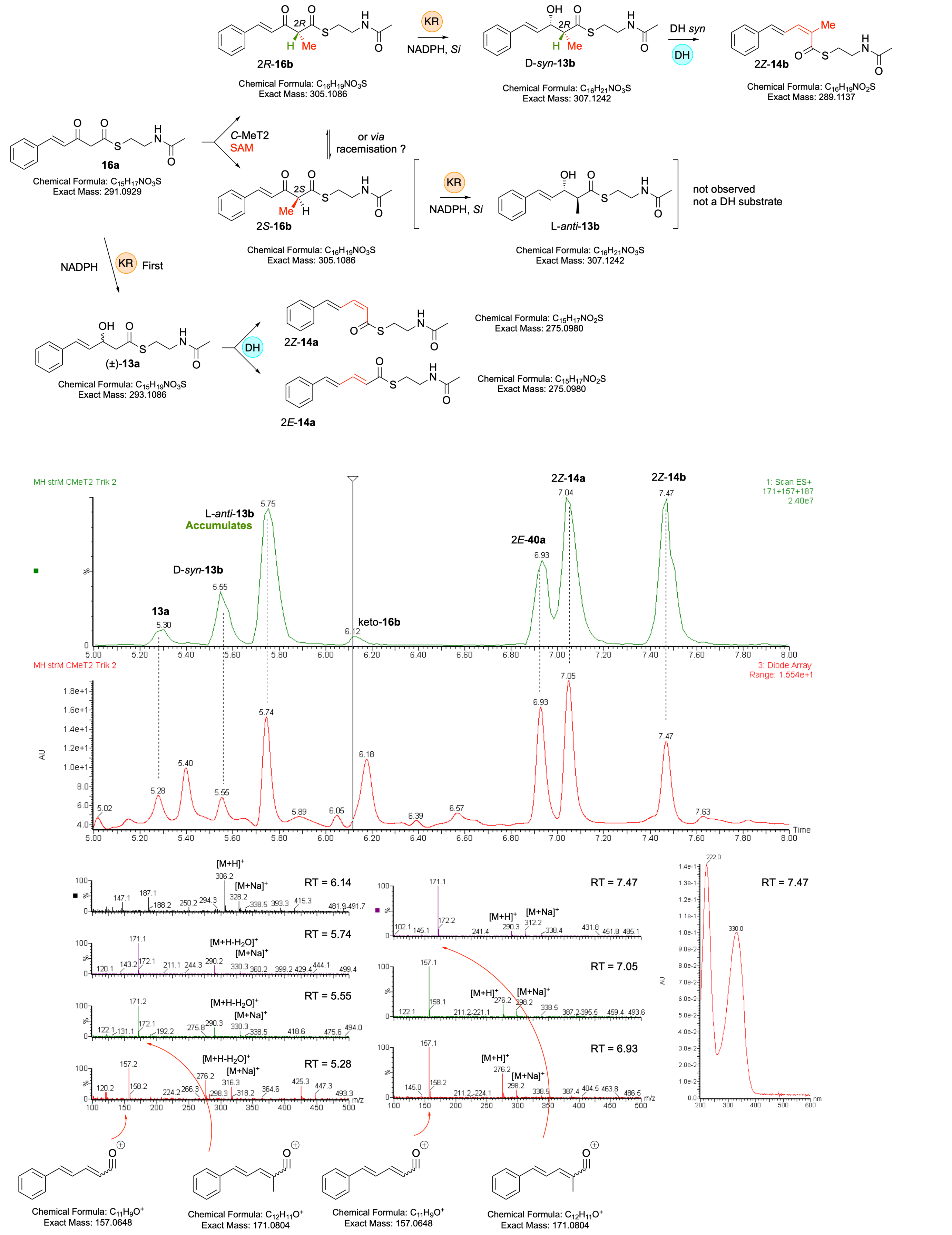

Figure S4.4.2. LCMS data corresponding to Scheme 7C. EIC ES+ chromatograms for enzyme reactions. Mass corresponds to expected product in each case, (EIC, ES+ at indicated masses corresponding to loss of SNAC for each species); ES$^+$ spectra of indicated product peaks, and uv of *Z*-**14b**. NB *E*-**14b** elutes at 7.40 min and has uv$_{max}$ of 334 nm.

4.4.3 Data for Scheme 7D - Epimerisation Assays

Experimental.

600 µL of each solution of each following combination was placed in an NMR tube at 21 ˚C and ^{1}H NMR data were collected with WATERGATE water suppression at t = 10 min and every 5 mins thereafter for 120 minutes. The integral between 1.25 and 1.27 was calculated for each spectrum and the integral plotted *vs* time. The first order rate constant was extracted by direct fit of the data to the integrated rate equation $[A] = [A]_{max} \times (1-e^{-kt})$ to find k and $[A]_{max}$.

Compound **17b** (70 µL, 38 mg/mL in D_2O/DMSO-d6) was placed in aqueous tris buffer (685 µL, pH 7.1, 50 mM Tris, 150 mM NaCl) in H_2O and DMSO-d6 (45 µL). The mixture was carefully mixed and 600 µL was transferred into an NMR tube.

Compound **17b** (70 µL, 38 mg/mL in D_2O/DMSO-d6) was placed in aqueous tris buffer (645 µL, pH 7.1, 50 mM Tris, 150 mM NaCl) in H_2O, StrM (40 µL, 53 mg/mL in tris) and DMSO-d6 (45 µL). The mixture was carefully mixed and 600 µL was transferred into an NMR tube.

Compound **17b** (70 µL, 38 mg/mL in D_2O/DMSO-d6) was placed in aqueous tris buffer (565 µL, pH 7.1, 50 mM Tris, 150 mM NaCl) in H_2O, StrM (40 µL, 53 mg/mL in tris), $NADP^+$ (80 µL, 100 mM in tris) and DMSO-d6 (45 µL). The mixture was carefully mixed and 600 µL was transferred into an NMR tube.

Compound **17b** (70 µL, 38 mg/mL in D_2O/DMSO-d6) was placed in aqueous tris buffer (665 µL, pH 7.1, 50 mM Tris, 150 mM NaCl) in H_2O, C-MeT2 (20 µL, 32 mg/mL in tris buffer) and DMSO-d6 (45 µL). The mixture was carefully mixed and 600 µL was transferred into an NMR tube.

Compound **17b** (70 µL, 38 mg/mL in D_2O/DMSO-d6) was placed in aqueous tris buffer (585 µL, pH 7.1, 50 mM Tris, 150 mM NaCl) in H_2O, CMeT2 (20 µL, 32 mg/mL in tris), SAH (80 µL, 100 mM in tris) and DMSO-d6 (45 µL). The mixture was carefully mixed and 600 µL was transferred into an NMR tube.

**Experiment**	***k* / min^{-1}**	**0.693 / *k* (half-life / min)**
**(±)-2-^{2}H-17b** alone	0.024	28.9
**(±)-2-^{2}H-17b** + StrM	0.026	26.7
**(±)-2-^{2}H-17b** + StrM + NADP+	0.023	30.1
**(±)-2-^{2}H-17b** + *C*-MeT2	0.027	25.7
**(±)-2-^{2}H-17b** + *C*-MeT2 + SAH	0.027	25.7

(±)-2-^{2}H-17b alone

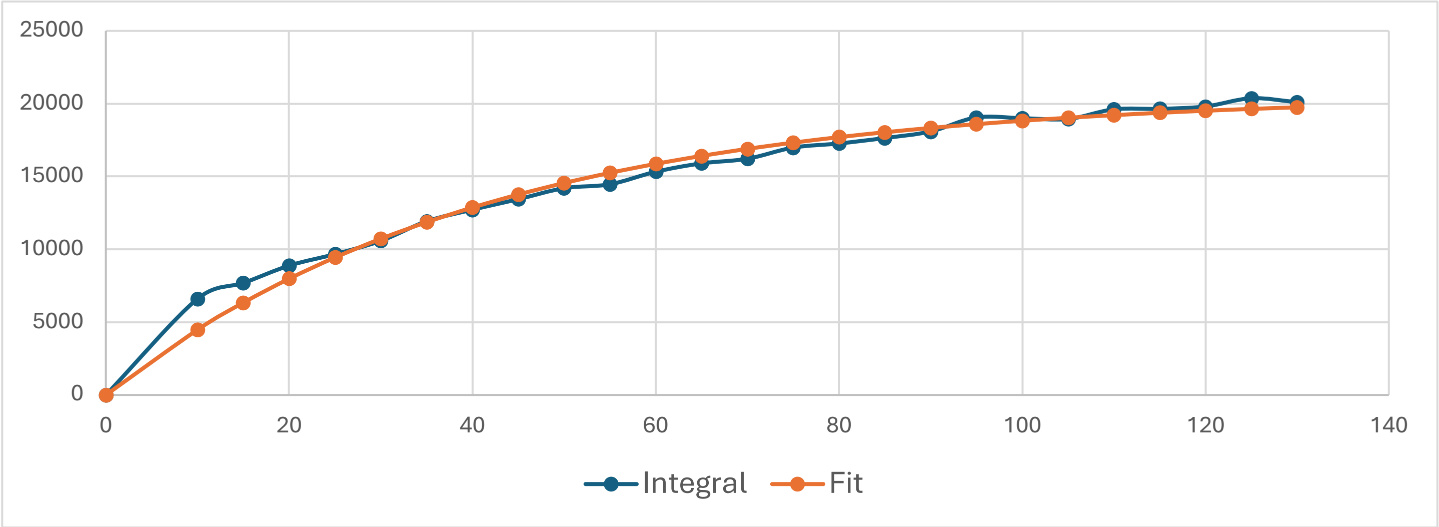

(±)-2-^{2}H-17b + StrM

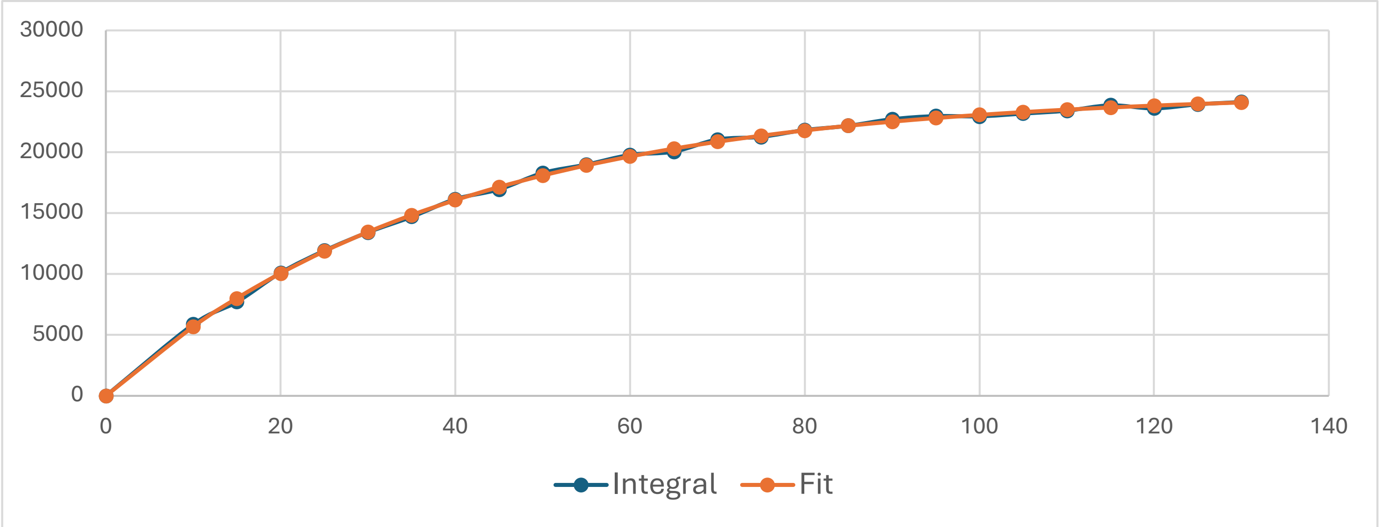

(±)-2-^{2}H-17b + StrM + NADP^{+}

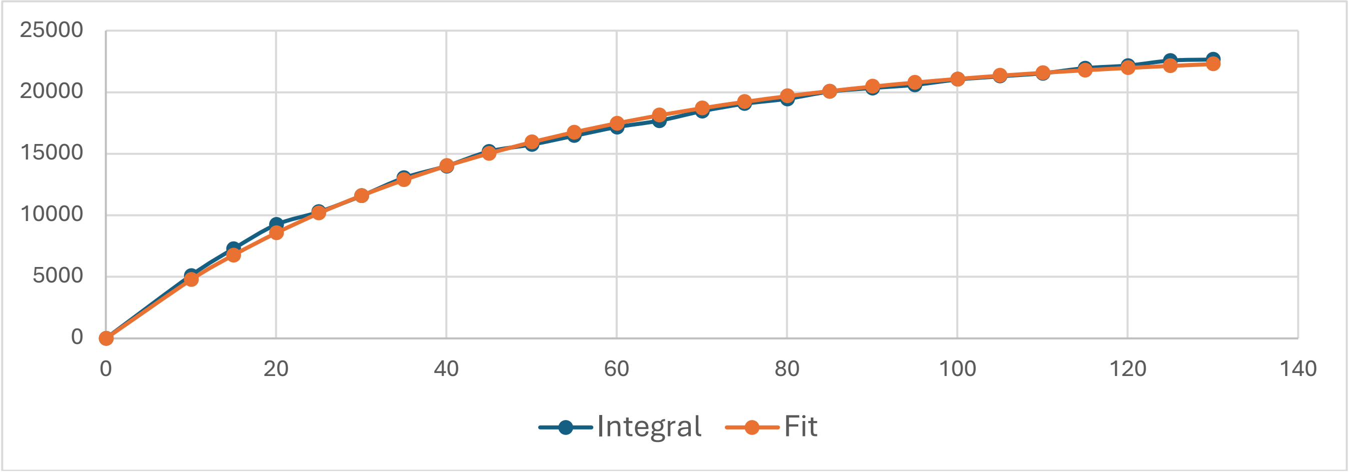

(±)-2-^{2}H-17b + *C*-MeT2

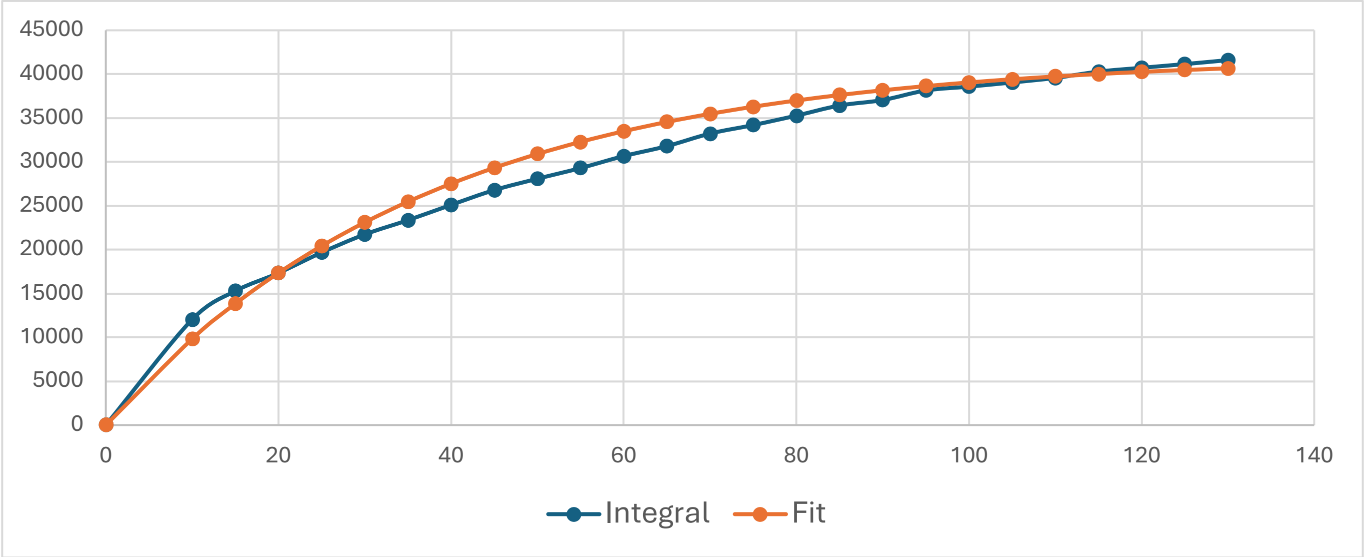

(±)-2-²H-17b + *C*-MeT2 + SAH

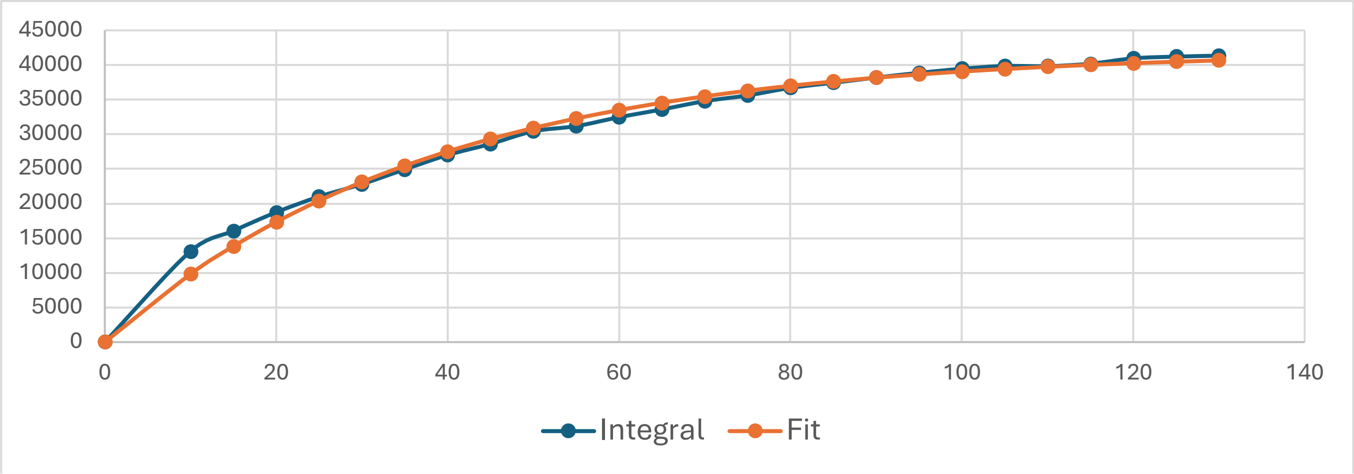

4.5 LCMS Data for Assays with Selected Mutant Proteins

4.5.1 KR Mutant A1943R

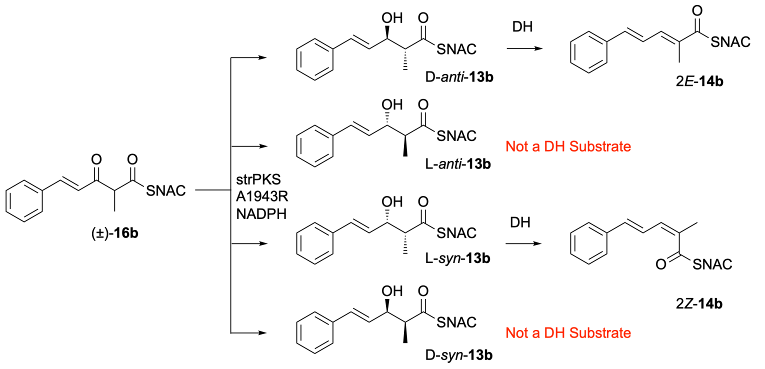

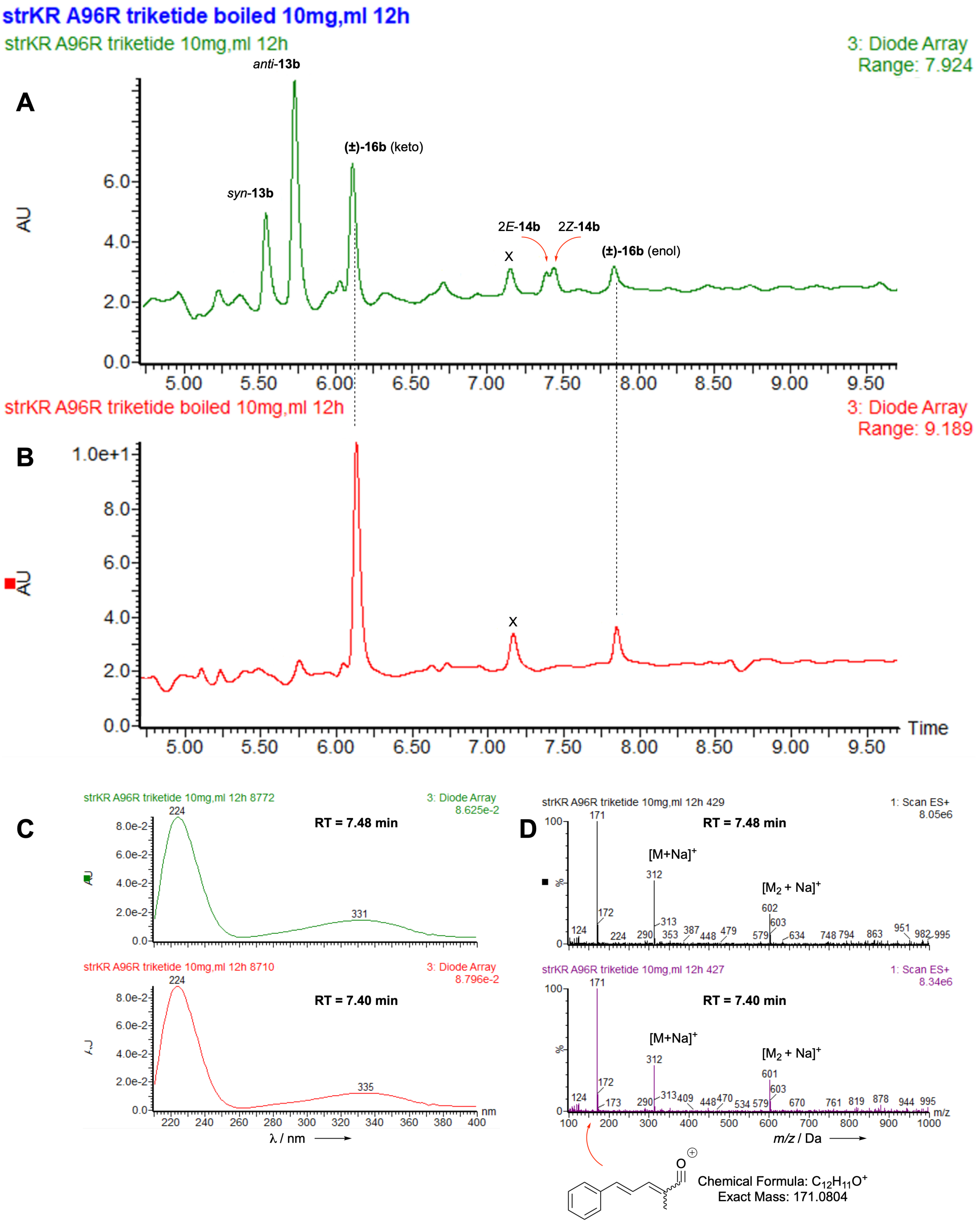

Figure S4.5.1. Reactions catalysed by strPKS A1943R: A, uv chromatogram trace of (±)-**16b** + strPKS(A1943R) + NADPH (uv, DAD, 200 - 600 nm); **B**, same as A, using boiled enzyme; **C**, extracted uv traces at indicated retention times; **D**, extracted ES^{+} spectra at indicated retention times.

4.5.2 KR Mutant M1942L

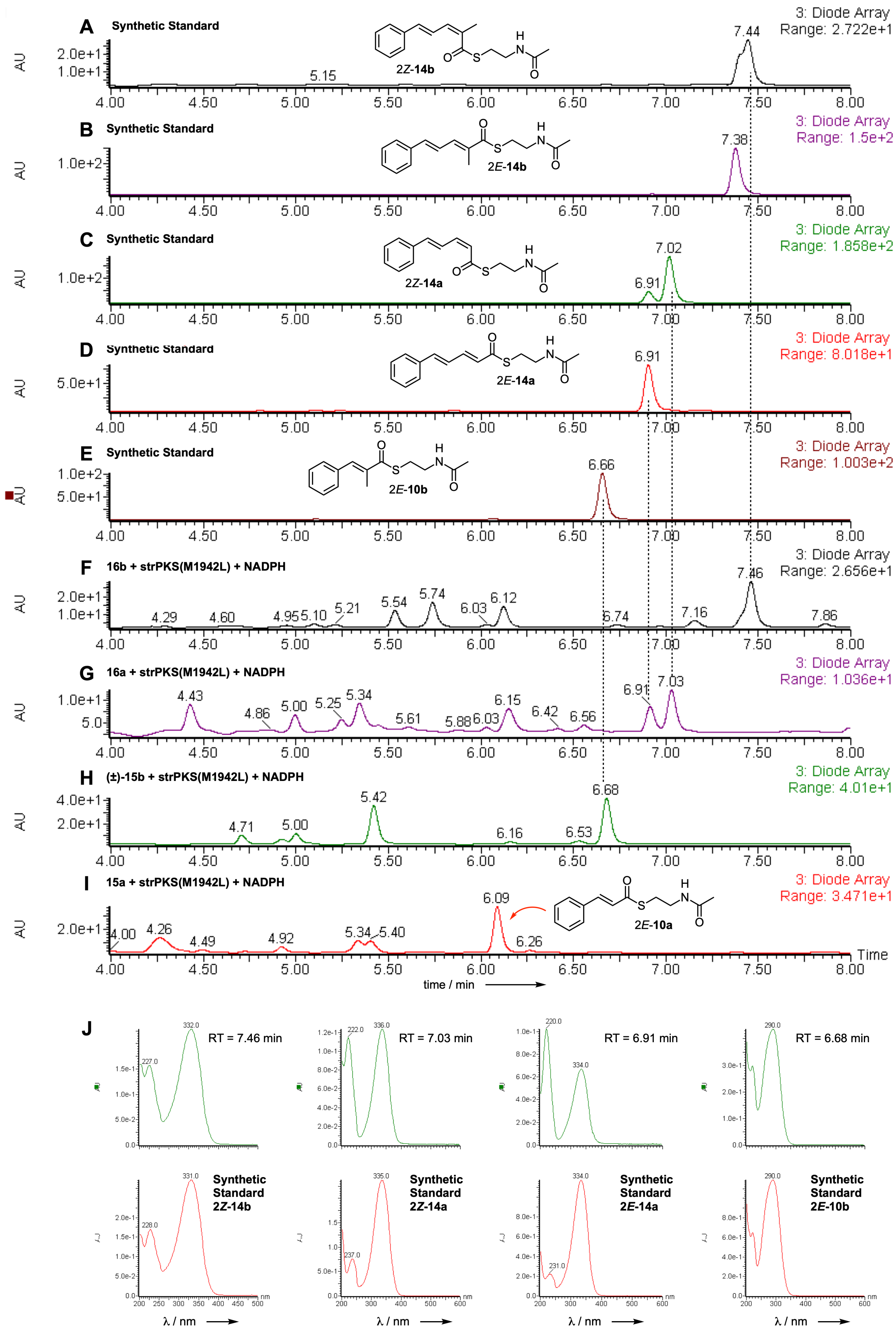

Figure S4.5.2. Reactions catalysed by strPKS A1943R. A-E, synthetic standards, uv chromatograms (DAD 200-600 nm); **F-I**, reactions as indicated uv chromatograms (DAD 200-600 nm); **J**, extracted uv spectra for indicated synthetic standards and selected peaks from chromatograms as indicated.

4.6 Inhibition of DH Reaction

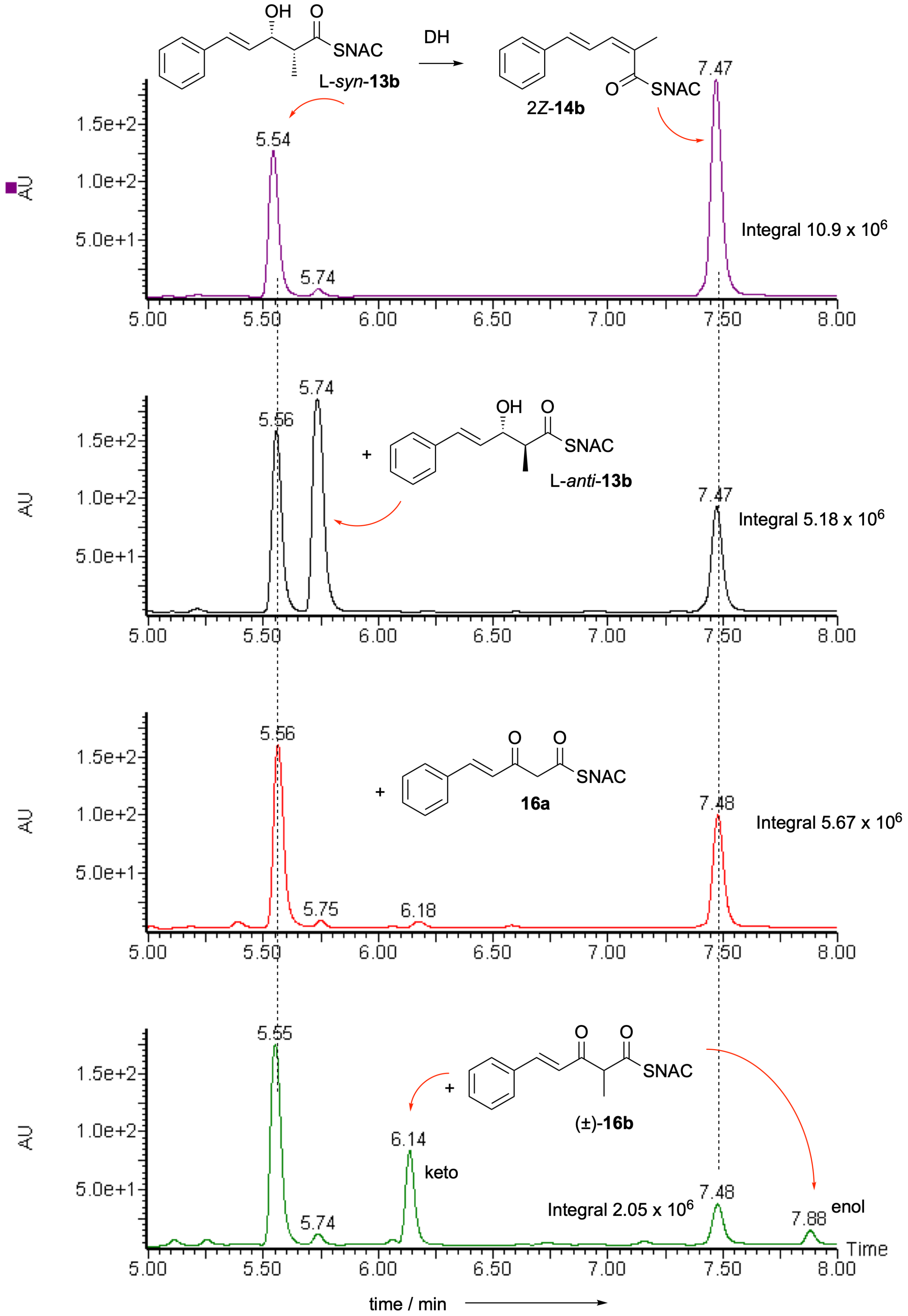

Figure S4.6.1. Apparent inhibition of DH reactions catalysed by strPKS multidomain in the presence of the indicated compounds. uv chromatograms of reactions catalysed by strPKS after 8h (uv, DAD, 200 - 600 nm).

4.7 Preparation, Characterisation and Use of 4'-^{2}H, 4'*R*-NADPH

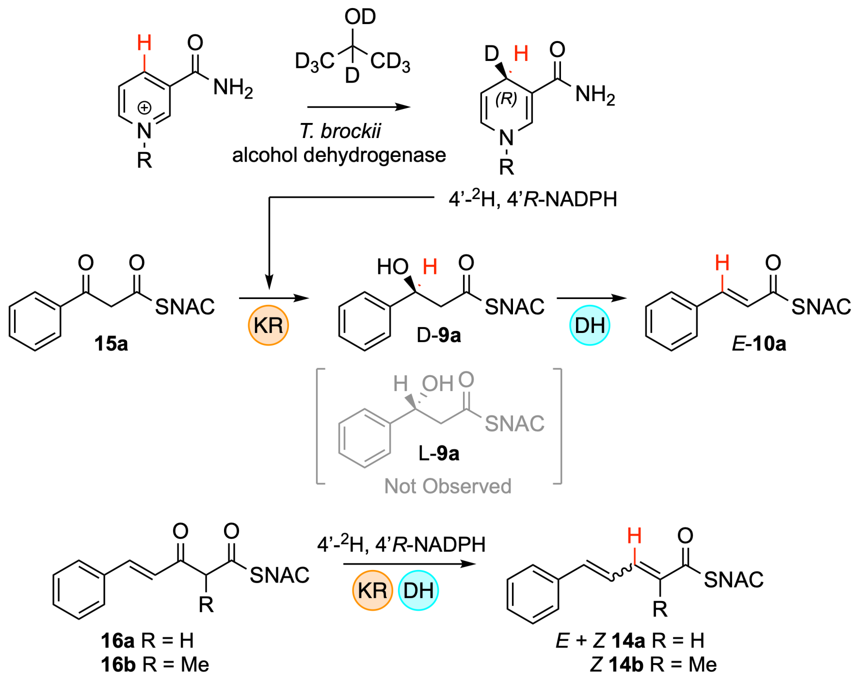

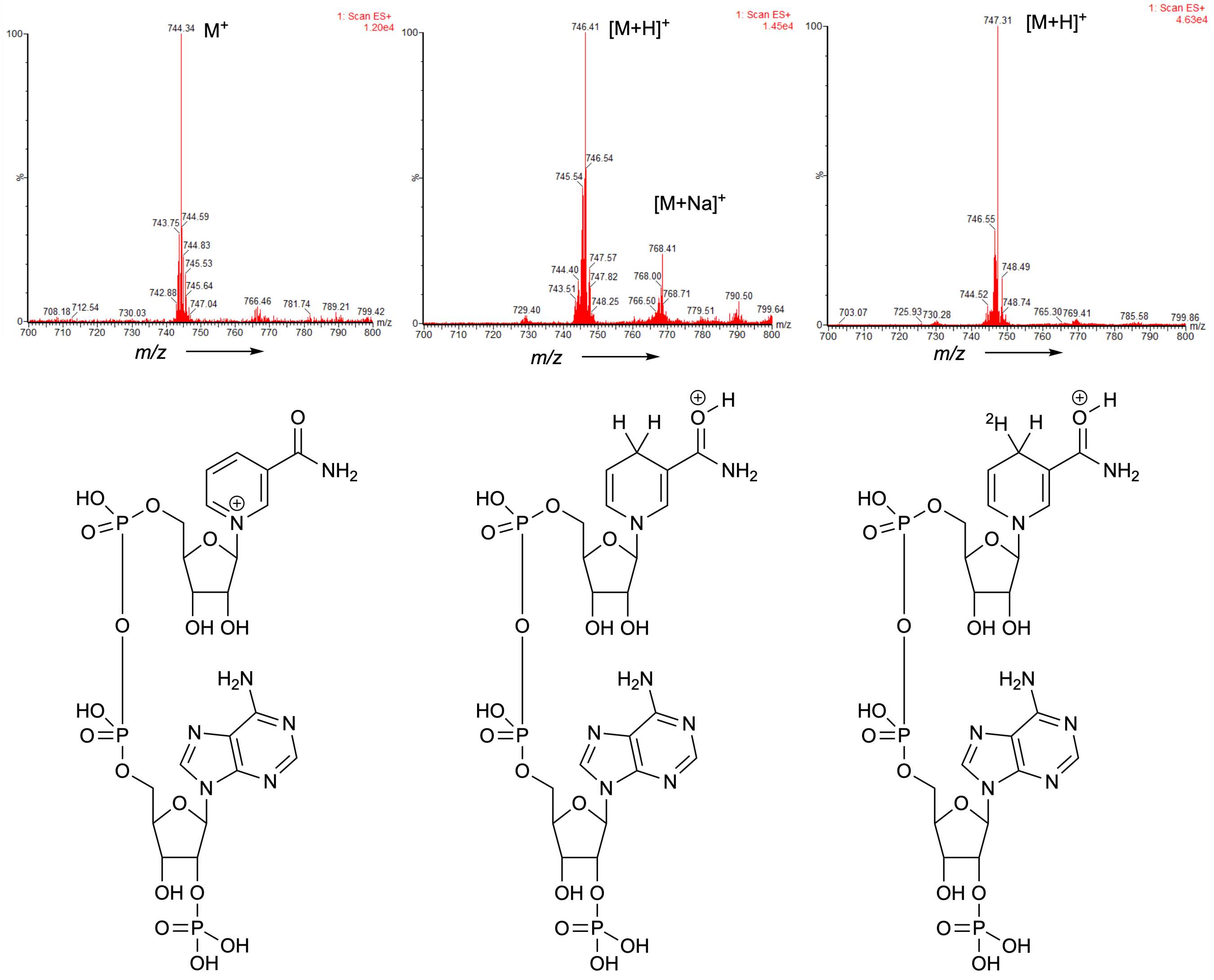

Chemical Formula: $C_{21}H_{29}N_7O_{17}P_3^+$
Exact Mass: 744.0827

Chemical Formula: $C_{21}H_{31}N_7O_{17}P_3^+$
Exact Mass: 746.0984

Chemical Formula: $C_{21}H_{30}DN_7O_{17}P_3^+$
Exact Mass: 747.1047

Figure S4.7.1. ESMS analysis of 4'-^{2}H, 4'*R*-NADPH.

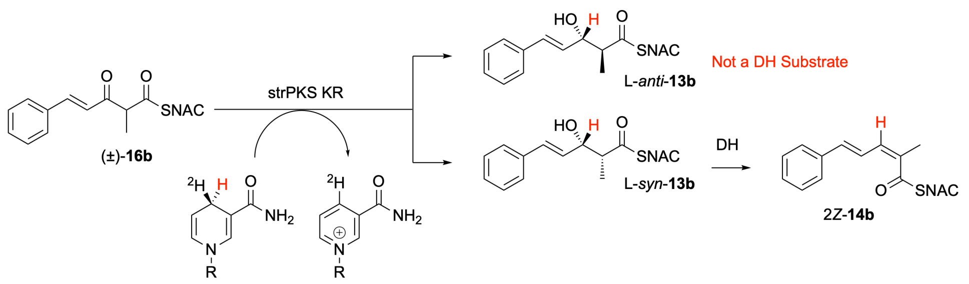

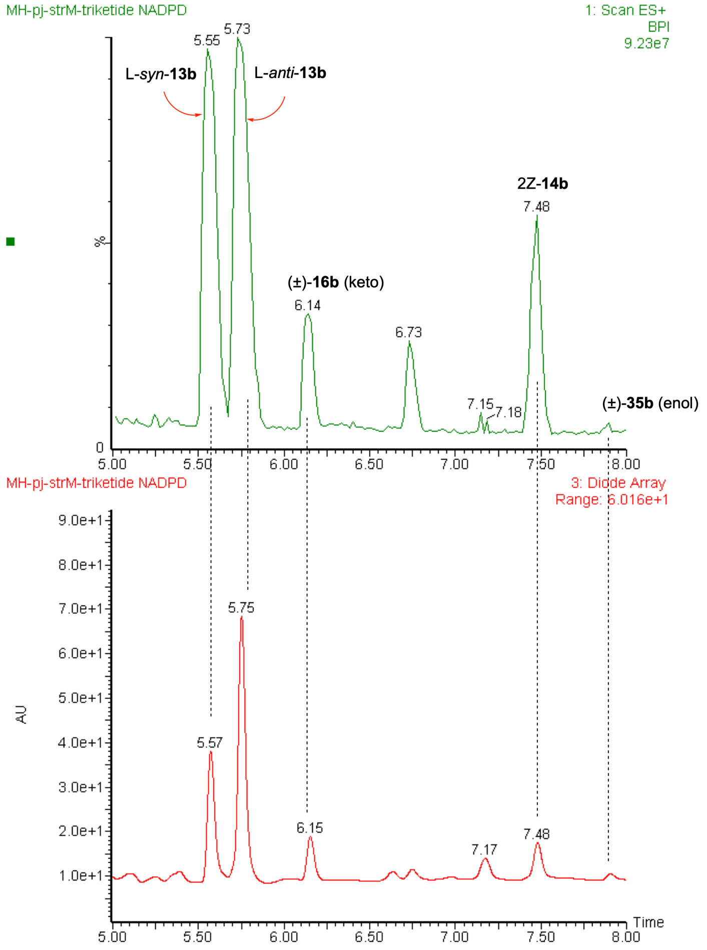

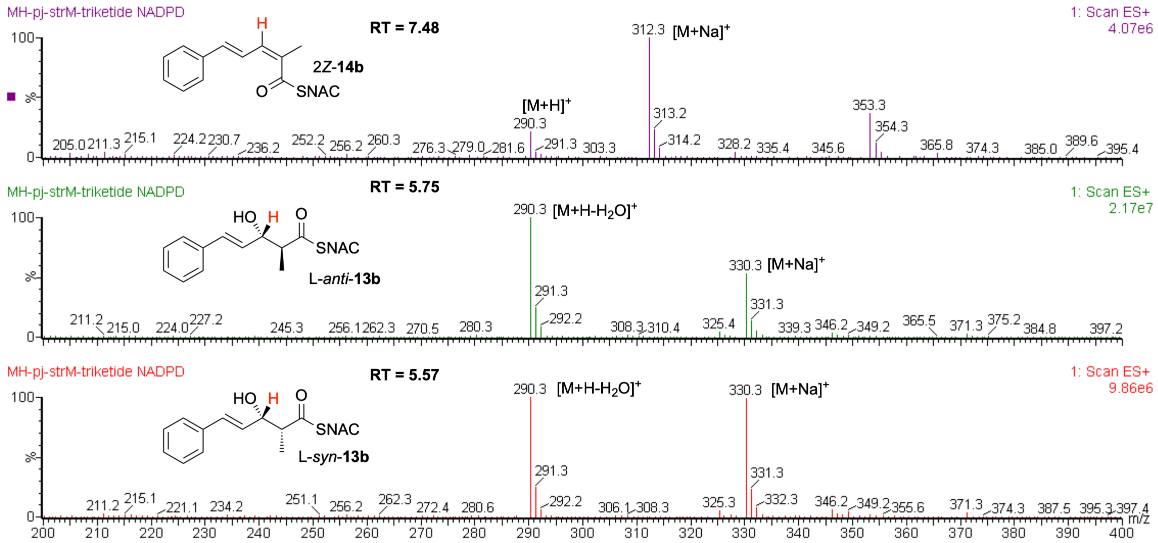

Figure S4.7.2. Reaction of 4'-^{2}H, 4'*R*-NADPH with (±)-16b.

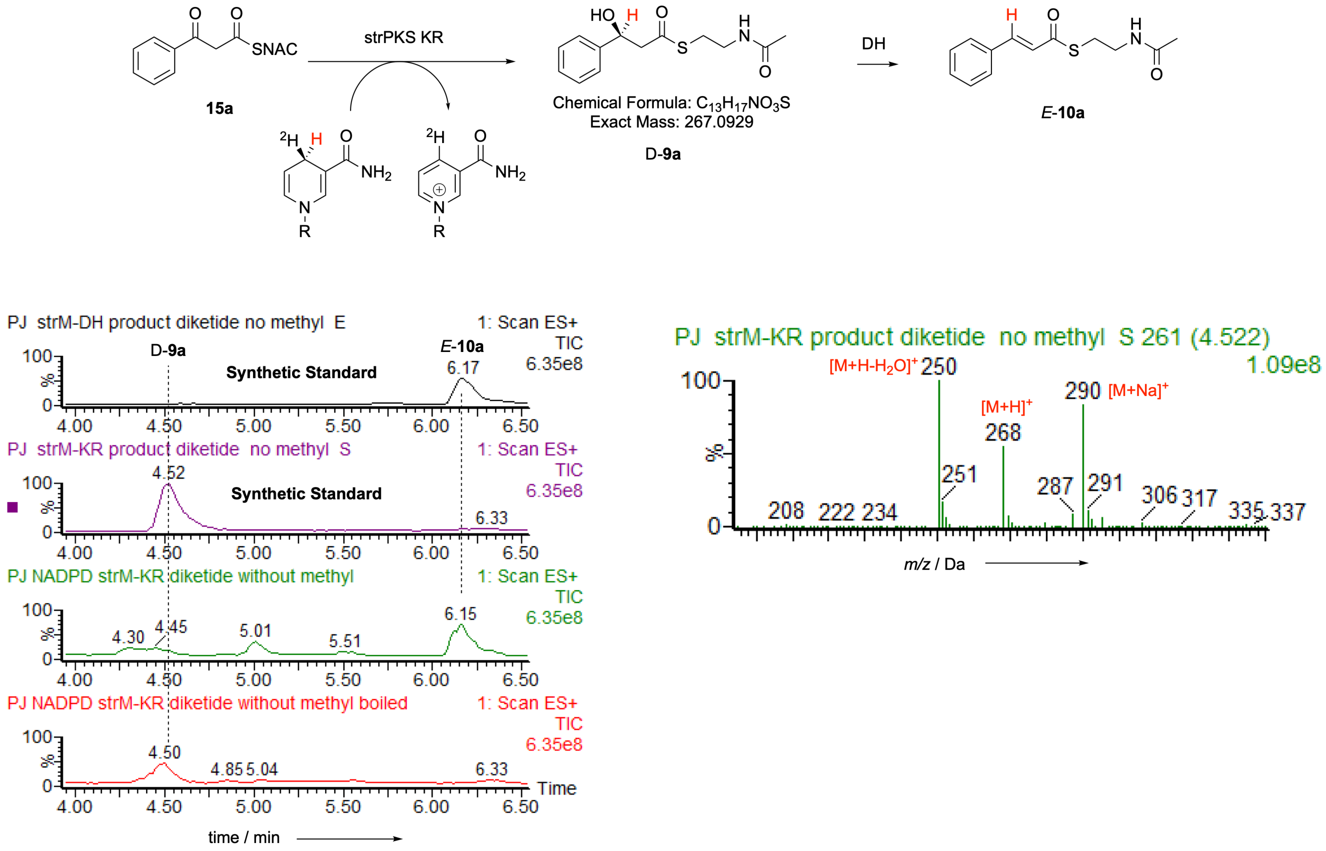

Figure S4.7.3. Reaction of 4'-^{2}H, 4'*R*-NADPH with 15b.

4.8 Mosher's Analysis of KR Products

4.8.1 Experimental

For the derivatisation of the KR products the previous described assay was upscaled to a volume of 1 mL. NADPH (200 µL, 20 mM), **16b** (10 µL, 100 mM) and multidomain (152 µL, 37 mg/mL) were dissolved in tris buffer (638 µL, pH 8.0, 50 mM Tris, 150 mM NaCl) and allowed to incubate at 25 °C overnight. After completion the protein was precipitated with the addition of CH_3CN (1 mL) and centrifuged. The clear solution was extracted with CH_2Cl_2 (2 × 2mL), dried over $MgSO_4$, filtered and concentrated under a stream of nitrogen. The crude residue was dried under high vacuum prior the derivatisation. The crude assay residue was redissolved in CH_2Cl_2 (0.5 mL) and 2,6-lutidine (3 µL, 30.5 µmol) and (*S*)-(+)-*α*-Methoxy-*α*-trifluormethylphenylacetyl-chloride (Mosher acid chloride, 3 µL, 8.8 µmol) was added. The reaction vial was sealed and stirred overnight. Afterward, 80 µL of the solution was transferred into an LCMS vial, and the solvent was removed under a stream of nitrogen. The resulting crude residue was redissolved in CH_3CN (100 µL) and submitted to LCMS-analysis.

Each individual synthetic standard (D-*anti*-**13b**; L-*anti*-**13b**, L-*syn*-**13b** and D-*syn*-**13b;** 1.0 eq., 1 mg, 3.2 µmol) was dissolved in CH_2Cl_2 (0.5 mL) and 2,6-lutidine (9.5 eq., 3 µL, 30.5 µmol) and (*S*)-(+)-*α*-Methoxy-*α*-trifluormethylphenylacetyl-chloride (Mosher acid chloride, 2.6 eq., 3 µL, 8.8 µmol) was added. The reaction vial was sealed and stirred overnight. Afterward, 50 µL of each solution was transferred into an LCMS vial, and the solvent was removed under a stream of nitrogen. The resulting crude residue was redissolved in CH_3CN (100 µL) and submitted to LCMS-analysis. Additionally, 20 µL of each derivatised synthetic standard (CH_3CN solution) was pooled to access the diastereomeric mixture solution and submitted for LCMS-analysis.

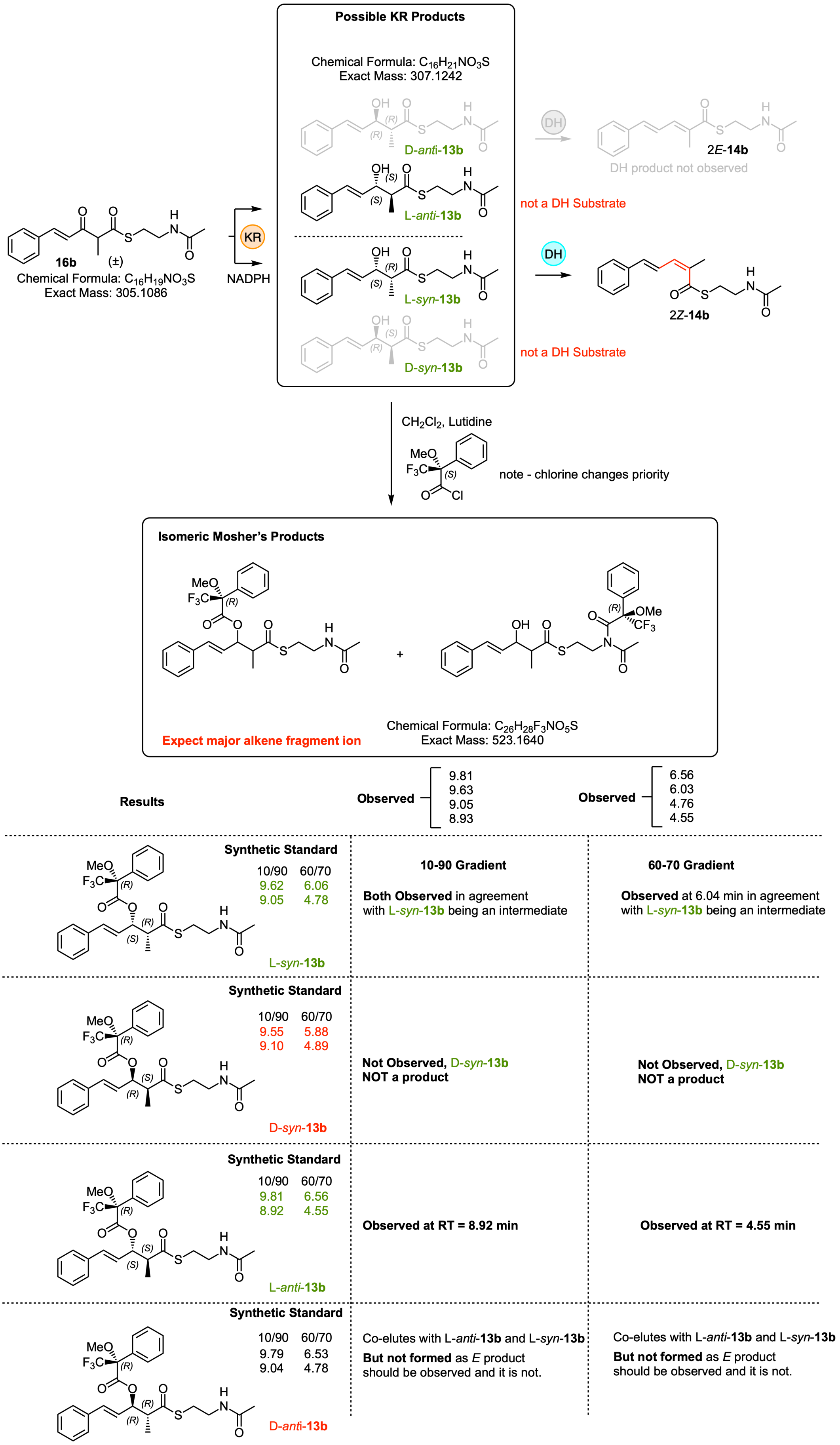

Figure S4.8.1. Analysis of Mosher's Derivatisation of 13b triketide alcohols produced by KR reactions of (±)-16b. L-*syn* diastereomer clearly observed in both gradients; D-*syn* diastereomer not observed in either conditions; L-*anti* diastereomer was observed in both gradients; D-*anti* diastereomer coelutes with L-*anti* and L-*syn* diastereomers under both gradients, but this *cannot* be produced by KR because the DH would convert it to *E*-**14b** and this is not observed.

4.8.2 Chromatograms

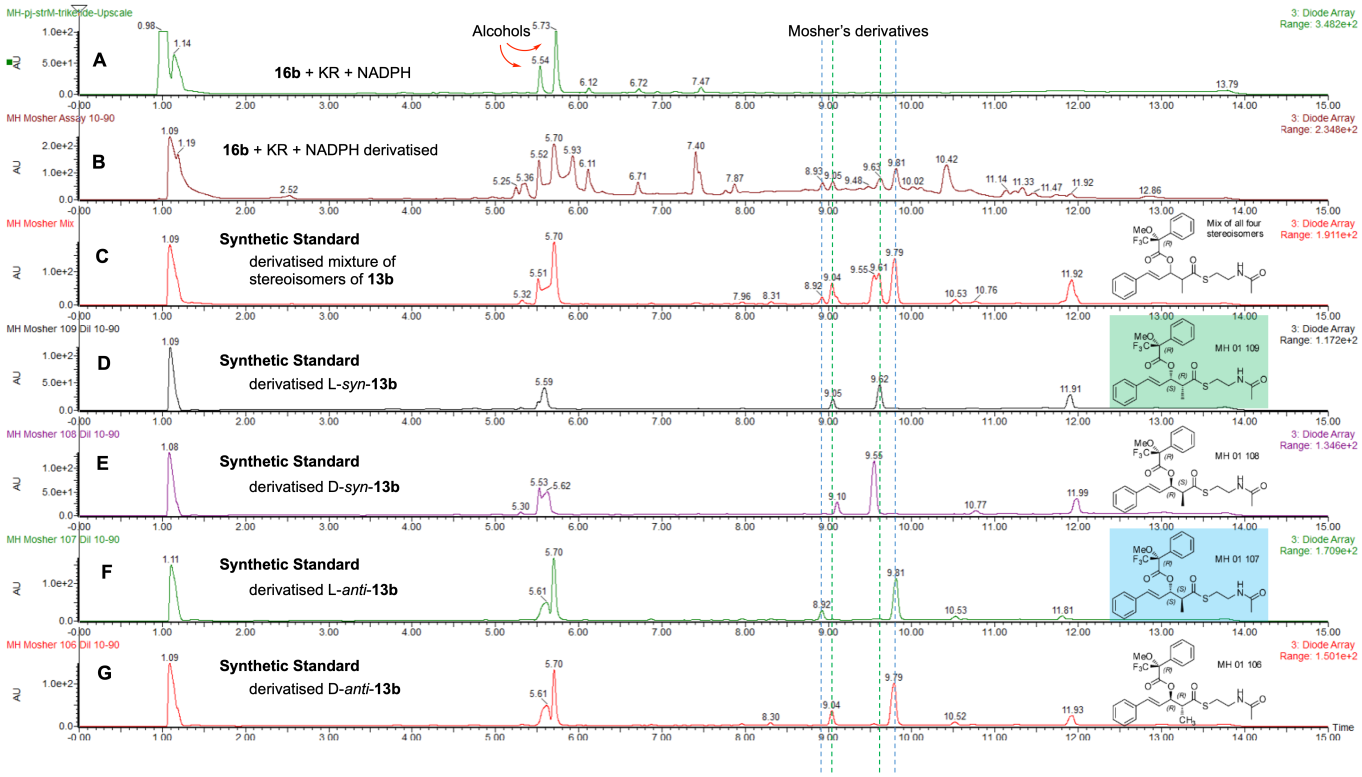

Figure S4.8.2A. LCMS analysis of Moshers derivatisation of KR products of (±)-**16b**, 10-90 gradient. **A**, Alcohol products of KR reaction(uv chromatograms (DAD 200 - 600 nm)); **B**, Derivatised products of KR reaction (uv chromatograms (DAD 200 - 600 nm)); **C-G**, synthetic standards as indicated (uv chromatograms (DAD 200 - 600 nm)).

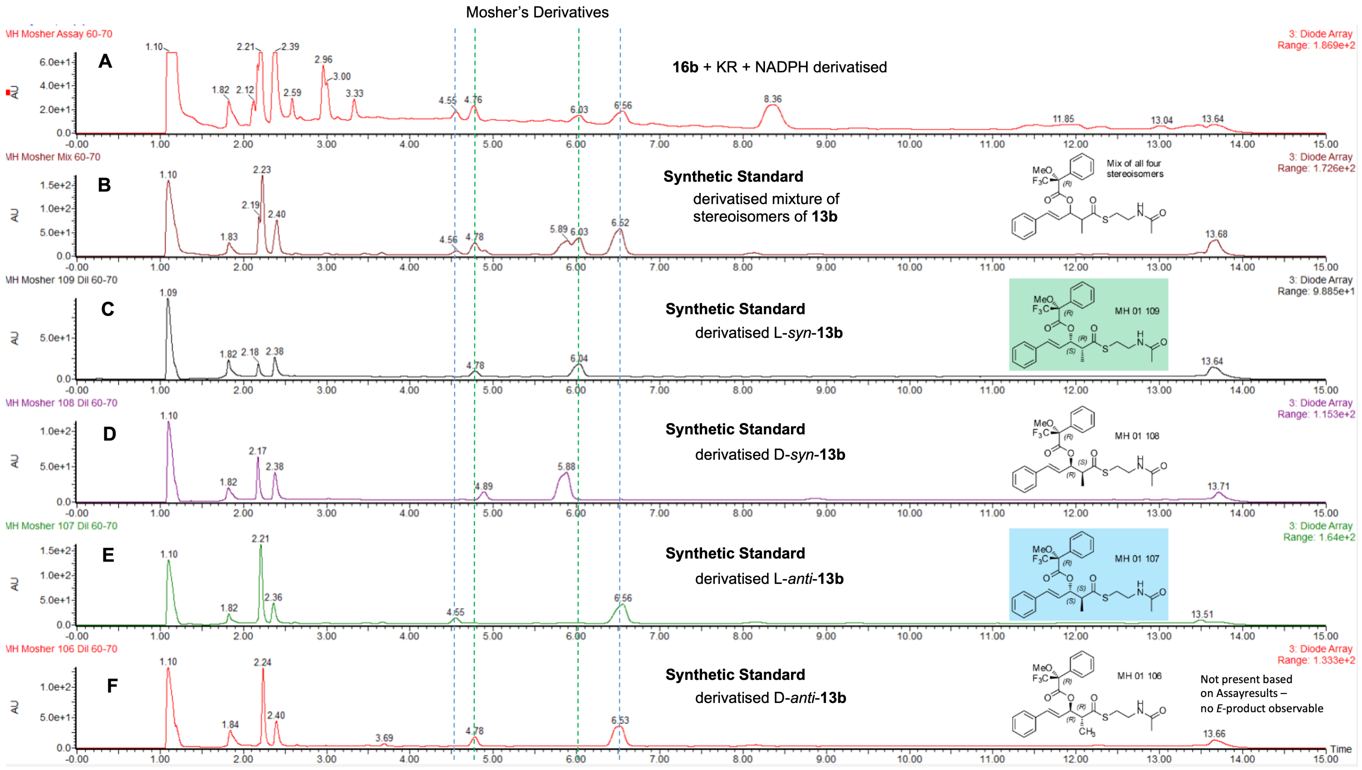

Figure S4.8.2B. LCMS analysis of Moshers derivatisation of KR products of (±)-**16b**, 60-70 gradient. **A**, Derivatised products of KR reaction (uv chromatograms (DAD 200 - 600 nm)); **B-F**, synthetic standards as indicated (uv chromatograms (DAD 200 - 600 nm)).

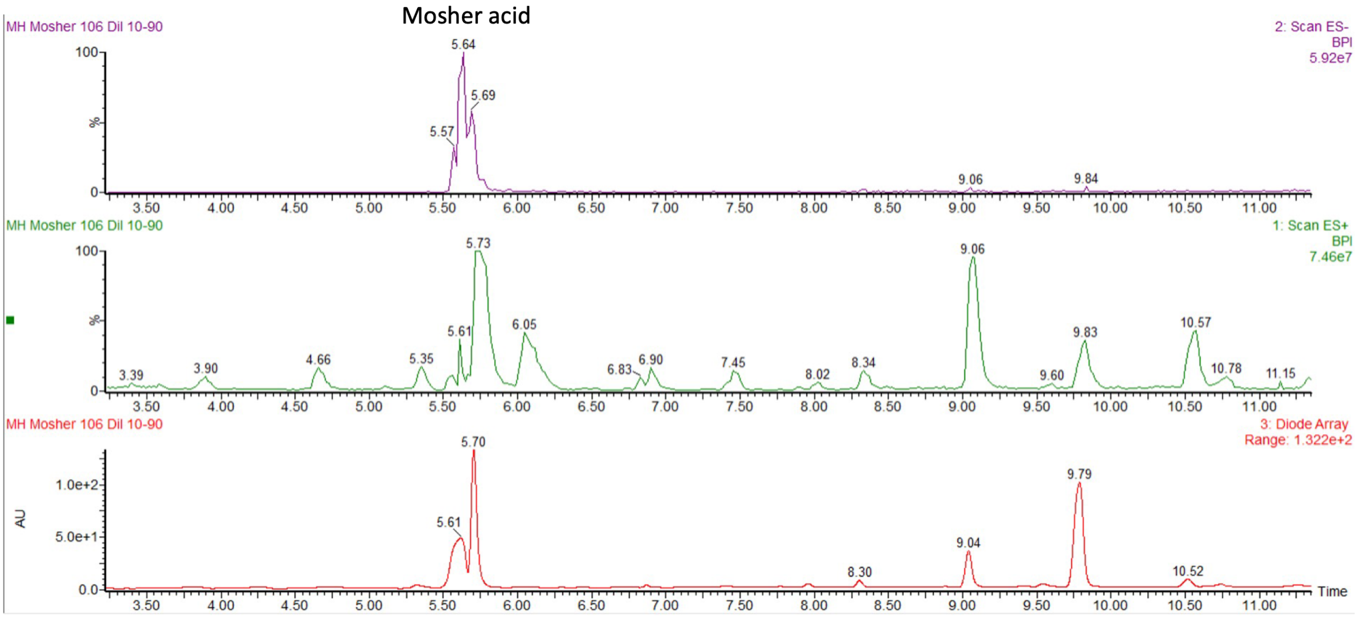

Figure S4.8.2C. Analysis of chromatogram of derivatised D-*anti*-**13b**.

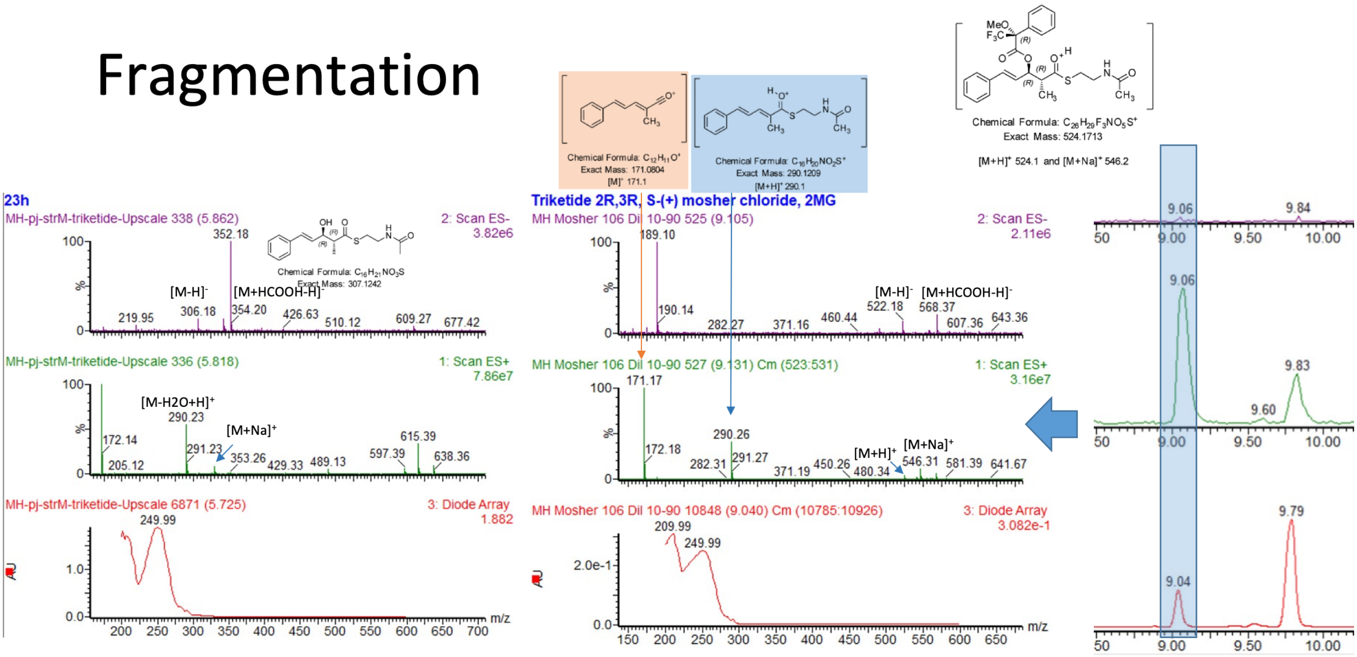

Figure S4.8.2D. Analysis of MS of peaks from derivatisation of D-*anti*-**13b**. 9.1 min peak likely to correspond to *O*-derivatised product due to presence of ion corresponding to elimination at 290.26 Da.

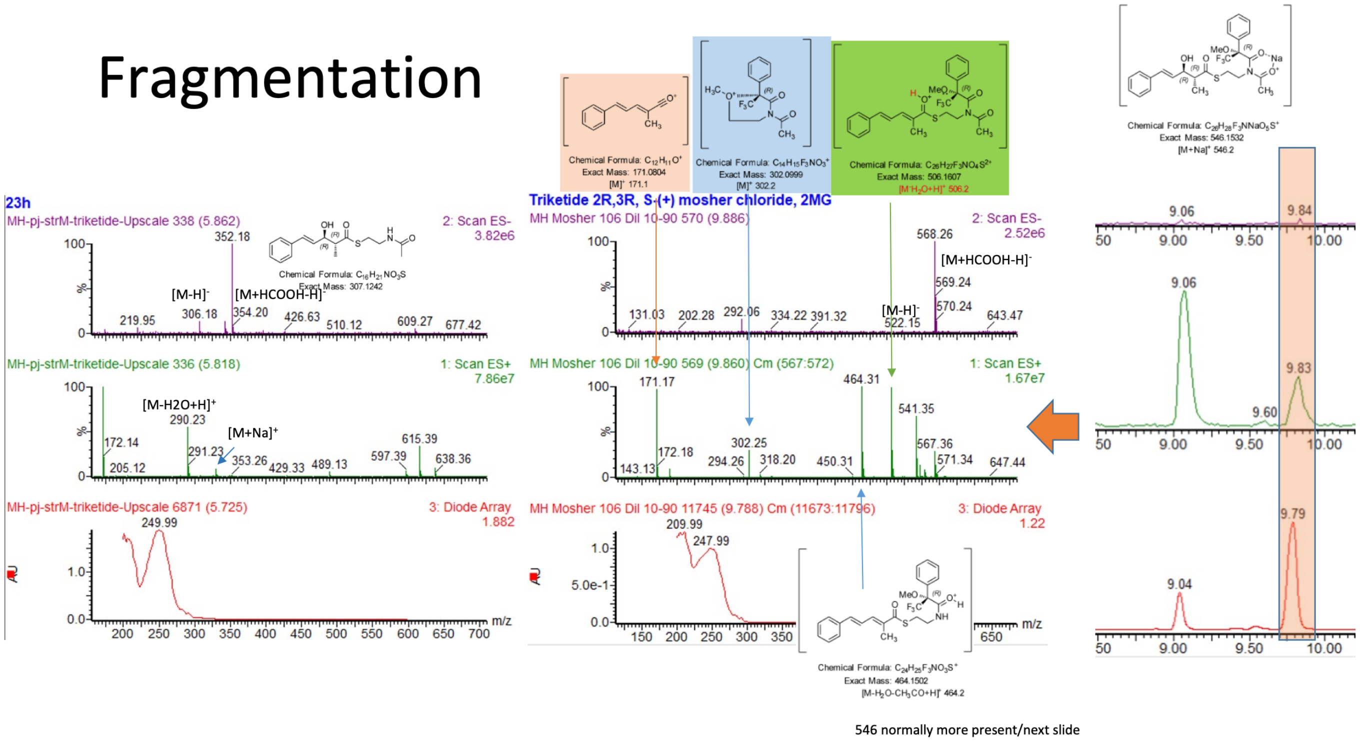

Figure S4.8.2E. Analysis of MS of peaks from derivatisation of D-*anti*-**13b**. 9.8 min peak likely to correspond to *N*-derivatised product due to ions corresponding to amide fragmentation.

5.0 Catalytic Domain Sequence Analysis

5.1 DH Alignment and Summary of Mutations

Sequence comparison of: EryDH4;[32] Curacin DH domains F,H,J and K;[33] Bongkrekic acid DH2;[34] Fostriecin DH1 and DH2;[35] the Gephyronic acid DH;[36] and the Gladiolin DH.[37] Red, conserved residues; yellow, residues known to be involved in catalysis; green and blue, highlighted residues.

```
                                                            H925A - Inactive
                                                            H925Q - Inactive

StrPKS DH     1         --------   ---LAVKMKRSrKGPLNYDTLAVNALTHPDLAEHVIKGEPILPATGFFEMIFEEGAR--------  54
Ery DH4       1    [24]ADVSALGV[4]HPLLLAAVDVP-GHGGAVFTGRLSTDEQPWLAEHVVGGRTLVPGSVLVDLALAAGEDVGLPV---  97
CurF DH       1         SNA-----   HPLLGEKINLAgIEDQHRFQSYIGAESPGYLNHHQVFGKVLFPSTGYLEIAASAGKSLFTSQ--E  66
CurH DH       1         SNASGQQV   HRLLGNKLELA-STGQTIYHQDINLNNHPWIGDHRVYDTPVIPGVSYIAMTLAAV--------gV  64
CurJ DH       1         SNASTTKL   HPLINQKFQSP-LSKEIFFESYFSTENLPFLADHIVYEQVVVPGASHISLLLAAASLTFAA---T  69
CurK DH       1         SNATAKNL   HPLLGEKLNLArIENQHHFQSYLTAESPAYLSQHQVFNKVLFPATGYLEIAAAVGKNLLTTG--E  71
Bon DH2       1    [25]QQPVAPWL   HPLV-HRNTSA--LDEQRFSTTLSGEEF-FLRDHLVQGRRVVPGVVQLEWARAAVALALGADalA  94
Fos DH1       1    [17]ADVTQAGL[4]HPLLAAAVELP-EGGGFVHTGRIGTLTHPWLADHAIHGTTLLPGTALLDLVLHAASDGAGEH---  90
Fos DH2       1    [20]TG-PGAGA[4]HPMLSQRTDLP-GGGGVLFSGRLAPGTDPWLPDHAVMGTLLLPGTGFVELALEAARAVGAGR---  92
GphF DH       1         SNATGERF   HPLLGRRVPDP-APGARCFTGTVSSDRPAYLGEHWVYDAIVVLGVTYLEMALAAASRLLPGNaaD  72
Gla DH13      1    [21]ENLYFQGL   DPFTMTHRHAA------SYELDFEHDNL-ILRDHRVHGVSILPGVTLIDVVYRLGQHLLGHQ---  84

                                       Implicated in gpfH DH isomerisation L1744

StrPKS DH    55    --TIWDIELRSLLPLLPEK-------VLNVNVKSDGHAWSIVSSSGGRNP----     -RLHATGFMTTEVMDKDA--GP  114
Ery DH4      98    ---LEELVLQRPLVLAGAG-AL-LRMSVGAPDESGRRTIDVHAAEDVADL---A[ 3]WSQHATGTLAQGVAAGPRdtE-  167
CurF DH      67    QVVVSDVDILQSLVIPETE-IKTVQTVVSFAENNSYKF-EIFSPSEGENQQTPQ     WVLHAQGKIYTEPTRNSQ--AK  138
CurH DH      65    PAAVEDINFQQPLFLAESNtTRETQLMLHTADNVGKQFVEVFSRDGAKQEE---     WQQHASMSVSENPP---P--PP  132
CurJ DH      70    ECQIEDILFPQALAIPEQG-VRTVQVVLTPQ-NNSFSF-QVISFDDSLESQINQ[10]WAVHATGKLSVANA--EQ--SL  148
CurK DH      72    QVVVSDVTIVRGLVIPETD-IKTVQTVISTLENNSYKL-EIFSTSEGDNQQANQ     WTLHAEGKIFLDSTTNTK--AK  143
Bon DH2      95    PLRLEQVSWMRPIVV-EGE--RELHIALSEDDSGRIGY-EIYGDEPGPTGEANG[ 2]-QVYSQGWAIVDASTRDDdaPR  167
Fos DH1      91    -PAVAELALQAPLVLPGER-GVDIRVTVQEADESGLRAFAVHSRPAPAGDDASG[ 3]WTRHASGALGPTEAPDAAdrA-  166
Fos DH2      93    ---VEELVLRAPMVFPGGR-ARDLQVWV-APDQGGERELLIRTR-TPGED----     WTLHATGVVTASRVDTDGftPD  158
GphF DH      73    TLIVEDVTVWSPLVLRAGA-PARLRLRSEDE-----RF-EIHSAEPERSDDESA     WTRHATGRIARRQL-SPD--AT  138
Gla DH13     85    RFELAQLLFRLPLATSGHL-ARRMTVRFAPGADHGCWTVSLSSVPLRSGVPGTG[ 1]-DLHAECVLRELDAEDLR---D  156

                                                 This Y (1856) implicated in isomerisation in gpfH DH
                                                      F1036Y - no change in activity for strPKS
                                                      F1036A - Dead
                                                   G           Add 1034G - same/better as WT

StrPKS DH   115    IDLAAIRARTTPADI   -SNLYAILNNT-AAFGPLYRRIEACYEGDHEILYQVRGNAPELTAHYN---YVFHPSLLDSC  186
Ery DH4     168    ----QWPPEDAVRIP   LDDHYDGLAEQGYEYGPSFQALRAAWRKDDSVYAEVSIAADE-------egYAFHPVLLDAV  233
CurF DH     139    IDLEKYQAECSQAIE   IEEHYREYRSKGIDYGSSFQGIKQLWKGQGKALGEMAFPEELTAQLAD---YQLHPALLDAA  212
CurH DH     133    TLSVDIPALCEQLRP[5]LTEIYASIS---LVYGPMLQAVRQAWIGEETSLLEIEVPKALAFQLAG---EPIHPVLIDAC  208
CurJ DH     149    IPLEEIQARCSQKID   SAEIYQHLWDRQIHLGQSFRWIEQVWLGEGEVLCQMKVPKTIL-NTTK---YQLHPTLVDSC  221
CurK DH     144    IDLEQYQRECSQVID   IQQHYQQFKSRGIDYGNSFQGIKQLWKGQGKALGKIALPEEIAGQATD---YQLHPALLDAA  217
Bon DH2     168    LDLEALRARCVEAHD   VDACYARFEAAGLLYGPSFRVLGELRSGEGIALGRLDASRRVGADLSDtrgFGWLPMLLDGA  244
Fos DH1     167    ---PQWPPADAAPVD   LTDLYPALALTGYEYGPDFRLLTAAWRTDDDVFAQVELGDDAAASDDV-drFSVHPALLDAS  239
Fos DH2     159    WTGAVWPPAGAEQIP   GDTFYPDLAERGYEYGPAFRSVKALWRRGDDLFAEVVLPEDQPYG------FGAHPALLDAS  229
GphF DH     139    GQLPRLDGE---AVE   LDAYYERMR---IYYGPRLRNIRHLERRGREAIGHVCLQGEEAQESAS---YELHPALLDAC  206
Gla DH13    157    PAEADFDVAGFIASA[5]VDEVYRGVRELGVVHGPFMQTLGEIFHRGDEELMRLSLGPLAESLRER---FHAHPALLDGA  235

                                                      H158 here implicated in gla vinylogous DH rxn

                    H1082Q - Dead
                    H1082A - Dead
                    H1082F - Dead  H1082F/F1036H - Dead

StrPKS DH   187    IHGLLHPV   FTGNADKSVFYLPSHIGRVTLYDRAIEEaVPETLYSyVVPHDWTP--DSIACDAFIVNERGERLVTLID  261
Ery DH4     234    AQTLSLGA   ---LGEPGGGKLPFAWNTVTLHASGATS---VRVVAtPAGAD--------AMALRVTDPAGHLVATVDS  296
CurF DH     213    FQ-IVSYA   IPHTET-DKIYLPVGVEKFKLYRQTISQ-VWAIAEI--RQT-------NLTANIFLVDNQGTVLVELEG  277
CurH DH     209    TR-LTPDL   FDFSSDSGVFWAPWRVKEMTLSHPTPSR-FYAYVEE-PSRVNEQL--QTRSYDIQLLDETGQAFGRING  280
CurJ DH     222    FQSIIALV   LDQSGNKNETFVPFSIDKFTFYNSSDNDlLWCYTCG--SKDKQSG--EKFKADIQLFDQHGQLVAQVIG  294
CurK DH     218    LQ-ILGHA   IGNTETDDKAYLPVGIDKLKQYRQTITQ-VWAIVEI--PEN-------TLKGSIKLVDNQGSLLAEIEG  283
Bon DH2     245    LQ----ST   LGLGWGDGRLALPFALESVSQWSALPEQ-GVAVVRE---AVDSGA--GLRKWDIEIADQAGRVALRIGG  311
Fos DH1     240    LHALLRSG[4]GVSGTDASGTLLPFSWGDVALHALGATA---LRVRFtRTGPT--------TVRVVASDPSGALILTAGE  309
Fos DH2     230    LHALP---[3]SFYETDDE-VRLPFSFGGVSLFATDVRR---VRVRL-RPRPE--------ATSVWITDAAGTPVLAMES  293
GphF DH     207    FQ-CVFAL   IYAHESHREPFVPLGCARIELRARGVRE-VRVHLRLhPPRSTDHN--QTHTADLRLFDMEGRLVASVDA  279
Gla DH13    236    TFAGSAFK[4]VAADFRDDRPHIPFSVERVRLLRPFPARiLVASRHGdKLGGAGAArrEVTSSDLRILDEEGRVLALFER  316

StrPKS DH   262    CVLSK---------         266
Ery DH4     297    LVVR-------STG[ 10]    313
CurF DH     278    LRVKVTEP------         285
CurH DH     281    FTVKRAPSQLFLK-         293
CurJ DH     295    FEGRKANPKILLMT         308
CurK DH     284    LRVTATTADALLK-         296
Bon DH2     312    VSTRAFDAGSAEQV[  2]    327
Fos DH1     310    LSLRPVVLDRLSDG[ 10]    333
Fos DH2     294    LILRAVERTQLQAA[  2]    309
GphF DH     280    LQLKRASKAALL--         291
Gla DH13    317    LSYKRVRQAADIVR[150]    480
```

CurH and CurJ - can do vinylogous elimination

E. coli FabA can catalyse alkene isomerisation

5.2 KR Alignment and Summary of Mutations

From Keatinge-Clay[38] - B1 type should have LDD in loop, no P in catalytic region;
A1 type should have no LDD in loop, W and no H in catalytic region.

All sequences obtained from NCBI and aligned with COBALT

```
   strPKS KR    1    ---------------------------ALADDKTYLVLGGIGSLGLQIAIWMYQKGARHIVLTSRTGVSRlagtknRSLR  53
   vFAS KR      1    -------------KGAKPKLMSAisKTFCPAHKSYIIAGGLGGFGLELAQWLIQRGVQKLVLTSRSGIRT--gyq----A  61
A1 Ery KR2     1    dqvavradavraRRLSPAHVTAT--SEYAVPGGTILVTGGTAGLGAEVARWLAGRGAEHLALVSRRGPDT------EGVG  72
A2 Amph KR1    1    ------------RRIVRASGDTRrkARSWKPRGTTLVTGGSGTLAPGLARHLAAQGAEHLVLLSRRGADA------PGAA  62
B1 Tyl KR1     1    ------------------AAAAG--AASWQPSGTVLITGGMGAIGRRLARRLAAEGAERLVLTSRRGPEA------PGAA  54
B2 Ery KR1     1    -----radgvygRRWVRAAAPAT--DDEWKPTGTVLVTGGTGGVGGQIARWLARRGAPHLLLVSRSGPDA------DGAG  67

                                                                      ** LOOP  **
                                                                           M1942L - no change in activity
                                                                           A1943R - More B-like

   strPKS KR   54    GAVEYLKTLpDLELRLEPCDASSEESLSKLISS---LDRPLAGAMLTAAVMADGLFLKQSADTYPIPFKPKTDAYFAFEK  130
   vFAS KR     62    KQVRRWRRQ-GVQVQVSTSNISSLEGARGLIAE-AAQLGPVGGVFNLAVVLRDGLLENQTPEFFQDVCKPKYSGTLNLDR  139
A1 Ery KR2    73    DLTAELTRL-GARVSVHACDVSSREPVRELVHGlIEQGDVVRGVVHAAGLPQQVAINDMDEAAFDEVVAAKAGGAVHLDE  151
A2 Amph KR1   63    ELAAELQAA-GTEVRFAACDITDPDAVAALLADlKAEGRTVRTVVHTAAVIELAALADTTVDAFADVVHAKVTGARILDE  141
B1 Tyl KR1    55    ELAEELRGH-GCEVVHAACDVAERDALAALVTA-Y----PPNAVFHTAGILDDAVIDTLSPESFETVRGAKVCGAELLHQ  128
B2 Ery KR1    68    ELVAELEAL-GARTTVAACDVTDRESVRELLGG-IGDDVPLSAVFHAAATLDDGTVDTLTGERIERASRAKVLGARNLHE  145

                                         ** Catalytic      **
                                              F1990W - completely inactive

   strPKS KR  131    VVDIK--KLDFLLAVSSVAG-FGAAGQTNYASANTGIEYLTARYpNAWSFVAPGIADSNVGFDLFTSTNSHLEQWESSTM  207
   vFAS KR    140    VTREAcpELDYFVVFSSVSCGRGNAGQSNYGFANSAMERICEKR-RHEGLPGLAVQWGAIGDVGILVETMSTNDTIVSGT  218
A1 Ery KR2   152    LCSDA----ELFLLFSSGAGVWGSARQGAYAAGNAFLDAFARHR-RGRGLPATSVAWGLWAAGGMTGDEEAVSFLRERGV  226
A2 Amph KR1  142    LLDDE--ELDDFVLYSSTAGMWGSGVHAAYVAGNAYLSALAEQR-RARGARATSIHWGKWPDD-LERELADPHQIRRSGL  217
B1 Tyl KR1   129    LTADI-kGLDAFVLFSSVTGTWGNAGQGAYAAANAALDALAERR-RAAGLPATSVAWGLWGGGGMAAGAGE-ESLSRRGL  205
B2 Ery KR1   146    LTREL--DLTAFVLFSSFASAFGAPGLGGYAPGNAYLDGLAQQR-RSDGLPATAVAWGTWAGSGMAEGPVA-DRFRRHGV  221

   strPKS KR  208    NSYEICLCLEdGLLRMANNERISIYVPNLNWDAISQSVS---ESVLYNHLVK----------------------------  256
   vFAS KR    219    LPQRMASCLE-VLDLFLNQPHMVLS-------------------------------------------------------  242
A1 Ery KR2   227    RAMPVPRALA-ALDRVLASGETAVVVTDVDWPAFAESYTAARPRPLLDRIVTTAP--SERAGEPET----ESlrdrLAGL  299
A2 Amph KR1  218    EYLDPELAMT-ALTRVMEDDETVIGLMDIDWGTYHDVFTAGRPSHLFDRIPEVARLLADRAAPAATavatSGlaarLQGV  296
B1 Tyl KR1   206    RAMDPDAAVD-ALLGAMGRNDVCVTVVDVDWERFAPATNAIRPGRLFDTVPEAREALTAA--------------------  264
B2 Ery KR1   222    IEMPPETACR-ALQNALDRAEVCPIVIDVRWDRFLLAYTAQRPTRLFDEIDDARRAAPQAAAEPRV----GA----LASL  292
```

5.3 *C*-MeT1 and *C*-MeT2 Alignment

Sequence comparison of the Curacin CMeT,[39] Pseurotin PKS CMeT,[40] and lovastatin PKS CMeT:[41] red, conserved residues; yellow, residues shown to be involved in catalysis; green, mutations in strPKS CMeT1.

C-MeT1 Analysis

```
CurJ MT      1     -----------MHHHHHHSSGVDLGTENLYFQSnalppDFLLDPVEVSQQLAPSLTELVTLLDNartsEIGTQLEELSVD  69
PsoF MT      1     -----------MLDGAEASNGVSKQT---------------VGGVHVTPEMLESV-------------QIPLEADKVGMT  41
LovB MT      1201  vdritlvpslcLATAESGCEKVAFNTINTYDKG-----DYLSGDIVVFDAEQTTLFQVENITFK------PFSPPDASTD  1269
strPKS MT1        --------------------------------------------------------------------------------

CurJ MT      70    YIVQGLlqmgWSYQPTESFDLD------AAAQCLGVVPTQVRLFERLLQILAEVGILQSNQQ-----QWQVQKTAQKVNP  138
PsoF MT      42    --------------PAEKSKLV------NAATAVYIDMAVEEMRSRGLAPKADYRV-----H-----WWKVM - --QDFVD  88
LovB MT      1270  HAMFAR----WSWGPLTPDSLLdnpeywATAQDKEAIPIIERIVYFYIRSFLSQLTLEERQQaafhlQKQIEWLEQVLAS  1345
strPKS MT1        ----------------------------------------------------WTGAVPTRPDtsy--EYIYQPLGLPAAE  26

CurJ MT      139   SKQSQSLLSQYPDEAATLTLLERCASQLS--GVLR--GEIDPVQLVFPQGDLTTATQLYKDSAVAKVMNT---------I  205
PsoF MT      89    SGEGQRVLQE---TSLTNQELERVIAKLGIEGEVI--ARMGPEIVNILTGKTHALAHIMRDDLLFRVYLSD------EGR  157
LovB MT      1346  AKEGRHLWYDPGWENDTEAQIEHLCTANSYHPHVRlvQRVGQHLLPTVRSNGNPFDLLDHDGLLTEFYTNTlsfgpaLHY  1425
strPKS MT1   27    LVKSEAQQQDYAFLDAIVAHADEKVAPPSANGHANg--------HANGSANGSAVGTVGEDRKVFEEIVQSiasdelELK  98
```

```
                                            SAM BINDING
CurJ MT      206   VEKVIMKAMEKLPPS--RGIRLLEIGAGTGGTTSYILPHLNPNQ----TEYIFTDIGALFTSKA---QEKFQDYRFLGYQ  276
PsoF MT      158   RANRYMAEYARLLTSqrRDIRILEIGAGTGGTTSEVLNLCSPNGesfcAEYMYTDLSPGFFNAAkttLKKWESHLA--FQ  235
LovB MT      1426  ARELVAQIAHRYQSM - ----DILEIGAGTGGATKYVLATPQLGF----NSYTYTDISTGFFEQAreqFAPFEDRMV--FE  1494
strPKS MT1   99    ASSILGLFSASLDAPvaAVRQILDHAAKSG---KQVVRILDIGD---aTASLYKQINAFASEYP----SLRVDYTACGHE  168

                                             missing active site NXXH
CurJ MT      277   --TLDIEVDP-------SSQGFESHRYDVIIAANVLHATTSLKQTLSHVRQLLAPGGILVLYEATTRS-----RWVDLIF  342
PsoF MT      236   --VLNIEDDP-------AGQGFKEHTYDLIIAANVIHATARLTNTLSNVHKLLKPGGVFGLVELTRLTPfynlTFGSL--  304
LovB MT      1495  --PLDIRRSP-------AEQGFEPHAYDLIIASNVLHATPDLEKTMAHARSLLKPGGQMVILEITHKEH----TRLGFIF  1561
strPKS MT1   169   haTLDLRLASynvdnvsKQAGLSPSTYDVIIETHTLGFAAELDRSLEYLHGLLLPGGFLVALEANGSAQasggKWIDQVF  248

CurJ MT      343   GLLEGWWKFTDyELRPDYPLLNREQWKKVLSETGFTQV------------------------------------------  380
PsoF MT      305   ---SGWWAGVD-EGRTESPLQSPQQWNSLLKQTGFSGVD-----------------------------------------  339
LovB MT      1562  GLFADWWAGVD-DGRCTEPFVSFDRWDAILKRVGFSGVDsrttdrdanlfptsvfsthaidatveyldaplassgtvkds  1640
strPKS MT1   249   S-PQGRWSGLR--SGKQHHRLSQSEWSGQLQKAKFQVVD-----------------------------------------  284
```

***C*-MeT2 Analysis**

```
CurJ MT      1     --------------------------------------XHHHHHHSSGVDLGTENLYFQSNAlPPDFLLDPVEVSQQLA-  41
PsoF MT      1     --------------------------------------MLDGAEASNGVSKQT---------------VGGVHVTPEMLE  27
LovB MT      1201  vdritlvpslclataesgcekvafntintydkgdylsgDIVVFDAEQTTLFQVENITFKPFS-PPDASTDHAMFARWSWG  1279
strPKS MT2   1     ---------------------------------------FEKPDFSRNLEFDW---------------VDP-HPVHQLAT  25

CurJ MT      42    ----PSLTELVTLLDNARTSEIGTQLEELSVDYIVQGLLQXGWSYQPTESFDLDAaaqCLGVVPTQVRLFERllqilaev  117
PsoF MT      28    SVQIPLEADKVGMTP-AEKSKLVNAATAVYIDMAVEEMRSRG----------------LAPKADYRVHWWK---------  81
LovB MT      1280  PLTPDSLLDNPEYWATAQDKEAIPIIERIVYFYIRSFLSQLTLEERQQAAFHLQK----------QIEWLEQ--------  1341
strPKS MT2   26    MIHNPAMNDLRALFK-ILDTKA--------LQVMADTISQNP----------------VVGSEISRQRLFE---------  71

CurJ MT      118   gILQSNQQQWQVQKTAQKVNPSKQS-QSLLSQYPDEAATLtLLERCASQLSGVLRGEIDPVQLVFPQGDLTtatQLYKDS  196
PsoF MT      82    -VMQDFVDSGEGQRVLQETSLTNQELERVIAKLGIEGEVI---ARMGPEIVNILTGKTHALAHIMRDDLLF---RVYLSD  154
LovB MT      1342  -VLASAKEGRHLWYDPGWENDTEAQIEHLCTANSYHPHVR-LVQRVGQHLLPTVRSNGNPFDLLDHDGLLT---EFYTNT  1416
strPKS MT2   72    -VCKEFV------RTQKHSTWTDEEYEHSKALFPTYFETT---ERISKVHPSIMESPAAAVGALYSDDMID---GFYRQN  138
```

```
                                                     SAM BINDING
CurJ MT      197   AVAKVXNTIVEKVIXKAXEK--LPPSRGIRLLEIGAGTGGTTSYILPHLN--PNQT----EYIFTDIGALFTSK-----A  263
PsoF MT      155   EGRRA-NRYMAEYARLLTS-----QRRDIRILEIGAGTGGTTSEVLNLCS--PNGESFCAEYMYTDLSPGFFN--AAKTT  224
LovB MT      1417  LSFGPALHYARELVAQIAHRY-----QSMDILEIGAGTGGATKYVLATPQ------LGFNSYTYTDISTGFFEQAREQFA  1485
strPKS MT2   139   KVFTSMNQEAAKTFKALVSSPdFGKQRPIRVLEVGAGVGGLTKFLVEALCdmPNAD---VEYTVTDLSYTLASSLAESFS  215

CurJ MT      264   --QEKFQDYRFlgYQTLDIEVDPSSQGFESHRYDVIIAANVLHATTSLKQTLSHVRQLLAPGGILVLYEATTRSR-----  336
PsoF MT      225   --LKKWESHLA--FQVLNIEDDPAGQGFKEHTYDLIIAANVIHATARLTNTLSNVHKLLKPGGVFGLVELTRLTPFYN--  298
LovB MT      1486  pfEDRMVFEPL------DIRRSPAEQGFEPHAYDLIIASNVLHATPDLEKTMAHARSLLKPGGQMVILEITHKEHTRL--  1557
strPKS MT2   216   --YKNMVAKMY------DLSKKPSEQGLQLGHYDVITGLNVIHAVPDLNATLTDLHSLLAPGGRILIVDTDGTARTSNpp  287

CurJ MT      337   -----WVDLIFGLLEGWWKFTDyELRPDYPLLNREQWKKVLSETGFTQVV------------------------------  381
PsoF MT      299   --------LTFGSLSGWWAGVD-EGRTESPLQSPQQWNSLLKQTGFSGVD------------------------------  339
LovB MT      1558  -------GFIFGLFADWWAGVD-DGRCTEPFVSFDRWDAILKRVGFSGVDsrttdrdanlfptsvfsthaidatveylda  1629
strPKS MT2   288   rpgaiWNDFIWGSFQGWFGYTD-D-RTHCTI-DEDEWRKRLTATGYSNVQ------------------------------  334
```

6. References

[1] R. Nofiani, K. de Mattos-Shipley, K. E. Lebe, L.-C. Han, Z. Iqbal, A. M. Bailey, C. L. Willis, T. J. Simpson, R. J. Cox, *Nature Communications* **2018**, *9*, 3940.

[2]. J. Klodmann, M. Senkler, C. Rode, H.-P. Braun, ""Defining the Protein Complex Proteome of Plant Mitochondria,"" *Plant Physiology* **2011**, *157*, 587-598.

[3] Bok, J.W., Keller, N.P. **(2012)**. Fast and Easy Method for Construction of Plasmid Vectors Using Modified Quick-Change Mutagenesis. In: Keller, N., Turner, G. (eds) Fungal Secondary Metabolism. Methods in Molecular Biology, vol 944. Humana Press, Totowa, NJ. https://doi.org/10.1007/978-1-62703-122-6_11

[4] J. Kim, K. A. D. Castro, M. Lim, H. Rhee, *Tetrahedron* **2010**, *66*, 3995-4001.

[5] G. E. Keck, D. F. Kachensky and E. J. Enholm, *J. Org. Chem.* 1985, 50, 22, 4317-4325

[6] S. Chang, S. Hur, R. Britton, *Chem. Eur. J.*, **2015**, *21(46)*, 16646-16653.

[7] E. Liddle, A. Scott, L.-C. Han, D. Ivison, T. J. Simpson, C. L. Willis, R. J. Cox, *Chem. Commun.,* **2017**, *53*, 1727-1730.

[8] K. Okamoto, A. Nanya, A. Eguchi, K. Ohe, *Angew. Chem. Int. Ed*. 2018, 57, 1039-1043

[9] K. Ishihara, T. Maruyama, M. Mouri, Q. Gao, K. Furuta, H. Yamamoto, *Bull. Chem. Soc. Japan*, **1993**, *66*(*11)*, 3483-3491.

[10] Z. Yin, D. Bär, B. Gust and J. Dickschat, *Org. Biomol. Chem*., **2022**, *20(46)*, 9103 - 9107.

[11] C. Le Sann, D. M. Muñoz, N. Saunders, T. J. Simpson, D. I. Smith, F. Soulas, P. Watts and C. L. Willis,*Org. Biomol. Chem***.**, 2005, **3**, 1719-1728.

[12] T. Murata, H. Tsutsui, and I. Shiina**,** *J. Org. Chem.,* **2024** *89*(21), 15414-15435**,** DOI: 10.1021/acs.joc.4c01140

[13] R. C. Harris, A. L. Cutter, K. J. Weissman, U. Hanefeld, M. C. Timoney and J. Staunton. *J. Chem. Res., Miniprint*, **1998**, 6, 1230 - 1247

[14] D. A. Evans, J. S. Tedrow, J. T. Shaw, C. W. Downey, *J. Am. Chem. Soc.* **2002**, *124*, 392-393.

[15] J. Merad, P. S. Grant, T. Stopka, J. Sabbatani, R. Meyrelles, A. Preinfalk, J. Matyasovsky, B. Maryasin, L. González, N. Maulide, *J. Am. Chem. Soc.* **2022**, *144*, 12536-12543.

[16] A. Suzuki, Y. Kamei, M. Yamashita, Y. Seino, Y. Yamaguchi, T. Yoshino, M. Kojima, S. Matsunaga, *Angew. Chem.* **2023**, *135*, DOI 10.1002/ange.202214433.

[17] D. A. Evans, D. L. Rieger, T. K. Jones, S. W. Kaldor, *J. Org. Chem.* **1990**, *55*, 6260-6268.

[18] X.-Y. Liu, X.-R. Li, C. Zhang, X.-Q. Chu, W. Rao, T.-P. Loh, Z.-L. Shen, *Org. Lett.* **2019**, *21*, 5873-5878.

[19] J. R. Clark, J. M. French, S. T. Diver, *J. Org. Chem.* **2012**, *77*, 1599-1604.

[20] L. C. Dias, L. G. de Oliveira, J. D. Vilcachagua, F. Nigsch, *J. Org. Chem.* **2005**, *70*, 2225-2234.

[21] https://doi.org/10.1021/jo047732k_enantiomere +78.6 in CHCl3 https://doi.org/10.1021/ja00216a026

[22] G. Sirasani, T. Paul, R. B. Andrade, *Tetrahedron,* **2011**, *67*, 2197-2205.

[23] D. A. Evans, S. L. Bender, J. Morris, *J. Am. Chem. Soc.* **1988**, *110*, 2506-2526.

[24] C. J. O'Brien, Z. S. Nixon, A. J. Holohan, S. R. Kunkel, J. L. Tellez, B. J. Doonan, E. E. Coyle, F. Lavigne, L. J. Kang, K. C. Przeworski, *Chem. A Eur. J.* **2013**, *19*, 15281-15289.

[25] K. Beautement, J. M. Clough, *Tetrahedron Lett* **1987**, *28*, 475-478.

[26] N. J. Foy, K. C. Forbes, A. M. Crooke, M. D. Gruber, J. S. Cannon, *Org. Lett.* **2018**, *20*, 5727-5731.

[27] A. Padwa, D. N. Kline, B. H. Norman, *J. Org. Chem.* **1989**, *54*, 810-817.

[28] A. K. Banerjee, D. Nasipuri, S. C. Pakrashi, *J. Org. Chem.* **1990**, *55*, 3952-3954.

[29] D. M. Roberts, C. Bartel, A. Scott, D. Ivison, T. J. Simpson, R. J. Cox, *Chem. Sci.* **2016**, *8*, 1116-1126.

[30] A. J. Hughes, A. Keatinge-Clay, *Chem. Biol.* **2011**, *18*, 165-176.

[31] M. Peretz, O. Bogin, E. Keinan, Y. Burstein, *Int. J. Pept. Protein Res.* **1993**, *42*, 490-495.

[32] C. R. Valenzano, Y.-O. You, A. Garg, A. Keatinge-Clay, C. Khosla, D. E. Cane, *J. Am. Chem. Soc.,* **2010**, *132*, 14697-14699.

[33] W. D. Fiers, G. J. Dodge, D. H. Sherman, J. L. Smith, C. C. Aldrich, *J. Am. Chem. Soc.,* **2016**, *138*, 16024-16036.

[34] X. Xie, D. E. Cane, *Biochemistry,* **2018**, *57*, 3126-3129.

[35] D. D. Shah, Y.-O. You, D. E. Cane, *J. Am. Chem. Soc.,* **2017**, *139*, 14322-14330.

[36] G. J. Dodge, D. Ronnow, R. E. Taylor, J. L. Smith, *ACS Chem. Biol.,* **2018**, *13*, 2699-2707.

[37] C. Hobson, M. Jenner, X. Jian, D. Griffiths, D. M. Roberts, M. Rey-Carrizo, G. L. Challis, *Nature Chem. Biol.,* **2022**, 1-7.

[38] A. T. Keatinge-Clay, *Chem. Biol.,* **2007**, *14*, 898-908.

[39] M. A. Skiba, A. P. Sikkema, W. D. Fiers, W. H. Gerwick, D. H. Sherman, C. C. Aldrich, J. L. Smith, *ACS Chem. Biol.,* **2016**, DOI 10.1021/acschembio.6b00759.

[40] S. Kishimoto, Y. Tsunematsu, T. Matsushita, K. Hara, H. Hashimoto, Y. Tang, K. Watanabe, *Biochemistry,* **2019**, *58*, 3933-3937.

[41] R. A. Cacho, J. Thuss, W. Xu, R. Sanichar, Z. Gao, A. Nguyen, J. C. Vederas, Y. Tang, *J. Am. Chem. Soc.,* **2015**, *137*, 15688-91.
